# Construction of axial chirality through addressing the *meta* constraint in the Catellani reaction

**DOI:** 10.1039/d6sc01871h

**Published:** 2026-05-19

**Authors:** Jin Ge, Yaopeng Liu, Xi Wu, Zhenghao Li, Jie Zhang, Xiaosha Wang, Shihan Liu, Guolin Cheng

**Affiliations:** a College of Materials Science and Engineering, Huaqiao University Xiamen 361021 China liushihan@henu.edu.cn; b College of Chemistry and Molecular Sciences, Henan University Kaifeng Henan 475004 China glcheng@hqu.edu.cn

## Abstract

Axially chiral biaryls represent an important class of atropisomers that are prevalent in organic ligands, bioactive molecules, and materials. Despite recent advances in the synthesis of atropisomers *via* the Catellani reaction, the construction of axial chirality at the *meta* position of aryl iodides remains unexplored due to the low reactivity of aryl iodides with bulky *meta* substituents, known as the *meta* constraint. Herein, we report that introducing a directing group at the *meta* position of aryl iodides enables the formation of the aryl-norbornyl-palladacycle (ANP) intermediate, thereby successfully addressing the *meta* constraint. Computational studies show that the designed directing group favors a palladium–potassium heterodimer low barrier transition state, enabling palladium to cleave *ortho*-C–H bonds so as to form the ANP intermediate in an enantioselective manner. A variety of indoloquinolone atropisomers were synthesized with good yields and excellent enantioselectivity using a chiral norbornene (59 examples, up to 80% yield and 99% ee). The practicality of this method is further demonstrated by successful scale-up synthesis and diverse transformations, including the preparation of a chiral[7]helicene and a chiral phosphine ligand. The polycyclic ring systems of the products and their helically chiral derivatives are crucial for potential applications in organic optoelectronic materials.

## Abstract 2

This study addresses the longstanding challenge of *meta* constraint in the Catellani reaction by pioneering a directing group strategy for *meta*-substituted aryl iodides, enabling an atroposelective synthesis of axially chiral biaryls.

## Graphical abstract

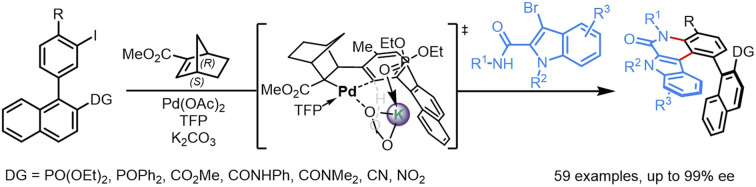

## Introduction

**Scheme 1:**
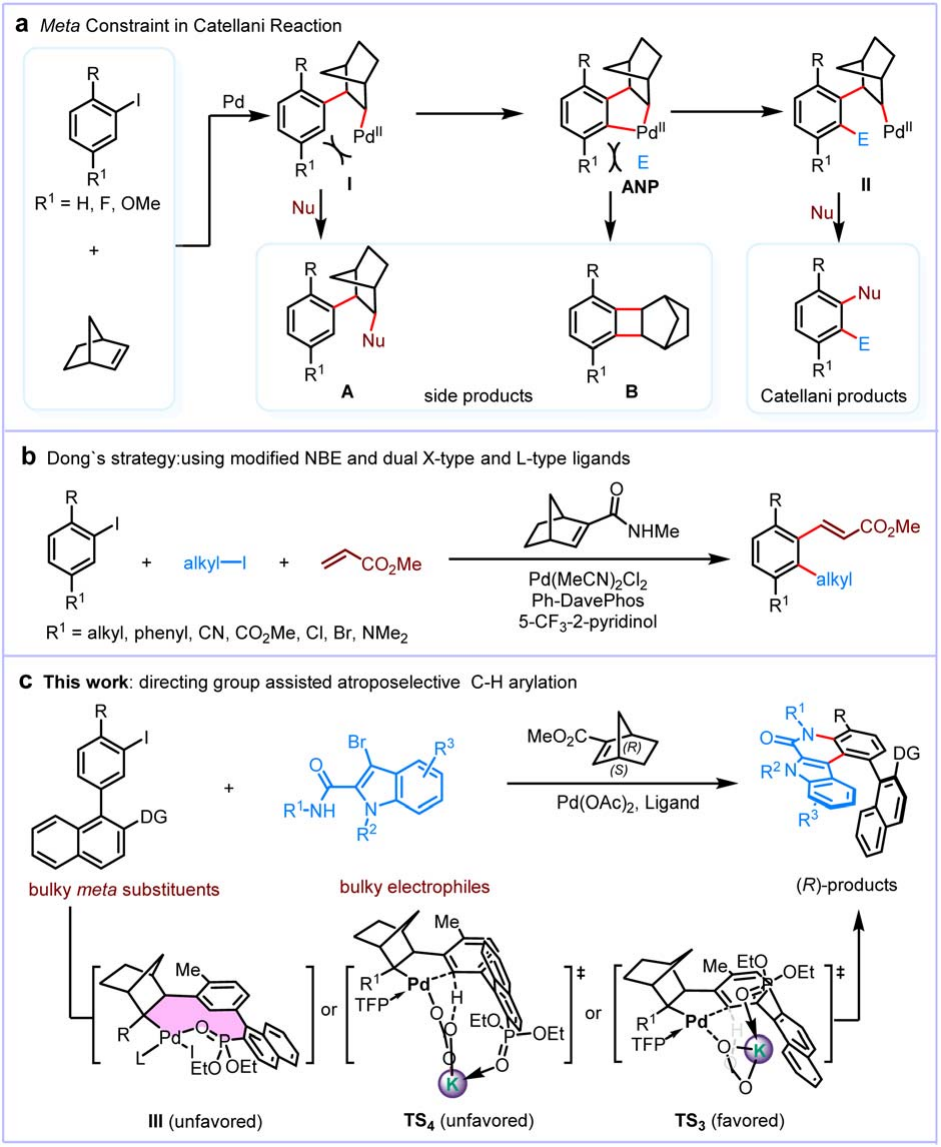
The *meta* constraint in the Catellani reaction.

The breakthroughs in palladium/norbornene (NBE) cooperative catalysis (Catellani reaction^1–7^) have provided a powerful disconnection strategy for target-oriented synthesis, enabling the efficient construction of polysubstituted arenes directly from simple aryl halides.^8–22^ Despite its potential, this strategy has faced significant challenges primarily because of a fundamental limitation in the Catellani reaction known as the *meta* constraint.^23^ Specifically, introducing a sizable substituent at the *meta* position (R^1^) of aryl halides can severely reduce the efficiency of *ortho* functionalization, resulting in NBE-tethered side products A and B (Scheme 1a). First, the *ortho* metalation of intermediate I could not occur efficiently due to the steric hindrance of the *meta* substituents. By contrast, intermediate I would progress to NBE-tethered side products A.^24^ Only aryl halides with small *meta* substituents (*e.g.*, F or OMe) could generate the aryl-norbornyl-palladacycle (ANP) intermediate, thus giving the desired Catellani products.^25–28^ Additionally, even though the ANP intermediate could be formed successfully, the steric hindrance near the ANP could impede its interaction with the electrophiles (*E*). This steric hindrance may instead favor direct reductive elimination, leading to the formation of undesired norbornyl-benzocyclobutene byproduct B by the least sterically demanding pathway possible.^29^ To address this challenge, Lautens realized the intramolecular electrophilic reaction between electrophiles and ANP using substrates tethered with a *meta* electrophile.^30–32^ This “*meta* constraint” was first summarized and overcome by Dong and co-workers.^33^ They developed a strategy using a modified NBE and dual X- and l-type ligands to promote the formation of the ANP intermediate (Scheme 1b). Primary alkyl iodides were proven to be suitable electrophiles to finish the *ortho* alkylation reaction. However, *ortho* amination and *ortho* acylation reactions were achieved less effectively. Thus, to enable the Pd/NBE catalysis to become a more general method for synthesizing polysubstituted arenes, the development of a new strategy to overcome the *meta* constraint, especially when both *meta* substituents and electrophiles are bulky, is essential.

On the other hand, the construction of axial chirality has garnered considerable research interest in recent decades, owing to its versatile applications in organic ligands, pharmaceuticals, agrochemicals, and functional materials.^34–38^ Transition metal-catalyzed enantioselective C–H functionalization enables the introduction of axial chirality in an efficient and atom-economic manner.^39,40^ The synthesis of chiral molecules *via* the Catellani reaction was widely studied by Lautens,^31^ Yu,^41–43^ Gu,^44^ Dong,^45,46^ Zhou,^47–55^ our group,^56–58^ and others.^59–63^ The application of this method to the synthesis of atropisomers is limited, yet highly desirable. In 2018, Gu reported the building of axial chirality at the *ipso* position of aryl iodides *via* the Catellani reaction using a chiral phosphine ligand.^44^ Subsequently, Zhou developed a Pd/chiral NBE-catalyzed construction of axial chirality at the *ortho* position of aryl iodides.^47,49^ However, the construction of axial chirality at the *meta* position of aryl iodides *via* Pd/NBE cooperative catalysis remains a major challenge due to the *meta* constraint.

We speculated that aryl iodides tethered with a directing group at the *meta* position can potentially assist the enantioselective formation of the ANP intermediate, resulting in Catellani products (Scheme 1c). However, the generation of intermediate III is exceedingly challenging because (1) ligand exchange between strongly coordinating phosphine ligands and weakly coordinating oxygen ligands is unlikely and (2) the newly formed 10-membered ring (outlined in color in intermediate III) is highly distorted as a result of the ring strain. Yu and Houk reported that the *meta*-C–H activation could occur through the lowest accessible transition state that contains a heterodimeric Pd–(OAc)–Ag complex, in which the directing group coordinates to Ag, rather than Pd.^64–68^ Inspired by these studies, we propose that the directing group at the *meta* position of aryl iodide substrates may coordinate K, which bridges the Pd by carbonate, placing Pd adjacent to the desired *ortho*-C–H bond (TS_3_). We reported herein an atroposelective synthesis of indoloquinolone atropisomers *via* the Pd/chiral NBE-catalyzed *ortho* C–H arylation/*ipso* amination reaction of *meta* substituted aryl iodides with 3-bromo-indole-2-carboxamides. Our directing group design and experimental efforts were guided by computational studies, and the reaction mechanism involving a heterodimeric Pd–(CO_3_)–K complex for this novel strategy's high yields and enantioselectivities was also evaluated by computational studies.

## Results and discussion

To test our hypothesis, a model reaction using diethyl (1-(3-iodo-4-methylphenyl)naphthalen-2-yl)phosphonate (1p) and 3-bromo-1-methyl-*N*-propyl-1*H*-indole-2-carboxamide (2a) as substrates was conducted. After a comprehensive evaluation of various reaction parameters, it was identified that the anticipated product 3p retains a good reaction efficiency and excellent enantioselectivity (71%, 91% ee) under the following optimal reaction conditions: Pd(OAc)_2_ (10 mol%) as the catalyst, TFP (10 mol%) as the ligand, NBE–CO_2_Me (50 mol%, >99% ee) as the chiral mediator^69^ and 2.5 equivalents of K_2_CO_3_ as the base in DMSO (0.2 M) at 100 °C (Table 1, entry 1). A set of control experiments was subsequently conducted to understand the role of each component. Not surprisingly, in the absence of the Pd catalyst or NBE–CO_2_Me, no desired product 3p was observed (entries 2 and 3). PdCl_2_ was found to give slightly lower yield and enantioselectivity than Pd(OAc)_2_ (entry 4). TFP is a better ligand than PPh_3_ in terms of both reaction efficiency and enantioselectivity (entry 5). When a weaker base Na_2_CO_3_ was used instead of K_2_CO_3_, the yield decreased dramatically (entry 6). Poor yield was obtained when DMF was used as the solvent (entry 7). Changing the NBE–CO_2_Me to other ester NBE N1 (ref. 45) or amide NBE N2 (ref. 70) led to slightly lower yield and ee of 3p (entries 8 and 9). However, only a trace of product was observed using amide NBE N3 (ref. 70) as a mediator (entry 10). The use of achiral NBE as a mediator and (*R*)-BINAP as a ligand only generated 3p in 12% yield with 0% ee (entry 11). Reducing the loading of NBE–CO_2_Me afforded the desired product with a significantly diminished yield (entry 12).

**Table 1:** Optimization of the reaction conditions

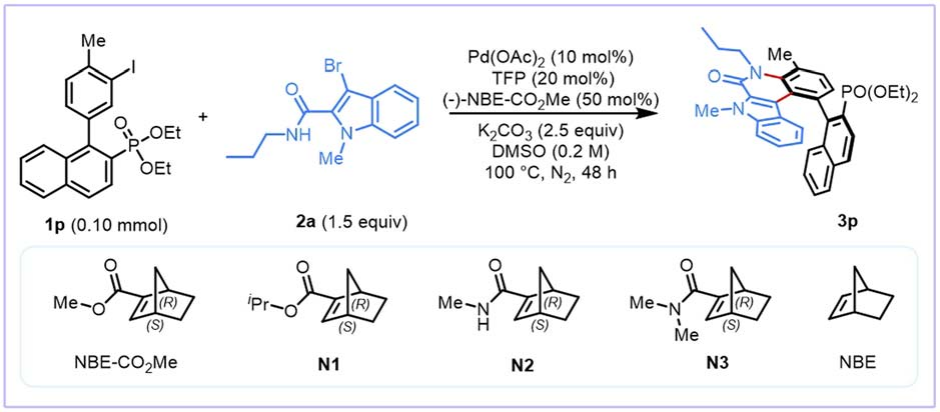			
Entry	Variation of reaction conditions^a^	Yield^b^ (%)	ee^c^ (%)
1	None	71	91
2	No Pd(OAc)_2_	0	—
3	No NBE–CO_2_Me	0	—
4	PdCl_2_ instead of Pd(OAc)_2_	59	89
5	PPh_3_ instead of TFP	51	90
6	Na_2_CO_3_ instead of K_2_CO_3_	26	90
7	DMF instead of DMSO	26	90
8	N1 instead of NBE–CO_2_Me	67	90
9	N2 instead of NBE–CO_2_Me	58	88
10	N3 instead of NBE–CO_2_Me	Trace	—
11^d^	NBE instead of NBE–CO_2_Me	12	0
12	25 mol% NBE–CO_2_Me	49	91

a Reaction conditions unless otherwise noted: 1p (0.10 mmol), 2a (0.15 mmol), Pd(OAc)_2_ (0.01 mmol), TFP (0.02 mmol), NBE–CO_2_Me (0.05 mmol), K_2_CO_3_ (0.25 mmol), DMSO (0.5 mL) under a N_2_ atmosphere at 100 °C for 48 h.

b Yields of isolated products.

c Determined by chiral HPLC.

d (*R*)-2,2′-Bis(diphenylphosphaneyl)-1,1′-binaphthalene (BINAP) instead of TFP. TFP = tri(2-furyl)phosphane. DMF = *N*,*N*-dimethylformamide. DMSO = dimethyl sulfoxide.

**Scheme 2:**
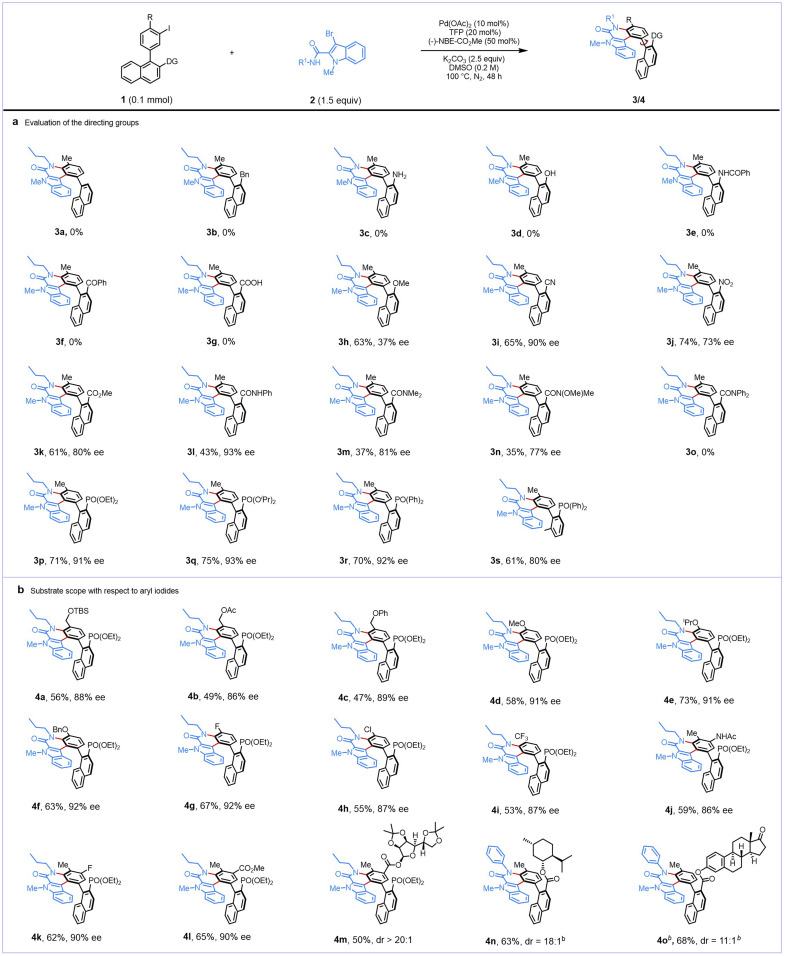
Evaluation of the directing groups and substrate scope of aryl iodides.^*a a*^Reaction conditions unless otherwise noted: 1 (0.10 mmol), 2a (0.15 mmol), Pd(OAc)_2_ (0.01 mmol), TFP (0.02 mmol), NBE–CO_2_Me (0.05 mmol), K_2_CO_3_ (0.25 mmol), DMSO (0.5 mL) under a N_2_ atmosphere at 100 °C for 48 h. ^*b*^Reaction performed with 1 (0.10 mmol), 2h (0.15 mmol), Pd(OAc)_2_ (0.01 mmol), TFP (0.02 mmol), NBE–CO_2_Me (0.05 mmol), KOAc (0.25 mmol), DMSO (0.5 mL) under a N_2_ atmosphere at 100 °C for 48 h.

With the optimized reaction conditions identified, we then attempted to probe the generality of the reaction by testing a representative set of directing groups (Scheme 2a). It was found that the directing groups had a significant influence on the reaction regarding both reactivity and enantioselectivity. When the aryl iodide (3a) lacks a directing group, no target product is formed. Aryl iodides with benzyl (3b), amine (3c), hydroxyl (3d), benzamido (3e), benzoyl (3f), and carboxylic acid (3g) as directing groups all resulted in no desired products. A substrate with a methoxy directing group could provide the desired product (3h) in good yield, albeit in poor enantioselectivity. To our surprise, when using the cyano group as the directing group, the desired product (3i) was obtained in 65% yield and 90% ee, whereas using the nitro group as the directing group gave the target product (3j) in moderate yield and enantioselectivity. We then investigated carboxylic acid derivatives as directing groups. To our delight, the ester directing group enabled the desired reactivity to provide 3k in 61% yield and 80% ee. We are also pleased to find that a range of amides, including *N*-phenyl amide, *N*,*N*-dimethyl amide, and Weinreb amide, were suitable directing groups, affording the desired indoloquinolone atropisomers (3l–3n) in 35–43% yields and 77–93% ee. However, aryl iodide with an *N*,*N*-diphenyl amide group engaged in the reaction ineffectively (3o), likely due to the large steric hindrance of the highly bulky directing group. Next, we examined substrates with phosphonates (1p and 1q) and phosphine oxide (1r) as directing groups, which demonstrated robust reactivity, leading to the desired products (3p–3r) with reasonable yields and excellent enantioselectivities. Finally, *meta* 2-(diphenylphosphoryl)-6-methylphenyl aryl iodide (1s) was successfully engaged in the reaction, providing the formation of the desired product (3s) with 61% yield and 80% ee.

We next examined the scope of *meta* 2-(diethoxyphosphoryl)-1-naphthyl aryl iodides 1. As shown in Scheme 2b, a selection of aryl iodides was well tolerated, providing the desired products (4a–4i) in moderate yields (47–73%) and high enantioselectivities (86–92% ee). A variety of substituents at the *ortho*' position were compatible, such as *tert*-butyldimethylsilyl (TBS)-protected hydroxymethyl (4a), acetoxymethyl (4b), phenoxymethyl (4c), methoxy (4d), isopropoxy (4e), benzyloxy (4f), fluoro (4g), chloro (4h), and trifluoromethyl (4i). Moreover, aryl iodides bearing an electron-donating group (acetamido) at the *meta* position were also examined, affording the corresponding product 4j in moderate yield and 86% ee and those bearing electron-withdrawing groups (fluoro and methoxycarbonyl) gave the corresponding products (4k and 4l) in moderate yields and 90% ee. To probe stereochemical interdependence, we endeavored to extend the reaction to more complex molecules. Chiral aryl iodides 1 bearing natural product moieties, including diacetone-d-glucose, l-menthol, and estrone, were well tolerated in this protocol, producing the corresponding derivatives (4m–4o) with satisfactory dr value.

**Scheme 3:**
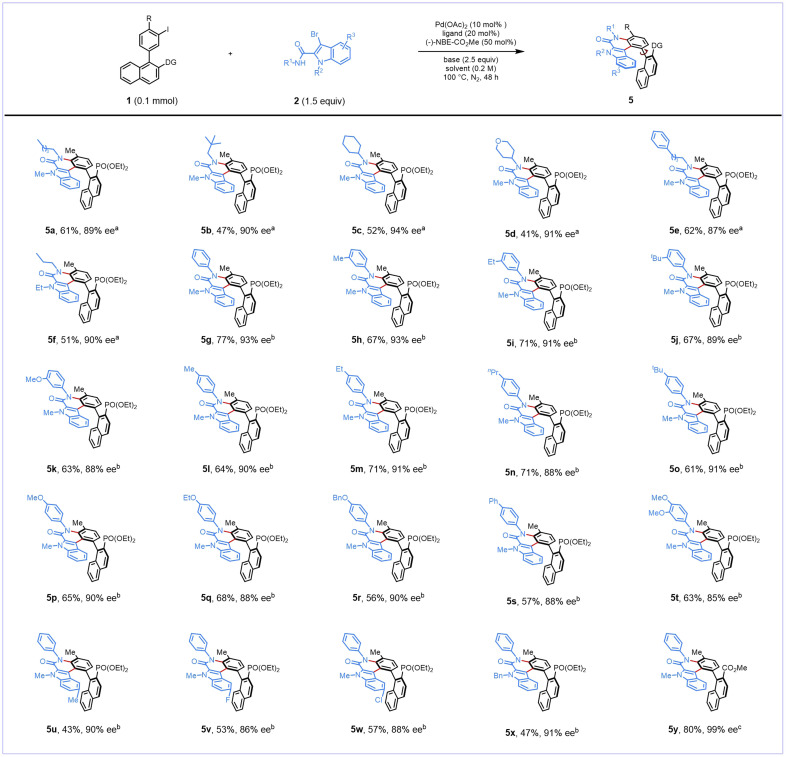
Scope of 3-bromo-indole-2-carboxamides. ^*a*^Reaction conditions: 1p (0.10 mmol), 2 (0.15 mmol), Pd(OAc)_2_ (0.01 mmol), TFP (0.02 mmol), NBE–CO_2_Me (0.05 mmol), K_2_CO_3_ (0.25 mmol), DMSO (0.5 mL) under a N_2_ atmosphere at 100 °C for 48 h. ^*b*^Reaction conditions: 1p (0.10 mmol), 2 (0.15 mmol), Pd(OAc)_2_ (0.01 mmol), DPPP (0.01 mmol), NBE–CO_2_Me (0.05 mmol), K_2_CO_3_ (0.25 mmol), DMA (0.5 mL) under a N_2_ atmosphere at 100 °C for 48 h. ^*c*^Reaction performed with 1k (0.10 mmol), 2h (0.15 mmol), Pd(OAc)_2_ (0.01 mmol), TFP (0.02 mmol), NBE–CO_2_Me (0.05 mmol), KOAc (0.25 mmol), DMSO (0.5 mL) under a N_2_ atmosphere at 100 °C for 48 h.

To further evaluate the generality of this transformation, the optimized reaction conditions were applied to a range of *N*-alkyl 3-bromo-indole-2-carboxamides (Scheme 3). Various alkyl substituents on the amide nitrogen atom (R^1^) were tolerated, and the corresponding products (5a–5e) were obtained generally in moderate yields (41–62%) and good-to-excellent enantioselectivities (87–94% ee). The reaction retained good reactivity when 3-bromo-1-ethyl-indole-2-carboxamide was used as the substrate to deliver 5f in 51% yield and 90% ee. However, unfortunately, when indole substrates bearing Boc, Ts, and Ns protecting groups were employed, none of the desired product was observed.

Subsequently, the reaction scope of *N*-aryl-3-bromo-indole-2-carboxamides was evaluated under slightly modified reaction conditions, including the employment of diphenylphosphopropane (dppp) as the ligand and a solvent change to *N*,*N*-dimethylacetamide (DMA). Specifically, a range of *N*-aryl substrates were subjected to the modified reaction conditions. These reactions resulted in successful generation of the corresponding products (5g–5t), in 63–67% yields and 88–93% ee. Substrates bearing a substituent at the C5 position (R^3^ = methyl, fluoro, and chloro) were also compatible with this atroposelective protocol, affording corresponding products (5u–5w) with good chiral induction. Besides, the 1-benzyl substrate was also suitable, giving the product (5x) in 47% yield and 91% ee. Particularly noteworthy is that the reaction of the *N*-phenyl substrate (2h) with aryl iodide bearing an ester group (1k) showed good reactivity and excellent enantioselectivity (5y). Moreover, the absolute configuration of 5y was unambiguously confirmed by X-ray crystallographic analysis.

**Fig. 1:**
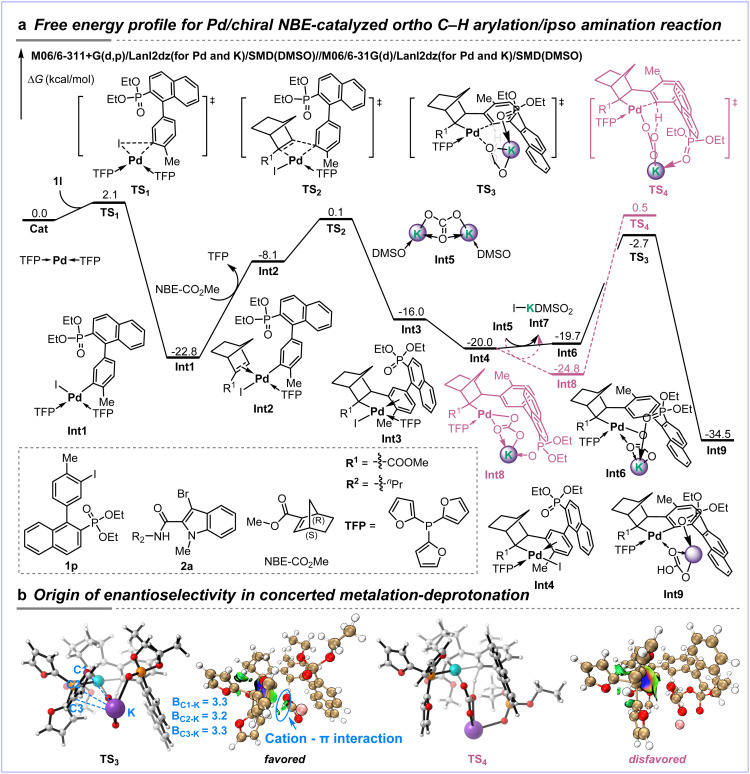
(a) DFT calculations for the Pd/chiral NBE-catalyzed *ortho* C–H arylation/*ipso* amination reaction. All energy values are reported in kcal mol^−1^. (b) Optimized geometries and IGMH analysis of transition states TS_3_ and TS_4_. The bond lengths are given in angstroms.

To elucidate the proposed Pd–K bimetallic catalytic induction model, a systematic mechanistic investigation was conducted using density functional theory (DFT) calculations. The computational results demonstrate that the reaction proceeds through a key C–H activation step involving a heterobimetallic Pd–(CO_3_)–K transition state, which not only significantly reduces the activation barrier but also underpins the observed high enantioselectivity. As depicted in Fig. 1a, Cat is selected as the zero-potential energy reference for the free energy surface. The catalytic cycle commences with the oxidative addition of aryl iodide 1p to Cat, which proceeds *via* transition state TS_1_ with a remarkably low energy barrier of only 2.1 kcal mol^−1^, yielding arylpalladium(ii) intermediate Int1. Subsequent ligand exchange with NBE–CO_2_Me to form the olefin coordinated Pd(ii)–aryl complex Int2 is endergonic by 14.7 kcal mol^−1^, attributable to the weak coordinating ability of the olefin. The coordinated NBE–CO_2_Me then undergoes migratory insertion into the C–Pd bond *via* transition state TS_2_, with an overall activation free energy of 22.7 kcal mol^−1^, affording alkylpalladium(ii) species Int3. Following this, Int3 undergoes an intramolecular isomerization to yield Int4, a process that is exergonic by 4.0 kcal mol^−1^. Subsequently, ligand exchange with Int5 leads to the formation of Int6 and Int7. In Int6, the potassium ion is stabilized in a trigonal coordination environment by a carbonate ligand and a phosphine oxide group. The key concerted metalation–deprotonation (CMD) step then proceeds *via* a six-membered-ring transition state TS_3_ to generate Int9 This step exhibits an overall activation barrier of 17.3 kcal mol^−1^ and is exergonic by 14.5 kcal mol^−1^, indicating its irreversible nature under the reaction conditions. In contrast, the corresponding transition state for the S-configured pathway (TS_4_) was calculated to be higher in energy by 3.2 kcal mol^−1^ (purple dashed lines), consistent with the predominant formation of the *R*-configured product observed experimentally. Following the C–H activation, Int7 undergoes a sequence of transformations including oxidative addition/reductive elimination and β-carbon elimination/N–H bond activation/reductive elimination, ultimately furnishing the final *R*-configured product 3l (see SI Fig. S3 for details).

To elucidate the stereochemical origin in the CMD step, we analyzed the optimized geometries of TS_3_ and TS_4_ (Fig. 1b). In TS_3_, a stabilizing cation–π interaction is clearly observed between the furan ring of the ligand and the potassium ion, which contributes to the stabilization of this transition state. In contrast, such an interaction is absent in TS_4_. This conclusion is further corroborated by an independent gradient model based on Hirshfeld partition (IGMH) analysis.

**Scheme 4:**
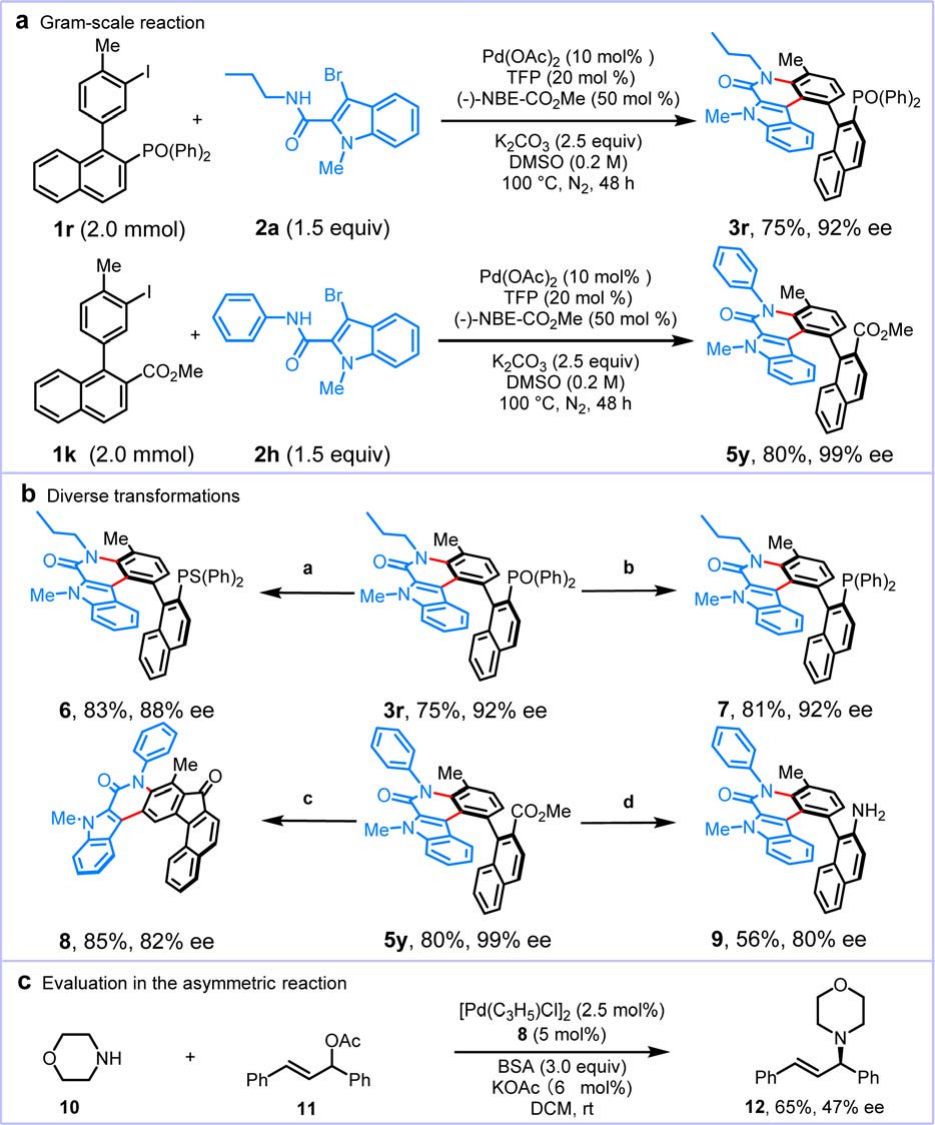
Gram-scale reactions, transformations, and synthetic application. Reaction conditions are as follows: ^*a*^3r (0.10 mmol), Lawesson's reagent (2.0 equiv.), toluene (2.0 mL), N_2_, 105 °C, 12 h. ^*b*^3r (0.10 mmol), Et_3_N (7.0 equiv.), HSiCl_3_ (5.0 equiv.), toluene (2.0 mL), N_2_, 105 °C, 12 h. ^*c*^(1) 5y (0.10 mmol), MeOH/H_2_O (3/1), KOH (2.5 equiv.), 100 °C, 12 h; (2) (COCl)_2_ (4.0 equiv.), DMF (2 drops), DCM (3.0 mL), N_2_, rt, 12 h; (3) AlCl_3_ (4.0 equiv.), N_2_, rt, overnight. ^*d*^(1) 5y (0.10 mmol), MeOH/H_2_O (3/1), KOH (2.5 equiv.), 100 °C, 12 h, (2) TsN_3_ (1.2 equiv.), K_2_CO_3_ (2.0 equiv.), 80 °C, 12 h.

To illustrate the synthetic value of this synthetic strategy, we performed two scale up experiments (2.0 mmol), which afforded the desired products 3r and 5y without any loss of the reaction efficiency and enantioselectivity (Scheme 4a). Given the significance of axially chiral skeletons 3r and 5y in organic synthesis, several transformations were performed (Scheme 4b). The reaction of 3r (92% ee) with Lawesson's reagent could generate the phosphine sulfide 6 in 83% yield with 88% ee. Then, 3r was reduced by HSiCl_3_ to give axially chiral phosphine 7 in 81% yield and 92% ee. In addition, starting from 5y (99% ee), the convenient synthesis of chiral[7]helicene 8 was also readily accomplished *via* sequential hydrolysis, chlorination of acyl groups, and an intramolecular Friedel–Crafts reaction, with efficiency (85% yield and 82% ee). Hydrolysis of 5y and subsequent Curtius rearrangement of the resulting carboxylic acid delivered the corresponding axially chiral amine 9 in 56% yield with 80% ee. To further explore the synthetic utility of this protocol, palladium-catalyzed asymmetric amination using 7 as a ligand was conducted, furnishing the desired product 12 in 65% yield and 47% ee without optimization of the reaction conditions (Scheme 4c).

**Scheme 5:**
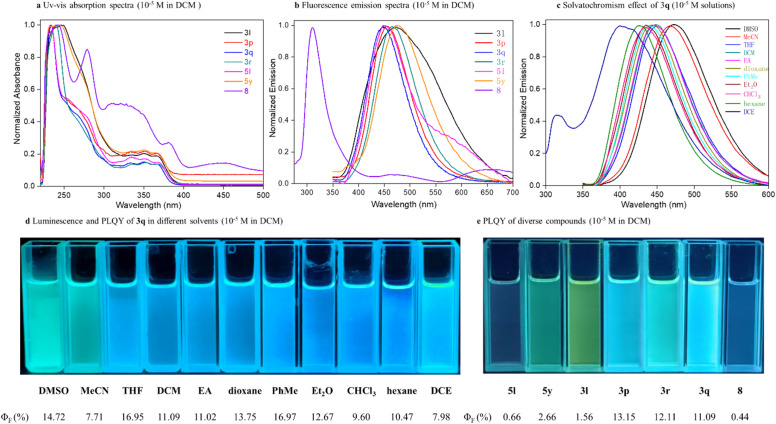
Investigations of photophysical properties.

To further demonstrate the potential applications of the desired chiral indoloquinolone atropisomers in materials science, photophysical and chiroptical characterization of selected synthetic derivatives was conducted in dichloromethane (Scheme 5). First, the UV/Vis absorption and fluorescence spectra of 3l, 3p, 3q, 3r, 5l, 5y, and 8 in dichloromethane with a specific concentration (*c* = 1.00 × 10^−5^ M) were measured. Broadened fluorescence bands were observed across all compounds, while the chiral[7]helicene 8 demonstrated the most significant bathochromic shift, achieving the longest-wavelength emission maxima in both UV-Vis absorption and fluorescence spectra (Scheme 5a and b, and also see Fig. S8–S15 in the SI for details). Next, the solvatochromism effects of 3q in different solvents (Scheme 5c) were obtained. The emission solvatochromism of phosphine oxide 3q showed significant solvent-dependent behavior, with increasing solvent orientation polarizability. Notably, a dual emission at 397 and 334 nm was detected for 3q in 1,2-dichloroethane. Moreover, the fluorescence quantum yields (*Φ*_F_) of 3q were also measured in several solvents, revealing the highest 16.97% in toluene (Scheme 5d). Finally, the fluorescence quantum yields (*Φ*_F_) of selected derivatives were quantified under standardized conditions, ranging from 0.66% to 13.15% (Scheme 5e, see Table S16 in the SI for details).

## Conclusions

In summary, we have realized a palladium/chiral NBE-catalyzed atroposelective *ortho* C–H arylation/*ipso* amination of *meta* substituted aryl iodides with 3-bromo-indole-2-carboxamides. The catalytic system overcomes the low reactivity of aryl iodides with a bulky *meta* substituent by introduction of a directing group to achieve the formation of an aryl-norbornyl-palladacycle intermediate, generating a range of indoloquinolone atropisomers in high reactivity and excellent enantioselectivity. The computational results indicated that potassium ions were involved in the transition state, forming the Pd–(CO_3_)–K bimetallic bridge to provide lower energy barriers. Further derivatizations and photophysical studies highlighted the promising potential for applications in phosphine ligand and organic optoelectronic materials. We anticipate that this method will not only pave the way for discovering other *ortho* C–H functionalizations of *meta* substituted aryl iodides but also inspire the development of new strategies for addressing the *meta* constraint in the Catellani reaction.

## Author contributions

G. C. conceived the work and designed the experiments. J. G. performed the laboratory experiments. Y. L., X. W., Z. L., J. Z., and X. W. explored the substrate scope. S. L. performed the DFT calculations. J. G., S. L., and G. C. analysed the data and co-wrote the manuscript.

## Conflicts of interest

There are no conflicts to declare.

## Supplementary Material

SC-017-D6SC01871H-s001

SC-017-D6SC01871H-s002

## Data Availability

Supplementary information (SI): experimental procedures, mechanistic experiments, characterization data of all the indoloquinolone atropisomers and X-ray data of 5y. See DOI: https://doi.org/10.1039/d6sc01871h.
