# Supplementary material for: Construction of axial chirality through addressing the meta constraint in the Catellani reaction
Source: Chem Sci. 2026 May 19;17(26):13092–100. doi: 10.1039/d6sc01871h (PMC13213624; doi:10.1039/d6sc01871h)
Supplement: SC-017-D6SC01871H-s001 [file SC-017-D6SC01871H-s001.pdf]

Supplementary Information (SI) for Chemical Science.
This journal is © The Royal Society of Chemistry 2026

Supporting Information

Construction of Axial Chirality through Addressing the *Meta* Constraint in Catellani Reaction

Jin Ge,[a] Yaopeng Liu,[a] Xi Wu,[a] Zhenghao Li,[a] Jie Zhang,[a] Xiaosha Wang,[a] Shihan Liu,*[b] and Guolin Cheng*[a]

[a]College of Materials Science and Engineering, Huaqiao University, Xiamen 361021, China

[b]College of Chemistry and Molecular Sciences, Henan University, Kaifeng, Henan 475004, China

*E-mail: liushihan@henu.edu.cn; glcheng@hqu.edu.cn

Contens

1. General Information 3

2. Optimization of reaction conditions 4

3. Preparation of substrates 11

3.1 Substrates involved in this work 11

3.1.1 Aryl Iodobenzenes Used in This Work 11

3.1.2 Amides Used in This Work 12

3.1.3 Preparation of aryl iodides 12

3.1.3 Preparation of Amides 18

4. General procedure for the synthesis of compounds 4 and 5 18

4.1 General procedure for the synthesis of compounds *(R)*-4 18

4.2 General procedure for the synthesis of compounds *(R)*-5 19

5. Kinetic Studies for 2a 19

6. Computational Methods. 21

6.1. M06 calculated thermal corrections, and Gibbs free energies of all structures in dimethylsulfoxide solvent 24

6.2. M06 geometries for all the optimized compounds and transition states in dimethylsulfoxide solvent. 27

6. Synthetic applications 123

7. X-ray crystallographic data 130
8. Optical Properties 132
8.1. UV-vis Absorption and Fluorescence Emission Spectra 132
8.2. Photoluminescence Quantum Yield (PLQY) 136
9. References 137
10. NMR Spectra 138
11. HPLC Charts 260

1. General Information

All the solvents were used without further purification. the other commercial chemicals were used without further purification. All reactions were performed under an inert atmosphere of nitrogen in flame-dried glassware, unless otherwise stated. Analytical thin layer chromatography was performed on 0.25 mm silica gel 60-F254. Visualization was carried out with UV light and Vogel's permanganate.

Preparative TLC was performed on 1.0 mm silica gel. ^{1}H NMR spectra were recorded on a Bruker Avance III instrument (500 MHz). ^{13}C NMR spectra were recorded on a Bruker Avance III instrument (126 MHz) and were fully decoupled by broad band proton decoupling. High-resolution mass spectra (HRMS) were recorded on an Agilent Mass spectrometer using ESI-TOF (electrospray ionization-time of flight). NMR spectra were recorded in $CDCl_3$. ^{1}H NMR spectra were referenced to residual $CHCl_3$ at 7.26 ppm, and ^{13}C NMR spectra were referenced to the central peak of $CDCl_3$ at 77.00 ppm. Chemical shifts (δ) are reported in ppm, and coupling constants (J) are in Hertz (Hz). Multiplicities are reported using the following abbreviations: s = singlet, d = doublet, t = triplet, q = quartet, m = multiplet. High resolution mass spectra (HRMS) were recorded on Thermo Orbitrap Elite (Mode: ESI+FTMS), Bruker Compact TOF (Mode: ESI-TOF or APCI+FTMS) or Bruker micrOTOFII ESI-TOF (Mode: ESI-TOF) mass spectrometer. Enantiomeric ratio (e.e.) values were determined by chiral HPLC (Agilent 1260) with chiral AD-H, IA-3, AS-H, IC-3, OD-H columns with hexane, ^{i}PrOH.

2. Optimization of reaction conditions

Table S1. Screening of the solvents[a]

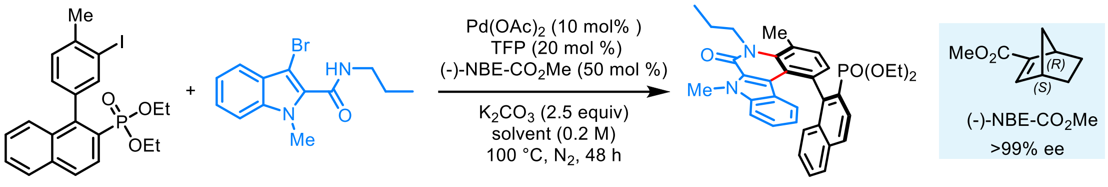

Entry	Solvent	Yield (%)	ee (%)
1	DMF	26	90
2	DMA	trace	--
3	DMSO	71	91
4	NMP	trace	--
5	MeCN	trace	--

[a]All reactions were performed on a 0.1 mmol scale.

Table S2. Screening of the bases[a]

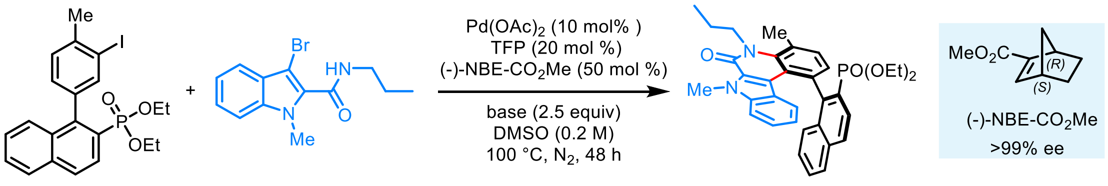

Entry	Base	Yield (%)	ee (%)
1	K_2CO_3	71	91
2	Li_2CO_3	trace	--
3	Na_2CO_3	26	90
4	Cs_2CO_3	37	80
5	KOAc	trace	--
6	CH_3ONa	trace	--
7	K_3PO_4	27	89
8	$KHCO_3$	--	--
9[b]	K_2CO_3	45	91

[a]All reactions were performed on a 0.1 mmol scale. [b]6 equiv 18-Crown-6 were added.

Table S3. Screening of the ligands[a]

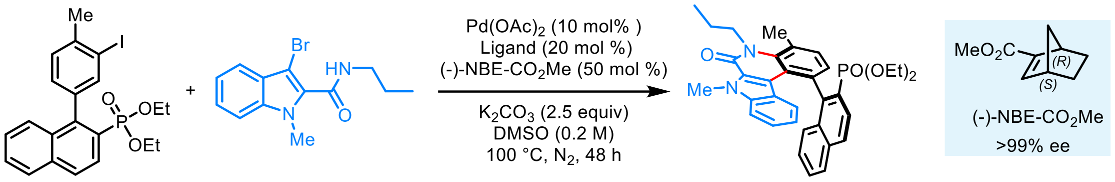

Entry	Ligand	Yield (%)	ee (%)
1	TFP	71	91
2	PCy_3	39	90
3	PPh_3	51	90
4	Ph_2PCy	29	90
5	DPPP	56	91
6	DavePhos	55	90
7	MePhos	46	90
8	DPPB	51	90

[a]All reactions were performed on a 0.1 mmol scale.

Table S4. Screening of the palladium catalysts[a]

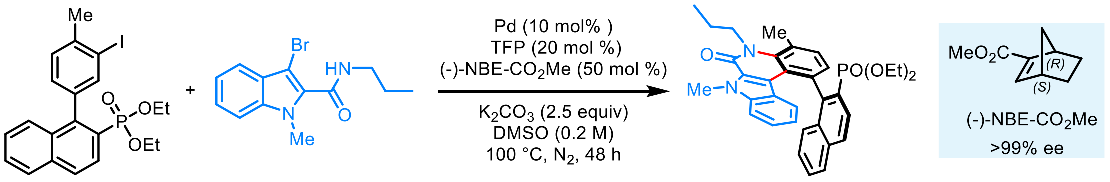

Entry	[Pd]	Yield (%)	ee (%)
1	$Pd(OAc)_2$	71	91
2	$Pd(TFA)_2$	31	90
3	$PdCl_2$	59	89
4	$Pd(dppf)Cl_2$	trace	--
5	$[Pd(C_3H_5)Cl]_2$	trace	--
6	$Pd_2(dba)_3$	43	90

[a]All reactions were performed on a 0.1 mmol scale.

Table S5. Screening of the reaction temperature[a]

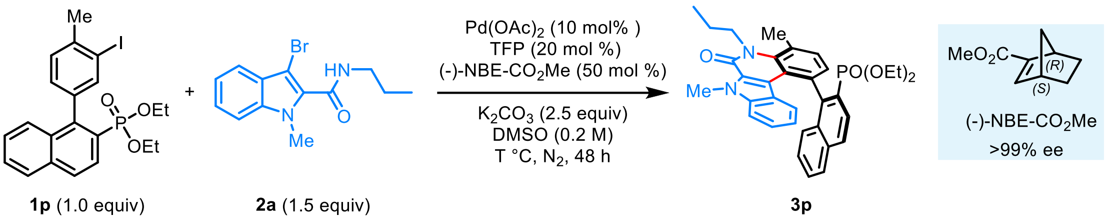

Entry	Temp. (°C)	Yield (%)	ee (%)
1	80	46	91
2	90	56	91
3	100	71	91
4	110	67	88
5	120	66	85

[a]All reactions were performed on a 0.1 mmol scale.

Table S6. Screening of the scope and loading of NBE[a]

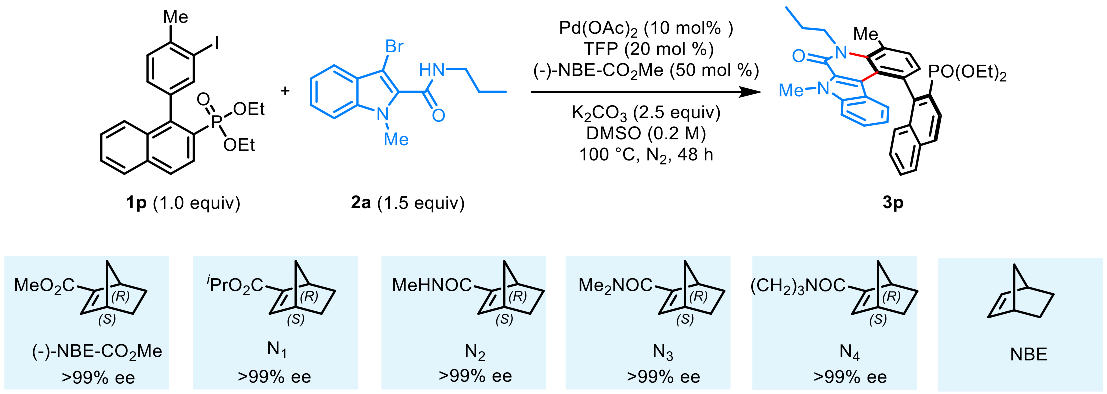

Entry	NBE (equiv)	Yield (%)	ee (%)
1	(-)-NBE-CO_2Me (1.0)	73	91
2	(-)-NBE-CO_2Me (0.5)	71	91
3	(-)-NBE-CO_2Me (0.25)	49	91
4	N_1 (0.5)	67	91
5	N_2 (0.5)	58	88
6	N_3 (0.5)	trace	--
7	N_4 (0.5)	trace	--
8[b]	NBE (0.5)	12	--

[a]All reactions were performed on a 0.1 mmol scale. [b](R)-BINAP instead of TFP as ligand

Table S7. Screening of the loading of **2a** and DMSO[a]

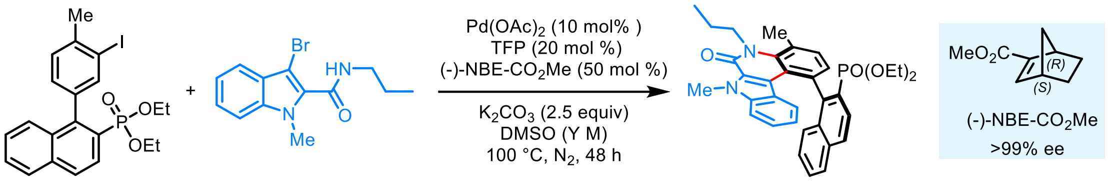

1p (1.0 equiv) **2a** (X equiv) **3p**

Entry	**2a** (equiv)	DMSO (M)	Yield (%)	ee (%)
1	1.0	0.2	47	88
2	1.5	0.2	71	91
3	2.0	0.2	73	90
4	1.5	0.1	63	91
5	1.5	0.05	58	90

[a]All reactions were performed on a 0.1 mmol scale.

Table S8. Screening of the solvents[a]

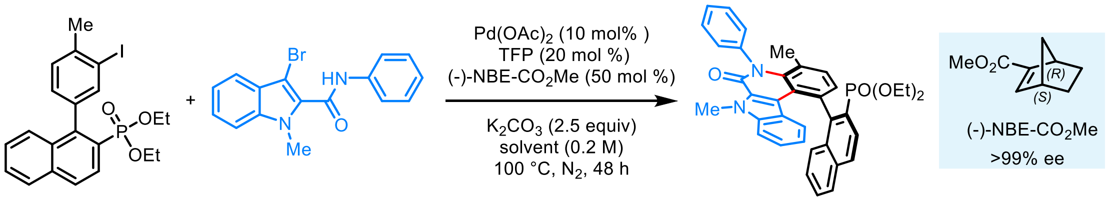

1p (1.0 equiv) **2h** (1.5 equiv) **5g**

Entry	Solvent	Yield (%)	ee (%)
1	DMF	44	64
2	DMA	58	93
3	DMSO	23	66
4	NMP	33	83
5	MeCN	39	60
6	DME	trace	--
7	THF	46	33
8	Acetone	52	56

[a]All reactions were performed on a 0.1 mmol scale.

Table S9. Screening of the bases[a]

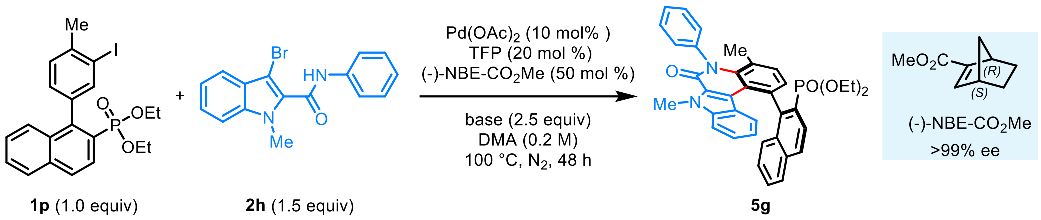

Entry	Base	Yield (%)	ee (%)
1	K_2CO_3	58	93
2	Na_2CO_3	26	92
3	Cs_2CO_3	30	88
4	KOAc	18	90
5	CH_3ONa	trace	--
6	K_3PO_4	24	89
7	$KHCO_3$	trace	--

[a]All reactions were performed on a 0.1 mmol scale.

Table S10. Screening of the ligands[a]

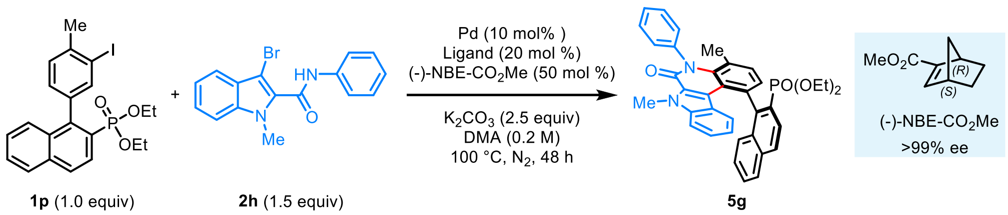

Entry	Ligand	Yield (%)	ee (%)
1	TFP	58	92
2	XPhos	39	87
3	PPh_3	47	92
4	Ph_2PCy	41	93
5	BIANP	46	91
6	RuPhos	45	88
7	DavePhos	37	90
8	MePhos	26	91
9[b]	DPPP	65	93

[a]All reactions were performed on a 0.1 mmol scale. [b]10 mol% ligand was used.

Table S11. Screening of the palladium catalysts[a]

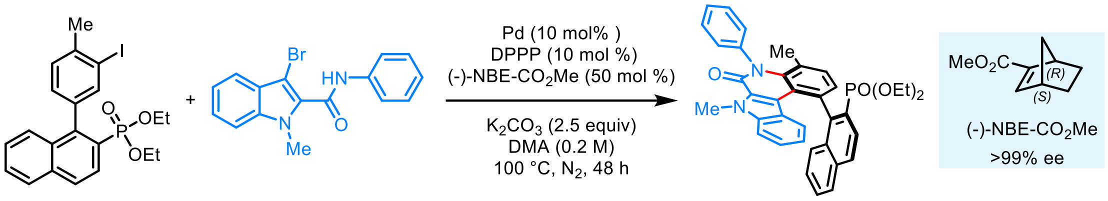

1p (1.0 equiv) **2h** (1.5 equiv) **5g**

Entry	[Pd]	Yield (%)	ee (%)
1	$Pd(OAc)_2$	65	93
2	$Pd(TFA)_2$	33	92
3	$PdCl_2$	54	93
4	$Pd(dppf)\ Cl_2$	27	92
5	$[Pd(C_3H_5)Cl]_2$	35	92
6	$Pd_2(dba)_3$	48	93

[a]All reactions were performed on a 0.1 mmol scale.

Table S12. Screening of the reaction temperature[a]

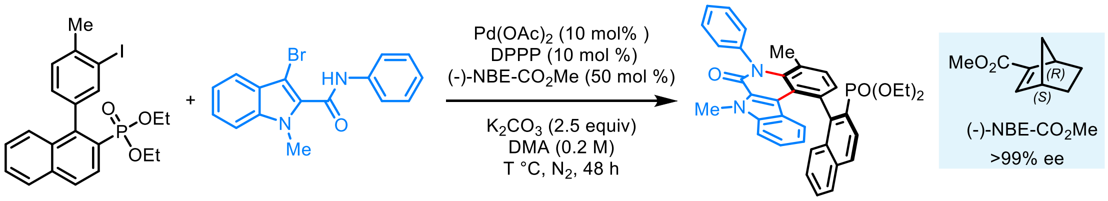

1p (1.0 equiv) **2h** (1.5 equiv) **5g**

Entry	Temp.	Yield (%)	ee (%)
1	80	77	93
2	90	70	93
3	100	65	93
4	110	53	92
5	120	52	89

[a]All reactions were performed on a 0.1 mmol scale.

Table S13. Screening of the scope and loading of NBE[a]

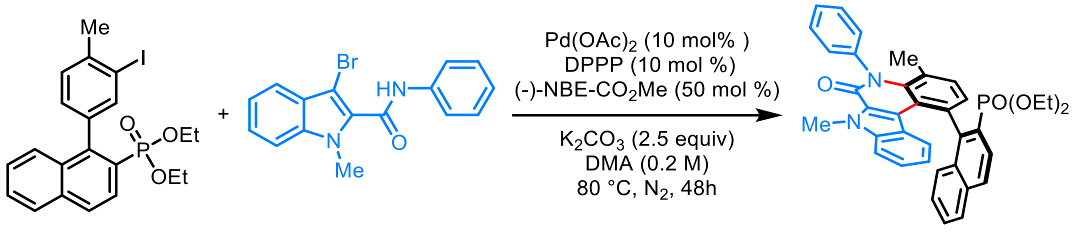

1p (1.0 equiv) **2h** (1.5 equiv) **5g**

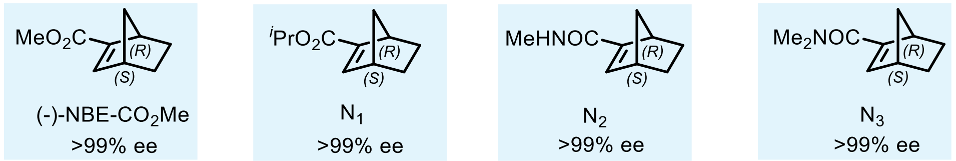

Entry	NBE (equiv)	Yield (%)	ee (%)
1	(-)-NBE-CO$_2$Me (1.0)	79	93
2	(-)-NBE-CO$_2$Me (0.5)	77	93
3	(-)-NBE-CO$_2$Me (0.25)	63	93
4	N_1 (0.5)	66	92
5	N_2 (0.5)	57	88
6	N_3 (0.5)	trace	--

[a]All reactions were performed on a 0.1 mmol scale.

Table S14. Screening of the loading of **2a** and DMA[a]

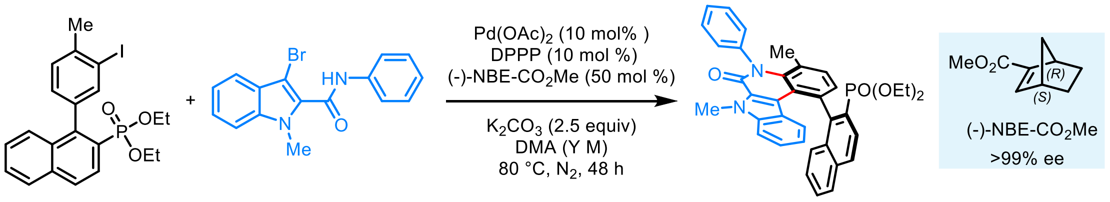

1p (1.0 equiv) **2h** (X equiv) **5g**

Entry	**2a** (equiv)	DMA (M)	Yield (%)	ee (%)
1	1.0	0.2	58	93
2	1.5	0.2	77	93
3	2.0	0.2	75	93
4	1.5	0.1	67	93
5	1.5	0.05	58	92

[a]All reactions were performed on a 0.1 mmol scale.

3. Preparation of substrates

3.1 Substrates involved in this work

3.1.1 Aryl Iodobenzenes Used in This Work

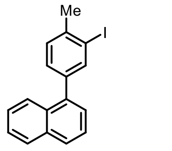

1a

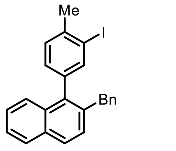

1b

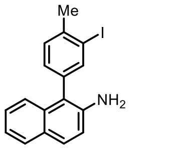

1c

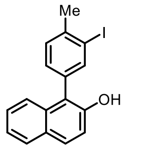

1d

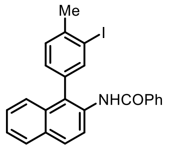

1e

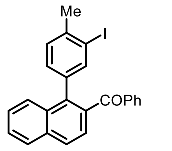

1f

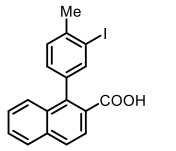

1g

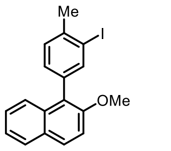

1h

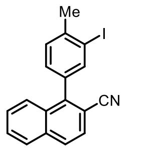

1i

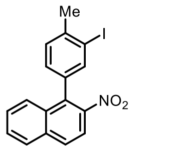

1j

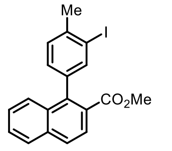

1k

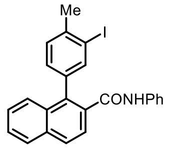

1l

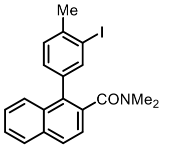

1m

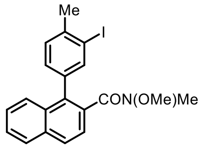

1n

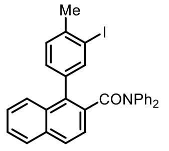

1o

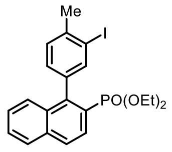

1p

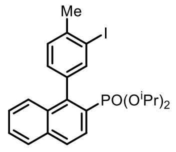

1q

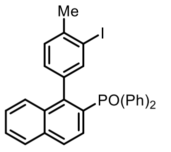

1r

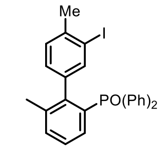

1s

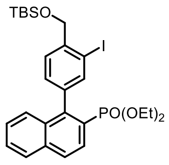

1t

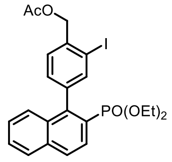

1u

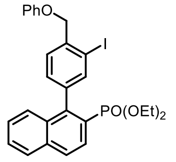

1v

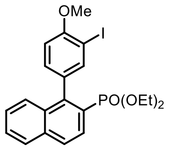

1w

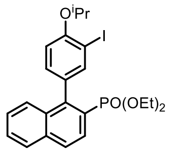

1x

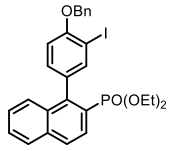

1y

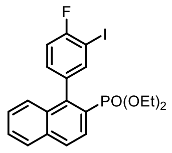

1z

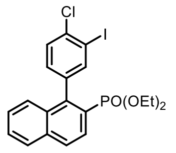

1aa

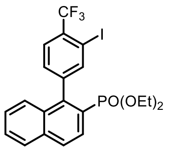

1ab

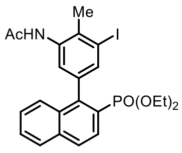

1ac

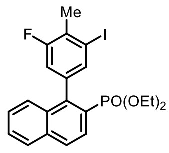

1ad

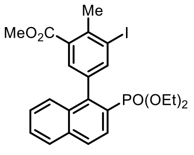

1ae

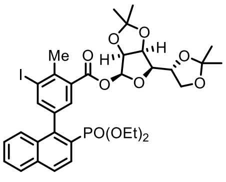

1af

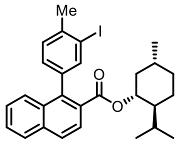

1ag

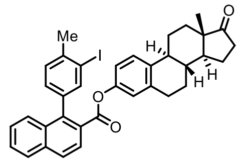

1ah

3.1.2 Amides Used in This Work

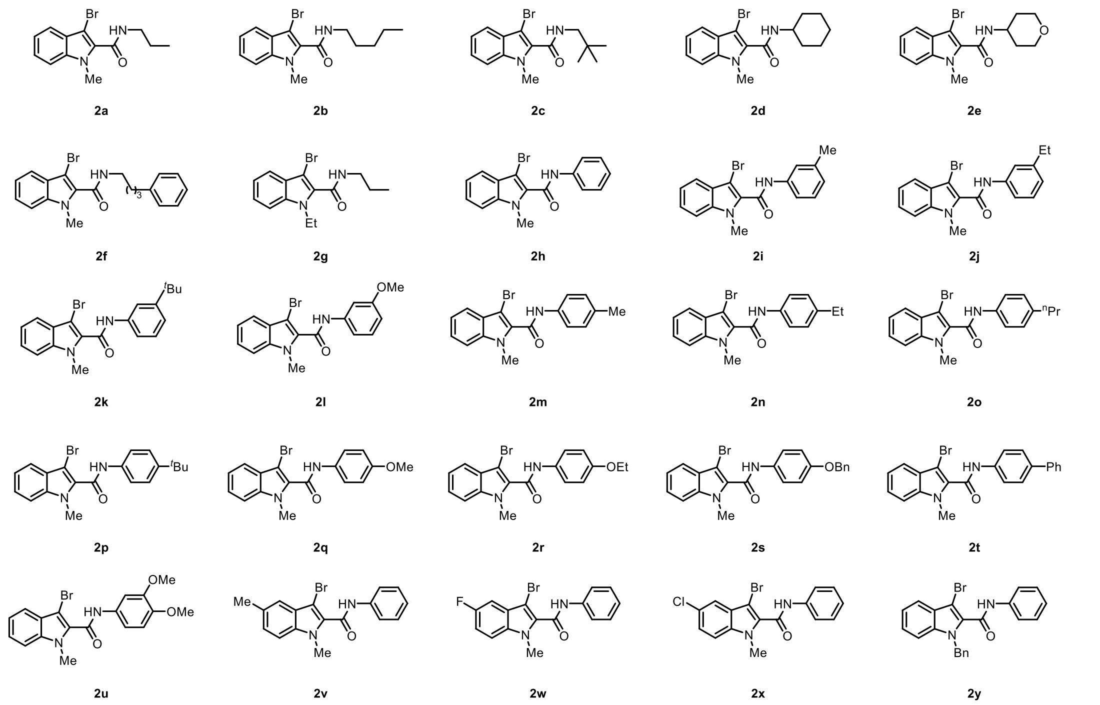

All amides were prepared according to the methodology described by the literature procedures.[1]

3.1.3 Preparation of aryl iodides

1-Bromonaphthalen-2-amine (**S1**), 1-bromo-2-naphthol (**S2**), 1-bromo-2-methoxynaphthalene (**S3**), 1-bromo-2-naphthoic acid (**S4**) were commercially available and were used without further purification. Other aryl bromides were synthesized as follows.

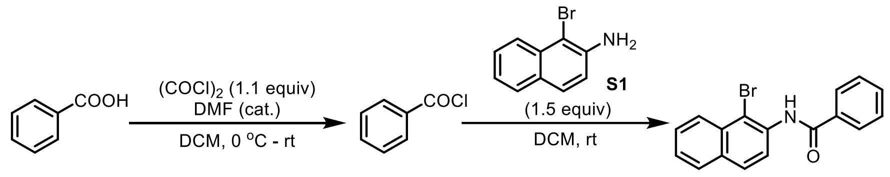

S5

To a solution of benzoic acid (0.48 g, 4.0 mmol, 1.0 equiv) and DMF (one drop) in DCM (5 mL) was added oxalyl chloride (0.56 g, 4.4 mmol, 1.1 equiv) dropwise at 0 °C. The reaction mixture was then stirred at room temperature for 1 h until gas evolution ceased, and concentrated *in vacuo* to afford benzoyl chloride as a yellow solid. This intermediate was used in the next step without further purification. The crude benzoyl chloride was dissolved in DCM (5 mL). To this solution, 1-bromonaphthalen-2-amine (1.32 g, 6.0 mmol, 1.5 equiv) was added dropwise at 0 °C. The mixture was then warmed to room temperature and stirred for 12 h. After completion, the reaction mixture was concentrated *in vacuo*, and

the residue was directly purified by column chromatography on silica gel to yield the desired product **S5**.

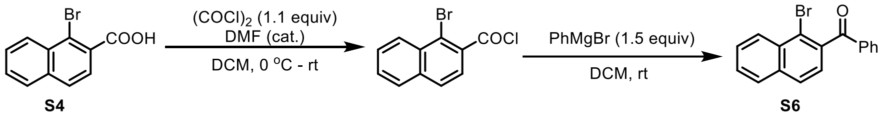

To a solution of 1-bromo-2-naphthoic acid **S4** (1.0 g, 4.0 mmol, 1.0 equiv) and DMF (one drop) in DCM (5 mL) was added oxalyl chloride (0.56 g, 4.4 mmol, 1.1 equiv) dropwise at 0 °C. The reaction mixture was then stirred at room temperature for 1 h until gas evolution ceased, and concentrated *in vacuo* to afford 1-bromo-2-naphthoyl chloride as a yellow solid. This intermediate was used in the next step without further purification. The crude acyl chloride was dissolved in dry THF (5 mL). To this solution, the Grignard reagent (0.87 g, 4.8 mmol, 1.2 equiv) was added dropwise at 0 °C. The mixture was then warmed to room temperature and stirred for 2 h. The reaction was quenched by the addition of a saturated aqueous NH_4Cl solution. The mixture was transferred to a separatory funnel, and the organic layer was separated. The aqueous layer was further extracted with EtOAc (2 × 10 mL). The combined organic layers were washed with brine, dried over anhydrous Na_2SO_4, filtered, and concentrated *in vacuo*. The residue was purified by column chromatography on silica gel to afford the desired product **S6.**[1]

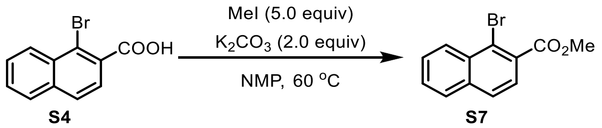

A mixture of 1-bromo-2-naphthoic acid **S4** (1.0 g, 4.0 mmol, 1.0 equiv) and iodomethane (2.8 g, 20.0 mmol, 5.0 equiv) in NMP (10 mL) was treated with K_2CO_3 (1.1 g, 8.0 mmol, 2.0 equiv). The resulting mixture was stirred at 60 °C for 4 h. After cooling, water (10 mL) was added, and the mixture was extracted with ethyl acetate (3 × 10 mL). The combined organic layers were washed with water to remove residual NMP, dried over anhydrous $MgSO_4$, and concentrated under reduced pressure. The residue was purified by flash column chromatography on silica gel to afford the desired product **S7**.

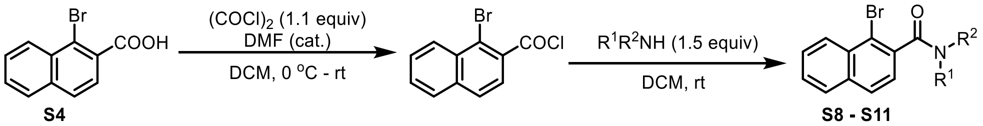

To a solution of 1-bromo-2-naphthoic acid **S4** (1.0 g, 4.0 mmol, 1.0 equiv) and DMF (one drop) in DCM (5 mL) was added oxalyl chloride (0.56 g, 4.4 mmol, 1.1 equiv) dropwise at 0 °C. The reaction mixture was then stirred at room temperature for 1 h until gas evolution ceased, and concentrated *in vacuo* to afford 1-bromo-2-naphthoyl chloride as a yellow solid. This crude intermediate was used directly in the next step without further purification. The crude acyl chloride from the previous step was dissolved in dry DCM (5 mL). To this solution at 0 °C was added dropwise one of the following amine components (1.5 equiv): Aniline (0.56 g, 6.0 mmol), or $NHMe_2$ (2 M in THF, 3.0 mL, 6.0 mmol), or Diphenylamine (1.0 g, 6.0 mmol), or *N*,*O*-dimethylhydroxylamine hydrochloride (0.58 g, 6.0 mmol), After the addition, the reaction mixture was warmed to room temperature and stirred for 12 h. The mixture was then concentrated *in vacuo*, and the residue was purified directly by column chromatography on silica gel to yield the corresponding desired products (**S8–S11**).

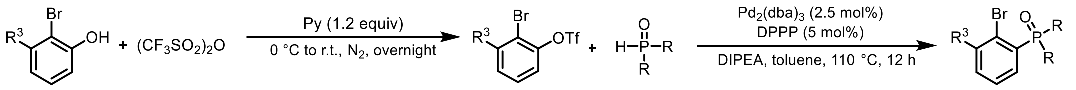

S12 - S15

R = EtO, *i*PrO, Ph

To a solution of the phenol derivative (20.0 mmol, 1.0 equiv) in pyridine (20 mL) under N_2 at 0 °C was added dropwise triflic anhydride (6.21 g, 22 mmol, 1.1 equiv) over 10 minutes. After stirring at 0 °C for an additional 30 minutes, the cooling bath was removed, and the mixture was stirred at room temperature overnight. The reaction mixture was then diluted with EtOAc (50 mL) and 3 M HCl (60 mL). The aqueous layer was extracted with EtOAc (100 mL). The combined organic layers were washed sequentially with 3 M HCl (100 mL), saturated aqueous $NaHCO_3$ (100 mL), and brine (100 mL), dried over anhydrous Na_2SO_4, and concentrated under reduced pressure. The residue was purified by flash chromatography on silica gel to afford the corresponding 1-bromo-2-naphthyl triflate, which was used without further purification.

A mixture of the 1-bromo-2-naphthyl triflate (19.2 mmol, 1.0 equiv), diethyl phosphite (3.18 g, 23.04 mmol, 1.2 equiv), *N*,*N*-diisopropylethylamine (3.72 g, 28.8 mmol, 1.5 equiv), $Pd_2(dba)_3$ (439.5 mg, 0.48 mmol, 2.5 mol%), and 1,3-bis(diphenylphosphino)propane

(dppp, 395.9 mg, 0.96 mmol, 5 mol%) in anhydrous toluene (30 mL) was degassed and placed under a nitrogen atmosphere. The reaction mixture was stirred at 110 °C for 12 hours. After cooling to room temperature, it was diluted with EtOAc (40 mL). The aqueous layer was extracted with EtOAc (40 mL). The combined organic layers were dried over anhydrous Na_2SO_4 and concentrated under reduced pressure. The residue was purified by column chromatography on silica gel to yield the desired products **S12–S15**.[2]

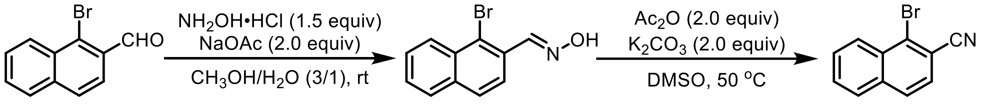

a solution of 1-bromo-2- naphthaldehyde (1.0 g, 4.3 mmol), hydroxylamine hydrochloride (420 mg, 6.0 mmol), sodium acetate (1.2 g, 8.5 mmol), water (5 ml) and methanol (15 ml) was stirred for 4 h at rt. Upon completion, the mixture was extracted with H_2O (10 mL) and DCM (10 mL×3). The organic phases were combined, dried over Na_2SO_4, filtered and concentrated under reduced pressure. The crude product was used directly in the next step. To a solution of the crude product in DMSO (15 mL), K_2CO_3 (1.2 g, 8.5 mmol) was added, then Ac_2O (0.80 mL, 8.5 mmol). The resulting mixture was stirred at 50 ℃ overnight. Upon completion, the mixture was extracted with H_2O (10 mL) and DCM (10 mL×3). The organic phases were combined, dried over Na_2SO_4, and concentrated under reduced pressure. The residue was purified by column chromatography on silica gel to yield the desired products **S16**.[3]

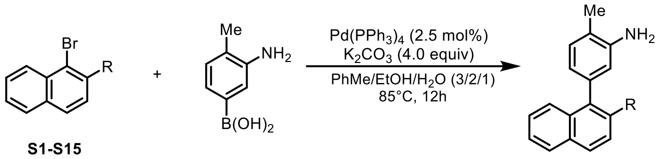

A mixture of the starting material (**S1–S16**, 3.0 mmol, 1.0 equiv), aryl boronic acid (452.9 mg, 3.6 mmol, 1.2 equiv), $Pd(PPh_3)_4$ (86.7 mg, 2.5 mol%), and K_2CO_3 (1.66 g, 12.0 mmol, 4.0 equiv) in a solvent mixture of toluene (15 mL), ethanol (10 mL), and water (5 mL) was stirred at 85 °C under a nitrogen atmosphere overnight. After the reaction was complete (as monitored by TLC), the mixture was cooled to room temperature, diluted with water (5 mL), and extracted with diethyl ether (3 × 20 mL). The combined organic extracts were dried over anhydrous Na_2SO_4, filtered, and concentrated under reduced pressure.

The crude residue was purified by column chromatography on silica gel to afford the desired arylamine products.

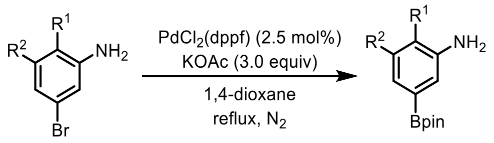

5-Bromo-2-methylaniline (6.49 g, 34.9 mmol, 1.0 equiv), bis(pinacolato)diboron (B_2pin_2, 9.74 g, 38.4 mmol, 1.1 equiv), [$PdCl_2$(dppf)] (684 mg, 0.94 mmol, 3 mol%), and potassium acetate (KOAc, 14.8 g, 105 mmol, 3.0 equiv) were placed in a side-armed round-bottom flask under a nitrogen atmosphere. Anhydrous 1,4-dioxane (90 mL) was added via syringe. The reaction mixture was heated to reflux for 24 hours. After cooling to room temperature, the solvent was removed *in vacuo*. The resulting black residue was dissolved in a 1:9 (v/v) mixture of ethyl acetate and dichloromethane, filtered through a pad of Celite, and concentrated. The crude material was purified by column chromatography on silica gel to afford the corresponding aryl pinacol boronate ester.

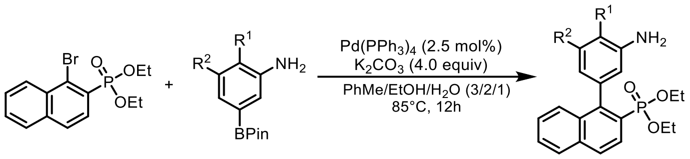

A mixture of the 1-bromo-2-naphthyl phosphonate ester (3.0 mmol, 1.0 equiv), arylboronic acid (3.6 mmol, 1.2 equiv), $Pd(PPh_3)_4$ (86.7 mg, 2.5 mol%), and K_2CO_3 (1.66 g, 12.0 mmol, 4.0 equiv) in a solvent mixture of toluene (15 mL), ethanol (10 mL), and water (5 mL) was stirred at 85 °C under a nitrogen atmosphere overnight. After the reaction was complete (as monitored by TLC), the mixture was cooled to room temperature, diluted with water (10 mL), and extracted with diethyl ether (3 × 20 mL). The combined organic extracts were dried over anhydrous Na_2SO_4, filtered, and concentrated under reduced pressure. The crude residue was purified by column chromatography on silica gel to afford the corresponding aryl-substituted naphthyl phosphonate derivatives.

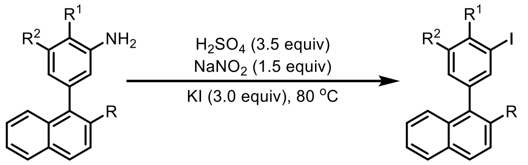

1b - 1aa, 1ae-1ag

A 25 mL round-bottom flask was charged with the arylamine (2 mmol) and sulfuric acid

(10 mL, 6.9 M). The mixture was stirred at room temperature until the formation of the ammonium sulfate salt was complete. The resulting mixture was then cooled to 0 °C, and a solution of sodium nitrite ($NaNO_2$, 0.20 g, 3.0 mmol, 1.5 equiv) in water (1 mL) was added dropwise. During this addition, the color of the mixture gradually changed from pink to dark brown. After stirring at 0 °C for 20 minutes, an aqueous solution of potassium iodide (KI, 1.00 g, 6.0 mmol, 3.0 equiv) in water (1 mL) was added dropwise. Subsequently, the reaction mixture was warmed to 80 °C and heated until gas evolution ceased. The mixture was then cooled and carefully neutralized by the dropwise addition of a sodium hydroxide solution. The neutralized mixture was extracted with ethyl acetate. The combined organic extracts were dried over anhydrous Na_2SO_4, filtered, and concentrated under reduced pressure. The crude residue was purified by column chromatography on silica gel to afford the desired aryl iodides **1a–1aa** and **1ae–1ag**.

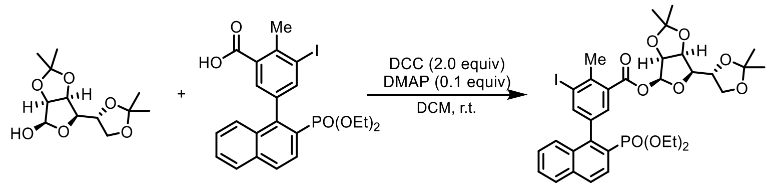

1ab

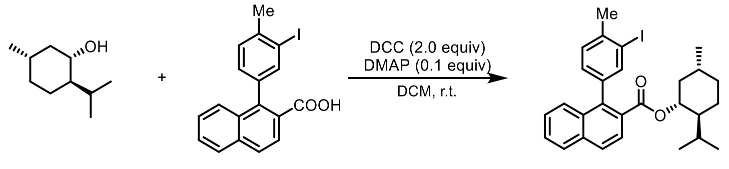

1ac

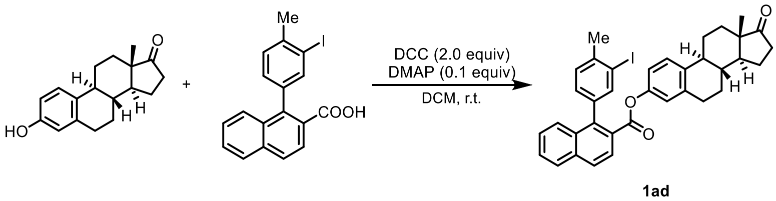

1ad

To a stirred solution of the appropriate alcohol substrate diacetone-D-glucose (0.52 g, 2.0 mmol, 1.0 equiv), L(-)-menthol (0.33 g, 2.0 mmol, 1.0 equiv), or estrone (0.54 g, 2.0 mmol, 1.0 equiv) in dry dichloromethane (DCM, 15 mL) were added N,N'-dicyclohexylcarbodiimide (DCC, 0.83 g, 4.0 mmol, 2.0 equiv) and 4-dimethylaminopyridine (DMAP, 24.4 mg, 0.2 mmol, 0.1 equiv). After stirring for 5 minutes, iodobenzoic acid (1.05 g, 2.0 mmol, 1.0 equiv) was added. The reaction mixture was stirred at room temperature overnight. The resulting mixture was filtered. The filtrate was washed sequentially with

water (15 mL) and brine (15 mL). The organic layer was then dried over anhydrous Na_2SO_4, filtered, and concentrated *in vacuo*. The crude residue was purified by column chromatography on silica gel to afford the desired ester products (**1ab–1ad**).

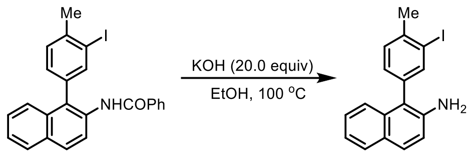

The benzamide substrate **1d** (352.2 mg, 1.0 mmol, 1.0 equiv) was dissolved in anhydrous ethanol (5 mL). Potassium hydroxide (KOH, 1.10 g, 20 mmol, 20 equiv) was added, and the reaction mixture was heated at reflux for 48 hours. After cooling to room temperature, the mixture was transferred to a separatory funnel. The reaction vessel was rinsed with ethyl acetate (10 mL) and water (5 mL), and the rinses were combined with the main mixture. The resulting mixture was diluted with additional water (15 mL) and extracted with ethyl acetate (3 × 15 mL). The combined organic extracts were washed with brine (20 mL), dried over anhydrous Na_2SO_4, filtered, and concentrated *in vacuo*. The residue was purified by column chromatography on silica gel to afford the deprotected amine product (**1a**, 55% yield).

3.1.3 Preparation of Amides

Amides were prepared according to the reported procedures.[4]

4. General procedure for the synthesis of compounds 4 and 5

4.1 General procedure for the synthesis of compounds *(R)*-4

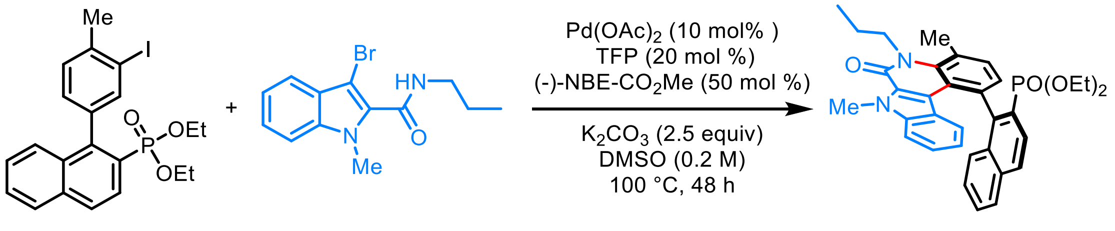

In an argon-filled glove box, a 10 mL oven-dried vial equipped with a magnetic stir bar was charged with $Pd(OAc)_2$ (2.3 mg, 0.01 mmol, 10 mol%), tri(2-furyl)phosphine (4.5 mg, 0.02 mmol, 20 mol%), K_2CO_3 (35.6 mg, 0.25 mmol, 2.5 equiv), (-)-NBE-CO_2Me (8.3 mg, 0.05 mmol, 50 mol%), aryl iodobenzene **1** (0.10 mmol, 1.0 equiv), amide **2** (0.15 mmol, 1.5

equiv) and dry DMSO (0.5 mL). The vial was sealed with a cap and then transferred out of the glove box and stirred at 100 °C for 48 h. After the mixture was cooled to room temperature, it was filtered through a thin pad of celite (eluting with ethyl acetate, 10 mL) and the combined filtrate was concentrated in vacuo. The residue was directly purified by column chromatography on silica gel to give the desired products.

4.2 General procedure for the synthesis of compounds *(R)*-5

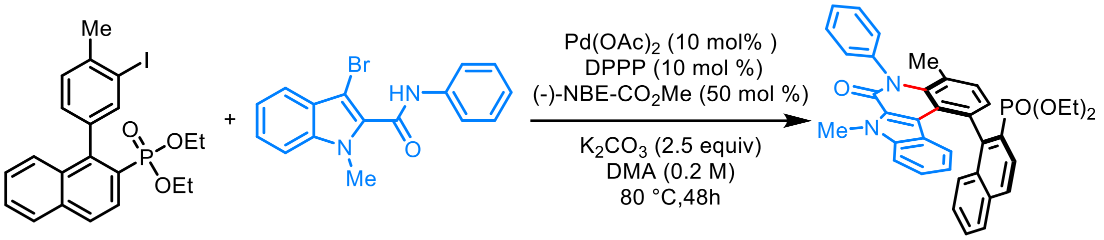

1 (1.0 equiv) **2** (1.5 equiv) (*R*)-**5**

In an argon-filled glove box, a 10 mL oven-dried vial equipped with a magnetic stir bar was charged with $Pd(OAc)_2$ (2.3 mg, 0.01 mmol, 10 mol%), 1,3-Bis(diphenylphosphino)propane (4.1 mg, 0.01 mmol, 10 mol%), K_2CO_3 (35.6 mg, 0.25 mmol, 2.5 equiv), (-)-NBE-CO_2Me (8.3 mg, 0.05 mmol, 50 mol%), aryl iodobenzene **1** (0.10 mmol, 1.0 equiv), amide **2** (0.15 mmol, 1.5 equiv) and dry DMA (0.5 mL). The vial was sealed with a cap and then transferred out of the glove box and stirred at 80 °C for 48 h. After the mixture was cooled to room temperature, it was filtered through a thin pad of celite (eluting with ethyl acetate, 10 mL) and the combined filtrate was concentrated in vacuo. The residue was directly purified by column chromatography on silica gel to give the desired products.

5. Kinetic Studies for 2a

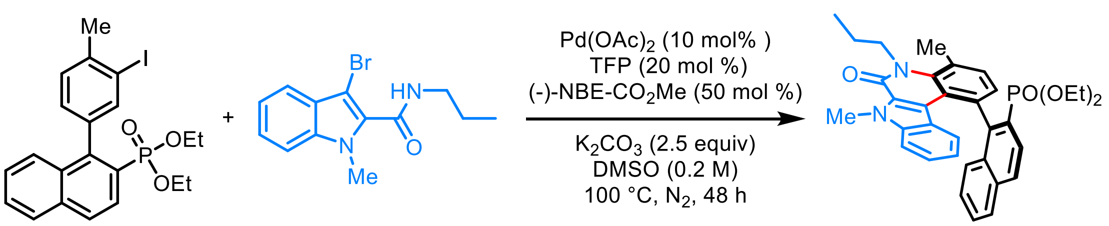

1p **2a** **3p**

Under nitrogen atmosphere, a Schlenk flask (10 mL) was sequentially charged with **1p** (240.1 mg, 0.5 mmol), **2a** (n mmol), NBE-CO_2Me (38.0 mg, 0.25 mmol), K_2CO_3 (172.8 mg, 1.25 mmol), $Pd(OAc)_2$ (11.2 mg, 10 mol%), DMSO (0.2 M). Then the mixture was

stirred at 100 ºC.

Samples (0.25 mL of reaction mixture) were taken from the flask and quickly filtered through a short pad of silica gel. The conversions of **2a** were measured by crude ^{1}H NMR. The slope of the concentration of **3p** vs time line was presented in Figure S1. After calculation of all the reaction rates, the reaction rate vs the amount of **2a** (Figure S2) function was simulated. The result suggests that the reaction rate was the first-order dependence on the concentration of **2a**.

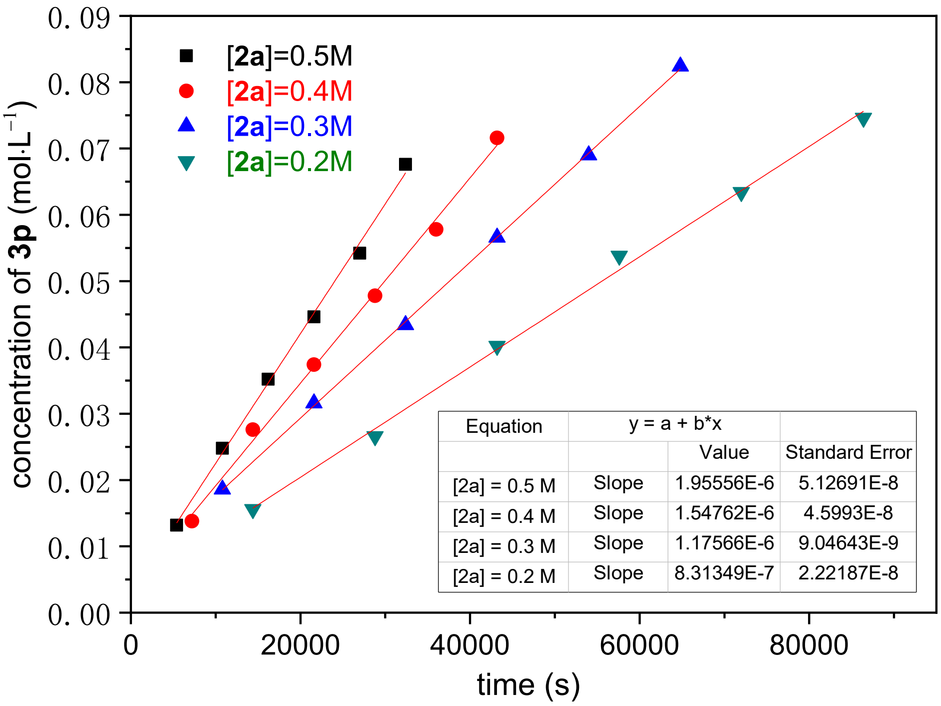

Figure S1

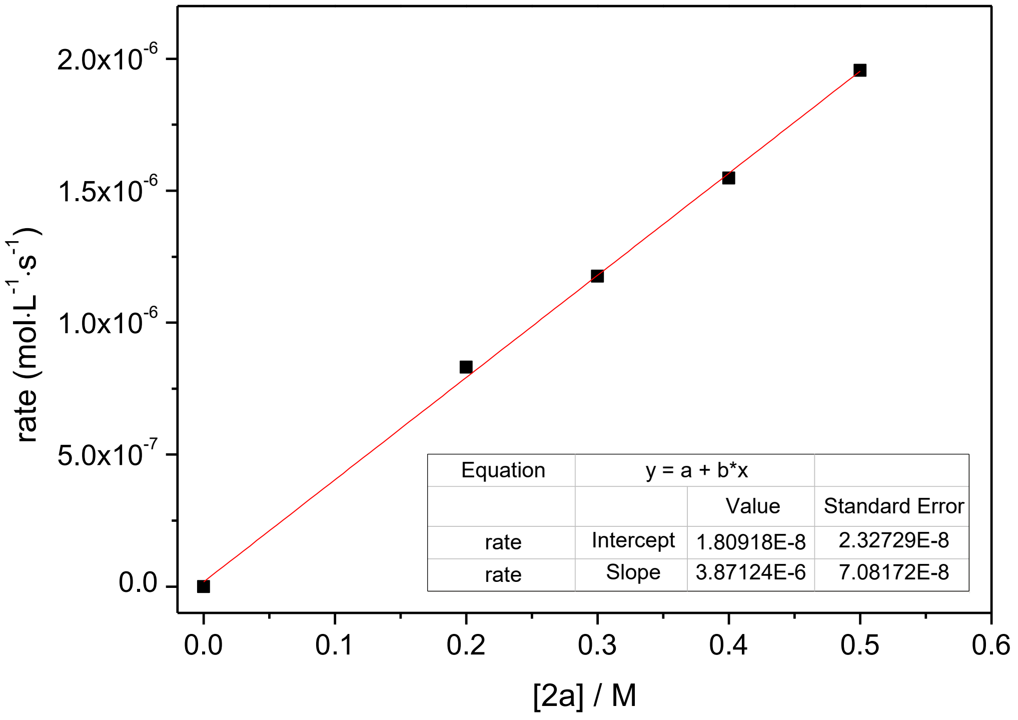

Figure S2

6. Computational Methods.

All DFT calculations were carried out using Gaussian 16 program. All the geometry optimizations and frequency calculations in this paper were performed with M06 functional[5] in implicit dimethylsulfoxide by using the Solvation Model based on Density[6] (SMD) with keyword in the Gaussian code route section "SCRF = (SMD, Solvent = dimethylsulfoxide)". The 6-31G(d) basis set was used for C, H, O, N, P, Br, S atoms, while the LANL2DZ[7] basis set was used for Pd and K atoms. The vibrational frequencies were computed at the same level of theory as for the geometry optimizations to confirm whether each optimized structure is an energy minimum or a transition state, and to evaluate the zero-point vibrational energy (ZPVE) and thermal corrections. The thermal corrections Gibbs free energies were calculated at 298.15 K (default) under standard state in solution conditions of 1 M[8-9] (ideal solvent) by adding 0.00301 Hartree to the free energies. Single-point energy calculations were also performed on an optimized geometry using a higher level basis set with the 6-311+G(d,p) for C, H, O, N, P, Br, S atoms and LANL2DZ basis set for Pd and K

atoms. the IGMH analysis have been plotted with the Multiwfn[10] and VMD[11] program package. The three-dimensional molecular diagrams were generated using CYLView[12].

Calculated potential energy surface for **1p**:

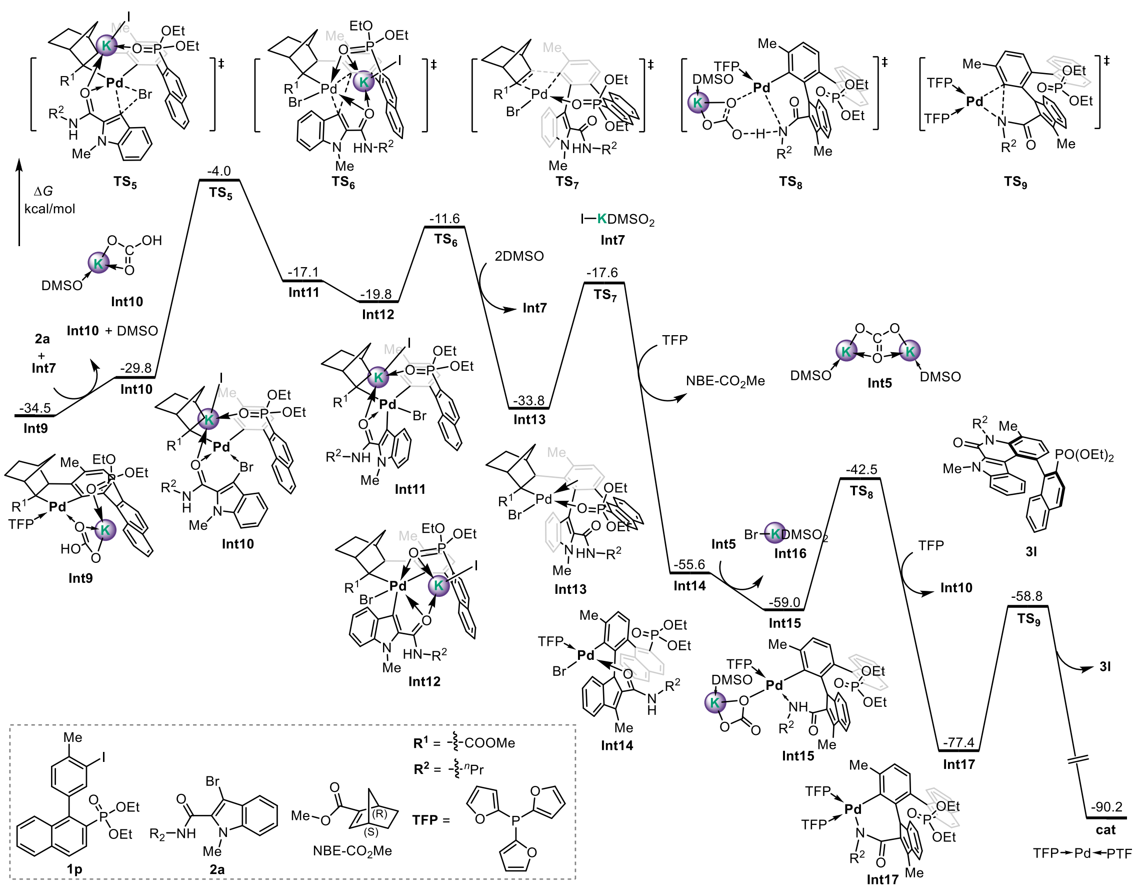

Figure S3. DFT calculations for Pd/chiral NBE-catalyzed *ortho* C-H arylation/*ipso* amination reaction. All energy values are reported in kcal/mol.

Following the formation of **Int9**, ligand exchange with **Int7** generates **Int10** with concomitant release of one DMSO molecule. Subsequently, C−Br bond oxidative addition proceeds through a three-membered-ring transition state (**TS$_6$**) to afford the Pd(IV) species **Int11**. This step is calculated to have an overall activation free energy of 30.5 kcal/mol relative to **Int9**, thereby establishing it as the rate-determining step (RDS) of the catalytic cycle. Thereafter, a cascade of reductive elimination and *β*-C elimination ensues. From **Int12**, reductive elimination via **TS$_6$** (ΔG‡ = 8.2kcal/mol) yields **Int13**, which undergoes migratory insertion through **TS$_7$** to give the aryl palladium(II) intermediate **Int14**. Subsequent N–H bond activation then occurs through the six-membered transition state

TS$_8$ (ΔG‡ = 16.5 kcal/mol), delivering **Int17**. Finally, C–N reductive elimination via **TS**$_9$ (ΔG‡ = 18.6 kcal/mol) affords the target product **3p**.

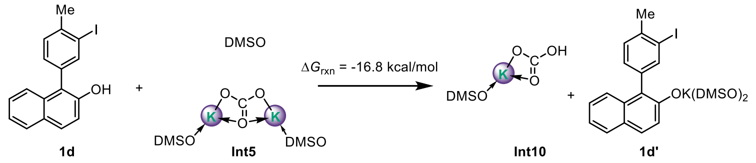

Figure S4. Calculated Gibbs free energy for deprotonation process of **1d** by **Int5**.

Calculated potential energy surface for **1d**:

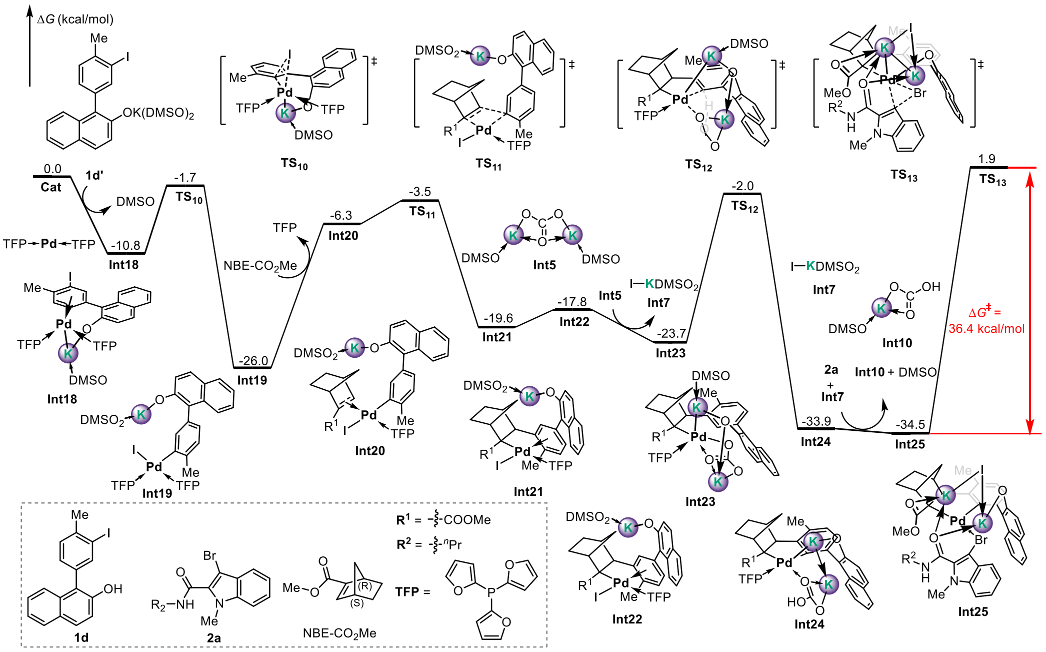

Figure S5. DFT calculations for Pd/chiral NBE-catalyzed *ortho* C-H arylation/*ipso* amination reaction.

We have performed additional DFT calculations on the hydroxyl-directed substrate **1d** based on our existing work and compared its potential energy surface with that of the phosphonate substrate **1p**. As shown in Figure S4, the computational results show that under basic conditions, the hydroxyl group is readily deprotonated. After which the coordination mode with palladium changes significantly. This leads to an increased barrier for the key C–Br bond oxidative addition step (the oxidative addition barrier for **1p** is 30.5 kcal/mol, compared to 36.4 kcal/mol for **1d**) (See Figure S3 and Figure S5), rendering the reaction infeasible due to the prohibitive activation energy.

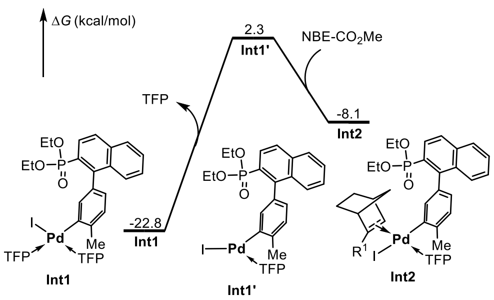

Figure S6. Free energy profiles of ligand exchange process between **Int1** and NBE-CO_2Me.

6.1. M06 calculated thermal corrections, and Gibbs free energies of all structures in dimethylsulfoxide solvent

Geometry	Thermal Correction to Free Energy	Thermal Correction to Enthalpy	Electronic Energy	Gibbs Free Energy$_{(1M)}$	IF
**Cat**	0.299158	0.398563	-2185.531308	-2185.22914	
**1p**	0.324852	0.409780	-1391.493118	-1391.165256	
**TS_1**	0.651664	0.809237	-3577.045718	-3576.391044	126.72*i*
**Int1**	0.654248	0.811211	-3577.088019	-3576.430761	
**NBE-CO_2Me**	0.160562	0.206620	-500.415327	-500.251755	
**TFP**	0.139694	0.197195	-1029.351458	-1029.208754	
**Int2**	0.673291	0.820718	-3048.126622	-3047.450321	
**TS_2**	0.673982	0.819419	-3048.114287	-3047.437295	261.48*i*
**Int3**	0.676815	0.821760	-3048.142843	-3047.463018	
**Int4**	0.675750	0.822205	-3048.148117	-3047.469357	
**Int5**	0.113070	0.200727	-1426.446651	-1426.330571	

**Int6**	0.698274	0.843613	-3328.718299	-3328.01702	
**Int7**	0.105463	0.179941	-1145.890933	-1145.78246	
**Int8**	0.693199	0.843658	-3328.720073	-3328.023864	
**TS_3**	0.689275	0.837054	-3328.682172	-3327.989887	1808.34*i*
**TS_4**	0.692671	0.837761	-3328.680233	-3327.984552	1786.40*i*
**Int9**	0.693296	0.843050	-3328.73688	-3328.040574	
**Int10**	0.737026	0.891404	-5309.266341	-5308.526305	
**DMSO**	0.051213	0.085906	-553.135261	-553.081038	
**2a**	0.212344	0.277351	-3262.883904	-3262.66855	
**TS_5**	0.737742	0.890834	-5309.225894	-5308.485142	214.14*i*
**Int11**	0.741404	0.891678	-5309.250589	-5308.506175	
**Int12**	0.743529	0.893694	-5309.25702	-5308.510481	
**TS_6**	0.740592	0.891951	-5309.240943	-5308.497341	193.98*i*
**Int13**	0.752505	0.889618	-5269.667932	-5268.912417	
**TS_7**	0.744435	0.885915	-5269.633942	-5268.886497	162.64*i*
**Int14**	0.726392	0.877983	-5798.633595	-5797.904193	
**Int15**	0.810875	0.987633	-4069.703468	-4068.889583	
**Int16**	0.107733	0.178870	-3708.54229	-3708.431547	
**TS_8**	0.811560	0.981872	-4069.677916	-4068.863346	1253.44*i*
**Int17**	0.884640	1.061357	-4253.34747	-4252.45982	
**TS_9**	0.884809	1.059936	-4253.317892	-4252.430073	387.27*i*
**3p**	0.554303	0.662384	-2067.808291	-2067.250978	

**1d**	0.203733	0.266187	-741.879359	-741.672616	
**1d'**	0.331234	0.431854	-1875.777145	-1875.442901	
**Int18**	0.590912	0.744949	-3508.202248	-3507.608326	
**TS_{10}**	0.590772	0.743727	-3508.187542	-3507.59376	149.57*i*
**Int19**	0.589568	0.744768	-3508.225057	-3507.632479	
**Int20**	0.682363	0.836944	-3532.410466	-3531.725093	
**TS_{11}**	0.683579	0.840533	-3532.407353	-3531.720764	245.21*i*
**Int21**	0.682246	0.844851	-3532.431661	-3531.746405	
**Int22**	0.688364	0.844144	-3532.43494	-3531.743566	
**Int23**	0.630261	0.778044	-3259.853159	-3259.219888	
**TS_{12}**	0.622865	0.771179	-3259.81118	-3259.185305	1851.11*i*
**In24**	0.626853	0.777002	-3259.865997	-3259.236134	
**Int25**	0.600443	0.738785	-4687.252966	-4686.649513	
**TS_{13}**	0.602965	0.736595	-4687.197297	-4686.591322	118.36*i*

6.2. M06 geometries for all the optimized compounds and transition states in dimethylsulfoxide solvent.

Cat

Pd	0.00063900	-0.00234300	-0.00185300
P	-2.31521200	-0.00013600	-0.00132900
P	2.31718700	-0.00161500	-0.00173900
C	-3.06697200	1.45074400	-0.76802700
C	-2.46346400	2.53789600	-1.33527800
O	-4.42428200	1.58395600	-0.84866000
C	-3.50884700	3.39188000	-1.79067200
H	-1.39306300	2.69821800	-1.41274100
C	-4.66920600	2.76096300	-1.46667200
H	-3.41166100	4.34716800	-2.29221100
H	-5.71351200	3.01293500	-1.60632900
C	-3.06354800	-0.05994900	1.64050200
C	-2.45788700	-0.10882000	2.86454900
O	-4.42062000	-0.05796300	1.79790700
C	-3.50181600	-0.13757800	3.83352500
H	-1.38727500	-0.12311200	3.04025700
C	-4.66331200	-0.10469200	3.12713400
H	-3.40301900	-0.17616900	4.91157900
H	-5.70714900	-0.10794600	3.41678200
C	-3.06648900	-1.39155100	-0.87167500
C	-2.46479800	-2.42589900	-1.53199000
O	-4.42378200	-1.52736200	-0.94325300
C	-3.51178600	-3.24800300	-2.03940000
H	-1.39477000	-2.57319600	-1.63581200
C	-4.67102400	-2.65262200	-1.65022100
H	-3.41627700	-4.16035900	-2.61554300
H	-5.71602400	-2.89980000	-1.79392800
C	3.06669100	0.65931500	1.50132100
C	2.46159300	1.16576100	2.61725700
O	4.42369000	0.70797700	1.65108000
C	3.50589200	1.54844600	3.50733500
H	1.39110600	1.25078100	2.77294100
C	4.66699400	1.24685800	2.86665500
H	3.40771100	1.98973700	4.49176700
H	5.71102600	1.35309000	3.13559600
C	3.06628800	-1.63372700	-0.18101500
C	2.46119400	-2.85346300	-0.29925400
O	4.42318500	-1.78757900	-0.21466700
C	3.50565500	-3.81570700	-0.41230500
H	1.39058400	-3.03047200	-0.30389100
C	4.66670000	-3.10992100	-0.35528800
H	3.40763800	-4.88885400	-0.52268600
H	5.71064200	-3.39587200	-0.39994100
C	3.06626800	0.97198200	-1.32327300
C	2.46117300	1.68848400	-2.31737200
O	4.42311100	1.07968000	-1.43877100
C	3.50538400	2.27132500	-3.09155400
H	1.39066900	1.78008100	-2.46906700
C	4.66642400	1.86708600	-2.51018900
H	3.40700300	2.90584800	-3.96401100
H	5.71029400	2.04946800	-2.73513800

1l

C	2.30512500	0.17687500	0.40934400
C	1.05080300	0.52739500	-0.07542300
C	-0.01802800	0.69795500	0.80837400
C	0.20496500	0.51907300	2.17301900
C	1.46889500	0.17760800	2.63881900
C	2.55506300	-0.00223200	1.77555200
H	-0.61879600	0.64849300	2.87361300
H	1.63008000	0.04597900	3.70895600
H	0.88849900	0.66387500	-1.14460300
C	-1.35461200	1.11566700	0.30259900
C	-1.57949300	2.49872400	0.02353000
C	-2.38125000	0.20176400	0.10809700
C	-0.57333600	3.48305000	0.20799800
C	-2.85987300	2.91782600	-0.44679900
C	-3.65253700	0.63499600	-0.34895700
C	-0.82136000	4.80468500	-0.06944100
H	0.40543100	3.18249200	0.57780100
C	-3.08406600	4.28830200	-0.72532700
C	-3.88258000	1.95664600	-0.62055800
H	-4.44480900	-0.09542800	-0.49222600
C	-2.08730800	5.21350200	-0.54322600
H	-0.03702000	5.54510500	0.07863900
H	-4.06763900	4.58991200	-1.08594900
H	-4.85841200	2.28674800	-0.97665900
H	-2.26921000	6.26494300	-0.75946900
P	-2.11997400	-1.54389100	0.47638000
I	3.87266500	-0.09201800	-1.01897300
C	3.89640700	-0.37079500	2.32403300

H	4.24253800	-1.33538700	1.92672300
H	4.65945500	0.37406300	2.05783600
H	3.86213800	-0.44669000	3.41693500
O	-3.38406300	-2.20661100	-0.26441200
O	-0.88677600	-1.97086900	-0.46293000
O	-1.96136500	-1.92275200	1.90346500
C	-3.41071000	-3.64394500	-0.35312700
C	-4.74228300	-4.05460800	-0.91710700
H	-3.25167100	-4.07009500	0.64816500
H	-2.58342700	-3.97059600	-1.00118700
H	-4.78818900	-5.14636000	-1.01023300
H	-5.56079200	-3.72926500	-0.26317700
H	-4.89601900	-3.61734700	-1.91151700
C	0.19263800	-2.76932000	0.05338600
C	1.05788200	-3.16225800	-1.11184600
H	-0.21349200	-3.65261000	0.56646700
H	0.75445300	-2.17719700	0.79047400
H	1.92890400	-3.72971100	-0.76092400
H	0.49931300	-3.78692500	-1.82118100
H	1.41863700	-2.27231300	-1.64524800

TS_1

Pd	-0.97118300	0.68213800	-0.66818800
P	-2.32805900	1.56824100	1.10803700
P	-2.01998200	-1.50801600	-0.99645000
C	-2.48342200	3.35833300	1.35223300
C	-2.44986200	4.17980600	2.44152500
O	-2.72015100	4.09387400	0.22525800
C	-2.68348600	5.50099500	1.95670100
H	-2.28498000	3.87333400	3.46840800
C	-2.84560400	5.38550200	0.61240200
H	-2.72421400	6.41639300	2.53485900
H	-3.03951500	6.09926500	-0.17844900
C	-1.80132900	1.02041300	2.75044600
C	-2.45308800	0.80797700	3.93118800
O	-0.51100100	0.58643900	2.85121100
C	-1.50672000	0.20657000	4.81152300
H	-3.49173000	1.04763000	4.13541500
C	-0.35282100	0.09141600	4.09734800
H	-1.66361000	-0.10269700	5.83826100
H	0.63458900	-0.28688300	4.33698800
C	-4.09926600	1.15660900	1.13623300
C	-5.23167500	1.91981800	1.08181200
O	-4.42977200	-0.16196100	1.03248300
C	-6.32337700	1.01091200	0.93667100
H	-5.28255200	3.00215300	1.14048500
C	-5.77621300	-0.23302300	0.91219500
H	-7.37659100	1.25524500	0.86560000
H	-6.18886900	-1.23230200	0.83692300
C	-2.50191500	-2.38419200	0.51907000
C	-3.22569700	-3.51337300	0.78097300
O	-2.14029600	-1.79085600	1.69287900
C	-3.32692700	-3.60947500	2.19880000
H	-3.64070200	-4.18861500	0.04058600
C	-2.65063800	-2.53581000	2.69453600
H	-3.83037400	-4.37756100	2.77422500
H	-2.43163100	-2.18967700	3.69878500
C	-3.59529300	-1.21044600	-1.82852300
C	-4.08135100	-0.05569300	-2.37369000
O	-4.54303900	-2.19269400	-1.91367000
C	-5.40471700	-0.33993500	-2.81941900
H	-3.54610200	0.88788000	-2.43347800
C	-5.62721400	-1.64548400	-2.50929300
H	-6.09959100	0.33576500	-3.30341100
H	-6.47316900	-2.30727600	-2.65101400
C	-1.38604800	-2.91326000	-1.95922600
C	-1.59510600	-3.37167000	-3.22799900
O	-0.37057100	-3.61498800	-1.37662400
C	-0.65106200	-4.42338200	-3.43699800
H	-2.33440400	-3.00133900	-3.93024300
C	0.06323300	-4.52284900	-2.28550600
H	-0.52868500	-5.02987000	-4.32664500
H	0.86902100	-5.16912700	-1.95984200
C	0.89830900	0.38137900	-1.72135600
C	1.69296700	0.27606200	-0.57722400
C	2.50408400	-0.84839700	-0.39352200
C	2.57550800	-1.80159800	-1.40616300
C	1.86178100	-1.61679900	-2.59191000
C	1.00889700	-0.53178100	-2.78716200
H	3.18956800	-2.69288500	-1.27066500
H	1.93598600	-2.36491700	-3.38285500
H	1.66446200	1.05050600	0.18896100
C	3.16149900	-1.03118900	0.92688000
C	2.50228400	-1.86346600	1.88434600
C	4.33131400	-0.37134300	1.27829300
C	1.28402500	-2.53183400	1.59234700
C	3.06321600	-2.01115600	3.18816000
C	4.88106200	-0.53699700	2.57676100

C	0.66216100	-3.30880100	2.53860000
H	0.84245000	-2.43142200	0.60121100
C	2.39472300	-2.81530200	4.14439500
C	4.26613100	-1.33646800	3.50259200
H	5.80197100	-0.01872900	2.83232300
C	1.21849200	-3.45020300	3.82905300
H	-0.26948500	-3.81782800	2.29008600
H	2.83586400	-2.91674500	5.13621100
H	4.69109700	-1.45750600	4.49906400
H	0.71200800	-4.06502900	4.57158500
P	5.10180300	0.80603200	0.14042800
I	0.27814200	2.61798400	-2.24594500
C	0.21053000	-0.39018300	-4.04218700
H	-0.85866400	-0.23901900	-3.82308300
H	0.53803300	0.48232400	-4.62800400
H	0.30836300	-1.27938100	-4.67727800
O	6.55464600	0.98730900	0.80967800
O	5.35340700	0.03782400	-1.24444500
O	4.37094500	2.07645100	-0.08736600
C	7.22974300	2.25712200	0.70916500
C	7.83633500	2.47667600	-0.65260700
H	8.00074400	2.22867300	1.48714200
H	6.52062500	3.05610500	0.96074400
H	8.36210800	3.44001900	-0.66839100
H	8.56043100	1.68979500	-0.89789800
H	7.06757300	2.49958500	-1.43632900
C	6.02818400	-1.23128400	-1.30170600
C	7.48790000	-1.06508700	-1.63509600
H	5.50663300	-1.80282600	-2.07918400
H	5.89609600	-1.77354000	-0.35258000
H	7.94340300	-2.04989700	-1.80017200
H	7.61200900	-0.47190500	-2.55030600
H	8.02765500	-0.56892500	-0.81971500
H	2.33289500	-3.71376900	4.49193400
H	5.03100800	-3.64290400	3.97377200
C	4.10720600	0.72805600	1.57946500
C	4.31446100	0.92079300	2.91521600
O	4.43594100	1.85629000	0.89015400
C	4.79714700	2.25570200	3.05559300
H	4.13496700	0.20096400	3.70713200
C	4.84507700	2.77368900	1.79998700
H	5.07295000	2.76145400	3.97325900
H	5.15824600	3.73021500	1.39946600
C	4.67780100	-1.11988500	-0.47475200
C	4.64560000	-1.48718300	-1.79091100
O	5.97011800	-1.11377800	-0.02927100
C	5.99438600	-1.72965400	-2.17638200
H	3.75588100	-1.57301200	-2.40666200
C	6.74653700	-1.48611500	-1.06938700
H	6.35704500	-2.04056900	-3.14853000
H	7.81077100	-1.52950300	-0.87229900
C	1.09600200	2.51768100	1.22088000
C	1.10120200	1.86349600	2.42016100
O	1.03532200	3.86751500	1.42735600
C	1.05388300	2.86871900	3.42699900
H	1.12351300	0.78701900	2.55966400
C	1.01598400	4.05660800	2.76398300
H	1.04673200	2.72716200	4.50094800
H	0.97316100	5.08752000	3.09394000
C	2.54407700	2.54908400	-1.28504700
C	3.35883300	2.00810400	-2.23774700
O	2.95493900	3.81332500	-0.98075800
C	4.33878100	2.99749000	-2.53396300
H	3.27026900	1.01507700	-2.66557900
C	4.04680500	4.06176500	-1.73890700
H	5.15583200	2.92482800	-3.24147200
H	4.50119700	5.03598600	-1.60627900

Int1

Pd	1.17001100	-0.44642200	-0.48883500
P	3.35380100	-0.63250400	0.64529500
P	1.09471200	1.87921600	-0.45659700
C	3.32084900	-1.86575100	1.95918600
C	2.28139400	-2.32932300	2.71374200
O	4.49960400	-2.36714900	2.43458600
C	2.85241700	-3.16654600	3.71478500
H	1.23179800	-2.09786800	2.56374400
C	4.19409000	-3.15244700	3.49190600
C	-0.22843500	2.77369800	-1.28587700
C	-0.26435100	3.51885900	-2.43127800
O	-1.48883200	2.57297100	-0.81706100
C	-1.64130200	3.78538400	-2.68185800
H	0.58837000	3.82542700	-3.02806100
C	-2.33499000	3.18182800	-1.67675400
H	-2.06063500	4.35077500	-3.50552600
H	-3.39016700	3.08464300	-1.44454300
C	-0.69232100	-0.38976800	-1.35743600
C	-1.78111300	-0.71217300	-0.55377400

C	-3.07862200	-0.77028100	-1.08413100
C	-3.26616000	-0.44988500	-2.42863400
C	-2.17363100	-0.12894300	-3.22742500
C	-0.86846000	-0.12165000	-2.72210600
H	-4.26707600	-0.48696100	-2.85634500
H	-2.32839200	0.09037200	-4.28556700
H	-1.63359900	-0.97649100	0.49768000
C	-4.20100100	-1.33595500	-0.28702100
C	-4.18321000	-2.74623800	-0.02805000
C	-5.26689000	-0.57578300	0.18036200
C	-3.15140500	-3.59598200	-0.50961700
C	-5.24271400	-3.33822400	0.72281400
C	-6.32271400	-1.18541400	0.90902400
C	-3.15327200	-4.94085300	-0.23219100
H	-2.34905500	-3.17530000	-1.11373100
C	-5.21667300	-4.72827600	0.99381800
C	-6.30751900	-2.52665600	1.17658000
H	-7.14446400	-0.57117400	1.26716300
C	-4.19144300	-5.51495100	0.53323300
H	-2.34900500	-5.56989600	-0.61169700
H	-6.03330100	-5.15761600	1.57457500
H	-7.11581200	-2.98808100	1.74381000
H	-4.17970800	-6.58238400	0.74743000
P	-5.30934200	1.21249100	-0.06846300
I	1.10207600	-3.16793500	-0.95814500
C	0.28775400	0.13442700	-3.64274000
H	0.85752800	1.02964300	-3.35356900
H	0.99656600	-0.70814500	-3.63805200
H	-0.05251500	0.28106500	-4.67620700
O	-6.51664500	1.62499300	0.91305400
O	-3.99597100	1.81248800	0.63491600
O	-5.42960000	1.73208300	-1.45266300
C	-6.89147800	3.01622400	0.94557300
C	-7.77648400	3.23055900	2.14146700
H	-7.40665600	3.26260000	0.00751400
H	-5.98489800	3.63721100	1.00744200
H	-8.09388100	4.27922000	2.18977500
H	-8.67380300	2.60213400	2.08047200
H	-7.24435300	2.98754000	3.07008400
C	-3.73818800	1.54458100	2.02286800
C	-2.30559300	1.89461700	2.31325500
H	-3.93054000	0.48102500	2.23646200
H	-4.43238700	2.14043600	2.63441100
H	-2.08605200	1.71323100	3.37315000
H	-1.62757700	1.28453200	1.70259800
H	-2.10584300	2.95099000	2.09034600

NBE-CO_2Me

C	0.50591300	-1.28249700	0.03092300
C	1.99650300	-1.10079300	0.12949100
C	1.06007500	0.89280200	0.56095600
C	-0.05849600	-0.08980900	0.29326900
H	0.00207800	-2.17841700	-0.32532900
C	2.08040800	-0.03410100	1.23176400
H	3.08056300	0.41285900	1.32120300
H	1.75020300	-0.40202100	2.21194400
C	2.38687500	-0.24624600	-1.11457800
H	3.48031200	-0.17193300	-1.18205800
H	2.02617700	-0.69458700	-2.04852100
C	1.74580400	1.12841100	-0.81100300
H	2.50928000	1.91308300	-0.72578100
H	1.02354200	1.44762600	-1.57365000
H	2.58904700	-2.01208700	0.25424600
H	0.76919200	1.80503800	1.09055100
C	-1.45338100	0.31378900	0.11640300
O	-1.83194900	1.46964200	0.10598900
O	-2.27865500	-0.73545900	-0.03546900
C	-3.65227000	-0.40938500	-0.22893200
H	-4.17838600	-1.36215800	-0.32387700
H	-4.04597300	0.15088400	0.62674200
H	-3.78979100	0.18520700	-1.13938500

TFP

P	0.00105500	0.00156800	-1.20803100
C	-0.73381200	1.45781500	-0.42149600
C	-1.27317400	2.56080800	-1.02211200
O	-0.82075600	1.59089200	0.93668800
C	-1.71727100	3.42245700	0.02246400
H	-1.34111700	2.72852400	-2.09161300
C	-1.41552200	2.77944600	1.18203900
H	-2.19688400	4.38907200	-0.07425600
H	-1.55756700	3.03028800	2.22640400
C	-0.89467400	-1.36348000	-0.42484400
C	-1.57988400	-2.38117500	-1.02735100
O	-0.96993900	-1.50583400	0.93304700
C	-2.10724400	-3.19654500	0.01564800
H	-1.68928000	-2.52302500	-2.09712700
C	-1.70338000	-2.61471100	1.17643300

H	-2.70543700	-4.09443400	-0.08303400
H	-1.85291200	-2.86314300	2.22041800
C	1.63008200	-0.09247500	-0.42252200
C	2.85531800	-0.16382300	-1.02412800
O	1.78897200	-0.09924100	0.93571900
C	3.82385100	-0.21926700	0.01975100
H	3.03460600	-0.17541700	-2.09385400
C	3.11594700	-0.17680500	1.18000200
H	4.90099100	-0.28231400	-0.07768500
H	3.40413600	-0.19306100	2.22425500

Int2

Pd	1.69224000	0.69254300	0.01798200
P	2.17749000	-1.56440000	0.31855000
C	1.37131300	-2.58242100	1.57210900
C	1.75885700	-2.95729600	2.82726000
O	0.11560200	-3.03890500	1.31706500
C	0.66371000	-3.68155700	3.38163000
H	2.71137300	-2.73761800	3.29915500
C	-0.30176700	-3.69579800	2.42360700
H	0.60689800	-4.13750400	4.36258600
H	-1.29572600	-4.12376900	2.36794400
C	3.88494000	-1.81033300	0.82945000
C	4.73293400	-0.97910200	1.50369300
O	4.42345000	-3.06241600	0.74970700
C	5.86813400	-1.76408300	1.85317500
H	4.56038400	0.07130700	1.71697000
C	5.62467100	-3.01117500	1.36699400
H	6.75283400	-1.44131000	2.38826100
H	6.19500800	-3.93205400	1.36745600
C	1.84207400	-2.47046800	-1.19373700
C	1.40299600	-2.03614900	-2.41331000
O	1.94265500	-3.83357100	-1.19467200
C	1.23582200	-3.19778100	-3.21704600
H	1.22348900	-1.00341700	-2.69406800
C	1.57763000	-4.25098500	-2.42543300
H	0.90126600	-3.24500600	-4.24608900
H	1.61317800	-5.32101900	-2.59095700
C	-0.05636100	0.12432700	0.95804300
C	-1.10559700	-0.21730900	0.11588200
C	-2.33345600	-0.64575200	0.63664100
C	-2.47065200	-0.76336900	2.01864400
C	-1.41045900	-0.42618100	2.85506500
C	-0.18854700	0.03413400	2.35001800
H	-3.41216500	-1.11537800	2.44250700
H	-1.52793100	-0.51857200	3.93646400
H	-0.99671600	-0.13884100	-0.96851200
C	-3.45258700	-0.96046500	-0.28663100
C	-3.35897900	-2.11296000	-1.13082100
C	-4.58012400	-0.15013200	-0.35787500
C	-2.24577900	-2.99346200	-1.08965600
C	-4.42406000	-2.41348600	-2.03109100
C	-5.63941300	-0.47733700	-1.24719000
C	-2.18676600	-4.09573800	-1.90755700
H	-1.43549400	-2.79630300	-0.39057600
C	-4.33041500	-3.55310900	-2.86686300
C	-5.56107600	-1.57310700	-2.06307500
H	-6.51951500	0.16123100	-1.28245400
C	-3.23439900	-4.37706000	-2.81109000
H	-1.32652100	-4.76213300	-1.85296000
H	-5.15245900	-3.76220400	-3.55184000
H	-6.37473000	-1.81415100	-2.74709400
H	-3.17346500	-5.25253800	-3.45552800
P	-4.74295400	1.34647300	0.65450100
I	3.95744100	0.97410900	-1.68490200
C	0.91201000	0.42925100	3.28863300
H	1.90218900	0.15064700	2.89948100
H	0.93317800	1.51920300	3.44177100
H	0.78177900	-0.03833300	4.27370100
O	-5.98951900	2.12226300	-0.00708100
O	-5.33997700	0.88164500	2.06806000
O	-3.53322300	2.18515100	0.81952500
C	-5.74614900	3.29315500	-0.81233000
C	-5.11737400	2.96161600	-2.14169400
H	-5.12069100	3.99208600	-0.24244000
H	-6.73271400	3.75055000	-0.94464900
H	-5.01094900	3.87888200	-2.73486200
H	-4.11564100	2.52687500	-2.01612700
H	-5.73588000	2.25673200	-2.71169100
C	-6.47683400	0.00445400	2.14972900
C	-6.77698000	-0.22679900	3.60494300
H	-6.24089200	-0.94236900	1.64024100
H	-7.32935800	0.46882600	1.63329700
H	-7.63873100	-0.89732400	3.70694600
H	-5.91915900	-0.68845500	4.11065200
H	-7.01168200	0.71754800	4.11136100
C	0.42929500	2.71822900	0.40119500
C	-0.66777600	2.96417000	-0.59912100

C	1.25607900	3.60406400	-1.57891100
C	1.61511000	3.08324100	-0.20265500
H	0.26777800	2.67597400	1.47638500
C	0.05885900	2.71613400	-1.92608900
H	-0.50570600	3.08560800	-2.79275000
H	0.33409900	1.66260000	-2.08781900
C	-0.78221700	4.51673300	-0.63083000
H	-1.65972400	4.80343800	-1.22580300
H	-0.91162000	4.94048900	0.37324300
C	0.53614700	4.95380600	-1.31101400
H	0.34504700	5.46273600	-2.26464700
H	1.14281300	5.62548500	-0.69141300
H	-1.61302600	2.44827900	-0.40527000
H	2.08292700	3.65622900	-2.29305800
C	2.83759300	3.52486700	0.50314100
O	3.58790200	4.37604400	0.07643900
O	3.00495200	2.90081100	1.67820900
C	4.19514800	3.23378700	2.39078200
H	4.18043800	2.62554000	3.29841500
H	5.08172700	2.99507500	1.79088500
H	4.21060900	4.29764400	2.65204600

TS_2

Pd	-1.76981800	-0.76592400	-0.12963700
P	-2.21076600	1.51927600	0.39933200
C	-1.28231300	2.43071400	1.65811100
C	-1.54542300	2.73114500	2.96442400
O	-0.05163200	2.89680000	1.31303300
C	-0.39835700	3.42357600	3.45235900
H	-2.45247500	2.48940800	3.50949500
C	0.47369800	3.48973400	2.41089000
H	-0.24681200	3.82369100	4.44770600
H	1.46082900	3.91890600	2.28629200
C	-3.86325000	1.72156200	1.08399000
C	-4.66660000	0.81524600	1.71545800
O	-4.39950200	2.97319100	1.19349000
C	-5.76752700	1.55015200	2.24052600
H	-4.48378800	-0.25219500	1.79110500
C	-5.55109500	2.84636300	1.88910500
H	-6.61205000	1.16471400	2.79864200
H	-6.11194400	3.75982200	2.04493200
C	-2.05808200	2.58145800	-1.04135200
C	-1.61385400	2.28443800	-2.29918700
O	-2.33580200	3.91606000	-0.94925000
C	-1.63260200	3.50547200	-3.03124100
H	-1.30797700	1.30333100	-2.64961100
C	-2.07716500	4.45311400	-2.16153600
H	-1.34982500	3.65976900	-4.06549000
H	-2.26170800	5.51626500	-2.25606100
C	0.14970000	-0.60496600	0.81785100
C	1.15996100	-0.11593400	-0.01177100
C	2.28010300	0.53556500	0.51051300
C	2.35255400	0.74325600	1.88653400
C	1.33004000	0.28932900	2.71285700
C	0.22013100	-0.39331600	2.21044800
H	3.21234500	1.26052300	2.31420300
H	1.38966700	0.46808500	3.78755000
H	1.11376400	-0.27688000	-1.08966700
C	3.39185900	0.95112700	-0.38421200
C	3.22384900	2.07451500	-1.25212700
C	4.59896100	0.26113600	-0.38469800
C	2.02095100	2.82604900	-1.28883700
C	4.30058700	2.47787100	-2.09704100
C	5.66521000	0.68612800	-1.22183800
C	1.89129200	3.90996600	-2.12280600
H	1.19785000	2.54237500	-0.63622700
C	4.13413500	3.59543400	-2.95067700
C	5.51904000	1.76134300	-2.05566300
H	6.60690500	0.14178500	-1.19963200
C	2.95528000	4.29811600	-2.96584800
H	0.95973200	4.47568000	-2.13015700
H	4.96649200	3.88692700	-3.59151900
H	6.33977200	2.08133200	-2.69755300
H	2.83865800	5.15832900	-3.62304300
P	4.85229000	-1.19980200	0.65942700
I	-4.01915000	-0.90558900	-1.73488000
C	-0.84556400	-0.86269500	3.15283800
H	-1.84929300	-0.57305800	2.80911400
H	-0.85769400	-1.95901900	3.24926100
H	-0.68959600	-0.44384300	4.15507800
O	6.22472000	-1.83538600	0.10816800
O	5.28389900	-0.66238800	2.10701900
O	3.72877500	-2.16189400	0.74158600
C	6.18602200	-3.03393000	-0.69192900
C	5.66762400	-2.78151700	-2.08448400
H	5.57957500	-3.78747600	-0.17368100
H	7.22227600	-3.38848700	-0.71383900
H	5.71285800	-3.71109300	-2.66607600

H	4.62118600	-2.44660700	-2.07005800
H	6.26970400	-2.02525500	-2.60387300
C	6.31975200	0.32474600	2.25639600
C	6.49651400	0.58978300	3.72593600
H	6.02569100	1.24111700	1.72196100
H	7.24739900	-0.05354800	1.80328000
H	7.27751600	1.34446100	3.87815000
H	5.56472100	0.96351400	4.16933700
H	6.79104900	-0.32428200	4.25613600
C	-0.26279200	-2.58914600	0.35058100
C	0.81151300	-2.98106800	-0.64384400
C	-1.13332000	-3.48016500	-1.66459500
C	-1.50123700	-2.91382000	-0.30518700
H	-0.13670700	-2.83688700	1.40441100
C	0.13320800	-2.68707800	-1.98800400
H	0.68827500	-3.10435000	-2.83849100
H	-0.06471200	-1.62058500	-2.16997400
C	0.78124100	-4.53235700	-0.63841900
H	1.66216600	-4.90997300	-1.17377700
H	0.81905900	-4.93872700	0.38101600
C	-0.52996800	-4.87572800	-1.37982800
H	-0.32962500	-5.38871400	-2.33009300
H	-1.20678100	-5.51050600	-0.79649700
H	1.80221800	-2.56083300	-0.44550700
H	-1.94154200	-3.47124600	-2.40224600
C	-2.66976200	-3.47968700	0.41166400
O	-3.42918000	-4.30045900	-0.05883300
O	-2.79672700	-2.98357200	1.65431500
C	-3.95351100	-3.39903400	2.37524800
H	-3.92333600	-2.85972800	3.32574200
H	-4.86556600	-3.14081600	1.82344500
H	-3.93579600	-4.47948400	2.55690400

Int3

Pd	-1.66247600	-0.60111100	-0.12294800
P	-1.26610700	1.85379900	0.35963800
C	0.21469300	2.66391300	1.03905800
C	0.56198100	3.13746300	2.27139800
O	1.22723700	2.88656500	0.15392400
C	1.87393300	3.68253100	2.13513800
H	-0.04492300	3.10367400	3.16935100
C	2.22670400	3.49895900	0.83422600
H	2.47077400	4.15922400	2.90414200
H	3.10443500	3.75530600	0.25250300
C	-2.41884700	2.11702600	1.72137600
C	-3.11552800	1.18989800	2.44349800
O	-2.69448000	3.36991600	2.19335900
C	-3.86319500	1.90928700	3.41824200
H	-3.09250300	0.11802600	2.27007300
C	-3.56782200	3.22153800	3.21456700
H	-4.53415200	1.50435100	4.16662600
H	-3.89100500	4.13693600	3.69517700
C	-1.68851100	3.09288800	-0.89164400
C	-1.94794500	2.99487200	-2.22883000
O	-1.65670700	4.41291600	-0.52917400
C	-2.09560000	4.32476200	-2.71611700
H	-2.03556900	2.07360900	-2.79316100
C	-1.91044200	5.13594800	-1.64064200
H	-2.31251200	4.63479400	-3.73111100
H	-1.92915600	6.21121400	-1.51055300
C	0.03526300	-1.89372400	0.85895400
C	0.95284100	-1.40112000	-0.09814600
C	1.89028700	-0.40912600	0.18460400
C	1.88114300	0.14704900	1.46168900
C	1.01149900	-0.33055000	2.43128600
C	0.10198300	-1.35809000	2.18183100
H	2.57238400	0.95120200	1.70889000
H	1.05353600	0.09501000	3.43537100
H	0.99694500	-1.86761800	-1.07707400
C	2.90032000	0.01303600	-0.81713300
C	2.49383100	0.56090500	-2.07812200
C	4.25926000	-0.10409700	-0.52985000
C	1.13329400	0.67134600	-2.47491200
C	3.49086200	1.02073500	-2.99187900
C	5.22915400	0.39776100	-1.43602100
C	0.79131100	1.16388100	-3.71071600
H	0.34148000	0.35333200	-1.79533100
C	3.10530000	1.54033700	-4.25216800
C	4.85520300	0.95174700	-2.62933000
H	6.28356000	0.32217600	-1.17996900
C	1.78405000	1.60211300	-4.61302300
H	-0.25842500	1.21915400	-3.99644900
H	3.88720500	1.88331100	-4.92990600
H	5.60363600	1.33026000	-3.32537400
H	1.49674400	1.99324200	-5.58753300
P	4.85446900	-1.03444500	0.91384600
I	-3.98261900	-0.02391400	-1.41675700
C	-0.68791400	-1.86363900	3.35124000

H	-1.59657600	-1.26871600	3.51146800
H	-1.00488300	-2.90555700	3.24402400
H	-0.08261400	-1.78189600	4.26265400
O	6.43446200	-1.18797800	0.64513100
O	4.83360000	-0.04922700	2.17989400
O	4.15272300	-2.30922400	1.18746400
C	6.98052800	-2.46501600	0.26066800
C	6.70899500	-2.79330300	-1.18453000
H	6.57769600	-3.23928400	0.92572500
H	8.05575400	-2.37902600	0.45250100
H	7.18326800	-3.74992900	-1.43845300
H	5.63221200	-2.89079200	-1.37767300
H	7.11588500	-2.02382500	-1.85263300
C	5.32745700	1.29705300	2.08728800
C	5.06694700	1.97019500	3.40673200
H	4.80739500	1.82029900	1.26871900
H	6.40065500	1.27453200	1.84798300
H	5.38228100	3.01994800	3.36333400
H	3.99615600	1.93922300	3.65106400
H	5.61806000	1.47530400	4.21590300
C	-0.82501000	-3.11688500	0.50040400
C	-0.26001000	-4.00460300	-0.63096000
C	-2.27569600	-3.40877500	-1.42996700
C	-2.17753600	-2.66780300	-0.10293600
H	-0.97299800	-3.71645200	1.41047100
C	-0.83148900	-3.38705800	-1.92098500
H	-0.67410000	-4.04010300	-2.79051300
H	-0.48181300	-2.37868000	-2.17489000
C	-1.04034200	-5.32537800	-0.59322900
H	-0.56737800	-6.06488300	-1.25291400
H	-1.07026600	-5.75677100	0.41641900
C	-2.43865000	-4.91718300	-1.11619100
H	-2.68982700	-5.44953100	-2.04394500
H	-3.24331600	-5.11318500	-0.40055900
H	0.82919000	-4.12635600	-0.57954200
H	-3.04040300	-3.01496300	-2.10679900
C	-3.41875900	-2.72623800	0.72179600
O	-4.48670600	-3.16300200	0.34516300
O	-3.24412100	-2.23451800	1.96443000
C	-4.41176100	-2.18221600	2.77978200
H	-4.08895600	-1.78704800	3.74783900
H	-5.16430800	-1.51778500	2.33501400
H	-4.84505400	-3.17969500	2.91040500

Int4

Pd	1.29056500	-0.36539400	-0.58705000
P	2.67398900	0.39799000	1.08058100
C	1.99366300	2.00324900	1.53891200
C	2.49526300	3.27140700	1.54308300
O	0.65917700	2.02463900	1.83331000
C	1.40324600	4.12886700	1.86666300
H	3.51916300	3.55998500	1.33428700
C	0.32257700	3.32106900	2.03253100
H	1.42153600	5.20787400	1.96390600
H	-0.71201900	3.50575200	2.29618400
C	4.36645700	0.72889900	0.60732400
C	5.03630600	0.48888900	-0.55739900
O	5.21730600	1.26469200	1.53538800
C	6.37816500	0.91055700	-0.34121300
H	4.61107500	0.05942700	-1.45815400
C	6.42476700	1.36978500	0.93843600
H	7.20128300	0.87499000	-1.04425600
H	7.21651800	1.78942600	1.54692600
C	2.84091900	-0.40229700	2.69046500
C	2.06548600	-0.34372700	3.81436600
O	3.82693100	-1.32809300	2.84756700
C	2.61679500	-1.28961200	4.72517600
H	1.20533200	0.29752800	3.97237000
C	3.67787000	-1.85301600	4.08755100
H	2.27337500	-1.51318300	5.72788200
H	4.40820300	-2.59914800	4.37427200
C	-0.72726900	-1.50035500	-1.35164200
C	-1.45803000	-0.60228100	-0.54235100
C	-2.23731100	0.42725000	-1.06737300
C	-2.26852200	0.59062900	-2.45306500
C	-1.55615400	-0.27514400	-3.27029100
C	-0.78960400	-1.32852800	-2.75984400
H	-2.86988500	1.38661200	-2.89389800
H	-1.60404500	-0.14579900	-4.35182200
H	-1.48261300	-0.75296700	0.53330300
C	-3.03949000	1.29597500	-0.16642300
C	-2.72317800	2.68916500	-0.07268400
C	-4.08709000	0.78348800	0.59262900
C	-1.65818400	3.27505200	-0.80415300
C	-3.47367400	3.52508100	0.80795800
C	-4.81698800	1.63013900	1.46912100
C	-1.36351200	4.61077500	-0.68289400
H	-1.05593300	2.65424000	-1.46514300

C	-3.14759600	4.90006300	0.91124300
C	-4.52120300	2.96163900	1.57228400
H	-5.63109300	1.21067500	2.05689900
C	-2.11621000	5.43573800	0.18034600
H	-0.53342400	5.03126500	-1.25014800
H	-3.73555000	5.52169200	1.58682500
H	-5.08815600	3.60569300	2.24436700
H	-1.87319800	6.49345600	0.26860600
P	-4.51910000	-0.97409100	0.62919200
I	1.68207100	1.84182400	-2.32549200
C	-0.08397500	-2.21136900	-3.74329400
H	0.97494300	-1.93426100	-3.83452100
H	-0.11027200	-3.27204300	-3.46502000
H	-0.54011100	-2.11010500	-4.73507900
O	-6.08370800	-0.90204100	0.98959400
O	-4.47532100	-1.55341200	-0.86657600
O	-3.70390100	-1.81609000	1.53987100
C	-6.67535200	-1.92247000	1.82006700
C	-6.68855100	-1.47893800	3.25845800
H	-6.13171000	-2.86817500	1.69647600
H	-7.69010700	-2.06409000	1.43143300
H	-7.17154900	-2.23956300	3.88500700
H	-5.66556100	-1.32762500	3.62708600
H	-7.24465200	-0.53926700	3.37154000
C	-5.30623800	-0.99235200	-1.89823400
C	-5.05069600	-1.75873200	-3.16610600
H	-5.06199300	0.07469700	-2.02149000
H	-6.35816600	-1.06623600	-1.59044300
H	-5.66609500	-1.35410900	-3.97884400
H	-3.99506700	-1.68320200	-3.45994000
H	-5.30125000	-2.81919300	-3.03817200
C	-0.06136900	-2.72417500	-0.69427700
C	-0.82846600	-3.31458100	0.51497100
C	1.22955100	-2.95001400	1.34641800
C	1.30706500	-2.40544700	-0.06375500
H	0.05651800	-3.49038400	-1.47350400
C	-0.20526800	-2.62139600	1.74060900
H	-0.50739400	-3.10359500	2.68093900
H	-0.38835500	-1.54363400	1.82934800
C	-0.29142500	-4.73852900	0.71130600
H	-0.88480500	-5.26708700	1.46936700
H	-0.33180600	-5.33144200	-0.21197600
C	1.15412800	-4.49383000	1.20954700
H	1.32893100	-4.95480700	2.19071000
H	1.91740900	-4.89049900	0.52697300
H	-1.91783600	-3.23411400	0.42263400
H	2.03608600	-2.62199300	2.00449800
C	2.59192000	-2.68904200	-0.76465700
O	3.66369400	-2.80876600	-0.20283600
O	2.44736100	-2.77790500	-2.09858100
C	3.65242900	-2.90627200	-2.85024500
H	3.34875200	-2.97255700	-3.89808900
H	4.29449800	-2.02945000	-2.70086000
H	4.20009000	-3.80965900	-2.56096200

Int5

C	0.06445600	2.50009400	0.18529400
O	0.12807800	1.18751200	0.19469000
O	-1.07325000	3.07667700	0.34880900
O	1.14134400	3.18099000	0.01049000
K	-2.41562900	0.87032800	0.45847400
K	2.66300000	1.11044800	-0.25989600
O	-4.35680200	-0.83709500	0.22857400
S	-5.49874000	-1.49593500	-0.52630800
C	-6.68843800	-0.18690300	-0.88608800
H	-6.92214200	0.35283600	0.04030100
H	-6.23409900	0.49358900	-1.61417300
H	-7.59602700	-0.62811400	-1.31531700
C	-6.49913800	-2.33641400	0.71839300
H	-6.75561300	-1.62593800	1.51406700
H	-7.40561500	-2.73477300	0.24752100
H	-5.90497500	-3.16031100	1.12773500
O	4.70245700	-0.42887500	-0.80067700
S	5.51688000	-1.66521800	-0.45944100
C	7.15875000	-1.07248400	-0.00119100
H	7.75903400	-1.91246700	0.36809400
H	7.62689100	-0.65254700	-0.89779200
H	7.06028200	-0.29783900	0.76965600
C	5.01633900	-2.16060100	1.20362400
H	5.66406200	-2.97425000	1.55150600
H	5.09034300	-1.29685700	1.87653400
H	3.98112900	-2.51543100	1.15559100

Int6

Pd	1.01772700	0.53948000	0.76303400
P	2.63270000	-1.08285900	0.38115300
C	1.86337100	-2.69842200	0.67210200
C	2.01567000	-3.64668600	1.64000600

O	0.83235300	-3.03756800	-0.15529700
C	1.02023300	-4.63952700	1.38651600
H	2.74627800	-3.64143800	2.44041400
C	0.33633100	-4.21926000	0.28694600
H	0.84579800	-5.55263100	1.94453200
H	-0.49539900	-4.62385200	-0.27741000
C	3.77303900	-0.91941600	1.76165500
C	3.89623700	0.07519100	2.68978900
O	4.73795200	-1.86785900	1.96463700
C	4.99490800	-0.28547100	3.52191300
H	3.27885800	0.96734600	2.74395000
C	5.46027200	-1.46690900	3.03507700
H	5.38930900	0.26368200	4.36818100
H	6.26979800	-2.12323300	3.33019000
C	3.75653600	-1.49004700	-0.98343500
C	3.67475900	-2.38531900	-2.01200200
O	4.86825300	-0.71549500	-1.12627900
C	4.81173700	-2.14676400	-2.83840700
H	2.89884400	-3.12830300	-2.16177800
C	5.49580800	-1.12785300	-2.25419900
H	5.08746400	-2.67385600	-3.74383300
H	6.41759100	-0.61352500	-2.49555400
C	-0.51429300	2.15662000	-2.00073200
C	-1.29529500	1.62457100	-0.97374400
C	-2.56226100	1.08258100	-1.17968000
C	-3.09341300	1.07989100	-2.46580600
C	-2.38016800	1.70470800	-3.48170300
C	-1.12135500	2.28212200	-3.27416100
H	-4.07596300	0.64838600	-2.65962000
H	-2.82653300	1.78583900	-4.47434000
C	-3.33525000	0.58748300	-0.01207200
C	-3.87654900	1.55154200	0.90011500
C	-3.56718100	-0.76157100	0.22158000
C	-3.69201600	2.94789800	0.72308200
C	-4.64090800	1.11279600	2.02137100
C	-4.35981500	-1.18002800	1.32790700
C	-4.21544300	3.85087600	1.61651600
H	-3.12621300	3.30666900	-0.13474800
C	-5.16875000	2.06622000	2.92466200
C	-4.87702300	-0.26950300	2.20533000
H	-4.55766400	-2.24269400	1.46970500
C	-4.95805800	3.40852700	2.73213700
H	-4.06140200	4.91746400	1.45966500
H	-5.74864200	1.70788800	3.77555800

H	-5.47965000	-0.59383300	3.05374100
H	-5.36775800	4.13464300	3.43239900
P	-2.92723400	-2.12308300	-0.75873500
C	-0.54450100	3.06677700	-4.42299100
H	0.09581900	3.89389500	-4.09217800
H	0.04783000	2.44786500	-5.11214100
H	-1.36016400	3.50058400	-5.01635500
O	-3.96023300	-2.30457100	-1.97387700
O	-1.58368100	-1.56914600	-1.40688800
O	-2.75178400	-3.36137400	0.06049700
C	-4.20182000	-3.61941100	-2.52329100
C	-4.94345000	-3.44138800	-3.81802100
H	-4.78325100	-4.19988800	-1.79522900
H	-3.24567400	-4.13805800	-2.68154000
H	-5.16129600	-4.42073900	-4.26102300
H	-5.89344300	-2.91711600	-3.65594000
H	-4.34534400	-2.86352800	-4.53446900
C	-1.04955000	-2.13482000	-2.61483700
C	0.17967700	-1.35613500	-2.99275800
H	-1.81596900	-2.07543900	-3.40326400
H	-0.81135300	-3.19585600	-2.44332800
H	0.65160000	-1.80314200	-3.87705800
H	-0.07145700	-0.31062300	-3.22083700
H	0.90340400	-1.36815600	-2.16706600
C	0.87488900	2.70061800	-1.69604700
C	1.96452400	2.44423300	-2.78140400
C	3.07563400	2.10557800	-0.86872100
C	1.63389300	2.28207900	-0.38015200
H	0.73689000	3.79644900	-1.63531400
C	2.84192600	1.35521100	-2.17750200
H	3.76121900	1.18646500	-2.75819100
H	2.31313600	0.40263400	-2.05020600
C	2.92746800	3.63521400	-2.75346100
H	3.64856100	3.56579000	-3.58022800
H	2.40923700	4.60020100	-2.84792300
C	3.63185900	3.45355300	-1.38592900
H	4.72330900	3.39595400	-1.50228400
H	3.42688400	4.27433100	-0.68533500
H	1.57322200	2.20924800	-3.77284800
H	3.73283200	1.63556100	-0.12794100
C	1.50617500	3.20797800	0.77214200
O	2.33232500	3.34629300	1.66255200
O	0.34783300	3.90043400	0.76115200
C	0.07202000	4.65669100	1.93148000

H	-0.92853600	5.07592400	1.79315700
H	0.08677400	4.01429800	2.81845400
H	0.79797200	5.46889200	2.05960600
C	-0.71593900	0.15498900	2.59624400
O	-1.60896000	-0.10970100	3.41396300
O	0.09656600	-0.78466400	2.13955600
O	-0.48795000	1.34085800	2.09873000
K	-1.57311200	-2.77019500	2.47511000
H	-0.94017500	1.66252100	0.05246100

Int7

K	-0.05245300	-0.76119500	0.03985300
I	0.14412200	2.74124000	0.01807800
O	-2.35200500	-1.89140600	0.15794200
S	-3.83940300	-1.94337700	-0.15071200
C	-4.66856300	-1.17645700	1.25646700
H	-5.73928300	-1.08189500	1.03984000
H	-4.52608100	-1.82614200	2.12648600
H	-4.22352400	-0.19136700	1.44513800
C	-4.16261300	-0.60752100	-1.32139900
H	-5.24253800	-0.53435100	-1.49764200
H	-3.77585700	0.33570700	-0.91498200
H	-3.65579900	-0.85090400	-2.26133000
O	2.17109500	-2.01845300	-0.18760300
S	3.67008900	-2.16189800	0.01842900
C	4.10006000	-1.06962700	1.38991600
H	3.72591900	-0.05872200	1.18371800
H	3.63369700	-1.46781400	2.29738400
H	5.18938300	-1.05629800	1.51519000
C	4.45109000	-1.17425500	-1.27405100
H	4.03763900	-0.15802100	-1.25501900
H	5.53488900	-1.15294900	-1.10903000
H	4.23614900	-1.64982900	-2.23689000

Int8

Pd	1.22853100	0.02106700	0.62770000
P	3.35711100	-0.58329000	-0.06055100
C	3.32784400	-2.11403700	-1.02097400
C	3.60934000	-3.41085900	-0.70978800
O	2.94295900	-2.01612400	-2.32693300
C	3.39914900	-4.16136400	-1.90472200
H	3.93543800	-3.78438700	0.25448500
C	3.00512000	-3.26778300	-2.84853100
H	3.53289900	-5.22760600	-2.04260800
H	2.75581100	-3.35536700	-3.89858600
C	4.19132300	-1.08776500	1.45313200
C	3.76842500	-1.11045000	2.75148500
O	5.48585700	-1.52557100	1.37894400
C	4.86257900	-1.59452600	3.52319700
H	2.77984000	-0.83081100	3.09933100
C	5.86916100	-1.82776300	2.63853100
H	4.89656900	-1.74829800	4.59489200
H	6.88351400	-2.19161600	2.74877700
C	4.69144100	0.29938700	-0.92086400
C	5.17987900	0.24088400	-2.19475400
O	5.33943000	1.27366200	-0.22008000
C	6.19051800	1.24242000	-2.28221100
H	4.85735400	-0.43345300	-2.97914900
C	6.24090900	1.83581200	-1.06053300
H	6.80290200	1.48323400	-3.14278200
H	6.84813800	2.62731600	-0.63960400
C	-0.99130200	2.09557700	-1.43338400
C	-1.39896400	0.83325800	-1.01365900
C	-2.52158000	0.18218600	-1.52846900
C	-3.24530500	0.79162000	-2.54602200
C	-2.88220600	2.07089100	-2.95299500
C	-1.79557200	2.75279000	-2.39787800
H	-4.10498200	0.29159400	-2.99258400
H	-3.48139000	2.57969700	-3.70970500
C	-2.84276900	-1.16072900	-0.97684400
C	-2.04786600	-2.26378800	-1.42728100
C	-3.80374500	-1.37680000	0.00228500
C	-1.07745300	-2.11582700	-2.45249600
C	-2.22950000	-3.55545600	-0.85187000
C	-3.98139700	-2.67828500	0.55688300
C	-0.32692500	-3.18633000	-2.87191200
H	-0.94119000	-1.14410500	-2.92332700
C	-1.42515700	-4.63532600	-1.28989300
C	-3.21348200	-3.73269700	0.14860600
H	-4.73577400	-2.83041300	1.32859100
C	-0.48856900	-4.45614600	-2.27699200
H	0.39294200	-3.05560400	-3.67761600
H	-1.57156500	-5.61179200	-0.82796800
H	-3.34867600	-4.72121800	0.58719100
H	0.12626700	-5.29091700	-2.61105400
P	-4.93980400	-0.17520700	0.73400800
C	-1.59752000	4.18887500	-2.79720500
H	-1.26579200	4.80815700	-1.95217300

H	-0.85657500	4.31959500	-3.59794000
H	-2.54162200	4.61037300	-3.16567600
O	-4.56768300	1.31532000	0.29924100
O	-6.36105300	-0.40342000	0.04735600
O	-5.01866000	-0.36707200	2.20973100
C	-3.65015100	2.06758600	1.12261700
C	-3.60969600	3.47954000	0.60800000
H	-2.65151200	1.60250700	1.06810300
H	-4.01284600	2.04059700	2.16103200
H	-2.88324100	4.06555800	1.18498500
H	-3.30837400	3.49766300	-0.44727000
H	-4.59330300	3.95710100	0.70013400
C	-6.54036800	-0.23722000	-1.37439600
C	-7.95883300	-0.60632200	-1.70658300
H	-5.82533400	-0.88623900	-1.90290700
H	-6.32194700	0.80666600	-1.63793000
H	-8.13064700	-0.48610400	-2.78292800
H	-8.16409000	-1.64997500	-1.43848900
H	-8.66615400	0.03809200	-1.17026600
C	0.18542100	2.81618300	-0.80298400
C	1.21877900	3.36345400	-1.83938700
C	2.49738900	2.69032600	-0.13532300
C	1.12148500	2.16947800	0.28079000
H	-0.27160900	3.69644000	-0.31043200
C	2.43689400	2.46350600	-1.64479800
H	3.32311000	2.83145100	-2.18362600
H	2.24675300	1.41721700	-1.92603600
C	1.75254000	4.69261400	-1.30256900
H	2.41156000	5.17069400	-2.04119500
H	0.95058300	5.40556200	-1.06481600
C	2.54922100	4.23481200	-0.05766400
H	3.59333900	4.57694600	-0.09958000
H	2.12646900	4.60801900	0.88512300
H	0.83656600	3.40610700	-2.86358400
H	3.31694500	2.23069400	0.42113600
C	0.79749500	2.45147600	1.69938700
O	1.61054500	2.50996500	2.60908000
O	-0.52601500	2.62608500	1.92416200
C	-0.89249000	2.69391200	3.29465000
H	-1.98494800	2.76098500	3.32606900
H	-0.54210100	1.80357600	3.83262200
H	-0.46702000	3.58064800	3.77913600
C	-0.16382700	-1.59845500	2.02397900
O	-0.81611600	-2.35833100	2.75942900

O	0.89959900	-1.96353500	1.36585200
O	-0.50171100	-0.32866100	1.86061500
K	-2.68492900	-0.62456300	3.42883800
H	-0.86335500	0.33594000	-0.20829500

TS_3

Pd	0.88501000	0.33057300	0.10666600
P	2.48897400	-1.31835300	-0.16887600
C	1.74872000	-2.76391400	-0.97162500
C	1.70857500	-4.09165900	-0.65755900
O	1.03197300	-2.53076700	-2.10523500
C	0.90841700	-4.71370500	-1.66593600
H	2.19116000	-4.56873600	0.18831000
C	0.52802300	-3.71780300	-2.51327500
H	0.66077700	-5.76573100	-1.75350800
H	-0.07266700	-3.69305200	-3.41299400
C	2.96509600	-1.96308800	1.43612900
C	2.49188600	-1.66518800	2.68125600
O	3.93494600	-2.92460700	1.51845300
C	3.21146000	-2.49458500	3.58902000
H	1.70888400	-0.94235600	2.90248100
C	4.06694800	-3.23052300	2.82918800
H	3.10891700	-2.53604400	4.66671800
H	4.80941200	-3.98301500	3.06508200
C	4.04599600	-1.20998300	-1.08617000
C	4.31721800	-1.45313300	-2.40409400
O	5.12896200	-0.65329200	-0.48167500
C	5.65806500	-1.02228200	-2.61938000
H	3.64184200	-1.88508200	-3.13440000
C	6.09641600	-0.54898300	-1.42208500
H	6.22335500	-1.06774800	-3.54233900
H	7.03957300	-0.13805000	-1.08423200
C	-0.30411800	3.06259200	0.08017900
C	-0.83718800	1.78908600	0.42973300
C	-2.24232000	1.61348100	0.35455200
C	-3.06814600	2.69373400	0.03353000
C	-2.53497700	3.95571000	-0.16871900
C	-1.15829500	4.16504100	-0.14208700
H	-4.14982700	2.55014900	0.01040700
H	-3.19862000	4.80317500	-0.34765100
C	-3.00889300	0.40362100	0.79092300
C	-3.51650200	0.41181400	2.13585700
C	-3.41079400	-0.61074200	-0.06300500
C	-3.19313000	1.44339700	3.05499700

C	-4.38417500	-0.62683300	2.57993400
C	-4.28313000	-1.64128400	0.39771700
C	-3.69868300	1.44383500	4.33163500
H	-2.52682700	2.24487000	2.74225800
C	-4.88705400	-0.60617900	3.90369100
C	-4.75394300	-1.65355900	1.67948200
H	-4.60352400	-2.42020400	-0.29577600
C	-4.55373900	0.40808000	4.76546900
H	-3.43546100	2.24820500	5.01685300
H	-5.55028800	-1.41260200	4.21799200
H	-5.42977600	-2.43898600	2.01835200
H	-4.94752900	0.41902800	5.78063300
P	-2.92921400	-0.90167100	-1.77574500
C	-0.66575300	5.56974500	-0.35958600
H	0.29094300	5.77037000	0.13804500
H	-0.52998800	5.80537100	-1.42515400
H	-1.40090600	6.28665100	0.02920200
O	-4.36733000	-0.81750200	-2.49916800
O	-2.08342400	0.30389800	-2.39656100
O	-2.25519700	-2.21633500	-1.96537100
C	-4.50935300	-1.42869200	-3.79720700
C	-5.77844100	-0.91124300	-4.41488300
H	-4.53482900	-2.51919000	-3.66828600
H	-3.63704500	-1.18686900	-4.42364500
H	-5.93214400	-1.37110800	-5.39863300
H	-6.64377800	-1.15022800	-3.78402700
H	-5.73506300	0.17783000	-4.54513300
C	-2.72509700	1.42195900	-3.04490300
C	-1.66507300	2.42801700	-3.39533200
H	-3.47734400	1.85430100	-2.37052500
H	-3.24086500	1.06233100	-3.94586800
H	-2.12832500	3.30413900	-3.86681300
H	-1.13151600	2.75983300	-2.49556300
H	-0.93716300	2.00286000	-4.09868300
C	1.17243000	3.27121900	-0.14015400
C	1.51709200	3.71257400	-1.60277300
C	3.00468600	2.08967900	-1.19985200
C	2.09612500	2.03548800	0.02576600
H	1.51494500	4.07433100	0.53619600
C	1.97853100	2.42098300	-2.28146700
H	2.42314500	2.60751300	-3.27015800
H	1.19438000	1.65447000	-2.37379800
C	2.83652600	4.48756600	-1.53850600
H	3.07349400	4.92317000	-2.51938000
H	2.80207300	5.31085600	-0.81098300
C	3.85178000	3.38172400	-1.16020200
H	4.67297200	3.32305300	-1.88822200
H	4.30720600	3.53766000	-0.17292300
H	0.70808200	4.24711200	-2.11125200
H	3.60912400	1.19102100	-1.34966700
C	2.79854600	1.90137400	1.32450100
O	3.89113700	1.38730300	1.49068400
O	2.07808800	2.39911700	2.35501800
C	2.62341800	2.17686000	3.64845800
H	1.89939700	2.58509900	4.35817700
H	2.76566000	1.10539400	3.83722400
H	3.58644700	2.68810000	3.76619400
C	-0.68383300	-1.18184000	1.89413100
O	-1.13851700	-2.16429800	2.51873500
O	-0.36283800	-1.31724600	0.61898700
O	-0.49562600	-0.01199000	2.44199500
K	-1.47962100	-3.60380200	0.19969800
H	-0.45967800	0.95995100	1.41294500

TS_4

Pd	0.98260300	0.22386300	-0.32774400
P	2.91164900	-0.94402400	0.20248400
C	3.02028400	-2.41380500	-0.83371000
C	3.56736700	-3.65392800	-0.68063400
O	2.52718600	-2.27597600	-2.09915000
C	3.38596900	-4.32752500	-1.92274300
H	4.04222900	-4.03714700	0.21498700
C	2.75306100	-3.44389000	-2.74046600
H	3.68638500	-5.33794100	-2.17289700
H	2.41690700	-3.49248700	-3.76863300
C	2.74055700	-1.53178200	1.90540200
C	2.69105100	-0.80541100	3.06101600
O	2.52329900	-2.84795000	2.16718700
C	2.42478400	-1.74129500	4.10453400
H	2.81889300	0.27056900	3.13471700
C	2.32530800	-2.95618900	3.50060200
H	2.32416700	-1.53622800	5.16411400
H	2.13725400	-3.95722400	3.86814700
C	4.63037600	-0.37717300	0.10206100
C	5.53568900	-0.47466200	-0.91829200
O	5.12182000	0.41940900	1.08752200
C	6.65891500	0.31082600	-0.52847900
H	5.41510100	-1.04233600	-1.83514700

C	6.35106300	0.82569300	0.69211800
H	7.57913300	0.46247500	-1.07975400
H	6.88642300	1.45681600	1.39046000
C	-0.73646600	2.45336500	-1.30605900
C	-1.01279500	1.12146300	-0.89528400
C	-2.19080100	0.50601900	-1.37959900
C	-3.06594400	1.20492000	-2.20949800
C	-2.83676800	2.53990800	-2.49798900
C	-1.68604800	3.18621200	-2.05002700
H	-3.96082400	0.70781200	-2.58720100
H	-3.56514100	3.10207700	-3.08500200
C	-2.59768500	-0.89104300	-1.05480800
C	-2.18875900	-1.92961600	-1.95031200
C	-3.43299600	-1.20139300	0.00625200
C	-1.34967900	-1.67234700	-3.06510000
C	-2.62694500	-3.26770500	-1.73077200
C	-3.85945300	-2.54234900	0.21291000
C	-0.95703200	-2.68569800	-3.90580900
H	-1.00658000	-0.65478100	-3.24843100
C	-2.20739400	-4.29218500	-2.61434100
C	-3.47309200	-3.54623800	-0.63104000
H	-4.50563800	-2.77022900	1.06083200
C	-1.38657500	-4.01154000	-3.67832800
H	-0.31296100	-2.46514600	-4.75604500
H	-2.55059200	-5.31032200	-2.42787900
H	-3.80534600	-4.57146100	-0.46680600
H	-1.06837100	-4.80685700	-4.35076800
P	-4.13810500	-0.06958300	1.22893800
C	-1.54702500	4.65389200	-2.35362100
H	-0.95314700	5.18879000	-1.60149000
H	-1.07957700	4.84572200	-3.32992500
H	-2.54043400	5.12135600	-2.38442400
O	-3.67463100	1.42963100	0.93792200
O	-5.70365200	-0.03821600	0.85223600
O	-3.99945800	-0.51909600	2.64082000
C	-2.85658600	2.17440900	1.86453200
C	-2.88458400	3.62098300	1.45352200
H	-1.83476300	1.77470900	1.83029100
H	-3.25998400	2.04390100	2.87937900
H	-2.27898700	4.21487500	2.15028900
H	-2.46638100	3.74667500	0.44489700
H	-3.90778200	4.01776600	1.46158900
C	-6.14481100	0.37696800	-0.44878900
C	-7.64612200	0.28763500	-0.48108700

H	-5.69393500	-0.27853000	-1.21082600
H	-5.80163300	1.40470400	-0.63653600
H	-8.01898800	0.59624700	-1.46525800
H	-7.97938700	-0.74060800	-0.29280200
H	-8.09281600	0.94197500	0.27787800
C	0.58105600	3.10807700	-0.98834600
C	1.38258200	3.56749800	-2.24920000
C	2.92241900	2.47780600	-1.03983100
C	1.63076000	2.22032300	-0.27181600
H	0.37869500	4.00576100	-0.37484700
C	2.39381200	2.44037000	-2.47360800
H	3.14904200	2.70406000	-3.22821800
H	1.93691400	1.47442700	-2.73758900
C	2.31182600	4.70047500	-1.80392000
H	2.81701300	5.14585100	-2.67239600
H	1.77039100	5.50620100	-1.28809800
C	3.32190300	3.96349400	-0.89040900
H	4.35616100	4.11736500	-1.22842100
H	3.27265500	4.29323200	0.15630900
H	0.75576500	3.81546700	-3.11122900
H	3.73647800	1.78959300	-0.79104200
C	1.72086400	2.35153700	1.19975900
O	2.72735200	2.17386300	1.86810600
O	0.53591300	2.67408400	1.77054500
C	0.50588400	2.60150400	3.18887400
H	-0.50814400	2.87692400	3.49392400
H	0.73895300	1.58287100	3.52781100
H	1.22494200	3.29495100	3.64028600
C	-0.56352600	-1.51192000	1.21982700
O	-0.84595400	-2.43129400	2.01500100
O	0.15877500	-1.71628600	0.15403900
O	-0.99164100	-0.27446300	1.44760500
K	-1.73031800	-0.86167600	3.92095100
H	-0.83768400	0.53260700	0.30294600

Int9

Pd	0.69192000	0.44871600	0.34230400
P	2.49080200	-1.08695600	-0.50504100
C	1.69647700	-2.16347600	-1.73612300
C	1.76619200	-3.48957900	-2.05300300
O	0.85444600	-1.52664300	-2.60291600
C	0.91742900	-3.67736300	-3.18879600
H	2.36351100	-4.24114500	-1.54944200
C	0.39731100	-2.45327300	-3.47839800

H	0.72767000	-4.60186100	-3.72250400
H	-0.28998100	-2.09389700	-4.23299500
C	3.28652800	-2.28827800	0.58017200
C	3.19930700	-2.49238200	1.92676900
O	4.21001000	-3.14832200	0.04355900
C	4.10819200	-3.54418000	2.23899200
H	2.53926700	-1.96537600	2.60695600
C	4.68842900	-3.89765400	1.06157300
H	4.30597400	-3.97703900	3.21215800
H	5.43632400	-4.63440100	0.79462900
C	3.90367800	-0.57651200	-1.53393000
C	4.07948900	-0.53149500	-2.88857300
O	4.95313100	0.02228200	-0.90928600
C	5.31654500	0.14442600	-3.10770300
H	3.41018300	-0.93001300	-3.64353700
C	5.79806900	0.45476600	-1.87492500
H	5.78795500	0.36145400	-4.05882300
H	6.70016800	0.94375900	-1.52843300
C	-0.58693500	3.11136500	0.65491400
C	-0.89267000	1.72959000	0.68536400
C	-2.23567900	1.36085700	0.89263200
C	-3.21047900	2.34860700	1.09058800
C	-2.88153000	3.69564800	1.09452700
C	-1.56479300	4.09954100	0.88090200
H	-4.24240400	2.04174600	1.27562800
H	-3.65368100	4.44628200	1.27111600
C	-2.73930900	-0.02705100	1.10201900
C	-2.75933400	-0.50280500	2.45685800
C	-3.31414000	-0.80511500	0.11170000
C	-2.23331000	0.26412100	3.52957800
C	-3.32608300	-1.77497900	2.75873900
C	-3.90039900	-2.06596000	0.43454800
C	-2.25626600	-0.20993300	4.81910600
H	-1.80404000	1.24388200	3.32406200
C	-3.32952500	-2.24172200	4.09439500
C	-3.90022800	-2.54165300	1.71521600
H	-4.37423100	-2.65439600	-0.35322600
C	-2.80463300	-1.47698000	5.10676900
H	-1.84827300	0.39729500	5.62578000
H	-3.76789400	-3.21846600	4.30130300
H	-4.35478500	-3.50366400	1.95286800
H	-2.81690300	-1.84201600	6.13258100
P	-3.27100100	-0.55037100	-1.66962000
C	-1.24387400	5.56639200	0.86377500
H	-0.27444500	5.79329100	1.32845100
H	-1.20109100	5.96214400	-0.16256400
H	-2.01520700	6.13940900	1.39478100
O	-4.83797900	-0.48693500	-2.02383100
O	-2.67615400	0.86715600	-2.09597900
O	-2.54368200	-1.65978700	-2.35206300
C	-5.23557200	-0.71932400	-3.39228500
C	-6.64043400	-0.21140800	-3.55742100
H	-5.16482100	-1.79626700	-3.59512300
H	-4.54380800	-0.20340300	-4.07535700
H	-6.98575100	-0.39055500	-4.58281200
H	-7.32331300	-0.72504000	-2.86917600
H	-6.69314600	0.86666100	-3.35804700
C	-3.50552400	2.04007600	-2.23594600
C	-2.60247500	3.21259900	-2.49968300
H	-4.09144200	2.18592600	-1.31818300
H	-4.20430400	1.88088800	-3.06886900
H	-3.20015400	4.12824700	-2.59350100
H	-1.89131600	3.34442100	-1.67425600
H	-2.03796900	3.07134800	-3.43067300
C	0.82637600	3.49064100	0.37033700
C	1.05888900	4.15016500	-1.01904100
C	2.53819700	2.46544500	-0.98535600
C	1.75947000	2.25585200	0.31483500
H	1.18012800	4.19439700	1.14371600
C	1.42777700	2.96305700	-1.91239200
H	1.79507400	3.27895300	-2.90003000
H	0.61829200	2.22945700	-2.04536600
C	2.39798700	4.89078200	-0.95240600
H	2.56162600	5.48016800	-1.86559600
H	2.44455700	5.58361800	-0.09982300
C	3.41737600	3.72836500	-0.84885700
H	4.15445200	3.76442500	-1.66388200
H	3.98114800	3.73276400	0.09405700
H	0.21936800	4.76856700	-1.36015700
H	3.10209300	1.59761700	-1.32818200
C	2.57463300	1.96959700	1.51960300
O	3.63787100	1.37100900	1.53199600
O	2.00045900	2.41707700	2.66341300
C	2.61582300	1.97420400	3.86471900
H	2.03836800	2.41220200	4.68303500
H	2.58226900	0.87842400	3.93434400
H	3.65928900	2.30330800	3.92836500
C	-0.12840100	-2.27861600	1.62078900

O	-0.29375500	-3.49933300	1.68330200
O	-0.27711100	-1.55531800	0.57801700
O	0.24938700	-1.63722500	2.76644600
K	-1.28122700	-3.50743200	-0.89565000
H	0.28075800	-0.68276200	2.55926600

Int10

Pd	-0.61589700	-0.77502600	-0.07982400
C	-0.19892300	-3.29628800	1.36463400
C	0.25992600	-1.96550700	1.27511100
C	1.28611000	-1.53807900	2.13150400
C	1.78483400	-2.41910700	3.09975100
C	1.30269900	-3.71743900	3.19833300
C	0.31245100	-4.18115500	2.32936700
H	2.56332500	-2.07309100	3.78319500
H	1.69709300	-4.38624300	3.96504700
C	1.87565000	-0.17136800	2.08493200
C	1.53080400	0.76321900	3.11291300
C	2.79188800	0.20466100	1.10903900
C	0.55318000	0.47071500	4.10037200
C	2.15704700	2.04484100	3.14318800
C	3.38758200	1.49581800	1.13725900
C	0.23569400	1.38617900	5.07474400
H	0.05073000	-0.49497900	4.08040600
C	1.81978000	2.96260900	4.16805200
C	3.09034100	2.38268300	2.13600800
H	4.09732300	1.77287900	0.36122100
C	0.88199900	2.64080600	5.11779500
H	-0.52116000	1.14434400	5.81933100
H	2.31892300	3.93173300	4.18112500
H	3.56144300	3.36518200	2.16192400
H	0.62631300	3.35410800	5.90007700
P	3.17481000	-0.83743100	-0.31355600
C	-0.20689100	-5.58266200	2.46258400
H	-1.29940800	-5.60140800	2.58970300
H	0.01353900	-6.19649700	1.57762000
H	0.23860200	-6.08516600	3.33047200
O	4.60995700	-0.25992100	-0.73609100
O	3.45006000	-2.34653900	0.14401500
O	2.16889100	-0.83095800	-1.41484900
C	5.15892700	-0.66480200	-2.00643200
C	6.54686100	-0.09790100	-2.10507300
H	4.50989300	-0.28368700	-2.80814000
H	5.16747000	-1.76409100	-2.06290000
H	7.00018300	-0.38119700	-3.06259200
H	6.52034700	0.99803600	-2.04611400
H	7.18340900	-0.47646300	-1.29510400
C	4.52673100	-2.64537200	1.05323400
C	4.53845900	-4.13105600	1.27844600
H	4.36508800	-2.09790100	1.99475400
H	5.47372200	-2.29856300	0.61464300
H	5.33889300	-4.39739300	1.97926500
H	3.58115000	-4.46584200	1.69954200
H	4.71170800	-4.66672600	0.33669900
C	-1.25800200	-3.69824900	0.39500400
C	-0.77791900	-4.60763900	-0.77193300
C	-1.71156700	-2.86326300	-1.83864100
C	-1.80446000	-2.46719600	-0.36806300
H	-2.07680900	-4.21600800	0.92442200
C	-0.37250900	-3.60670600	-1.85982100
H	-0.17143800	-4.09976000	-2.82228100
H	0.48206000	-2.96875600	-1.59295000
C	-2.02501700	-5.23351900	-1.40526500
H	-1.74151000	-5.99635100	-2.14348600
H	-2.66791400	-5.72014600	-0.65827700
C	-2.70535200	-4.02128300	-2.09117000
H	-2.82444800	-4.18684500	-3.17098300
H	-3.70212900	-3.80258300	-1.68605500
H	-0.01033000	-5.33222400	-0.47282900
H	-1.82974200	-2.02290300	-2.53206100
C	-3.08876000	-1.87731000	0.07558800
O	-3.87722400	-1.28377300	-0.64788600
O	-3.31479800	-2.04385700	1.39576200
C	-4.40144200	-1.30254200	1.93568800
H	-4.50146500	-1.62168000	2.97600500
H	-4.17985500	-0.22525900	1.90363300
H	-5.33316300	-1.49902100	1.39353100
C	-3.76182000	2.65372000	1.57095000
C	-2.35801300	2.78423800	1.70984900
C	-1.81278400	3.38641200	2.85271500
C	-2.67630000	3.83188700	3.83351700
C	-4.07284900	3.69287100	3.68806700
C	-4.63462800	3.10754000	2.56728000
C	-2.83270100	1.74052700	-0.24949700
C	-1.80462800	2.19279400	0.54911200
H	-0.73171600	3.48750000	2.95987000
H	-2.27751000	4.29740600	4.73381300
H	-4.72687800	4.05904600	4.47775000

H	-5.71404700	3.01637900	2.46269900
Br	0.03463100	2.10986200	0.17741900
C	-5.36268400	1.57775100	-0.01942100
H	-5.28596100	0.60714400	-0.51926900
H	-5.97331200	1.44909700	0.87997300
H	-5.85429300	2.30822000	-0.67486200
C	-2.67513900	1.08131500	-1.55321900
O	-1.66075900	0.38211300	-1.78522000
N	-3.60587700	1.28956500	-2.48310500
H	-4.32518400	1.98698300	-2.31742600
C	-3.66097100	0.55493700	-3.73746900
H	-3.05952900	1.06660600	-4.50611400
H	-3.20745300	-0.42957700	-3.56362400
C	-5.09982300	0.40600000	-4.19493800
H	-5.66983800	-0.10530900	-3.40321600
H	-5.55005400	1.40391900	-4.31436600
C	-5.18627100	-0.36810800	-5.49713900
H	-4.76437900	-1.37693300	-5.38598700
H	-6.22549000	-0.47592400	-5.83069200
H	-4.62954200	0.13921400	-6.29753000
N	-4.03621500	2.02510600	0.37727500
K	0.66312600	0.44328300	-3.10268000
I	3.04580300	3.01390100	-2.80849300

DMSO

O	0.00303900	1.49786200	0.38696200
S	0.00025100	0.23667200	-0.44310400
C	1.33816900	-0.81214100	0.18179300
H	1.29993400	-1.79185900	-0.30934100
H	2.28862700	-0.32128200	-0.05429700
H	1.23616300	-0.92105400	1.26897500
C	-1.34108300	-0.80738100	0.18205500
H	-1.30614500	-1.78709400	-0.30945100
H	-1.23925300	-0.91731500	1.26911700
H	-2.29016500	-0.31390900	-0.05412400

2a

C	-2.00185400	-1.28085200	-0.00623600
C	-2.13679000	0.12342300	-0.11734900
C	-3.38509100	0.68118100	-0.42537700
C	-4.46189400	-0.16810500	-0.60343600
C	-4.31515600	-1.56413400	-0.47933400
C	-3.09180800	-2.13982100	-0.17972300
C	0.02067700	-0.38804700	0.37607000
C	-0.84475200	0.65650000	0.14320400
H	-3.49730900	1.76108300	-0.51918400
H	-5.44056000	0.24459100	-0.84314500
H	-5.18260100	-2.20548200	-0.62746200
H	-2.98134100	-3.21967400	-0.09392900
Br	-0.42897100	2.48140000	0.06712900
C	-0.22972900	-2.91453300	0.60901200
H	-0.98572900	-3.41973900	1.22034800
H	-0.05502000	-3.50310900	-0.30077000
H	0.69814100	-2.86230100	1.18270500
C	1.48360100	-0.29806300	0.63374900
O	1.94537300	0.54768700	1.39054900
N	2.24458600	-1.20425800	-0.03314700
H	1.82129100	-1.74645400	-0.77910900
C	3.69453000	-1.18217800	0.04850200
H	3.96510500	-0.91748800	1.07895200
H	4.05909400	-2.20154000	-0.13740900
C	4.33341100	-0.20429900	-0.92554700
H	4.02392800	-0.46286300	-1.94990600
H	3.93974400	0.80261900	-0.72121600
C	5.84687300	-0.21649600	-0.81127200
H	6.25302600	-1.21566200	-1.02460700
H	6.31154900	0.48794400	-1.51248200
H	6.16964000	0.06086000	0.20239200
N	-0.69030800	-1.57584800	0.28705000

TS_5

Pd	0.81024500	0.01346300	-0.40674900
C	1.28558300	2.64025900	-1.65986600
C	0.49452500	2.02616700	-0.66563500
C	-0.50257200	2.78249300	-0.01929700
C	-0.58744300	4.15577600	-0.30042600
C	0.26954300	4.77176400	-1.19752100
C	1.20365700	4.02255700	-1.90913300
H	-1.35988100	4.74389100	0.19741200
H	0.19111200	5.84501800	-1.37529300
C	-1.57205200	2.25883700	0.87444800
C	-1.63521000	2.70724100	2.23370700
C	-2.57589500	1.42488500	0.39366800
C	-0.67343100	3.59030700	2.79155300
C	-2.68436400	2.24307100	3.08173000
C	-3.61404600	0.97296800	1.25093300
C	-0.73930300	3.97805200	4.10778200
H	0.12893800	3.97068200	2.16242200

C	-2.73174200	2.66466500	4.43293000
C	-3.66246900	1.36603700	2.55965300
H	-4.38050600	0.30817000	0.85923800
C	-1.77912700	3.51221600	4.94005700
H	0.01526200	4.65223100	4.51034700
H	-3.54396200	2.29716300	5.06047800
H	-4.46067000	1.01516200	3.21384200
H	-1.82289900	3.82768700	5.98117200
P	-2.55087800	0.72447800	-1.26477800
C	2.08672000	4.68990100	-2.92187600
H	3.15243600	4.51879400	-2.71129200
H	1.90636800	4.31879300	-3.94117600
H	1.91792500	5.77381900	-2.93072800
O	-4.09950000	0.38864900	-1.48694600
O	-2.19040200	1.82799800	-2.36198900
O	-1.64280000	-0.45392400	-1.45474300
C	-4.47795600	-0.43504800	-2.61047800
C	-5.97219300	-0.36191900	-2.74635100
H	-4.14255400	-1.46455000	-2.41767400
H	-3.97197500	-0.07022500	-3.51694800
H	-6.30190300	-0.98848500	-3.58375600
H	-6.46005500	-0.72245600	-1.83155300
H	-6.29923000	0.66858100	-2.93481100
C	-3.01201800	2.99802200	-2.54084100
C	-2.37304600	3.84023800	-3.60937600
H	-3.07940900	3.54103700	-1.58547400
H	-4.02526300	2.68151300	-2.82840700
H	-2.96275500	4.75024000	-3.77302900
H	-1.35543000	4.13317900	-3.31465000
H	-2.31705300	3.28914200	-4.55668500
C	2.14953900	1.77268700	-2.50324300
C	1.56764400	1.49232700	-3.92208800
C	1.94336800	-0.56552800	-3.09951100
C	2.28712900	0.34874600	-1.92607000
H	3.14528800	2.23023500	-2.62100500
C	0.77594200	0.19638000	-3.73267700
H	0.45748100	-0.23753000	-4.69198500
H	-0.09693200	0.28969400	-3.07560000
C	2.72763500	1.00089200	-4.79296800
H	2.41484500	0.91890500	-5.84292500
H	3.59015900	1.68152800	-4.75723700
C	3.03104300	-0.39428000	-4.19033800
H	2.92104000	-1.18669400	-4.94352100
H	4.04635000	-0.47568600	-3.78321600
H	1.00873300	2.34030700	-4.33869900
H	1.77145300	-1.60835600	-2.81200600
C	3.57098900	0.04736000	-1.24186600
O	4.11716400	-1.04191900	-1.22012900
O	4.09371900	1.13030700	-0.63588000
C	5.35256700	0.95017300	0.00392100
H	5.51619400	1.84429600	0.61205100
H	5.35571200	0.05840600	0.63897600
H	6.15081000	0.85779700	-0.74329100
C	3.75748200	-0.25991100	2.69951800
C	2.60674800	0.47413700	2.30759100
C	2.39196300	1.76238600	2.80779600
C	3.32297400	2.30722300	3.67166700
C	4.46996700	1.57656600	4.04284800
C	4.70321800	0.29688400	3.56927400
C	2.61893600	-1.54162800	1.26855900
C	1.91411800	-0.35251400	1.38198300
H	1.49828100	2.31067200	2.51324200
H	3.17203800	3.30902400	4.07123400
H	5.19089200	2.02787500	4.72287500
H	5.59275800	-0.25518800	3.86838200
Br	-0.30838400	-0.63445500	1.93571400
C	4.88377800	-2.39377100	2.02420700
H	5.09427300	-2.67217900	0.98460900
H	5.76901200	-1.88900300	2.42221600
H	4.70825000	-3.29702400	2.62344000
C	2.14769000	-2.57991200	0.38064200
O	1.22461100	-2.27256500	-0.42817000
N	2.63726100	-3.82440000	0.41392900
H	3.24960300	-4.09840700	1.17547000
C	2.17655200	-4.87328500	-0.48311200
H	1.18775800	-5.24409700	-0.16105600
H	2.04959800	-4.43709800	-1.48371400
C	3.17071900	-6.01726900	-0.52032600
H	4.14778000	-5.63142900	-0.84781600
H	3.31087200	-6.40861600	0.49945400
C	2.70031300	-7.12513800	-1.44430200
H	2.57651900	-6.75748300	-2.47256400
H	3.41700100	-7.95486800	-1.47074800
H	1.73250800	-7.53025600	-1.11673800
N	3.75368100	-1.48771700	2.07606000
K	-1.38031700	-2.86398600	-0.42426200
I	-4.61621100	-3.04544600	0.84697800

Int11

Pd	-0.81024500	-0.01346300	-0.40674900
C	-1.28558300	-2.64025900	-1.65986600
C	-0.49452500	-2.02616700	-0.66563500
C	0.50257200	-2.78249300	-0.01929700
C	0.58744300	-4.15577600	-0.30042600
C	-0.26954300	-4.77176400	-1.19752100
C	-1.20365700	-4.02255700	-1.90913300
H	1.35988100	-4.74389100	0.19741200
H	-0.19111100	-5.84501800	-1.37529300
C	1.57205200	-2.25883700	0.87444800
C	1.63521000	-2.70724100	2.23370700
C	2.57589500	-1.42488500	0.39366800
C	0.67343100	-3.59030700	2.79155300
C	2.68436400	-2.24307100	3.08173000
C	3.61404600	-0.97296800	1.25093300
C	0.73930300	-3.97805200	4.10778200
H	-0.12893800	-3.97068200	2.16242200
C	2.73174200	-2.66466500	4.43293000
C	3.66246900	-1.36603700	2.55965300
H	4.38050600	-0.30817000	0.85923800
C	1.77912700	-3.51221600	4.94005700
H	-0.01526200	-4.65223100	4.51034700
H	3.54396200	-2.29716300	5.06047800
H	4.46067000	-1.01516200	3.21384200
H	1.82289900	-3.82768700	5.98117200
P	2.55087800	-0.72447800	-1.26477800
C	-2.08672000	-4.68990100	-2.92187600
H	-3.15243600	-4.51879400	-2.71129200
H	-1.90636800	-4.31879300	-3.94117600
H	-1.91792400	-5.77381900	-2.93072800
O	4.09950000	-0.38864900	-1.48694600
O	2.19040200	-1.82799800	-2.36198900
O	1.64280000	0.45392400	-1.45474300
C	4.47795600	0.43504800	-2.61047800
C	5.97219300	0.36192000	-2.74635100
H	4.14255400	1.46455000	-2.41767400
H	3.97197500	0.07022500	-3.51694800
H	6.30190300	0.98848600	-3.58375600
H	6.46005500	0.72245700	-1.83155300
H	6.29923000	-0.66858000	-2.93481100
C	3.01201800	-2.99802200	-2.54084100
C	2.37304600	-3.84023800	-3.60937600
H	3.07940900	-3.54103700	-1.58547400
H	4.02526300	-2.68151300	-2.82840700
H	2.96275500	-4.75024000	-3.77302900
H	1.35543000	-4.13317900	-3.31465000
H	2.31705300	-3.28914200	-4.55668500
C	-2.14953900	-1.77268700	-2.50324300
C	-1.56764400	-1.49232700	-3.92208800
C	-1.94336800	0.56552800	-3.09951100
C	-2.28712900	-0.34874600	-1.92607000
H	-3.14528800	-2.23023500	-2.62100500
C	-0.77594200	-0.19638000	-3.73267700
H	-0.45748100	0.23753000	-4.69198500
H	0.09693200	-0.28969400	-3.07560000
C	-2.72763500	-1.00089200	-4.79296800
H	-2.41484500	-0.91890500	-5.84292500
H	-3.59015900	-1.68152800	-4.75723700
C	-3.03104300	0.39428000	-4.19033800
H	-2.92104000	1.18669400	-4.94352100
H	-4.04635000	0.47568600	-3.78321600
H	-1.00873300	-2.34030700	-4.33869900
H	-1.77145300	1.60835600	-2.81200600
C	-3.57098900	-0.04736000	-1.24186600
O	-4.11716400	1.04191900	-1.22012900
O	-4.09371900	-1.13030700	-0.63588000
C	-5.35256700	-0.95017300	0.00392100
H	-5.51619400	-1.84429700	0.61205100
H	-5.35571200	-0.05840600	0.63897600
H	-6.15081000	-0.85779800	-0.74329100
C	-4.17774156	4.15878471	2.39492031
C	-3.74273384	2.86111566	2.77366202
C	-4.30724630	2.22836290	3.88580984
C	-5.29963308	2.88036871	4.59288751
C	-5.73550994	4.16210445	4.19995904
C	-5.18851092	4.81459240	3.10848484
C	-2.62481359	3.53148105	0.91788479
C	-2.77442925	2.47735203	1.80673387
H	-3.95540911	1.23973485	4.17713832
H	-5.75046386	2.40297042	5.46172765
H	-6.52076661	4.65497696	4.77118407
H	-5.53438817	5.80594960	2.82019810
Br	-2.00023184	0.72967013	1.85112266
C	-3.84012429	5.70745751	0.45441181
H	-3.92836967	5.40174468	-0.59503356
H	-4.81215790	6.09456435	0.77400701
H	-3.10406281	6.51715556	0.54583841

C	-1.70093211	3.41975189	-0.18776555
O	-1.22461100	2.27256500	-0.42817000
N	-1.33466741	4.47646009	-0.92176564
H	-1.58893099	5.40927860	-0.61324188
C	-0.41352161	4.37179005	-2.04320653
H	0.62171078	4.25576105	-1.67746571
H	-0.66243116	3.46218159	-2.60733176
C	-0.51389997	5.59641974	-2.93127218
H	-1.54937536	5.69370014	-3.29057379
H	-0.30087203	6.49610078	-2.33299156
C	0.44822382	5.50794787	-4.10126266
H	0.23580549	4.62696838	-4.72301140
H	0.37893814	6.39413076	-4.74365843
H	1.48800967	5.42768210	-3.75417753
N	-3.48710779	4.56644791	1.27551976
K	1.38031700	2.86398600	-0.42426200
I	4.61621100	3.04544600	0.84697800

Int12

Pd	-1.08441600	-0.32966500	-0.79241700
C	-2.94844400	-1.16536600	1.28133800
C	-1.70319300	-0.54972300	1.13264900
C	-0.94083600	-0.16089200	2.23751300
C	-1.50592900	-0.32628000	3.50812000
C	-2.77579200	-0.86531300	3.66406500
C	-3.51067200	-1.30758700	2.56464500
H	-0.92917700	-0.02117600	4.38282500
H	-3.20667100	-0.95221100	4.66182700
C	0.46143100	0.33154900	2.17303300
C	0.75341300	1.69893600	2.45151500
C	1.50121700	-0.54572100	1.89356300
C	-0.26984000	2.65728500	2.67247600
C	2.10944300	2.14301500	2.43233500
C	2.84873700	-0.10120200	1.91345100
C	0.03509000	3.98277800	2.86078000
H	-1.31008400	2.33362700	2.67464900
C	2.39374200	3.51501700	2.64274300
C	3.14255500	1.20933700	2.18260400
H	3.65493400	-0.80414700	1.71017800
C	1.37972000	4.41786200	2.84905800
H	-0.76697300	4.70602000	3.00676000
H	3.43623700	3.83578500	2.63232100
H	4.17837400	1.55188500	2.19210200
H	1.60754600	5.47156100	3.00235200
P	1.14938800	-2.17080500	1.21136000
C	-4.89238100	-1.85688800	2.75335700
H	-5.63390800	-1.27037800	2.18966700
H	-4.98672300	-2.89520300	2.40756600
H	-5.17996500	-1.83083100	3.81128600
O	2.58437500	-2.87414100	1.28291700
O	0.17192700	-3.01979500	2.13944900
O	0.61095500	-2.13212600	-0.19212500
C	2.71676700	-4.24403000	0.82387300
C	4.02443100	-4.37723000	0.09552800
H	1.87058400	-4.50147200	0.17225700
H	2.67737600	-4.88983400	1.71039800
H	4.18269500	-5.42360700	-0.19279300
H	4.03830600	-3.76795200	-0.81904200
H	4.86124500	-4.06237500	0.73154100
C	0.49075900	-3.21603600	3.53674200
C	-0.58719300	-4.07658700	4.13034700
H	0.53985900	-2.23339600	4.02879900
H	1.47900100	-3.69077600	3.61507400
H	-0.39367600	-4.23295000	5.19818500
H	-1.56782200	-3.59510700	4.02182500
H	-0.62218000	-5.05582400	3.63734400
C	-3.65416300	-1.66116400	0.06244500
C	-3.62504900	-3.21064300	-0.08012600
C	-2.64022600	-2.51263900	-1.97528600
C	-2.96731000	-1.19943500	-1.24910900
H	-4.70109400	-1.32810300	0.08412300
C	-2.31105500	-3.45738700	-0.81705000
H	-2.20654500	-4.49880900	-1.15281900
H	-1.41624600	-3.17434600	-0.25158400
C	-4.65917300	-3.59087800	-1.14637700
H	-4.82241800	-4.67649000	-1.15786200
H	-5.63157100	-3.11155300	-0.96710300
C	-3.98643400	-3.11519100	-2.45567100
H	-3.76252200	-3.96010100	-3.12049200
H	-4.59978400	-2.41205300	-3.03465500
H	-3.74417700	-3.73024900	0.87782800
H	-1.90431200	-2.39518500	-2.77544300
C	-3.73158800	-0.24764800	-2.14866900
O	-3.34148900	0.18380400	-3.20738200
O	-4.96516300	0.00495000	-1.67979900
C	-5.74321300	0.91244400	-2.45693300
H	-6.67841200	1.05126600	-1.90890400
H	-5.22421800	1.87254100	-2.56357300

H	-5.94624100	0.49770900	-3.45080800
C	-2.60972100	3.60527800	-0.01420600
C	-2.94240700	2.21770100	-0.00977800
C	-4.21242300	1.84160700	0.47190300
C	-5.11349100	2.81407600	0.85517500
C	-4.78246300	4.18133900	0.78109000
C	-3.53463500	4.59178800	0.35589500
C	-0.77996700	2.49140600	-0.62571300
C	-1.76984300	1.53515700	-0.45029200
H	-4.48566100	0.79438800	0.55010500
H	-6.09416000	2.51970800	1.22585000
H	-5.51661000	4.92815600	1.07914600
H	-3.28213200	5.64988900	0.31962100
Br	-0.12099700	-0.39068300	-3.30433500
C	-0.58891200	5.02170400	-0.40093200
H	-0.33460200	5.32598200	-1.42463500
H	-1.22686500	5.79428900	0.03442400
H	0.32046500	4.96318400	0.21066300
C	0.61978200	2.11142900	-0.80430800
O	0.91402800	0.90482800	-0.63575000
N	1.56661400	3.01597600	-1.08377300
H	1.30115900	3.95158900	-1.37004400
C	2.98185300	2.68549000	-1.14349600
H	3.19290800	1.94038400	-0.35963700
H	3.23030900	2.23039300	-2.11879100
C	3.81862400	3.93085400	-0.91639100
H	3.62387300	4.65246500	-1.72510300
H	3.48497200	4.40883900	0.01865600
C	5.29811400	3.60446100	-0.84259200
H	5.64429700	3.10643500	-1.75957300
H	5.89984000	4.51153200	-0.70709700
H	5.51106200	2.93079300	0.00056800
N	-1.29716300	3.75748100	-0.38450300
K	2.57676100	-0.91414800	-1.72990800
I	5.94225100	-0.60330800	-0.80459700

TS₆

Pd	0.82858700	-0.06723400	-1.03396600
C	3.05454300	1.16997600	0.29732900
C	1.74288300	0.79317700	0.64098100
C	1.13260600	1.31741500	1.79851600
C	1.91689000	2.07810000	2.66586500
C	3.24718100	2.35838300	2.38477400
C	3.82602100	1.94510500	1.18944900
H	1.46438800	2.45286000	3.58514100
H	3.84475800	2.92131500	3.10203200
C	-0.30024200	1.15996600	2.16674000
C	-0.67354400	0.24449600	3.19542300
C	-1.26767700	1.96618000	1.58078500
C	0.27960400	-0.53844800	3.89963300
C	-2.05174100	0.10721700	3.54053500
C	-2.63467100	1.81843500	1.93321000
C	-0.11245300	-1.39846000	4.89515700
H	1.33588600	-0.44920000	3.64686900
C	-2.42639400	-0.80329100	4.55971200
C	-3.01535700	0.89932900	2.87456600
H	-3.38706100	2.44553400	1.45907500
C	-1.47882900	-1.53652300	5.22828400
H	0.63524900	-1.98632800	5.42665900
H	-3.48364100	-0.89644000	4.80839700
H	-4.06646000	0.78375200	3.13969800
H	-1.77530800	-2.22645900	6.01669900
P	-0.84660800	3.09803700	0.24105800
C	5.26787700	2.25850300	0.91892100
H	5.83961400	1.35176200	0.67043700
H	5.40466000	2.95374300	0.07988100
H	5.73203000	2.71479000	1.80150400
O	-2.17797900	3.99702200	0.17798800
O	0.33325200	4.08136200	0.67713300
O	-0.52446700	2.45962600	-1.07080300
C	-2.24398600	5.05565400	-0.80653500
C	-3.68586700	5.44306400	-0.97725700
H	-1.81011600	4.69883300	-1.75127400
H	-1.63753600	5.89927900	-0.44823700
H	-3.76589100	6.26090500	-1.70350500
H	-4.28092500	4.59700100	-1.34524500
H	-4.11492200	5.78312800	-0.02665900
C	0.20285100	4.88295700	1.87177400
C	1.41197200	5.76969800	1.96248500
H	0.12714300	4.21425700	2.74157100
H	-0.72393000	5.47093800	1.81185300
H	1.35436300	6.38724900	2.86674500
H	2.33042100	5.17027900	2.00932900
H	1.47393400	6.43516600	1.09244400
C	3.63997700	0.76516200	-1.01923400
C	3.83636300	1.96456800	-2.00280900
C	2.72824700	0.45280800	-3.23509400
C	2.75465700	-0.18899200	-1.84870600

H	4.62147300	0.29769800	-0.84557200
C	2.57909500	1.93010100	-2.87095600
H	2.65831300	2.59301200	-3.74399600
H	1.64952500	2.16112600	-2.33061800
C	4.90606200	1.52716900	-3.00622500
H	5.18159700	2.36155200	-3.66497000
H	5.82241500	1.17700700	-2.51089600
C	4.17542900	0.41470200	-3.79418400
H	4.14390900	0.63358600	-4.86955500
H	4.64706500	-0.57195600	-3.68542200
H	4.04051300	2.91454200	-1.49799100
H	1.98855500	0.01282600	-3.90564100
C	3.10163300	-1.65732600	-1.88845400
O	2.48246700	-2.49792700	-2.50170500
O	4.22300500	-1.93387600	-1.20653800
C	4.58276600	-3.31277400	-1.14009000
H	5.51110300	-3.35460800	-0.56556000
H	3.80016000	-3.88946800	-0.63091100
H	4.74143600	-3.72337800	-2.14304000
C	1.73159200	-2.96487000	2.03947300
C	2.34700000	-1.87324300	1.36835000
C	3.70392600	-1.61519800	1.64303000
C	4.39173300	-2.43543900	2.51633300
C	3.76148700	-3.52461800	3.14808400
C	2.42560400	-3.79668300	2.92631400
C	0.13857900	-1.96268300	0.85591000
C	1.30696700	-1.23317800	0.61371100
H	4.21863100	-0.78266800	1.17292200
H	5.44195200	-2.23428500	2.72164900
H	4.33281400	-4.15601800	3.82652700
H	1.92775100	-4.62949300	3.42019000
Br	-0.48622400	-0.67405600	-3.21200400
C	-0.55911200	-3.81713100	2.45997000
H	-0.54872800	-4.85753300	2.11308500
H	-0.30535300	-3.79518300	3.52606700
H	-1.56336000	-3.40241600	2.33837600
C	-1.15683100	-1.62076900	0.25875200
O	-1.32993400	-0.42787100	-0.08281600
N	-2.12847700	-2.53583500	0.08645600
H	-2.98472300	-2.11580000	-0.28858700
C	-1.93251200	-3.93083600	-0.31328500
H	-0.88132100	-4.20208800	-0.15467400
H	-2.54479200	-4.58675200	0.32260500
C	-2.29415300	-4.11296600	-1.77850700
H	-3.33953300	-3.79757800	-1.93029400
H	-1.67119000	-3.43061000	-2.37927600
C	-2.10943600	-5.55332000	-2.21910400
H	-2.73700600	-6.23444100	-1.62707400
H	-2.37764400	-5.68631500	-3.27447500
H	-1.06521900	-5.87445600	-2.09736000
N	0.39703500	-3.00676600	1.72427800
K	-2.75289100	1.06435100	-1.81447300
I	-5.48553200	-0.81355000	-0.70302200

Int13

Pd	1.10173300	-1.42224100	-0.42245400
C	1.12806000	0.27503000	-1.99213100
C	0.02875400	0.70781400	-1.14432700
C	-1.31151400	0.43031200	-1.54155100
C	-1.56919400	-0.11105200	-2.78424400
C	-0.52719700	-0.43284000	-3.63882400
C	0.80582000	-0.29618100	-3.27829000
H	-2.60094200	-0.27215500	-3.09807800
H	-0.76172300	-0.82916100	-4.62602400
C	-2.50885500	0.64974400	-0.68764100
C	-3.11494600	1.93487900	-0.58052000
C	-3.12815500	-0.45684200	-0.11896300
C	-2.55383500	3.08450300	-1.19030700
C	-4.33358800	2.07313300	0.14457700
C	-4.34903100	-0.30430600	0.59098000
C	-3.14701100	4.31547000	-1.05226100
H	-1.64140700	2.98410100	-1.77600100
C	-4.92718500	3.35267000	0.26273900
C	-4.92896400	0.92834700	0.72315800
H	-4.83159400	-1.17882200	1.02444300
C	-4.34289800	4.45340200	-0.31377500
H	-2.69613100	5.18949400	-1.52009800
H	-5.85733800	3.44428500	0.82402400
H	-5.86615700	1.04258100	1.26803000
H	-4.80417000	5.43446200	-0.21202200
P	-2.49648800	-2.13368300	-0.38045100
C	1.74142900	-0.79871200	-4.35236000
H	2.67578600	-0.25390400	-4.46446300
H	1.99138500	-1.85685000	-4.19598100
H	1.22479400	-0.74639100	-5.31775300
O	-3.16794000	-3.02343600	0.76831600
O	-3.20312300	-2.70335900	-1.69435800
O	-1.02260400	-2.31452700	-0.48678400

C	-2.50559600	-3.17762500	2.04438300
C	-3.36450700	-2.61328000	3.14417700
H	-1.51643500	-2.69868000	2.00231800
H	-2.34343600	-4.25423700	2.17709900
H	-2.88180400	-2.77851600	4.11643700
H	-3.52638400	-1.53381300	3.01566600
H	-4.34617900	-3.10387300	3.16387500
C	-4.62619900	-2.62161000	-1.90413300
C	-4.92216700	-3.21127100	-3.25485500
H	-4.93802200	-1.56759500	-1.85591300
H	-5.13884900	-3.17088900	-1.10198300
H	-5.99937200	-3.16373600	-3.45441600
H	-4.39969000	-2.65490900	-4.04353600
H	-4.60747500	-4.26099300	-3.30173800
C	2.56860400	0.58992300	-1.48361400
C	3.78117300	0.38119900	-2.40419400
C	4.17676600	-1.11807500	-0.77461000
C	2.89181000	-0.39470900	-0.33423700
H	2.57321700	1.62028300	-1.12168600
C	4.04671600	-1.12872300	-2.29246200
H	4.97378300	-1.43776700	-2.79602000
H	3.22672200	-1.76991200	-2.62829300
C	5.01200600	0.95546800	-1.67483600
H	5.84225400	1.08202700	-2.38186100
H	4.80181200	1.94196100	-1.23818800
C	5.33858100	-0.11479600	-0.60902600
H	6.28360100	-0.62734800	-0.83672200
H	5.43719800	0.29226400	0.40552400
H	3.66987000	0.81893100	-3.39958600
H	4.32533000	-2.07817900	-0.27307100
C	3.10692700	0.13282700	1.06112100
O	3.50116000	-0.53706900	1.99151500
O	2.95483900	1.46501400	1.15241100
C	3.32624600	2.04811500	2.39666300
H	3.14496400	3.12174800	2.29119700
H	2.72949100	1.64441900	3.22408600
H	4.38786200	1.86662000	2.60649100
C	0.72696300	3.79398000	0.79075500
C	0.77045300	3.01407400	-0.38321900
C	1.25093100	3.59819300	-1.56591700
C	1.69947800	4.90866100	-1.53571700
C	1.68139900	5.65450700	-0.34274300
C	1.19428300	5.11062500	0.83531700
C	-0.18973300	1.79650700	1.25753100
C	0.22007400	1.71934300	-0.06762600
H	1.26404100	3.03379200	-2.50008000
H	2.07064000	5.37120300	-2.44908600
H	2.04209100	6.68202300	-0.34812500
H	1.15374300	5.69610800	1.75270800
Br	1.97807900	-3.52529600	0.58798400
C	-0.07551500	3.49372500	3.13069700
H	0.75624200	4.13840200	3.43569500
H	-1.01475900	4.05450200	3.21428200
H	-0.11080600	2.64136900	3.81651200
C	-1.15871800	1.02417400	2.11590300
O	-2.24207600	1.54591800	2.36163100
N	-0.81506200	-0.17948700	2.62992800
H	-1.53596500	-0.53566500	3.25406200
C	0.53876000	-0.65875600	2.88902800
H	0.94780100	-1.15712800	1.99880900
H	1.19323200	0.19400400	3.12376600
C	0.53180600	-1.63990600	4.04669000
H	0.06033800	-1.16536000	4.92289000
H	-0.10114200	-2.50222400	3.77808200
C	1.93168200	-2.11762500	4.38471900
H	2.57785000	-1.27727800	4.67466300
H	1.91497000	-2.83456600	5.21592300
H	2.39141500	-2.60203600	3.51268600
N	0.15057900	3.03504100	1.77628900

TS_7

Pd	2.17313500	-1.15701200	-0.72397800
C	0.63440400	-0.16136000	-1.74056100
C	-0.43778000	0.37007500	-0.98851600
C	-1.74681700	0.23714000	-1.50650700
C	-1.94574500	-0.29700400	-2.77460800
C	-0.87585100	-0.77671100	-3.50823100
C	0.42844100	-0.74966600	-3.01162200
H	-2.95773900	-0.36254000	-3.17473500
H	-1.04981400	-1.22187500	-4.48826600
C	-2.96417500	0.58087100	-0.71623000
C	-3.45280800	1.92230900	-0.66725900
C	-3.66750400	-0.42750100	-0.07510500
C	-2.83669100	2.97640400	-1.38674100
C	-4.61404500	2.21954300	0.10548400
C	-4.83624900	-0.12013000	0.66904400
C	-3.31329400	4.26211800	-1.30780500
H	-1.97657400	2.75207800	-2.01479100

C	-5.08151800	3.55472300	0.17175300
C	-5.28605400	1.16783900	0.77230800
H	-5.36899500	-0.92456600	1.17289700
C	-4.44160500	4.55824800	-0.51270400
H	-2.81903100	5.05788300	-1.86383000
H	-5.96551600	3.76545000	0.77422500
H	-6.17559800	1.40076400	1.35801800
H	-4.80862600	5.58176500	-0.45457400
P	-3.16169200	-2.16707900	-0.21130000
C	1.47589600	-1.41223200	-3.86513800
H	2.10086800	-0.68726500	-4.40172100
H	2.14244900	-2.06293400	-3.28592000
H	0.98827500	-2.03804100	-4.62203700
O	-4.10481800	-2.93067300	0.84019000
O	-3.77477800	-2.66364400	-1.61564200
O	-1.71966900	-2.49000900	-0.12946700
C	-3.59432700	-3.49574400	2.06256500
C	-4.41622900	-3.00202500	3.22162800
H	-2.53950200	-3.22162500	2.18078300
H	-3.66058200	-4.58689300	1.95875100
H	-4.09512300	-3.49962500	4.14599000
H	-4.28671600	-1.92044600	3.34880200
H	-5.48173700	-3.21947000	3.07031900
C	-5.14813300	-2.43332100	-1.95983300
C	-5.39800700	-3.05566300	-3.30660900
H	-5.34164800	-1.34953000	-1.98662000
H	-5.79698900	-2.87527900	-1.18948700
H	-6.43912700	-2.89128800	-3.60982200
H	-4.74297900	-2.61178400	-4.06747200
H	-5.21246500	-4.13659200	-3.27888800
C	2.41170400	0.96913300	-1.42261100
C	3.05235600	1.26730500	-2.76528700
C	4.73311000	0.45995200	-1.49627900
C	3.50208200	0.45835700	-0.61594200
H	1.77765000	1.72127500	-0.96577400
C	4.15180600	0.20139200	-2.87994000
H	4.86446700	0.42825900	-3.68378600
H	3.78869200	-0.82319600	-2.99674700
C	3.90887400	2.52645200	-2.46574000
H	4.26596500	2.96177500	-3.40725300
H	3.33032900	3.29922200	-1.94034000
C	5.07810300	1.97082800	-1.61810100
H	6.03739800	2.07315800	-2.14105600
H	5.18444600	2.46955400	-0.64555500
H	2.35885400	1.38790500	-3.60321600
H	5.55235000	-0.17506200	-1.14900600
C	3.75507600	0.82412700	0.81381500
O	4.72312200	0.44149100	1.43340000
O	2.85801700	1.69102600	1.29206100
C	3.11560100	2.16688100	2.61398900
H	2.31535700	2.87712800	2.83715800
H	3.11211100	1.34184200	3.33610400
H	4.08714200	2.67247600	2.65461800
C	-0.12205900	2.96602600	1.65700900
C	-0.03094500	2.55569700	0.30947000
C	0.26821500	3.51611000	-0.67022900
C	0.46894800	4.83166900	-0.28480000
C	0.36870900	5.21642200	1.06526000
C	0.07202700	4.29137400	2.05466000
C	-0.50429000	0.76155100	1.58359000
C	-0.27733900	1.13629400	0.27487000
H	0.33973800	3.23133900	-1.72236600
H	0.70218400	5.58302400	-1.03805900
H	0.52732700	6.25874300	1.33781200
H	-0.00496800	4.58503900	3.10098400
Br	3.56104700	-3.00623600	0.17811800
C	-0.60053900	1.88901200	3.85280400
H	0.32700200	2.18011800	4.36390100
H	-1.38952900	2.60420300	4.11623900
H	-0.90059700	0.90023900	4.21107600
C	-0.95532700	-0.53347600	2.19623700
O	-2.15334800	-0.75429600	2.35397600
N	0.00693700	-1.38291900	2.61345800
H	-0.35667300	-2.20606800	3.08961700
C	1.40729100	-1.05435800	2.85687300
H	1.89081600	-0.77464100	1.90658900
H	1.48095300	-0.18933800	3.53851100
C	2.11807200	-2.24599800	3.46624600
H	1.57189100	-2.56568400	4.36850600
H	2.08461200	-3.07940000	2.74798300
C	3.55884300	-1.91825300	3.80710900
H	3.62012800	-1.09998900	4.54009600
H	4.07249400	-2.78772400	4.23742000
H	4.10835400	-1.61335200	2.90597000
N	-0.41241700	1.86010800	2.41815400

Int14

Pd	1.82028300	-1.36197000	-0.79702100

C	0.45665300	-0.08368900	-1.57445200
C	-0.53150000	0.37218100	-0.69469100
C	-1.58647900	1.16774200	-1.19109400
C	-1.61393000	1.48622700	-2.54861900
C	-0.63217000	1.00873900	-3.41046600
C	0.42000700	0.21571400	-2.94098800
H	-2.42865200	2.10111400	-2.93424500
H	-0.67668200	1.25923600	-4.47175800
C	-2.66154000	1.65414300	-0.28170100
C	-2.57649400	2.97152600	0.26342600
C	-3.73702200	0.84341700	0.06531300
C	-1.59613000	3.90597700	-0.16259100
C	-3.50413000	3.37532700	1.26877400
C	-4.67369100	1.27203300	1.04092400
C	-1.51680000	5.15452300	0.40213100
H	-0.89390700	3.61789400	-0.94455000
C	-3.38157500	4.66139100	1.85081200
C	-4.53461300	2.48793900	1.65574600
H	-5.50421600	0.62184800	1.30363600
C	-2.40714100	5.53191000	1.43181600
H	-0.75795400	5.85715200	0.06051600
H	-4.08649500	4.94578000	2.63221300
H	-5.23634900	2.80199300	2.42833600
H	-2.32409000	6.51939800	1.88276000
P	-4.07006300	-0.64048600	-0.90924600
C	1.49921200	-0.24166200	-3.87734400
H	2.44140700	0.29737000	-3.68744400
H	1.71849800	-1.31213500	-3.75763200
H	1.22149300	-0.06370200	-4.92432500
O	-5.46274700	-1.12074200	-0.26031300
O	-4.47146200	-0.10793100	-2.37474600
O	-3.02534200	-1.68561100	-1.02003400
C	-6.22335900	-2.14467000	-0.93013600
C	-7.63102800	-2.10161300	-0.40370200
H	-5.74949300	-3.11808500	-0.73979700
H	-6.20198700	-1.96493200	-2.01550400
H	-8.23233000	-2.88432600	-0.88201600
H	-7.65197000	-2.26721800	0.68114400
H	-8.09802700	-1.13083200	-0.61484200
C	-5.46208600	0.92172400	-2.53322700
C	-5.82164000	0.99284500	-3.99153400
H	-5.04655300	1.87675500	-2.17658700
H	-6.34321500	0.68719700	-1.91595400
H	-6.56507400	1.78168600	-4.15787600

H	-4.93563500	1.21926200	-4.59844200
H	-6.24422600	0.04031700	-4.33634800
C	-0.03756500	-0.07718000	2.98209200
C	-0.14629200	0.78727500	1.86780800
C	0.12431300	2.15354400	2.04264300
C	0.44628100	2.62020400	3.30447600
C	0.51901600	1.74496900	4.40616700
C	0.29309900	0.38594200	4.25958800
C	-0.43884100	-1.33675100	1.18541600
C	-0.42414500	-0.03323100	0.72459700
H	0.11350100	2.83142100	1.19085000
H	0.65906600	3.67971300	3.44626000
H	0.77122400	2.14160200	5.38875600
H	0.37226400	-0.29557600	5.10562800
Br	3.43256900	-3.15694300	0.09619700
C	0.12989300	-2.51598700	3.36626100
H	1.17142300	-2.41919500	3.70225400
H	-0.52235200	-2.59130400	4.24479900
H	0.03902400	-3.43256600	2.77777500
C	-0.59688700	-2.56609900	0.37548100
O	0.10807200	-2.76609400	-0.63894300
N	-1.47305400	-3.49085800	0.78839000
H	-1.52003100	-4.31096500	0.18707900
C	-2.62287300	-3.26179100	1.65928700
H	-2.37842500	-3.53157300	2.69886700
H	-2.87110200	-2.19087900	1.63899500
C	-3.80845300	-4.07459300	1.17639300
H	-3.97673800	-3.82792400	0.11725300
H	-3.55892400	-5.14626000	1.22409500
C	-5.04998300	-3.78154100	1.99737300
H	-5.33139800	-2.72144300	1.91093200
H	-5.90392500	-4.38534500	1.66371000
H	-4.88562800	-3.99836700	3.06252900
N	-0.23688200	-1.36877400	2.55773100
P	3.33439200	0.30184400	-0.51879200
C	4.73441400	0.26487500	-1.64337900
C	5.12259700	-0.65437000	-2.57466500
O	5.61413900	1.31183900	-1.62508900
C	6.31711300	-0.14909100	-3.16265200
H	4.60883900	-1.58198300	-2.80205000
C	6.56289200	1.03973300	-2.54902100
H	6.91287100	-0.60776800	-3.94237200
H	7.34203100	1.78423800	-2.65855400
C	2.81711000	2.03971600	-0.50912800

C	1.96890300	2.77881300	-1.28626900
O	3.41017100	2.85014000	0.41926300
C	2.03976500	4.11678000	-0.80348900
H	1.36118400	2.41109800	-2.10625900
C	2.91957600	4.09388200	0.23394300
H	1.50401300	4.98060800	-1.17989800
H	3.28811200	4.85271000	0.91331700
C	4.10820500	0.15042000	1.10215000
C	5.39826300	0.00723100	1.51732000
O	3.25985300	0.08830900	2.16356100
C	5.33916900	-0.15448600	2.93331000
H	6.28321700	0.01052300	0.89091200
C	4.02165900	-0.09787400	3.26566200
H	6.17089500	-0.29213900	3.61399100
H	3.48591400	-0.15565700	4.20547100

Int15

Pd	1.23176500	0.08618200	-1.10775900
C	-0.59461400	0.76100800	-1.62367700
C	-1.65081400	0.55919500	-0.72051800
C	-2.97534500	0.80500300	-1.15762600
C	-3.19207100	1.29598900	-2.44449000
C	-2.13071800	1.51667600	-3.31277000
C	-0.81946300	1.24064000	-2.92114600
H	-4.21513000	1.47979100	-2.77473200
H	-2.32204200	1.89807100	-4.31759800
C	-4.15324200	0.49690500	-0.30535300
C	-4.94993000	1.57400600	0.20637400
C	-4.48261900	-0.81049600	0.03682600
C	-4.70343500	2.93339500	-0.12852800
C	-6.00123500	1.29393400	1.12847600
C	-5.52540800	-1.06726100	0.96763200
C	-5.45204600	3.94649500	0.41766500
H	-3.88860600	3.17974900	-0.80879800
C	-6.75794300	2.35976700	1.67466500
C	-6.25439800	-0.04447100	1.50783100
H	-5.74077300	-2.08863200	1.27550400
C	-6.49161100	3.66021000	1.32917700
H	-5.23789500	4.98009700	0.14985100
H	-7.55402500	2.12056300	2.37991900
H	-7.04140200	-0.24670000	2.23389500
H	-7.07574300	4.47298400	1.75751700
P	-3.73548100	-2.26078700	-0.73855100
C	0.32053500	1.50453600	-3.86153100
H	0.84082300	2.43644000	-3.58781300
H	1.07397500	0.70435000	-3.83085700
H	-0.03114200	1.61607800	-4.89606800
O	-3.29814500	-3.11746600	0.54701900
O	-4.92631200	-3.08398800	-1.43203700
O	-2.67215100	-2.05609200	-1.75575600
C	-2.60634900	-4.36514900	0.32033300
C	-2.14545100	-4.88452600	1.65143100
H	-1.75725900	-4.18301100	-0.35510700
H	-3.29014400	-5.06939000	-0.17726000
H	-1.61134400	-5.83304400	1.51796300
H	-1.46084200	-4.16711600	2.12207100
H	-2.99590300	-5.05636600	2.32292500
C	-5.95730000	-3.81300700	-0.74418300
C	-6.36705000	-4.97141800	-1.61258600
H	-6.80201100	-3.13370100	-0.56395600
H	-5.58276500	-4.16236100	0.22871400
H	-7.17657100	-5.53176000	-1.12914000
H	-6.72624400	-4.61979700	-2.58764400
H	-5.52398100	-5.65421900	-1.77851800
C	-1.21203800	0.08291000	2.95354600
C	-1.72851100	0.79656300	1.84734700
C	-2.38030500	2.02106100	2.06789000
C	-2.54939600	2.47714500	3.35953600
C	-2.06322200	1.73098000	4.45308300
C	-1.38482400	0.53991500	4.26873900
C	-0.61959900	-1.01332500	1.09158500
C	-1.36257400	0.08798000	0.65984100
H	-2.72157700	2.61379500	1.22376100
H	-3.05426000	3.42686000	3.53545500
H	-2.21219700	2.10563000	5.46487400
H	-1.00364700	-0.01498900	5.12397800
C	0.25655400	-1.88160300	3.33204800
H	1.29570300	-1.90618500	2.98957900
H	0.22938400	-1.48186100	4.34903900
H	-0.12520300	-2.90687800	3.34994900
C	0.08144300	-2.07429300	0.33640100
O	0.64142200	-3.01726300	0.87335300
N	0.11388000	-1.91064200	-1.06215200
H	-0.81840600	-1.66510400	-1.42897400
C	0.70971900	-3.00270900	-1.83757800
H	1.76643900	-3.08396500	-1.54913800
H	0.21873400	-3.95722900	-1.58156300
C	0.58340400	-2.70924200	-3.31775300

H	-0.48069200	-2.57045000	-3.57162100
H	1.10871900	-1.76402700	-3.51927700
C	1.18714800	-3.82429200	-4.15186300
H	0.69911600	-4.78915700	-3.95206100
H	1.09417600	-3.62042100	-5.22602800
H	2.25764200	-3.94095900	-3.92755200
N	-0.54742300	-1.01935200	2.48328300
P	1.86960800	2.14419400	-0.42561500
C	3.22596400	2.86163000	-1.35511200
C	3.99599900	2.31939700	-2.34601500
O	3.64202100	4.13620400	-1.07510200
C	4.95190900	3.31882200	-2.69223100
H	3.86636100	1.31929400	-2.76426900
C	4.68749000	4.38913500	-1.89492700
H	5.72896200	3.25398500	-3.44450800
H	5.13263400	5.37225700	-1.80149900
C	0.64275900	3.45585600	-0.26294900
C	0.01712000	3.99419900	0.82423300
O	0.01453100	3.84802900	-1.40734800
C	-1.07050900	4.76483900	0.31445700
H	0.28853700	3.84574500	1.86440700
C	-1.02530700	4.63306200	-1.03851800
H	-1.79425000	5.33661900	0.88471300
H	-1.61736900	5.04394600	-1.84755200
C	2.53163400	2.05355300	1.25499700
C	3.54825400	2.70109700	1.90167100
O	1.97642000	1.12289100	2.08423800
C	3.62575800	2.12635900	3.20558400
H	4.16386600	3.49742400	1.49631700
C	2.64707900	1.18051800	3.25680700
H	4.31393300	2.38835300	4.00045500
H	2.31451400	0.49849300	4.03015900
C	3.81701700	-0.88625000	-1.66831100
O	5.05338800	-1.14645400	-1.54569000
O	3.12870800	-0.68296200	-0.54131000
O	3.20236800	-0.77364600	-2.77523800
K	5.21238600	0.16906200	0.79269300
O	6.52926100	-1.67788000	2.11204100
S	6.52746200	-3.13146500	1.65643900
C	6.12625700	-4.11282000	3.11685100
H	6.96807600	-4.04698800	3.81437100
H	5.21846300	-3.70878000	3.58237100
H	5.97535700	-5.15760000	2.81982900
C	4.96406200	-3.38389000	0.79274000
H	4.13457300	-3.12271700	1.46355100
H	4.96128400	-2.72546900	-0.09313900
H	4.88836200	-4.43506400	0.48686200

Int16

K	0.01767800	0.09509200	0.11628400
Br	-0.16426800	-2.98733100	0.00305300
O	-2.08049500	1.59539900	0.51939800
S	-3.49608900	1.80057800	0.00683200
C	-3.60993700	0.89430200	-1.55182600
H	-4.64265400	0.93298600	-1.91864500
H	-2.94979300	1.38091300	-2.27793700
H	-3.30207500	-0.14776600	-1.39528500
C	-4.54345200	0.67795600	0.95640300
H	-5.56102300	0.69635400	0.54806100
H	-4.12644900	-0.33610500	0.90709700
H	-4.55818100	1.02701500	1.99419900
O	2.14062200	1.56064300	-0.16936000
S	3.65346100	1.64631100	-0.06404900
C	4.11280400	0.71033600	1.41017500
H	5.20610000	0.66257300	1.48287200
H	3.71086300	1.23751300	2.28209500
H	3.68747100	-0.29991100	1.35187800
C	4.31212500	0.46472700	-1.25993300
H	5.40077700	0.39926700	-1.14539700
H	3.84888400	-0.51631200	-1.09533200
H	4.07138400	0.83084100	-2.26358200

TS_8

Pd	1.36097100	0.00592600	-1.03144700
C	-0.44715200	0.90367700	-1.35238900
C	-1.59000400	0.71038700	-0.55034500
C	-2.75249900	1.47840200	-0.82352900
C	-2.74246700	2.40322000	-1.86509200
C	-1.64200300	2.51739800	-2.69769900
C	-0.49006700	1.76284100	-2.46599300
H	-3.63705300	2.99850200	-2.05312600
H	-1.66996900	3.19856200	-3.55026900
C	-4.01703500	1.34563700	-0.04486900
C	-4.35637900	2.38605900	0.88284200
C	-4.87334900	0.26075500	-0.17686600
C	-3.57361000	3.56176400	1.02018100
C	-5.49056300	2.23627200	1.73371700
C	-5.99243100	0.12237000	0.68750800

C	-3.89149600	4.52481100	1.94705300
H	-2.69756900	3.69606500	0.38851400
C	-5.79048600	3.24094100	2.68552200
C	-6.28168600	1.06863000	1.63108200
H	-6.63279800	-0.75436100	0.60884700
C	-5.00887900	4.36403100	2.79386700
H	-3.27414000	5.41900400	2.03027800
H	-6.65778800	3.09978400	3.33093100
H	-7.13230900	0.94544200	2.30126600
H	-5.24706600	5.13050700	3.52951900
P	-4.71809000	-0.96395200	-1.49514200
C	0.66205100	1.92441500	-3.41813900
H	1.39763700	2.66080900	-3.05870800
H	1.20160300	0.98081800	-3.57932900
H	0.30830500	2.28316000	-4.39425600
O	-4.47612200	-2.32435600	-0.67522900
O	-6.21202800	-1.11030200	-2.09241100
O	-3.78534100	-0.66052500	-2.59973000
C	-4.19799200	-3.51747100	-1.44064400
C	-4.80823100	-4.69909000	-0.73768100
H	-3.10731100	-3.59595600	-1.53949700
H	-4.60996500	-3.41670800	-2.45667600
H	-4.52885100	-5.62606900	-1.25430700
H	-4.45618500	-4.76641200	0.29950600
H	-5.90483000	-4.63440200	-0.72158800
C	-7.25533600	-1.95140000	-1.58464100
C	-7.74750300	-2.85534800	-2.68490200
H	-8.06072100	-1.29674700	-1.22321600
H	-6.88961000	-2.53963800	-0.73168400
H	-8.58072700	-3.47035200	-2.32152700
H	-8.10106900	-2.26918500	-3.54239900
H	-6.94946900	-3.52593500	-3.03125700
C	-2.28724500	-1.76184300	2.17051300
C	-2.19247100	-0.40630100	1.77147400
C	-2.54115800	0.58655800	2.70032400
C	-3.02065700	0.21224500	3.94556700
C	-3.15122500	-1.14320700	4.29859200
C	-2.77423200	-2.14858000	3.42078600
C	-1.39309300	-1.71732000	0.12596100
C	-1.65880300	-0.39322000	0.43419600
H	-2.41923200	1.64046600	2.45162500
H	-3.29493600	0.98240800	4.66628900
H	-3.53208300	-1.40726200	5.28421600
H	-2.84035700	-3.19905900	3.70163800
C	-1.71587900	-3.98515600	1.19662300
H	-0.91596500	-4.31835900	1.87322800
H	-2.66693700	-4.39797500	1.55406000
H	-1.52698800	-4.37966500	0.19249300
C	-0.56425800	-2.21757800	-1.02861200
O	-1.04525400	-2.58836500	-2.08717600
N	0.80291600	-2.12442800	-0.80363400
H	1.62521300	-2.37032900	-1.64520900
C	1.34340000	-2.45521500	0.53132900
H	2.25446300	-1.86281400	0.68238000
H	0.63918800	-2.15943500	1.32407900
C	1.70231500	-3.92999600	0.64977700
H	0.85102600	-4.55467600	0.33752200
H	2.52035200	-4.14560900	-0.05661800
C	2.11347400	-4.27184300	2.07140900
H	1.26416500	-4.16987000	2.76344100
H	2.49076200	-5.29934400	2.15393200
H	2.90440200	-3.59473500	2.43040300
N	-1.80127400	-2.54347200	1.14755800
P	2.26992100	1.96853300	-0.32022300
C	3.55354200	2.70669600	-1.33307000
C	4.20172100	2.20726900	-2.42849300
O	4.17094200	3.85045200	-0.90784400
C	5.28494900	3.09443300	-2.69244300
H	3.93187500	1.30523900	-2.96999000
C	5.21372200	4.06624100	-1.74053900
H	6.01106500	3.02741800	-3.49374200
H	5.79955000	4.95572300	-1.54354800
C	1.24036600	3.34282900	0.21182800
C	0.80476400	4.49530700	-0.37302900
O	0.59786400	3.14132500	1.39760500
C	-0.16019600	5.04872700	0.52008600
H	1.13671300	4.90092400	-1.32273100
C	-0.24395500	4.18751600	1.56961300
H	-0.71331300	5.97278600	0.39830400
H	-0.81004500	4.18925200	2.49298500
C	3.20468800	1.48726200	1.15598900
C	4.45982700	1.78420200	1.61265600
O	2.66109000	0.49601200	1.91351100
C	4.70287400	0.90895200	2.71258300
H	5.12486600	2.55157800	1.22738400
C	3.57884700	0.15217300	2.84439800
H	5.59487200	0.85580200	3.32570700
H	3.27724000	-0.62204300	3.54029100

C	3.67034300	-1.67613800	-1.93374800
O	4.85909900	-1.79139000	-2.32190800
O	3.38650900	-0.75690600	-1.02581700
O	2.72064500	-2.43804200	-2.37903900
K	5.91549300	0.03858000	-0.69586900
O	7.20531300	-1.37114800	1.10898400
S	6.80613200	-2.79375200	1.47495600
C	6.17347700	-2.72797300	3.16500300
H	7.00527300	-2.46589700	3.82766000
H	5.38588400	-1.96536400	3.22765600
H	5.78037500	-3.71401700	3.44159500
C	5.21005300	-3.08714800	0.68089500
H	4.50455000	-2.29882300	0.98580400
H	5.35131400	-3.05298700	-0.40820400
H	4.84029900	-4.07642000	0.97861900

Int17

Pd	1.45001000	-0.19237500	-0.62083600
C	-0.20913800	0.49602300	-1.58699200
C	-1.39937300	0.49030900	-0.84847100
C	-2.56137500	1.02959000	-1.44752300
C	-2.49412600	1.48702900	-2.76512200
C	-1.31871500	1.40263700	-3.50154600
C	-0.14884200	0.90371500	-2.92211200
H	-3.39052500	1.90885100	-3.22226500
H	-1.30239800	1.74122500	-4.53934000
C	-3.85064900	1.20149500	-0.71789200
C	-4.22243100	2.53620500	-0.33542400
C	-4.69622800	0.15317100	-0.38510500
C	-3.39884200	3.65917800	-0.61007600
C	-5.42542600	2.75994200	0.39612600
C	-5.87948500	0.39358400	0.36513200
C	-3.75506900	4.92349100	-0.20856800
H	-2.45456600	3.50099400	-1.12446100
C	-5.77470800	4.07501400	0.78971100
C	-6.23693600	1.65630800	0.74514500
H	-6.51383700	-0.44119000	0.65678900
C	-4.96136000	5.13910100	0.49163600
H	-3.09724800	5.76455700	-0.42507400
H	-6.70315700	4.21942800	1.34277300
H	-7.14520500	1.82907800	1.32228900
H	-5.23644800	6.14566600	0.80246300
P	-4.40336500	-1.56521500	-0.85611400
C	1.11471900	0.79137600	-3.72575100
H	1.99477600	1.13558200	-3.16120700
H	1.31700400	-0.25629500	-4.00472500
H	1.05539000	1.37459000	-4.65489400
O	-4.35578300	-2.23852700	0.59767100
O	-5.78234800	-2.08523200	-1.51564300
O	-3.29191800	-1.85259100	-1.78612400
C	-3.98029400	-3.62735600	0.71891200
C	-4.28074500	-4.04718000	2.13190300
H	-2.91263400	-3.71845600	0.46985000
H	-4.56038000	-4.22710400	-0.00167900
H	-3.94221000	-5.07678000	2.30319200
H	-3.76403000	-3.39014800	2.84556700
H	-5.35750700	-3.99844700	2.34353900
C	-6.95222200	-2.52632600	-0.81508300
C	-7.50725800	-3.73603800	-1.51985600
H	-7.68329800	-1.70496700	-0.81495700
H	-6.70256800	-2.75844200	0.23033000
H	-8.42867100	-4.06852000	-1.02565600
H	-7.74311900	-3.50307000	-2.56575300
H	-6.78762700	-4.56466000	-1.50427300
C	-1.73581400	-0.64224700	2.69983600
C	-1.85586400	0.38915300	1.73665500
C	-2.27797100	1.65807000	2.16038200
C	-2.61246300	1.85201500	3.49191200
C	-2.51621400	0.80347700	4.42490600
C	-2.06096000	-0.45175600	4.04518900
C	-0.99309300	-1.45251800	0.74531000
C	-1.40822400	-0.15141800	0.48438300
H	-2.31294700	2.49006900	1.45698200
H	-2.93861900	2.83714300	3.82529000
H	-2.78502300	0.98352500	5.46510800
H	-1.95619200	-1.25649300	4.77226700
C	-0.79037700	-2.93757900	2.78831700
H	0.14964700	-2.74335000	3.32718500
H	-1.55346800	-3.23713800	3.51728000
H	-0.64779200	-3.74833500	2.07060600
C	-0.42467700	-2.49386300	-0.17822600
O	-0.85850000	-3.66279300	-0.14325300
N	0.56594200	-2.06777000	-0.96599800
C	1.10476100	-3.00509200	-1.93338300
H	2.09667900	-2.64378900	-2.24743900
H	1.25523600	-3.99122200	-1.45967800
C	0.23897700	-3.16568500	-3.17526600
H	-0.73299400	-3.58538600	-2.87885200

H	0.03391100	-2.16736400	-3.59727900
C	0.91751900	-4.04353800	-4.21173500
H	1.11342700	-5.04998800	-3.81354400
H	0.30960400	-4.15822900	-5.11849800
H	1.88758700	-3.61707200	-4.51182000
N	-1.23361600	-1.75867100	2.07407300
P	2.00565800	2.03206700	-0.23209200
C	3.75101000	2.45598900	-0.38021000
C	4.74683200	1.87544300	-1.11141100
O	4.19096300	3.63346800	0.15506800
C	5.87290400	2.74428500	-1.01714700
H	4.67680200	0.93877100	-1.65586600
C	5.47603500	3.78570300	-0.23754500
H	6.85036700	2.60785200	-1.46375600
H	5.97904700	4.67370900	0.12527800
C	1.33726400	3.34452000	-1.28628100
C	1.93515000	4.04596400	-2.29516800
O	0.01061000	3.65213500	-1.22820000
C	0.91158400	4.83050100	-2.89960900
H	2.98307000	4.00419900	-2.57464200
C	-0.22771300	4.55274600	-2.21269100
H	1.01355900	5.51978500	-3.72902500
H	-1.24523200	4.91751300	-2.29339500
C	1.47341400	2.55354400	1.40787800
C	1.17622100	3.77326600	1.94853100
O	1.31508000	1.57861900	2.35063800
C	0.81312700	3.53424100	3.30306400
H	1.21778100	4.72630200	1.43212500
C	0.91347900	2.18819500	3.48397200
H	0.50819800	4.26444400	4.04306100
H	0.73404500	1.53838400	4.33201800
P	3.35067800	-1.30393300	0.43500900
C	4.28461300	-2.19298300	-0.83105300
C	4.81162300	-3.45001900	-0.89699200
O	4.40177400	-1.57666700	-2.04755200
C	5.28383300	-3.61961300	-2.23112800
H	4.84932300	-4.17065300	-0.08673100
C	5.01032900	-2.45632300	-2.87924200
H	5.76459800	-4.49411200	-2.65261300
H	5.18631000	-2.10705000	-3.88910000
C	4.65222500	-0.40514600	1.30676300
C	6.01518000	-0.47321400	1.29213500
O	4.24405500	0.61541000	2.11486600
C	6.47577700	0.57459000	2.14217300
H	6.61400500	-1.18674000	0.73648400
C	5.36089900	1.19868500	2.60857800
H	7.50269700	0.83070800	2.37363800
H	5.20054100	2.03328600	3.28015100
C	3.03619200	-2.64339000	1.60993200
C	3.53078500	-2.91314800	2.85476400
O	2.17185400	-3.62568900	1.22980300
C	2.93416900	-4.14232200	3.26299800
H	4.24580800	-2.31320800	3.40718200
C	2.12744900	-4.52665300	2.23787200
H	3.09139800	-4.67122600	4.19528900
H	1.48239700	-5.38161100	2.07641400

TS_9

Pd	1.53876700	-0.19954400	-0.53804200
C	-0.27709100	0.02227100	-1.57387100
C	-1.42619300	0.30610200	-0.78867400
C	-2.50275700	0.98556200	-1.38768200
C	-2.40665000	1.40706300	-2.71329000
C	-1.25244000	1.15915100	-3.44807800
C	-0.17053800	0.46627200	-2.91230300
H	-3.24501700	1.93510500	-3.16870000
H	-1.17221000	1.53984000	-4.46871200
C	-3.78880300	1.24198700	-0.68563900
C	-4.11649500	2.59479700	-0.34240600
C	-4.69272900	0.23150700	-0.38714700
C	-3.20300100	3.66259300	-0.54620600
C	-5.35965800	2.88665000	0.28996200
C	-5.91330800	0.53791100	0.27198700
C	-3.52419100	4.94930000	-0.18841800
H	-2.21750100	3.44102200	-0.95115900
C	-5.67154600	4.22543200	0.63255000
C	-6.24344800	1.82529100	0.59438500
H	-6.60708600	-0.26143500	0.52668400
C	-4.77834300	5.23933200	0.39195500
H	-2.80150100	5.74931000	-0.34608700
H	-6.63327300	4.42888400	1.10422600
H	-7.18597000	2.04947000	1.09381400
H	-5.02546300	6.26413200	0.66448300
P	-4.42669000	-1.51073700	-0.79597600
C	1.07427300	0.31689600	-3.74386500
H	1.98278900	0.29936900	-3.12170600
H	1.07647700	-0.60043300	-4.35325600
H	1.16560400	1.16028400	-4.44269500

O	-4.31494100	-2.14817300	0.67403500	H	-0.42824900	-4.19644800	-5.15129600
O	-5.84430900	-2.02394900	-1.36948200	H	1.11384200	-3.34391500	-4.95412200
O	-3.34659600	-1.85144000	-1.74704500	N	-1.18581800	-1.90802600	2.10490200
C	-3.89708000	-3.52541100	0.78594400	P	1.98036200	2.08725600	-0.20354100
C	-4.08656600	-3.93864600	2.21919700	C	3.69910100	2.63610300	-0.14556200
H	-2.84766900	-3.60384400	0.46658000	C	4.81238300	2.08492700	-0.71171000
H	-4.50637800	-4.14937500	0.11185700	O	4.01305600	3.83923800	0.41947500
H	-3.71345100	-4.95915600	2.37223000	C	5.88023300	2.99981500	-0.47646100
H	-3.53524200	-3.26306000	2.88729500	H	4.85601600	1.13817300	-1.24141700
H	-5.14714800	-3.91234800	2.50309800	C	5.33593300	4.03562700	0.21675100
C	-6.92872900	-2.57404700	-0.60830000	H	6.91679900	2.89581700	-0.77400500
C	-7.35016900	-3.88184500	-1.22547800	H	5.74711300	4.94808800	0.63100200
H	-7.75376900	-1.84796000	-0.63067100	C	1.37031600	3.25463100	-1.45274700
H	-6.62576700	-2.70951900	0.43956100	C	2.01576600	3.89903100	-2.47045400
H	-8.21038300	-4.29312800	-0.68243900	O	0.02588400	3.44370400	-1.56702600
H	-7.64066200	-3.74188200	-2.27416400	C	1.00334000	4.52676200	-3.25547100
H	-6.53460500	-4.61589200	-1.18789100	H	3.08790600	3.92565400	-2.63587400
C	-1.77191400	-0.84339800	2.75061100	C	-0.17909700	4.21810600	-2.66081200
C	-1.97417500	0.20355800	1.81386400	H	1.14137000	5.13795700	-4.13933300
C	-2.51106300	1.41511600	2.27359200	H	-1.21058500	4.47510500	-2.87356600
C	-2.87051000	1.54120700	3.60594900	C	1.20936200	2.73511300	1.29585900
C	-2.67937700	0.48268600	4.51126600	C	0.70180400	3.95255800	1.65395400
C	-2.11099200	-0.71401700	4.10046500	O	1.06060000	1.85376500	2.33050700
C	-0.95920300	-1.53524100	0.79476300	C	0.21966000	3.81412200	2.98657900
C	-1.46383200	-0.26587700	0.55355600	H	0.68678900	4.84347800	1.03531200
H	-2.61503500	2.26248700	1.59881800	C	0.46412400	2.52202500	3.33856200
H	-3.28613600	2.48357300	3.96186700	H	-0.25471500	4.57185200	3.59893700
H	-2.96501200	0.60893600	5.55482700	H	0.27326700	1.94790700	4.23761800
H	-1.93506100	-1.52424500	4.80698400	P	3.48583700	-1.31908000	0.43544100
C	-0.59558200	-3.04773200	2.77597100	C	4.55498500	-2.04109000	-0.83508100
H	0.40385700	-2.80355600	3.16801600	C	5.19559300	-3.24069200	-0.95585800
H	-1.23513200	-3.35622400	3.61042100	O	4.72177400	-1.31245300	-1.98118200
H	-0.51555900	-3.87135900	2.06219600	C	5.80112600	-3.24826200	-2.24697000
C	-0.41397800	-2.43594000	-0.22906800	H	5.22798900	-4.02459800	-0.20614000
O	-0.57093600	-3.66547200	-0.19665600	C	5.48429700	-2.05668300	-2.81814200
N	0.25747500	-1.77999200	-1.22244800	H	6.39400800	-4.03848000	-2.69174500
C	0.77615600	-2.61033000	-2.30597800	H	5.71434500	-1.60454600	-3.77486800
H	1.58050700	-2.05963200	-2.81007700	C	4.67481000	-0.43144900	1.46843900
H	1.23694700	-3.50205700	-1.84894200	C	6.03462800	-0.45465600	1.59312500
C	-0.28073800	-3.03078200	-3.31599300	O	4.15570100	0.56533400	2.24386300
H	-1.07752300	-3.57112800	-2.78780900	C	6.37270100	0.59211800	2.49978000
H	-0.74872000	-2.13157600	-3.74632200	H	6.70939000	-1.13937600	1.09034800
C	0.32391700	-3.88761000	-4.41400500	C	5.19580500	1.17434700	2.85635800
H	0.77889200	-4.80012400	-4.00160700	H	7.36185200	0.87703600	2.83804600

H	4.94109200	1.99369600	3.51754700
C	3.18118000	-2.79719300	1.43105300
C	3.66700600	-3.28609500	2.60950200
O	2.26510700	-3.66320800	0.90970300
C	3.01083800	-4.53591200	2.82223900
H	4.41269700	-2.81460500	3.24024700
C	2.17904200	-4.71146800	1.76044400
H	3.14448700	-5.21485000	3.65601500
H	1.49149000	-5.49719100	1.47294900

3l

C	-2.27170500	-0.62735400	0.76259900
C	-1.04604800	0.08101900	0.55139500
C	0.06891300	-0.24703600	1.36151800
C	-0.09234400	-1.12325600	2.43007800
C	-1.28000900	-1.81000500	2.60954300
C	-2.35939600	-1.63829300	1.74872700
H	0.75930100	-1.33553300	3.07642700
H	-1.35094400	-2.56379000	3.39391300
C	1.47484000	0.18187700	1.12140900
C	2.10669600	1.03728300	2.08300900
C	2.22231600	-0.31544500	0.05873600
C	1.39932800	1.59549300	3.18032200
C	3.47503200	1.40218600	1.91241900
C	3.57417300	0.08875200	-0.11186200
C	2.02295100	2.43136000	4.07427100
H	0.34420000	1.36208500	3.30732900
C	4.09403900	2.25828500	2.85532900
C	4.18287600	0.91979300	0.78764300
H	4.13757400	-0.28842800	-0.96170900
C	3.38679500	2.75987300	3.91872500
H	1.45948100	2.84850100	4.90724700
H	5.14345000	2.51528600	2.71013100
H	5.22330800	1.21536000	0.65347200
H	3.86976600	3.42043100	4.63689800
P	1.55208900	-1.43541400	-1.19864900
C	-3.48089100	-2.63175700	1.87625100
H	-4.38035300	-2.23143000	2.36466200
H	-3.78074800	-3.03315900	0.89980100
H	-3.14238200	-3.47877900	2.48537600
O	2.90371100	-2.19408700	-1.64068000
O	0.68272400	-2.57278900	-0.47002000
O	0.78818500	-0.83224300	-2.31544200
C	2.80238400	-3.14520100	-2.71804300
C	4.07854300	-3.93885800	-2.75657600
H	2.63407300	-2.59902200	-3.65588400
H	1.93407300	-3.80033100	-2.54787500
H	4.04260800	-4.66740200	-3.57556600
H	4.94199200	-3.28153800	-2.91849600
H	4.22921400	-4.48428100	-1.81594000
C	1.28336400	-3.46911500	0.47944600
C	0.28178400	-4.54497600	0.79623900
H	1.56281900	-2.90266600	1.38083300
H	2.20155400	-3.89606800	0.05054000
H	0.69996200	-5.24263700	1.53208000
H	-0.63798500	-4.11158900	1.21230600
H	0.02080900	-5.11083100	-0.10724100
C	-0.59646100	2.44352900	-2.29984100
C	-0.12339500	2.05240100	-1.02040000
C	0.95934700	2.76941000	-0.48466700
C	1.56564700	3.76412000	-1.23176400
C	1.11085600	4.08586500	-2.52271400
C	0.01643800	3.43894500	-3.06753700
C	-1.99712400	0.90453300	-1.53844600
C	-1.01109800	1.00867500	-0.56644100
H	1.30734200	2.57841900	0.52449100
H	2.40383700	4.31319700	-0.80466000
H	1.61021100	4.86969500	-3.09036800
H	-0.36672400	3.70539900	-4.05119500
C	-2.47249100	1.91881500	-3.83444600
H	-2.86541200	2.94225300	-3.89577500
H	-1.81196700	1.74312700	-4.69267700
H	-3.30356000	1.21529200	-3.85606300
C	-3.26043400	0.23468400	-1.32465700
O	-4.20752900	0.27983700	-2.11040700
N	-3.38114700	-0.35791500	-0.06832500
C	-4.76371000	-0.40719000	0.44709700
H	-5.38660700	-0.00685500	-0.35784200
H	-5.09988700	-1.43592300	0.60358200
C	-4.95196300	0.43007500	1.70125800
H	-4.28158000	0.09022200	2.50424300
H	-4.66821900	1.47011900	1.47854800
C	-6.39417000	0.35977700	2.17134500
H	-6.67971400	-0.67350100	2.41733600
H	-6.55477700	0.97093800	3.06805900
H	-7.08496700	0.71687600	1.39450300
N	-1.73004000	1.73540900	-2.60284000

1d

C	-1.83187100	0.35084200	0.18558400
C	-0.50424900	0.15715700	-0.17799500
C	0.49672300	0.99400000	0.32620700
C	0.11998100	2.02098900	1.19495100
C	-1.21082500	2.19968200	1.54550600
C	-2.22810700	1.37261800	1.05435000
H	0.88094800	2.68668800	1.60022300
H	-1.48373400	3.00592400	2.22695300
H	-0.23552500	-0.64538300	-0.86429600
C	1.91363500	0.79612000	-0.06715400
C	2.61349500	-0.41136300	0.24158200
C	2.57901700	1.79460900	-0.76264700
C	2.02726300	-1.46604500	0.99051300
C	3.96642400	-0.57509400	-0.18777500
C	3.91985200	1.63058600	-1.18081500
C	2.72840300	-2.61394900	1.27209200
H	1.00710600	-1.35764100	1.35473400
C	4.66077300	-1.77167500	0.11410200
C	4.59271700	0.47196600	-0.90406600
H	4.40419700	2.43623500	-1.73411900
C	4.05748700	-2.77798600	0.82609900
H	2.25338200	-3.40490500	1.85096400
H	5.69062800	-1.87130900	-0.23083900
H	5.62435800	0.34270400	-1.23133200
H	4.59912200	-3.69438100	1.05451000
I	-3.28210800	-0.98427400	-0.64677700
C	-3.64833600	1.60504500	1.46034700
H	-4.28858600	1.80561900	0.58972300
H	-4.07220100	0.72824900	1.97008100
H	-3.72294600	2.46136100	2.14055900
O	1.91005900	2.93541000	-1.05942200
H	2.50023800	3.52508000	-1.55787000

1b’

C	2.96969900	0.42845300	0.19625200
C	1.76161200	0.84456700	-0.34553800
C	0.80290100	1.48791600	0.44896200
C	1.11988200	1.69215400	1.79659000
C	2.33528900	1.26976600	2.32287700
C	3.29730900	0.61613200	1.54388600
H	0.39276000	2.17639600	2.44936000
H	2.55163400	1.43613600	3.37907400
H	1.54201500	0.66466400	-1.39620600
C	-0.50565300	1.87193900	-0.12492800
C	-0.98343600	3.21245800	-0.05561600
C	-1.28277100	0.84563400	-0.73134300
C	-0.21806900	4.28820100	0.47950300
C	-2.28142100	3.53987600	-0.56838500
C	-2.58231800	1.22983300	-1.23564100
C	-0.71000900	5.57204800	0.53000300
H	0.78754800	4.08983500	0.84844500
C	-2.76068400	4.86643400	-0.49774900
C	-3.05908000	2.50523800	-1.15226400
H	-3.18755600	0.43547300	-1.68229700
C	-2.00008800	5.87569800	0.04645100
H	-0.08952200	6.36628300	0.94538100
H	-3.75685300	5.07383800	-0.89369000
H	-4.05081600	2.75382700	-1.53666000
H	-2.38010100	6.89500800	0.09552900
I	4.34376500	-0.55740800	-1.12239600
C	4.58002000	0.15003600	2.15593000
H	4.69696600	-0.94011500	2.06676200
H	5.45398300	0.60289000	1.66646200
H	4.61847100	0.40684000	3.22117300
O	-0.91158700	-0.37980800	-0.80764600
K	-2.00881900	-2.59417100	-1.61094500
O	-4.50948100	-1.84186600	-1.20871200
S	-4.91202100	-1.16723900	0.09630700
C	-3.48923000	-1.34060000	1.19547700
H	-3.23937600	-2.40372000	1.31951500
H	-2.64779400	-0.82074800	0.71727700
H	-3.71286200	-0.88062100	2.16573700
C	-5.98192200	-2.33870000	0.95822600
H	-5.48748200	-3.31745400	0.99329900
H	-6.17921300	-1.96988700	1.97199800
H	-6.92369900	-2.41275000	0.40423400
O	-1.10417800	-3.71600500	0.59299000
S	0.16793300	-3.35605600	1.34921400
C	-0.11399700	-1.74315700	2.11715500
H	-0.44627800	-1.04740500	1.33228300
H	-0.87541000	-1.86078100	2.89641200
H	0.82616900	-1.40307300	2.57132000
C	1.34181200	-2.74727200	0.11545500
H	0.88783000	-1.89671700	-0.41518100
H	2.25925600	-2.43085500	0.62984000
H	1.57662300	-3.57097300	-0.56831500

Int18

Pd	0.89717500	-0.79545100	-0.42691400	C	4.40707500	2.45541500	-1.40515800
P	-0.19486400	-1.92738200	1.30136400	O	2.32258600	2.77955400	-0.68073700
P	2.94180800	0.25746600	-0.03714600	C	3.98587200	3.76925600	-1.77949700
C	0.65296000	-3.27353500	2.16864500	H	5.36624700	1.99391500	-1.60820900
C	0.22524600	-4.32522500	2.92820500	C	2.71961800	3.91680900	-1.31053700
O	2.01514900	-3.31104800	2.05816100	H	4.56072000	4.50912100	-2.32415000
C	1.39098800	-5.04783400	3.31419400	H	1.99084800	4.72053400	-1.30372600
H	-0.80645700	-4.54594100	3.18183500	C	-0.26468400	-0.11930300	-2.53447200
C	2.44133500	-4.38478600	2.75983100	C	-1.45763300	-0.23610400	-1.81075200
H	1.44119800	-5.94399900	3.92112700	C	-2.21984900	0.90152300	-1.47489900
H	3.51175400	-4.54916800	2.76919100	C	-1.73720300	2.13716200	-1.92148200
C	-1.83993400	-2.65765800	1.05566300	C	-0.53807500	2.24184000	-2.62378000
C	-3.02711900	-2.57159900	1.72549900	C	0.23595500	1.12662000	-2.95696700
O	-2.01181200	-3.37108000	-0.09845900	H	-2.30709500	3.03922600	-1.70447600
C	-3.98374500	-3.28847000	0.94600200	H	-0.18515900	3.22709800	-2.93433700
H	-3.19596100	-2.05669000	2.66625800	H	-1.81921200	-1.22376100	-1.52664500
C	-3.31454300	-3.74646100	-0.14420100	C	-3.43777500	0.78515300	-0.64268600
H	-5.03443500	-3.43696000	1.16627100	C	-4.46581800	-0.15297800	-0.96459000
H	-3.60700200	-4.33432700	-1.00568700	C	-3.53835700	1.61013000	0.52161800
C	-0.62020300	-0.84841900	2.69694100	C	-4.48224000	-0.91672800	-2.16814900
C	-0.59085100	-0.93257600	4.05815100	C	-5.58375300	-0.32780800	-0.08531300
O	-1.19606600	0.33335300	2.32309700	C	-4.67246000	1.36717900	1.38590600
C	-1.19133500	0.26746100	4.55007900	C	-5.49664300	-1.80254300	-2.45512300
H	-0.20120600	-1.76059300	4.63998100	H	-3.68265700	-0.78105500	-2.89529000
C	-1.54739900	0.99340200	3.45671600	C	-6.60719100	-1.24722300	-0.40352400
H	-1.34270400	0.54729000	5.58612300	C	-5.63787900	0.44782800	1.10381600
H	-2.05356400	1.94050400	3.29804300	H	-4.72234700	1.97110900	2.29394400
C	3.07308800	0.56447900	1.75649700	C	-6.57288200	-1.98844500	-1.56329100
C	3.86405500	0.09615900	2.76229000	H	-5.46930700	-2.35975000	-3.39172200
O	2.05626400	1.31894100	2.28880400	H	-7.43728300	-1.35313100	0.29803200
C	3.31172200	0.59337800	3.98389700	H	-6.47971700	0.29861500	1.78357900
H	4.74048800	-0.53196600	2.64950700	H	-7.36892600	-2.69325300	-1.79859300
C	2.22668000	1.33054600	3.64015900	I	0.62437500	-1.94728700	-3.26770600
H	3.68476600	0.42699500	4.98751900	C	1.51473500	1.28618400	-3.71381800
H	1.50791800	1.91439500	4.20236700	H	2.35808400	0.81513000	-3.18480100
C	4.44782100	-0.67107800	-0.35076100	H	1.46101500	0.81849100	-4.70785400
C	4.59299400	-1.91274500	-0.90061500	H	1.75230700	2.34802300	-3.85504400
O	5.67796900	-0.16739000	-0.01898800	O	-2.68627100	2.50741600	0.84456300
C	5.98950300	-2.19322500	-0.91123600	K	-0.08813000	2.28297000	0.65448200
H	3.78349200	-2.54711800	-1.24831100	O	0.06908200	4.86277700	0.09280700
C	6.59176100	-1.10232900	-0.36733400	S	-0.91568300	5.87992700	0.65820100
H	6.48129900	-3.08686100	-1.27561200	C	-1.50907000	5.20322900	2.22972400
H	7.62602600	-0.84800100	-0.16970500	H	-2.01190400	4.24090400	2.04786600
C	3.36350200	1.88868500	-0.73389000	H	-0.64457000	5.08914800	2.89440300

H	-2.21322400	5.91669900	2.67644700
C	-2.46688700	5.63582500	-0.23484800
H	-2.77478500	4.58706500	-0.11063700
H	-3.22431300	6.31686200	0.17306200
H	-2.28851600	5.87455600	-1.28934900

TS_{10}

Pd	1.03046500	-0.76833500	-0.74301600
P	0.59662700	-2.08718300	1.28877900
P	2.73964200	0.86254900	-0.17684800
C	1.66752900	-3.19749800	2.22508700
C	1.44126500	-4.24626500	3.07039600
O	3.01308000	-3.03065800	2.04529300
C	2.72076600	-4.74692700	3.44341300
H	0.46734600	-4.60674500	3.38541800
C	3.63016800	-3.97055300	2.79384300
H	2.93727100	-5.57723200	4.10482500
H	4.71263200	-3.96604400	2.76098800
C	-0.86567800	-3.11814500	0.98202600
C	-2.17141700	-3.01187300	1.37316300
O	-0.75110400	-4.05582100	-0.00161900
C	-2.90319200	-3.94846100	0.58317600
H	-2.57132000	-2.33594500	2.12297300
C	-1.99311600	-4.55044100	-0.22699500
H	-3.96996400	-4.14199100	0.61329900
H	-2.06240900	-5.32862500	-0.97700400
C	-0.11371200	-1.05761900	2.60623200
C	-0.12905900	-1.11045300	3.96878900
O	-0.93540900	-0.04916600	2.17726600
C	-1.01534500	-0.07971400	4.40578600
H	0.41729700	-1.81377300	4.58730200
C	-1.48375800	0.52574600	3.28279200
H	-1.27431300	0.17118000	5.42768700
H	-2.19216400	1.32445700	3.07459900
C	2.86878800	1.11543000	1.61904400
C	3.69313000	0.65447900	2.60088700
O	1.81748100	1.79483000	2.18042500
C	3.12626700	1.08236900	3.84134600
H	4.60278200	0.08236700	2.45859800
C	1.99869100	1.76899000	3.53104500
H	3.51808500	0.90538400	4.83585400
H	1.25230900	2.29060200	4.11755500
C	4.34969800	0.17817300	-0.57016500
C	4.66876300	-0.99020000	-1.20168700
O	5.49476100	0.83845100	-0.21106100
C	6.09067500	-1.05636900	-1.23687500
H	3.95789800	-1.71433200	-1.58797200
C	6.53141800	0.07517800	-0.62483300
H	6.70455000	-1.84110100	-1.66164300
H	7.51908300	0.46753700	-0.41543700
C	2.86578600	2.55753200	-0.81762700
C	3.78401700	3.29316000	-1.50724500
O	1.70593500	3.27578800	-0.69150700
C	3.15396500	4.53758700	-1.81691300
H	4.79158000	2.98663800	-1.76324300
C	1.90051800	4.47724300	-1.29614400
H	3.58787100	5.37264700	-2.35408900
H	1.06164500	5.16134900	-1.23025100
C	-0.34987500	0.09610300	-2.06414900
C	-1.53323600	-0.23229000	-1.40577300
C	-2.57261400	0.70578600	-1.28713500
C	-2.40160400	1.92733900	-1.94606800
C	-1.23771400	2.20449100	-2.66692700
C	-0.17854000	1.29268600	-2.76936700
H	-3.19315400	2.67471500	-1.89575700
H	-1.13201500	3.17214200	-3.16255100
H	-1.65503400	-1.21224100	-0.94090000
C	-3.73363600	0.38721700	-0.42351900
C	-4.55756400	-0.74020600	-0.69945200
C	-3.89255700	1.14687500	0.77067800
C	-4.42589900	-1.53341800	-1.87627500
C	-5.58502500	-1.12049200	0.22401200
C	-4.94743500	0.72423000	1.66382600
C	-5.23455900	-2.62245300	-2.10793100
H	-3.66987800	-1.26605300	-2.61493800
C	-6.40033100	-2.24256500	-0.04291600
C	-5.74680600	-0.35108600	1.40695200
H	-5.06852700	1.30455700	2.58057900
C	-6.23552100	-2.99333900	-1.18522800
H	-5.10182900	-3.20237800	-3.02133100
H	-7.17273700	-2.50112000	0.68413800
H	-6.52595800	-0.64440200	2.11389200
H	-6.87077600	-3.85593200	-1.38078800
I	0.63053700	-2.05251800	-3.15247300
C	1.04557000	1.60217100	-3.56758800
H	1.95747400	1.25727300	-3.05871900
H	1.01526100	1.09491900	-4.54385400
H	1.13544300	2.67975900	-3.75644000

O	-3.13892900	2.13400200	1.09280000
K	-0.56405700	2.30875600	0.63321600
O	-0.70670600	4.93040400	0.35869000
S	-1.91870700	5.77873100	0.72953400
C	-2.54255700	5.12376900	2.29584500
H	-2.80603700	4.06178100	2.17274700
H	-1.75829300	5.25712600	3.04944000
H	-3.42819400	5.70101600	2.59038300
C	-3.28490000	5.16831100	-0.28152600
H	-3.39873400	4.09093800	-0.08962500
H	-4.20023500	5.71190500	-0.01554100
H	-3.03551600	5.36029000	-1.33135200

Int19

Pd	1.12632800	-0.01200100	-1.02790700
P	2.53137800	-1.35852000	0.46808400
P	1.29067100	1.97782500	0.13759400
C	1.58364400	-2.84859000	0.83149900
C	0.25693400	-3.11537700	0.64011400
O	2.16941900	-3.88315000	1.50587500
C	0.00972800	-4.38728100	1.23146100
H	-0.45597600	-2.47668400	0.12618300
C	1.20384300	-4.80163900	1.73436300
H	-0.92971200	-4.92641700	1.26471400
H	1.51907800	-5.69950200	2.25204200
C	2.84117400	-0.80003600	2.16423900
C	2.32210000	-1.17789500	3.36913600
O	3.62019100	0.30836600	2.32059000
C	2.81432000	-0.24305600	4.32762300
H	1.65730800	-2.01607100	3.55167700
C	3.59021700	0.63285600	3.63646300
H	2.61797500	-0.22977800	5.39308300
H	4.18542000	1.49010700	3.92661900
C	4.15533600	-1.88569100	-0.09885600
C	4.76168100	-1.71748700	-1.31225500
O	5.00058900	-2.53968500	0.75355600
C	6.05138300	-2.31012200	-1.20498100
H	4.32673500	-1.22795400	-2.17755000
C	6.13627400	-2.78750900	0.06603100
H	6.81611700	-2.37005300	-1.96980700
H	6.91534100	-3.30569200	0.61180700
C	0.55527800	1.84668900	1.76836400
C	-0.16330900	0.82545600	2.32569100
O	0.54084400	2.93288800	2.59865000
C	-0.63600200	1.30292400	3.58176600
H	-0.32976400	-0.15114700	1.87880700
C	-0.17727400	2.58034500	3.69031100
H	-1.25918500	0.77363000	4.29165900
H	-0.27384100	3.33952500	4.45703300
C	3.01815000	2.41938900	0.31007600
C	4.09219300	2.07022900	-0.45701700
O	3.40817400	3.24026500	1.32513300
C	5.22110700	2.70947900	0.12914800
H	4.07015000	1.42355800	-1.32864700
C	4.74678400	3.39313700	1.20557300
H	6.25117000	2.66266400	-0.20242900
H	5.21839500	4.01806100	1.95419800
C	0.57603500	3.51869100	-0.47245100
C	1.10643800	4.55979800	-1.17355000
O	-0.77543800	3.68677800	-0.33845300
C	0.01764500	5.42140100	-1.50068200
H	2.15122300	4.68735300	-1.43620600
C	-1.09447300	4.84548800	-0.97685100
H	0.06061200	6.35207300	-2.05314700
H	-2.14102000	5.12531000	-0.95917800
C	-0.48781100	0.87430600	-1.91645600
C	-1.66824900	0.43743400	-1.32477600
C	-2.92920900	0.86053700	-1.77690700
C	-2.94062800	1.81238900	-2.80627000
C	-1.75600000	2.25654300	-3.38429800
C	-0.49992600	1.78013500	-2.97635600
H	-3.89605800	2.19740200	-3.16131300
H	-1.79748500	2.98393300	-4.19854700
H	-1.62662600	-0.27660500	-0.49403500
C	-4.16264900	0.32425100	-1.15742800
C	-4.37725700	-1.08483600	-1.08658800
C	-5.08409400	1.24679400	-0.56745400
C	-3.49650300	-2.04745700	-1.66372400
C	-5.53220200	-1.60175300	-0.41310900
C	-6.26219000	0.66577100	0.04865400
C	-3.70393400	-3.40154300	-1.52155300
H	-2.63355400	-1.70489000	-2.23405900
C	-5.71933700	-2.99472100	-0.28555300
C	-6.46683000	-0.67819000	0.13252600
H	-6.97572400	1.36846000	0.48343700
C	-4.81882300	-3.89465100	-0.81292800
H	-2.99650700	-4.09838300	-1.97315000
H	-6.60526200	-3.34601800	0.24789600

H	-7.35216800	-1.07552300	0.63479800
H	-4.97217200	-4.96701700	-0.70143500
I	1.03474000	-1.94270700	-3.01379300
C	0.75011500	2.20302300	-3.68818000
H	1.61969700	2.21008900	-3.01468200
H	0.99098700	1.50796400	-4.50797000
H	0.64626500	3.20312300	-4.13079200
O	-4.88418800	2.50255600	-0.50819100
K	-2.78438100	2.26918100	1.07846200
O	-3.57243100	0.43798400	2.76389600
S	-4.07314900	-0.99414800	2.86549700
C	-3.69721200	-1.52468000	4.55102800
H	-2.63639500	-1.34291800	4.76539300
H	-4.32373300	-0.94244700	5.23526500
H	-3.93272300	-2.59049800	4.65661400
C	-2.82313300	-2.01992200	2.05916700
H	-1.84264400	-1.81156100	2.51006700
H	-3.08638700	-3.07810200	2.18247500
H	-2.82624300	-1.76389400	0.99150800

Int20

Pd	-1.73468800	-0.42078800	-0.41313500
P	-2.25324500	1.30402000	1.06891500
C	-1.26221200	1.69722400	2.52673200
C	-1.39952200	1.36678700	3.84499400
O	-0.11698200	2.40863300	2.34182500
C	-0.25993500	1.90579500	4.51021900
H	-2.21709800	0.80685600	4.28540000
C	0.47866600	2.52474900	3.55020200
H	-0.02879800	1.84950900	5.56706000
H	1.40684600	3.08407400	3.56342200
C	-3.86141200	1.02807200	1.83367600
C	-4.41273200	-0.10399300	2.35808300
O	-4.73116900	2.06956200	1.93749900
C	-5.71011200	0.26503800	2.81769600
H	-3.95634500	-1.08944400	2.38378600
C	-5.84889300	1.58640600	2.52918700
H	-6.44612400	-0.37336500	3.29105000
H	-6.64912700	2.30103300	2.67548800
C	-2.28680200	2.86567800	0.18935400
C	-2.06803900	3.14544800	-1.13051700
O	-2.42337500	4.02878700	0.89459300
C	-2.08525900	4.56267900	-1.24909700
H	-1.91332800	2.41587200	-1.91866900
C	-2.29925400	5.03866200	0.00774000
H	-1.94639000	5.15003300	-2.14838800
H	-2.39652300	6.03783400	0.41487700
C	0.15135800	-0.05821800	0.33695600
C	0.92107000	0.85723800	-0.37351700
C	2.20056300	1.23436400	0.06497000
C	2.66905000	0.66079800	1.25178800
C	1.90223300	-0.27059700	1.95078900
C	0.62604200	-0.65359600	1.51391100
H	3.66163000	0.93010300	1.61771400
H	2.29542300	-0.71046100	2.87043000
H	0.53339900	1.30506200	-1.29274200
C	3.03875000	2.16417700	-0.73301400
C	2.78200600	3.55949800	-0.74255100
C	4.07643400	1.58965500	-1.52026300
C	1.70112900	4.15526300	-0.02853400
C	3.62780000	4.43706300	-1.49782500
C	4.92627400	2.52082800	-2.23050500
C	1.47561700	5.51222000	-0.06812400
H	1.03856100	3.51581500	0.55566100
C	3.37151700	5.82586100	-1.51168300
C	4.71033300	3.86899800	-2.22197200
H	5.74653400	2.09213800	-2.81042800
C	2.31525400	6.36779100	-0.81423600
H	0.63328000	5.93077500	0.48476000
H	4.03359600	6.46538300	-2.09924900
H	5.36366100	4.53795200	-2.78688300
H	2.12588800	7.44002500	-0.83654700
I	-4.35984900	-0.25870800	-1.50476600
C	-0.16982200	-1.66628500	2.28242500
H	-1.21195100	-1.34543900	2.42762900
H	-0.22260000	-2.62513300	1.74329500
H	0.27225900	-1.85220800	3.27042400
C	-0.29596900	-2.06408500	-1.40770200
C	0.41653900	-1.58122800	-2.64753500
C	-1.65514600	-2.16280300	-3.29133800
C	-1.57681500	-2.39331000	-1.79904300
H	0.20362100	-2.50653900	-0.54420200
C	-0.72730200	-0.95744400	-3.45123300
H	-0.45281800	-0.76585500	-4.49650200
H	-1.12516000	-0.03723900	-2.99744200
C	0.66103000	-2.88937100	-3.45468000
H	1.31238200	-2.67810000	-4.31185500
H	1.14629700	-3.66740700	-2.84857800

C	-0.76753000	-3.28252100	-3.90420500
H	-0.85988200	-3.26354300	-4.99733100
H	-1.06822900	-4.28070400	-3.56293000
H	1.30139500	-0.95038600	-2.50002100
H	-2.66334200	-2.09454400	-3.70817800
C	-2.46243500	-3.34841500	-1.09766500
O	-3.30622100	-4.02014800	-1.65016700
O	-2.20749700	-3.39465700	0.21707100
C	-3.08271200	-4.20045100	1.00240700
H	-2.76223000	-4.06651900	2.03890200
H	-4.12070700	-3.86526300	0.88547400
H	-3.00885000	-5.25621700	0.71936900
O	4.26244400	0.32842300	-1.62470900
K	3.07996100	-1.75799900	-0.62389700
O	1.88341000	-3.85980400	0.37995200
S	2.16542900	-4.23174800	1.83353700
C	3.71072800	-5.16119900	1.81800500
H	3.65541500	-5.93243100	1.03914600
H	4.52271500	-4.45665900	1.60049800
H	3.87436100	-5.61566200	2.80347300
C	1.06924100	-5.61630600	2.19351100
H	1.22159700	-6.40581800	1.44671800
H	1.26990700	-5.99301800	3.20427000
H	0.04027000	-5.24107500	2.13322300
O	5.36754800	-1.93070000	0.67732600
S	6.14797300	-0.63160400	0.83895400
C	7.09563900	-0.40373900	-0.67685100
H	7.68278100	-1.30797700	-0.88336600
H	6.34681000	-0.21934500	-1.45848200
H	7.75261600	0.46790300	-0.56344700
C	7.54779300	-1.04066000	1.90492500
H	8.06474400	-1.91940000	1.49941200
H	8.22891700	-0.18236600	1.95497500
H	7.16299100	-1.26234800	2.90605600

TS_{11}

Pd	-1.44243300	-0.59220700	-0.53619300
P	-2.67956200	1.16420800	0.51058800
C	-2.19939900	1.89845800	2.09476100
C	-2.63132300	1.68996400	3.37422100
O	-1.19449100	2.81537000	2.08667600
C	-1.83604200	2.53335700	4.20449800
H	-3.42819400	1.02029100	3.68210800
C	-0.98219700	3.18588200	3.37076800
H	-1.89754400	2.64335300	5.28056900
H	-0.20593100	3.92617500	3.52361000
C	-4.35615200	0.66886300	0.94085300
C	-4.87410100	-0.57193400	1.18088000
O	-5.27194800	1.62634100	1.27413500
C	-6.19003800	-0.36827500	1.68575600
H	-4.36839400	-1.51724000	1.01076500
C	-6.37316500	0.97931600	1.71492100
H	-6.90759600	-1.12423400	1.98041500
H	-7.20760700	1.60812900	2.00049600
C	-2.77046400	2.59956300	-0.56562900
C	-2.15701400	2.82987400	-1.76498100
O	-3.45846000	3.71298500	-0.17386800
C	-2.49876700	4.15935400	-2.14282500
H	-1.53364400	2.12423800	-2.30541700
C	-3.28462700	4.63985400	-1.14091400
H	-2.19741500	4.68963800	-3.03812400
H	-3.78405300	5.58784300	-0.98184000
C	0.23385200	-0.11384800	0.72372800
C	1.05454000	0.93334200	0.28968900
C	1.82220300	1.69774100	1.17767500
C	1.70491500	1.40979000	2.54141200
C	0.86523400	0.39361600	2.98678800
C	0.11775600	-0.39194500	2.10222800
H	2.29389300	1.98137100	3.25965200
H	0.77719900	0.19766900	4.05728000
H	1.14827300	1.14379500	-0.77732900
C	2.80900300	2.69010300	0.69007000
C	2.41304800	3.88783400	0.04027700
C	4.18386100	2.34199100	0.85518000
C	1.04951700	4.26508300	-0.13558800
C	3.40793800	4.79307800	-0.45682200
C	5.14539400	3.30258500	0.35306000
C	0.70473200	5.44039200	-0.76418200
H	0.26592000	3.61218000	0.25162600
C	3.01948500	5.98820100	-1.09918100
C	4.77699500	4.45627800	-0.27495500
H	6.19942900	3.04810100	0.48116100
C	1.69216200	6.31852300	-1.25967500
H	-0.34998200	5.69746500	-0.87386100
H	3.80412800	6.65310200	-1.46649200
H	5.53369300	5.14817200	-0.65237500
H	1.40534700	7.24459700	-1.75586900
I	-3.44235700	-0.98864800	-2.39735000

C	-0.76196300	-1.47484400	2.64655400
H	-1.79370400	-1.39032700	2.27292900
H	-0.40849700	-2.47390400	2.35249500
H	-0.78984200	-1.43566300	3.74303700
C	0.64063900	-1.79286400	-0.41212700
C	1.78698300	-1.41234700	-1.32975800
C	0.20314700	-2.20933000	-2.70792200
C	-0.36629800	-2.30855200	-1.30337100
H	0.85997500	-2.33113400	0.51205700
C	1.06250500	-0.94804900	-2.59718100
H	1.74498000	-0.82524000	-3.44884700
H	0.47070200	-0.03014200	-2.46772900
C	2.36997800	-2.76806400	-1.79896000
H	3.33533100	-2.58552600	-2.28828000
H	2.56167000	-3.43885200	-0.94925000
C	1.30155100	-3.29812400	-2.78062700
H	1.69369600	-3.35734100	-3.80482500
H	0.92111200	-4.29285000	-2.51600100
H	2.54540000	-0.74654000	-0.90701800
H	-0.53799800	-2.24746200	-3.51213600
C	-1.19560700	-3.48681700	-0.95740500
O	-1.62814600	-4.28617200	-1.76090500
O	-1.39565600	-3.59553700	0.36952700
C	-2.23333500	-4.66677300	0.79573300
H	-2.27313600	-4.59856200	1.88655800
H	-3.24081900	-4.55659100	0.37686100
H	-1.82093200	-5.63680900	0.49346000
O	4.57675000	1.24368300	1.36857700
K	3.44603600	-1.11670000	1.69626900
O	2.21713500	-3.45050600	1.85440900
S	1.44061100	-4.73231400	2.13843800
C	2.61315900	-5.83898400	2.94727800
H	3.52359400	-5.89909900	2.33831200
H	2.84500600	-5.41955500	3.93196800
H	2.15855100	-6.82959000	3.06572000
C	1.35368400	-5.63509700	0.57787700
H	2.36100400	-5.73417800	0.15400500
H	0.91487800	-6.62329700	0.76238000
H	0.70883700	-5.07119300	-0.10586900
O	5.03455800	-1.42636800	-0.40050200
S	5.80201900	-0.26756500	-1.01891300
C	4.62240500	0.67179000	-2.02075200
H	4.13156000	0.00080400	-2.73810800
H	3.88362100	1.12205000	-1.34551100
H	5.16013300	1.46856400	-2.55024800
C	6.67697500	-0.99611000	-2.42541200
H	5.96728300	-1.56274000	-3.04159000
H	7.15270300	-0.20373600	-3.01590000
H	7.44591200	-1.66921800	-2.03077900

Int21

Pd	-1.67443500	0.53938400	-0.25553100
P	-1.77726000	-1.90143100	0.34371100
C	-0.46681500	-3.15394900	0.20688200
C	-0.15882000	-4.09506800	-0.73314500
O	0.43219400	-3.20411900	1.22853900
C	1.01105900	-4.76174700	-0.26067200
H	-0.70053700	-4.28726200	-1.65339500
C	1.32362500	-4.17884800	0.92758600
H	1.54542500	-5.57248800	-0.74174600
H	2.10881900	-4.34144300	1.65605800
C	-2.89379000	-2.51215500	-0.93501100
C	-3.19324200	-1.94295400	-2.13979300
O	-3.49631300	-3.73405600	-0.83908300
C	-4.03326000	-2.86414900	-2.82776900
H	-2.84694200	-0.96905300	-2.47333200
C	-4.18207500	-3.92477500	-1.98891300
H	-4.47288300	-2.74950300	-3.81160200
H	-4.73353200	-4.85434400	-2.06046700
C	-2.48685500	-2.40763300	1.92791300
C	-2.73124200	-1.71336700	3.07848400
O	-2.73628700	-3.73768800	2.13542300
C	-3.16878100	-2.66378200	4.04422500
H	-2.61536300	-0.64397400	3.21269800
C	-3.15234700	-3.86814400	3.41284200
H	-3.45764100	-2.47545200	5.07110200
H	-3.40276300	-4.87398300	3.72769000
C	0.33203900	0.99894900	-1.38975000
C	0.95174600	0.80962400	-0.13111100
C	1.70066900	-0.32564800	0.21483900
C	1.82021100	-1.31021100	-0.76923700
C	1.24601200	-1.14434600	-2.02737200
C	0.51330600	-0.01204100	-2.38189400
H	2.40436400	-2.20447500	-0.56269200
H	1.39120000	-1.92404300	-2.77775100
H	0.93506200	1.62635200	0.58144300
C	2.43726000	-0.39884100	1.49307800
C	1.83015000	-0.04568600	2.73620400

C	3.82790900	-0.73357200	1.42334900
C	0.45120300	0.28710400	2.87953300
C	2.61533400	-0.01118500	3.93544000
C	4.56034100	-0.71395000	2.67181800
C	-0.08385300	0.68115400	4.08591600
H	-0.20817900	0.23081800	2.01162900
C	2.03733800	0.38794100	5.15997600
C	3.98909400	-0.36749500	3.85896100
H	5.61845500	-0.97466300	2.61432200
C	0.71148200	0.74741800	5.24762600
H	-1.14285500	0.94041300	4.13875900
H	2.67661500	0.40810200	6.04491000
H	4.58173100	-0.34729100	4.77630400
H	0.27974900	1.06364500	6.19588000
I	-4.14071500	0.93749400	0.81290500
C	0.05194400	0.12559900	-3.80088800
H	-1.03953000	0.09214300	-3.89554200
H	0.37411300	1.07977600	-4.24270100
H	0.47254600	-0.68099800	-4.41312700
C	-0.28775200	2.36810900	-1.71425200
C	0.36278400	3.56428900	-0.98186600
C	-1.79512500	3.71195100	-0.35979000
C	-1.74540000	2.44634500	-1.20601600
H	-0.24025000	2.51815700	-2.80317000
C	-0.43568600	3.69465200	0.33103700
H	-0.21822300	4.64155600	0.84699600
H	-0.33366800	2.87712900	1.05504600
C	-0.10769700	4.83488400	-1.70180600
H	0.45052500	5.70807900	-1.33568900
H	0.04870600	4.77692600	-2.78754200
C	-1.60461000	4.91985200	-1.31238000
H	-1.82468500	5.84698400	-0.76544200
H	-2.27814100	4.88426300	-2.17509000
H	1.45403900	3.46699300	-0.88875200
H	-2.68022800	3.78893400	0.27998700
C	-2.87935000	2.31514800	-2.16275400
O	-3.86896100	3.01794000	-2.17293000
O	-2.70766500	1.31274400	-3.05000900
C	-3.80454200	1.07044300	-3.92826600
H	-3.49070400	0.25677000	-4.58952600
H	-4.69418200	0.76959300	-3.35947500
H	-4.03903900	1.96181200	-4.51981100
O	4.44407300	-0.99616000	0.33667700
K	4.06792200	0.77895500	-1.58865400
O	3.85417600	3.10645900	-0.40995600
S	4.14741000	3.88073600	0.86824600
C	3.20020600	3.07753800	2.17944500
H	2.14164300	3.02157400	1.88912100
H	3.61145600	2.06996600	2.31216100
H	3.31287000	3.64949500	3.10888700
C	3.15642300	5.38786300	0.78915600
H	2.09496900	5.13108300	0.68338200
H	3.31916100	5.96752500	1.70593500
H	3.49555300	5.96724100	-0.07639600
O	4.87025300	-1.30292900	-2.99406100
S	4.78334900	-2.69313900	-2.38079300
C	6.22502300	-2.87292800	-1.31393600
H	7.12083500	-2.56567200	-1.86907400
H	6.04032200	-2.22313200	-0.44950200
H	6.31088100	-3.91845300	-0.99309200
C	5.34268900	-3.82148600	-3.67686300
H	6.31925600	-3.48812900	-4.04991800
H	5.41170700	-4.83832700	-3.27200100
H	4.60610600	-3.79868700	-4.48725400

Int22

Pd	1.15661700	-0.35275100	-0.97185200
P	2.08360600	-1.60767600	0.71711800
C	2.44673800	-0.38116800	1.98764100
C	3.59397800	0.07051200	2.57074600
O	1.39133900	0.40260600	2.35983400
C	3.22083700	1.20076300	3.35639100
H	4.58733500	-0.34592100	2.44649600
C	1.87965200	1.35346500	3.19114300
H	3.86875600	1.81906800	3.96613900
H	1.15014200	2.04904900	3.59075000
C	3.64551600	-2.40754200	0.37176700
C	4.34381800	-2.54439800	-0.79320400
O	4.28801700	-3.06766100	1.38345300
C	5.49078500	-3.32720600	-0.48238000
H	4.06313600	-2.12766600	-1.75478300
C	5.40175900	-3.61074600	0.84517600
H	6.27807700	-3.63921700	-1.15745700
H	6.03155500	-4.16841500	1.52751100
C	1.18436900	-2.85783000	1.66297500
C	0.36450000	-2.76588200	2.75246700
O	1.21206100	-4.13977500	1.20397100
C	-0.13547000	-4.08046500	2.98362900

H	0.15188700	-1.87000300	3.32584900
C	0.40713400	-4.86714400	2.01578900
H	-0.79904000	-4.40165300	3.77801000
H	0.33998900	-5.92384900	1.78917900
C	-0.77687900	0.81819800	-1.94104100
C	-0.76403600	1.42192700	-0.66773100
C	-0.43953300	2.77039500	-0.45023500
C	-0.11870100	3.52583900	-1.57949900
C	-0.11963500	2.95378100	-2.85078000
C	-0.45008600	1.61751900	-3.06882700
H	0.11444300	4.58581800	-1.47208900
H	0.11663200	3.57803500	-3.71344800
H	-1.08793200	0.85727600	0.19834000
C	-0.50124400	3.33101000	0.91770100
C	0.60566400	4.03224000	1.48180200
C	-1.68459700	3.09637300	1.68177100
C	1.85411900	4.18941000	0.81425100
C	0.52281000	4.56244500	2.81142800
C	-1.72131700	3.67235600	3.00769800
C	2.92654100	4.81123900	1.41122300
H	1.97404700	3.78185200	-0.18806300
C	1.63560800	5.20981700	3.39311000
C	-0.67901600	4.36988400	3.54294900
H	-2.64035200	3.51765600	3.57629800
C	2.82672300	5.33871800	2.71605700
H	3.86726600	4.88971500	0.86475900
H	1.52980800	5.59763900	4.40836800
H	-0.74584200	4.78248400	4.55219300
H	3.68000700	5.83304400	3.17814000
I	3.41403700	1.29925200	-1.40243600
C	-0.54279600	1.10721800	-4.47492300
H	0.07566900	0.21662000	-4.64082300
H	-1.57839600	0.83078300	-4.73061900
H	-0.22691900	1.88036300	-5.18550000
C	-1.32579500	-0.60967300	-2.10101900
C	-2.59428900	-0.91778900	-1.27379800
C	-1.18265500	-2.55844100	-0.66375000
C	-0.35564100	-1.68863900	-1.58786400
H	-1.53372500	-0.76882300	-3.16911700
C	-2.06063900	-1.52774700	0.04032700
H	-2.86969700	-1.99014300	0.62521900
H	-1.48183700	-0.86273300	0.69530500
C	-3.24675900	-2.16272400	-1.88611800
H	-4.21609800	-2.35157900	-1.40091200
H	-3.42930000	-2.05141600	-2.96316600
C	-2.22898600	-3.28087200	-1.55296600
H	-2.69406000	-4.09781300	-0.98464000
H	-1.77484600	-3.72706700	-2.44704700
H	-3.27501300	-0.05822500	-1.20994500
H	-0.60213900	-3.24409900	-0.04119300
C	0.55296600	-2.42208100	-2.51191800
O	0.97819500	-3.54403300	-2.31939300
O	0.89378600	-1.68406400	-3.58773600
C	1.89553100	-2.24290900	-4.43585700
H	2.05482700	-1.51305800	-5.23349600
H	2.82745900	-2.39747700	-3.87826100
H	1.56240100	-3.19668900	-4.85893900
O	-2.69261200	2.43536400	1.25431300
K	-4.28373900	0.43560500	1.23745800
O	-5.54068400	0.96303700	-0.99903800
S	-5.47378400	2.15449400	-1.94545800
C	-5.38129400	1.46031500	-3.60924100
H	-4.58514800	0.70339700	-3.63314300
H	-6.34621200	1.00053800	-3.84765000
H	-5.16760500	2.26380600	-4.32451600
C	-3.77247400	2.76155800	-1.88680600
H	-3.09942900	2.02573800	-2.34812700
H	-3.71315600	3.71692600	-2.42330900
H	-3.49873800	2.89491100	-0.82807600
O	-4.99209500	-2.03243300	1.82121300
S	-4.70677200	-3.07318900	2.89519300
C	-3.36221000	-2.39673200	3.89537000
H	-2.53244800	-2.09781700	3.23996200
H	-3.75054700	-1.52796300	4.43909700
H	-3.02664600	-3.15100400	4.61747300
C	-3.74309800	-4.38090600	2.10235200
H	-2.81019400	-3.97402800	1.69045200
H	-3.52456000	-5.15887100	2.84443000
H	-4.36145700	-4.80412300	1.30267400

Int23

Pd	0.14728800	-0.96304300	0.95901900
P	2.15408700	-1.56560100	-0.03889600
C	1.74700700	-2.32070700	-1.63503800
C	1.76900600	-3.58992900	-2.12499900
O	1.19578900	-1.47364600	-2.56214200
C	1.21019700	-3.52619000	-3.43945200
H	2.13784400	-4.47123300	-1.61369300

C	0.88508600	-2.22496500	-3.65806800	H	-0.20356300	4.89270200	1.95164200
H	1.08117700	-4.34592900	-4.13676700	H	0.34944100	5.37802900	0.34146000
H	0.47523600	-1.68542400	-4.50405000	H	-1.25495700	5.84443800	0.89541700
C	2.76262600	-2.93545800	0.94554900	C	0.14268300	2.19595000	1.85352000
C	2.39779700	-3.35480200	2.19332200	C	1.51344300	2.89571100	1.59069100
O	3.81383900	-3.68190800	0.48925100	C	2.12159300	0.87909600	2.34130300
C	3.26687000	-4.43375800	2.52222300	C	0.60031100	0.73593500	2.21783900
H	1.61141100	-2.91814400	2.80226800	H	-0.26465300	2.62890500	2.78553700
C	4.09696400	-4.58453900	1.45517100	C	2.42231700	1.76304900	1.13414100
H	3.27552600	-5.01854100	3.43382400	H	3.47639000	2.07145700	1.06471200
H	4.91116900	-5.26579300	1.23971000	H	2.11270800	1.33687800	0.16988000
C	3.68135300	-0.69957400	-0.49034400	C	2.13111600	3.23282900	2.95046200
C	4.13905000	-0.18480000	-1.67031600	H	3.03851400	3.83940800	2.82025700
O	4.54677600	-0.40119400	0.51790600	H	1.44564600	3.79853000	3.59717900
C	5.36929400	0.46983300	-1.36594200	C	2.47556500	1.83035000	3.50859500
H	3.66581100	-0.27164100	-2.64267100	H	3.54377200	1.74777300	3.75357900
C	5.56324800	0.30764200	-0.03027300	H	1.91654100	1.58144300	4.42054800
H	6.02915700	0.98336000	-2.05574100	H	1.46484200	3.73982400	0.89933900
H	6.35115500	0.60585700	0.64998600	H	2.64743100	-0.08006200	2.41146700
C	-0.92803900	2.47081100	0.80386800	C	-0.03504800	0.12984300	3.41598900
C	-1.80181100	1.46703600	0.35787100	O	0.46937100	-0.73718900	4.11484200
C	-2.77608400	1.67574100	-0.62579200	O	-1.26278800	0.62365900	3.66588500
C	-2.87072000	2.95078900	-1.18836200	C	-1.99538300	-0.04271500	4.68579600
C	-2.08699300	3.98374900	-0.69167700	H	-2.98013300	0.43125000	4.71111600
C	-1.15049000	3.78639200	0.32908800	H	-2.09945500	-1.10745800	4.45082800
H	-3.60612200	3.14484800	-1.97079800	H	-1.50636100	0.06788700	5.66104100
H	-2.23016700	4.99678100	-1.07283100	C	-1.91283100	-2.42916600	0.70303800
C	-3.73394300	0.61776300	-1.04819200	O	-2.94647500	-3.04621000	0.41569700
C	-5.00076500	0.54136600	-0.41022700	O	-0.77236500	-2.66128800	0.07119600
C	-3.39077200	-0.25450900	-2.11314800	O	-1.83457600	-1.48857100	1.60922800
C	-5.35443100	1.36304400	0.70000900	K	-1.92231700	-2.96572700	-2.24736900
C	-5.98790900	-0.39107000	-0.86864200	H	-1.76303500	0.48144500	0.82117100
C	-4.44783800	-1.12339900	-2.58797900	O	-2.22720600	-0.30746300	-2.66193500
C	-6.58320900	1.25928600	1.30861100	K	-0.03556000	1.03319300	-2.06072700
H	-4.62745900	2.08302600	1.07677900	O	1.91505700	2.66953200	-2.26611100
C	-7.24309300	-0.46937100	-0.22466900	S	3.25082900	3.33062700	-1.95895900
C	-5.67488300	-1.19855500	-1.99084000	C	2.93109800	5.10655100	-1.97193300
H	-4.24056500	-1.71820000	-3.48296000	H	2.40101900	5.37071700	-2.89555300
C	-7.54744700	0.33572400	0.84817600	H	2.30875000	5.34144400	-1.10082700
H	-6.81580300	1.90019400	2.15917400	H	3.88173700	5.64861900	-1.90093700
H	-7.97173300	-1.18894600	-0.60344000	C	4.18567500	3.27864100	-3.50209500
H	-6.43894100	-1.87672200	-2.37692300	H	3.59206800	3.73980800	-4.30136600
H	-8.51712300	0.26523700	1.33870200	H	5.13673200	3.80861700	-3.37128700
C	-0.52263600	5.02620900	0.91118200	H	4.37864200	2.22695300	-3.74132800

TS_{12}

Pd	0.99681100	0.29012900	0.40148900
P	2.63662200	-1.16278000	-0.31097800
C	1.83284500	-2.30544900	-1.47033900
C	1.87290000	-3.65917600	-1.62558300
O	0.94401300	-1.76454900	-2.36173400
C	0.95466700	-3.97648800	-2.67357700
H	2.48714200	-4.35115200	-1.06036800
C	0.42150000	-2.79432400	-3.08153000
H	0.72856200	-4.95761900	-3.07493500
H	-0.29099600	-2.52756300	-3.85379200
C	3.22153600	-2.23468600	1.00074200
C	2.89301000	-2.27787800	2.32484600
O	4.15669300	-3.19265100	0.71828600
C	3.66880200	-3.32684800	2.89519300
H	2.17955400	-1.63121500	2.82506600
C	4.40997500	-3.84155800	1.87734400
H	3.67712200	-3.65322100	3.92796800
H	5.14054600	-4.63925000	1.82123600
C	4.13958200	-0.75468700	-1.22629800
C	4.36381000	-0.57043200	-2.56100500
O	5.23081400	-0.36659100	-0.50943900
C	5.68193900	-0.03953300	-2.67214000
H	3.67255600	-0.78633400	-3.36885500
C	6.15625900	0.06211200	-1.40205800
H	6.21131200	0.22013800	-3.58091700
H	7.10008000	0.38745900	-0.98298000
C	-0.49798500	2.82207800	0.21108500
C	-0.92395000	1.51491100	0.60417700
C	-2.29165300	1.15916300	0.42256600
C	-3.17355500	2.11139700	-0.10555300
C	-2.76337400	3.40182200	-0.38984400
C	-1.43571500	3.78721500	-0.21636000
H	-4.22078200	1.83658900	-0.23927700
H	-3.48915000	4.14017900	-0.73616400
C	-2.98390500	-0.10258300	0.84175200
C	-3.71530200	-0.08095100	2.06071800
C	-3.12379700	-1.18176300	-0.06739300
C	-3.61380000	0.99045200	2.99582800
C	-4.60273300	-1.15245700	2.40422100
C	-4.06514900	-2.21757000	0.30228800
C	-4.32416800	0.99444100	4.17276000
H	-2.94366500	1.82082100	2.77304000
C	-5.32007600	-1.12060100	3.62205500
C	-4.76337100	-2.21216300	1.47717500
H	-4.22421800	-3.01682700	-0.42916200
C	-5.19186800	-0.07098800	4.50128700
H	-4.21400000	1.83093600	4.86325900
H	-5.98834300	-1.95462800	3.84682300
H	-5.46653900	-3.01450600	1.71199300
H	-5.75022000	-0.05652100	5.43628300
C	-1.10056100	5.23307600	-0.47034900
H	-0.21902800	5.57361700	0.08523600
H	-0.91742500	5.44569800	-1.53326500
H	-1.94665900	5.86439800	-0.16756800
C	0.95891700	3.22640800	0.19430200
C	1.48155000	3.72727500	-1.19732700
C	3.10614000	2.35409500	-0.49970400
C	1.99615400	2.12743700	0.51924500
H	1.08151200	4.06286300	0.90640900
C	2.27856500	2.54703800	-1.76632200
H	2.87197300	2.83355900	-2.64694900
H	1.68484100	1.65457600	-2.01687700
C	2.62663500	4.70245700	-0.90529500
H	2.98273200	5.16775000	-1.83509400
H	2.32657200	5.51027000	-0.22367400
C	3.70458100	3.76730400	-0.30328400
H	4.66020200	3.85530600	-0.83818700
H	3.90767600	3.97832400	0.75615200
H	3.86846100	1.57205700	-0.50466100
C	2.46278900	1.99319800	1.91925400
O	3.53052500	1.51175100	2.26121200
O	1.55706000	2.43920000	2.81543900
C	1.85891900	2.16804800	4.17790600
H	1.01483900	2.54900500	4.75822500
H	1.96709700	1.08959300	4.34458800
H	2.78140000	2.67262000	4.48836200
C	-0.51500800	-1.47303000	1.95926600
O	-0.94255000	-2.52616900	2.47303800
O	-0.03815900	-1.51555200	0.72419500
O	-0.48297000	-0.32085400	2.57218800
K	-1.25424300	-3.57552400	-0.14845900
H	-0.52408300	0.69636600	1.61366100
K	-0.95550200	0.45991300	-2.33966500
O	-2.48425000	-1.29299400	-1.18019500
O	-3.14947900	0.73467400	-3.77034100
S	-4.43038800	0.26722600	-3.08875500

C	-4.58810600	-1.49216300	-3.44859800
H	-4.47909300	-1.65457400	-4.52882500
H	-3.78467500	-1.97756100	-2.88186800
H	-5.56599800	-1.84779000	-3.10035200
C	-5.76925900	0.81867300	-4.16999100
H	-5.57265800	0.47910100	-5.19465300
H	-6.72098600	0.41365600	-3.80523800
H	-5.80096300	1.91310100	-4.13891500
H	0.70403200	4.12782200	-1.85507000

Int24

Pd	0.76654700	0.33755000	0.49100000
P	2.61517400	-0.88545700	-0.62478300
C	1.85711200	-1.60976200	-2.11631000
C	2.01230200	-2.79123500	-2.77954500
O	0.92168800	-0.84167200	-2.76312300
C	1.12271600	-2.75579200	-3.89765700
H	2.68902000	-3.59304900	-2.50819100
C	0.49337900	-1.55214500	-3.84398700
H	0.98228300	-3.52266600	-4.65084500
H	-0.22979500	-1.06337300	-4.48794600
C	3.34502300	-2.31384900	0.19109500
C	3.22538200	-2.74437800	1.48098600
O	4.24995300	-3.09690400	-0.47584000
C	4.09280300	-3.86619900	1.61621000
H	2.57356500	-2.31339300	2.23414600
C	4.68271700	-4.02994700	0.40230700
H	4.25857900	-4.46874600	2.50098400
H	5.40878200	-4.73686900	0.01984200
C	4.07536700	-0.14549500	-1.41359600
C	4.28840500	0.37191600	-2.65854900
O	5.12346600	0.15783100	-0.59684400
C	5.54960600	1.03846200	-2.60342500
H	3.62515600	0.29244600	-3.51357300
C	6.00741300	0.87606700	-1.33465100
H	6.05365700	1.55859900	-3.40914000
H	6.91383100	1.17614900	-0.82396600
C	-0.79135900	2.82114200	0.67593100
C	-0.96998900	1.41712200	0.75992800
C	-2.28133400	0.89792000	0.80805200
C	-3.35758800	1.79527700	0.70658700
C	-3.16883600	3.16512800	0.59168600
C	-1.88367200	3.70685000	0.60065700
H	-4.37220000	1.39277200	0.73212500
H	-4.03155600	3.83113600	0.52763100
C	-2.68364000	-0.52326700	1.02606500
C	-2.90022000	-0.96965000	2.35390000
C	-3.00292600	-1.34531300	-0.08341300
C	-2.54411200	-0.18794400	3.49372400
C	-3.49008900	-2.25302000	2.60454500
C	-3.65814500	-2.59719300	0.21465800
C	-2.73153400	-0.65330500	4.77323300
H	-2.09918600	0.79605300	3.33870200
C	-3.67618100	-2.69885200	3.93330800
C	-3.88345800	-3.03529000	1.49217800
H	-3.99358700	-3.19673300	-0.63810000
C	-3.30348800	-1.92494300	5.00725400
H	-2.43685900	-0.03160000	5.61920100
H	-4.12716400	-3.68135400	4.08799900
H	-4.37392600	-3.99458900	1.67217200
H	-3.44845700	-2.28133700	6.02614700
C	-1.71064500	5.19844500	0.55024900
H	-0.93072200	5.55021200	1.24045200
H	-1.42695600	5.55408400	-0.45088200
H	-2.64712200	5.70548500	0.81688100
C	0.60878700	3.33519200	0.73750400
C	1.15727600	4.05714600	-0.53122700
C	2.76784800	2.58696800	-0.00853600
C	1.63543900	2.19928500	0.93929800
H	0.67470600	4.05162400	1.57718500
C	1.97545500	2.97884600	-1.25431900
H	2.59619100	3.39710900	-2.06082900
H	1.38781300	2.14042800	-1.65783300
C	2.28583700	4.97486400	-0.05301600
H	2.67031400	5.58500100	-0.88251800
H	1.95673300	5.66159500	0.73963800
C	3.34882300	3.95440300	0.42709100
H	4.31992900	4.12390800	-0.05903700
H	3.52046600	3.99695900	1.51223200
H	3.53944400	1.82425100	-0.11570200
C	2.07272900	1.84753000	2.30947500
O	3.11655800	1.27395700	2.58233000
O	1.18354800	2.19622800	3.26736400
C	1.45178700	1.67517400	4.56213800
H	0.64270300	2.02770000	5.20717900
H	1.45719200	0.57660500	4.54272000
H	2.41533000	2.02881900	4.94657300
C	-0.01586400	-2.62161900	1.11607300

O	-0.15326900	-3.83318800	0.93119900
O	-0.00709900	-1.72901700	0.20228400
O	0.15018900	-2.19066800	2.40094300
K	-1.13956400	-3.26503600	-1.61476100
H	0.13725800	-1.21395500	2.37441300
K	-1.43662800	1.06722400	-2.17617100
O	-2.73266400	-1.04411200	-1.30929700
O	-3.82624100	1.46541800	-3.22053800
S	-4.99023400	0.83225900	-2.46721200
C	-5.19799200	-0.82779900	-3.14000500
H	-5.23935700	-0.77412300	-4.23569100
H	-4.32472000	-1.39402600	-2.79259100
H	-6.12018200	-1.26736900	-2.73958200
C	-6.47480300	1.54353000	-3.21409400
H	-6.43268400	1.41258100	-4.30268400
H	-7.36293100	1.05063200	-2.80051600
H	-6.49954700	2.61039900	-2.96706600
H	0.38565700	4.55685000	-1.12930200

Int25

Pd	1.86211000	-0.78265000	-0.63339500
C	3.54127500	0.70729400	1.18921500
C	2.68666600	0.87605000	0.08189700
C	2.56124000	2.12588200	-0.55847200
C	3.44718000	3.13848300	-0.13605900
C	4.31302800	2.96344700	0.93209300
C	4.35290200	1.76117000	1.64129600
H	3.44942700	4.09339000	-0.66195100
H	4.96678200	3.78380200	1.23421200
C	1.60755400	2.42872000	-1.64783000
C	0.94987800	3.70477300	-1.72543200
C	1.30921300	1.45479300	-2.62654100
C	0.90951300	4.63102900	-0.64278800
C	0.19305300	4.06329600	-2.88783300
C	0.62768200	1.88110000	-3.81911600
C	0.22766700	5.82212700	-0.72509000
H	1.40013600	4.37498000	0.29372900
C	-0.47583600	5.30855000	-2.95670000
C	0.09613300	3.13242800	-3.95114000
H	0.55412600	1.15515100	-4.63296700
C	-0.45986400	6.18659400	-1.90163400
H	0.21206700	6.48793900	0.13783900
H	-1.02279600	5.54546400	-3.87115600
H	-0.42813300	3.42242800	-4.86338800
H	-0.98437600	7.13908400	-1.96078400
C	5.25091600	1.62551400	2.83664500
H	4.75806400	1.10138800	3.66833100
H	6.16575300	1.05811500	2.60992900
H	5.56676600	2.61151500	3.20188200
C	3.49438600	-0.61679000	1.88045100
C	4.77493000	-1.49251000	1.77735300
C	3.13969100	-2.89105900	1.13530500
C	2.40737000	-1.55261200	1.27959500
H	3.29773800	-0.43953800	2.95402500
C	4.48935600	-2.41806000	0.58985800
H	5.23335500	-3.22390600	0.50157400
H	4.40133000	-1.89736800	-0.37493200
C	4.72808300	-2.49503000	2.93407700
H	5.66520300	-3.06709500	2.98930800
H	4.58628700	-2.00045600	3.90601000
C	3.54108500	-3.40898400	2.53587900
H	3.85018000	-4.46276700	2.48399700
H	2.70346800	-3.35463700	3.24516500
H	5.70384600	-0.91116300	1.72333600
H	2.60123200	-3.63358100	0.53757400
C	1.11365600	-1.61357400	1.95696500
O	0.34944600	-2.58055500	1.95594400
O	0.77878900	-0.48002100	2.61200600
C	-0.55877900	-0.41108000	3.08228900
H	-0.66026200	0.56560800	3.56513900
H	-1.26479800	-0.48122000	2.23932500
H	-0.77547100	-1.20646600	3.80546700
C	-3.46572900	1.37531400	2.94991700
C	-2.74492800	2.20980400	2.06456300
C	-2.16461300	3.39521000	2.53595400
C	-2.30087200	3.70246600	3.87717700
C	-3.00696400	2.85207900	4.75247900
C	-3.59620400	1.68056700	4.30815400
C	-3.46346100	0.37031900	0.95052300
C	-2.75240500	1.53799100	0.81226600
H	-1.60875700	4.04464300	1.85914900
H	-1.85260900	4.61360200	4.26950900
H	-3.09089800	3.12390000	5.80341300
H	-4.13991000	1.02555000	4.98695700
Br	-1.95554200	2.22718800	-0.73793200
C	-4.46937300	-0.89627000	2.91347100
H	-3.77592600	-1.25859800	3.68383100
H	-5.42858100	-0.64937300	3.38312200

H	-4.63478400	-1.69522400	2.18533300
C	-3.71564000	-0.67841500	-0.06740600
O	-2.76832800	-1.21510600	-0.67289000
N	-4.99623200	-1.00053000	-0.27639600
H	-5.71300500	-0.49443100	0.23700900
C	-5.41469900	-2.08369700	-1.15511100
H	-6.35729000	-1.78488700	-1.62987800
H	-4.66217200	-2.18688400	-1.94664000
C	-5.58836200	-3.39540700	-0.40578100
H	-4.64980100	-3.63645400	0.12135700
H	-6.35058400	-3.26645700	0.37738900
C	-5.96832900	-4.52466000	-1.34450100
H	-5.19500000	-4.67897800	-2.11008400
H	-6.09879600	-5.46960800	-0.80325000
H	-6.91103300	-4.30366400	-1.86407700
N	-3.91491900	0.27413400	2.25664500
K	-1.06569700	-0.46961800	-2.60488600
I	0.91335500	-3.26586600	-1.98632800
O	1.59816000	0.18647800	-2.54456100
K	-1.57201700	-3.41697600	0.47292300

TS_{13}

Pd	-0.47828800	0.03760200	-0.28936900
C	-3.05690800	-0.54202500	-1.48371600
C	-1.89450400	-1.13811500	-0.97700300
C	-1.69732500	-2.52202600	-0.86066800
C	-2.82827700	-3.29836200	-1.19231200
C	-4.04569200	-2.72339700	-1.52983200
C	-4.19146700	-1.34236500	-1.69139400
H	-2.74913300	-4.38500900	-1.17119600
H	-4.90500500	-3.36917500	-1.72000100
C	-0.41410300	-3.18529500	-0.51587300
C	-0.39545400	-4.38723300	0.26955700
C	0.82144800	-2.65439300	-0.98381700
C	-1.50556300	-4.82695900	1.04784300
C	0.80012000	-5.16851200	0.37860500
C	1.97698500	-3.51316700	-0.92779000
C	-1.45366100	-5.96994400	1.81123500
H	-2.41195900	-4.22490000	1.06572000
C	0.81687600	-6.35490400	1.14859300
C	1.97005000	-4.71731000	-0.28200200
H	2.88196500	-3.14517000	-1.41838000
C	-0.28993900	-6.76613300	1.85051500
H	-2.32400800	-6.25674900	2.40108500
H	1.74377500	-6.93016700	1.18222200
H	2.86881600	-5.33561100	-0.24669300
H	-0.26625300	-7.67778000	2.44536700
C	-5.51962700	-0.77731500	-2.10433300
H	-5.62365200	0.28034500	-1.83046000
H	-5.68759600	-0.85348200	-3.18886200
H	-6.33631800	-1.33049100	-1.62091800
C	-2.89915500	0.87200100	-1.97426600
C	-3.08745200	0.89296800	-3.51347300
C	-0.86999300	1.15636900	-3.30133700
C	-1.39756700	1.26647300	-1.84169000
H	-3.57042500	1.58807400	-1.47631100
C	-1.81978200	0.16486600	-3.97228100
H	-1.69953900	0.16326100	-5.06459800
H	-1.73619100	-0.86604700	-3.60678200
C	-2.84480900	2.31706000	-4.03936700
H	-3.25344300	2.43630300	-5.05127800
H	-3.32966900	3.07029300	-3.40190900
C	-1.30112300	2.44420900	-4.03446000
H	-0.90213000	2.43194900	-5.05908100
H	-0.94061800	3.36448700	-3.55951500
H	-4.03748500	0.45991800	-3.84432500
H	0.19386500	0.92269400	-3.42329100
C	-1.00673300	2.55152700	-1.24883000
O	0.13864000	3.00243400	-1.30881600
O	-1.99668400	3.23002400	-0.64484300
C	-1.64727100	4.51739000	-0.14724500
H	-2.47239200	4.83506900	0.49587900
H	-0.70798900	4.48319700	0.41370300
H	-1.53394600	5.22510900	-0.97896100
C	-2.45331900	2.48144100	2.63239500
C	-2.52981800	1.15776300	2.13126600
C	-3.76332600	0.49979200	2.05350000
C	-4.90059900	1.17685100	2.45422500
C	-4.81616300	2.49750400	2.93951200
C	-3.60717000	3.16362600	3.04024900
C	-0.36641600	1.86673200	2.09122200
C	-1.19988200	0.79088700	1.75944900
H	-3.81615100	-0.52338800	1.67808800
H	-5.87200400	0.68903900	2.39757800
H	-5.72699600	3.00800800	3.24872500
H	-3.56195100	4.18093200	3.42518000
Br	-0.61013700	-1.10221200	2.23188800
C	-0.69125200	4.19764800	3.07743400

H	-1.53580800	4.89255400	3.08455400
H	-0.27802300	4.13937100	4.09386000
H	0.06869100	4.60843800	2.40331200
C	1.03036300	1.88498000	1.71514100
O	1.40598000	1.10818500	0.78554900
N	1.91228600	2.67899300	2.34086800
H	1.60824500	3.21125300	3.14848700
C	3.22775500	2.97672600	1.78985100
H	3.86234300	3.32034500	2.61528900
H	3.67498800	2.04864900	1.40458100
C	3.14899500	4.03067000	0.69376100
H	2.39760300	3.71662400	-0.05012200
H	2.76433400	4.96644000	1.12813300
C	4.49779200	4.26434400	0.03829500
H	4.89007200	3.33971700	-0.41240100
H	4.43496600	5.02310800	-0.75151400
H	5.23931500	4.60763500	0.77345500
N	-1.14413700	2.90085100	2.61854500
K	2.61278400	-1.36850000	1.18062000
I	5.31499300	-0.10273800	-0.65451300
O	0.99664700	-1.44353800	-1.42062100
K	2.21885500	1.17039600	-1.78358300

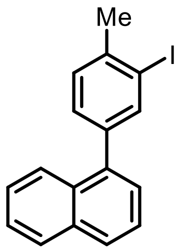

1a

1-(3-iodo-4-methylphenyl)naphthalene （1a）

Physical state: yellow oil.

Yield: 0.43 g, 63%.

^{1}H NMR (500 MHz, Chloroform-d) δ 8.04 (d, J = 1.8 Hz, 1H), 7.95 (d, J = 8.6 Hz, 2H), 7.91 (d, J = 8.2 Hz, 1H), 7.57 – 7.53 (m, 2H), 7.52 – 7.48 (m, 1H), 7.45 – 7.42 (m, 2H), 7.39 (d, J = 7.7 Hz, 1H), 2.59 (s, 3H).

^{13}C NMR (126 MHz, Chloroform-d) δ 140.21, 140.03, 139.99, 138.23, 133.69, 131.40, 129.85, 129.31, 128.27, 127.90, 126.92, 126.18, 125.81, 125.65, 125.26, 101.02, 27.82.

HRMS (ESI-TOF): calcd for $C_{17}H_{14}I^+$ [M+H$^+$] 345.0135, found 345.0140.

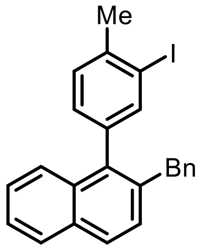

1b

2-benzyl-1-(3-iodo-4-methylphenyl)naphthalene （1b）

Physical state: yellow oil.

Yield: 0.45 g, 52%.

^{1}H NMR (500 MHz, Chloroform-d)δ 7.86 (dd, J = 16.8, 8.2 Hz, 2H), 7.74 (s, 1H), 7.49 – 7.45 (m, 2H), 7.41 (t, J = 7.5 Hz, 2H), 7.35 (d, J = 7.6 Hz, 1H), 7.26 (t, J = 7.3 Hz, 2H), 7.19 (t, J = 7.8 Hz, 2H), 7.04 (d, J = 7.4 Hz, 2H), 4.01 – 3.94 (m, 2H), 2.57 (s, 3H).

^{13}C NMR (126 MHz, Chloroform-*d*) δ 141.19, 140.47, 140.17, 138.41, 136.65, 136.12, 132.91, 132.09, 130.20, 129.39, 128.81, 128.24, 128.13, 127.88, 127.77, 126.33, 126.04, 125.86, 125.24, 101.02, 39.63, 27.87.

HRMS (ESI-TOF): calcd for $C_{24}H_{20}I^+$ [M+H$^+$] 435.0604, found 435.0606.

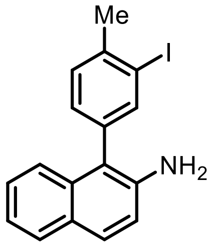

1c

1-(3-iodo-4-methylphenyl)naphthalen-2-amine（1c）

Physical state: yellow oil.
Yield: 0.37 g, 51%.
^{1}H NMR (500 MHz, Chloroform-d) δ 7.74 (d, *J* = 7.9 Hz, 1H), 7.70 (d, *J* = 8.7 Hz, 1H), 7.37 (d, *J* = 7.8 Hz, 2H), 7.33 (d, *J* = 8.4 Hz, 1H), 7.30 – 7.26 (m, 3H), 7.23 (t, *J* = 7.2 Hz, 1H), 7.05 (d, *J* = 8.7 Hz, 1H), 3.74 (s, 2H), 2.48 (s, 3H).
^{13}C NMR (126 MHz, Chloroform-d) δ 141.09, 137.10, 133.93, 133.87, 130.70, 129.94, 128.51, 127.95, 127.84, 126.16, 124.27, 122.05, 119.88, 118.05, 21.29.
HRMS (ESI-TOF): calcd for $C_{17}H_{15}I^+$ [M+H$^+$] 360.0244, found 360.0240.

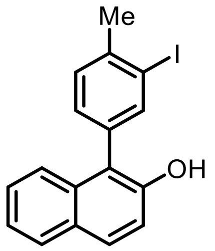

1d

1-(3-iodo-4-methylphenyl)naphthalen-2-ol (1d)
Physical state: colorless oil.
Yield: 0.36 g, 49%.
^{1}H NMR (500 MHz, Chloroform-d) δ 7.96 (s, 1H), 7.88 – 7.83 (m, 2H), 7.47 (t, 2H), 7.43 – 7.35 (m, 3H), 7.29 (d, *J* = 8.8 Hz, 1H), 5.20 (s, 1H), 2.60 (s, 3H).
^{13}C NMR (126 MHz, Chloroform-d) δ 150.15, 141.69, 141.13, 133.41, 133.10, 130.93, 130.56, 129.74, 128.79, 128.01, 126.61, 124.36, 123.36, 119.23, 117.30, 102.22, 27.97.
HRMS (ESI-TOF): calcd for $C_{17}H_{14}I^+$ [M+H$^+$] 345.0315, found 345.0319.

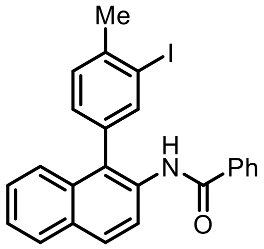

1e

N-(1-(3-iodo-4-methylphenyl)naphthalen-2-yl)benzamide （1e）
Physical state: yellow solid.
Yield: 0.51 g, 55%.
^{1}H NMR (500 MHz, Chloroform-d) δ 8.67 (d, *J* = 9.0 Hz, 1H), 7.94 (d, *J* = 6.9 Hz, 2H), 7.88 (d, *J* = 8.0 Hz, 2H), 7.61 (d, *J* = 7.6 Hz, 2H), 7.50 (t, *J* = 7.3 Hz, 1H), 7.47 – 7.38 (m, 6H), 7.32 (d, *J* = 7.4 Hz, 1H), 2.58 (s, 3H).
^{13}C NMR (126 MHz, Chloroform-d) δ 165.16, 141.76, 140.64, 134.81, 134.72, 133.24, 132.39, 131.76, 130.75, 130.56, 130.49, 128.86, 128.74, 127.98, 126.84, 126.51, 125.69, 125.45, 124.91, 120.38, 101.90, 27.97.
HRMS (ESI-TOF): calcd for $C_{24}H_{19}INO^+$ [M+H$^+$] 464.0506, found 464.0505.

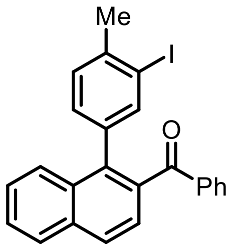

1f

(1-(3-iodo-4-methylphenyl)naphthalen-2-yl)(phenyl)methanone (1f)

Physical state: yellow solid.

Yield: 0.54 g, 60%.

^{1}H NMR (500 MHz, Chloroform-d) δ 8.03 – 8.00 (m, 2H), 7.98 – 7.93 (m, 3H), 7.69 (d, *J* = 8.4 Hz, 1H), 7.61 – 7.57 (m, 1H), 7.54 – 7.50 (m, 1H), 7.49 – 7.43 (m, 4H), 7.34 – 7.32 (m, 1H), 7.28 (d, *J* = 7.8 Hz, 1H), 2.45 (s, 3H).

^{13}C NMR (126 MHz, Chloroform-d) δ 165.20, 145.62, 140.48, 140.41, 134.02, 133.32, 132.91, 131.72, 130.18, 129.92, 129.24, 129.18, 129.07, 128.92, 128.33, 128.03, 126.62, 125.74, 125.55, 121.55, 100.64, 27.75.

HRMS (ESI-TOF): calcd for $C_{24}H_{18}IO^+$ [M+H$^+$] 449.0397, found 449.0393.

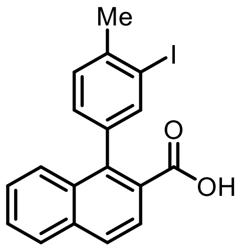

1g

1-(3-iodo-4-methylphenyl)-2-naphthoic acid (1g)

Physical state: white solid.

Yield: 0.66 g, 86%.

^{1}H NMR (500 MHz, Chloroform-d) δ 10.17 (s, 1H), 8.08 – 8.01 (m, 1H), 7.91 (t, *J* = 7.8 Hz, 2H), 7.78 (s, 1H), 7.58 (t, *J* = 8.3 Hz, 2H), 7.45 (t, *J* = 7.7 Hz, 1H), 7.34 (d, *J* = 7.6 Hz, 1H), 7.21 (d, *J* = 7.9 Hz, 1H), 2.56 (s, 3H).

^{13}C NMR (126 MHz, Chloroform-d) δ 172.92, 140.87, 140.30, 139.39, 137.79, 135.11, 132.60, 129.48, 129.02, 128.02, 127.91, 127.84, 126.78, 126.35, 125.87, 100.51, 27.97.

HRMS (ESI-TOF): calcd for $C_{18}H_{14}IO_2^+$ [M+H$^+$] 389.0033, found 389.0027.

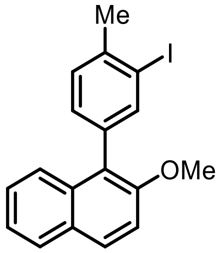

1h

1-(3-iodo-4-methylphenyl)-2-methoxynaphthalene (1h)

Physical state: white solid.

Yield: 0.42 g, 52%.

^{1}H NMR (500 MHz, Chloroform-d) δ 7.91 (d, *J* = 9.0 Hz, 1H), 7.89 (d, *J* = 1.5 Hz, 1H), 7.85 (dd, *J* = 6.6, 2.7 Hz, 1H), 7.56 – 7.52 (m, 1H), 7.41 – 7.35 (m, 4H), 7.32 – 7.29 (m, 1H), 3.88 (s, 3H), 2.57 (s, 3H).

^{13}C NMR (126 MHz, Chloroform-d) δ 153.70, 140.96, 139.93, 135.62, 133.38, 130.85, 129.38, 129.34, 128.86, 127.86, 126.48, 124.92, 123.52, 123.25, 113.40, 100.97, 56.60, 27.92.

HRMS (ESI-TOF): calcd for $C_{18}H_{16}I^+$ [M+H$^+$] 375.0240, found 375.0245.

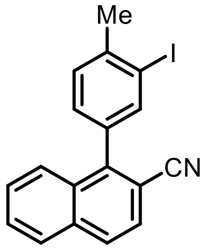

1i

1-(3-iodo-4-methylphenyl)-2-naphthonitrile (1i)

Physical state: yellow solid.

Yield: 0.53 g, 72%.

^{1}H NMR (500 MHz, Chloroform-*d*) δ 7.93 (d, *J* = 8.7 Hz, 2H), 7.90 (d, *J* = 1.8 Hz, 1H), 7.70 (t, *J* = 9.1 Hz, 2H), 7.64 (t, *J* = 7.4 Hz, 1H), 7.53 (t, *J* = 7.7 Hz, 1H), 7.43 (d, *J* = 7.7 Hz, 1H), 7.36 (dd, *J* = 7.7, 1.4 Hz, 1H), 2.55 (s, 3H).

^{13}C NMR (126 MHz, Chloroform-*d*) δ 144.36, 142.15, 139.91, 135.51, 134.81, 131.49, 129.79, 129.74, 128.76, 128.27, 127.76, 127.05, 126.61, 118.62, 109.84, 101.06, 28.01.

HRMS (ESI-TOF): calcd for $C_{18}H_{12}IN^+$ [M+H$^+$] 370.0087, found 370.0085.

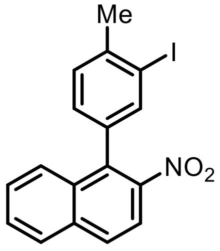

1j

1-(3-iodo-4-methylphenyl)-2-nitronaphthalene (1i)

Physical state: yellow solid.

Yield: 0.52 g, 67%.

^{1}H NMR (500 MHz, Chloroform-*d*) δ 7.99 – 7.91 (m, 3H), 7.79 (s, 1H), 7.66 – 7.60 (m, 2H), 7.55 – 7.50 (m, 1H), 7.37 (d, *J* = 7.7 Hz, 1H), 7.23 (d, *J* = 7.7 Hz, 1H), 2.54 (s, 3H).

^{13}C NMR (126 MHz, Chloroform-*d*) δ 146.44, 141.53, 139.17, 134.58, 133.88, 132.71, 132.33, 129.52, 129.33, 129.24, 128.61, 128.11, 128.04, 127.95, 119.86, 100.96, 27.97.

HRMS (ESI-TOF): calcd for $C_{17}H_{13}INO_2^+$ [M+H$^+$] 389.9985, found 389.9988.

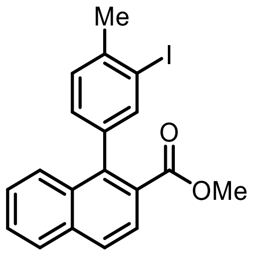

1k

methyl 1-(3-iodo-4-methylphenyl)-2-naphthoate (1k)

Physical state: light yellow oil.

Yield: 0.51 g, 63%.

^{1}H NMR (500 MHz, Chloroform-d) δ 7.96 – 7.88 (m, 3H), 7.77 (d, J = 4.4 Hz, 1H), 7.61 – 7.54 (m, 2H), 7.47 – 7.43 (m, 1H), 7.35 (d, J = 7.6 Hz, 1H), 7.21 (d, J = 7.6 Hz, 1H), 3.69 (s, 3H), 2.54 (s, 3H).

^{13}C NMR (126 MHz, Chloroform-d) δ 168.11, 140.16, 139.66, 139.41, 138.04, 134.62, 132.39, 129.48, 128.94, 127.91, 127.81, 127.70, 127.50, 127.48, 126.68, 125.32, 100.46, 51.99, 27.90.

HRMS (ESI-TOF): calcd for $C_{19}H_{16}IO_2^+$ [M+H$^+$] 403.0189, found 403.0193.

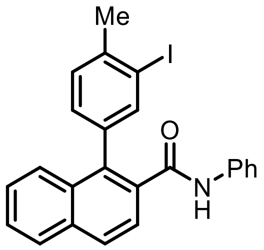

1l

1-(3-iodo-4-methylphenyl)-N-phenyl-2-naphthamide (1l)

Physical state: white solid.

Yield: 0.49 g, 53%.

^{1}H NMR (500 MHz, Chloroform-d) δ 7.93 (s, 1H), 7.88 – 7.73 (m, 3H), 7.64 (d, J = 8.3 Hz, 1H), 7.54 (t, J = 7.0 Hz, 1H), 7.47 (t, J = 7.2 Hz, 1H), 7.39 – 7.17 (m, 7H), 7.06 (t, J = 7.2 Hz, 1H), 2.49 (s, 3H).

^{13}C NMR (126 MHz, Chloroform-d) δ 167.11, 141.41, 139.79, 137.46, 136.94, 135.29, 134.09, 133.19, 131.74, 129.97, 129.90, 128.72, 128.41, 128.01, 127.08, 126.89, 125.00, 124.35, 119.92, 101.30, 27.84.

HRMS (ESI-TOF): calcd for $C_{24}H_{19}INO^+$ [M+H$^+$] 464.0506, found 464.0508.

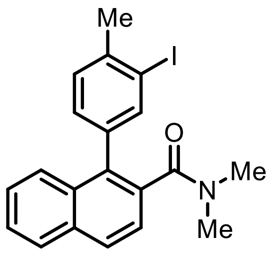

1m

1-(3-iodo-4-methylphenyl)-N,N-dimethyl-2-naphthamide（1m）

Physical state: yellow solid.

Yield: 0.40 g, 48%.

^{1}H NMR (500 MHz, Chloroform-d) δ 7.89 (s, 3H), 7.71 (d, *J* = 8.4 Hz, 1H), 7.52 (d, *J* = 14.7 Hz, 1H), 7.50 – 7.36 (m, 3H), 7.33 (d, *J* = 6.0 Hz, 1H), 2.87 (s, 3H), 2.66 (s, 3H), 2.51 (s, 3H).

^{13}C NMR (126 MHz, Chloroform-d) δ 170.74, 140.74, 136.47, 133.98, 133.90, 133.42, 131.68, 128.50, 128.06, 126.78, 126.38, 126.14, 123.39, 38.35, 34.31, 27.82.

HRMS (ESI-TOF): calcd for $C_{20}H_{19}INO^+$ [M+H$^+$] 416.0506, found 416.0503.

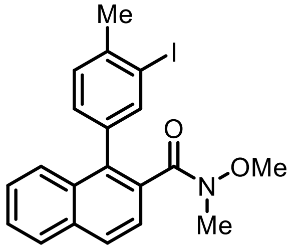

1n

1-(3-iodo-4-methylphenyl)-N-methoxy-N-methyl-2-naphthamide (1n)

Physical state: light yellow solid.

Yield: 0.40 g, 46%.

^{1}H NMR (500 MHz, Chloroform-d) δ 7.99 – 7.81 (m, 3H), 7.71 (d, *J* = 8.5 Hz, 1H), 7.51 (t, *J* = 7.4 Hz, 1H), 7.49 – 7.36 (m, 3H), 7.33 (d, *J* = 6.1 Hz, 1H), 2.87 (s, 3H), 2.65 (s, 3H), 2.51 (s, 3H).

^{13}C NMR (126 MHz, Chloroform-d) δ 170.76, 140.76, 139.62, 136.50, 134.00, 133.93, 133.44, 131.70, 130.62, 129.71, 128.52, 128.08, 126.80, 126.40, 126.17, 123.4, 38.37, 34.33, 27.84.

HRMS (ESI-TOF): calcd for $C_{20}H_{19}INO_2^+$ [M+H$^+$] 432.0455, found 432.0457.

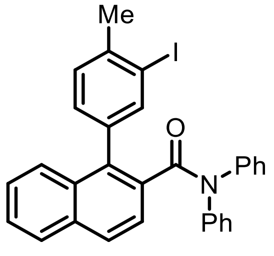

1o

1-(3-iodo-4-methylphenyl)-N,N-diphenyl-2-naphthamide (1o)

Physical state: white solid.

Yield: 0.45 g, 42%.

^{1}H NMR (500 MHz, Chloroform-d) δ 7.91 (d, *J* = 8.5 Hz, 1H), 7.85 (d, *J* = 8.1 Hz, 1H), 7.78 (d, *J* = 8.4 Hz, 1H), 7.58 (d, *J* = 8.5 Hz, 1H), 7.48 (t, *J* = 7.4 Hz, 1H), 7.41 – 6.93 (m, 12H), 6.54 (s, 2H), 2.56 (s, 3H).

^{13}C NMR (126 MHz, Chloroform-d) δ 170.76, 142.72, 141.76, 136.13, 133.99, 133.96, 133.61, 131.29, 131.01, 129.25, 129.06, 128.87, 128.13, 128.07, 126.68, 126.55, 126.42, 126.12, 125.89, 120.85, 117.69, 100.51, 27.96.

HRMS (ESI-TOF): calcd for $C_{30}H_{23}INO^+$ [M+H$^+$] 540.0819, found 540.0822.

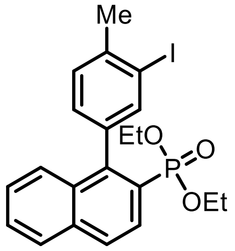

1p

diethyl (1-(3-iodo-4-methylphenyl)naphthalen-2-yl)phosphonate (1p)

Physical state: yellow solid.

Yield: 0.58 g, 61%.

^{1}H NMR (500 MHz, Chloroform-d) δ 8.11 – 8.07 (m, 1H), 7.94 – 7.91 (m, 1H), 7.90 (d, *J* = 1.7 Hz, 1H), 7.86 (d, *J* = 2.0 Hz, 1H), 7.59 – 7.54 (m, 1H), 7.46 – 7.41 (m, 1H), 7.31 – 7.29 (m, 1H), 4.05 – 3.84 (m, 4H), 1.26 (t, *J* = 7.1 Hz, 3H), 1.17 (t, *J* = 7.1 Hz, 3H).

^{13}C NMR (126 MHz, Chloroform-d) δ 143.23 (d, *J* = 9.7 Hz), 140.58, 140.40, 137.39 (d, *J* = 5.6 Hz), 134.93 (d, *J* = 2.7 Hz), 132.72 (d, *J* = 15.9 Hz), 130.46, 128.47, 128.30 (d, *J* = 9.8 Hz), 127.80 (d, *J* = 4.0 Hz), 127.60, 127.48, 127.35, 126.63, 124.83 (d, *J* = 187.5 Hz), 99.92, 61.81 (d, *J* = 5.8 Hz), 61.67 (d, *J* = 5.9 Hz), 27.89, 16.27 (d, *J* = 6.9 Hz), 16.13 (d, *J* = 6.9 Hz).

^{31}P NMR (202 MHz, Chloroform-*d*) δ +18.04.

HRMS (ESI-TOF): calcd for $C_{21}H_{23}IO_3P^+$ [M+H$^+$] 481.0424, found 481.0420.

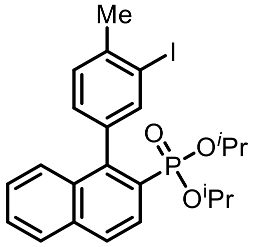

1q

diethyl (1-(3-iodo-4-methylphenyl)naphthalen-2-yl)phosphonate (1q)

Physical state: yellow solid.

Yield: 0.66 g, 65%.

^{1}H NMR (500 MHz, Chloroform-d) δ 8.12 (dd, *J* = 12.4, 8.6 Hz, 1H), 7.92 (dd, *J* = 8.6, 3.6 Hz, 1H), 7.88 (d, *J* = 8.2 Hz, 1H), 7.79 (d, *J* = 1.3 Hz, 1H), 7.54 (t, *J* = 7.4 Hz, 1H), 7.46 (d, *J* = 8.5 Hz, 1H), 7.42 – 7.38 (m, 1H), 7.32 (d, *J* = 7.7 Hz, 1H), 7.27 – 7.24 (m, 1H), 4.72 – 4.65 (m, 1H), 4.61 – 4.54 (m, 1H), 2.53 (s, 3H), 1.23 (dd, *J* = 6.2, 2.9 Hz, 6H), 1.14 (dd, *J* = 10.2, 6.1 Hz, 6H).

^{13}C NMR (126 MHz, Chloroform-d) δ 142.90 (d, *J* = 9.5 Hz), 140.76, 140.31, 137.67 (d, *J* = 5.2 Hz), 134.81 (d, *J* = 2.6 Hz), 132.82 (d, *J* = 15.9 Hz), 130.93, 128.31, 128.03 (d, *J* = 10.3 Hz), 127.80, 127.59, 127.46, 127.41, 127.34, 126.52 (d, *J* = 189.4 Hz), 126.51, 99.88, 70.76 (d, *J* = 6.3 Hz), 70.46 (d, *J* = 6.5 Hz), 27.88, 24.00 (d, *J* = 4.8 Hz), 23.95 (d, *J* = 4.5 Hz), 23.72 (d, *J* = 4.2 Hz).

^{31}P NMR (202 MHz, Chloroform-*d*) δ +16.09.

HRMS (ESI-TOF): calcd for $C_{23}H_{27}IO_3P^+$ [M+H$^+$] 509.0737, found 509.0741.

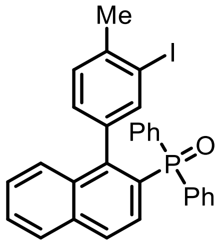

1r

(1-(3-iodo-4-methylphenyl)naphthalen-2-yl)diphenylphosphine oxide (1r)

Physical state: yellow solid.

Yield: 0.59 g, 55%.

1H NMR (500 MHz, Chloroform-d) δ 7.88 – 7.83 (m, 2H), 7.69 – 7.64 (m, 2H), 7.59 – 7.43 (m, 8H), 7.40 – 7.36 (m, 3H), 7.33 – 7.28 (m, 2H), 7.25 (d, *J* = 1.8 Hz, 1H), 7.19 – 7.15 (m, 1H), 7.04 (d, *J* = 7.7 Hz, 1H), 2.35 (s, 3H).

13C NMR (126 MHz, Chloroform-d) δ 144.42 (d, *J* = 8.6 Hz), 141.05, 140.23, 135.72 (d, *J* = 5.4 Hz), 134.60 (d, *J* = 2.1 Hz), 133.44 (d, *J* = 14.5 Hz), 132.86 (d, *J* = 11.3 Hz), 132.61 (d, *J* = 14.6 Hz), 131.67 (d, *J* = 9.3 Hz), 131.56, 131.30 (d, *J* = 2.7 Hz), 131.24, 131.21, 131.13, 129.45 (d, *J* = 102.9 Hz), 128.11, 128.11 (d, *J* = 12.2 Hz), 127.97 (d, *J* = 12.6 Hz), 127.82, 127.79, 127.28 (d, *J* = 12.4 Hz), 126.86 (d, *J* = 33.6 Hz), 99.89, 27.66.

^{31}P NMR (202 MHz, Chloroform-*d*) δ +27.73.

HRMS (ESI-TOF): calcd for $C_{29}H_{22}IOP^+$ [M+H$^+$] 545.0526, found 545.0523.

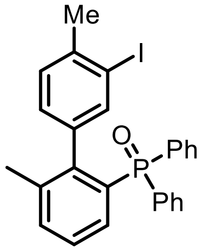

1s

(3'-iodo-4',6-dimethyl-[1,1'-biphenyl]-2-yl)diphenylphosphine oxide (1s)

Physical state: yellow solid.

Yield: 0.54 g, 53%.

^{1}H NMR (500 MHz, Chloroform-d) δ 7.63 (dd, *J* = 11.6, 7.3 Hz, 2H), 7.47 – 7.43 (m, 4H), 7.41 (d, *J* = 7.2 Hz, 1H), 7.38 – 7.35 (m, 2H), 7.33 – 7.27 (m, 3H), 7.25 – 7.23 (m, 1H), 7.05 (s, 1H), 7.02 – 6.96 (m, 2H), 2.29 (s, 3H), 2.00 (s, 3H).

^{13}C NMR (126 MHz, Chloroform-d) δ 144.26 (d, *J* = 8.8 Hz), 140.23, 139.71, 138.15 (d, *J* = 9.8 Hz), 137.01 (d, *J* = 4.7 Hz), 133.78 (d, *J* = 2.5 Hz), 133.29 (d, *J* = 11.4 Hz), 132.47 (d, *J* = 11.6 Hz), 132.46 (d, *J* = 102.5 Hz), 131.77, 131.70, 131.41 (d, *J* = 2.7 Hz), 131.25 (d, *J* = 2.2 Hz), 131.22, 131.17, 131.09, 130.71, 128.26, 128.13, 128.03, 127.97, 127.87, 126.90 (d, *J* = 13.3 Hz), 99.88, 27.63, 20.73.

^{31}P NMR (202 MHz, Chloroform-*d*) δ +27.74.

HRMS (ESI-TOF): calcd for $C_{26}H_{23}IOP^+$ [M+H$^+$] 509.0526, found 509.0528.

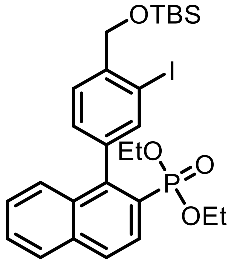

1t

diethyl (1-(4-(((tert-butyldimethylsilyl)oxy)methyl)-3-iodophenyl)naphthalen-2-yl)phosphonate (1t)

Physical state: colorless oil.

Yield: 0.70 g, 57%.

^{1}H NMR (500 MHz, Chloroform-d) δ 8.14 (dd, *J* = 12.2, 8.6 Hz, 1H), 7.97 (dd, *J* = 8.5, 3.7 Hz, 1H), 7.92 (d, *J* = 8.2 Hz, 1H), 7.81 (s, 1H), 7.64 (d, *J* = 7.8 Hz, 1H), 7.57 (d, *J* = 7.2 Hz, 1H), 7.49 (d, *J* = 8.4 Hz, 1H), 7.44 (d, *J* = 7.5 Hz, 1H), 7.42 – 7.39 (m, 1H), 4.77 (s, 2H), 4.04 – 3.86 (m, 4H), 1.27 (t, *J* = 7.0 Hz, 3H), 1.18 (t, *J* = 7.1 Hz, 3H), 1.03 (s, 9H), 0.22 (s, 6H).

^{13}C NMR (126 MHz, Chloroform-d) δ 143.41 (d, *J* = 9.7 Hz), 142.29, 140.28, 138.62 (d, *J* = 5.4 Hz), 135.06 (d, *J* = 2.4 Hz), 132.80 (d, *J* = 15.8 Hz), 130.52, 128.42 (d, *J* = 10.2 Hz), 127.91, 127.69 (d, *J* = 14.5 Hz), 127.50, 126.75, 126.20, 124.88 (d, *J* = 187.3 Hz), 94.60, 69.34, 61.92 (d, *J* = 5.9 Hz), 61.80 (d, *J* = 5.9 Hz), 26.00, 18.45, 16.36 (d, *J* = 6.9 Hz), 16.20 (d, *J* = 6.9 Hz), -5.22.

^{31}P NMR (202 MHz, Chloroform-*d*) δ +18.07.

HRMS (ESI-TOF): calcd for $C_{27}H_{37}IO_4PSi^+$ [M+H$^+$] 611.1238, found 611.1242.

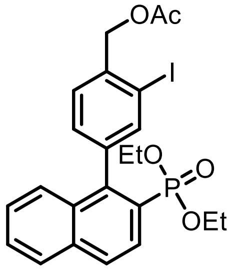

1u

4-(2-(diethoxyphosphoryl)naphthalen-1-yl)-2-iodobenzyl acetate (1u)

Physical state: colorless oil.

Yield: 0.51 g, 47%.

^{1}H NMR (500 MHz, Chloroform-d) δ 8.08 (dd, *J* = 12.2, 8.6 Hz, 1H), 7.95 (dd, *J* = 8.5, 3.6 Hz, 1H), 7.89 (d, *J* = 8.1 Hz, 1H), 7.86 (d, *J* = 1.4 Hz, 1H), 7.59 – 7.54 (m, 1H), 7.48 (d, *J* = 7.8 Hz, 1H), 7.42 (d, *J* = 6.5 Hz, 2H), 7.39 – 7.36 (m, 1H), 5.33 – 5.19 (m, 2H), 4.03 – 3.81 (m, 4H), 2.19 (d, *J* = 1.2 Hz, 3H), 1.25 (t, *J* = 7.0 Hz, 3H), 1.15 (t, *J* = 7.1 Hz, 3H).

^{13}C NMR (126 MHz, CDCl3) δ 170.53, 142.67 (d, *J* = 9.7 Hz), 141.05, 140.00 (d, *J* = 5.4 Hz), 137.56, 134.92 (d, *J* = 2.6 Hz), 132.47 (d, *J* = 15.8 Hz), 130.64, 128.15 (d, *J* = 10.0 Hz), 128.00, 127.89, 127.86, 127.75, 127.23, 126.80, 124.91 (d, *J* = 187.3 Hz), 96.95,

69.83, 61.88 (d, J = 5.9 Hz), 61.74 (d, J = 5.9 Hz), 20.90, 16.26 (d, J = 6.8 Hz), 16.12 (d, J = 6.8 Hz).

^{31}P NMR (202 MHz, Chloroform-*d*) δ +17.78.

HRMS (ESI-TOF): calcd for $C_{23}H_{25}IO_5P^+$ [M+H$^+$] 539.0479, found 539.0481.

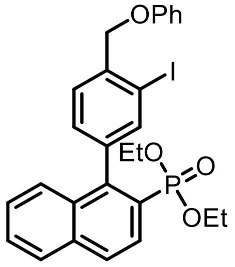

1v

diethyl (1-(3-iodo-4-(phenoxymethyl)phenyl)naphthalen-2-yl)phosphonate (1v)

Physical state: yellow oil.

Yield: 0.61 g, 53%.

^{1}H NMR (500 MHz, Chloroform-d) δ 8.15 -8.11 (m, 1H), 7.97 - 7.95 (m, 1H), 7.91-7.89 (m, 2H), 7.65 (d, J = 7.8 Hz, 1H), 7.57 (t, J = 7.4 Hz, 1H), 7.49-7.48 (m, 1H), 7.43 (d, J = 7.4 Hz, 1H), 7.41 – 7.39 (m, 1H), 7.34 (t, J = 7.8 Hz, 2H), 7.06 (d, J = 8.1 Hz, 1H), 7.01 (t, J = 7.4 Hz, 1H), 5.17 (s, 2H), 4.05 – 3.83 (m, 4H), 1.27 (t, J = 7.1 Hz, 3H), 1.15 (t, J = 7.1 Hz, 3H).

^{13}C NMR (126 MHz, CDCl3) δ 158.27, 142.84 (d, J = 9.6 Hz), 140.75, 139.52 (d, J = 5.5 Hz), 138.44, 134.90 (d, J = 2.5 Hz), 132.49 (d, J = 15.8 Hz), 130.61, 129.45, 128.20 (d, J = 10.0 Hz), 127.84, 127.77, 127.66, 127.29, 127.25, 126.73, 124.82 (d, J = 188.0 Hz), 95.85, 73.57, 61.82 (d, J = 5.9 Hz), 61.71 (d, J = 6.0 Hz), 16.25 (d, J = 6.9 Hz), 16.09 (d, J = 6.8 Hz).

^{31}P NMR (202 MHz, Chloroform-*d*) δ +17.93.

HRMS (ESI-TOF): calcd for $C_{27}H_{27}IO_4P^+$ [M+H$^+$] 573.0686, found 573.0684.

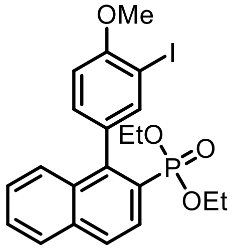

1w

diethyl (1-(3-iodo-4-methoxyphenyl)naphthalen-2-yl)phosphonate (1w)

Physical state: colorless oil.

Yield: 0.53 g, 54%.

^{1}H NMR (500 MHz, Chloroform-d) δ 8.08 (dd, J = 12.2, 8.6 Hz, 1H), 7.92 (dd, J = 8.6, 3.7 Hz, 1H), 7.88 (d, J = 8.2 Hz, 1H), 7.75 (d, J = 2.0 Hz, 1H), 7.54 (t, J = 7.4 Hz, 1H), 7.48 (d, J = 8.5 Hz, 1H), 7.41 (d, J = 15.2 Hz, 1H), 7.33 (dd, J = 8.3, 2.0 Hz, 1H), 6.93 (d, J = 8.4 Hz, 1H), 4.08 – 3.97 (m, 2H), 3.96 (s, 3H), 3.93 – 3.81 (m, 2H), 1.27 (t, J = 7.1 Hz, 3H), 1.17 (t, J = 7.1 Hz, 3H).

^{13}C NMR (126 MHz, Chloroform-d) δ 157.54, 143.30 (d, *J* = 9.8 Hz), 141.06, 134.95 (d, *J* = 2.4 Hz), 132.99 (d, *J* = 15.8 Hz), 132.26 (d, *J* = 5.6 Hz), 131.83, 128.20 (d, *J* = 10.1 Hz), 127.79 (d, *J* = 5.9 Hz), 127.55, 127.44, 127.32, 126.63, 125.06 (d, *J* = 187.5 Hz), 109.52, 84.64, 61.83 (d, *J* = 5.9 Hz), 61.66 (d, *J* = 5.9 Hz), 56.32, 16.29 (d, *J* = 6.9 Hz), 16.17 (d, *J* = 6.9 Hz).
^{31}P NMR (202 MHz, Chloroform-*d*) δ +18.11.
HRMS (ESI-TOF): calcd for $C_{21}H_{23}IO_4P^+$ [M+H$^+$] 497.0373, found 497.0367.

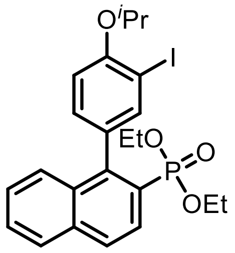

1x

diethyl (1-(3-iodo-4-isopropoxyphenyl)naphthalen-2-yl)phosphonate (1x)
Physical state: yellow solid.
Yield: 0.64 g, 61%.
^{1}H NMR (500 MHz, Chloroform-d) δ 8.08 (dd, *J* = 12.3, 8.5 Hz, 1H), 7.95 – 7.84 (m, 2H), 7.75 (d, *J* = 2.1 Hz, 1H), 7.59 – 7.47 (m, 2H), 7.44 – 7.37 (m, 1H), 7.31 – 7.26 (m, 1H), 4.71 – 4.62 (m, 1H), 4.08 – 3.76 (m, 4H), 1.50 – 1.40 (m, 6H), 1.27 (t, *J* = 7.1 Hz, 3H), 1.16 (t, *J* = 7.1 Hz, 3H).
^{13}C NMR (126 MHz, Chloroform-d) δ 156.21, 143.39 (d, *J* = 9.9 Hz), 141.13, 134.94 (d, *J* = 2.7 Hz), 132.96 (d, *J* = 15.8 Hz), 132.13 (d, *J* = 5.5 Hz), 131.58, 127.75 (d, *J* = 5.6 Hz), 127.47, 127.37, 126.56, 125.00 (d, *J* = 187.7 Hz), 87.10, 72.00, 61.78 (d, *J* = 5.9 Hz), 61.60 (d, *J* = 6.0 Hz), 22.09, 22.03, 16.26 (d, *J* = 6.9 Hz), 16.12 (d, *J* = 6.9 Hz).
^{31}P NMR (202 MHz, Chloroform-*d*) δ +18.22.
HRMS (ESI-TOF): calcd for $C_{23}H_{26}IO_4P^+$ [M+H$^+$] 525.0686, found 525.0684.

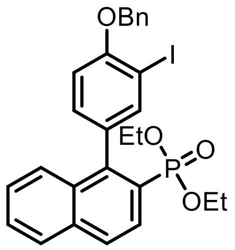

1y

diethyl (1-(4-(benzyloxy)-3-iodophenyl)naphthalen-2-yl)phosphonate (1y)
Physical state: white solid.
Yield: 0.45 g, 40%.
^{1}H NMR (500 MHz, Chloroform-d) δ 8.15 – 8.08 (m, 1H), 7.93 (dd, *J* = 8.5, 3.2 Hz, 1H), 7.88 (d, *J* = 8.3 Hz, 1H), 7.80 (s, 1H), 7.62 – 7.49 (m, 4H), 7.42 (t, *J* = 6.3 Hz, 3H), 7.37 – 7.29 (m, 2H), 6.98 (d, *J* = 8.3 Hz, 1H), 5.25 (s, 2H), 4.06 – 3.94 (m, 2H), 3.92 – 3.80 (m, 2H), 1.28 (t, *J* = 7.0 Hz, 3H), 1.15 (t, *J* = 7.0 Hz, 3H).

13C NMR (126 MHz, CDCl3) δ 156.55, 143.20 (d, *J* = 9.8 Hz), 141.05, 136.28, 134.88 (d, *J* = 2.4 Hz), 132.86 (d, *J* = 15.8 Hz), 132.47 (d, *J* = 5.5 Hz), 131.63, 128.42, 128.14 (d, *J* = 10.3 Hz), 127.77, 127.75, 127.72, 127.45 (d, *J* = 14.3 Hz), 127.26, 126.88, 126.58, 124.94 (d, *J* = 187.8 Hz), 85.41, 70.68, 61.78 (d, *J* = 5.9 Hz), 61.61 (d, *J* = 6.0 Hz), 16.23 (d, *J* = 6.9 Hz), 16.08 (d, *J* = 6.9 Hz).
^{31}P NMR (202 MHz, Chloroform-*d*) δ +18.13.
HRMS (ESI-TOF): calcd for $C_{27}H_{27}IO_4P^+$ [M+H$^+$] 573.0686, found 573.0688.

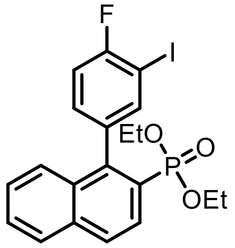

1z

diethyl (1-(4-fluoro-3-iodophenyl)naphthalen-2-yl)phosphonate (1z)
Physical state: yellow solid.
Yield: 0.65 g, 67%.
^{1}H NMR (500 MHz, Chloroform-d) δ 8.11 – 8.07 (m, 1H), 7.97 – 7.95 (m, 1H), 7.90 (d, *J* = 8.2 Hz, 1H), 7.76 – 7.74 (m, 1H), 7.59 – 7.56 (m, 1H), 7.46 – 7.41 (m, 2H), 7.34 – 7.31 (m, 1H), 7.17 (t, *J* = 8.1 Hz, 1H), 4.06 – 3.81 (m, 4H), 2.53 (s, 3H), 1.27 (t, *J* = 7.1 Hz, 3H), 1.18 (t, *J* = 7.1 Hz, 3H).
^{13}C NMR (126 MHz, Chloroform-d) δ 161.32 (d, *J* = 246.6 Hz), 142.01 (d, *J* = 9.5 Hz), 141.86, 138.25 (d, *J* = 5.5 Hz), 141.15, 136.01 (d, *J* = 5.6 Hz), 135.98 (d, *J* = 5.3 Hz), 134.99 (d, *J* = 2.7 Hz), 132.68 (d, *J* = 15.8 Hz), 132.44 (d, *J* = 7.4 Hz), 128.19 (d, *J* = 10.0 Hz), 127.96 (d, *J* = 3.8 Hz), 127.85, 127.00 (d, *J* = 23.9 Hz), 125.19 (d, *J* = 187.8 Hz), 80.14 (d, *J* = 25.8 Hz), 61.94 (d, *J* = 6.0 Hz), 61.79 (d, *J* = 6.0 Hz), 16.33 (d, *J* = 6.9 Hz), 16.21 (d, *J* = 6.8 Hz).
^{31}P NMR (202 MHz, Chloroform-*d*) δ +17.83.
^{19}F NMR (471 MHz, Chloroform-*d*) δ -95.43.
HRMS (ESI-TOF): calcd for $C_{20}H_{19}IO_3P^+$ [M+H$^+$] 485.0173, found 485.0176.

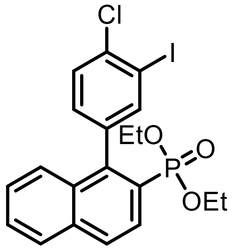

1aa

diethyl (1-(4-chloro-3-iodophenyl)naphthalen-2-yl)phosphonate (1aa)
Physical state: yellow solid.
Yield: 0.57 g, 57%.
^{1}H NMR (500 MHz, Chloroform-d) δ 8.11 – 8.07 (m, 1H), 7.97 – 7.94 (m, 1H), 7.89 – 7.81 (m, 1H), 7.80 (d, *J* = 8.3 Hz, 1H), 7.56 – 7.52 (m, 1H), 7.47 (d, *J* = 8.4 Hz, 1H), 7.42 – 7.38

(m, 1H), 7.34 (d, J = 7.7 Hz, 1H), 7.27 – 7.24 (m, 1H), 4.06 – 3.81 (m, 4H), 2.53 (s, 3H), 1.27 (t, J = 7.1 Hz, 3H), 1.18 (t, J = 7.1 Hz, 3H).

^{13}C NMR (126 MHz, Chloroform-d) δ 142.01 (d, J = 9.5 Hz), 141.86, 138.25 (d, J = 5.5 Hz), 137.85, 134.92 (d, J = 2.6 Hz), 132.38 (d, J = 15.6 Hz), 131.77, 128.13 (d, J = 9.9 Hz), 128.04, 128.01, 127.95, 127.89, 127.00, 126.92, 124.99 (d, J = 187.8 Hz), 96.94, 61.95 (d, J = 6.0 Hz), 61.80 (d, J = 6.0 Hz), 16.29 (d, J = 6.9 Hz), 16.16 (d, J = 6.8 Hz).

^{31}P NMR (202 MHz, Chloroform-*d*) δ +17.71.

HRMS (ESI-TOF): calcd for $C_{20}H_{19}IO_3P^+$ [M+H$^+$] 500.9878, found 500.9873.

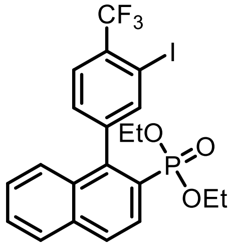

1ab

diethyl (1-(3-iodo-4-(trifluoromethyl)phenyl)naphthalen-2-yl)phosphonate (1ab)

Physical state: yellow solid.

Yield: 0.55 g, 52%.

1H NMR (500 MHz, Chloroform-d) δ 8.13 – 8.06 (m, 1H), 8.03 (s, 1H), 7.99 – 7.94 (m, 1H), 7.89 (d, J = 8.3 Hz, 1H), 7.75 (d, J = 8.1 Hz, 1H), 7.55 (t, J = 7.6 Hz, 1H), 7.46 (d, J = 8.0 Hz, 1H), 7.42 (t, J = 7.7 Hz, 1H), 7.35 (d, J = 8.6 Hz, 1H), 4.05 – 3.83 (m, 4H), 1.24 (t, J = 7.1 Hz, 3H), 1.15 (t, J = 7.1 Hz, 3H).

13C NMR (126 MHz, Chloroform-d) δ 143.62, 143.43 (d, J = 5.5 Hz), 141.40 (d, J = 9.4 Hz), 134.81 (d, J = 2.6 Hz), 132.61 (q, J = 31.2 Hz), 131.95 (d, J = 15.5 Hz), 130.22, 128.16 (d, J = 14.4 Hz), 128.01, 127.97, 127.95, 127.93, 127.03, 126.72, 126.30 (q, J = 5.5 Hz), 124.93 (d, J = 187.5 Hz), 122.78 (d, J = 273.7 Hz), 89.77 (d, J = 2.2 Hz), 61.94 (d, J = 6.2 Hz), 61.75 (d, J = 6.0 Hz), 16.16 (d, J = 6.8 Hz), 16.02 (d, J = 6.7 Hz).

^{31}P NMR (202 MHz, Chloroform-*d*) δ +17.46.

^{19}F NMR (471 MHz, Chloroform-*d*) δ -62.42.

HRMS (ESI-TOF): calcd for $C_{21}H_{20}F_3IO_3P^+$ [M+H$^+$] 535.0141, found 535.0145.

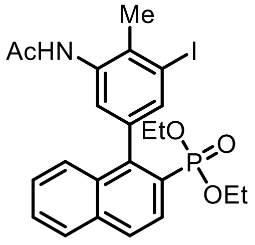

1ac

diethyl (1-(3-acetamido-5-iodo-4-methylphenyl)naphthalen-2-yl)phosphonate (1ac)

Physical state: light yellow solid.

Yield: 0.52 g, 49%.

^{1}H NMR (500 MHz, Chloroform-d) δ 8.04 -8.00 (m, 1H), 7.93 - 7.91 (m, 1H), 7.88 (d, J = 8.2 Hz, 1H), 7.70 (s, 1H), 7.62 (s, 1H), 7.58 – 7.54 (m, 2H), 7.45 – 7.40 (m, 1H), 4.08 –

3.96 (m, 2H), 3.94 – 3.81 (m, 2H), 2.49 (s, 3H), 2.15 (s, 3H), 2.04 (d, *J* = 2.9 Hz, 1H), 1.28 (t, *J* = 7.0 Hz, 3H), 1.16 (t, *J* = 7.1 Hz, 3H).
^{13}C NMR (126 MHz, CDCl3) δ 158.30, 142.84 (d, *J* = 10.0 Hz), 140.76, 139.53 (d, *J* = 5.4 Hz), 138.44, 134.91, 132.50 (d, *J* = 15.8 Hz), 130.62, 129.46, 128.20 (d, *J* = 10.0 Hz), 127.85, 127.66, 127.29, 126.74, 124.82 (d, *J* = 188.3 Hz), 121.16, 114.85, 95.85, 73.57, 61.82 (d, *J* = 5.7 Hz), 61.72 (d, *J* = 5.8 Hz), 16.25 (d, *J* = 6.8 Hz), 16.09 (d, *J* = 6.7 Hz).
^{31}P NMR (202 MHz, Chloroform-*d*) δ +17.95.
HRMS (ESI-TOF): calcd for $C_{23}H_{26}INO_4P^+$ [M+H$^+$] 538.0639, found 538.0642.

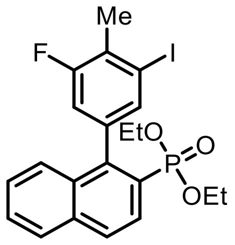

1ad

diethyl (1-(3-fluoro-5-iodo-4-methylphenyl)naphthalen-2-yl)phosphonate (1ad)
Physical state: yellow solid.
Yield: 0.54 g, 54%.
^{1}H NMR (500 MHz, Chloroform-d) δ 8.12 - 8.08 (m, 1H), 7.96 - 7.89 (m, 1H), 7.89 (d, *J* = 8.1 Hz, 1H), 7.61 (s, 1H), 7.58 – 7.55 (m, 1H), 7.48 – 7.42 (m, 2H), 7.06 (dd, *J* = 9.7, 1.6 Hz, 1H), 4.05 – 3.90 (m, 4H), 2.47 (d, *J* = 2.4 Hz, 3H), 1.25 (t, *J* = 7.1 Hz, 3H), 1.21 (t, *J* = 7.1 Hz, 3H).
^{13}C NMR (126 MHz, CDCl3) δ 158.72 (d, *J* = 249.7 Hz), 141.88 (dd, *J* = 9.3, 1.8 Hz), 138.76 (dd, *J* = 9.0, 5.5 Hz), 136.21 (d, *J* = 3.5 Hz), 134.87 (d, *J* = 2.6 Hz), 132.30 (d, *J* = 15.6 Hz), 128.20 (d, *J* = 10.0 Hz), 128.07 (d, *J* = 17.9 Hz), 127.89, 127.86, 127.77, 126.90 (d, *J* = 24.6 Hz), 124.80 (d, *J* = 187.3 Hz), 124.80 (d, *J* = 186.9 Hz), 117.64 (d, *J* = 24.3 Hz), 100.62 (d, *J* = 4.1 Hz), 61.79 (d, *J* = 5.8 Hz), 61.72 (d, *J* = 6.0 Hz), 19.83 (d, *J* = 3.6 Hz), 16.19 (d, *J* = 7.1 Hz), 16.05 (d, *J* = 6.9 Hz).
^{31}P NMR (202 MHz, Chloroform-*d*) δ +17.78.
^{19}F NMR (471 MHz, Chloroform-*d*) δ -111.16.
HRMS (ESI-TOF): calcd for $C_{23}H_{24}IO_5P^+$ [M+H$^+$] 499.0330, found 499.0332.

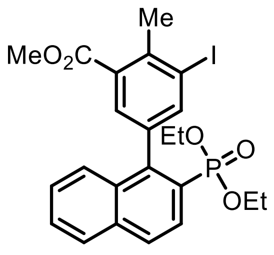

1ae

methyl 5-(2-(diethoxyphosphoryl)naphthalen-1-yl)-3-iodo-2-methylbenzoate (1ae)
Physical state: yellow solid.
Yield: 0.79 g, 73%.

^{1}H NMR (500 MHz, Chloroform-d) δ 8.14 -8.10 (m, 1H), 7.99 (d, *J* = 1.8 Hz, 1H), 7.96 (dd, *J* = 8.6, 3.7 Hz, 1H), 7.90 (d, *J* = 8.2 Hz, 1H), 7.79 (d, *J* = 1.9 Hz, 1H), 7.58 – 7.55 (m, 1H), 7.45 – 7.41 (m, 1H), 4.02 – 3.87 (m, 4H), 3.85 (s, 3H), 2.79 (s, 3H), 1.23 (t, *J* = 7.0 Hz, 3H), 1.17 (t, *J* = 7.1 Hz, 3H).
^{13}C NMR (126 MHz, CDCl3) δ 167.58, 144.11, 142.03 (d, *J* = 9.5 Hz), 141.05, 137.24 (d, *J* = 5.6 Hz), 134.99 (d, *J* = 2.5 Hz), 132.50 (d, *J* = 15.5 Hz), 132.29, 130.44, 128.38 (d, *J* = 10.1 Hz), 127.96, 127.94, 127.85, 127.12, 126.92, 125.03 (d, *J* = 187.2 Hz), 103.02, 61.87 (d, *J* = 5.8 Hz), 61.79 (d, *J* = 5.8 Hz), 52.20, 26.33, 16.26 (d, *J* = 7.1 Hz), 15.98 (d, *J* = 7.0 Hz).
^{31}P NMR (202 MHz, Chloroform-*d*) δ +17.81.
HRMS (ESI-TOF): calcd for $C_{23}H_{25}IO_5P^+$ [M+H$^+$] 539.0479, found 539.0482.

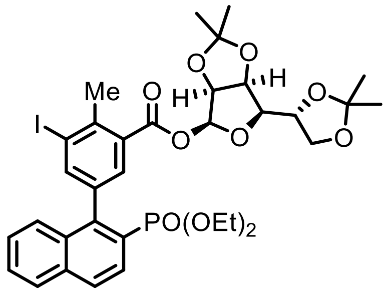

1af

(3a*S*,4*S*,6*R*,6a*S*)-6-(*(R)*-2,2-dimethyl-1,3-dioxolan-4-yl)-2,2-dimethyltetrahydrofuro[3,4-d][1,3]dioxol-4-yl 5-(2-(diethoxyphosphoryl)naphthalen-1-yl)-3-iodo-2-methylbenzoate (1af)
Physical state: white solid.
Yield: 0.47 g, 37%.
^{1}H NMR (500 MHz, Chloroform-d) δ 8.15 – 8.09 (m, 1H), 8.02 (dd, *J* = 4.0, 1.8 Hz, 1H), 7.99 – 7.96 (m, 1H), 7.91 (d, *J* = 8.2 Hz, 1H), 7.74 – 7.71 (m, 1H), 7.60 – 7.56 (m, 1H), 7.46 – 7.40 (m, 2H), 6.32 (d, *J* = 2.4 Hz, 1H), 4.83 – 4.80 (m, 1H), 4.78 – 4.75 (m, 1H), 4.40 – 4.36 (m, 1H), 4.08 – 4.05 (m, 1H), 4.02 – 3.92 (m, 5H), 3.60 – 3.53 (m, 1H), 2.79 (s, 3H), 1.48 (s, 3H), 1.36 (d, *J* = 5.4 Hz, 3H), 1.33 (s, 3H), 1.31 (d, *J* = 3.7 Hz, 3H), 1.25 – 1.22 (m, 3H), 1.16 (t, *J* = 7.1 Hz, 3H).
^{13}C NMR (126 MHz, Chloroform-d) δ 165.52, 144.80 (d, *J* = 5.1 Hz), 141.58 (d, *J* = 2.5 Hz), 137.42 (d, *J* = 5.4 Hz), 135.10, 132.57 (d, *J* = 15.9 Hz), 132.24 (d, *J* = 5.3 Hz), 129.72 (d, *J* = 5.2 Hz), 128.19, 128.11, 127.19, 125.17 (d, *J* = 187.0 Hz), 113.42, 109.35, 103.32 (d, *J* = 1.8 Hz), 101.96 (d, *J* = 6.4 Hz), 85.16, 82.69 (d, *J* = 6.8 Hz), 79.32, 72.82, 66.74 (d, *J* = 5.9 Hz), 62.00 (d, *J* = 5.9 Hz), 61.91 (d, *J* = 5.8 Hz), 33.98, 26.91, 26.58, 25.97, 25.10, 24.66, 16.38 (d, *J* = 7.1 Hz), 16.23 (d, *J* = 6.8 Hz).
^{31}P NMR (202 MHz, Chloroform-*d*) δ +17.79.
HRMS (ESI-TOF): calcd for $C_{34}H_{41}IO_{10}P^+$ [M+H$^+$] 767.1477, found 767.1475.

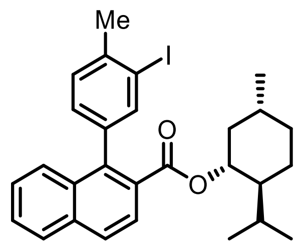

1ag

(1*R*,2*S*,5*R*)-2-isopropyl-5-methylcyclohexyl 1-(3-iodo-4-methylphenyl)-2-naphthoate (1ag)

Physical state: white solid.

Yield: 0.38 g, 75%.

^{1}H NMR (500 MHz, Chloroform-*d*) δ 7.97 – 7.86 (m, 3H), 7.79 (d, *J* = 36.0 Hz, 1H), 7.62 – 7.52 (m, 2H), 7.44 (t, *J* = 7.8 Hz, 1H), 7.35 (t, *J* = 7.3 Hz, 1H), 7.23 (dd, *J* = 23.9, 7.7 Hz, 1H), 4.81 – 4.71 (m, 1H), 2.55 (s, 3H), 1.92 – 1.78 (m, 1H), 1.71 – 1.59 (m, 3H), 1.48 – 1.40 (m, 1H), 1.11 – 0.95 (m, 2H), 0.95 – 0.85 (m, 5H), 0.82 (d, *J* = 7.7 Hz, 2H), 0.77 – 0.69 (m, 3H), 0.67 – 0.60 (m, 1H).

^{13}C NMR (126 MHz, Chloroform-*d*) δ 167.96, 140.23, 139.76, 138.58, 129.97, 129.11, 128.89, 128.01, 127.96, 127.57, 127.45, 127.35, 126.77, 125.61, 125.22, 75.09, 46.79, 40.45, 34.27, 31.32, 27.98, 26.20, 23.26, 22.23, 21.06, 16.20.

HRMS (ESI-TOF): calcd for $C_{28}H_{32}IO_2^+$ [M+H$^+$] 527.1441, found 527.1438.

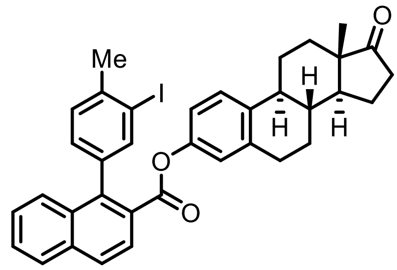

1ah

(8*R*,9*S*,13*S*,14*S*)-13-methyl-17-oxo-7,8,9,11,12,13,14,15,16,17-decahydro-6H-cyclopenta[a]phenanthren-3-yl 1-(3-iodo-4-methylphenyl)-2-naphthoate (1ah)

Physical state: yellow solid.

Yield: 0.35 g, 55%.

^{1}H NMR (500 MHz, Chloroform-*d*) δ 8.06 (d, *J* = 8.6 Hz, 1H), 7.98 (d, *J* = 8.6 Hz, 1H), 7.94 (d, *J* = 8.2 Hz, 1H), 7.88 (s, 1H), 7.66 (d, *J* = 8.5 Hz, 1H), 7.60 (t, *J* = 7.4 Hz, 1H), 7.48 (t, *J* = 7.6 Hz, 1H), 7.36 (dd, *J* = 7.7, 2.3 Hz, 1H), 7.32 – 7.29 (m, 1H), 7.25 (d, *J* = 8.8 Hz, 1H), 6.74 (d, *J* = 8.4 Hz, 1H), 6.56 (s, 1H), 2.93 – 2.87 (m, 2H), 2.55 – 2.53 (m, 3H), 2.53 – 2.48 (m, 1H), 2.42 – 2.37 (m, 1H), 2.29 – 2.23 (m, 1H), 2.19 – 2.12 (m, 1H), 2.08 – 1.96 (m, 3H), 1.66 – 1.58 (m, 2H), 1.54 – 1.42 (m, 4H), 0.91 (s, 3H).

^{13}C NMR (126 MHz, Chloroform-*d*) δ 166.94, 148.45, 140.43, 139.77, 139.51, 138.04, 137.82, 137.28, 134.81, 132.30, 129.75, 129.16, 128.14, 127.95, 127.74, 127.69, 127.50, 126.89, 126.20, 125.43, 121.37, 118.59, 100.72, 50.28, 47.83, 44.02, 37.87, 35.76, 31.43, 29.33, 27.87, 26.23, 25.65, 21.49, 13.72.

HRMS (ESI-TOF): calcd for $C_{36}H_{34}IO_3^+$ [M+H⁺] 641.1547, found 641.1543.

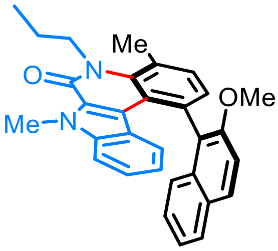

3h

***(R)*-1-(2-methoxynaphthalen-1-yl)-4,7-dimethyl-5-propyl-5,7-dihydro-6H-indolo[2,3-c]quinolin-6-one (3h)**

Physical state: yellow solid.

Yield: *(R)*-**3e**, 29.0 mg, 63%.

^{1}H NMR (500 MHz, Chloroform-d) δ 7.95 (d, *J* = 9.0 Hz, 1H), 7.90 – 7.87 (m, 1H), 7.85 – 7.82 (m, 1H), 7.37 – 7.33 (m, 2H), 7.31 (d, *J* = 7.6 Hz, 1H), 7.28 (d, *J* = 8.3 Hz, 1H), 7.22 (dd, *J* = 11.0, 8.3 Hz, 2H), 7.12 (t, *J* = 7.6 Hz, 1H), 6.34 (t, *J* = 7.7 Hz, 1H), 5.80 (d, *J* = 8.5 Hz, 1H), 4.54 (t, *J* = 7.2 Hz, 2H), 4.35 (s, 3H), 3.34 (s, 3H), 2.72 (s, 3H), 1.78 – 1.71 (m, 2H), 0.80 (t, *J* = 7.4 Hz, 3H).

^{13}C NMR (126 MHz, Chloroform-d) δ 159.88, 154.44, 140.54, 137.70, 134.03, 129.69, 129.68, 129.66, 129.40, 128.47, 128.08, 127.56, 127.08, 126.79, 125.82, 125.52, 124.86, 124.38, 123.82, 123.58, 121.76, 119.79, 119.42, 114.24, 109.28, 56.28, 49.56, 31.80, 23.57, 22.00, 11.14.

HPLC: *(R)*-**3e** 37% ee, Chiralpak IC-3 column, hexane : isopropanol = 95 : 5, 0.5 mL/min, λ = 254 nm, t_R (major) = 40.40 min, t_R (minor) = 43.13 min.

HRMS (ESI-TOF): calcd for $C_{31}H_{29}N_2O_2^+$ [M+H⁺] 461.2224, found 461.2222.

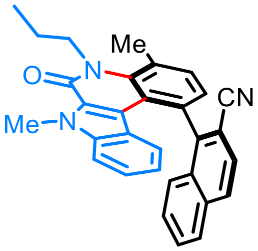

3i

(*R*)-1-(4,7-dimethyl-6-oxo-5-propyl-6,7-dihydro-5H-indolo[2,3-c]quinolin-1-yl)-2-naphthonitrile (3i)

Physical state: yellow solid.

Yield: *(R)*-**3i**, 29.6 mg, 65%.

^{1}H NMR (500 MHz, Chloroform-*d*) δ 7.98 (d, *J* = 8.6 Hz, 1H), 7.94 (d, *J* = 8.2 Hz, 1H), 7.89 (d, *J* = 8.6 Hz, 1H), 7.63 (d, *J* = 8.5 Hz, 1H), 7.58 (t, *J* = 7.5 Hz, 1H), 7.42 (t, *J* = 7.7 Hz, 1H), 7.34 (d, *J* = 7.7 Hz, 1H), 7.29 (d, *J* = 8.4 Hz, 1H), 7.24 (d, *J* = 7.6 Hz, 1H), 7.10 (t, *J* = 7.6 Hz, 1H), 6.30 (t, *J* = 7.7 Hz, 1H), 5.51 (d, *J* = 8.5 Hz, 1H), 4.62 – 4.55 (m, 1H), 4.50 – 4.43 (m, 1H), 4.34 (s, 3H), 2.73 (s, 3H), 1.75 – 1.67 (m, 2H), 0.82 (t, *J* = 7.4 Hz, 3H).

^{13}C NMR (126 MHz, Chloroform-*d*) δ 159.49, 147.71, 140.39, 138.03, 135.23, 132.41, 129.39, 129.35, 128.82, 128.72, 128.33, 127.89, 127.85, 127.47, 127.27, 127.23, 124.87, 122.89, 121.35, 119.74, 118.62, 117.99, 111.36, 109.81, 49.53, 31.82, 23.61, 21.96, 11.23.
HPLC: *(R)*-**3i** 90% ee, Chiralpak AD-H column, hexane : isopropanol = 90 : 10, 0.5 mL/min, λ = 254 nm, t_R (major) = 28.81 min, t_R (minor) = 27.14 min.
HRMS (ESI-TOF): calcd for $C_{31}H_{26}N_3O^+$ [M+H$^+$] 456.2070, found 456.2072.

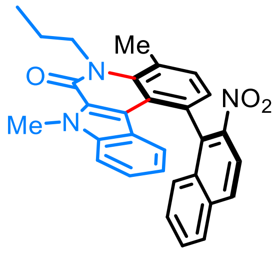

3j

(*R*)-4,7-dimethyl-1-(2-nitronaphthalen-1-yl)-5-propyl-5,7-dihydro-6H-indolo[2,3-c]quinolin-6-one (3i)
Physical state: white solid.
Yield: *(R)*-**3j**, 35.2 mg, 74%.
^{1}H NMR (500 MHz, Chloroform-*d*) δ 8.06 (d, *J* = 8.9 Hz, 1H), 7.97 (d, *J* = 8.9 Hz, 1H), 7.90 (d, *J* = 8.2 Hz, 1H), 7.58 (d, *J* = 8.6 Hz, 1H), 7.49 (t, *J* = 7.5 Hz, 1H), 7.30 – 7.24 (m, 3H), 7.08 (t, *J* = 7.6 Hz, 1H), 7.04 (d, *J* = 7.6 Hz, 1H), 6.35 (t, *J* = 7.7 Hz, 1H), 5.69 (d, *J* = 8.6 Hz, 1H), 4.56 – 4.44 (m, 2H), 4.31 (s, 3H), 2.70 (s, 3H), 2.59 (s, 3H), 1.75 – 1.65 (m, 2H), 0.79 (t, *J* = 7.4 Hz, 3H).
^{13}C NMR (126 MHz, Chloroform-*d*) δ 159.51, 147.34, 140.38, 137.73, 136.86, 134.70, 132.76, 129.70, 129.36, 128.64, 128.23, 128.08, 128.02, 128.00, 127.93, 127.17, 126.09, 124.92, 122.89, 122.79, 121.37, 120.58, 119.80, 117.95, 109.67, 49.72, 40.85, 31.77, 23.48, 21.82, 11.09.
HPLC: *(R)*-**3j** 73% ee, Chiralpak AD-H column, hexane : isopropanol = 90 : 10, 1.0 mL/min, λ = 254 nm, t_R (major) = 13.82 min, t_R (minor) = 17.57 min.
HRMS (ESI-TOF): calcd for $C_{30}H_{26}N_3O_3^+$ [M+H$^+$] 476.1969, found 476.1967.

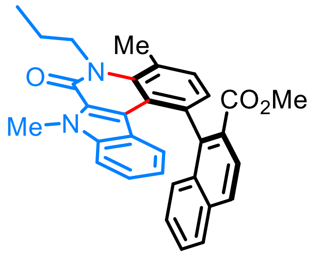

3k

methyl *(R)*-1-(4,7-dimethyl-6-oxo-5-propyl-6,7-dihydro-5H-indolo[2,3-c]quinolin-1-yl) -2-naphthoate (3k)
Physical state: yellow solid.
Yield: *(R)*-**3k**, 29.8 mg, 61%.
^{1}H NMR (500 MHz, Chloroform-d) δ 8.03 – 7.97 (m, 2H), 7.85 (d, *J* = 8.2 Hz, 1H), 7.55 (d, *J* = 8.6 Hz, 1H), 7.39 (t, *J* = 7.3 Hz, 1H), 7.27 (d, *J* = 7.6 Hz, 1H), 7.23 – 7.16 (m, 2H), 7.10

– 7.05 (m, 2H), 6.35 (t, J = 7.7 Hz, 1H), 5.65 (d, J = 8.6 Hz, 1H), 4.62 – 4.49 (m, 2H), 4.31 (s, 3H), 3.45 (s, 3H), 2.73 (s, 3H), 1.78 – 1.68 (m, 2H), 0.82 (t, J = 7.4 Hz, 3H).
^{13}C NMR (126 MHz, Chloroform-d) δ 168.73, 159.59, 143.07, 140.31, 137.33, 134.70, 132.86, 132.46, 129.35, 129.29, 128.08, 127.81, 127.68, 127.44, 127.35, 126.99, 126.75, 126.00, 125.83, 124.74, 123.81, 122.81, 121.61, 119.51, 118.63, 109.25, 51.82, 49.63, 31.67, 23.44, 21.79, 11.11.
HPLC: *(R)*-**3k** 80% ee, Chiralpak AD-H column, hexane : isopropanol = 80 : 20, 1.0 mL/min, λ = 254 nm, t_R (major) = 7.45 min, t_R (minor) = 8.27 min.
HRMS (ESI-TOF): calcd for $C_{32}H_{29}N_2O_3^+$ [M+H$^+$] 489.2173, found 489.2169.

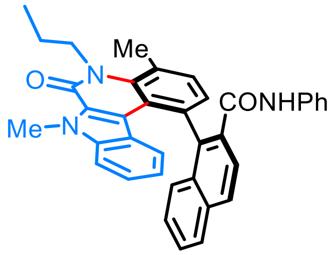

3l

(R)-1-(4,7-dimethyl-6-oxo-5-propyl-6,7-dihydro-5H-indolo[2,3-c]quinolin-1-yl)-N-phenyl-2-naphthamide (3l)
Physical state: yellow solid.
Yield: *(R)*-**3l**, 23.6 mg, 43%.
^{1}H NMR (500 MHz, Chloroform-d) δ 8.05 (d, J = 8.5 Hz, 1H), 7.96 (d, J = 8.2 Hz, 1H), 7.83 (d, J = 8.5 Hz, 1H), 7.79 (d, J = 8.5 Hz, 1H), 7.52 (t, J = 7.5 Hz, 1H), 7.38 (t, J = 7.5 Hz, 1H), 7.33 – 7.29 (m, 2H), 7.25 (d, J = 7.7 Hz, 1H), 7.16 (t, J = 7.6 Hz, 1H), 7.11 (t, J = 7.8 Hz, 2H), 6.96 (t, J = 7.4 Hz, 1H), 6.80 (d, J = 7.7 Hz, 2H), 6.75 (s, 1H), 6.40 (t, 1H), 5.56 (d, J = 8.6 Hz, 1H), 4.45 – 4.32 (m, 2H), 4.24 (s, 3H), 2.67 (s, 3H), 1.58 – 1.52 (m, 2H), 0.73 (t, J = 7.4 Hz, 3H).
^{13}C NMR (126 MHz, Chloroform-d) δ 166.94, 159.21, 140.30, 139.07, 137.84, 137.46, 134.49, 134.40, 132.94, 130.69, 129.79, 128.66, 128.51, 128.09, 128.03, 127.95, 127.29, 127.25, 127.13, 126.85, 125.26, 125.13, 123.96, 123.09, 122.60, 121.08, 120.20, 118.92, 117.65, 109.76, 49.73, 31.69, 23.42, 21.65, 11.12.
HPLC: *(R)*-**3l** 93% ee, Chiralpak AD-H column, hexane : isopropanol = 80 : 20, 1.0 mL/min, λ = 254 nm, t_R (major) = 19.59 min, t_R (minor) = 33.63 min.
HRMS (ESI-TOF): calcd for $C_{37}H_{32}N_3O_2$ [M+H$^+$] 550.2489, found 550.2486.

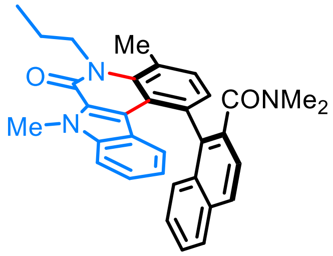

3m

(R)-1-(4,7-dimethyl-6-oxo-5-propyl-6,7-dihydro-5H-indolo[2,3-c]quinolin-1-yl)-N,N-dimethyl-2-naphthamide (3m)

Physical state: yellow oil.

Yield: *(R)*-**3m**, 18.5 mg, 37%.

^{1}H NMR (500 MHz, Chloroform-d) δ 7.96 (d, *J* = 8.4 Hz, 1H), 7.93 – 7.71 (m, 2H), 7.53 – 7.30 (m, 4H), 7.22 – 6.97 (m, 3H), 6.32 (s, 1H), 6.09 – 5.58 (m, 1H), 4.62 – 4.09 (m, 5H), 2.99 – 2.77 (m, 3H), 2.69 (s, 3H), 2.66 – 2.61 (m, 1H), 2.25 – 2.01 (m, 2H), 1.80 – 1.65 (m, 2H), 0.88 – 0.78 (m, 3H).

^{13}C NMR (126 MHz, Chloroform-d) δ 162.32, 159.27, 140.20, 137.78, 133.56, 129.02, 128.21, 127.99, 127.87, 127.23, 126.70, 126.49, 126.38, 126.23, 125.75, 124.78, 124.42, 124.18, 120.26, 119.59, 109.32, 49.32, 34.40, 31.62, 23.41, 11.16.

HPLC: *(R)*-**3m** 82% ee, Chiralpak AD-H column, hexane : isopropanol = 80 : 20, 1.0 mL/min, λ = 254 nm, t_R (major) = 27.11 min, t_R (minor) = 14.52 min.

HRMS (ESI-TOF): calcd for $C_{33}H_{32}N_3O_2^+$ [M+H$^+$] 502.2489, found 502.2493.

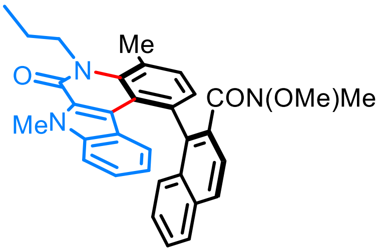

3n

***(R)*-1-(4,7-dimethyl-6-oxo-5-propyl-6,7-dihydro-5H-indolo[2,3-c]quinolin-1-yl)-N-methoxy-N-methyl-2-naphthamide (3n)**

Physical state: yellow oil.

Yield: *(R)*-**3n**, 18.1 mg, 35%.

^{1}H NMR (500 MHz, Chloroform-d) δ 8.02 (d, *J* = 7.7 Hz, 1H), 7.97 – 7.62 (m, 4H), 7.59 – 7.36 (m, 3H), 7.19 (s, 1H), 7.03 (t, *J* = 7.9 Hz, 1H), 6.30 (t, *J* = 7.7 Hz, 1H), 5.61 (d, *J* = 7.5 Hz, 1H), 4.48 (s, 2H), 4.30 (s, 3H), 3.23 (s, 3H), 3.05 (s, 2H), 2.69 (s, 3H), 1.80 – 1.60 (m, 3H), 0.77 (s, 3H).

^{13}C NMR (126 MHz, Chloroform-d) δ 172.01, 159.56, 140.29, 139.54, 137.56, 133.64, 132.76, 131.32, 129.12, 128.16, 127.84, 127.62, 127.48, 127.45, 126.57, 126.30, 124.99, 124.76, 124.24, 121.67, 119.33, 109.02, 61.37, 49.73, 31.75, 29.20, 23.44, 21.86, 11.14.

HPLC: *(R)*-**3n** 82% ee, Chiralpak AD-H column, hexane : isopropanol = 80 : 20, 1.0 mL/min, λ = 254 nm, t_R (major) = 20.47 min, t_R (minor) = 13.68 min.

HRMS (ESI-TOF): calcd for $C_{33}H_{32}N_3O_3^+$ [M+H$^+$] 518.2438, found 518.2437.

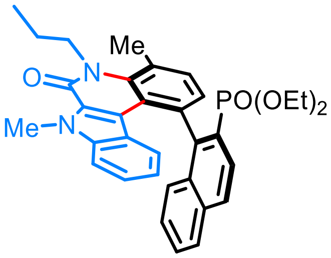

3p

diethyl *(R)*-(1-(4,7-dimethyl-6-oxo-5-propyl-6,7-dihydro-5H-indolo[2,3-c]quinolin-1-y l)naphthalen-2-yl)phosphonate (3p)

Physical state: white solid.
Yield: *(R)*-**3p**, 36.8 mg, 65%.
^{1}H NMR (500 MHz, Chloroform-d) δ 8.23 (dd, *J* = 12.2, 8.6 Hz, 1H), 8.09 (dd, *J* = 8.5, 3.6 Hz, 1H), 7.85 (d, *J* = 8.2 Hz, 1H), 7.40 – 7.36 (m, 1H), 7.28 – 7.18 (m, 4H), 7.12 – 7.08 (m, 1H), 7.06 – 7.02 (m, 1H), 6.31 – 6.26 (m, 1H), 5.24 – 5.19 (m, 1H), 4.62 – 4.55 (m, 1H), 4.45 – 4.38 (m, 1H), 4.30 (s, 3H), 3.81 – 3.73 (m, 2H), 3.63 – 3.52 (m, 2H), 2.71 (s, 3H), 1.67 – 1.61 (m, 2H), 0.95 (t, *J* = 7.1 Hz, 3H), 0.85 (t, *J* = 7.0 Hz, 3H), 0.76 (t, *J* = 7.4 Hz, 3H).
^{13}C NMR (126 MHz, Chloroform-d) δ 159.71, 147.28 (d, *J* = 9.4 Hz), 140.35, 137.04, 134.90 (d, *J* = 2.6 Hz), 133.84 (d, *J* = 15.7 Hz), 131.76 (d, *J* = 5.3 Hz), 129.00, 128.92, 128.66, 127.95, 127.84 (d, *J* = 15.1 Hz), 127.83, 127.34, 126.92, 126.78, 126.31 (d, *J* = 189.0 Hz), 124.79, 124.27, 123.57, 121.61, 119.59, 118.93, 109.21, 61.80 (d, *J* = 6.0 Hz), 61.53 (d, *J* = 6.0 Hz), 50.12, 31.86, 23.53, 21.65, 15.95 (d, *J* = 6.6 Hz), 15.82 (d, *J* = 6.6 Hz), 11.22.
^{31}P NMR (202 MHz, Chloroform-*d*) δ +17.97.
HPLC: *(R)*-**3p** 91% ee, Chiralpak OD-H column, hexane : isopropanol = 95 : 5, 0.5 mL/min, λ = 254 nm, t_R (major) = 37.13 min, t_R (minor) = 34.18 min.
HRMS (ESI-TOF): calcd for $C_{34}H_{35}N_2O_4P^+$ [M+H$^+$] 567.2407, found 567.2410.

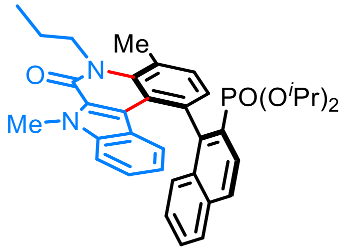

3q

diisopropyl *(R)*-(1-(4,7-dimethyl-6-oxo-5-propyl-6,7-dihydro-5H-indolo[2,3-c]quinolin-1-yl)naphthalen-2-yl)phosphonate (3q)
Physical state: yellow solid.
Yield: *(R)*-**3q**, 44.6 mg, 75%.
^{1}H NMR (500 MHz, Chloroform-d) δ 8.24 (dd, *J* = 12.4, 8.5 Hz, 1H), 8.07 (dd, *J* = 8.6, 3.5 Hz, 1H), 7.82 (d, *J* = 8.2 Hz, 1H), 7.34 (t, *J* = 7.5 Hz, 1H), 7.27 – 7.17 (m, 4H), 7.07 (t, *J* = 7.7 Hz, 1H), 7.02 (t, *J* = 7.7 Hz, 1H), 6.30 (t, *J* = 7.8 Hz, 1H), 5.31 (d, *J* = 8.7 Hz, 1H), 4.56 – 4.46 (m, 3H), 4.45 – 4.39 (m, 1H), 4.29 (s, 3H), 2.71 (s, 3H), 1.76 – 1.61 (m, 2H), 1.07 (d, *J* = 6.2 Hz, 3H), 0.95 (t, *J* = 5.0 Hz, 6H), 0.85 (d, *J* = 6.2 Hz, 3H), 0.81 (t, *J* = 7.4 Hz, 3H).
^{13}C NMR (126 MHz, Chloroform-d) δ 159.67, 147.02 (d, *J* = 9.2 Hz), 140.27, 136.96, 134.65 (d, *J* = 2.6 Hz), 133.64 (d, *J* = 15.7 Hz), 132.02 (d, *J* = 5.1 Hz), 129.09, 128.67 (d, *J* = 10.0 Hz), 128.54, 127.79, 127.72, 127.67, 127.61, 127.55 (d, *J* = 191.6 Hz), 127.23, 126.66, 126.58, 124.62, 124.46, 123.74, 121.77, 119.45, 119.19, 108.99, 70.33 (d, *J* = 6.0 Hz), 70.25 (d, *J* = 6.7 Hz), 50.17, 31.75, 23.84, 23.81, 23.49, 23.48 (q, *J* = 5.4 Hz), 23.43 (d, *J* = 5.5 Hz), 21.73, 11.27.
^{31}P NMR (202 MHz, Chloroform-*d*) δ +15.70.

HPLC: *(R)*-**3q** 93% ee, Chiralpak AD-H column, hexane : isopropanol = 80 : 20, 1.0 mL/min, λ = 254 nm, t_R (major) = 8.27 min, t_R (minor) = 11.04 min.
HRMS (ESI-TOF): calcd for $C_{36}H_{40}N_2O_4P^+$ [M+H$^+$] 595.2720, found 595.2723.

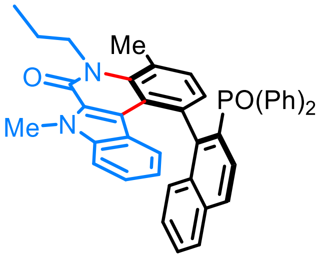

3r

***(R)*-1-(2-(diphenylphosphoryl)naphthalen-1-yl)-4,7-dimethyl-5-propyl-5,7-dihydro-6H-indolo[2,3-c]quinolin-6-one (3r)**
Physical state: yellow solid.
Yield: *(R)*-**3r**, 44.1 mg, 70%.
^{1}H NMR (500 MHz, Chloroform-d) δ 8.06 (d, *J* = 8.7 Hz, 1H), 7.89 (d, *J* = 8.4 Hz, 1H), 7.78 (dd, *J* = 11.6, 8.8 Hz, 1H), 7.46 – 7.41 (m, 4H), 7.34 (t, *J* = 7.4 Hz, 1H), 7.25 (d, *J* = 8.4 Hz, 1H), 7.21 – 7.00 (m, 9H), 6.75 – 6.69 (m, 2H), 6.41 (t, *J* = 7.7 Hz, 1H), 5.14 (d, *J* = 8.6 Hz, 1H), 4.56 – 4.48 (m, 1H), 4.28 (s, 3H), 4.12 – 4.07 (m, 1H), 2.56 (s, 3H), 1.70 – 1.62 (m, 2H), 0.76 (t, *J* = 7.4 Hz, 3H).
^{13}C NMR (126 MHz, Chloroform-d) δ 159.18, 147.77 (d, *J* = 8.6 Hz), 140.14, 137.04, 134.56 (d, *J* = 2.2 Hz), 134.27 (d, *J* = 11.0 Hz), 133.76, 132.93, 132.26 (d, *J* = 104.6 Hz), 131.65 (d, *J* = 9.2 Hz), 130.95 (d, *J* = 2.7 Hz), 130.79, 130.71, 130.37 (d, *J* = 97.7 Hz), 129.86 (d, *J* = 2.7 Hz), 129.06 (d, *J* = 12.1 Hz), 128.97, 128.68, 128.06, 127.84, 127.79, 127.74, 127.71, 127.65, 127.17, 127.03, 126.87, 126.76, 126.66, 124.73, 124.25, 123.13, 121.48, 119.56, 118.36, 109.45, 50.32, 31.70, 23.25, 21.71, 11.11.
^{31}P NMR (202 MHz, Chloroform-*d*) δ +27.63.
HPLC: *(R)*-**3r** 92% ee, Chiralpak IA-3 column, hexane : isopropanol = 70 : 30, 0.5 mL/min, λ = 254 nm, t_R (major) = 27.22 min, t_R (minor) = 21.15 min.
HRMS (ESI-TOF): calcd for $C_{42}H_{36}N_2O_2P^+$ [M+H$^+$] 631.2509, found 631.2512.

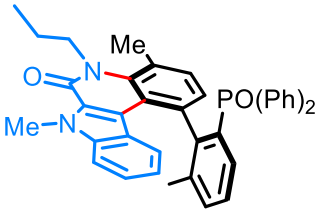

3s

***(R)*-1-(2-(diphenylphosphoryl)-6-methylphenyl)-4,7-dimethyl-5-propyl-5,7-dihydro-6H-indolo[2,3-c]quinolin-6-one (3s)**
Physical state: yellow solid.
Yield: *(R)*-**3r**, 36.2 mg, 61%.
^{1}H NMR (500 MHz, Chloroform-d) δ 7.63 (dd, *J* = 13.4, 7.7 Hz, 1H), 7.54 (dd, *J* = 11.2, 8.2 Hz, 2H), 7.50 – 7.46 (m, 1H), 7.44 – 7.31 (m, 4H), 7.26 – 7.22 (m, 2H), 7.11 – 7.04 (m, 4H), 6.95 (t, *J* = 7.4 Hz, 1H), 6.73 – 6.68 (m, 1H), 6.68 – 6.62 (m, 2H), 5.59 (d, *J* = 8.6 Hz, 1H),

4.50 – 4.44 (m, 1H), 4.34 (s, 3H), 4.08 – 4.00 (m, 1H), 2.50 (s, 3H), 1.79 (s, 3H), 1.60 – 1.51 (m, 2H), 0.65 (t, J = 7.4 Hz, 3H).

^{13}C NMR (126 MHz, Chloroform-d) δ 159.24, 147.75 (d, J = 8.8 Hz), 140.47, 140.26 (d, J = 9.5 Hz), 137.11, 134.15 (d, J = 102.0 Hz), 134.06 (d, J = 2.7 Hz), 132.93, 132.66 (d, J = 104.3 Hz), 132.49, 132.40, 132.15 (d, J = 4.3 Hz), 131.88, 131.81, 131.04 (d, J = 2.8 Hz), 130.85, 130.77, 129.91 (d, J = 2.8 Hz), 128.85, 128.07, 127.87, 127.77, 127.65, 126.74, 126.65, 126.46, 125.08, 124.60, 122.37, 122.03, 119.89, 118.84, 109.64, 50.26, 31.87, 23.18, 21.67, 20.23, 11.05.

^{31}P NMR (202 MHz, Chloroform-d) δ +27.11.

HPLC: *(R)*-**3s** 80% ee, Chiralpak IA-3 column, hexane : isopropanol = 70 : 30, 0.5 mL/min, λ = 254 nm, t_R (major) = 22.26 min, t_R (minor) = 17.61 min.

HRMS (ESI-TOF): calcd for $C_{39}H_{36}N_2O_2P^+$ [M+H$^+$] 595.2509, found 595.2512.

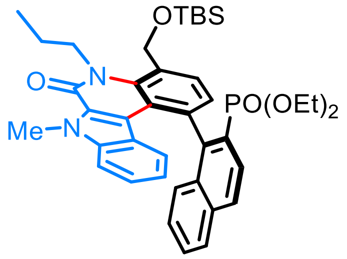

4a

diethyl *(R)*-(1-(4-(((tert-butyldimethylsilyl)oxy)methyl)-7-methyl-6-oxo-5-propyl-6,7-dihydro-5H-indolo[2,3-c]quinolin-1-yl)naphthalen-2-yl)phosphonate (4a)

Physical state: yellow oil.

Yield: *(R)*-**4a**, 40.0 mg, 56%.

^{1}H NMR (500 MHz, Chloroform-*d*) δ 8.25 (dd, J = 12.2, 8.6 Hz, 1H), 8.10 (dd, J = 8.6, 3.6 Hz, 1H), 7.86 (d, J = 8.2 Hz, 1H), 7.56 (d, J = 7.8 Hz, 1H), 7.39 (t, J = 7.4 Hz, 1H), 7.27 (d, J = 7.8 Hz, 1H), 7.24 (d, J = 8.7 Hz, 1H), 7.21 (d, J = 8.3 Hz, 1H), 7.12 – 7.09 (m, 1H), 7.04 (t, J = 7.6 Hz, 1H), 6.31 – 6.27 (m, 1H), 5.19 (d, J = 8.6 Hz, 1H), 5.00 (s, 2H), 4.67 – 4.61 (m, 1H), 4.45 – 4.40 (m, 1H), 4.30 (s, 3H), 3.79 – 3.73 (m, 2H), 3.65 – 3.60 (m, 1H), 3.51 – 3.46 (m, 1H), 1.68 – 1.62 (m, 2H), 0.98 – 0.94 (m, 12H), 0.79 (q, J = 7.1 Hz, 6H), 0.12 (d, J = 2.9 Hz, 6H).

^{13}C NMR (126 MHz, Chloroform-*d*) δ 159.21, 147.18 (d, J = 9.2 Hz), 140.39, 136.38, 134.97 (d, J = 2.7 Hz), 133.81 (d, J = 15.6 Hz), 133.08 (d, J = 5.4 Hz), 130.19, 129.10 (d, J = 10.1 Hz), 128.92, 128.07, 127.98, 127.96, 127.87, 127.41, 126.87, 126.74, 126.41 (d, J = 188.9 Hz), 124.91, 124.34, 123.19, 121.63, 119.72, 119.04, 109.30, 64.53, 61.85 (d, J = 6.0 Hz), 61.57 (d, J = 6.1 Hz), 51.16, 31.91, 25.90, 21.82, 18.32, 16.02 (d, J = 6.8 Hz), 15.81 (d, J = 6.7 Hz), 11.31, -5.14.

^{31}P NMR (202 MHz, Chloroform-d) δ +17.94.

HPLC: *(R)*-**4a** 88% ee, Chiralpak IA-3 column, hexane : isopropanol = 90 : 10, 0.3 mL/min, λ = 254 nm, t_R (major) = 50.22 min, t_R (minor) = 53.08 min.

HRMS (ESI-TOF): calcd for $C_{40}H_{50}N_2O_5PSi^+$ [M+H$^+$] 697.3221, found 697.3224.

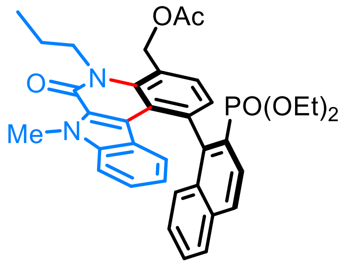

4b

(R)-(1-(2-(diethoxyphosphoryl)naphthalen-1-yl)-7-methyl-6-oxo-5-propyl-6,7-dihydro-5H-indolo[2,3-c]quinolin-4-yl)methyl acetate (4b)

Physical state: yellow oil.

Yield: *(R)*-**4b**, 30.6 mg, 49%.

^{1}H NMR (500 MHz, Chloroform-d) δ 8.22 (dd, *J* = 12.2, 8.6 Hz, 1H), 8.10 (dd, *J* = 8.6, 3.5 Hz, 1H), 7.86 (d, *J* = 8.2 Hz, 1H), 7.49 (d, *J* = 7.8 Hz, 1H), 7.40 (t, *J* = 7.5 Hz, 1H), 7.29 – 7.24 (m, 2H), 7.21 (d, *J* = 8.3 Hz, 1H), 7.13 (t, *J* = 7.7 Hz, 1H), 7.05 (t, *J* = 7.6 Hz, 1H), 6.29 (t, *J* = 7.8 Hz, 1H), 5.46 (s, 2H), 5.19 (d, *J* = 8.6 Hz, 1H), 4.54 – 4.47 (m, 1H), 4.35 – 4.30 (m, 1H), 4.29 (s, 3H), 3.81 – 3.70 (m, 2H), 3.63 – 3.47 (m, 2H), 2.18 (s, 3H), 1.76 – 1.66 (m, 2H), 0.94 (t, *J* = 7.1 Hz, 3H), 0.84 (t, *J* = 7.0 Hz, 3H), 0.80 (t, *J* = 7.4 Hz, 3H).

^{13}C NMR (126 MHz, Chloroform-d) δ 170.83, 159.19, 146.77 (d, *J* = 9.3 Hz), 140.41, 137.20, 134.96 (d, *J* = 2.5 Hz), 134.19 (d, *J* = 5.3 Hz), 133.65 (d, *J* = 15.5 Hz), 128.95 (d, *J* = 10.0 Hz), 128.95, 128.20 (d, *J* = 14.4 Hz), 128.07, 127.92, 127.37, 127.30, 127.02, 126.47 (d, *J* = 188.9 Hz), 125.05, 124.83, 124.26, 123.69, 121.55, 119.86, 118.79, 109.38, 65.26, 61.90 (d, *J* = 5.9 Hz), 61.61 (d, *J* = 6.0 Hz), 51.76, 31.92, 21.78, 21.12, 15.99 (d, *J* = 6.6 Hz), 15.86 (d, *J* = 6.6 Hz), 11.27.

^{31}P NMR (202 MHz, Chloroform-d) δ +17.68.

HPLC: *(R)*-**4b** 86% ee, Chiralpak OD-H column, hexane : isopropanol = 80 : 20, 1.0 mL/min, λ = 254 nm, t_R (major) = 7.03 min, t_R (minor) = 6.29 min.

HRMS (ESI-TOF): calcd for $C_{36}H_{38}N_2O_6P^+$ [M+H$^+$] 625.2462, found 625.2458.

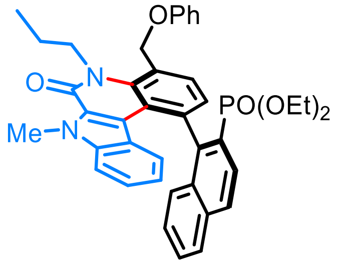

4c

diethyl (R)-(1-(7-methyl-6-oxo-4-(phenoxymethyl)-5-propyl-6,7-dihydro-5H-indolo[2,3-c]quinolin-1-yl)naphthalen-2-yl)phosphonate (4c)

Physical state: yellow solid.

Yield: *(R)*-**4c**, 30.9 mg, 47%.

^{1}H NMR (500 MHz, Chloroform-d) δ 8.24 (dd, *J* = 12.1, 8.7 Hz, 1H), 8.11 (dd, *J* = 8.5, 3.3 Hz, 1H), 7.87 (d, *J* = 8.2 Hz, 1H), 7.62 (d, *J* = 7.7 Hz, 1H), 7.41 (t, *J* = 7.4 Hz, 1H), 7.34 –

7.28 (m, 3H), 7.24 (dd, J = 12.0, 8.1 Hz, 2H), 7.16 – 7.11 (m, 1H), 7.06 (t, J = 7.6 Hz, 1H), 7.02 – 6.95 (m, 3H), 6.31 (t, J = 7.8 Hz, 1H), 5.34 (s, 2H), 5.19 (d, J = 8.7 Hz, 1H), 4.59 – 4.52 (m, 1H), 4.46 – 4.39 (m, 1H), 4.32 (s, 3H), 3.78 – 3.69 (m, 2H), 3.63 – 3.56 (m, 1H), 3.52 – 3.45 (m, 1H), 1.77 – 1.66 (m, 2H), 0.95 (t, J = 7.0 Hz, 3H), 0.81 (t, J = 7.0 Hz, 3H), 0.76 (t, J = 7.3 Hz, 3H).

^{13}C NMR (126 MHz, Chloroform-d) δ 159.17, 158.24, 146.83 (d, J = 9.1 Hz), 140.36, 137.16, 134.90 (d, J = 2.4 Hz), 134.00 (d, J = 5.2 Hz), 133.63 (d, J = 15.5 Hz), 129.55, 129.01, 129.00, 128.99, 128.90, 128.08 (d, J = 14.4 Hz), 127.99, 127.85, 127.72, 127.27, 126.94, 126.40 (d, J = 188.7 Hz), 125.65, 124.97, 124.28, 123.56, 121.53, 121.29, 119.80, 118.85, 114.79, 109.31, 69.04, 61.80 (d, J = 6.0 Hz), 61.55 (d, J = 6.0 Hz), 51.59, 31.88, 21.82, 15.95 (d, J = 6.6 Hz), 15.77 (d, J = 6.6 Hz), 11.18.

^{31}P NMR (202 MHz, Chloroform-*d*) δ +17.70.

HPLC: *(R)*-**4c** 89% ee, Chiralpak IA-3 column, hexane : isopropanol = 70 : 30, 0.5 mL/min, λ = 254 nm, t_R (major) = 15.28 min, t_R (minor) = 16.34 min.

HRMS (ESI-TOF): calcd for $C_{40}H_{40}N_2O_5P^+$ [M+H$^+$] 659.2669, found 659.2666.

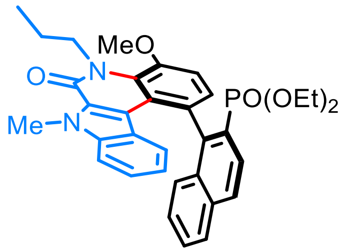

4d

diethyl *(R)*-(1-(4-methoxy-7-methyl-6-oxo-5-propyl-6,7-dihydro-5H-indolo[2,3-c]quinolin-1-yl)naphthalen-2-yl)phosphonate (4d)

Physical state: white solid.

Yield: *(R)*-**4d**, 33.8 mg, 58%.

^{1}H NMR (500 MHz, Chloroform-d) δ 8.24 – 8.18 (m, 1H), 8.06 (dd, J = 8.3, 3.2 Hz, 1H), 7.82 (d, J = 8.1 Hz, 1H), 7.36 – 7.33 (m, 1H), 7.25 (dd, J = 14.2, 8.6 Hz, 2H), 7.19 (d, J = 8.2 Hz, 1H), 7.13 – 7.00 (m, 3H), 6.28 (t, J = 7.7 Hz, 1H), 5.31 (d, J = 8.6 Hz, 1H), 4.58 – 4.43 (m, 2H), 4.31 (s, 3H), 4.04 (s, 3H), 3.86 – 3.82 (m, 1H), 3.78 – 3.73 (m, 1H), 3.67 – 3.56 (m, 2H), 2.07 – 1.95 (m, 2H), 0.99 (t, J = 7.3 Hz, 3H), 0.96 – 0.88 (m, 6H).

^{13}C NMR (126 MHz, Chloroform-d) δ 158.60, 149.33, 147.51 (d, J = 9.5 Hz), 140.31, 134.81 (d, J = 2.6 Hz), 133.92 (d, J = 15.5 Hz), 129.14, 128.78 (d, J = 10.0 Hz), 128.14, 127.86, 127.78 (d, J = 13.4 Hz), 127.28, 127.08, 126.70, 126.66, 126.62, 126.42 (d, J = 189.6 Hz), 124.72, 124.25, 123.51, 121.62, 119.47, 118.57, 109.15, 108.22, 61.80 (d, J = 6.0 Hz), 61.59 (d, J = 6.2 Hz), 56.06, 51.32, 31.88, 22.94, 15.97 (d, J = 6.7 Hz), 15.87 (d, J = 6.7 Hz), 11.57.

^{31}P NMR (202 MHz, Chloroform-*d*) δ +18.07.

HPLC: *(R)*-**4d** 91% ee, Chiralpak OD-H column, hexane : isopropanol = 95 : 5, 0.5 mL/min, λ = 254 nm, t_R (major) = 22.07 min, t_R (minor) = 12.37 min.

HRMS (ESI-TOF): calcd for $C_{34}H_{36}N_2O_5P^+$ [M+H$^+$] 583.2356, found 583.2360.

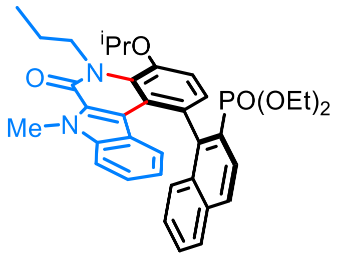

4e

diethyl *(R)-(*1-(4-isopropoxy-7-methyl-6-oxo-5-propyl-6,7-dihydro-5H-indolo[2,3-c]quinolin-1-yl)naphthalen-2-yl)phosphonate (4e)

Physical state: yellow oil.

Yield: *(R)*-**4e**, 44.6 mg, 73%.

^{1}H NMR (500 MHz, Chloroform-d) δ 8.21 (dd, *J* = 12.2, 8.6 Hz, 1H), 8.06 (dd, *J* = 8.6, 3.7 Hz, 1H), 7.81 (d, *J* = 8.2 Hz, 1H), 7.33 (t, *J* = 7.5 Hz, 1H), 7.25 (dd, *J* = 8.2, 3.2 Hz, 2H), 7.18 (d, *J* = 8.3 Hz, 1H), 7.07 (dd, *J* = 7.9, 4.6 Hz, 2H), 7.02 (t, *J* = 7.6 Hz, 1H), 6.28 (dd, *J* = 8.5, 7.1 Hz, 1H), 5.32 (d, *J* = 8.7 Hz, 1H), 4.82 – 4.75 (m, 1H), 4.67 – 4.56 (m, 2H), 4.30 (s, 3H), 3.87 – 3.74 (m, 2H), 3.65 – 3.54 (m, 2H), 2.02 – 1.90 (m, 2H), 1.49 (d, *J* = 6.0 Hz, 6H), 1.02 – 0.87 (m, 9H).

^{13}C NMR (126 MHz, Chloroform-d) δ 158.71, 147.51 (d, *J* = 9.3 Hz), 147.32, 140.28, 134.80 (d, *J* = 2.4 Hz), 133.90 (d, *J* = 15.9 Hz), 129.13, 128.81 (d, *J* = 10.0 Hz), 128.06, 127.73 (d, *J* = 13.8 Hz), 127.70, 127.63, 127.27, 126.65, 126.41 (d, *J* = 189.6 Hz), 126.29, 126.24, 124.67, 124.22, 123.91, 121.60, 119.43, 118.57, 110.29, 109.11, 71.13, 61.75 (d, *J* = 6.1 Hz), 61.54 (d, *J* = 6.1 Hz), 50.52, 31.84, 22.52, 21.98, 21.86, 15.96 (d, *J* = 6.6 Hz), 15.84 (d, *J* = 6.7 Hz), 11.23.

^{31}P NMR (202 MHz, Chloroform-*d*) δ +18.19.

HPLC: *(R)*-**4e** 91% ee, Chiralpak AD-H column, hexane : isopropanol = 80 : 20, 1.0 mL/min, λ = 254 nm, t_R (major) = 19.95 min, t_R (minor) = 11.18 min.

HRMS (ESI-TOF): calcd for $C_{36}H_{40}N_2O_5P^+$ [M+H$^+$] 611.2669, found 611.2674.

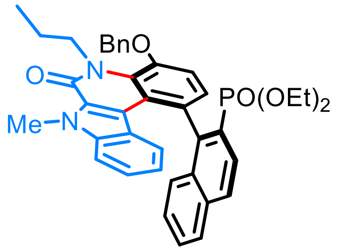

4f

diethyl *(R)-(*1-(4-(benzyloxy)-7-methyl-6-oxo-5-propyl-6,7-dihydro-5H-indolo[2,3-c]quinolin-1-yl)naphthalen-2-yl)phosphonate (4f)

Physical state: yellow oil.

Yield: *(R)*-**4f**, 41.5 mg, 63%.

^{1}H NMR (500 MHz, Chloroform-d) δ 8.21 (dd, *J* = 12.1, 8.6 Hz, 1H), 8.07 (dd, *J* = 8.6, 3.7 Hz, 1H), 7.82 (d, *J* = 8.1 Hz, 1H), 7.53 (d, *J* = 7.0 Hz, 2H), 7.45 (t, *J* = 7.3 Hz, 2H), 7.41 – 7.38 (m, 1H), 7.36 (t, *J* = 7.5 Hz, 1H), 7.27 – 7.24 (m, 2H), 7.18 (dd, *J* = 13.0, 8.4 Hz, 2H), 7.10 – 7.06 (m, 1H), 7.04 (t, *J* = 7.6 Hz, 1H), 6.29 (t, *J* = 7.8 Hz, 1H), 5.33 (d, *J* = 8.6 Hz,

1H), 5.28 – 5.22 (m, 2H), 4.57 – 4.46 (m, 2H), 4.31 (s, 3H), 3.88 – 3.82 (m, 1H), 3.79 – 3.73 (m, 1H), 3.68 – 3.57 (m, 2H), 1.90 – 1.78 (m, 2H), 0.94 (t, *J* = 7.0 Hz, 6H), 0.64 (t, *J* = 7.4 Hz, 3H).

^{13}C NMR (126 MHz, Chloroform-d) δ 158.60, 148.59, 147.44 (d, *J* = 9.5 Hz), 140.29, 135.98, 134.80 (d, *J* = 2.5 Hz), 133.86 (d, *J* = 15.4 Hz), 129.10, 128.79 (d, *J* = 10.2 Hz), 128.66, 128.41, 128.32, 128.12, 127.81, 127.80 (d, *J* = 14.5 Hz), 127.72, 127.25, 127.05, 126.93, 126.89, 126.69, 126.41 (d, *J* = 189.6 Hz), 124.71, 124.22, 123.72, 121.60, 119.47, 118.49, 109.40, 109.14, 71.88, 61.78 (d, *J* = 6.2 Hz), 61.57 (d, *J* = 6.2 Hz), 50.72, 31.86, 22.67, 15.98 (d, *J* = 6.5 Hz), 15.88 (d, *J* = 6.7 Hz), 10.90.

^{31}P NMR (202 MHz, Chloroform-*d*) δ +18.04.

HPLC: *(R)*-**4f** 92% ee, Chiralpak AD-H column, hexane : isopropanol = 80 : 20, 1.0 mL/min, λ = 254 nm, t_R (major) = 30.16 min, t_R (minor) = 17.40 min.

HRMS (ESI-TOF): calcd for $C_{40}H_{40}N_2O_5P^+$ [M+H$^+$] 659.2669, found 659.2671.

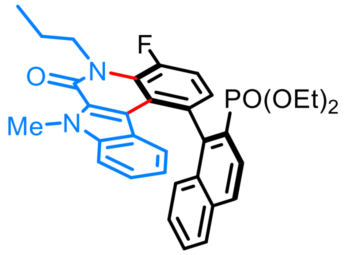

4g

diethyl *(R)-(*1-(4-fluoro-7-methyl-6-oxo-5-propyl-6,7-dihydro-5H-indolo[2,3-c]quinolin-1-yl)naphthalen-2-yl)phosphonate (4g)

Physical state: light yellow solid.

Yield: *(R)*-**4g**, 38.2 mg, 67%.

^{1}H NMR (500 MHz, Chloroform-d) δ 8.20 (dd, *J* = 12.1, 8.6 Hz, 1H), 8.10 (dd, *J* = 8.6, 3.6 Hz, 1H), 7.86 (d, *J* = 8.2 Hz, 1H), 7.39 (t, *J* = 7.4 Hz, 1H), 7.30 – 7.25 (m, 2H), 7.23 (d, *J* = 2.4 Hz, 1H), 7.22 (d, *J* = 2.7 Hz, 1H), 7.15 – 7.12 (m, 1H), 7.06 (t, *J* = 7.5 Hz, 1H), 6.30 (t, *J* = 8.0 Hz, 1H), 5.24 (d, *J* = 8.7 Hz, 1H), 4.57 – 4.47 (m, 2H), 4.33 (s, 3H), 3.83 – 3.74 (m, 2H), 3.66 – 3.56 (m, 2H), 2.09 – 2.00 (m, 2H), 1.07 (t, *J* = 7.4 Hz, 3H), 0.96 (t, *J* = 7.1 Hz, 3H), 0.89 (t, *J* = 7.1 Hz, 3H).

13C NMR (126 MHz, Chloroform-d) δ 157.42, 151.26 (d, *J* = 246.3 Hz), 146.77 (d, *J* = 9.4 Hz), 140.34, 134.86 (d, *J* = 2.5 Hz), 133.63 (d, *J* = 15.5 Hz), 130.28 (d, *J* = 8.9 Hz), 130.27 (d, *J* = 1.6 Hz), 128.77 (d, *J* = 9.9 Hz), 128.65 (d, *J* = 8.7 Hz), 128.14 (d, *J* = 14.4 Hz), 128.01, 127.85, 127.13, 126.97, 126.55 (d, *J* = 189.1 Hz), 125.14 (d, *J* = 5.9 Hz), 125.04, 124.27, 123.83, 121.44, 119.81, 118.36 (d, *J* = 2.4 Hz), 112.67 (d, *J* = 25.1 Hz), 109.31, 61.80 (d, *J* = 6.0 Hz), 61.60 (d, *J* = 6.0 Hz), 49.48 (d, *J* = 16.2 Hz), 31.95, 22.88 (d, *J* = 6.0 Hz), 15.94 (d, *J* = 6.5 Hz), 15.81 (d, *J* = 6.8 Hz), 11.39.

^{31}P NMR (202 MHz, Chloroform-*d*) δ +17.61.

^{19}F NMR (471 MHz, Chloroform-*d*) δ -119.25.

HPLC: *(R)*-**4g** 92% ee, Chiralpak AD-H column, hexane : isopropanol = 80 : 20, 1.0 mL/min, λ = 254 nm, t_R(major) = 9.87 min, t_R (minor) = 8.31 min.

HRMS (ESI-TOF): calcd for $C_{33}H_{33}FN_2O_4P^+$ [M+H$^+$] 571.2156, found 571.2153.

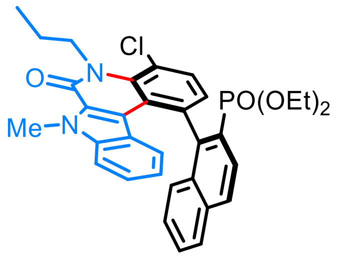

4h

diethyl *(R)-(*1-(4-chloro-7-methyl-6-oxo-5-propyl-6,7-dihydro-5H-indolo[2,3-c]quinolin-1-yl)naphthalen-2-yl)phosphonate (4h)

Physical state: yellow oil.

Yield: *(R)*-**4h**, 32.2 mg, 55%.

^{1}H NMR (500 MHz, Chloroform-d) δ 8.28 – 8.21 (m, 1H), 8.14 (dd, *J* = 8.5, 3.9 Hz, 1H), 7.89 (d, *J* = 8.4 Hz, 1H), 7.51 (d, *J* = 8.2 Hz, 1H), 7.43 (t, *J* = 7.4 Hz, 1H), 7.30 – 7.21 (m, 3H), 7.17 (t, *J* = 7.7 Hz, 1H), 7.08 (t, *J* = 7.5 Hz, 1H), 6.32 (t, *J* = 7.8 Hz, 1H), 5.22 (d, *J* = 8.6 Hz, 1H), 4.79 – 4.61 (m, 2H), 4.33 (s, 3H), 3.89 – 3.74 (m, 2H), 3.72 – 3.65 (m, 1H), 3.63 – 3.52 (m, 1H), 1.94 – 1.84 (m, 2H), 1.05 – 0.95 (m, 3H), 0.93 – 0.84 (m, 6H).

^{13}C NMR (126 MHz, Chloroform-d) δ 159.21, 146.17 (d, *J* = 9.4 Hz), 140.36, 134.90 (d, *J* = 2.6 Hz), 134.62, 133.50 (d, *J* = 15.4 Hz), 132.89 (d, *J* = 5.5 Hz), 129.39, 128.90 (d, *J* = 9.9 Hz), 128.27 (d, *J* = 14.3 Hz), 128.15, 128.08, 127.91, 127.53, 127.08, 127.05, 126.48 (d, *J* = 188.6 Hz), 125.52, 125.07, 124.13, 123.32, 121.41, 119.96, 118.02, 109.38, 61.91 (d, *J* = 6.0 Hz), 61.64 (d, *J* = 6.0 Hz), 51.00, 31.94, 22.14, 15.99 (d, *J* = 6.6 Hz), 15.85 (d, *J* = 6.5 Hz), 11.29.

^{31}P NMR (202 MHz, Chloroform-*d*) δ +17.61.

HPLC: *(R)*-**4h** 87% ee, Chiralpak AD-H column, hexane : isopropanol = 80 : 20, 1.0 mL/min, λ = 254 nm, t_R (major) = 11.89 min, t_R (minor) = 9.94 min.

HRMS (ESI-TOF): calcd for $C_{33}H_{33}ClN_2O_4P^+$ [M+H$^+$] 587.1861, found 587.1864.

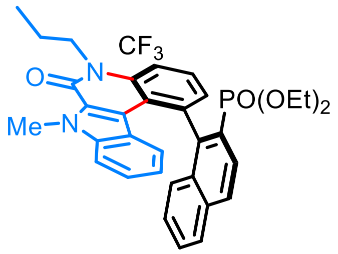

4i

diethyl *(R)-(*1-(7-methyl-6-oxo-5-propyl-4-(trifluoromethyl)-6,7-dihydro-5H-indolo[2,3-c]quinolin-1-yl)naphthalen-2-yl)phosphonate (4i)

Physical state: white solid.

Yield: *(R)*-**4i**, 32.9 mg, 53%.

^{1}H NMR (500 MHz, Chloroform-d) δ 8.24 (dd, *J* = 12.1, 8.7 Hz, 1H), 8.15 (dd, *J* = 8.5, 3.4 Hz, 1H), 7.91 (d, *J* = 8.2 Hz, 1H), 7.70 (d, *J* = 8.0 Hz, 1H), 7.45 (t, *J* = 7.4 Hz, 1H), 7.33 (d, *J* = 8.0 Hz, 1H), 7.25 (dd, *J* = 8.3, 2.2 Hz, 2H), 7.22 – 7.16 (m, 1H), 7.08 (t, *J* = 7.6 Hz, 1H), 6.32 (t, *J* = 7.8 Hz, 1H), 5.08 (d, *J* = 8.6 Hz, 1H), 4.63 – 4.55 (m, 1H), 4.53 – 4.45 (m, 1H),

4.31 (s, 3H), 3.81 – 3.71 (m, 2H), 3.70 – 3.62 (m, 1H), 3.52 – 3.43 (m, 1H), 1.63 – 1.53 (m, 2H), 0.95 (t, *J* = 7.0 Hz, 3H), 0.80 (t, *J* = 7.0 Hz, 3H), 0.73 (t, *J* = 7.3 Hz, 3H).
^{13}C NMR (126 MHz, Chloroform-d) δ 158.12, 145.66 (d, *J* = 9.0 Hz), 140.31, 137.37, 135.82, 134.96 (d, *J* = 2.5 Hz), 133.30 (d, *J* = 15.4 Hz), 128.95 (d, *J* = 9.9 Hz), 128.56 (d, *J* = 14.4 Hz), 128.30, 128.29, 128.23, 128.04, 127.29, 126.98, 126.62 (d, *J* = 188.7 Hz), 125.22, 124.72, 124.63 (d, *J* = 272.4 Hz), 124.30 (d, *J* = 5.5 Hz), 124.10, 121.34, 120.22, 119.52 (d, *J* = 31.3 Hz), 117.92, 109.52, 61.97 (d, *J* = 6.1 Hz), 61.60 (d, *J* = 6.0 Hz), 52.95 (d, *J* = 5.6 Hz), 31.91, 21.36, 15.90 (d, *J* = 6.8 Hz), 15.75 (d, *J* = 6.7 Hz), 11.16.
^{31}P NMR (202 MHz, Chloroform-*d*) δ +17.34.
^{19}F NMR (471 MHz, Chloroform-*d*) δ -55.52.
HPLC: *(R)*-**4i** 87% ee, Chiralpak IA-3 column, hexane : isopropanol = 70 : 30, 0.5 mL/min, λ = 254 nm, t_R (major) = 8.29 min, t_R (minor) = 6.88 min.
HRMS (ESI-TOF): calcd for $C_{34}H_{33}F_3N_2O_4P^+$ [M+H$^+$] 621.2125, found 621.2119.

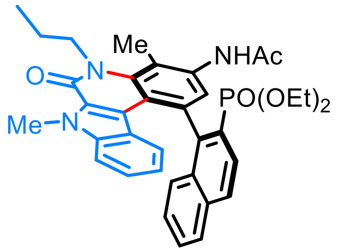

4j

diethyl *(R)*-(1-(3-acetamido-4,7-dimethyl-6-oxo-5-propyl-6,7-dihydro-5H-indolo[2,3-c]quinolin-1-yl)naphthalen-2-yl)phosphonate (4j)
Physical state: yellow solid.
Yield: *(R)*-**4j**, 36.8 mg, 59%.
^{1}H NMR (500 MHz, Chloroform-d) δ 8.54 (q, *J* = 16.6, 14.8 Hz, 1H), 8.11 – 7.99 (m, 2H), 7.81 (d, *J* = 8.1 Hz, 1H), 7.41 – 7.32 (m, 2H), 7.23 – 7.18 (m, 2H), 7.11 (t, *J* = 7.5 Hz, 1H), 7.04 (t, *J* = 7.5 Hz, 1H), 6.27 (t, *J* = 7.7 Hz, 1H), 5.10 (d, *J* = 8.7 Hz, 1H), 4.59 – 4.50 (m, 1H), 4.44 – 4.36 (m, 1H), 4.29 (s, 3H), 3.85 – 3.78 (m, 1H), 3.67 – 3.60 (m, 1H), 3.58 – 3.51 (m, 1H), 3.40 – 3.29 (m, 1H), 2.47 (s, 3H), 2.15 (s, 3H), 1.77 – 1.66 (m, 2H), 0.98 (t, *J* = 6.7 Hz, 3H), 0.73 (t, *J* = 7.3 Hz, 3H), 0.67 (t, *J* = 6.9 Hz, 3H).
^{13}C NMR (126 MHz, Chloroform-d) δ 168.29, 159.74, 146.87 (d, *J* = 9.3 Hz), 140.34, 138.89, 134.75 (d, *J* = 1.6 Hz), 133.64, 133.52, 131.38 (d, *J* = 9.3 Hz), 128.19, 128.11, 127.99 (d, *J* = 3.9 Hz), 127.91 (d, *J* = 2.6 Hz), 127.76, 127.36, 127.06, 126.02 (d, *J* = 191.2 Hz), 124.83, 124.52, 124.16, 123.38, 121.40, 121.22, 119.50, 118.51, 109.33, 62.16 (d, *J* = 6.1 Hz), 61.70 (d, *J* = 6.4 Hz), 51.34, 31.77, 23.51, 21.34, 19.08, 15.92 (d, *J* = 6.7 Hz), 15.55 (d, *J* = 6.1 Hz), 11.22.
^{31}P NMR (202 MHz, Chloroform-*d*) δ +17.56.
HPLC: *(R)*-**4j** 86% ee, Chiralpak AD-H column, hexane : isopropanol = 90 : 10, 0.5 mL/min, λ = 254 nm, t_R (major) = 39.52 min, t_R (minor) = 33.73 min.
HRMS (ESI-TOF): calcd for $C_{36}H_{39}N_3O_5P^+$ [M+H$^+$] 624.2622, found 624.2625.

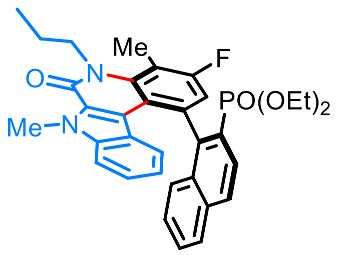

4k

diethyl *(R)-(*1-(3-fluoro-4,7-dimethyl-6-oxo-5-propyl-6,7-dihydro-5H-indolo[2,3-c]quinolin-1-yl)naphthalen-2-yl)phosphonate (4k)

Physical state: white solid.

Yield: *(R)*-**4k**, 36.2 mg, 62%.

^{1}H NMR (500 MHz, Chloroform-d) δ 8.26 (dd, *J* = 12.2, 8.6 Hz, 1H), 8.11 (dd, *J* = 8.6, 3.5 Hz, 1H), 7.84 (d, *J* = 8.2 Hz, 1H), 7.37 (t, *J* = 7.4 Hz, 1H), 7.24 (d, *J* = 8.6 Hz, 1H), 7.17 (d, *J* = 8.3 Hz, 1H), 7.13 – 7.08 (m, 1H), 7.08 – 6.96 (m, 2H), 6.27 (t, *J* = 7.7 Hz, 1H), 5.11 (d, *J* = 8.6 Hz, 1H), 4.57 – 4.49 (m, 1H), 4.43 – 4.36 (m, 1H), 4.27 (s, 3H), 3.88 – 3.81 (m, 1H), 3.80 – 3.70 (m, 2H), 3.52 – 3.43 (m, 1H), 2.58 (s, 3H), 1.71 – 1.59 (m, 2H), 1.05 (t, *J* = 7.1 Hz, 3H), 0.81 – 0.70 (m, 6H).

^{13}C NMR (126 MHz, Chloroform-d) δ 159.77, 158.62 (d, *J* = 242.8 Hz), 145.99 (d, *J* = 8.8 Hz), 140.27, 138.81 (d, *J* = 6.2 Hz), 134.84 (d, *J* = 2.7 Hz), 133.40 (d, *J* = 15.4 Hz), 132.92 (d, *J* = 5.6 Hz), 132.85 (d, *J* = 5.2 Hz), 129.01 (d, *J* = 9.9 Hz), 128.19 (d, *J* = 14.4 Hz), 127.88 (d, *J* = 22.4 Hz), 127.29, 126.98, 126.94, 126.28 (d, *J* = 188.2 Hz), 124.88, 124.06, 121.24, 120.29, 119.58, 118.59, 115.84 (d, *J* = 24.7 Hz), 114.10 (d, *J* = 17.9 Hz), 109.19, 61.75 (d, *J* = 5.9 Hz), 61.50 (d, *J* = 6.0 Hz), 50.98, 31.72, 21.57, 15.87 (d, *J* = 6.9 Hz), 15.69 (d, *J* = 6.5 Hz), 14.79 (d, *J* = 6.9 Hz), 11.14.

^{31}P NMR (202 MHz, Chloroform-*d*) δ +17.69.

^{19}F NMR (471 MHz, Chloroform-*d*) δ -111.70.

HPLC: *(R)*-**4k** 90% ee, Chiralpak AD-H column, hexane : isopropanol = 80 : 20, 1.0 mL/min, λ = 254 nm, t_R (major) = 9.51 min, t_R (minor) = 12.86 min.

HRMS (ESI-TOF): calcd for $C_{34}H_{35}FN_2O_4P^+$ [M+H$^+$] 585.2313, found 585.2318.

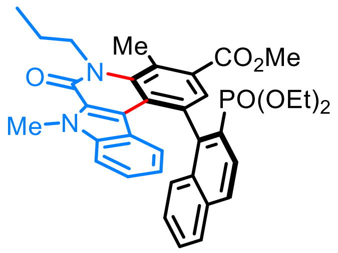

4l

methyl *(R)*-1-(2-(diethoxyphosphoryl)naphthalen-1-yl)-4,7-dimethyl-6-oxo-5-propyl-6,7-dihydro-5H-indolo[2,3-c]quinoline-3-carboxylate (4l)

Physical state: yellow solid.

Yield: *(R)*-**4l**, 40.6 mg, 65%.

^{1}H NMR (500 MHz, Chloroform-d) δ 8.28 (dd, *J* = 12.2, 8.5 Hz, 1H), 8.14 (dd, *J* = 8.6, 3.6 Hz, 1H), 7.89 (d, *J* = 8.2 Hz, 1H), 7.81 (s, 1H), 7.41 (t, *J* = 7.4 Hz, 1H), 7.26 – 7.20 (m, 2H),

7.17 – 7.11 (m, 1H), 7.05 (t, J = 7.6 Hz, 1H), 6.31 (t, J = 7.8 Hz, 1H), 5.06 (d, J = 8.7 Hz, 1H), 4.51 – 4.44 (m, 1H), 4.39 – 4.34 (m, 1H), 4.30 (s, 3H), 3.85 (s, 3H), 3.83 – 3.68 (m, 3H), 3.48 – 3.39 (m, 1H), 2.84 (s, 3H), 1.67 – 1.54 (m, 2H), 1.02 (t, J = 7.0 Hz, 3H), 0.79 – 0.67 (m, 6H).

^{13}C NMR (126 MHz, Chloroform-d) δ 167.51, 159.65, 146.21 (d, J = 8.8 Hz), 140.35, 139.00, 134.94 (d, J = 2.4 Hz), 133.80 (d, J = 15.3 Hz), 130.74 (d, J = 5.5 Hz), 130.72, 129.51, 129.14 (d, J = 10.0 Hz), 128.77, 128.18 (d, J = 14.4 Hz), 128.05, 127.89, 127.17, 127.03, 126.84, 126.55 (d, J = 188.1 Hz), 125.04, 124.14, 121.52, 120.22, 117.89, 109.43, 61.75 (d, J = 5.9 Hz), 61.54 (d, J = 5.7 Hz), 52.54, 51.88, 31.88, 21.73, 21.44, 15.86 (d, J = 6.8 Hz), 15.66 (d, J = 6.6 Hz), 11.27.

^{31}P NMR (202 MHz, Chloroform-*d*) δ +17.75.

HPLC: *(R)*-**4l** 90% ee, Chiralpak AD-H column, hexane : isopropanol = 80 : 20, 1.0 mL/min, λ = 254 nm, t_R (major) = 14.35 min, t_R (minor) = 17.77 min.

HRMS (ESI-TOF): calcd for $C_{36}H_{38}N_2O_6P^+$ [M+H$^+$] 625.2462, found 625.2458.

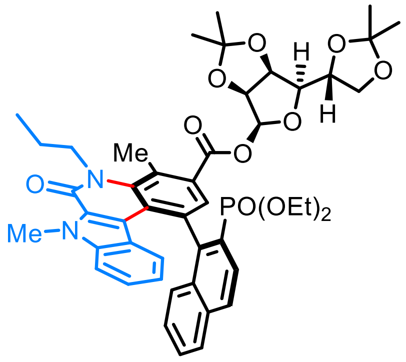

4m

(3a*S*,4*S*,6*R*,6a*S*)-6-(*(R)*-2,2-dimethyl-1,3-dioxolan-4-yl)-2,2-dimethyltetrahydrofuro[3,4-d][1,3]dioxol-4-yl 1-(2-(diethoxyphosphoryl)naphthalen-1-yl)-4,7-dimethyl-6-oxo-5-propyl-6,7-dihydro-5H-indolo[2,3-c]quinoline-3-carboxylate (4m)

Physical state: white solid.

Yield: *(R)*-**4m**, 42.6 mg, 50%, dr > 20:1.

^{1}H NMR (500 MHz, Chloroform-d) δ 8.28 (dd, J = 12.2, 8.7 Hz, 1H), 8.16 (dd, J = 8.5, 3.5 Hz, 1H), 7.92 (d, J = 8.2 Hz, 1H), 7.73 (s, 1H), 7.45 (t, J = 7.4 Hz, 1H), 7.24 (d, J = 8.3 Hz, 2H), 7.19 – 7.15 (m, 1H), 7.07 (t, J = 7.6 Hz, 1H), 6.34 (s, 1H), 6.32 (d, J = 8.0 Hz, 1H), 5.02 (d, J = 8.6 Hz, 1H), 4.86 (dd, J = 5.7, 3.7 Hz, 1H), 4.79 (d, J = 5.9 Hz, 1H), 4.49 – 4.39 (m, 2H), 4.31 (s, 4H), 4.10 – 4.06 (m, 2H), 4.00 (dd, J = 8.8, 4.3 Hz, 1H), 3.82 – 3.68 (m, 3H), 3.41 – 3.35 (m, 1H), 2.84 (s, 3H), 1.64 – 1.55 (m, 2H), 1.50 (s, 3H), 1.38 (s, 3H), 1.35 (s, 3H), 1.32 (s, 3H), 0.99 (t, J = 7.0 Hz, 3H), 0.72 (q, J = 7.1 Hz, 6H).

^{13}C NMR (126 MHz, Chloroform-d) δ 165.44, 159.62, 146.00 (d, J = 9.4 Hz), 140.40, 139.25, 135.02 (d, J = 2.7 Hz), 133.90 (d, J = 15.2 Hz), 130.84 (d, J = 5.2 Hz), 130.71, 130.04, 129.29, 129.21, 128.95, 128.37, 128.25, 128.16, 127.98, 127.24, 127.13, 126.73 (d, J = 179.1 Hz), 125.15, 124.16, 121.56, 120.34, 117.81, 113.25, 109.52, 109.26, 101.75, 85.21, 82.52, 79.38, 72.89, 66.68, 61.86 (d, J = 6.0 Hz), 61.62 (d, J = 5.7 Hz), 52.73, 31.95, 26.88, 25.94, 25.09, 24.65, 21.92, 21.48, 16.03 (d, J = 6.3 Hz), 15.76 (d, J = 6.4 Hz), 11.32.

^{31}P NMR (202 MHz, Chloroform-*d*) δ +17.74.

HRMS (ESI-TOF): calcd for $C_{47}H_{54}N_2O_{11}P^+$ [M+H$^+$] 853.3460, found 853.3464.

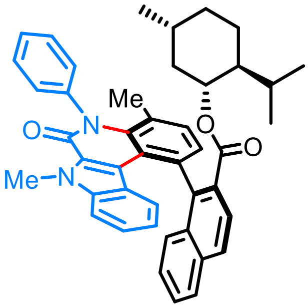

4n

(1*R*,2*S*,5*R*)-2-isopropyl-5-methylcyclohexyl 1-(4,7-dimethyl-6-oxo-5-phenyl-6,7-dihydro-5H-indolo[2,3-c]quinolin-1-yl)-2-naphthoate (4n)

Physical state: white solid.

Yield: *(R)*-**4n**, 40.7 mg, 63%, dr = 18:1 (determined by ^{1}H NMR), the diastereoisomers were inseparable.

^{1}H NMR (500 MHz, Chloroform-*d*) δ 8.04 (d, *J* = 8.6 Hz, 1H), 7.99 – 7.93 (m, 2H), 7.87 (d, *J* = 8.6 Hz, 1H), 7.74 (s, 1H), 7.61 – 7.45 (m, 3H), 7.42 (t, *J* = 7.4 Hz, 1H), 7.37 (d, *J* = 7.5 Hz, 1H), 7.27 (d, *J* = 8.3 Hz, 2H), 7.18 – 7.09 (m, 3H), 6.36 (t, *J* = 7.7 Hz, 1H), 5.64 (d, *J* = 8.6 Hz, 1H), 4.55 – 4.48 (m, 1H), 4.31 (s, 3H), 1.88 (s, 3H), 1.66 (s, 1H), 1.60 (d, *J* = 12.8 Hz, 1H), 1.49 (d, *J* = 9.9 Hz, 1H), 1.12 – 1.04 (m, 1H), 0.85 (d, *J* = 6.4 Hz, 5H), 0.69 (q, *J* = 12.2 Hz, 1H), 0.56 (d, *J* = 6.9 Hz, 3H), 0.50 – 0.41 (m, 4H).

^{13}C NMR (126 MHz, Chloroform-*d*) δ 167.40, 158.12, 142.59, 142.10, 140.69, 137.48, 135.07, 133.37, 132.93, 129.98, 129.79, 128.28, 128.00, 127.94, 127.81, 127.55, 127.33, 127.28, 127.18, 126.85, 126.34, 125.08, 124.16, 122.54, 121.64, 119.72, 119.62, 109.29, 74.41, 46.54, 40.13, 34.12, 31.81, 31.04, 25.44, 23.53, 22.85, 22.03, 20.58, 15.76.

HRMS (ESI-TOF): calcd for $C_{44}H_{43}N_2O_3^+$ [M+H$^+$] 647.3268, found 647.3265.

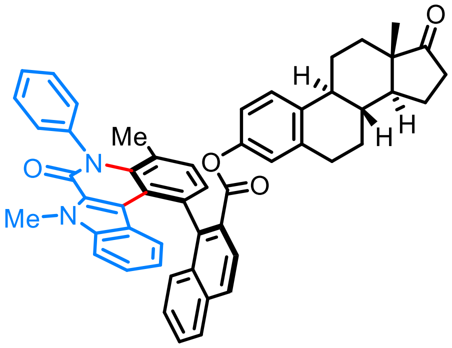

4o

(8*R*,9*S*,13*S*,14*S*)-13-methyl-17-oxo-7,8,9,11,12,13,14,15,16,17-decahydro-6H-cyclopenta[a]phenanthren-3-yl 1-(4,7-dimethyl-6-oxo-5-phenyl-6,7-dihydro-5H-indolo[2,3-c]quinolin-1-yl)-2-naphthoate (4o)

Physical state: white solid.

Yield: *(R)*-**4o**, 51.7 mg, 68%, dr = 11:1 (determined by ^{1}H NMR), the diastereoisomers were inseparable.

^{1}H NMR (500 MHz, Chloroform-*d*) δ 8.08 (d, *J* = 8.6 Hz, 1H), 8.04 (d, *J* = 8.6 Hz, 1H), 7.96 (dd, *J* = 13.4, 8.4 Hz, 2H), 7.57 – 7.47 (m, 3H), 7.41 – 7.35 (m, 3H), 7.28 (d, *J* = 8.3 Hz,

1H), 7.23 (d, J = 7.6 Hz, 1H), 7.18 (d, J = 7.8 Hz, 1H), 7.16 – 7.12 (m, 2H), 7.00 (s, 1H), 6.45 (dd, J = 8.4, 2.4 Hz, 1H), 6.42 – 6.38 (m, 1H), 6.21 (d, J = 2.3 Hz, 1H), 5.81 (d, J = 8.6 Hz, 1H), 4.31 (s, 3H), 2.81 – 2.73 (m, 2H), 2.50 (dd, J = 19.2, 8.6 Hz, 1H), 2.37 – 2.30 (m, 1H), 2.15 (dd, J = 18.9, 9.2 Hz, 2H), 2.05 – 2.00 (m, 1H), 1.98 – 1.92 (m, 2H), 1.82 (s, 3H), 1.57 – 1.34 (m, 6H), 0.90 (s, 3H).

^{13}C NMR (126 MHz, Chloroform-*d*) δ 166.92, 157.90, 148.28, 143.08, 141.80, 140.58, 137.67, 137.61, 137.00, 135.28, 132.97, 132.24, 129.87, 129.47, 129.30, 129.16, 128.94, 128.31, 128.18, 127.99, 127.83, 127.74, 127.26, 127.20, 127.06, 127.04, 126.14, 125.95, 125.14, 123.81, 122.19, 121.65, 120.84, 119.74, 119.33, 118.01, 109.42, 50.17, 47.72, 43.87, 37.68, 35.68, 31.71, 31.35, 29.14, 26.12, 25.56, 23.18, 21.37, 13.63.

HRMS (ESI-TOF): calcd for $C_{52}H_{45}N_2O_4^+$ [M+H$^+$] 761.3374, found 761.3376.

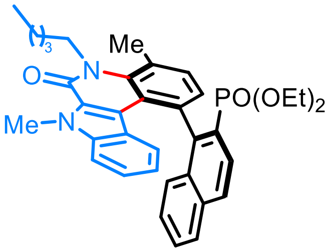

5a

diethyl *(R)*-(1-(4,7-dimethyl-6-oxo-5-pentyl-6,7-dihydro-5H-indolo[2,3-c]quinolin-1-yl)naphthalen-2-yl)phosphonate (5a)

Physical state: white solid.

Yield: *(R)*-**5a**, 36.3 mg, 61%.

^{1}H NMR (500 MHz, Chloroform-d) δ 8.22 (dd, J = 12.2, 8.6 Hz, 1H), 8.09 (dd, J = 8.6, 3.6 Hz, 1H), 7.86 (d, J = 8.2 Hz, 1H), 7.39 (t, J = 7.4 Hz, 1H), 7.28 – 7.24 (m, 2H), 7.23 – 7.18 (m, 2H), 7.13 – 7.09 (m, 1H), 7.04 (t, J = 7.6 Hz, 1H), 6.29 (t, J = 8.3 Hz, 1H), 5.20 (d, J = 8.6 Hz, 1H), 4.62 – 4.54 (m, 1H), 4.50 – 4.44 (m, 1H), 4.30 (s, 3H), 3.80 – 3.72 (m, 2H), 3.61 – 3.50 (m, 2H), 2.71 (s, 3H), 1.68 – 1.53 (m, 3H), 1.19 – 1.08 (m, 3H), 0.94 (t, J = 7.1 Hz, 3H), 0.86 (d, J = 7.0 Hz, 3H), 0.82 (t, J = 7.2 Hz, 3H).

^{13}C NMR (126 MHz, Chloroform-d) δ 159.64, 147.32 (d, J = 9.5 Hz), 140.38, 137.11, 134.93 (d, J = 2.3 Hz), 133.88 (d, J = 15.6 Hz), 131.75 (d, J = 5.3 Hz), 128.97 (d, J = 10.1 Hz), 128.90, 128.67, 127.98 (d, J = 3.7 Hz), 127.92, 127.81, 127.37, 126.91, 126.79, 126.41 (d, J = 189.2 Hz), 124.80, 124.34, 123.62, 121.65, 119.61, 118.98, 109.22, 61.80 (d, J = 6.1 Hz), 61.54 (d, J = 5.9 Hz), 48.61, 31.88, 29.04, 28.18, 23.55, 22.35, 15.98 (d, J = 6.6 Hz), 15.84 (d, J = 6.8 Hz), 13.92.

^{31}P NMR (202 MHz, Chloroform-*d*) δ +17.93.

HPLC: *(R)*-**5a** 89% ee, Chiralpak AD-H column, hexane : isopropanol = 80 : 20, 1.0 mL/min, λ = 254 nm, t_R (major) = 11.81 min, t_R (minor) = 12.55 min.

HRMS (ESI-TOF): calcd for $C_{36}H_{40}N_2O_4P^+$ [M+H$^+$] 595.2720, found 595.2718.

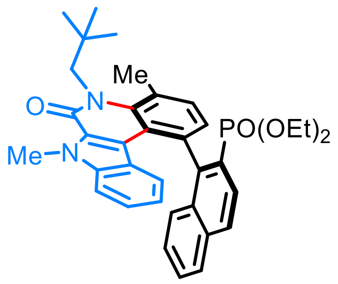

5b

diethyl (1-(4,7-dimethyl-5-neopentyl-6-oxo-6,7-dihydro-5H-indolo[2,3-c]quinolin-1-yl)naphthalen-2-yl)phosphonate (5b)

Physical state: white solid.

Yield: *(R)*-**5b**, 25.8 mg, 47%.

^{1}H NMR (500 MHz, Chloroform-*d*) δ 8.29 (s, 1H), 8.10 (dd, *J* = 8.5, 3.5 Hz, 1H), 7.84 (d, *J* = 6.9 Hz, 1H), 7.49 – 7.29 (m, 2H), 7.21 – 7.15 (m, 3H), 7.04 (q, *J* = 12.1, 7.6 Hz, 2H), 6.28 (t, *J* = 7.6 Hz, 1H), 5.15 (s, 1H), 4.29 (s, 3H), 3.86 – 3.66 (m, 3H), 3.56 – 3.38 (m, 1H), 2.69 (s, 3H), 1.33 – 1.10 (m, 2H), 0.98 (s, 3H), 0.82 (t, *J* = 7.0 Hz, 3H), 0.69 (s, 9H).

^{13}C NMR (126 MHz, Chloroform-*d*) δ 160.39, 147.25 (d, *J* = 9.2 Hz), 140.29, 137.62, 134.86 (d, *J* = 2.6 Hz), 133.89 (d, *J* = 15.6 Hz), 131.45 (d, *J* = 5.2 Hz), 129.10, 128.34, 127.89, 127.86, 127.77, 127.45 (d, *J* = 186.4 Hz), 127.29 (d, *J* = 5.5 Hz), 124.69, 124.30, 123.57, 121.60, 119.56, 118.54, 109.21, 61.87 (d, *J* = 5.9 Hz), 61.40 (d, *J* = 6.0 Hz), 55.65, 35.73, 31.90, 27.97, 23.96, 15.96 (d, *J* = 2.3 Hz), 15.91.

^{31}P NMR (202 MHz, Chloroform-*d*) δ +18.47.

HPLC: *(R)*-**5b** 90% ee, Chiralpak AD-H column, hexane : isopropanol = 80 : 20, 1.0 mL/min, λ = 254 nm, t_R (major) = 10.68 min, t_R (minor) = 8.26 min.

HRMS (ESI-TOF): calcd for $C_{36}H_{40}N_2O_4P^+$ [M+H$^+$] 595.2720, found 595.2723.

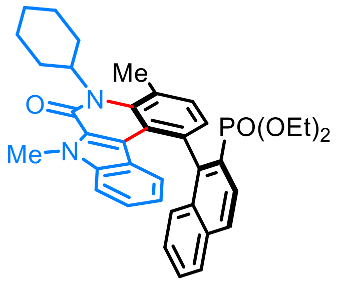

5c

diethyl *(R)-(*1-(5-cyclohexyl-4,7-dimethyl-6-oxo-6,7-dihydro-5H-indolo[2,3-c]quinolin-1-yl)naphthalen-2-yl)phosphonate (5c)

Physical state: yellow oil.

Yield: *(R)*-**5c**, 31.5 mg, 52%.

^{1}H NMR (500 MHz, Chloroform-*d*) δ 8.24 – 8.19 (m, 1H), 8.08 (dd, *J* = 8.6, 3.6 Hz, 1H), 7.84 (d, *J* = 8.3 Hz, 1H), 7.38 (t, *J* = 7.5 Hz, 1H), 7.27 (d, *J* = 8.2 Hz, 2H), 7.18 – 7.14 (m, 2H), 7.13 – 7.09 (m, 1H), 7.01 (t, *J* = 7.6 Hz, 1H), 6.28 – 6.23 (m, 1H), 5.15 (d, *J* = 8.6 Hz, 1H), 4.25 (s, 3H), 3.88 – 3.84 (m, 1H), 3.80 – 3.73 (m, 2H), 3.63 – 3.50 (m, 2H), 2.90 – 2.83 (m, 1H), 2.73 (s, 3H), 2.61 – 2.53 (m, 1H), 2.20 (d, *J* = 12.0 Hz, 1H), 1.84 (d, *J* = 11.1

Hz, 1H), 1.73 – 1.66 (m, 2H), 1.37 – 1.32 (m, 2H), 1.30 – 1.23 (m, 2H), 0.94 (t, *J* = 7.0 Hz, 3H), 0.84 (t, *J* = 7.1 Hz, 3H).

^{13}C NMR (126 MHz, Chloroform-*d*) δ 161.05, 147.10 (d, *J* = 9.5 Hz), 140.29, 140.10, 134.90 (d, *J* = 2.6 Hz), 133.86 (d, *J* = 15.7 Hz), 131.08 (d, *J* = 5.4 Hz), 129.39, 128.96 (d, *J* = 10.1 Hz), 128.72, 128.39, 128.04, 127.88, 127.78, 127.75, 127.40, 126.76, 126.28 (d, *J* = 188.6 Hz), 124.69, 124.26, 124.09, 121.46, 119.55, 119.00, 109.13, 67.97, 61.73 (d, *J* = 6.0 Hz), 61.46 (d, *J* = 6.0 Hz), 32.12, 31.93, 30.68, 27.19, 26.97, 25.48, 22.57, 15.89 (d, *J* = 6.7 Hz), 15.77 (d, *J* = 6.8 Hz).

^{31}P NMR (202 MHz, Chloroform-*d*) δ +17.99.

HPLC: *(R)*-**5c** 94% ee, Chiralpak AS-H column, hexane : isopropanol = 90 : 10, 1.0 mL/min, λ = 254 nm, t_R (major) = 7.73 min, t_R (minor) = 14.60 min.

HRMS (ESI-TOF): calcd for $C_{37}H_{40}N_2O_4P^+$ [M+H$^+$] 607.2720, found 607.2725.

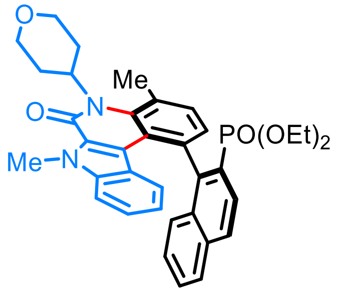

5d

diethyl *(R)*-(1-(4,7-dimethyl-6-oxo-5-(tetrahydro-2H-pyran-4-yl)-6,7-dihydro-5H-indolo[2,3-c]quinolin-1-yl)naphthalen-2-yl)phosphonate (5d)

Physical state: yellow oil.

Yield: *(R)*-**5d**, 25 mg, 41%.

^{1}H NMR (500 MHz, Chloroform-d) δ 8.19 (dd, *J* = 12.1, 8.6 Hz, 1H), 8.09 (dd, *J* = 8.6, 3.7 Hz, 1H), 7.86 (d, *J* = 8.2 Hz, 1H), 7.43 – 7.38 (m, 1H), 7.28 (dd, *J* = 10.3, 7.2 Hz, 2H), 7.18 (dd, *J* = 10.9, 8.1 Hz, 2H), 7.15 – 7.11 (m, 1H), 7.03 (t, *J* = 7.6 Hz, 1H), 6.26 (t, *J* = 7.8 Hz, 1H), 5.16 (d, *J* = 8.7 Hz, 1H), 4.26 (s, 3H), 4.19 – 4.14 (m, 1H), 4.13 – 4.05 (m, 2H), 3.84 – 3.70 (m, 2H), 3.64 – 3.51 (m, 2H), 3.50 – 3.44 (m, 1H), 3.43 – 3.37 (m, 1H), 3.33 – 3.22 (m, 1H), 3.03 – 2.91 (m, 1H), 2.73 (s, 3H), 2.07 (d, *J* = 12.7 Hz, 1H), 1.63 (d, *J* = 12.2 Hz, 1H), 0.93 (t, *J* = 7.1 Hz, 3H), 0.87 (t, *J* = 7.0 Hz, 3H).

^{13}C NMR (126 MHz, Chloroform-d) δ 161.09, 147.01 (d, *J* = 9.5 Hz), 140.38, 139.87, 134.95 (d, *J* = 2.5 Hz), 133.86 (d, *J* = 15.5 Hz), 131.37, 131.33, 129.25, 128.92 (d, *J* = 8.1 Hz), 128.48, 127.99, 127.89 (d, *J* = 16.1 Hz), 127.88, 127.74, 127.37, 126.83, 126.31 (d, *J* = 188.4 Hz), 124.87, 124.46, 124.11, 121.45, 119.68, 119.08, 109.23, 68.58, 68.35, 64.63, 61.74 (d, *J* = 5.9 Hz), 61.46 (d, *J* = 6.0 Hz), 32.31, 31.95, 31.01, 23.03, 15.89 (t, *J* = 6.8 Hz).

^{31}P NMR (202 MHz, Chloroform-*d*) δ +17.77.

HPLC: *(R)*-**5d** 91% ee, Chiralpak IA-3 column, hexane : isopropanol = 70 : 30, 0.5 mL/min, λ = 254 nm, t_R (major) = 39.69 min, t_R (minor) = 16.65 min.

HRMS (ESI-TOF): calcd for $C_{36}H_{38}N_2O_5P^+$ [M+H$^+$] 609.2513, found 609.2517.

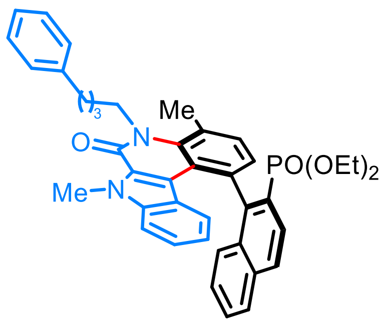

5e

diethyl (R)-(1-(4,7-dimethyl-6-oxo-5-(4-phenylbutyl)-6,7-dihydro-5H-indolo[2,3-c]quinolin-1-yl)naphthalen-2-yl)phosphonate (5e)

Physical state: white solid.

Yield: *(R)*-**5e**, 40.7 mg, 62%.

^{1}H NMR (500 MHz, Chloroform-*d*) δ 8.22 (dd, *J* = 12.0, 8.7 Hz, 1H), 8.09 (dd, *J* = 8.5, 3.3 Hz, 1H), 7.86 (d, *J* = 8.2 Hz, 1H), 7.38 (t, *J* = 7.4 Hz, 1H), 7.27 (d, *J* = 8.0 Hz, 1H), 7.26 – 7.09 (m, 7H), 7.05 (t, *J* = 6.9 Hz, 3H), 6.30 (t, *J* = 7.7 Hz, 1H), 5.21 (d, *J* = 8.6 Hz, 1H), 4.62 (dd, *J* = 13.9, 7.4 Hz, 1H), 4.50 (dd, *J* = 13.7, 6.3 Hz, 1H), 4.30 (s, 3H), 3.80 – 3.68 (m, 2H), 3.62 – 3.48 (m, 2H), 2.69 (s, 3H), 2.55 (t, *J* = 7.6 Hz, 2H), 1.71 – 1.62 (m, 2H), 1.56 – 1.46 (m, 2H), 0.92 (t, *J* = 7.1 Hz, 3H), 0.83 (t, *J* = 7.1 Hz, 3H).

^{13}C NMR (126 MHz, Chloroform-*d*) δ 159.57, 147.22 (d, *J* = 9.4 Hz), 141.84, 140.33, 137.07, 134.87 (d, *J* = 2.5 Hz), 133.79 (d, *J* = 15.5 Hz), 131.76 (d, *J* = 5.4 Hz), 128.94, 128.88, 128.71, 128.32, 128.22, 128.20, 127.89, 127.89 (d, *J* = 14.6 Hz), 127.77, 127.31, 126.81, 126.31 (d, *J* = 188.9 Hz), 125.67, 124.81, 124.26, 123.55, 121.59, 119.60, 118.95, 109.21, 61.76 (d, *J* = 6.0 Hz), 61.49 (d, *J* = 5.9 Hz), 48.36, 35.37, 31.85, 28.54, 27.98, 23.52, 15.94 (d, *J* = 6.7 Hz), 15.80 (d, *J* = 6.7 Hz).

^{31}P NMR (202 MHz, Chloroform-*d*) δ +17.89.

HPLC: *(R)*-**5e** 87% ee, Chiralpak AD-H column, hexane : isopropanol = 80 : 20, 1.0 mL/min, λ = 254 nm, t_R (major) = 21.58 min, t_R (minor) = 33.68 min.

HRMS (ESI-TOF): calcd for $C_{41}H_{42}N_2O_4P^+$ [M+H$^+$] 657.2877, found 657.2873.

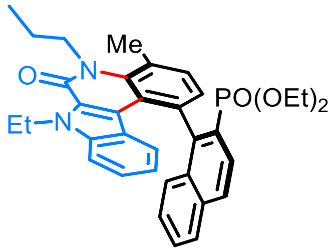

5f

diethyl (R)-(1-(7-ethyl-4-methyl-6-oxo-5-propyl-6,7-dihydro-5H-indolo[2,3-c]quinolin-1-yl)naphthalen-2-yl)phosphonate (5f)

Physical state: yellow solid.

Yield: *(R)*-**5f**, 29.6 mg, 51%.

^{1}H NMR (500 MHz, Chloroform-d) δ 8.23 (dd, *J* = 12.1, 8.6 Hz, 1H), 8.11 (dd, *J* = 8.6, 3.6 Hz, 1H), 7.89 (d, *J* = 8.2 Hz, 1H), 7.67 (s, 1H), 7.55 – 7.45 (m, 3H), 7.44 – 7.41 (m, 1H),

7.38 (t, J = 7.4 Hz, 1H), 7.32 (s, 1H), 7.27 – 7.19 (m, 3H), 7.15 (d, J = 7.7 Hz, 1H), 7.05 (t, J = 7.6 Hz, 1H), 6.33 – 6.28 (m, 1H), 5.39 (d, J = 8.7 Hz, 1H), 4.93 – 4.79 (m, 2H), 3.89 – 3.76 (m, 2H), 3.66 – 3.58 (m, 2H), 1.87 (s, 3H), 1.43 (t, J = 7.1 Hz, 3H), 0.98 (t, J = 7.1 Hz, 3H), 0.92 (t, J = 7.1 Hz, 3H).

^{13}C NMR (126 MHz, Chloroform-d) δ 157.35, 147.27 (d, J = 9.5 Hz), 142.07, 139.38, 137.27, 134.88 (d, J = 2.6 Hz), 133.70 (d, J = 15.5 Hz), 131.62 (d, J = 5.3 Hz), 129.78, 129.07 (d, J = 2.1 Hz), 128.83 (d, J = 9.8 Hz), 128.49, 128.22, 127.84 (d, J = 4.6 Hz), 127.80, 127.68, 127.42, 126.92, 126.75, 126.36, 126.25 (d, J = 188.5 Hz), 124.85, 124.33, 122.93, 121.74, 119.96, 119.44, 109.22, 61.59 (d, J = 6.0 Hz), 61.37 (d, J = 5.9 Hz), 39.66, 23.18, 15.81 (d, J = 6.9 Hz), 15.74 (d, J = 6.9 Hz), 15.55.

^{31}P NMR (202 MHz, Chloroform-*d*) δ +17.81.

HPLC: *(R)*-**5f** 90% ee, Chiralpak AD-H column, hexane : isopropanol = 80 : 20, 1.0 mL/min, λ = 254 nm, t_R (major) = 8.97 min, t_R (minor) = 12.61 min.

HRMS (ESI-TOF): calcd for $C_{35}H_{38}N_2O_4P^+$ [M+H$^+$] 581.2564, found 581.2565.

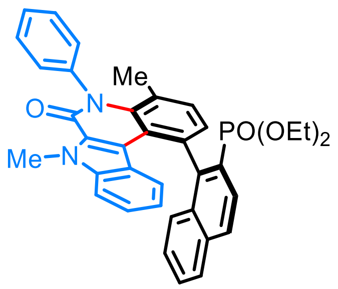

5g

diethyl *(R)-(*1-(4,7-dimethyl-6-oxo-5-phenyl-6,7-dihydro-5H-indolo[2,3-c]quinolin-1-yl)naphthalen-2-yl)phosphonate (5g)

Physical state: white solid.

Yield: *(R)*-**5g**, 43.8 mg, 73%.

^{1}H NMR (500 MHz, Chloroform-d) δ 8.21 (dd, J = 12.1, 8.6 Hz, 1H), 8.12 – 8.08 (m, 1H), 7.88 (d, J = 8.1 Hz, 1H), 7.65 (s, 1H), 7.58 – 7.36 (m, 6H), 7.24 – 7.17 (m, 3H), 7.14 (d, J = 7.7 Hz, 1H), 7.09 – 7.04 (m, 1H), 6.31 (t, J = 7.2 Hz, 1H), 5.38 (d, J = 8.7 Hz, 1H), 4.28 (s, 3H), 3.88 – 3.77 (m, 2H), 3.69 – 3.60 (m, 2H), 1.86 (s, 3H), 0.98 (t, J = 7.0 Hz, 3H), 0.93 (t, J = 7.0 Hz, 3H).

^{13}C NMR (126 MHz, Chloroform-d) δ 158.08, 147.35 (d, J = 9.5 Hz), 142.13, 140.61, 137.28, 134.99 (d, J = 2.5 Hz), 133.73 (d, J = 15.5 Hz), 131.88 (d, J = 5.5 Hz), 129.35, 129.33, 128.99 (d, J = 9.8 Hz), 128.07, 127.91 (d, J = 16.5 Hz), 127.44, 127.13, 126.88, 126.40 (d, J = 196.1 Hz), 125.07, 124.34, 123.06, 121.76, 109.29, 61.79 (d, J = 6.0 Hz), 61.59 (d, J = 6.0 Hz), 31.88, 23.40, 15.95 (t, J = 6.9 Hz).

^{31}P NMR (202 MHz, Chloroform-*d*) δ +17.83.

HPLC: *(R)*-**5g** 93% ee, Chiralpak OD-H column, hexane : isopropanol = 80 : 20, 1.0 mL/min, λ = 254 nm, t_R (major) = 7.94 min, t_R (minor) = 20.61 min.

HRMS (ESI-TOF): calcd for $C_{37}H_{34}N_2O_4P^+$ [M+H$^+$] 601.2251, found 601.2247.

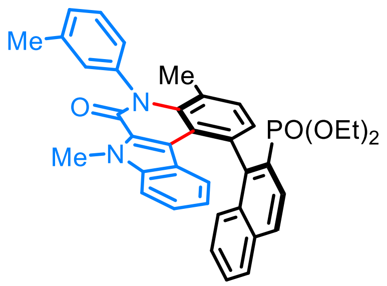

5h

diethyl *(R)-(*1-(4,7-dimethyl-6-oxo-5-(m-tolyl)-6,7-dihydro-5H-indolo[2,3-c]quinolin-1-yl)naphthalen-2-yl)phosphonate (5h)

Physical state: yellow solid.

Yield: *(R)*-**5h**, 41.2 mg, 67%.

^{1}H NMR (500 MHz, Chloroform-d) δ 8.22 (dd, *J* = 12.2, 8.6 Hz, 1H), 8.10 (dd, *J* = 8.7, 3.7 Hz, 1H), 7.88 (d, *J* = 8.2 Hz, 1H), 7.49 – 7.31 (m, 4H), 7.25 – 6.99 (m, 7H), 6.31 (t, *J* = 7.8 Hz, 1H), 5.40 (d, *J* = 8.7 Hz, 1H), 4.28 (s, 3H), 3.90 – 3.76 (m, 2H), 3.71 – 3.59 (m, 2H), 2.43 (d, *J* = 52.0 Hz, 3H), 1.88 (s, 3H), 0.99 (t, *J* = 7.2 Hz, 3H), 0.93 (t, *J* = 7.1 Hz, 3H).

^{13}C NMR (126 MHz, Chloroform-d) δ 158.16, 147.38 (d, *J* = 9.4 Hz), 141.99, 140.60, 137.30, 134.99 (d, *J* = 2.6 Hz), 133.73 (d, *J* = 15.5 Hz), 131.87 (d, *J* = 5.4 Hz), 129.37, 129.27, 129.00 (d, *J* = 10.0 Hz), 128.13, 127.96, 127.89 (d, *J* = 14.2 Hz), 127.45, 127.23, 126.86, 126.37 (d, *J* = 188.0 Hz), 125.04, 124.34, 122.98, 121.77, 119.99, 119.65, 109.27, 61.79 (d, *J* = 6.0 Hz), 61.59 (d, *J* = 5.9 Hz), 31.89, 23.49, 15.99 (d, *J* = 6.7 Hz), 15.92 (d, *J* = 6.8 Hz).

^{31}P NMR (202 MHz, Chloroform-*d*) δ +17.88.

HPLC: *(R)*-**5h** 93% ee, Chiralpak AS-H column, hexane : isopropanol = 70 : 30, 0.5 mL/min, λ = 254 nm, t_R (major) = 9.65 min, t_R (minor) = 13.27 min.

HRMS (ESI-TOF): calcd for $C_{38}H_{36}N_2O_4P^+$ [M+H$^+$] 615.2407, found 615.2410.

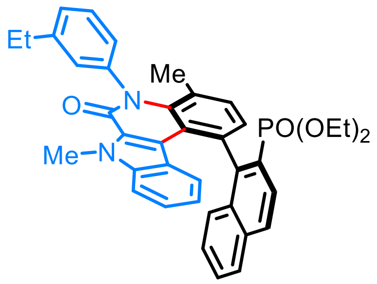

5i

diethyl *(R)-(*1-(5-(3-ethylphenyl)-4,7-dimethyl-6-oxo-6,7-dihydro-5H-indolo[2,3-c]quinolin-1-yl)naphthalen-2-yl)phosphonate (5i)

Physical state: light yellow solid.

Yield: *(R)*-**5i**, 48.4 mg, 77%.

^{1}H NMR (500 MHz, Chloroform-d) δ 8.29 – 8.21 (m, 1H), 8.12 (dd, *J* = 8.6, 3.7 Hz, 1H), 7.90 (d, *J* = 8.2 Hz, 1H), 7.53 – 7.38 (m, 4H), 7.27 – 7.15 (m, 5H), 7.14 – 7.04 (m, 2H), 6.33 (t, *J* = 7.5 Hz, 1H), 5.42 (d, *J* = 8.6 Hz, 1H), 4.31 (s, 3H), 3.91 – 3.78 (m, 2H), 3.71 –

3.61 (m, 2H), 2.84 – 2.65 (m, 2H), 1.89 (s, 3H), 1.34 – 1.22 (m, 3H), 1.05 – 0.98 (m, 3H), 0.95 (t, *J* = 7.0 Hz, 3H).

^{13}C NMR (126 MHz, Chloroform-d) δ 158.15, 147.40 (d, *J* = 9.4 Hz), 142.02, 140.60, 137.34, 135.00 (d, *J* = 2.5 Hz), 133.74 (d, *J* = 15.7 Hz), 131.87 (d, *J* = 5.2 Hz), 129.37, 129.28, 129.03 (d, *J* = 10.0 Hz), 128.16, 127.95, 127.90 (d, *J* = 16.5 Hz), 127.46, 126.86, 126.68, 126.37 (d, *J* = 188.0 Hz), 125.04, 124.35, 121.78, 119.99, 119.66, 109.28, 61.80 (d, *J* = 6.1 Hz), 61.59 (d, *J* = 5.9 Hz), 31.90, 28.68, 23.37, 16.01 (d, *J* = 5.0 Hz), 15.92 (d, *J* = 6.8 Hz).

^{31}P NMR (202 MHz, Chloroform-*d*) δ +18.01.

HPLC: *(R)*-**5i** 90% ee, Chiralpak IA-3 column, hexane : isopropanol = 70 : 30, 0.5 mL/min, λ = 254 nm, t_R (major) = 11.18 min, t_R (minor) = 12.85 min.

HRMS (ESI-TOF): calcd for $C_{39}H_{38}N_2O_4P^+$ [M+H$^+$] 629.2564, found 629.2567.

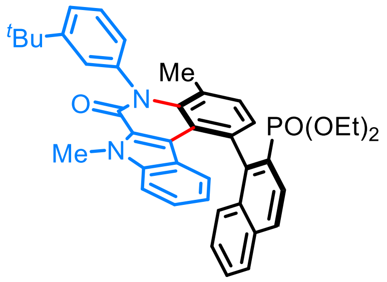

5j

diethyl *(R)*-(1-(5-(3-(tert-butyl)phenyl)-4,7-dimethyl-6-oxo-6,7-dihydro-5H-indolo[2,3-c]quinolin-1-yl)naphthalen-2-yl)phosphonate (5j)

Physical state: white solid.

Yield: *(R)*-**5j**, 44.0 mg, 67%.

^{1}H NMR (500 MHz, Chloroform-d) δ 8.29 – 8.18 (m, 1H), 8.10 (dd, *J* = 8.1, 2.9 Hz, 1H), 7.87 (t, *J* = 8.9 Hz, 1H), 7.61 (d, *J* = 8.9 Hz, 1H), 7.51 – 7.32 (m, 5H), 7.18 (dd, *J* = 37.9, 6.3 Hz, 5H), 7.07 (d, *J* = 7.5 Hz, 1H), 6.31 (t, *J* = 7.7 Hz, 1H), 5.41 (d, *J* = 8.5 Hz, 1H), 4.29 (s, 3H), 3.88 – 3.77 (m, 2H), 3.73 – 3.60 (m, 2H), 1.83 (s, 3H), 1.40 (s, 3H), 1.28 (s, 6H), 1.05 – 0.96 (m, 3H), 0.92 (t, *J* = 7.0 Hz, 3H).

^{13}C NMR (126 MHz, Chloroform-*d*) δ 158.13, 151.96, 151.56, 147.49 (d, *J* = 9.3 Hz), 141.85, 140.65, 137.44 (d, *J* = 18.7 Hz), 135.05, 133.79 (d, *J* = 15.5 Hz), 131.91 (d, *J* = 5.4 Hz), 129.41 (d, *J* = 8.9 Hz), 129.19 (d, *J* = 12.8 Hz), 128.30, 128.19, 128.13, 128.01, 127.88, 127.81, 127.45, 126.88, 126.88 (d, *J* = 191.8 Hz), 125.48, 125.08, 124.41, 121.83, 120.02, 119.72, 109.34, 61.89 (d, *J* = 4.6 Hz), 61.66 (d, *J* = 6.0 Hz), 34.77, 31.99, 31.34, 31.24, 23.30, 16.19 (d, *J* = 6.6 Hz), 16.01 (d, *J* = 5.9 Hz).

^{31}P NMR (202 MHz, Chloroform-*d*) δ +18.00 (d, *J* = 58.1 Hz).

HPLC: *(R)*-**5j** 90% ee, Chiralpak IA-3 column, hexane : isopropanol = 70 : 30, 0.5 mL/min, λ = 254 nm, t_R (major) = 9.61 min, t_R (minor) = 11.79 min.

HRMS (ESI-TOF): calcd for $C_{41}H_{42}N_2O_4P^+$ [M+H$^+$] 657.2877, found 657.2879.

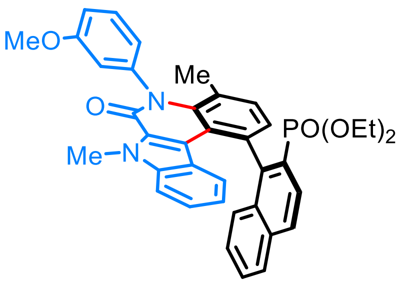

5k

diethyl *(R)-(*1-(5-(3-methoxyphenyl)-4,7-dimethyl-6-oxo-6,7-dihydro-5H-indolo[2,3-c] quinolin-1-yl)naphthalen-2-yl)phosphonate (5k)

Physical state: white solid.

Yield: *(R)*-**5k**, 39.7 mg, 63%.

^{1}H NMR (500 MHz, Chloroform-d) δ 8.22 (dd, *J* = 12.2, 8.6 Hz, 1H), 8.09 (dd, *J* = 8.6, 3.7 Hz, 1H), 7.88 (d, *J* = 8.1 Hz, 1H), 7.46 – 7.33 (m, 3H), 7.25 – 7.12 (m, 5H), 7.09 – 7.04 (m, 1H), 6.94 (dd, *J* = 8.3, 2.5 Hz, 1H), 6.85 (s, 1H), 6.31 (t, *J* = 7.5 Hz, 1H), 5.39 (d, *J* = 8.6 Hz, 1H), 4.28 (s, 3H), 3.92 – 3.81 (m, 3H), 3.81 – 3.75 (m, 2H), 3.68 – 3.58 (m, 2H), 1.94 (s, 3H), 0.99 (t, *J* = 7.1 Hz, 3H), 0.92 (t, *J* = 7.0 Hz, 3H).

^{13}C NMR (126 MHz, Chloroform-d) δ 158.10, 147.34 (d, *J* = 9.5 Hz), 143.11, 140.61, 137.23, 135.00 (d, *J* = 2.6 Hz), 133.73 (d, *J* = 15.7 Hz), 131.87 (d, *J* = 5.3 Hz), 129.36, 129.34, 129.02 (d, *J* = 10.0 Hz), 128.25, 127.95, 127.90 (d, *J* = 16.5 Hz), 127.45, 127.18, 126.87, 126.39 (d, *J* = 188.6 Hz), 125.07, 124.35, 123.05, 121.76, 120.00, 119.69, 112.65, 109.29, 61.78 (d, *J* = 6.0 Hz), 61.59 (d, *J* = 6.0 Hz), 31.89, 23.31, 15.99 (d, *J* = 6.8 Hz), 15.92 (d, *J* = 6.8 Hz).

^{31}P NMR (202 MHz, Chloroform-*d*) δ +17.86.

HPLC: *(R)-***5k** 88% ee, Chiralpak IA-3 column, hexane : isopropanol = 70 : 30, 0.5 mL/min, λ = 254 nm, t_R (major) = 13.92 min, t_R (minor) = 15.69 min.

HRMS (ESI-TOF): calcd for $C_{38}H_{36}N_2O_5P^+$ [M+H$^+$] 631.2356, found 631.2351.

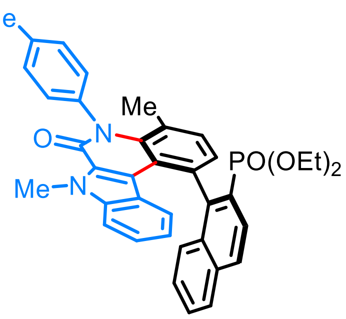

5l

diethyl *(R)-(*1-(4,7-dimethyl-6-oxo-5-(p-tolyl)-6,7-dihydro-5H-indolo[2,3-c]quinolin-1-yl)naphthalen-2-yl)phosphonate (5l)

Physical state: white solid.

Yield: *(R)*-**5l**, 39.3 mg, 64%.

^{1}H NMR (500 MHz, Chloroform-d) δ 8.21 (dd, *J* = 12.1, 8.6 Hz, 1H), 8.10 (dd, *J* = 8.6, 3.7 Hz, 1H), 7.88 (d, *J* = 8.2 Hz, 1H), 7.51 (s, 1H), 7.48 – 7.28 (m, 4H), 7.23 – 7.12 (m, 5H),

7.06 (t, J = 7.7 Hz, 1H), 6.30 (t, J = 7.8 Hz, 1H), 5.38 (d, J = 8.7 Hz, 1H), 4.28 (s, 3H), 3.86 – 3.77 (m, 2H), 3.67 – 3.59 (m, 2H), 2.44 (s, 3H), 1.89 (s, 3H), 0.98 (t, J = 7.1 Hz, 3H), 0.92 (t, J = 7.0 Hz, 3H).

^{13}C NMR (126 MHz, Chloroform-d) δ 158.23, 147.43 (d, J = 9.6 Hz), 140.60, 139.49, 137.38, 137.05, 135.00 (d, J = 2.5 Hz), 133.76 (d, J = 15.6 Hz), 131.87 (d, J = 5.3 Hz), 129.34, 129.21, 129.01 (d, J = 9.8 Hz), 128.00 (d, J = 14.8 Hz), 127.97, 127.84, 127.49, 127.24, 126.88, 126.39 (d, J = 189.0 Hz), 125.02, 124.37, 122.95, 121.80, 119.98, 119.65, 109.28, 61.80 (d, J = 6.0 Hz), 61.60 (d, J = 5.9 Hz), 31.87, 23.60, 21.19, 15.99 (d, J = 6.8 Hz), 15.91 (d, J = 6.8 Hz).

^{31}P NMR (202 MHz, Chloroform-*d*) δ +17.87.

HPLC: *(R)*-**5l** 90% ee, Chiralpak AS-H column, hexane : isopropanol = 70 : 30, 0.5 mL/min, λ = 254 nm, t_R (major) = 9.46 min, t_R (minor) = 12.79 min.

HRMS (ESI-TOF): calcd for $C_{38}H_{36}N_2O_4P^+$ [M+H$^+$] 615.2407, found 615.2403.

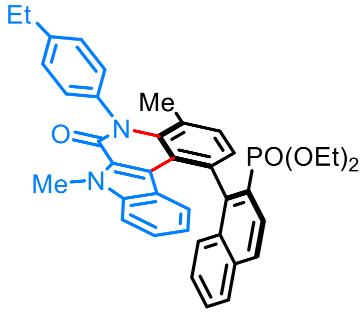

5m

diethyl *(R)*-(1-(5-(4-ethylphenyl)-4,7-dimethyl-6-oxo-6,7-dihydro-5H-indolo[2,3-c]quinolin-1-yl)naphthalen-2-yl)phosphonate (5m)

Physical state: white solid.

Yield: *(R)*-**5m**, 44.6 mg, 71%.

^{1}H NMR (500 MHz, Chloroform-d) δ 8.22 (dd, J = 12.1, 8.6 Hz, 1H), 8.10 (dd, J = 8.5, 3.7 Hz, 1H), 7.88 (d, J = 8.1 Hz, 1H), 7.54 (s, 1H), 7.46 – 7.28 (m, 4H), 7.24 – 7.12 (m, 5H), 7.06 (t, J = 7.3 Hz, 1H), 6.30 (t, J = 8.2 Hz, 1H), 5.38 (d, J = 8.6 Hz, 1H), 4.28 (s, 3H), 3.88 – 3.78 (m, 2H), 3.68 – 3.59 (m, 2H), 2.74 (q, J = 7.6 Hz, 2H), 1.87 (s, 3H), 1.30 (t, J = 7.6 Hz, 3H), 0.99 (t, J = 7.1 Hz, 3H), 0.92 (t, J = 7.0 Hz, 3H).

^{13}C NMR (126 MHz, Chloroform-d) δ 158.22, 147.42 (d, J = 9.5 Hz), 143.31, 140.60, 139.64, 137.41, 135.00 (d, J = 2.6 Hz), 133.76 (d, J = 15.5 Hz), 131.85 (d, J = 5.4 Hz), 129.33, 129.22, 129.02 (d, J = 9.9 Hz), 128.12, 127.94, 127.90 (d, J = 16.5 Hz), 127.48, 127.25, 126.87, 126.39 (d, J = 188.7 Hz), 125.02, 124.37, 122.96, 121.79, 119.97, 119.65, 109.28, 61.79 (d, J = 5.9 Hz), 61.60 (d, J = 5.9 Hz), 31.88, 28.54, 23.54, 16.00 (d, J = 6.8 Hz), 15.92 (d, J = 6.8 Hz), 15.52.

^{31}P NMR (202 MHz, Chloroform-*d*) δ +17.88.

HPLC: *(R)*-**5m** 90% ee, Chiralpak IA-3 column, hexane : isopropanol = 70 : 30, 0.5 mL/min, λ = 254 nm, t_R (major) = 13.28 min, t_R (minor) = 15.70 min.

HRMS (ESI-TOF): calcd for $C_{39}H_{38}N_2O_4P^+$ [M+H$^+$] 629.2564, found 629.2569.

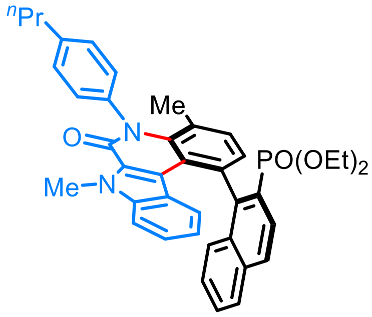

5n

diethyl *(R)-(*1-(4,7-dimethyl-6-oxo-5-(4-propylphenyl)-6,7-dihydro-5H-indolo[2,3-c]quinolin-1-yl)naphthalen-2-yl)phosphonate (5n)

Physical state: white solid.

Yield: *(R)*-**5n**, 43.2 mg, 71%.

^{1}H NMR (500 MHz, Chloroform-d) δ 8.22 (dd, J = 12.1, 8.6 Hz, 1H), 8.10 (dd, J = 8.6, 3.7 Hz, 1H), 7.87 (d, J = 8.2 Hz, 1H), 7.53 (s, 1H), 7.47 – 7.27 (m, 4H), 7.24 – 7.12 (m, 5H), 7.06 (t, J = 7.6 Hz, 1H), 6.31 (t, J = 7.8 Hz, 1H), 5.39 (d, J = 8.7 Hz, 1H), 4.28 (s, 3H), 3.89 – 3.76 (m, 2H), 3.71 – 3.60 (m, 2H), 2.68 (t, J = 7.6 Hz, 2H), 1.87 (s, 3H), 1.72 (q, J = 7.5 Hz, 2H), 1.04 – 0.96 (m, 6H), 0.93 (t, J = 7.1 Hz, 3H).

^{13}C NMR (126 MHz, Chloroform-d) δ 158.17, 147.40 (d, J = 9.3 Hz), 141.71, 140.58, 139.67, 137.40, 134.97 (d, J = 2.6 Hz), 133.73 (d, J = 15.5 Hz), 131.83 (d, J = 5.4 Hz), 129.32, 129.20, 129.00 (d, J = 9.9 Hz), 128.35, 128.11, 127.92, 127.87 (d, J = 15.9 Hz), 127.44, 127.24, 126.84, 126.38 (d, J = 188.6 Hz), 124.99, 124.34, 122.95, 121.77, 109.26, 61.77 (d, J = 6.0 Hz), 61.57 (d, J = 6.0 Hz), 37.63, 31.86, 24.43, 23.46, 15.98 (d, J = 6.7 Hz), 15.91 (d, J = 6.8 Hz), 13.76.

^{31}P NMR (202 MHz, Chloroform-*d*) δ +17.88.

HPLC: *(R)*-**5n** 88% ee, Chiralpak IA-3 column, hexane : isopropanol = 70 : 30, 0.5 mL/min, λ = 254 nm, t_R (major) = 14.92 min, t_R (minor) = 16.22 min.

HRMS (ESI-TOF): calcd for $C_{40}H_{40}N_2O_4P^+$ [M+H$^+$] 643.2720, found 643.2717.

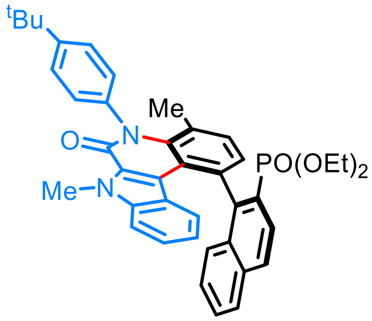

5o

diethyl *(R)-(*1-(5-(4-(tert-butyl)phenyl)-4,7-dimethyl-6-oxo-6,7-dihydro-5H-indolo[2,3-c]quinolin-1-yl)naphthalen-2-yl)phosphonate (5o)

Physical state: light yellow solid.

Yield: *(R)*-**5o**, 40.0 mg, 61%.

^{1}**H NMR** (500 MHz, Chloroform-d) δ 8.23 (dd, *J* = 12.1, 8.6 Hz, 1H), 8.10 (dd, *J* = 8.6, 3.7 Hz, 1H), 7.87 (d, *J* = 8.2 Hz, 1H), 7.56 – 7.40 (m, 5H), 7.23 – 7.12 (m, 5H), 7.06 (t, *J* = 7.6 Hz, 1H), 6.31 (t, *J* = 7.8 Hz, 1H), 5.38 (d, *J* = 8.7 Hz, 1H), 4.29 (s, 3H), 3.87 – 3.79 (m, 2H), 3.68 – 3.62 (m, 2H), 1.86 (s, 3H), 1.39 (s, 9H), 1.00 (t, *J* = 7.1 Hz, 3H), 0.93 (t, *J* = 7.0 Hz, 3H).

^{13}**C NMR** (126 MHz, Chloroform-d) δ 158.18, 150.14, 147.39 (d, *J* = 9.4 Hz), 140.58, 139.37, 137.40, 134.97 (d, *J* = 2.6 Hz), 133.74 (d, *J* = 15.5 Hz), 131.81 (d, *J* = 5.2 Hz), 129.29, 129.22, 129.00 (d, *J* = 9.9 Hz), 128.18, 127.87 (d, *J* = 15.6 Hz), 127.43, 127.24, 126.83, 126.36 (d, *J* = 188.9 Hz), 125.35, 125.00, 124.34, 122.97, 121.76, 119.94, 119.63, 109.26, 61.78 (d, *J* = 6.1 Hz), 61.59 (d, *J* = 6.0 Hz), 34.63, 31.86, 31.37, 23.45, 16.00 (d, *J* = 6.7 Hz), 15.91 (d, *J* = 6.7 Hz).

^{31}**P NMR** (202 MHz, Chloroform-*d*) δ +17.89.

HPLC: *(R)*-**5o** 90% ee, Chiralpak IA-3 column, hexane : isopropanol = 70 : 30, 0.5 mL/min, λ = 254 nm, t_R (major) = 10.96 min, t_R (minor) = 14.87 min.

HRMS (ESI-TOF): calcd for $C_{41}H_{42}N_2O_4P^+$ [M+H$^+$] 657.2877, found 657.2880.

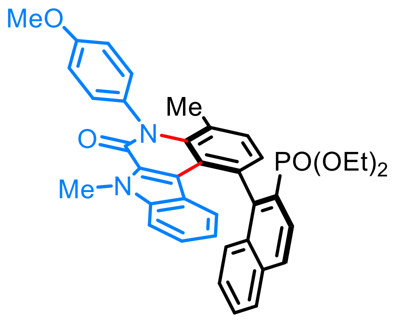

5p

diethyl *(R)*-(1-(5-(4-methoxyphenyl)-4,7-dimethyl-6-oxo-6,7-dihydro-5H-indolo[2,3-c] quinolin-1-yl)naphthalen-2-yl)phosphonate (5p)

Physical state: colorless oil.

Yield: *(R)*-**5p**, 41.0 mg, 65%.

1H NMR (500 MHz, Chloroform-d) δ 8.21 (dd, *J* = 12.1, 8.5 Hz, 1H), 8.09 (dd, *J* = 8.7, 3.6 Hz, 1H), 7.88 (d, *J* = 8.2 Hz, 1H), 7.53 (s, 1H), 7.44 (q, *J* = 8.0, 7.6 Hz, 2H), 7.25 – 7.12 (m, 5H), 7.06 (t, *J* = 7.7 Hz, 2H), 6.98 (s, 1H), 6.30 (t, *J* = 7.8 Hz, 1H), 5.38 (d, *J* = 8.7 Hz, 1H), 4.28 (s, 3H), 3.88 (s, 3H), 3.85 – 3.74 (m, 2H), 3.68 – 3.57 (m, 2H), 1.90 (s, 3H), 0.98 (t, *J* = 7.1 Hz, 3H), 0.93 (t, *J* = 7.1 Hz, 3H).

^{13}**C NMR** (126 MHz, Chloroform-d) δ 158.36, 158.33, 147.42 (d, *J* = 9.4 Hz), 140.58, 137.47, 134.98 (d, *J* = 2.6 Hz), 134.89, 133.73 (d, *J* = 15.5 Hz), 131.89 (d, *J* = 5.4 Hz), 130.78, 129.63, 129.38, 129.13, 128.98 (d, *J* = 9.9 Hz), 127.89 (d, *J* = 15.9 Hz), 127.46, 127.20, 126.86, 126.37 (d, *J* = 188.8 Hz), 125.01, 124.35, 122.85, 121.78, 119.96, 119.63, 113.71, 109.27, 61.76 (d, *J* = 6.0 Hz), 61.57 (d, *J* = 5.8 Hz), 55.49, 15.94 (t, *J* = 7.0 Hz).

^{31}**P NMR** (202 MHz, Chloroform-*d*) δ +17.84.

HPLC: *(R)*-**5p** 90% ee, Chiralpak IA-3 column, hexane : isopropanol = 70 : 30, 0.5 mL/min, λ = 254 nm, t_R (major) = 16.05 min, t_R (minor) = 21.43 min.

HRMS (ESI-TOF): calcd for $C_{38}H_{36}N_2O_5P^+$ [M+H$^+$] 631.2356, found 631.2353.

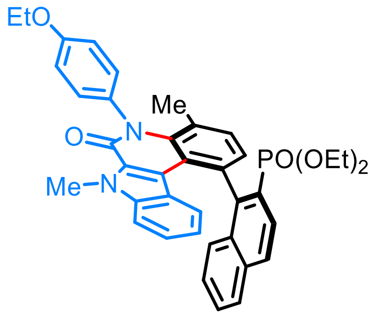

5q

diethyl *(R)-(*1-(5-(4-ethoxyphenyl)-4,7-dimethyl-6-oxo-6,7-dihydro-5H-indolo[2,3-c]quinolin-1-yl)naphthalen-2-yl)phosphonate (5q)

Physical state: yellow oil.

Yield: *(R)*-**5q**, 43.8 mg, 68%.

^{1}H NMR (500 MHz, Chloroform-d) δ 8.21 (dd, *J* = 12.1, 8.6 Hz, 1H), 8.09 (dd, *J* = 8.6, 3.7 Hz, 1H), 7.88 (d, *J* = 8.1 Hz, 1H), 7.51 (s, 1H), 7.43 (dd, *J* = 18.1, 7.9 Hz, 2H), 7.23 – 7.13 (m, 5H), 7.09 – 7.02 (m, 2H), 6.97 (s, 1H), 6.30 (t, *J* = 8.4 Hz, 1H), 5.39 (d, *J* = 8.6 Hz, 1H), 4.28 (s, 3H), 4.11 (q, *J* = 7.0 Hz, 2H), 3.87 – 3.77 (m, 2H), 3.67 – 3.60 (m, 2H), 1.91 (s, 3H), 1.46 (t, *J* = 7.0 Hz, 3H), 0.98 (t, *J* = 7.0 Hz, 3H), 0.93 (t, *J* = 7.0 Hz, 3H).

^{13}C NMR (126 MHz, Chloroform-d) δ 158.35, 157.72, 147.43 (d, *J* = 9.5 Hz), 140.57, 137.49, 134.97 (d, *J* = 2.7 Hz), 134.72, 133.73 (d, *J* = 15.6 Hz), 131.86 (d, *J* = 5.4 Hz), 130.75, 129.62, 129.37, 129.10, 128.97 (d, *J* = 10.0 Hz), 127.98, 127.92, 127.88 (d, *J* = 16.9 Hz), 127.46, 127.21, 126.85, 126.36 (d, *J* = 188.8 Hz), 124.99, 124.34, 122.83, 121.77, 119.94, 119.62, 114.23, 109.26, 63.70, 61.76 (d, *J* = 6.0 Hz), 61.57 (d, *J* = 5.9 Hz), 31.84, 23.63, 15.93 (t, *J* = 7.0 Hz), 14.79.

^{31}P NMR (202 MHz, Chloroform-*d*) δ +17.84.

HPLC: *(R)*-**5q** 88% ee, Chiralpak IA-3 column, hexane : isopropanol = 70 : 30, 0.5 mL/min, λ = 254 nm, t_R (major) = 15.10 min, t_R (minor) = 22.20 min.

HRMS (ESI-TOF): calcd for $C_{39}H_{38}N_2O_5P^+$ [M+H$^+$] 645.2513, found 645.2516.

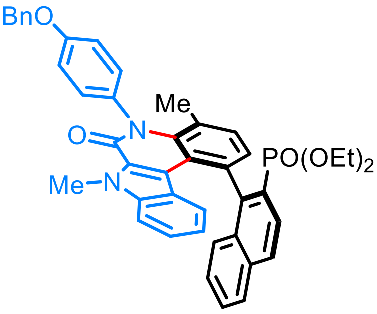

5r

diethyl *(R)-(*1-(5-(4-(benzyloxy)phenyl)-4,7-dimethyl-6-oxo-6,7-dihydro-5H-indolo[2,3-c]quinolin-1-yl)naphthalen-2-yl)phosphonate (5r)

Physical state: yellow solid.

Yield: *(R)*-**5r**, 39.6 mg, 56%.

^{1}H NMR (500 MHz, Chloroform-d) δ 8.21 (dd, *J* = 12.1, 8.6 Hz, 1H), 8.10 (dd, *J* = 8.6, 3.7 Hz, 1H), 7.88 (d, *J* = 8.2 Hz, 1H), 7.53 (s, 1H), 7.49 – 7.40 (m, 6H), 7.35 (t, *J* = 7.3 Hz, 1H), 7.24 – 7.12 (m, 6H), 7.07 (t, *J* = 7.7 Hz, 2H), 6.31 (t, *J* = 7.8 Hz, 1H), 5.39 (d, *J* = 8.6 Hz, 1H), 5.14 (s, 2H), 4.29 (s, 3H), 3.88 – 3.76 (m, 2H), 3.69 – 3.61 (m, 2H), 1.91 (s, 3H), 0.98 (t, *J* = 7.0 Hz, 3H), 0.93 (t, *J* = 7.0 Hz, 3H).
^{13}C NMR (126 MHz, Chloroform-d) δ 158.35, 157.57, 147.43 (d, *J* = 9.4 Hz), 140.60, 137.47, 136.68, 135.14, 135.00 (d, *J* = 2.6 Hz), 133.75 (d, *J* = 15.5 Hz), 130.80, 129.70, 129.40, 129.16, 128.99 (d, *J* = 9.9 Hz), 128.59, 128.04, 127.95, 127.91 (d, *J* = 16.5 Hz), 127.49, 127.20, 126.88, 126.39 (d, *J* = 188.6 Hz), 125.03, 124.37, 122.87, 121.79, 119.99, 119.65, 114.69, 70.32, 61.78 (d, *J* = 6.0 Hz), 61.58 (d, *J* = 5.9 Hz), 31.86, 23.67, 15.95 (t, *J* = 6.7 Hz).
^{31}P NMR (202 MHz, Chloroform-*d*) δ +17.81.
HPLC: *(R)*-**5r** 88% ee, Chiralpak IA-3 column, hexane : isopropanol = 70 : 30, 0.5 mL/min, λ = 254 nm, t_R (major) = 21.53 min, t_R (minor) = 36.87 min.
HRMS (ESI-TOF): calcd for $C_{44}H_{39}N_2O_5P^+$ [M+H$^+$] 707.2669, found 707.2665.

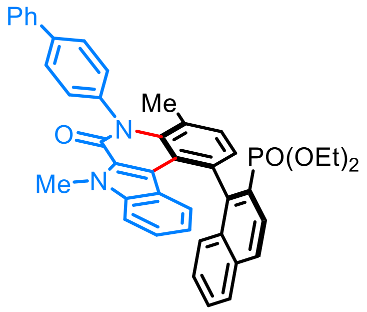

5s

diethyl *(R)*-(1-(5-([1,1'-biphenyl]-4-yl)-4,7-dimethyl-6-oxo-6,7-dihydro-5H-indolo[2,3-c]quinolin-1-yl)naphthalen-2-yl)phosphonate (5s)
Physical state: yellow solid.
Yield: *(R)*-**5s**, 38.6 mg, 57%.
^{1}H NMR (500 MHz, Chloroform-d) δ 8.23 (dd, *J* = 12.1, 8.6 Hz, 1H), 8.11 (dd, *J* = 8.6, 3.7 Hz, 1H), 7.90 (d, *J* = 8.2 Hz, 1H), 7.78 – 7.66 (m, 5H), 7.50 – 7.37 (m, 6H), 7.26 – 7.21 (m, 3H), 7.19 (d, *J* = 6.9 Hz, 1H), 7.08 (t, *J* = 7.6 Hz, 1H), 6.32 (t, *J* = 7.8 Hz, 1H), 5.40 (d, *J* = 8.7 Hz, 1H), 4.30 (s, 3H), 3.89 – 3.79 (m, 2H), 3.69 – 3.63 (m, 2H), 1.96 (s, 3H), 1.00 (t, *J* = 7.1 Hz, 3H), 0.95 (t, *J* = 7.0 Hz, 3H).
^{13}C NMR (126 MHz, Chloroform-d) δ 158.17, 147.34 (d, *J* = 9.4 Hz), 141.31, 140.64, 140.28, 139.96, 137.27, 135.01 (d, *J* = 2.6 Hz), 133.76 (d, *J* = 15.5 Hz), 131.93 (d, *J* = 5.4 Hz), 129.40, 129.35, 128.99 (d, *J* = 10.0 Hz), 128.80, 128.13, 127.92 (d, *J* = 16.5 Hz), 127.50, 127.46, 127.14, 126.90, 126.40 (d, *J* = 185.3 Hz), 125.11, 124.36, 123.14, 121.77, 120.08, 119.72, 109.31, 61.79 (d, *J* = 6.0 Hz), 61.59 (d, *J* = 5.9 Hz), 31.90, 23.60, 15.97 (t, *J* = 6.9 Hz).
^{31}P NMR (202 MHz, Chloroform-*d*) δ +17.80.
HPLC: *(R)*-**5s** 88% ee, Chiralpak IA-3 column, hexane : isopropanol = 70 : 30, 0.5 mL/min, λ = 254 nm, t_R (major) = 16.95 min, t_R (minor) = 25.99 min.
HRMS (ESI-TOF): calcd for $C_{43}H_{38}N_2O_4P^+$ [M+H$^+$] 677.2564, found 677.2567.

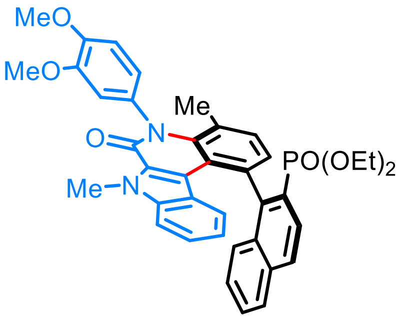

5t

diethyl *(R)-(*1-(5-(3,4-dimethoxyphenyl)-4,7-dimethyl-6-oxo-6,7-dihydro-5H-indolo[2,3-c]quinolin-1-yl)naphthalen-2-yl)phosphonate (5t)

Physical state: yellow oil.

Yield: *(R)*-**5t**, 42.0 mg, 63%.

^{1}H NMR (500 MHz, Chloroform-d) δ 8.19 (q, *J* = 10.6 Hz, 1H), 8.08 (dd, *J* = 8.5, 3.5 Hz, 1H), 7.88 (d, *J* = 6.2 Hz, 1H), 7.50 – 7.41 (m, 2H), 7.24 – 7.14 (m, 5H), 7.06 (t, *J* = 7.6 Hz, 1H), 7.02 – 6.89 (m, 1H), 6.88 – 6.73 (m, 1H), 6.30 (t, *J* = 7.8 Hz, 1H), 5.41 (t, *J* = 10.2 Hz, 1H), 4.28 (s, 3H), 3.98 – 3.94 (m, 4H), 3.87 – 3.60 (m, 6H), 1.94 (s, 3H), 1.00 – 0.92 (m, 6H).

^{13}C NMR (126 MHz, Chloroform-d) δ 158.46, 148.91, 148.56, 148.08 (d, *J* = 11.5 Hz), 147.43 (d, *J* = 12.7 Hz), 140.59, 137.46, 135.13, 135.01 (d, *J* = 2.2 Hz), 134.91, 133.71 (d, *J* = 15.0 Hz), 131.93 (d, *J* = 4.5 Hz), 129.38, 129.15, 128.11, 127.99, 127.94, 127.85, 127.83, 127.48, 127.18, 126.33 (d, *J* = 188.6 Hz), 125.05, 122.30, 121.77, 120.92, 119.66, 113.92, 112.69, 110.43 (d, *J* = 21.8 Hz), 109.29, 61.75, 61.51 (d, *J* = 9.5 Hz), 56.22, 56.04, 31.86, 23.48, 15.97 (d, *J* = 5.1 Hz), 15.92 (d, *J* = 4.1 Hz).

^{31}P NMR (202 MHz, Chloroform-*d*) δ +17.83.

HPLC: *(R)*-**5t** 85% ee, Chiralpak IA-3 column, hexane : isopropanol = 70 : 30, 0.5 mL/min, λ = 254 nm, t_R (major) = 14.91 min, t_R (minor) = 19.56 min.

HRMS (ESI-TOF): calcd for $C_{39}H_{38}N_2O_6P^+$ [M+H$^+$] 661.2462, found 661.2465.

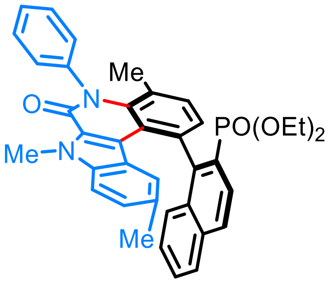

5u

diethyl *(R)-(*1-(4,7,9-trimethyl-6-oxo-5-phenyl-6,7-dihydro-5H-indolo[2,3-c]quinolin-1-yl)naphthalen-2-yl)phosphonate (5u)

Physical state: light yellow solid.

Yield: *(R)*-**5u**, 26.4 mg, 43%.

^{1}H NMR (500 MHz, Chloroform-d) δ 8.21 (dd, *J* = 12.0, 8.6 Hz, 1H), 8.12 (dd, *J* = 8.6, 3.5 Hz, 1H), 7.91 (d, *J* = 8.2 Hz, 1H), 7.64 (s, 1H), 7.56 – 7.45 (m, 4H), 7.38 (t, *J* = 7.3 Hz, 1H),

7.31 – 7.20 (m, 3H), 7.12 (dd, J = 13.5, 8.1 Hz, 2H), 6.91 (d, J = 8.4 Hz, 1H), 5.10 (s, 1H), 4.25 (s, 3H), 3.85 – 3.74 (m, 2H), 3.63 – 3.55 (m, 2H), 1.86 (s, 3H), 1.76 (s, 3H), 0.97 (t, J = 7.1 Hz, 3H), 0.92 (t, J = 7.0 Hz, 3H).
^{13}C NMR (126 MHz, Chloroform-d) δ 158.07, 147.51 (d, J = 9.5 Hz), 142.16, 139.04, 137.35, 135.04 (d, J = 2.6 Hz), 133.94 (d, J = 15.7 Hz), 131.68 (d, J = 5.4 Hz), 129.16, 129.15, 129.05, 128.92 (d, J = 9.9 Hz), 128.39, 128.01, 127.95, 127.85 (d, J = 14.4 Hz), 127.64, 127.07, 127.03, 126.92, 126.77, 126.42 (d, J = 188.5 Hz), 124.12, 123.22, 121.79, 119.51, 108.82, 61.67 (d, J = 5.9 Hz), 61.48 (d, J = 5.9 Hz), 31.83, 23.33, 20.60, 15.86 (t, J = 7.0 Hz), .
^{31}P NMR (202 MHz, Chloroform-*d*) δ +17.79.
HPLC: *(R)*-**5u** 90% ee, Chiralpak OD-H column, hexane : isopropanol = 80 : 20, 1.0 mL/min, λ = 254 nm, t_R (major) = 7.18 min, t_R (minor) = 18.82 min.
HRMS (ESI-TOF): calcd for $C_{38}H_{36}N_2O_4P^+$ [M+H$^+$] 615.2407, found 615.2409.

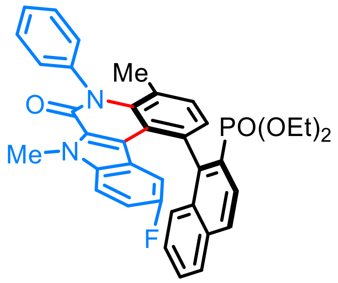

5v

diethyl *(R)*-(1-(9-fluoro-4,7-dimethyl-6-oxo-5-phenyl-6,7-dihydro-5H-indolo[2,3-c]quinolin-1-yl)naphthalen-2-yl)phosphonate (5v)
Physical state: light yellow solid.
Yield: *(R)*-**5v**, 32.8 mg, 53%.
^{1}H NMR (500 MHz, Chloroform-d) δ 8.24 – 8.12 (m, 2H), 7.92 (d, J = 8.2 Hz, 1H), 7.62 (s, 1H), 7.54 – 7.37 (m, 5H), 7.30 – 7.12 (m, 5H), 6.88 – 6.82 (m, 1H), 5.00 – 4.92 (m, 1H), 4.27 (s, 3H), 3.95 – 3.87 (m, 1H), 3.84 – 3.71 (m, 2H), 3.68 – 3.59 (m, 1H), 1.85 (s, 3H), 1.01 (t, J = 7.1 Hz, 3H), 0.97 (t, J = 7.1 Hz, 3H).
^{13}C NMR (126 MHz, Chloroform-d) δ 157.97, 156.82 (d, J = 234.4 Hz), 147.10 (d, J = 9.8 Hz), 142.02, 137.20 (d, J = 14.6 Hz), 135.01 (d, J = 2.7 Hz), 133.60 (d, J = 15.6 Hz), 131.79 (d, J = 5.4 Hz), 129.54, 129.38, 128.76 (d, J = 9.9 Hz), 128.34 (d, J = 14.2 Hz), 128.22, 128.11, 128.04, 127.23 (d, J = 3.1 Hz), 126.99, 126.50 (d, J = 188.6 Hz), 122.75, 121.90 (d, J = 10.4 Hz), 119.81 (d, J = 4.8 Hz), 113.88 (d, J = 26.6 Hz), 109.87 (d, J = 9.5 Hz), 109.50 (d, J = 27.6 Hz), 61.85 (d, J = 6.2 Hz), 61.63 (d, J = 5.9 Hz), 32.10, 23.38, 15.99 (d, J = 2.8 Hz), 15.94 (d, J = 2.7 Hz).
^{31}P NMR (202 MHz, Chloroform-*d*) δ +17.47.
^{19}F NMR (471 MHz, Chloroform-*d*) δ -123.50.
HPLC: *(R)*-**5v** 86% ee, Chiralpak IA-3 column, hexane : isopropanol = 70 : 30, 0.5 mL/min, λ = 254 nm, t_R (major) = 12.15 min, t_R (minor) = 16.90 min.
HRMS (ESI-TOF): calcd for $C_{37}H_{33}FN_2O_4P^+$ [M+H$^+$] 619.2156, found 619.2152.

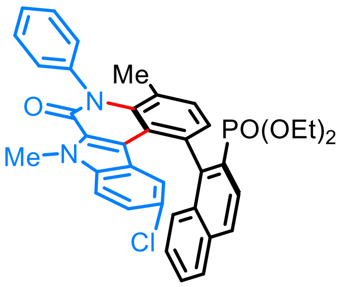

5w

diethyl *(R)-(*1-(9-chloro-4,7-dimethyl-6-oxo-5-phenyl-6,7-dihydro-5H-indolo[2,3-c]quinolin-1-yl)naphthalen-2-yl)phosphonate (5w)

Physical state: yellow solid.

Yield: *(R)*-**5w**, 36.2 mg, 57%.

^{1}**H NMR** (500 MHz, Chloroform-d) δ 8.17 (d, *J* = 8.3 Hz, 2H), 7.94 (d, *J* = 8.2 Hz, 1H), 7.61 (s, 1H), 7.53 (s, 1H), 7.46 (dd, *J* = 17.8, 8.1 Hz, 3H), 7.40 (t, *J* = 7.3 Hz, 1H), 7.29 (s, 1H), 7.26 – 7.20 (m, 2H), 7.18 – 7.13 (m, 2H), 7.06 – 7.01 (m, 1H), 5.22 (d, *J* = 1.7 Hz, 1H), 4.26 (s, 3H), 3.95 – 3.88 (m, 1H), 3.80 – 3.71 (m, 2H), 3.65 – 3.59 (m, 1H), 1.85 (s, 3H), 1.03 (t, *J* = 7.1 Hz, 3H), 0.96 (t, *J* = 7.1 Hz, 3H).

^{13}**C NMR** (126 MHz, Chloroform-d) δ 157.95, 146.84 (d, *J* = 9.5 Hz), 142.00, 138.89, 137.38, 135.13 (d, *J* = 2.7 Hz), 133.56 (d, *J* = 15.3 Hz), 131.99 (d, *J* = 5.6 Hz), 129.73, 129.34, 128.83, 128.72, 128.63 (d, *J* = 9.7 Hz), 128.14, 128.06, 128.04 (d, *J* = 17.4 Hz), 127.28, 127.20, 127.03, 126.45 (d, *J* = 190.1 Hz), 125.45, 125.31, 124.00, 122.66, 122.49, 119.53, 110.28, 61.91 (d, *J* = 6.0 Hz), 61.66 (d, *J* = 5.9 Hz), 32.10, 23.40, 16.01 (d, *J* = 6.6 Hz), 15.96 (d, *J* = 6.4 Hz). 131.99 (d, *J* = 5.6 Hz)

^{31}**P NMR** (202 MHz, Chloroform-*d*) δ +17.44.

HPLC: *(R)*-**5w** 88% ee, Chiralpak IA-3 column, hexane : isopropanol = 70 : 30, 0.5 mL/min, λ = 254 nm, t_R (major) = 12.37 min, t_R (minor) = 16.97 min.

HRMS (ESI-TOF): calcd for $C_{37}H_{33}ClN_2O_4P^+$ [M+H$^+$] 635.1861, found 635.1859.

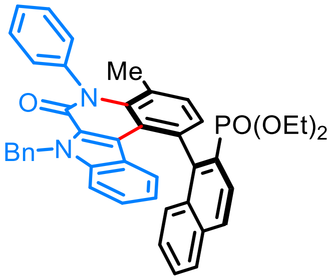

5x

diethyl *(R)-(*1-(7-benzyl-4-methyl-6-oxo-5-phenyl-6,7-dihydro-5H-indolo[2,3-c]quinolin-1-yl)naphthalen-2-yl)phosphonate (5x)

Physical state: yellow oil.

Yield: *(R)*-**5x**, 31.8 mg, 47%.

^{1}**H NMR** (500 MHz, Chloroform-d) δ 8.27 (dd, *J* = 12.0, 8.7 Hz, 1H), 8.17 – 8.12 (m, 1H), 7.91 (d, *J* = 8.2 Hz, 1H), 7.71 (s, 1H), 7.56 – 7.39 (m, 6H), 7.31 (d, *J* = 7.6 Hz, 1H), 7.26 – 7.18 (m, 6H), 7.09 (d, *J* = 7.3 Hz, 2H), 7.02 (t, *J* = 7.6 Hz, 1H), 6.38 – 6.30 (m, 2H), 6.11

(d, J = 16.3 Hz, 1H), 5.53 (d, J = 8.7 Hz, 1H), 3.94 – 3.83 (m, 2H), 3.77 – 3.66 (m, 2H), 1.90 (s, 3H), 1.05 – 0.99 (m, 6H).

^{13}C NMR (126 MHz, Chloroform-d) δ 157.85, 147.37 (d, J = 9.6 Hz), 142.22, 140.42, 138.31, 137.52, 135.12 (d, J = 2.4 Hz), 133.80 (d, J = 15.6 Hz), 132.11 (d, J = 5.3 Hz), 129.65, 129.57, 129.49, 129.08 (d, J = 9.8 Hz), 128.50, 128.32, 128.12, 128.02, 127.99, 127.58, 127.21, 127.05, 126.88, 126.52, 126.52 (d, J = 188.6 Hz), 125.44, 124.46, 123.14, 122.14, 120.99, 120.02, 110.21, 61.95 (d, J = 6.1 Hz), 61.74 (d, J = 5.9 Hz), 47.84, 23.46, 16.14, 16.09.

^{31}P NMR (202 MHz, Chloroform-*d*) δ +17.88.

HPLC: *(R)*-**5x** 92% ee, Chiralpak IA-3 column, hexane : isopropanol = 70 : 30, 0.5 mL/min, λ = 254 nm, t_R (major) = 14.00 min, t_R (minor) = 28.90 min.

HRMS (ESI-TOF): calcd for $C_{43}H_{38}N_2O_4P^+$ [M+H$^+$] 677.2564, found 677.2568.

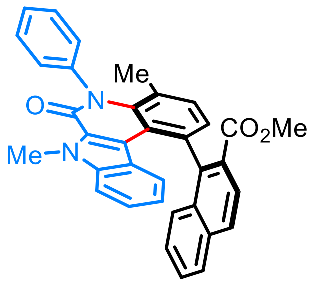

5y

methyl *(R)*-1-(4,7-dimethyl-6-oxo-5-phenyl-6,7-dihydro-5H-indolo[2,3-c]quinolin-1-yl)-2-naphthoate (5y)

Physical state: light yellow solid.

Yield: *(R)*-**5y**, 44.4 mg, 85%.

^{1}H NMR (500 MHz, Chloroform-d) δ 8.01 (d, J = 8.5 Hz, 1H), 7.94 (d, J = 8.5 Hz, 1H), 7.89 (d, J = 8.1 Hz, 1H), 7.72 (d, J = 8.5 Hz, 1H), 7.54 – 7.40 (m, 6H), 7.30 – 7.23 (m, 2H), 7.19 – 7.06 (m, 3H), 6.36 (t, J = 7.8 Hz, 1H), 5.80 (d, J = 8.5 Hz, 1H), 4.29 (s, 3H), 3.51 (s, 3H), 1.85 (s, 3H).

^{13}C NMR (126 MHz, Chloroform-d) δ 168.82, 158.21, 142.98, 142.01, 140.65, 137.46, 134.91, 132.82, 132.60, 130.08, 129.49, 128.56, 128.15, 127.97, 127.70, 127.56, 127.46, 127.31, 127.21, 127.14, 126.91, 125.91, 125.12, 123.90, 122.29, 121.87, 119.75, 119.69, 109.40, 51.98, 31.77, 23.32.

HPLC: *(R)*-**5y** >99% ee, Chiralpak OD-H column, hexane : isopropanol = 80 : 20, 1.0 mL/min, λ = 254 nm, t_R (major) = 22.07 min.

HRMS (ESI-TOF): calcd for $C_{35}H_{27}N_2O_3^+$ [M+H$^+$] 523.2016, found 523.2012.

6. Synthetic applications

6.1. Scale-up synthesis and synthetic transformation

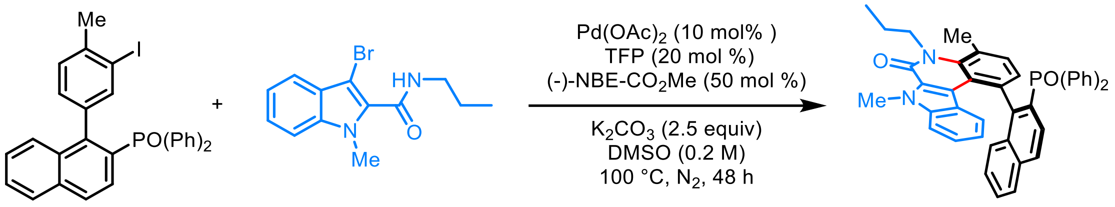

1r (1.0 equiv) **2a** (1.5 equiv) **3r**, 75%, 92% ee

In an argon-filled glove box, a 10 mL oven-dried vial equipped with a magnetic stir bar was charged with $Pd(OAc)_2$ (44.9 mg, 0.20 mmol, 10 mol%), tri(2-furyl)phosphine (92.9 mg, 0.40 mmol, 20 mol%), K_2CO_3 (691.0 mg, 5.00 mmol, 2.5 equiv), (-)-NBE-CO_2Me (152.1 mg, 1.0 mmol, 50 mol%), aryl Iodobenzene **1r** (2.0 mmol, 1.0 equiv), amide **2a** (3.00 mmol, 1.5 equiv) and dry DMSO (7.5 mL). The vial was sealed with a cap and then transferred out of the glove box and stirred at 100 °C for 48 h. After the mixture was cooled to room temperature, it was filtered through a thin pad of celite (eluting with ethyl acetate, 10 mL) and the combined filtrate was concentrated in vacuo. The residue was directly purified by column chromatography on silica to give the product **3r** as a yellow solid (0.95 g, 75%, 92% ee).

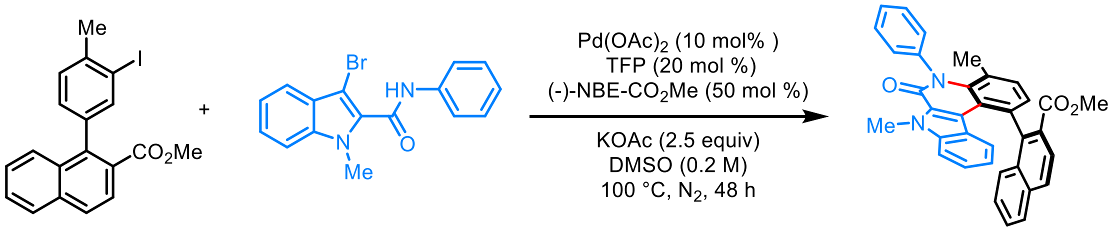

1k (1.0 equiv) **2h** (1.5 equiv) **5y**, 80%, 99% ee

In an argon-filled glove box, a 10 mL oven-dried vial equipped with a magnetic stir bar was charged with $Pd(OAc)_2$ (44.9 mg, 0.20 mmol, 10 mol%), tri(2-furyl)phosphine (92.9 mg, 0.40 mmol, 20 mol%), KOAc (691.0 mg, 5. mmol, 2.5 equiv), (-)-NBE-CO_2Me (152.1 mg, 1.0 mmol, 50 mol%), aryl Iodobenzene **1k** (2.0 mmol, 1.0 equiv), amide **2h** (3.0 mmol, 1.5 equiv) and dry DMSO (7.5 mL). The vial was sealed with a cap and then transferred out of the glove box and stirred at 100 °C for 48 h. After the mixture was cooled to room temperature, it was filtered through a thin pad of celite (eluting with ethyl acetate, 10 mL) and the combined filtrate was concentrated in vacuo. The residue was directly purified by

column chromatography on silica to give the product **5y** as a yellow solid (0.84 g, 80%, 99% ee).

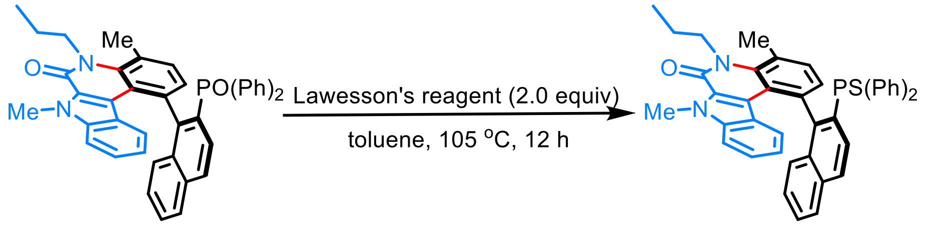

3r, 75%, 92% ee   **6**, 83%, 88% ee

To a dried Schlenk flask charged with the phosphine oxide **3r** (63.0 mg, 0.1mmol) in dry toluene (2.0 mL) was added Lawesson's reagent (80.9 mg, 0.2 mmol, 2.0 equiv) under N_2. The resulting mixture was stirred at 105 °C in an oil bath for 12 h. After cooled to 0 °C, it was diluted by EtOAc (5.0 mL), quenched with small amount of saturated Na_2CO_3 solution. The resulting suspension was filtered by a short celite column, and washed with EtOAc(3 × 5 mL). The filtrate was dried over anhydrous Na_2SO_4, filtered, concentrated, and the residue was purified by flash chromatography on silica gel (PE:EtOAc = 1:1) to afford the desired product **6** as a white solid (53.6 mg, 83% yield, 88% ee).

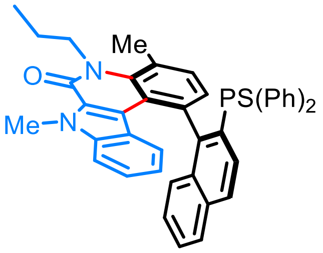

6

***(R)*-1-(2-((diphenyl-l4-sulfaneylidene)phosphaneyl)naphthalen-1-yl)-4,7-dimethyl-5-propyl-5,7-dihydro-6H-indolo[2,3-c]quinolin-6-one (7)**

Physical state: yellow solid.

Yield: *(R)*-**6**, 53.6 mg, 83%.

^{1}H NMR (500 MHz, Chloroform-*d*) δ 8.08 (dd, *J* = 8.8, 1.9 Hz, 1H), 7.91 (d, *J* = 8.2 Hz, 1H), 7.81 – 7.75 (m, 3H), 7.45 (t, *J* = 7.5 Hz, 1H), 7.42 – 7.31 (m, 4H), 7.30 – 7.24 (m, 3H), 7.20 – 7.12 (m, 4H), 6.93 (t, *J* = 7.3 Hz, 1H), 6.62 – 6.56 (m, 3H), 5.41 (d, *J* = 8.6 Hz, 1H), 4.62 – 4.55 (m, 1H), 4.37 (s, 3H), 4.09 – 4.00 (m, 1H), 2.55 (s, 3H), 1.72 – 1.64 (m, 2H), 0.77 (t, *J* = 7.4 Hz, 3H).

^{13}C NMR (126 MHz, Chloroform-*d*) δ 159.27, 146.19 (d, *J* = 8.2 Hz), 140.34, 136.92, 134.52 (d, *J* = 11.1 Hz), 134.18 (d, *J* = 2.3 Hz), 133.77 (d, *J* = 85.0 Hz), 132.45, 132.37, 131.99 (d, *J* = 87.3 Hz), 131.45, 131.36, 130.90, 130.87, 130.81, 130.80, 130.77, 129.16, 129.12 (d, *J* = 17.5 Hz), 129.10, 128.94, 128.29 (d, *J* = 12.9 Hz), 128.05, 128.02, 127.86, 127.79 (d,

J = 8.3 Hz), 127.13, 127.12 (d, *J* = 12.5 Hz), 126.25 (d, *J* = 12.8 Hz), 124.97, 124.70, 123.11, 121.59, 119.79, 118.57, 109.75, 50.42, 31.85, 23.30, 21.73, 11.14.
^{31}P NMR (202 MHz, Chloroform-*d*) δ +43.87.
HPLC: *(R)*-**6** 88% ee, Chiralpak AD-H column, hexane : isopropanol = 80 : 20, 1.0 mL/min, λ = 254 nm, t_R (major) = 13.61 min, t_R (minor) = 8.18 min.
HRMS (ESI-TOF): calcd for $C_{42}H_{36}N_2OPS^+$ [M+H$^+$] 647.2280, found 647.2283.

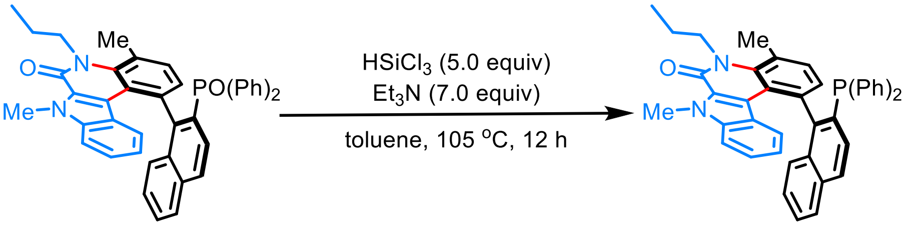

3r, 75%, 92% ee **7**, 81%, 92% ee

To a dried Schlenk flask charged with the phosphine oxide **3r** (63.0 mg, 0.1 mmol) in dry toluene (2 mL) was added Et_3N (70.8 mg, 0.7 mmol) and $HSiCl_3$ (67.7 mg, 0.5 mmol) successively under N_2 at 0°C. The resulting mixture was stirred at 105 °C for 12 h. After cooled to 0 °C, it was diluted by EtOAc (5 mL), quenched with small amount of saturated Na_2CO_3 solution. The resulting suspension was filtered by a short celite column, and washed with EtOAc (3 × 5 mL). The filtrate was dried over anhydrous Na_2SO_4, filtered, concentrated, and the residue was purified by flash chromatography on silica gel (PE:EtOAc = 5:1) to afford the desired phosphine **7** as a white solid (37.5 mg, 81% yield, 92% ee).

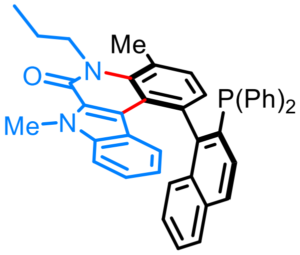

7

***(R)*-1-(2-(diphenylphosphaneyl)naphthalen-1-yl)-4,7-dimethyl-5-propyl-5,7-dihydro-6H-indolo[2,3-c]quinolin-6-one (7)**
Physical state: white solid.
Yield: *(R)*-**7**, 37.5 mg, 81%.
^{1}H NMR (500 MHz, Chloroform-*d*) δ 7.93 – 7.88 (m, 2H), 7.82 (d, *J* = 8.5 Hz, 1H), 7.50 (t, *J* = 7.4 Hz, 1H), 7.36 (t, *J* = 7.6 Hz, 1H), 7.27 – 7.20 (m, 5H), 7.12 (t, *J* = 7.6 Hz, 2H), 7.09 (d, *J* = 7.7 Hz, 1H), 7.04 (t, *J* = 6.9 Hz, 2H), 6.96 (t, *J* = 7.2 Hz, 2H), 6.89 (d, *J* = 7.6 Hz, 1H), 6.59 (t, *J* = 7.4 Hz, 2H), 6.28 (t, *J* = 7.7 Hz, 1H), 5.49 (d, *J* = 8.6 Hz, 1H), 4.60 – 4.53 (m, 1H), 4.51 – 4.45 (m, 1H), 4.27 (s, 3H), 2.69 (s, 3H), 1.75 – 1.70 (m, 2H), 0.84 (t, *J* = 7.4 Hz, 3H).

^{13}C NMR (126 MHz, Chloroform-*d*) δ 159.61, 148.94 (d, J = 33.3 Hz), 140.42, 137.96 (d, J = 13.5 Hz), 137.52, 137.04 (d, J = 13.7 Hz), 135.35 (d, J = 12.1 Hz), 133.98, 133.79 (d, J = 6.8 Hz), 133.41, 133.25, 132.91, 132.76 (d, J = 8.4 Hz), 132.75, 130.86 (d, J = 1.9 Hz), 128.69, 128.66, 128.08, 128.02, 128.00, 127.93, 127.83, 127.74 (d, J = 6.4 Hz), 127.51, 127.41 (d, J = 2.7 Hz), 126.88, 126.59, 126.09, 124.64, 124.54, 123.48 (d, J = 3.7 Hz), 121.70, 119.51, 119.01 (d, J = 2.2 Hz), 109.28, 49.71, 31.71, 23.48, 21.92, 11.29.
^{31}P NMR (202 MHz, Chloroform-*d*) δ +13.45.
HPLC: *(R)*-**7** 92% ee, Chiralpak IC-3 column, hexane : isopropanol = 95 : 5, 0.2 mL/min, λ = 254 nm, t_R (major) = 47.58 min, t_R (minor) = 45.97 min.
HRMS (ESI-TOF): calcd for $C_{42}H_{36}N_2OP^+$ [M+H$^+$] 615.2560, found 615.2561.

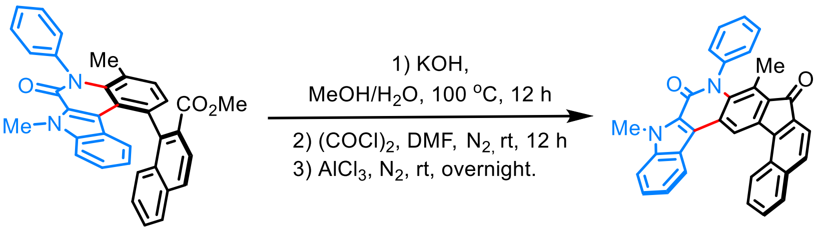

5y, 80%, 99% ee　　**8**, 85%, 82% ee

To a solution of compound **5y** (52.2 mg, 0.1 mmol) in MeOH (1 mL) and H_2O (0.2 mL) was added KOH (14.0 mg, 0.25 mmol) at room temperature. The mixture was stirred at 100 °C for 12 hours. Then MeOH was removed on rotary evaporator, and the residue was diluted with EtOAc and acidified with HCl (4 N). The aqueous phase was extracted with EtOAc. The organic layer was dried over anhydrous Na_2SO_4, concentrated to afford the product, which was pure enough for the next step. Anhydrous dichloromethane (3 mL) was added, and the suspension was stirred at room temperature under N_2. Oxalyl chloride (40.0 µL, 0.4 mmol, 4.0 eq) was added, followed by anhydrous dimethylformamide (2 d) dropwise. The suspension was stirred at room temperature for 12 h under N_2, after which time the solvent and excess oxalyl chloride was removed under reduced pressure. The resultant yellowish solid intermediate was immediately purged with N_2 and dissolved in anhydrous dichloromethane (2 mL). Aluminium trichloride (53.3 mg, 0.4 mmol, 4 equiv) was added under a positive N_2 pressure and it was stirred at room temperature overnight under N_2. The reaction solution was added with water and then extracted with EtOAc. The organic layer was dried over anhydrous Na_2SO_4, concentrated and purified by flash chromatography on silica gel (PE:EtOAc = 5:1) to afford compound **8** (41.8 mg, 85% yield, 82% ee) as a white solid.

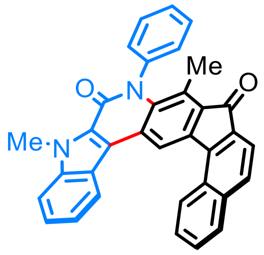

8

***(R)*-5,8-dimethyl-7-phenyl-5,7-dihydrobenzo[6,7]indeno[1,2-f]indolo[2,3-c]quinoline-6,10-dione (8)**

Physical state: white solid.

Yield: *(R)*-**9**, 41.8 mg, 85%.

^{1}H NMR (500 MHz, Chloroform-*d*) δ 7.84 (s, 2H), 7.77 (dd, *J* = 12.3, 8.6 Hz, 2H), 7.60 (dd, *J* = 15.0, 7.4 Hz, 2H), 7.44 (q, *J* = 8.5, 7.9 Hz, 3H), 7.36 (s, 1H), 7.25 – 7.20 (m, 2H), 7.12 – 7.09 (m, 1H), 6.83 (d, *J* = 8.3 Hz, 1H), 6.72 (t, *J* = 7.7 Hz, 1H), 6.56 (t, *J* = 7.6 Hz, 1H), 4.38 (s, 3H), 1.70 (s, 3H).

^{13}C NMR (126 MHz, Chloroform-*d*) δ 193.38, 158.19, 147.92, 143.27, 140.42, 140.05, 137.57, 132.49, 131.37, 129.87, 129.39, 128.52, 128.43, 128.23, 128.01, 127.98, 127.17, 126.74, 125.71, 125.67, 125.54, 125.29, 125.15, 122.97, 120.25, 119.79, 119.41, 119.06, 110.05, 31.47, 22.50.

HPLC: *(R)*-**8** 82% ee, Chiralpak IA-3 column, hexane : isopropanol = 90 : 10, 0.5 mL/min, λ = 254 nm, t_R (major) = 21.20 min, t_R (minor) = 22.39 min.

HRMS (ESI-TOF): calcd for $C_{34}H_{23}N_2O_2^+$ [M+H$^+$] 491.1754, found 491.1751.

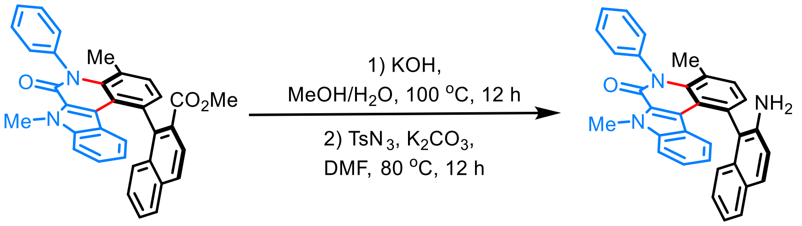

5y, 80%, 99% ee **9**, 56%, 80% ee

To a solution of compound **5y** (52.2 mg, 0.1 mmol) in MeOH (1 mL) and H_2O (0.2 mL) was added KOH (14.0 mg, 0.25 mmol) at room temperature. The mixture was stirred at 100 °C for 12 hours. Then MeOH was removed on rotary evaporator, and the residue was diluted with EtOAc and acidified with HCl (4 N). The aqueous phase was extracted with EtOAc. The organic layer was dried over anhydrous Na_2SO_4, concentrated to afford the product, which was pure enough for the next step. Then to a solution of just acquired compound (50.8 mg, 0.1 mmol) in DMF was added K_2CO_3 (27.6 mg, 0.20 mmol) and TsN_3 (23.7 mg, 0.12 mmol) at room temperature. The mixture was stirred at 80 °C for 12 hours. The reaction solution was cooled, added with water and then extracted with EtOAc. The

organic layer was dried over anhydrous Na_2SO_4, concentrated and purified by flash chromatography on silica gel (PE:EtOAc = 5:1) to afford compound **9** (26.8 mg, 56% yield, 80% ee) as a yellow solid.

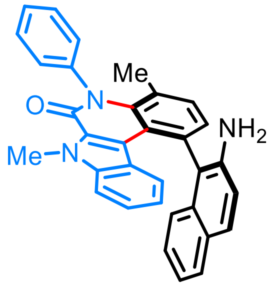

9

***(R)*-1-(2-aminonaphthalen-1-yl)-4,7-dimethyl-5-phenyl-5,7-dihydro-6H-indolo[2,3-c]quinolin-6-one (9)**

Physical state: yellow solid.

Yield: *(R)*-**10**, 26.8 mg, 56%.

^{1}H NMR (500 MHz, Chloroform-*d*) δ 7.79 (dd, *J* = 7.9, 5.5 Hz, 2H), 7.61 (d, *J* = 8.3 Hz, 1H), 7.50 (d, *J* = 8.6 Hz, 3H), 7.44 – 7.39 (m, 2H), 7.32 – 7.28 (m, 2H), 7.25 (s, 1H), 7.22 (t, *J* = 8.7 Hz, 2H), 7.17 (t, *J* = 7.3 Hz, 1H), 6.93 (d, *J* = 8.8 Hz, 1H), 6.42 (t, *J* = 8.0 Hz, 1H), 6.16 (d, *J* = 8.6 Hz, 1H), 4.31 (s, 3H), 3.66 (s, 2H), 1.83 (s, 3H).

^{13}C NMR (126 MHz, Chloroform-*d*) δ 142.01, 141.69, 134.40, 131.18, 130.52, 129.29, 128.59, 128.07, 127.41, 127.09, 126.72, 125.39, 124.77, 124.74, 122.41, 122.25, 121.88, 121.45, 119.93, 119.87, 118.33, 109.34, 31.84, 23.33.

HPLC: *(R)*-**9** 80% ee, Chiralpak AD-H column, hexane : isopropanol = 90 : 10, 1.0 mL/min, λ = 254 nm, t_R (major) = 29.95 min, t_R (minor) = 18.63 min.

HRMS (ESI-TOF): calcd for $C_{33}H_{26}N_3O^+$ [M+H$^+$] 480.2070, found 480.2068.

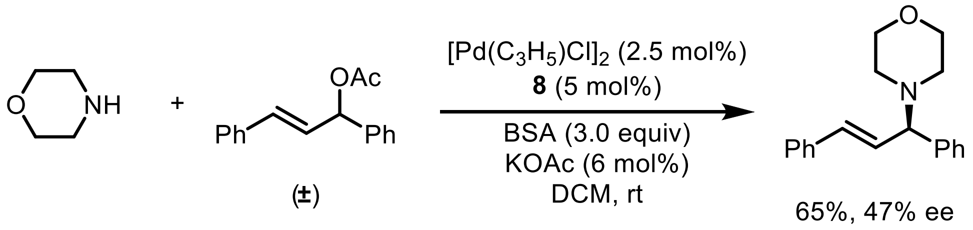

10 **11** **12**

To a dried Schlenk flask charged with $[Pd(C_3H_5)Cl]_2$ (0.9 mg, 0.0025 mmol, 0.025 equiv), chiral ligand **8** (2.7 mg, 0.005 mmol, 0.05 equiv) and KOAc (0.6 mg, 0.006 mmol, 0.06 equiv) was added dry DCM (1 mL) under argon. After stirring at r.t. for 30 min, 1,3-diphenyl-2-propenyl acetate (25.2 mg, 0.1 mmol, 1.0 equiv), morpholine (26.1 mg, 0.3 mmol, 3.0 equiv) and N,O bis(trimethylsilyl)-acetamide (BSA) (61.0 mg, 0.3 mmol, 3.0 equiv) were added. The mixture was stirred at room temperature for 24 h, then diluted with

EtOAc (5 mL) and quenched by saturated NH_4Cl aqueous solution (5 mL). The aqueous layer was extracted with EtOAc (3 × 5 mL) and the combined organics were washed with brine (10 mL), dried over Na_2SO_4, filtered, and concentrated. The residue was purified by flash column chromatography on silica gel (PE:EtOAc = 20:1) to afford the product **12** as a colorless oil (18.1 mg, 65% yield, 47% ee).

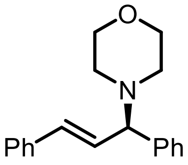

12

(*R*,E)-4-(1,3-diphenylallyl)morpholine (12)
Physical state: colorless oil.
Yield: *(R)*-**12**, 26.8 mg, 65%.
^{1}H NMR (500 MHz, Chloroform-*d*) δ 7.45 (d, *J* = 7.6 Hz, 2H), 7.41 – 7.35 (m, 4H), 7.34 – 7.27 (m, 3H), 7.24 (t, *J* = 7.3 Hz, 1H), 6.61 (d, *J* = 15.8 Hz, 1H), 6.33 (dd, *J* = 15.8, 8.9 Hz, 1H), 3.83 (d, *J* = 8.9 Hz, 1H), 3.75 (t, *J* = 4.6 Hz, 4H), 2.67 – 2.52 (m, 2H), 2.47 – 2.40 (m, 2H). The ^{1}H NMR matched the literature reported data.
^{13}C NMR (126 MHz, Chloroform-*d*) δ 141.50, 136.70, 131.53, 131.33, 128.62, 128.47, 127.99, 127.52, 127.26, 126.34, 74.75, 67.11, 52.14.
HPLC: *(R)*-**12** 47% ee, Chiralpak OD-H column, hexane : isopropanol = 90 : 10, 1.0 mL/min, λ = 254 nm, t_R (major) = 10.06 min, t_R (minor) = 5.64 min.

7. X-ray crystallographic data

A single crystal of compound **5y** suitable for X-ray crystallography was obtained by crystallization via slow evaporation from its ethyl acetate and hexane solution.

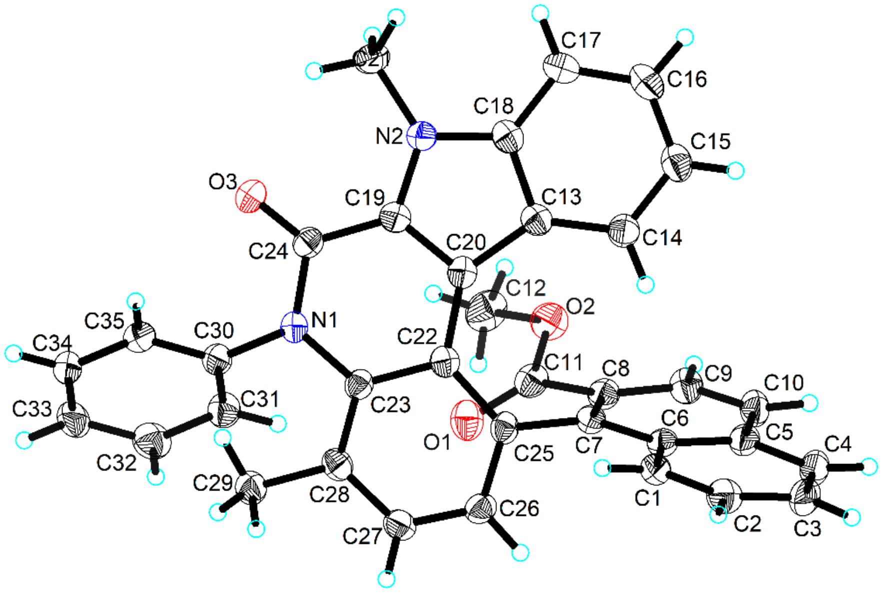

Thermal ellipsoids are shown at the 65% level.

Table S15. Crystal data and structure refinement for compound **5y**.

Identification code	exp_1222_auto
CCDC Deposition Number	2429005
Empirical formula	$C_{35}H_{26}N_2O_3$
Formula weight	522.58
Temperature/K	100(10)
Crystal system	orthorhombic
Space group	$P2_12_12_1$
a/Å	16.31170(10)
b/Å	17.10750(10)
c/Å	18.38360(10)
α/°	90
β/°	90
γ/°	90

Volume/Å^3	5129.99(5)
Z	8
ρ_{calc}g/cm^3	1.353
μ/mm^{-1}	0.690
F(000)	2192.0
Crystal size/mm^3	0.1 × 0.09 × 0.08
Radiation	Cu Kα (λ = 1.54184)
2Θ range for data collection/°	7.058 to 150.04
Index ranges	-19 ≤ h ≤ 20, -21 ≤ k ≤ 20, -16 ≤ l ≤ 22
Reflections collected	59047
Independent reflections	10346 [R_{int} = 0.0378, R_{sigma} = 0.0231]
Data/restraints/parameters	10346/0/727
Goodness-of-fit on F^2	1.039
Final R indexes [I>=2σ (I)]	R_1 = 0.0303, wR_2 = 0.0765
Final R indexes [all data]	R_1 = 0.0319, wR_2 = 0.0774
Largest diff. peak/hole / e Å^{-3}	0.23/-0.18
Flack parameter	0.03(6)

8. Optical Properties

8.1. UV-vis Absorption and Fluorescence Emission Spectra

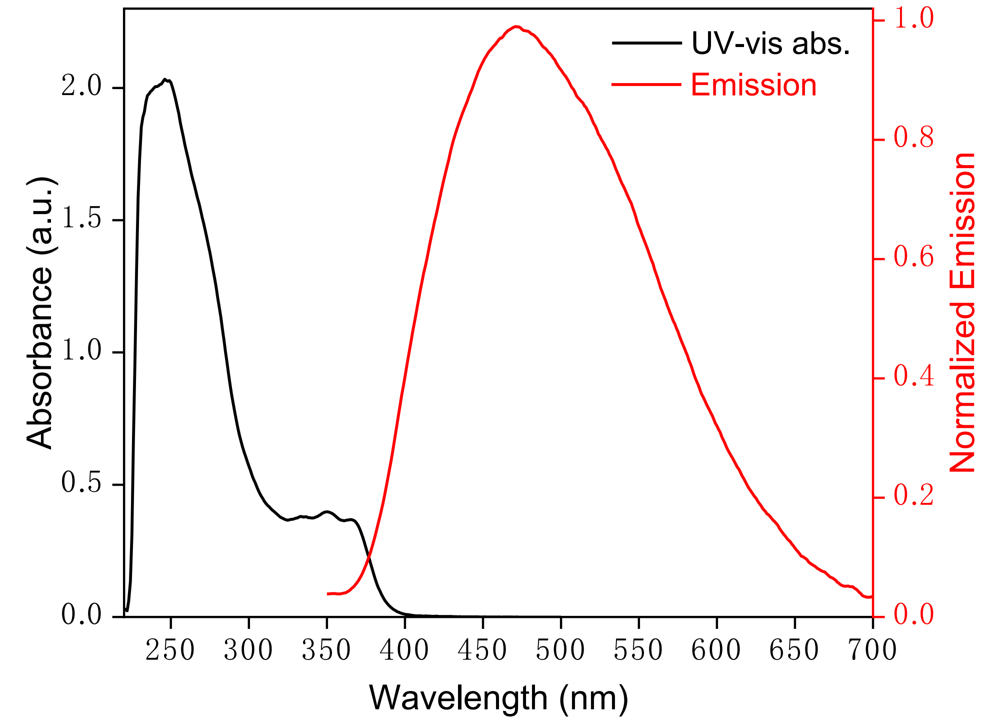

Figure S8. UV-vis absorption and fluorescence emission spectra of compound **3l** (1×10^{-5} M) in dichloromethane using a 10 mm × 10 mm quartz cell. Excited at 313 nm.

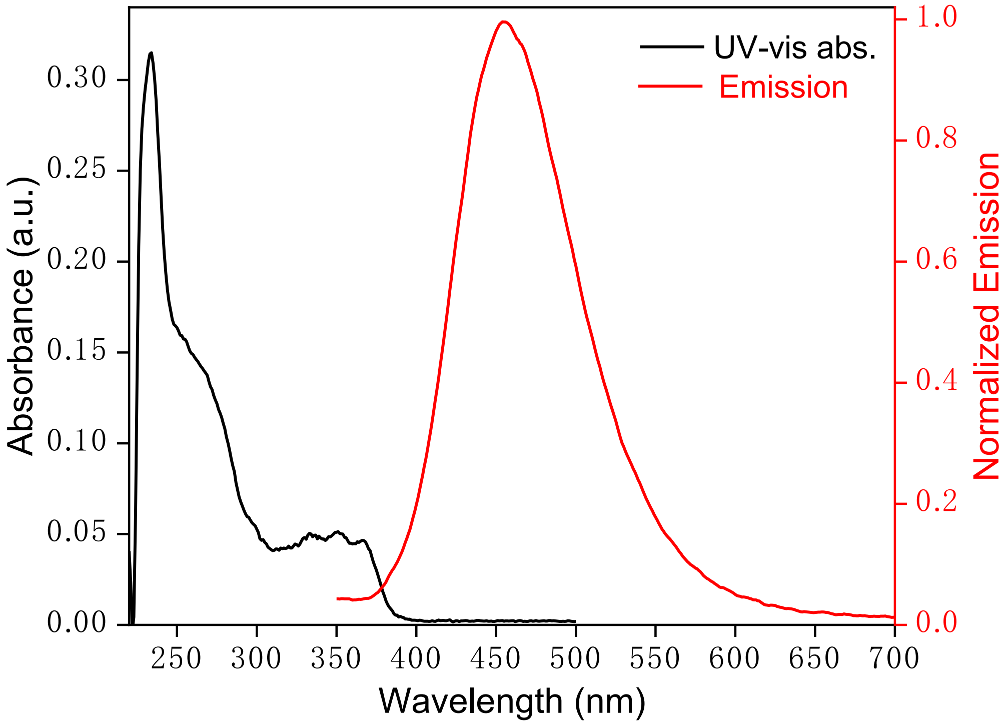

Figure S9. UV-vis absorption and fluorescence emission spectra of compound **3p** (1 × 10^{-5} M) in dichloromethane using a 10 mm × 10 mm quartz cell. Excited at 313 nm.

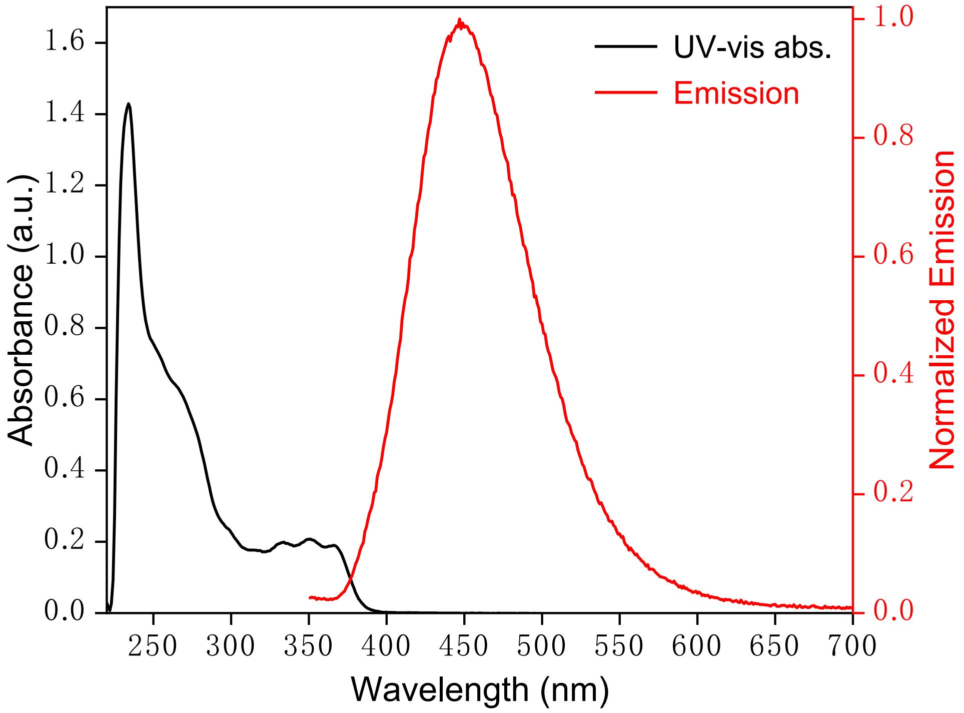

Figure S10. UV-vis absorption and fluorescence emission spectra of compound **3q** (1 × 10^{-5} M) in dichloromethane using a 10 mm × 10 mm quartz cell. Excited at 313 nm.

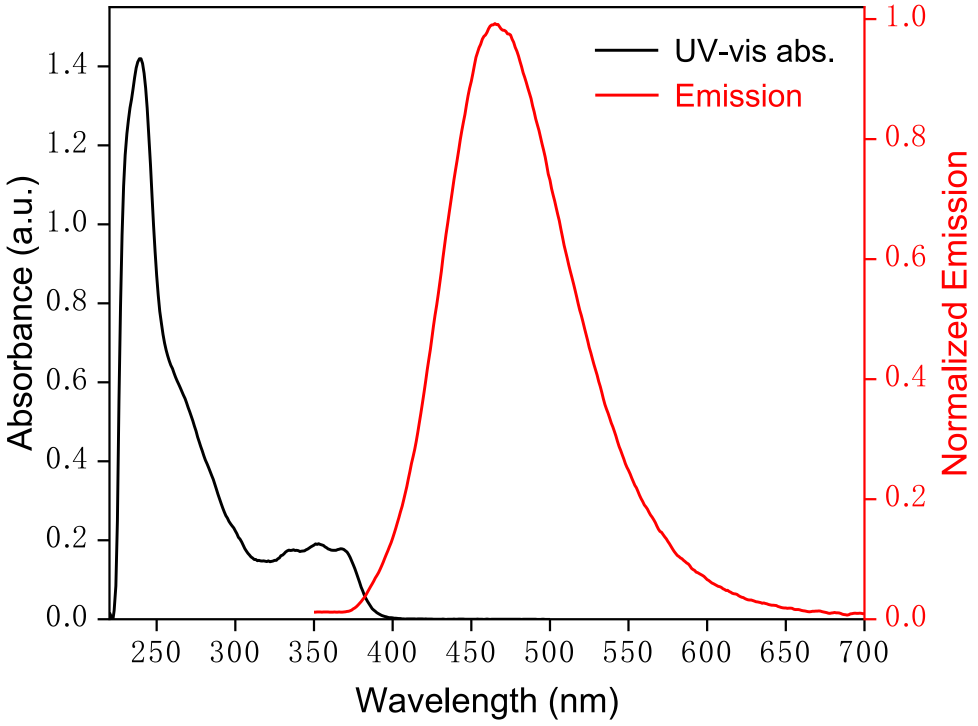

Figure S11. UV-vis absorption and fluorescence emission spectra of compound **3r** (1 × 10^{-5} M) in dichloromethane using a 10 mm × 10 mm quartz cell. Excited at 313 nm.

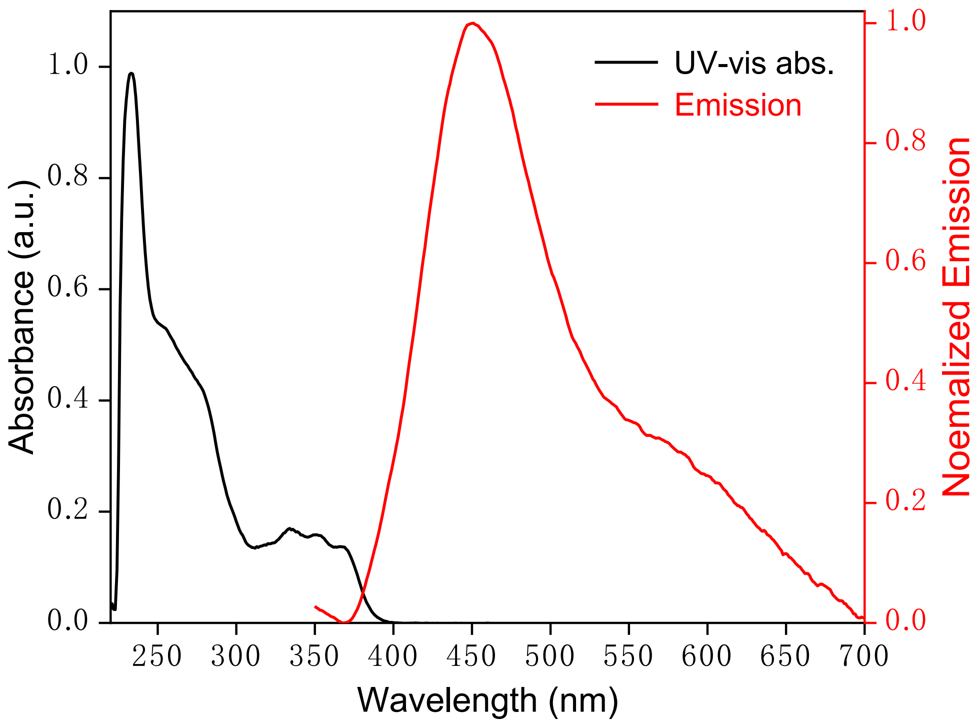

Figure S12. UV-vis absorption and fluorescence emission spectra of compound **5l** (1 × 10^{-5} M) in dichloromethane using a 10 mm × 10 mm quartz cell. Excited at 313 nm.

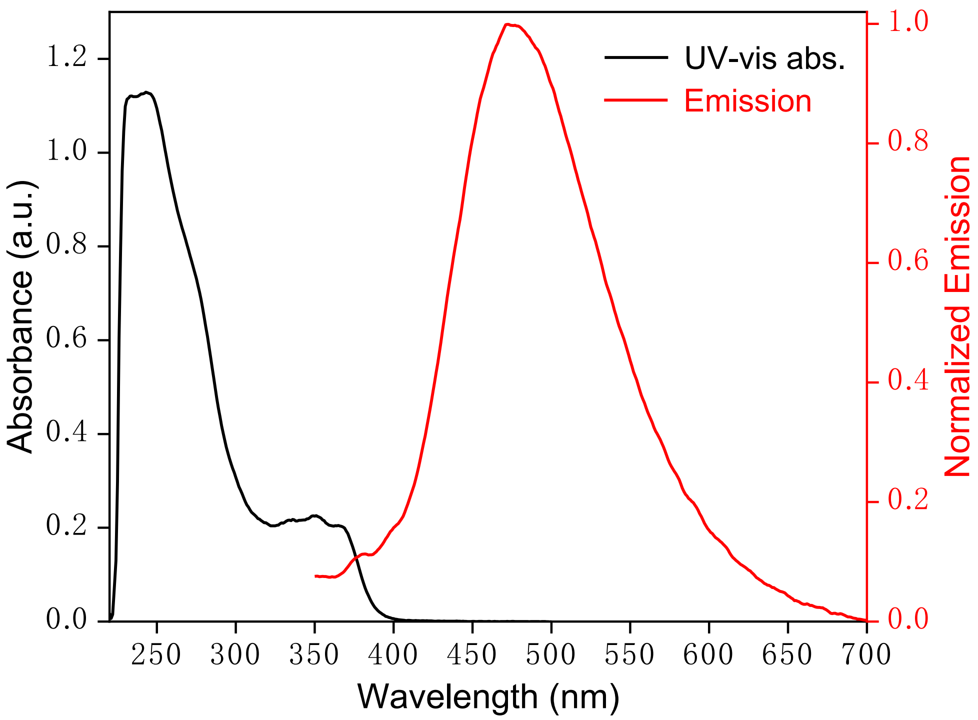

Figure S13. UV-vis absorption and fluorescence emission spectra of compound **5y** (1×10^{-5} M) in dichloromethane using a 10 mm × 10 mm quartz cell. Excited at 313 nm.

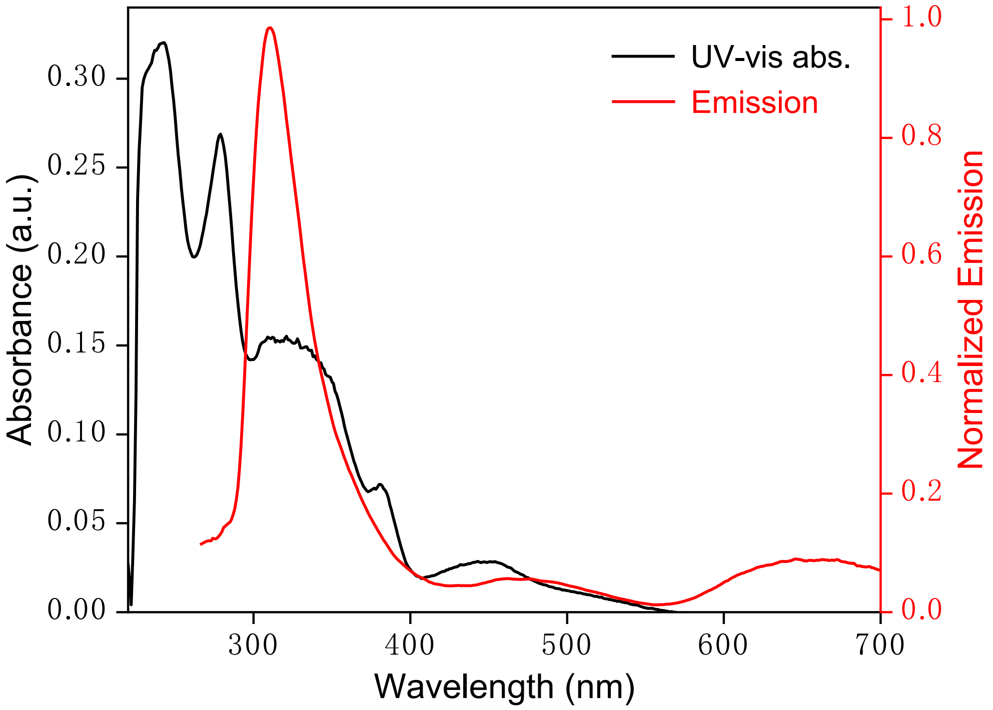

Figure S14. UV-vis absorption and fluorescence emission spectra of compound **9** (1×10^{-5} M) in dichloromethane using a 10 mm × 10 mm quartz cell. Excited at 313 nm.

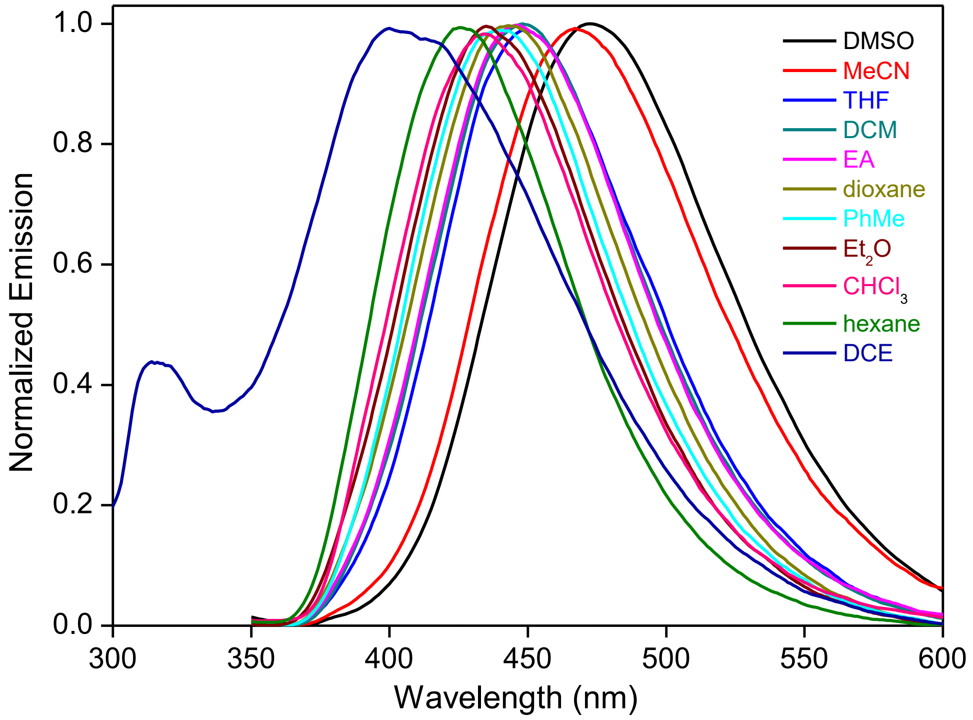

Figure S15. Fluorescence emission spectra of compound **3q** (1×10^{-5} M) in different solvents using a 10 mm × 10 mm quartz cell. Normalized Emission. Excited at 313 nm.

8.2. Photoluminescence Quantum Yield (PLQY)

The PLQY was measured in an integrating sphere with an integrating period for 10000 ms. The sample solution (1×10^{-5} M, 1 mL) was contained in a 10×10 mm quartz cell with a long neck.

Table S16. PLQY of representative compounds in dichloromethane.

Compound	Excitation (nm)	Integral interval of excitation (nm)	Integral interval of emission (nm)	PLQY
3l	313	300 - 330	400 - 800	1.56%
3p	313	290 - 330	300 - 800	13.15%
3q	313	300 - 330	300 - 800	11.09%
3r	313	300 - 330	300 - 800	12.11%
5l	313	290 - 330	400 - 800	0.66%
5y	313	290 - 330	400 - 800	2.66%
9	293	280 - 320	500 - 800	0.44%

Table S17. PLQY of **3q** in different solvents. Excited at 313 nm.

Compound	Solvent	Integral interval of excitation (nm)	Integral interval of emission (nm)	PLQY
3q	DMSO	300 - 330	300 - 800	14.72%
3q	MeCN	300 - 330	300 - 800	7.71%
3q	$CHCl_3$	290 - 330	300 - 800	9.60%
3q	DCM	300 - 330	300 - 800	11.09%
3q	DCE	300 - 330	300 - 800	7.98%
3q	THF	290 - 330	300 - 800	16.95%
3q	EA	300 - 330	300 - 800	11.02%
3q	dioxane	290 - 330	300 - 800	13.75%
3q	PhMe	290 - 330	300 - 800	16.97%
3q	Et_2O	300 - 330	300 - 800	12.67%

3q	hexane	290 - 330	300 - 800	10.47%

9. References

1. Y. Hua, Z.-S. Liu, P.-P. Xie, B. Ding, H.-G. Cheng, X. Hong and Q. Zhou, Angew. Chem. Int. Ed., 2021, 60, 12824-12828.
2. J. Feng, B. Li, Y. He and Z. Gu, Angew. Chem. Int. Ed., 2016, 55, 2186-2190.
3. Z. Liu, B. Gao, K. Chernichenko, H. Yang, S. Lemaire and W. Tang, Org. Lett., 2023, 25, 7004-7008.
4. R. Ferraccioli, D. Carenzi, E. Motti and M. Catellani, J. Am. Chem. Soc., 2006, 128, 722-723.
5. Y. Zhao and D. G. Truhlar, Theoretical Chemistry Accounts, 2008, 120, 215-241.
6. A. V. Marenich, C. J. Cramer and D. G. Truhlar, The Journal of Physical Chemistry B, 2009, 113, 6378-6396.
7. P. J. Hay and W. R. Wadt, The Journal of Chemical Physics, 1985, 82, 299-310.
8. P. A. Dub, N. J. Henson, R. L. Martin and J. C. Gordon, J. Am. Chem. Soc., 2014, 136, 3505-3521.
9. P. Winget, C. J. Cramer and D. G. Truhlar, Theoretical Chemistry Accounts, 2004, 112, 217-227.
10. T. Lu and F. Chen, Journal of Computational Chemistry, 2012, 33, 580-592.
11. W. Humphrey, A. Dalke and K. Schulten, Journal of Molecular Graphics, 1996, 14, 33-38.
12. CYLview, 1.0b; C. Y. Legault, , Université de Sherbrooke, 2009 (http://www.cylview.org)

10. NMR Spectra

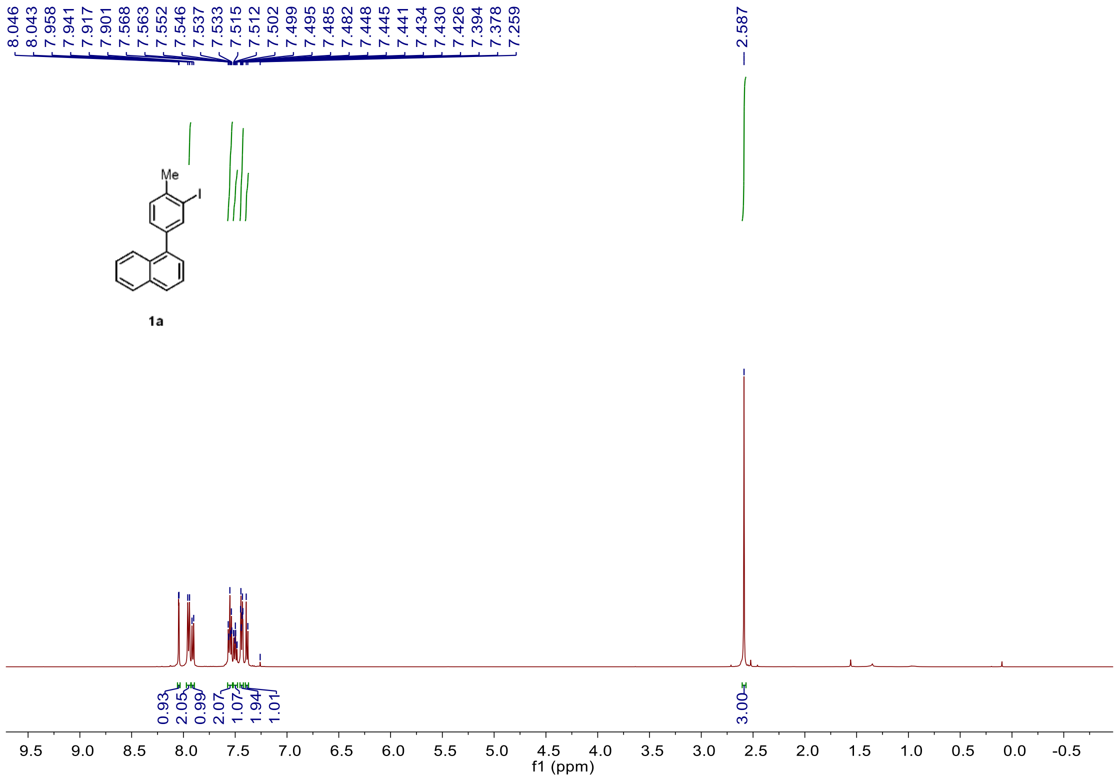

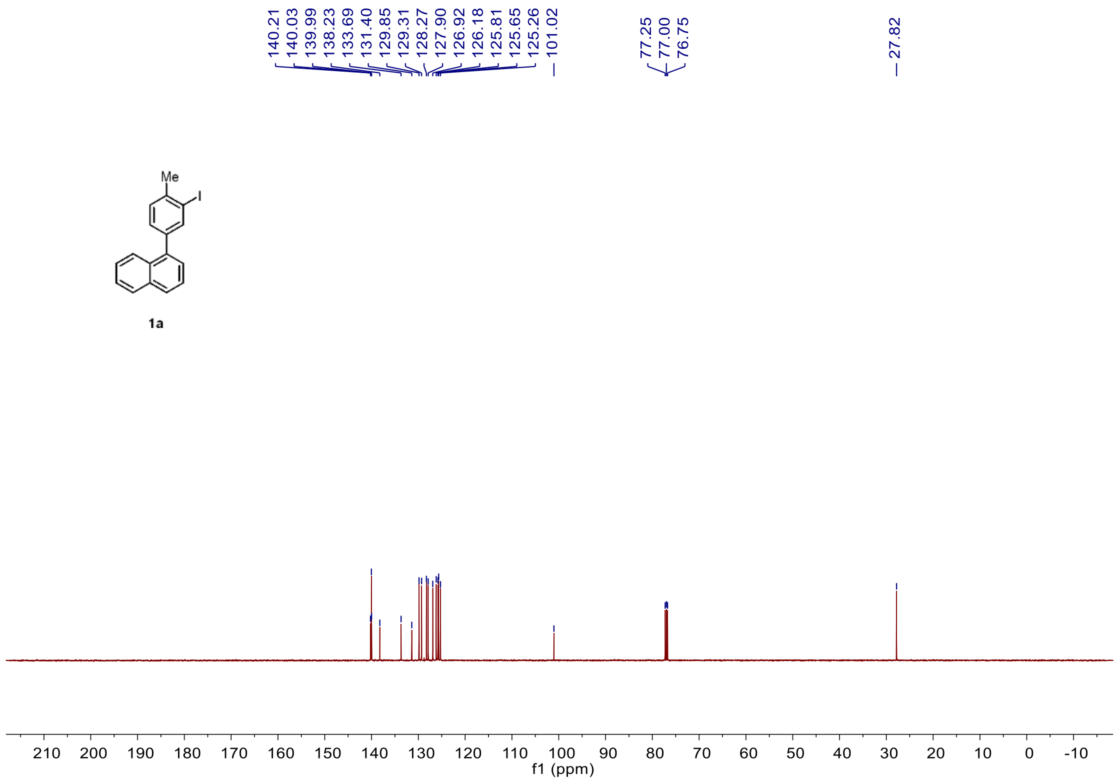

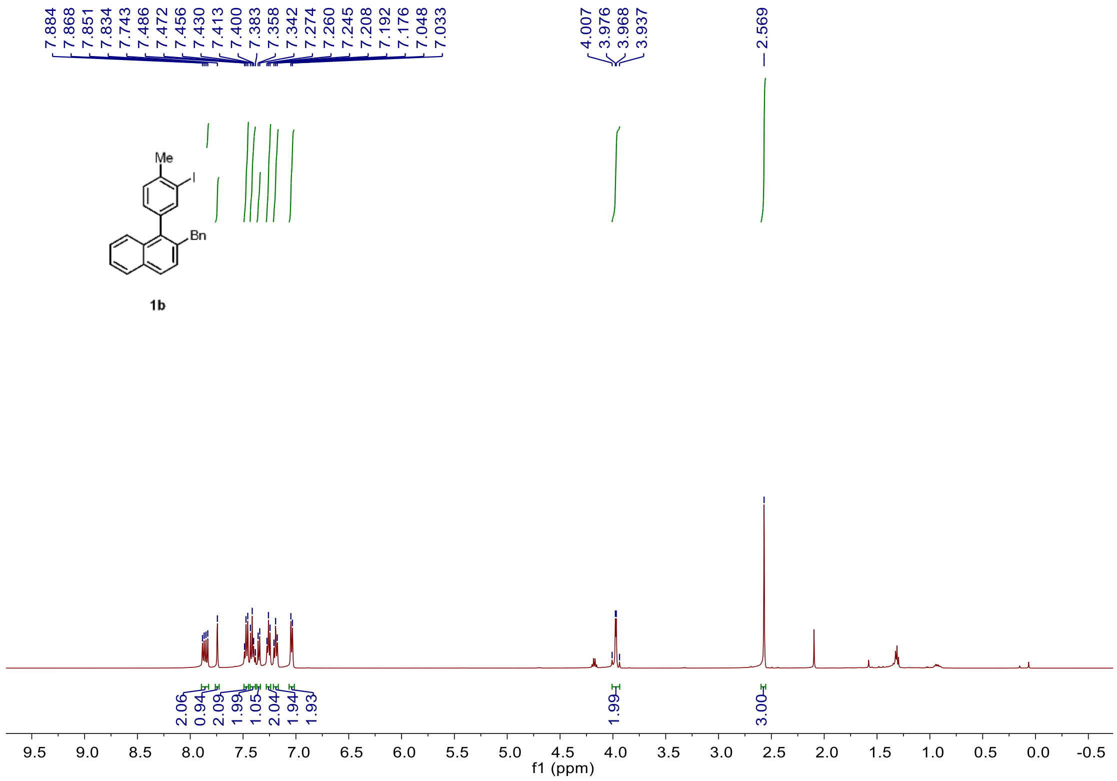
7.884
7.868
7.851
7.834
7.743
7.486
7.472
7.456
7.430
7.413
7.400
7.383
7.358
7.342
7.274
7.260
7.245
7.208
7.192
7.176
7.048
7.033
4.007
3.976
3.968
3.937
2.569
Me
I
Bn
1b
2.06
0.94
2.09
1.99
1.05
2.04
1.94
1.93
1.99
3.00
9.5 9.0 8.5 8.0 7.5 7.0 6.5 6.0 5.5 5.0 4.5 4.0 3.5 3.0 2.5 2.0 1.5 1.0 0.5 0.0 -0.5
f1 (ppm)

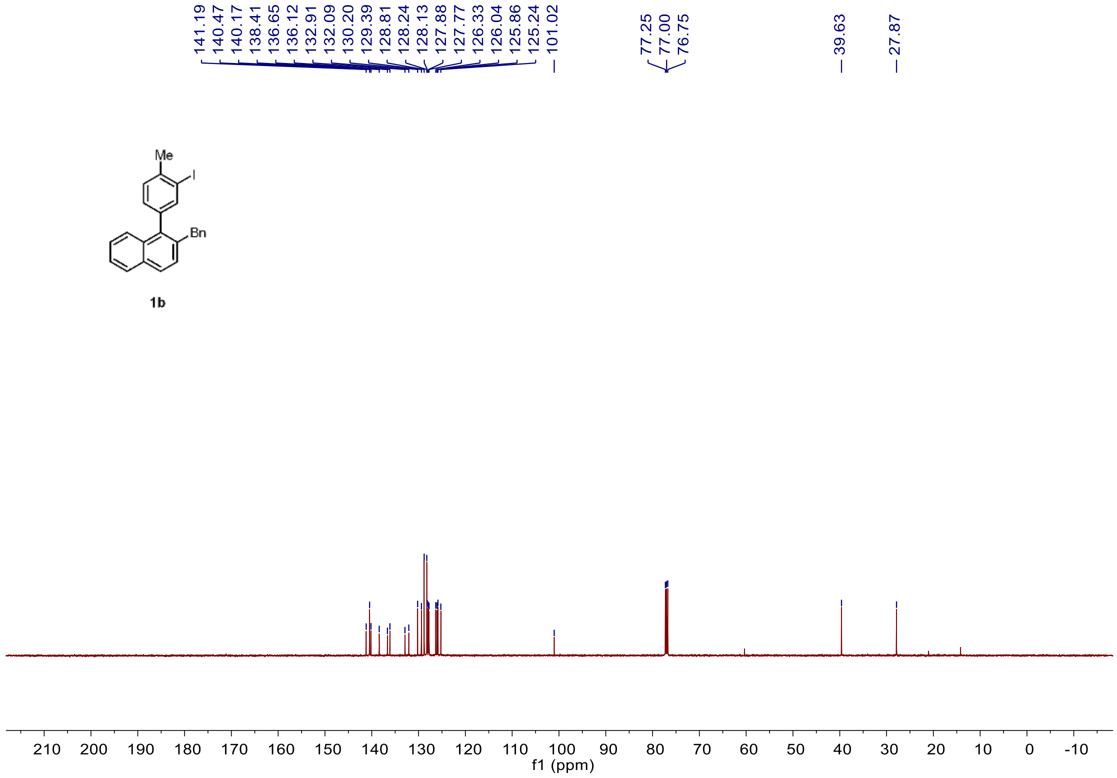
141.19
140.47
140.17
138.41
136.65
136.12
132.91
132.09
130.20
129.39
128.81
128.24
128.13
127.88
127.77
126.33
126.04
125.86
125.24
101.02
77.25
77.00
76.75
39.63
27.87
Me
I
Bn
1b
210 200 190 180 170 160 150 140 130 120 110 100 90 80 70 60 50 40 30 20 10 0 -10
f1 (ppm)

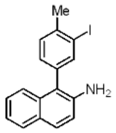

1c

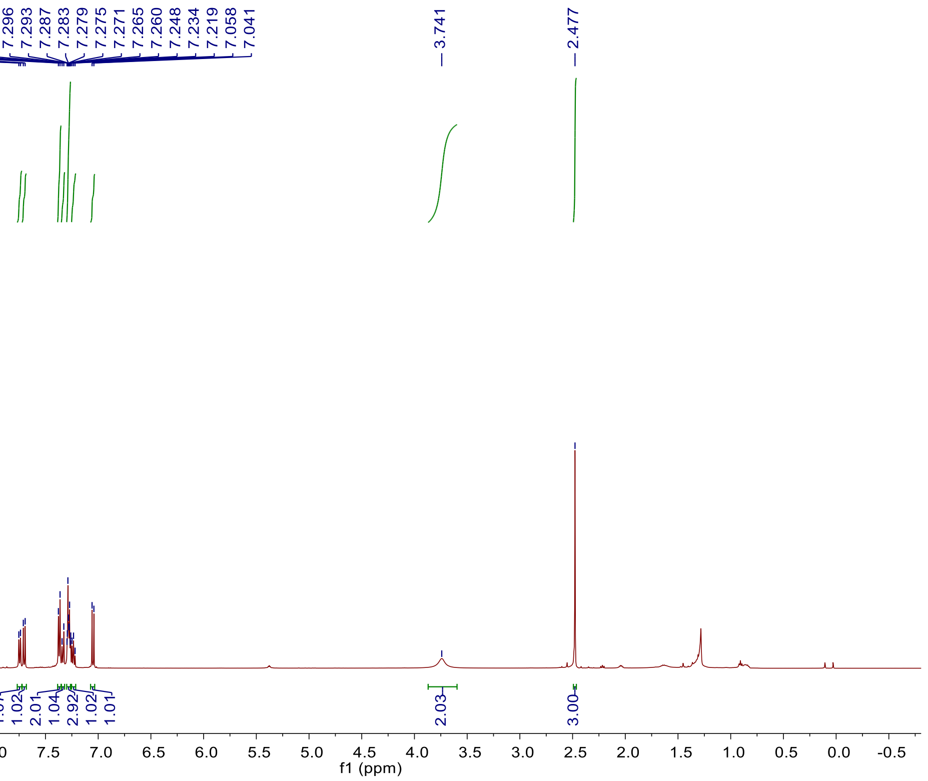

7.753
7.737
7.710
7.693
7.377
7.362
7.343
7.326
7.296
7.293
7.287
7.283
7.279
7.275
7.271
7.265
7.260
7.248
7.234
7.219
7.058
7.041
3.741
2.477
1.07
1.02
2.01
1.04
2.92
1.02
1.01
2.03
3.00
9.5 9.0 8.5 8.0 7.5 7.0 6.5 6.0 5.5 5.0 4.5 4.0 3.5 3.0 2.5 2.0 1.5 1.0 0.5 0.0 -0.5
f1 (ppm)

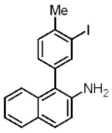

1c

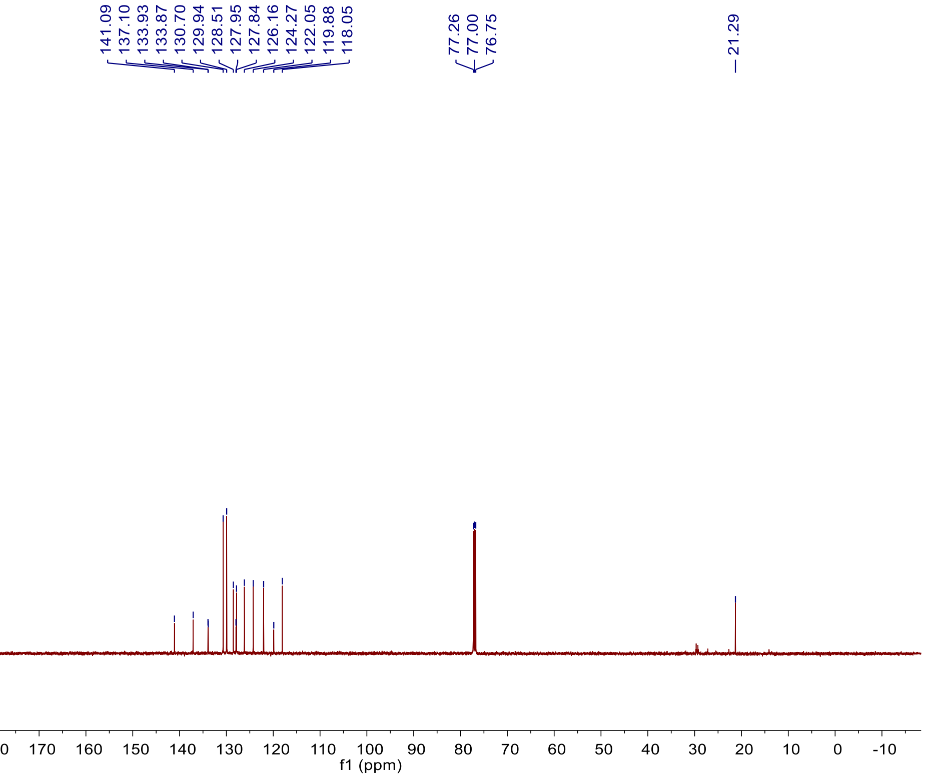

141.09
137.10
133.93
133.87
130.70
129.94
128.51
127.95
127.84
126.16
124.27
122.05
119.88
118.05
77.26
77.00
76.75
21.29
210 200 190 180 170 160 150 140 130 120 110 100 90 80 70 60 50 40 30 20 10 0 -10
f1 (ppm)

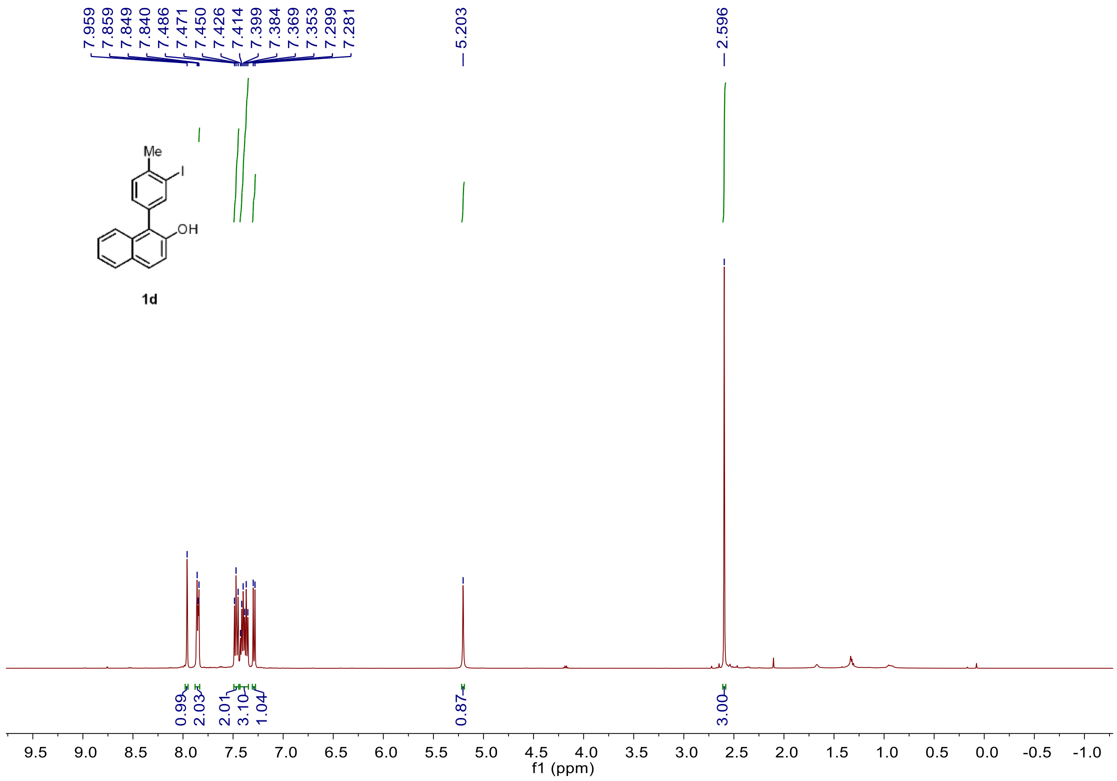
7.959
7.859
7.849
7.840
7.486
7.471
7.450
7.426
7.414
7.399
7.384
7.369
7.353
7.299
7.281
5.203
2.596
Me
I
OH
1d
0.99
2.03
2.01
3.10
1.04
0.87
3.00
9.5 9.0 8.5 8.0 7.5 7.0 6.5 6.0 5.5 5.0 4.5 4.0 3.5 3.0 2.5 2.0 1.5 1.0 0.5 0.0 -0.5 -1.0
f1 (ppm)

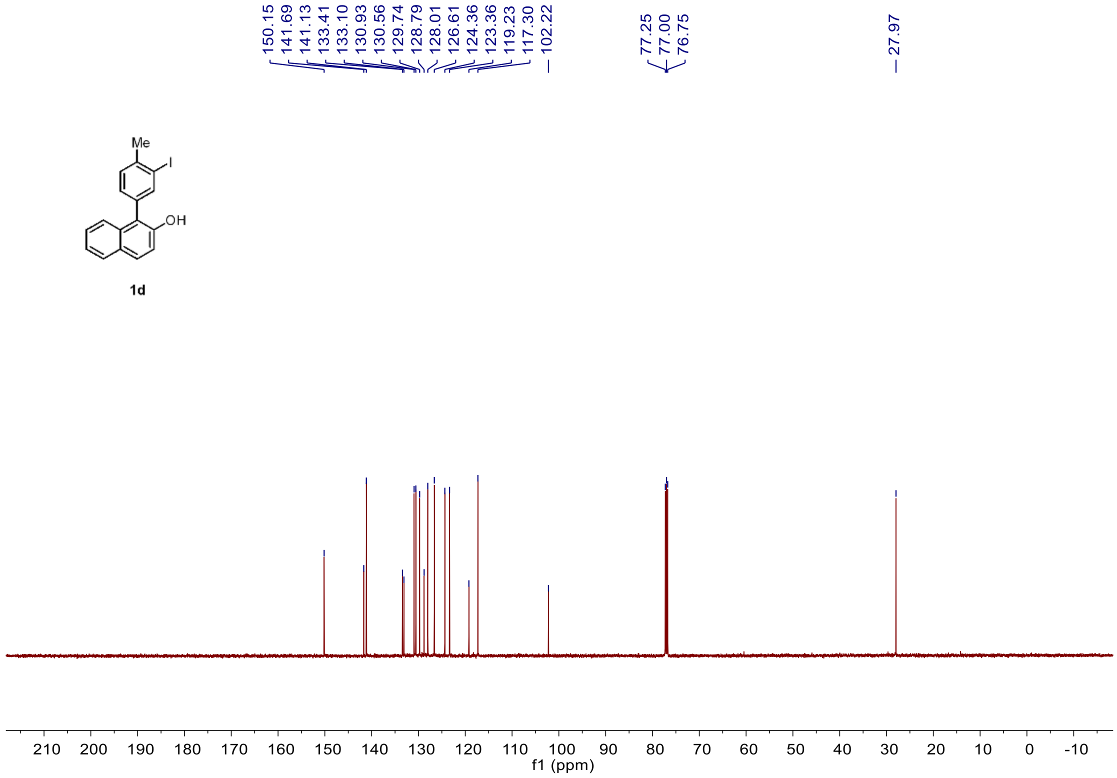
150.15
141.69
141.13
133.41
133.10
130.93
130.56
129.74
128.79
128.01
126.61
124.36
123.36
119.23
117.30
102.22
77.25
77.00
76.75
27.97
Me
I
OH
1d
210 200 190 180 170 160 150 140 130 120 110 100 90 80 70 60 50 40 30 20 10 0 -10
f1 (ppm)

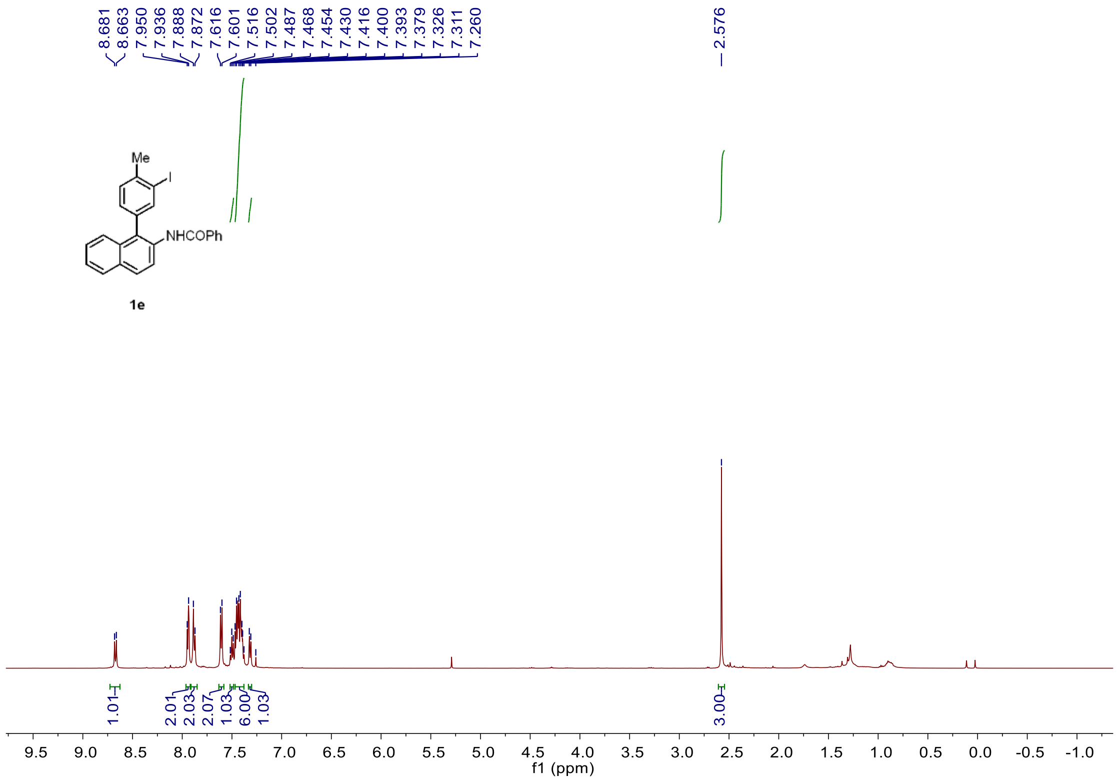

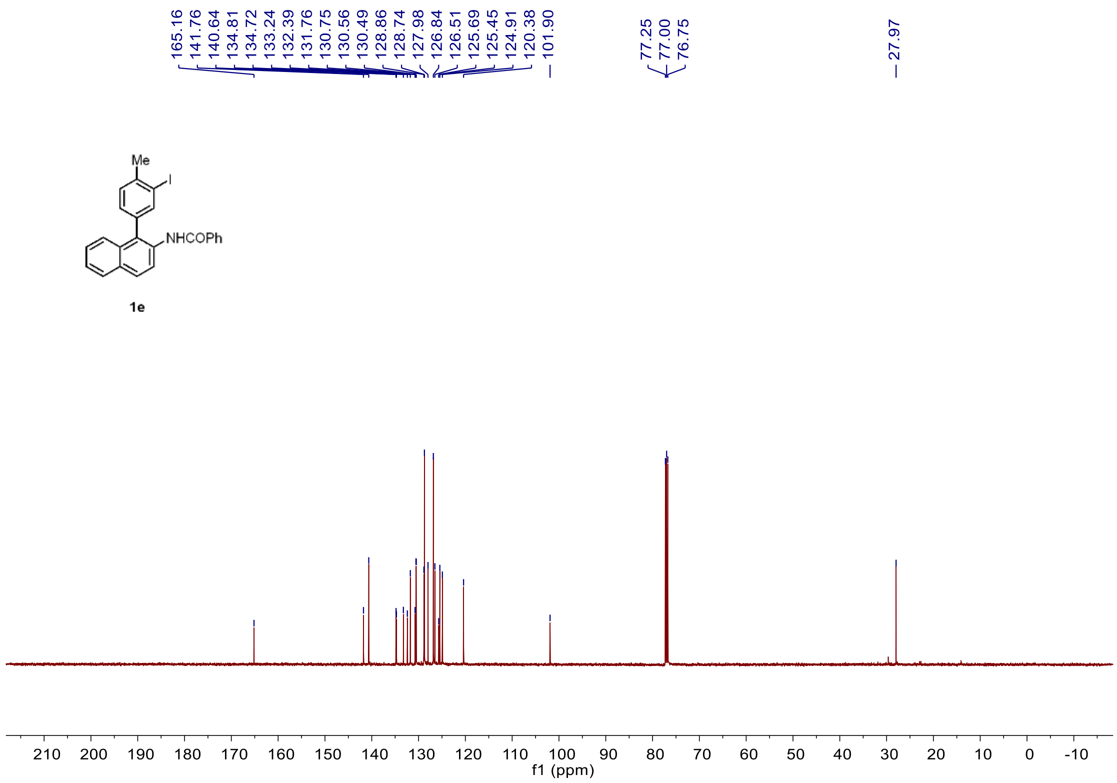

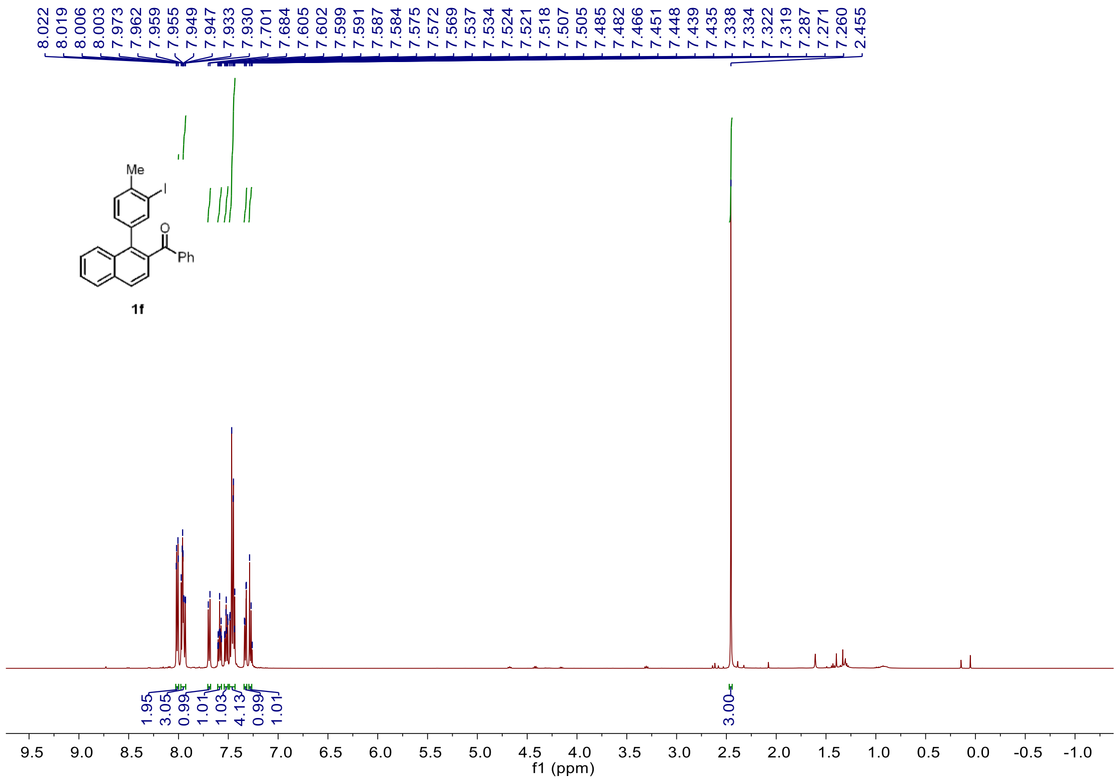
8.022 8.019 8.006 8.003 7.973 7.962 7.959 7.955 7.949 7.947 7.933 7.930 7.701 7.684 7.605 7.602 7.599 7.591 7.587 7.584 7.575 7.572 7.569 7.537 7.534 7.524 7.521 7.518 7.507 7.505 7.485 7.482 7.466 7.451 7.448 7.439 7.435 7.338 7.334 7.322 7.319 7.287 7.271 7.260 2.455
Me
I
O
Ph
1f
1.95 3.05 0.99 1.01 1.03 4.13 0.99 1.01
3.00
9.5 9.0 8.5 8.0 7.5 7.0 6.5 6.0 5.5 5.0 4.5 4.0 3.5 3.0 2.5 2.0 1.5 1.0 0.5 0.0 -0.5 -1.0
f1 (ppm)

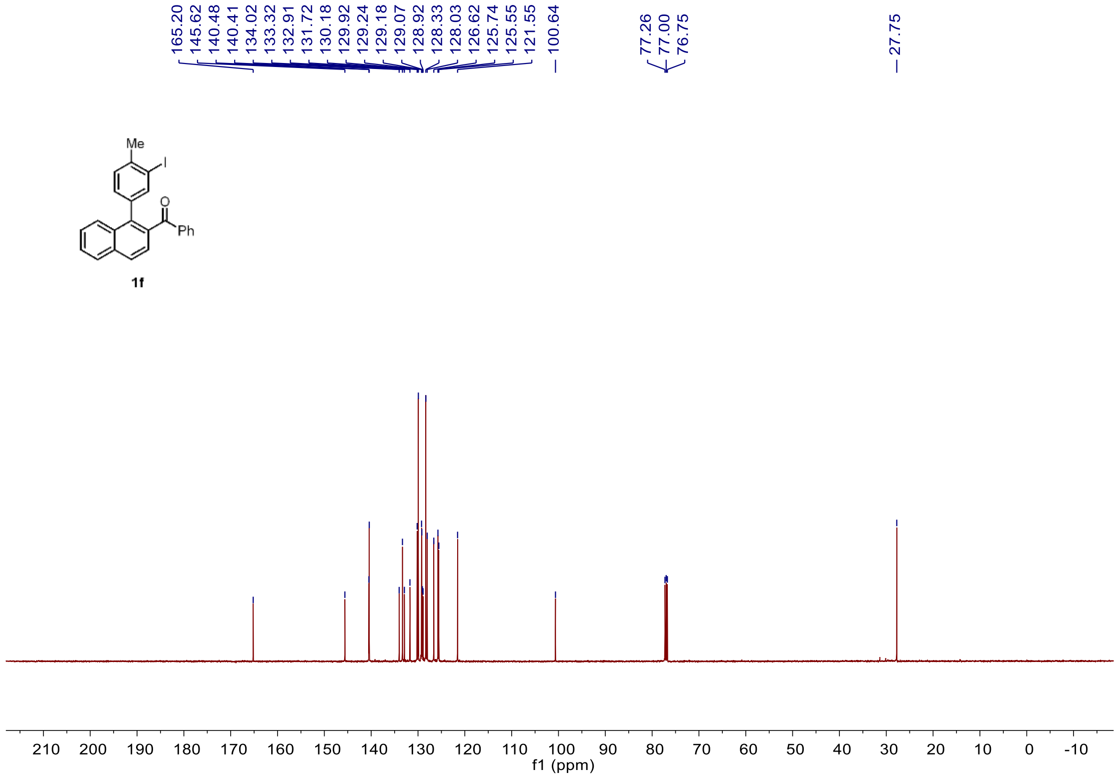
165.20 145.62 140.48 140.41 134.02 133.32 132.91 131.72 130.18 129.92 129.24 129.18 129.07 128.92 128.33 128.03 126.62 125.74 125.55 121.55
100.64
77.26 77.00 76.75
27.75
Me
I
O
Ph
1f
210 200 190 180 170 160 150 140 130 120 110 100 90 80 70 60 50 40 30 20 10 0 -10
f1 (ppm)

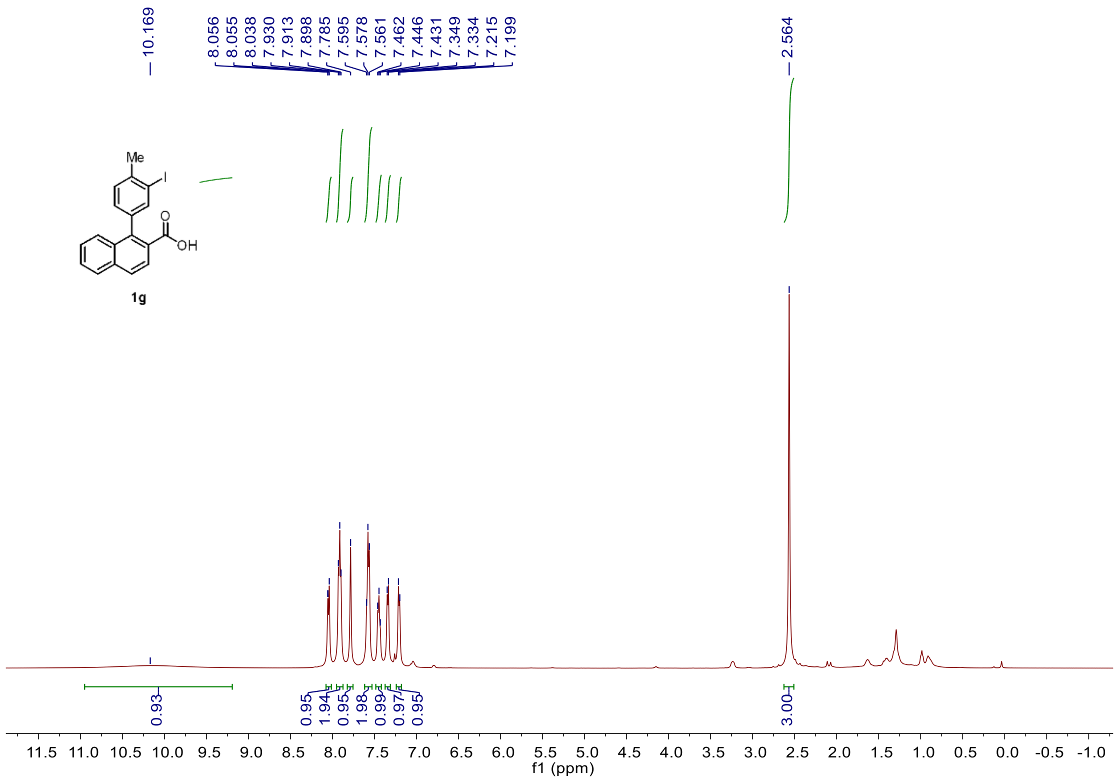

10.169
8.056
8.055
8.038
7.930
7.913
7.898
7.785
7.595
7.578
7.561
7.462
7.446
7.431
7.349
7.334
7.215
7.199
2.564
Me
I
O
OH
1g
0.93
0.95
1.94
0.95
1.98
0.99
0.97
0.95
3.00
11.5 11.0 10.5 10.0 9.5 9.0 8.5 8.0 7.5 7.0 6.5 6.0 5.5 5.0 4.5 4.0 3.5 3.0 2.5 2.0 1.5 1.0 0.5 0.0 -0.5 -1.0
f1 (ppm)

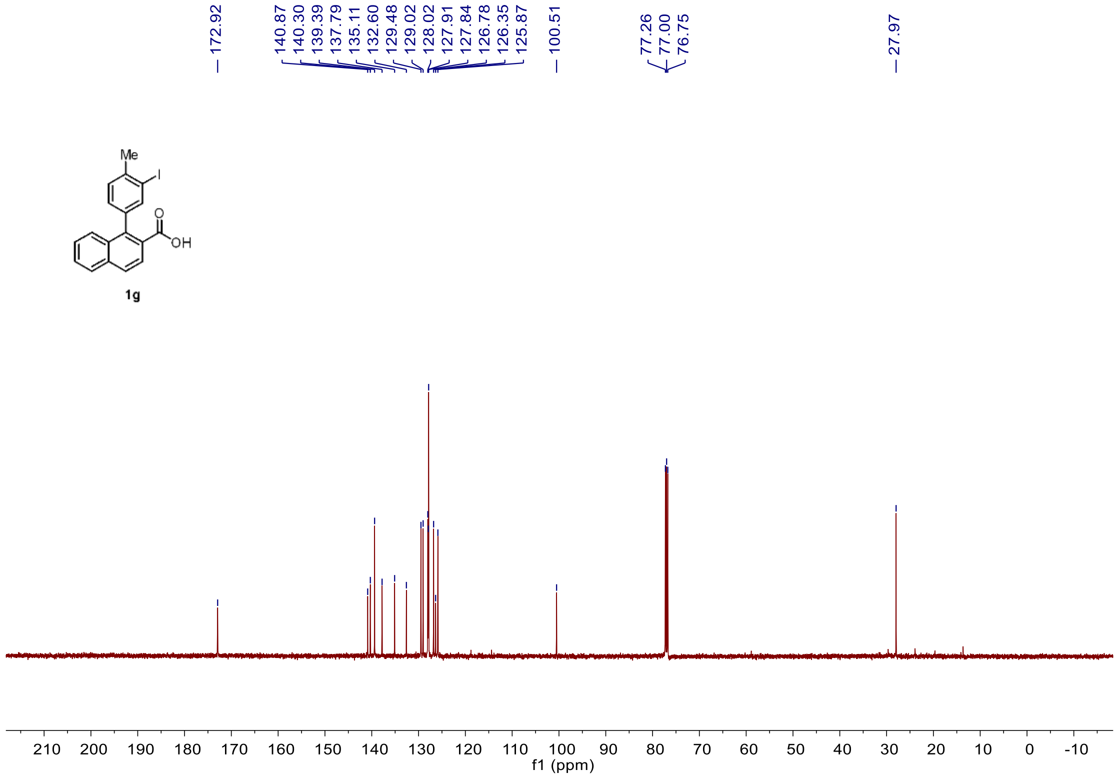

172.92
140.87
140.30
139.39
137.79
135.11
132.60
129.48
129.02
128.02
127.91
127.84
126.78
126.35
125.87
100.51
77.26
77.00
76.75
27.97
Me
I
O
OH
1g
210 200 190 180 170 160 150 140 130 120 110 100 90 80 70 60 50 40 30 20 10 0 -10
f1 (ppm)

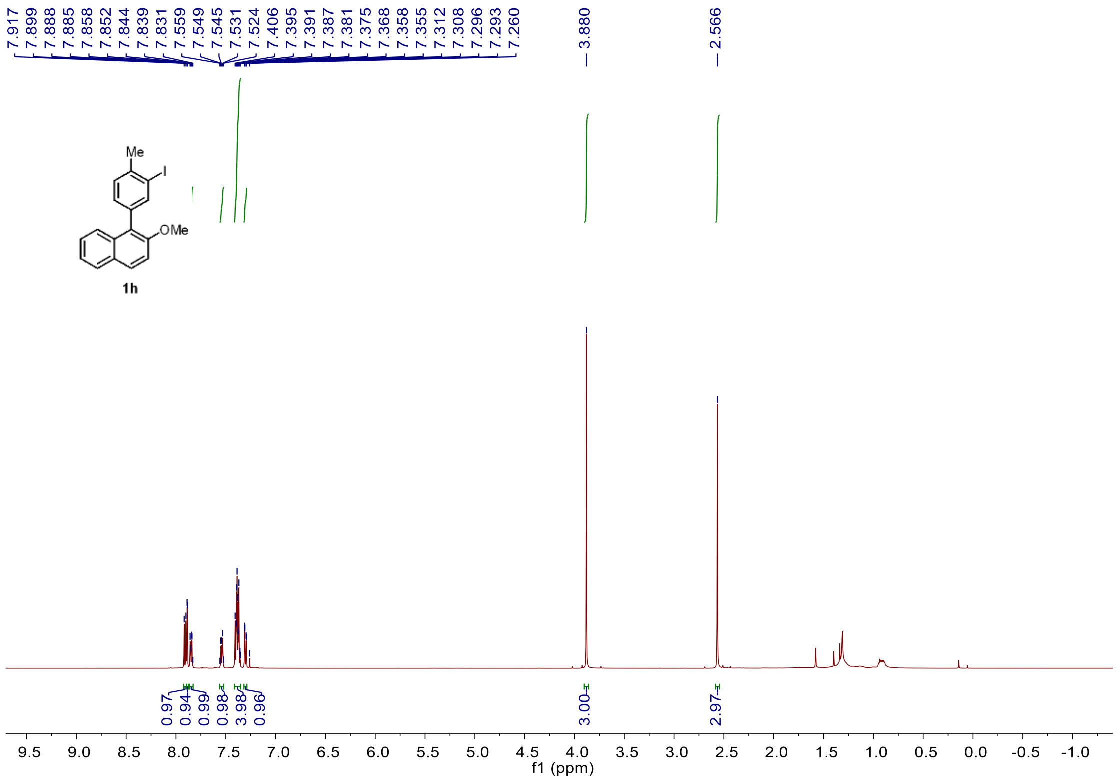
7.917
7.899
7.888
7.885
7.858
7.852
7.844
7.839
7.831
7.559
7.549
7.545
7.531
7.524
7.406
7.395
7.391
7.387
7.381
7.375
7.368
7.358
7.355
7.312
7.308
7.296
7.293
7.260
3.880
2.566
Me
I
OMe
1h
0.97
0.94
0.99
0.98
3.98
0.96
3.00
2.97
9.5 9.0 8.5 8.0 7.5 7.0 6.5 6.0 5.5 5.0 4.5 4.0 3.5 3.0 2.5 2.0 1.5 1.0 0.5 0.0 -0.5 -1.0
f1 (ppm)

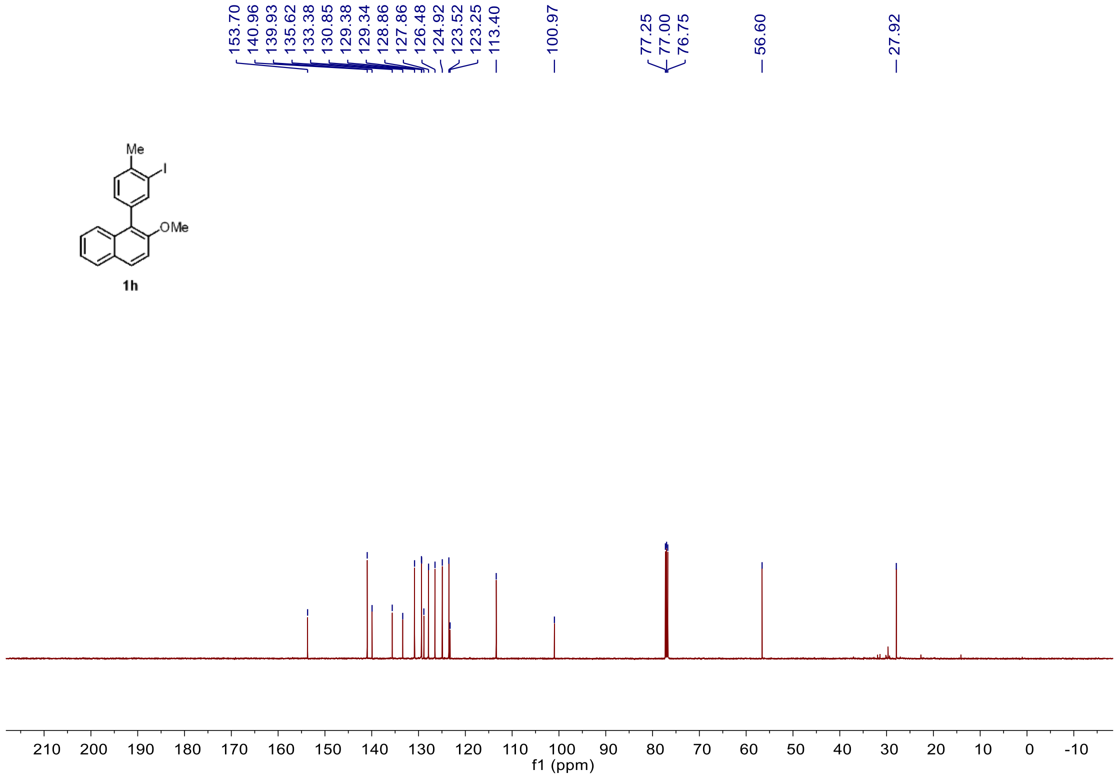
153.70
140.96
139.93
135.62
133.38
130.85
129.38
129.34
128.86
127.86
126.48
124.92
123.52
123.25
113.40
100.97
77.25
77.00
76.75
56.60
27.92
Me
I
OMe
1h
210 200 190 180 170 160 150 140 130 120 110 100 90 80 70 60 50 40 30 20 10 0 -10
f1 (ppm)

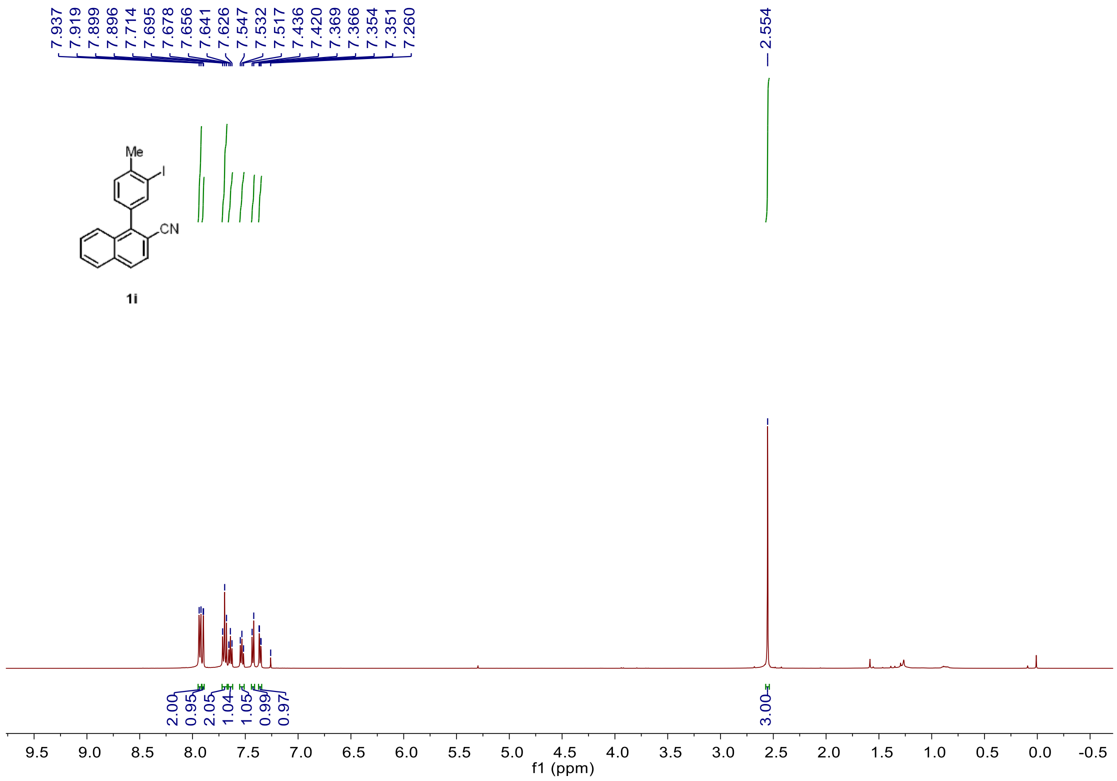
7.937
7.919
7.899
7.896
7.714
7.695
7.678
7.656
7.641
7.626
7.547
7.532
7.517
7.436
7.420
7.369
7.366
7.354
7.351
7.260
2.554
Me
I
CN
1i
2.00
0.95
2.05
1.04
1.05
0.99
0.97
3.00
9.5
9.0
8.5
8.0
7.5
7.0
6.5
6.0
5.5
5.0
4.5
4.0
3.5
3.0
2.5
2.0
1.5
1.0
0.5
0.0
-0.5
f1 (ppm)

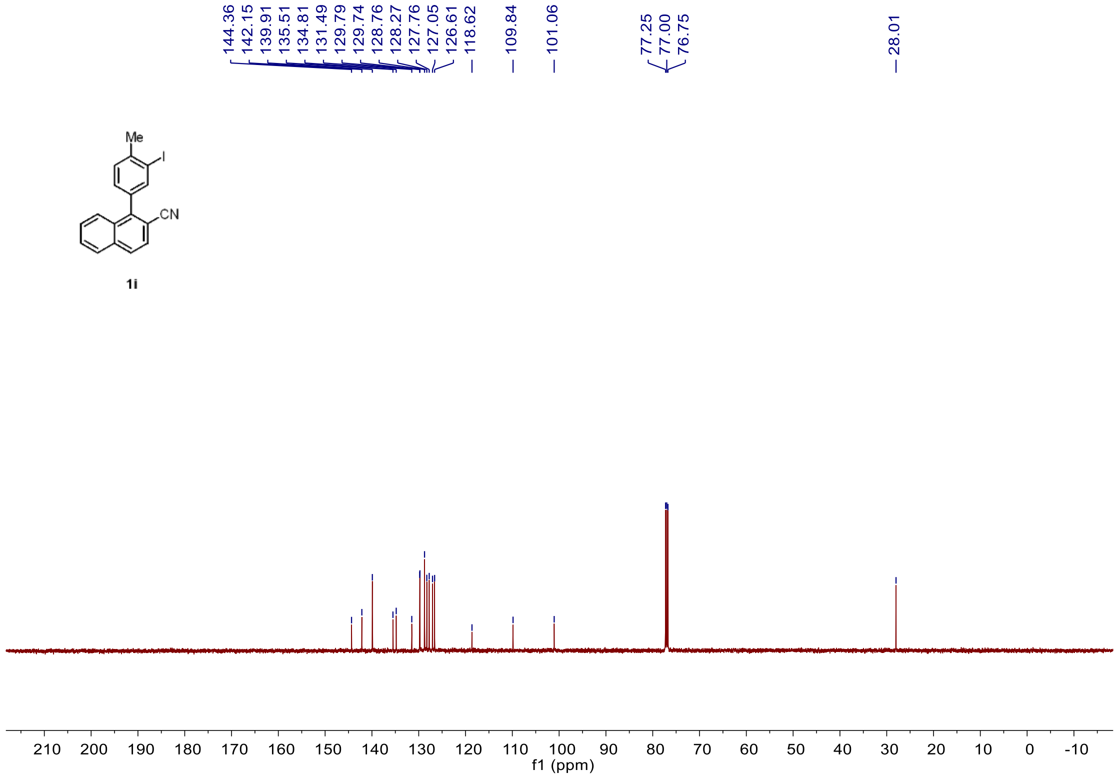
144.36
142.15
139.91
135.51
134.81
131.49
129.79
129.74
128.76
128.27
127.76
127.05
126.61
118.62
109.84
101.06
77.25
77.00
76.75
28.01
Me
I
CN
1i
210
200
190
180
170
160
150
140
130
120
110
100
90
80
70
60
50
40
30
20
10
0
-10
f1 (ppm)

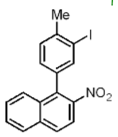

1j

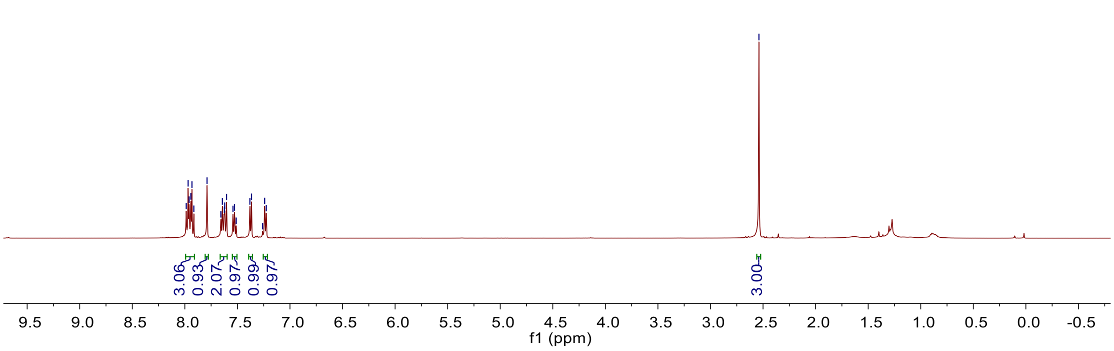

7.987
7.969
7.959
7.943
7.932
7.914
7.789
7.657
7.643
7.624
7.621
7.603
7.542
7.528
7.511
7.381
7.366
7.260
7.241
7.225
2.539
3.06
0.93
2.07
0.97
0.99
0.97
3.00
f1 (ppm)

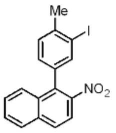

1j

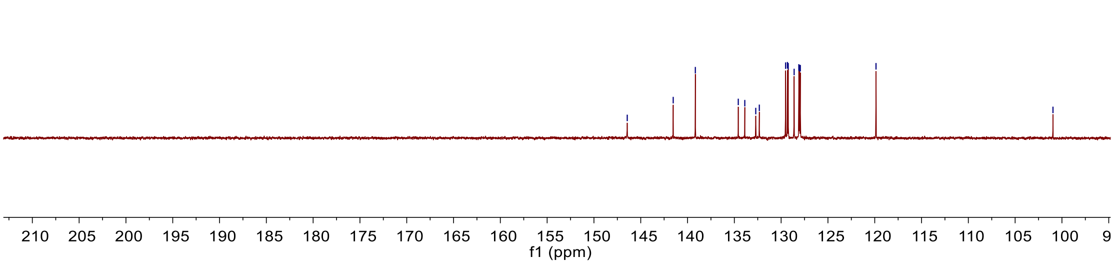

146.44
141.53
139.17
134.58
133.88
132.71
132.33
129.52
129.33
129.24
128.61
128.11
128.04
127.95
119.86
100.96
f1 (ppm)

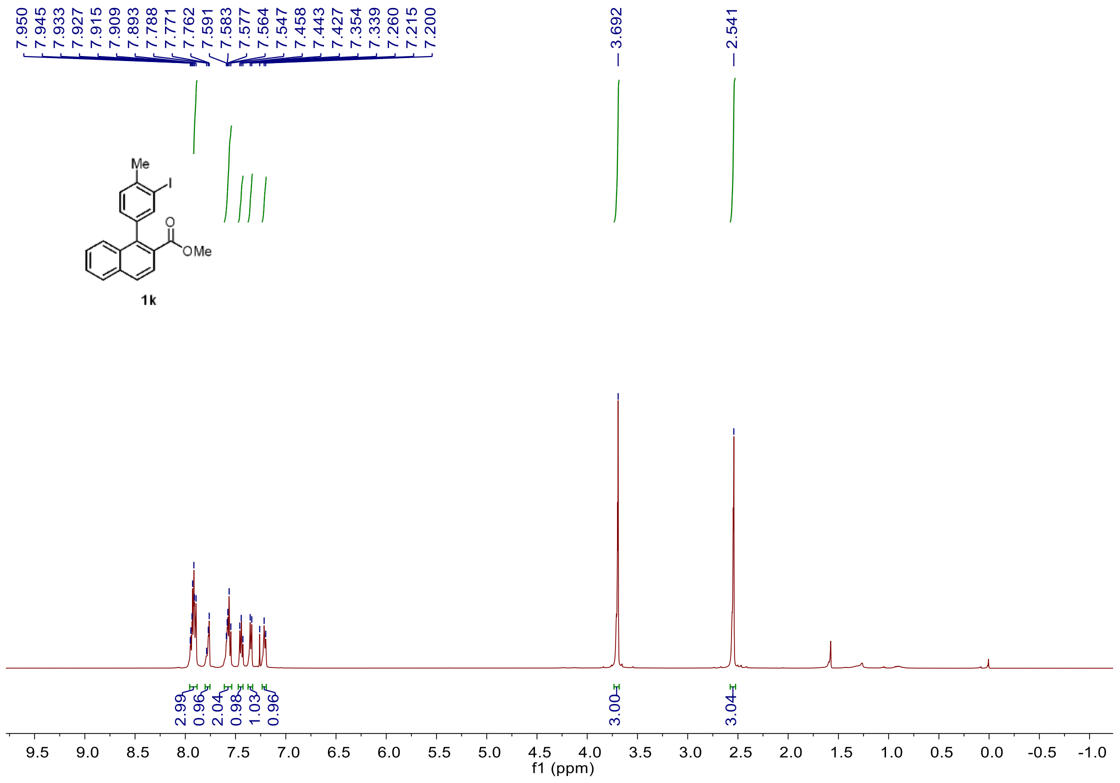
7.950
7.945
7.933
7.927
7.915
7.909
7.893
7.788
7.771
7.762
7.591
7.583
7.577
7.564
7.547
7.458
7.443
7.427
7.354
7.339
7.260
7.215
7.200
3.692
2.541
Me
I
O
OMe
1k
2.99
0.96
2.04
0.98
1.03
0.96
3.00
3.04
9.5 9.0 8.5 8.0 7.5 7.0 6.5 6.0 5.5 5.0 4.5 4.0 3.5 3.0 2.5 2.0 1.5 1.0 0.5 0.0 -0.5 -1.0
f1 (ppm)

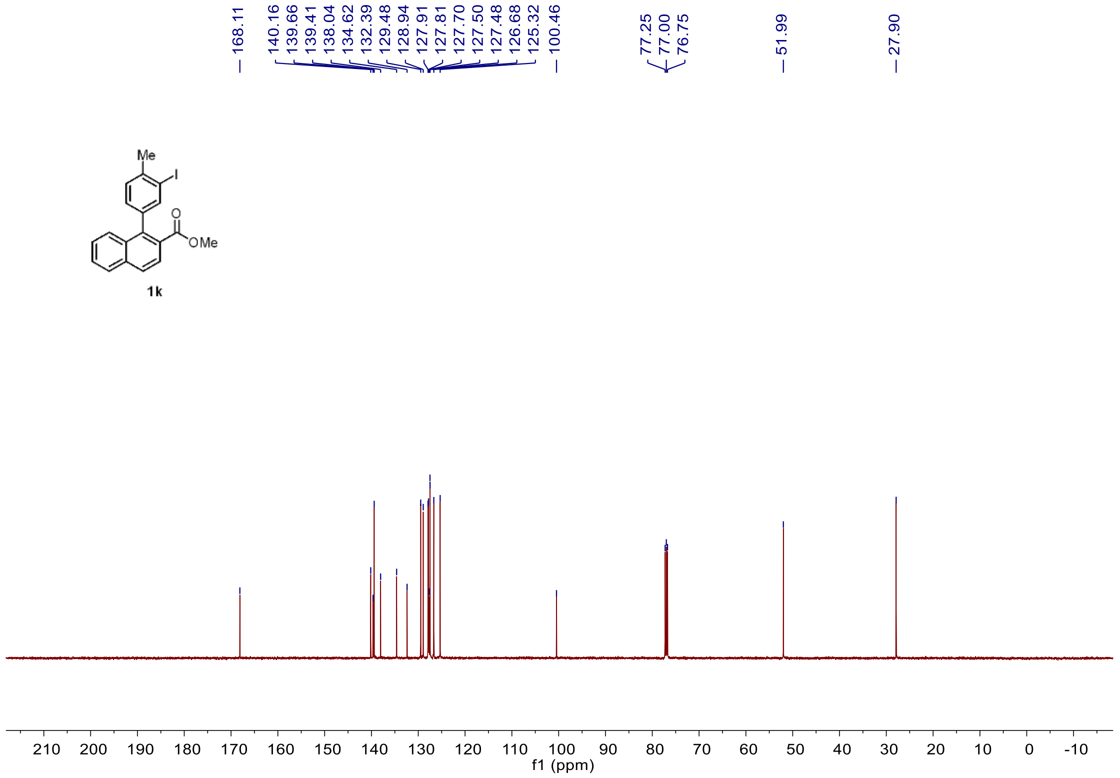
168.11
140.16
139.66
139.41
138.04
134.62
132.39
129.48
128.94
127.91
127.81
127.70
127.50
127.48
126.68
125.32
100.46
77.25
77.00
76.75
51.99
27.90
Me
I
O
OMe
1k
210 200 190 180 170 160 150 140 130 120 110 100 90 80 70 60 50 40 30 20 10 0 -10
f1 (ppm)

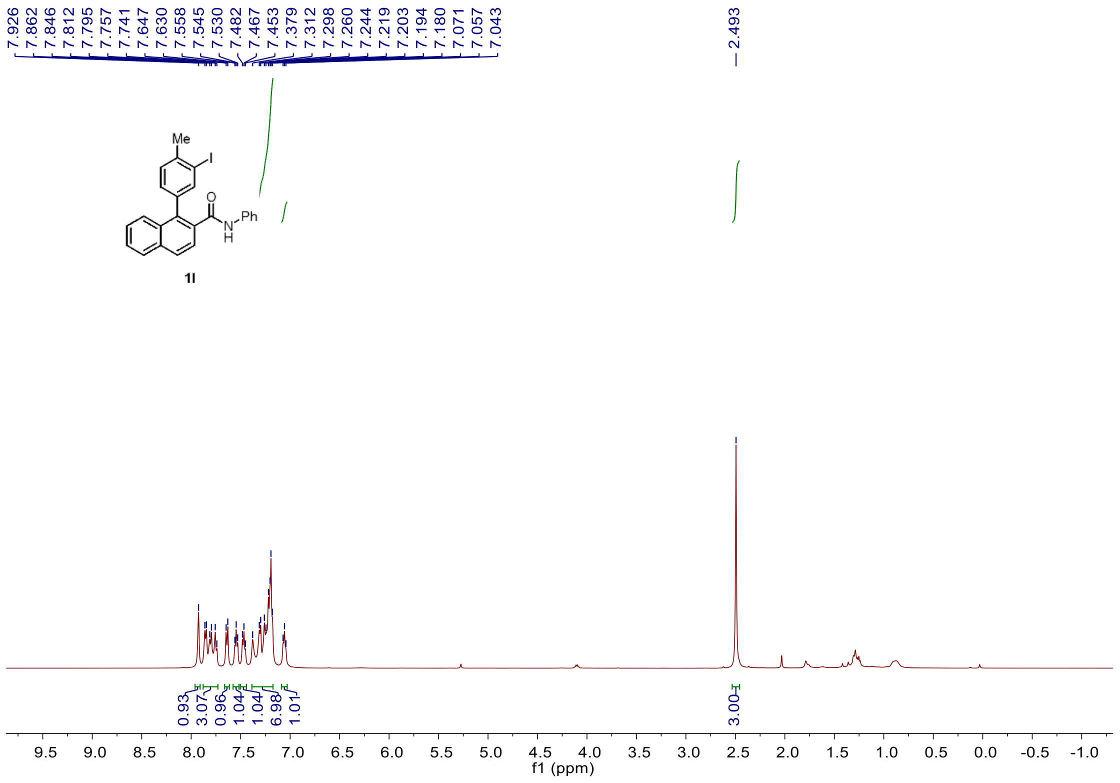
7.926 7.862 7.846 7.812 7.795 7.757 7.741 7.647 7.630 7.558 7.545 7.530 7.482 7.467 7.453 7.379 7.312 7.298 7.260 7.244 7.219 7.203 7.194 7.180 7.071 7.057 7.043
2.493
Me
I
O
N
H
Ph
1l
0.93 3.07 0.96 1.04 1.04 6.98 1.01
3.00
9.5 9.0 8.5 8.0 7.5 7.0 6.5 6.0 5.5 5.0 4.5 4.0 3.5 3.0 2.5 2.0 1.5 1.0 0.5 0.0 -0.5 -1.0
f1 (ppm)

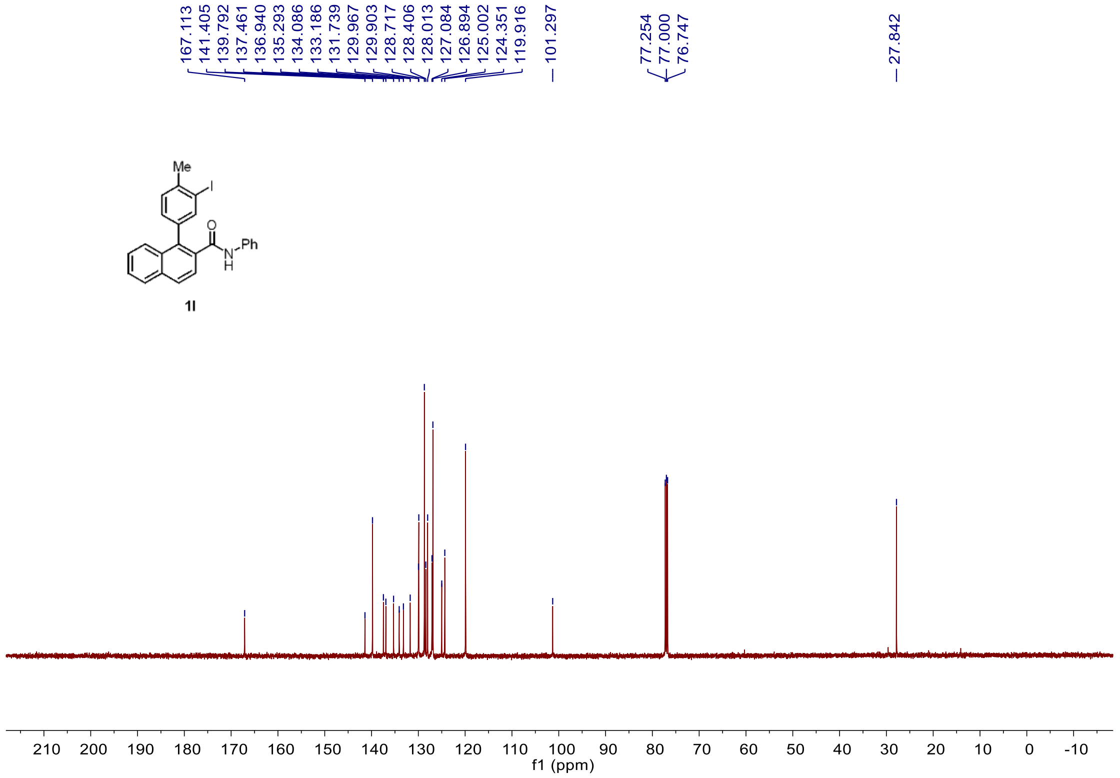
167.113 141.405 139.792 137.461 136.940 135.293 134.086 133.186 131.739 129.967 129.903 128.717 128.406 128.013 127.084 126.894 125.002 124.351 119.916
101.297
77.254 77.000 76.747
27.842
Me
I
O
N
H
Ph
1l
210 200 190 180 170 160 150 140 130 120 110 100 90 80 70 60 50 40 30 20 10 0 -10
f1 (ppm)

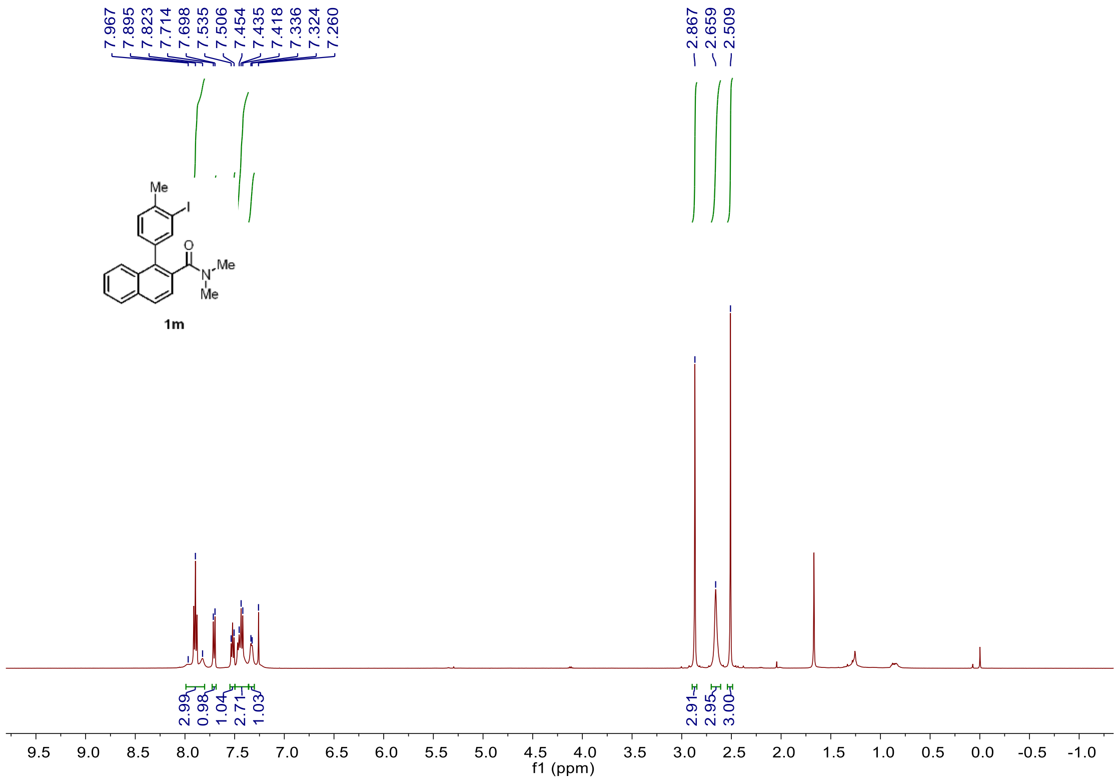
7.967
7.895
7.823
7.714
7.698
7.535
7.506
7.454
7.435
7.418
7.336
7.324
7.260
2.867
2.659
2.509
Me
I
O
N
Me
Me
1m
2.99
0.98
1.04
2.71
1.03
2.91
2.95
3.00
9.5 9.0 8.5 8.0 7.5 7.0 6.5 6.0 5.5 5.0 4.5 4.0 3.5 3.0 2.5 2.0 1.5 1.0 0.5 0.0 -0.5 -1.0
f1 (ppm)

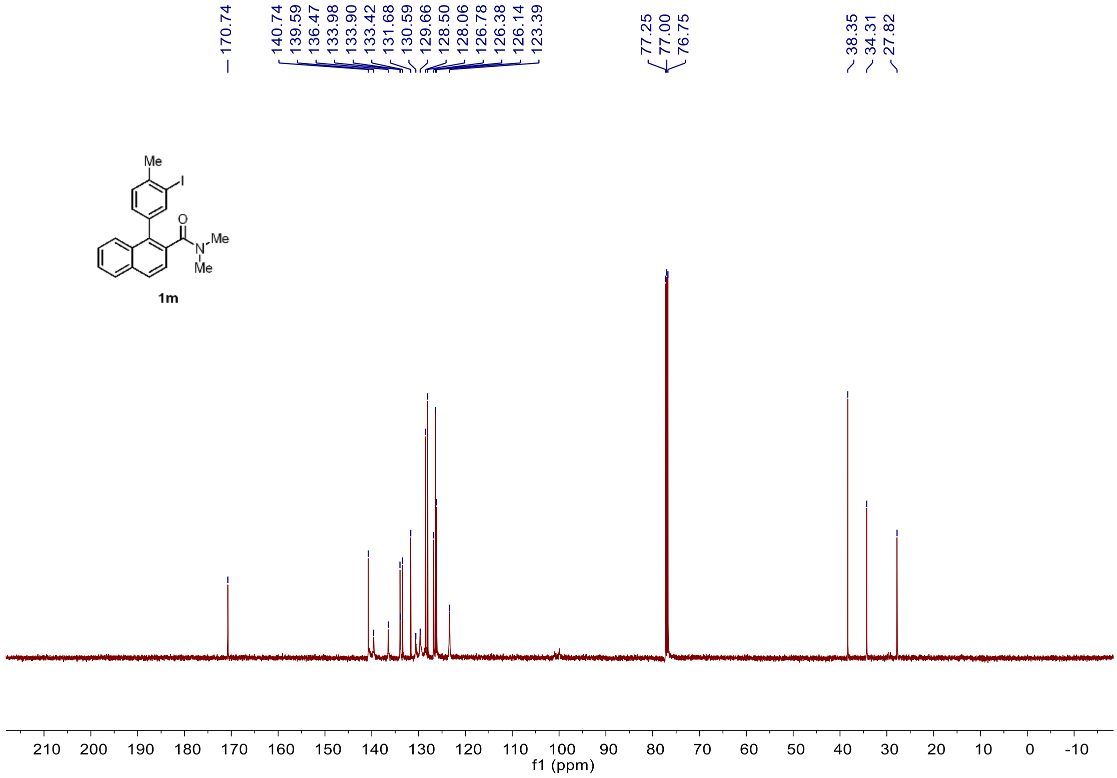
170.74
140.74
139.59
136.47
133.98
133.90
133.42
131.68
130.59
129.66
128.50
128.06
126.78
126.38
126.14
123.39
77.25
77.00
76.75
38.35
34.31
27.82
Me
I
O
N
Me
Me
1m
210 200 190 180 170 160 150 140 130 120 110 100 90 80 70 60 50 40 30 20 10 0 -10
f1 (ppm)

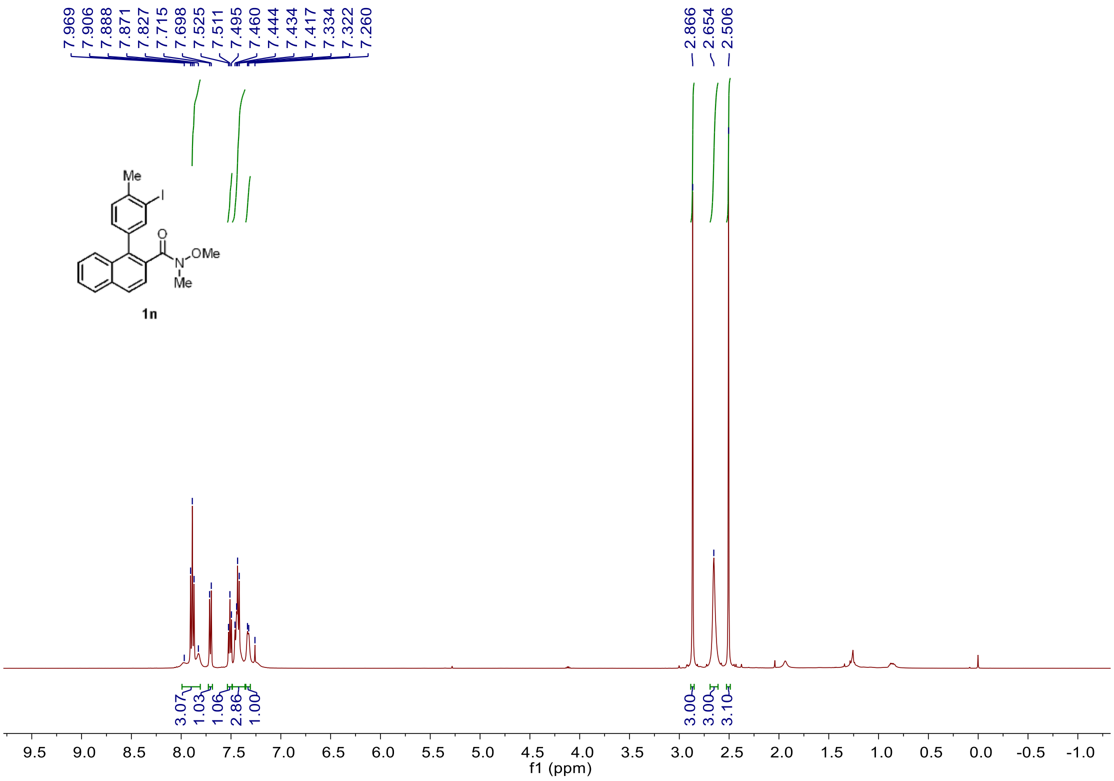
7.969
7.906
7.888
7.871
7.827
7.715
7.698
7.525
7.511
7.495
7.460
7.444
7.434
7.417
7.334
7.322
7.260
2.866
2.654
2.506
Me
I
O
N
OMe
Me
1n
3.07
1.03
1.06
2.86
1.00
3.00
3.00
3.10
9.5 9.0 8.5 8.0 7.5 7.0 6.5 6.0 5.5 5.0 4.5 4.0 3.5 3.0 2.5 2.0 1.5 1.0 0.5 0.0 -0.5 -1.0
f1 (ppm)

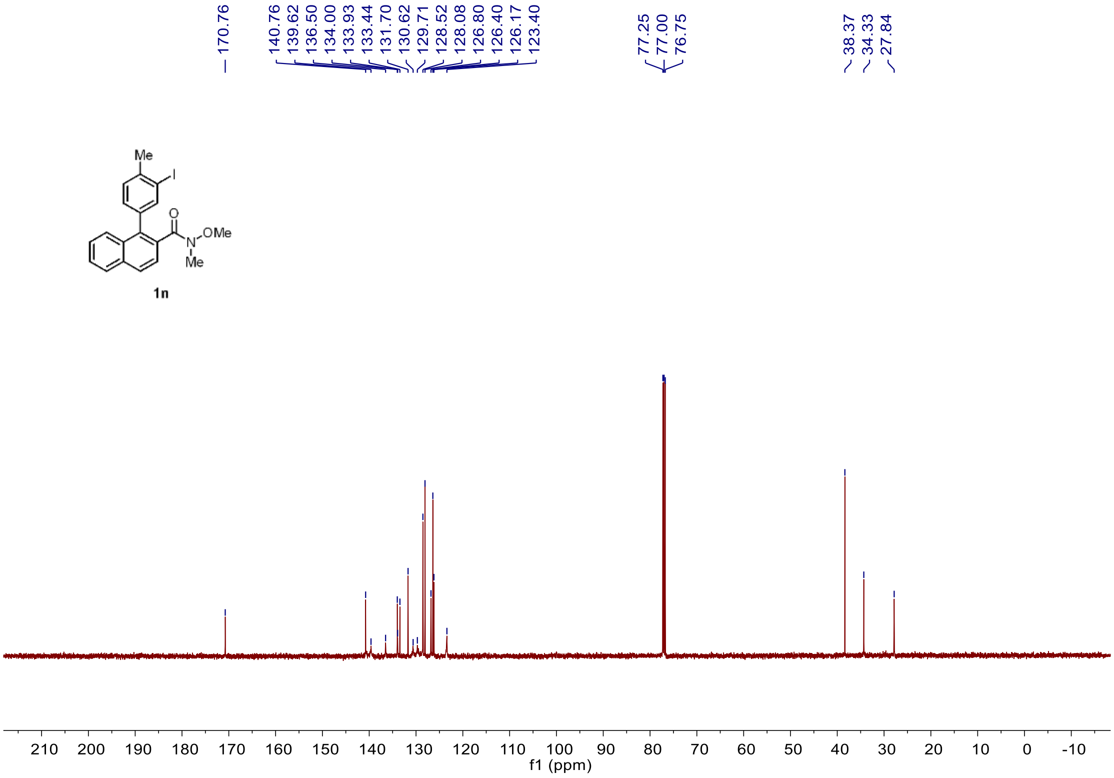
170.76
140.76
139.62
136.50
134.00
133.93
133.44
131.70
130.62
129.71
128.52
128.08
126.80
126.40
126.17
123.40
77.25
77.00
76.75
38.37
34.33
27.84
Me
I
O
N
OMe
Me
1n
210 200 190 180 170 160 150 140 130 120 110 100 90 80 70 60 50 40 30 20 10 0 -10
f1 (ppm)

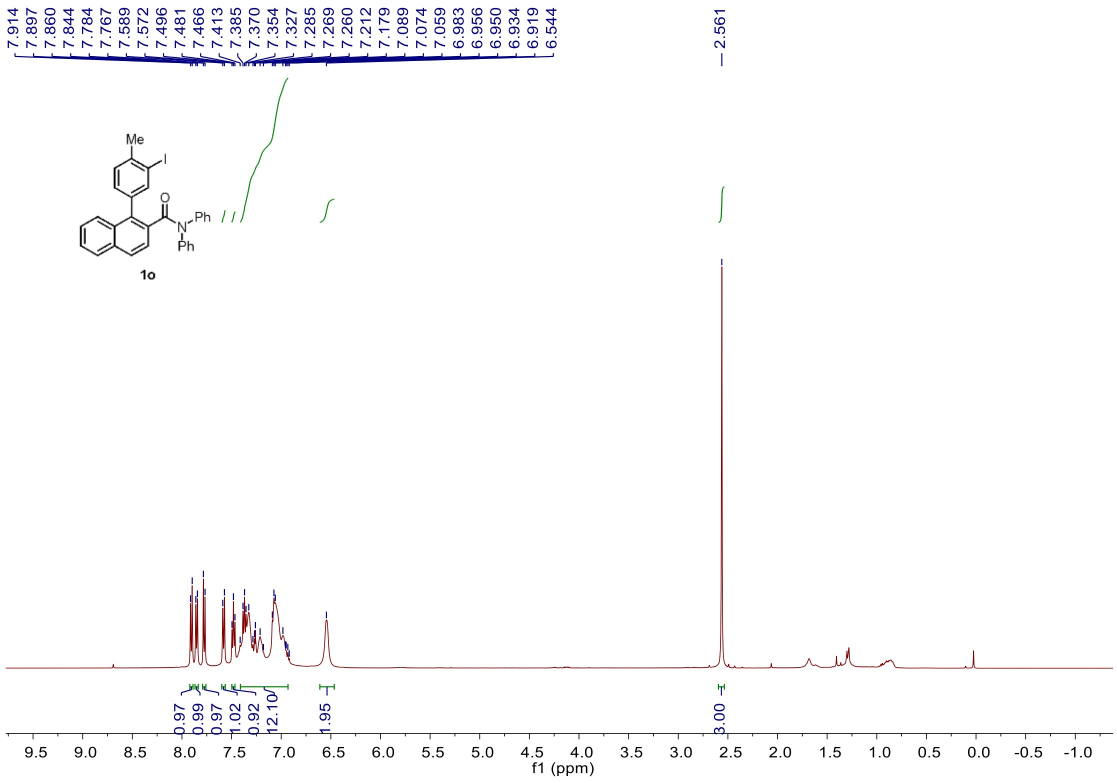
7.914
7.897
7.860
7.844
7.784
7.767
7.589
7.572
7.496
7.481
7.466
7.413
7.385
7.370
7.354
7.327
7.285
7.269
7.260
7.212
7.179
7.089
7.074
7.059
6.983
6.956
6.950
6.934
6.919
6.544
2.561
Me
I
O
N
Ph
Ph
1o
0.97
0.99
0.97
1.02
0.92
12.10
1.95
3.00
9.5 9.0 8.5 8.0 7.5 7.0 6.5 6.0 5.5 5.0 4.5 4.0 3.5 3.0 2.5 2.0 1.5 1.0 0.5 0.0 -0.5 -1.0
f1 (ppm)

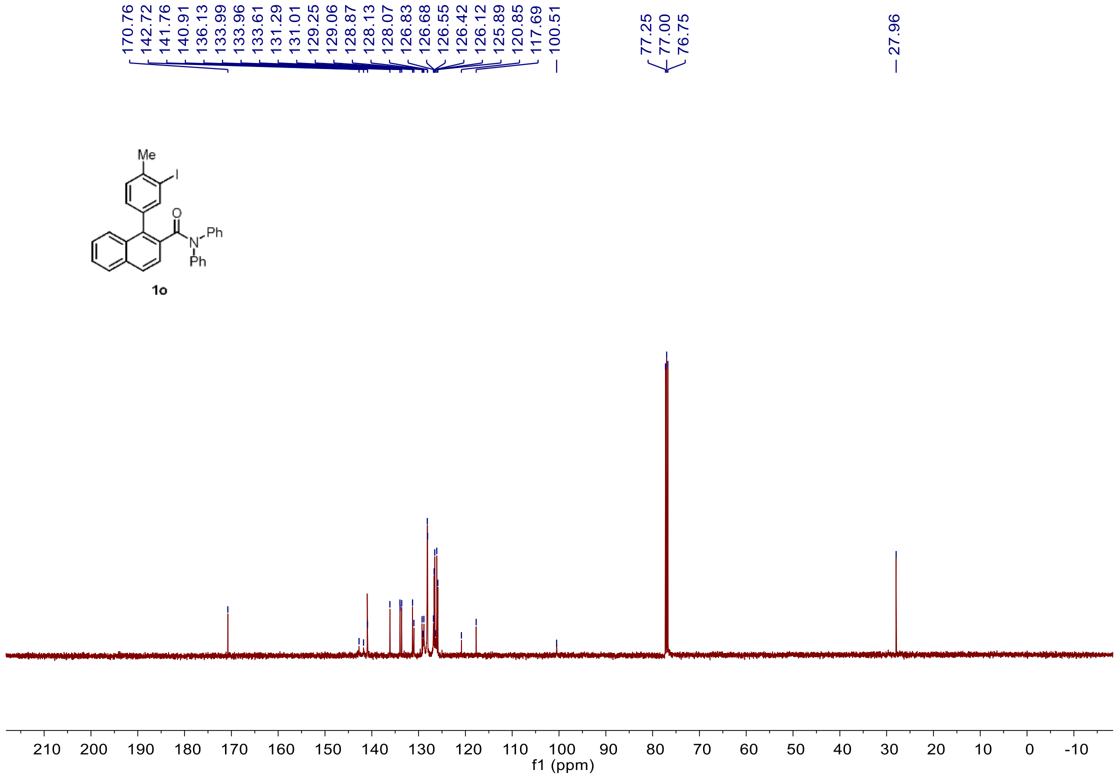
170.76
142.72
141.76
140.91
136.13
133.99
133.96
133.61
131.29
131.01
129.25
129.06
128.87
128.13
128.07
126.83
126.68
126.55
126.42
126.12
125.89
120.85
117.69
100.51
77.25
77.00
76.75
27.96
Me
I
O
N
Ph
Ph
1o
210 200 190 180 170 160 150 140 130 120 110 100 90 80 70 60 50 40 30 20 10 0 -10
f1 (ppm)

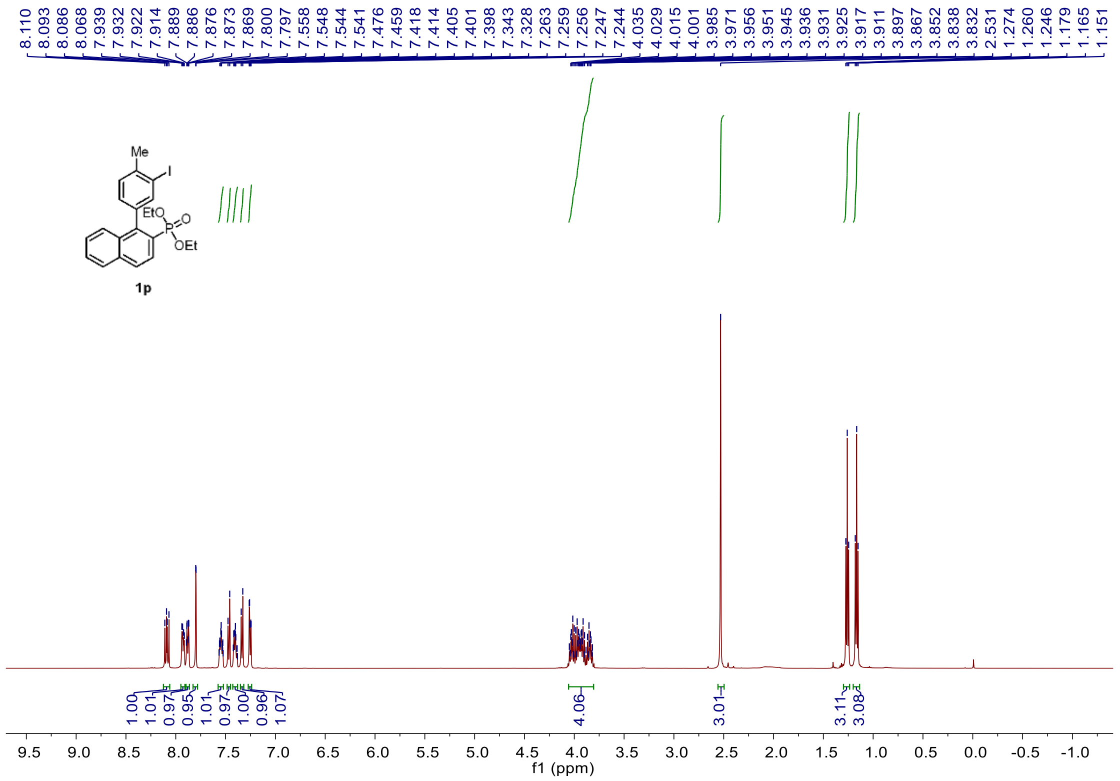
8.110 8.093 8.086 8.068 7.939 7.932 7.922 7.914 7.889 7.886 7.876 7.873 7.869 7.800 7.797 7.558 7.548 7.544 7.541 7.476 7.459 7.418 7.414 7.405 7.401 7.398 7.343 7.328 7.263 7.259 7.256 7.247 7.244 4.035 4.029 4.015 4.001 3.985 3.971 3.956 3.951 3.945 3.936 3.931 3.925 3.917 3.911 3.897 3.867 3.852 3.838 3.832 2.531 1.274 1.260 1.246 1.179 1.165 1.151
Me
I
EtO
P
O
OEt
1p
1.00 1.01 0.97 0.95 1.01 0.97 1.00 0.96 1.07
4.06
3.01
3.11 3.08
9.5 9.0 8.5 8.0 7.5 7.0 6.5 6.0 5.5 5.0 4.5 4.0 3.5 3.0 2.5 2.0 1.5 1.0 0.5 0.0 -0.5 -1.0
f1 (ppm)

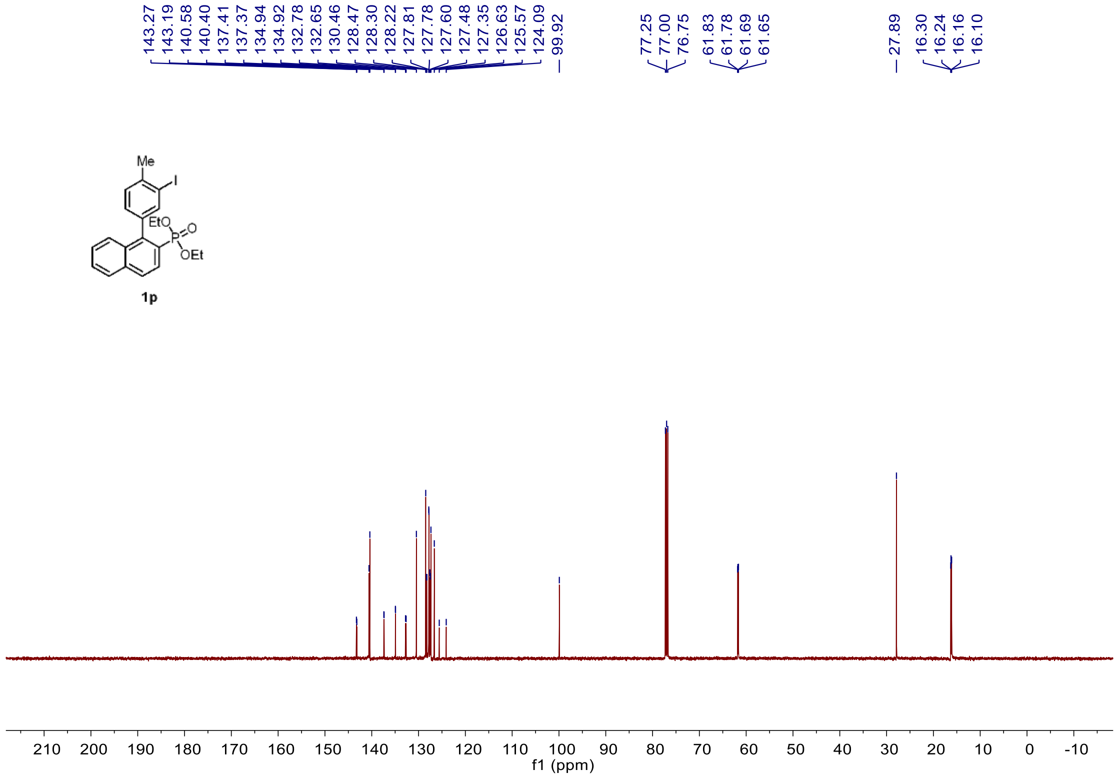
143.27 143.19 140.58 140.40 137.41 137.37 134.94 134.92 132.78 132.65 130.46 128.47 128.30 128.22 127.81 127.78 127.60 127.48 127.35 126.63 125.57 124.09 99.92
77.25 77.00 76.75 61.83 61.78 61.69 61.65
27.89 16.30 16.24 16.16 16.10
Me
I
EtO
P
O
OEt
1p
210 200 190 180 170 160 150 140 130 120 110 100 90 80 70 60 50 40 30 20 10 0 -10
f1 (ppm)

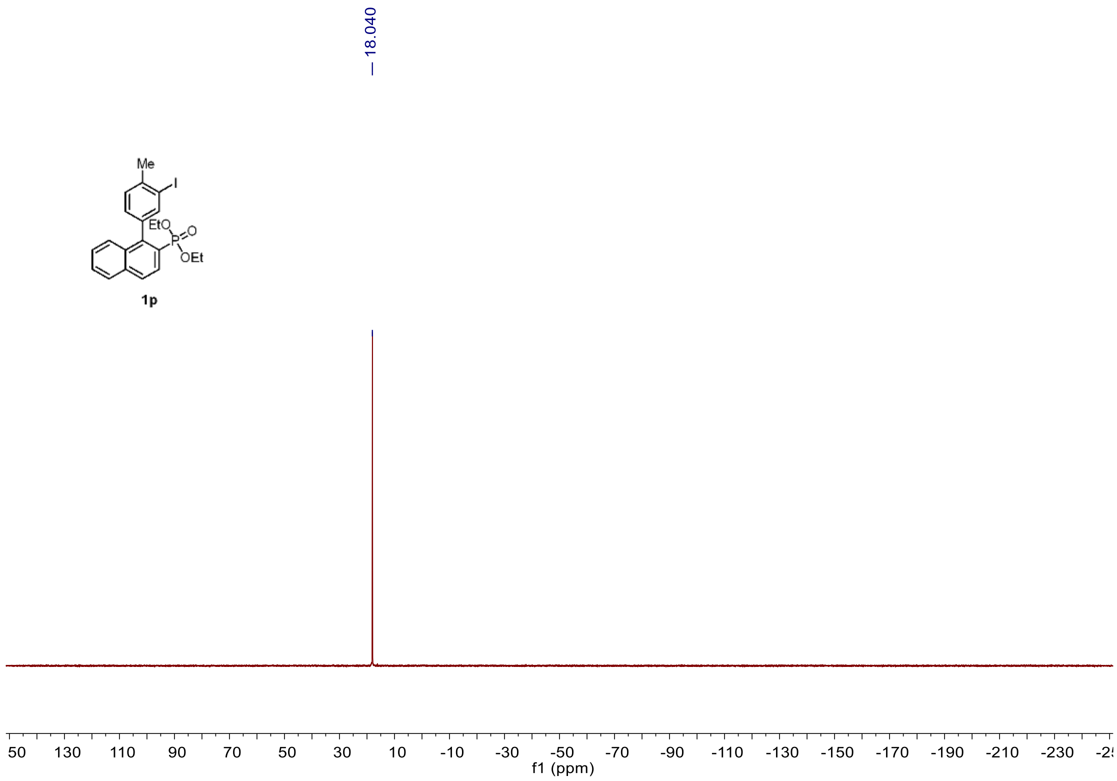
18.040
Me
I
EtO
P=O
OEt
1p
50 130 110 90 70 50 30 10 -10 -30 -50 -70 -90 -110 -130 -150 -170 -190 -210 -230
f1 (ppm)

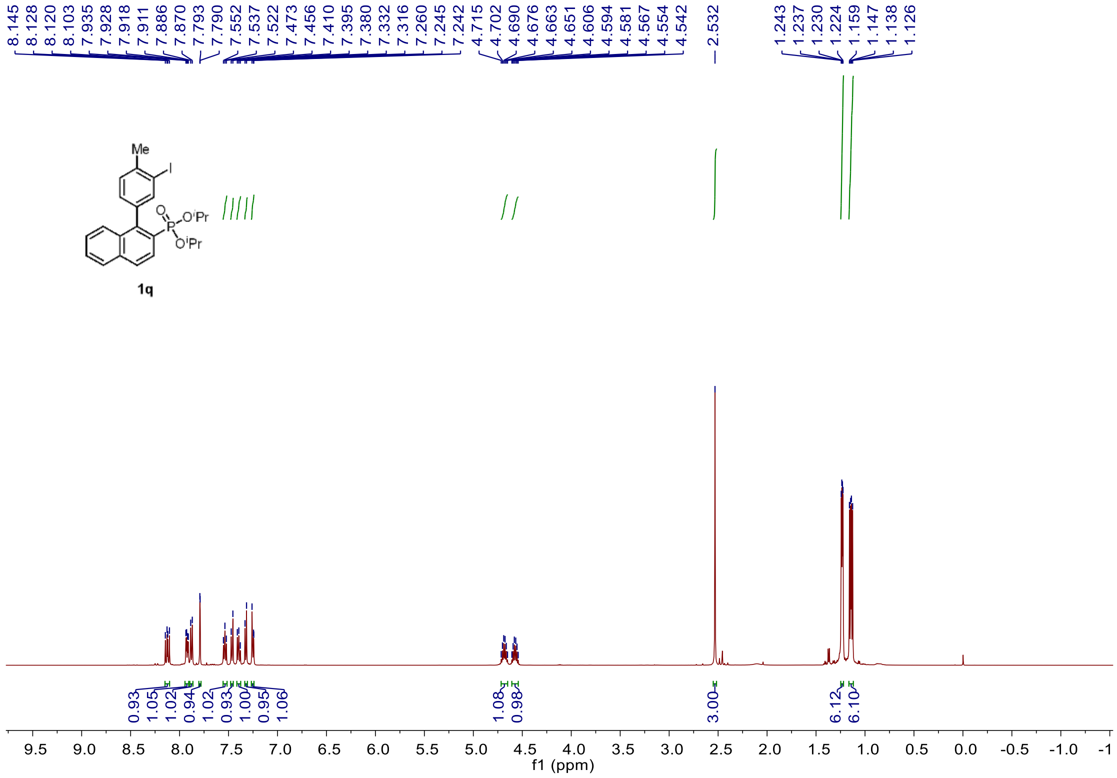
8.145 8.128 8.120 8.103 7.935 7.928 7.918 7.911 7.886 7.870 7.793 7.790 7.552 7.537 7.522 7.473 7.456 7.410 7.395 7.380 7.332 7.316 7.260 7.245 7.242 4.715 4.702 4.690 4.676 4.663 4.651 4.606 4.594 4.581 4.567 4.554 4.542
2.532
1.243 1.237 1.230 1.224 1.159 1.147 1.138 1.126
Me
I
O
P
O^{i}Pr
O^{i}Pr
1q
0.93 1.05 1.02 0.94 1.02 0.93 1.00 0.95 1.06
1.08 0.98
3.00
6.12 6.10
9.5 9.0 8.5 8.0 7.5 7.0 6.5 6.0 5.5 5.0 4.5 4.0 3.5 3.0 2.5 2.0 1.5 1.0 0.5 0.0 -0.5 -1.0
f1 (ppm)

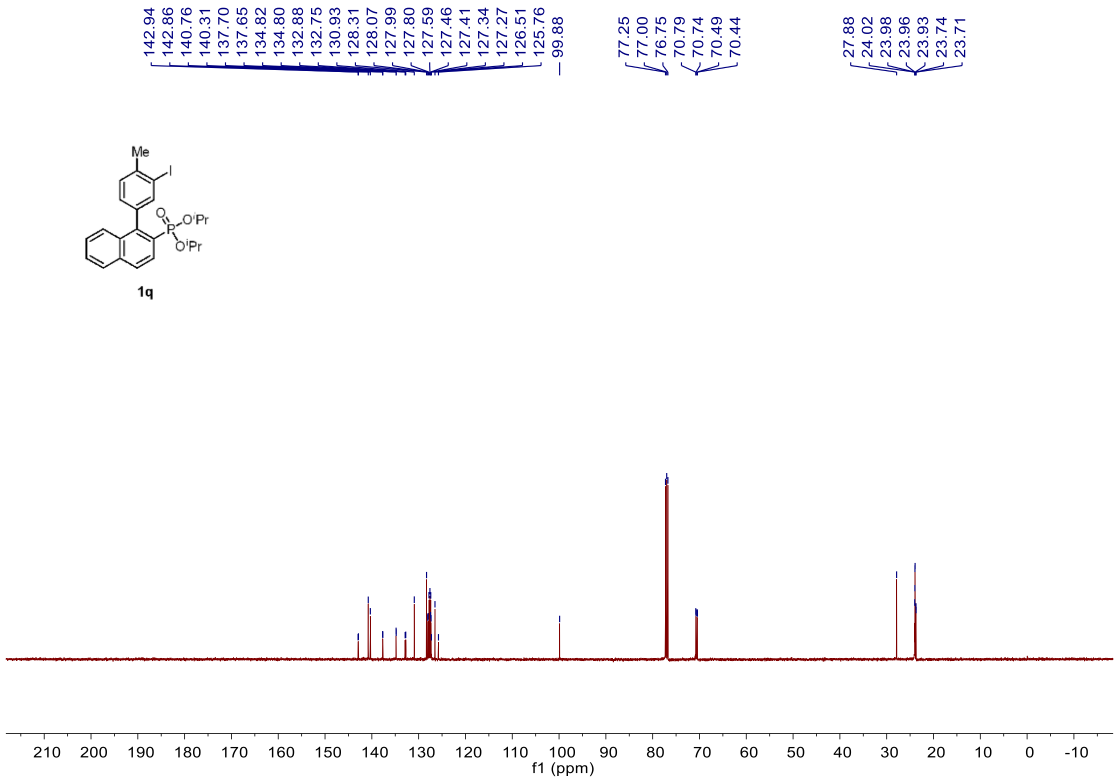
142.94
142.86
140.76
140.31
137.70
137.65
134.82
134.80
132.88
132.75
130.93
128.31
128.07
127.99
127.80
127.59
127.46
127.41
127.34
127.27
126.51
125.76
99.88
77.25
77.00
76.75
70.79
70.74
70.49
70.44
27.88
24.02
23.98
23.96
23.93
23.74
23.71
Me
I
O
P
O^{i}Pr
O^{i}Pr
1q
f1 (ppm)

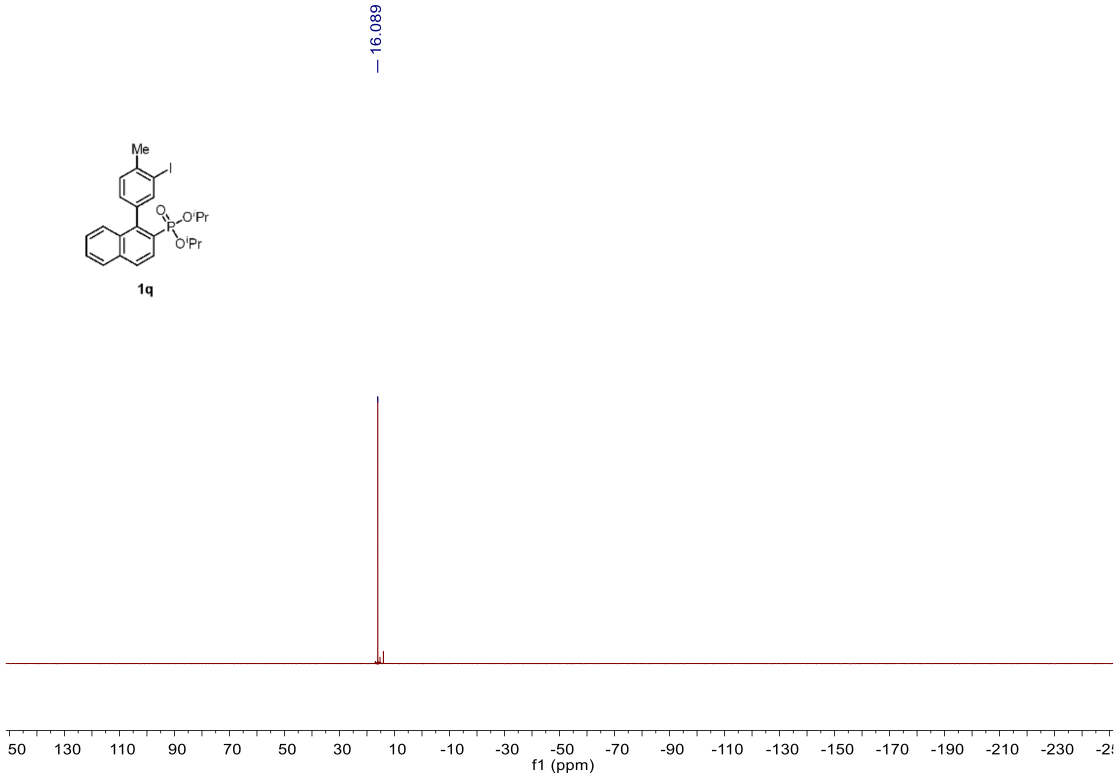
16.089
Me
I
O
P
O^{i}Pr
O^{i}Pr
1q
f1 (ppm)

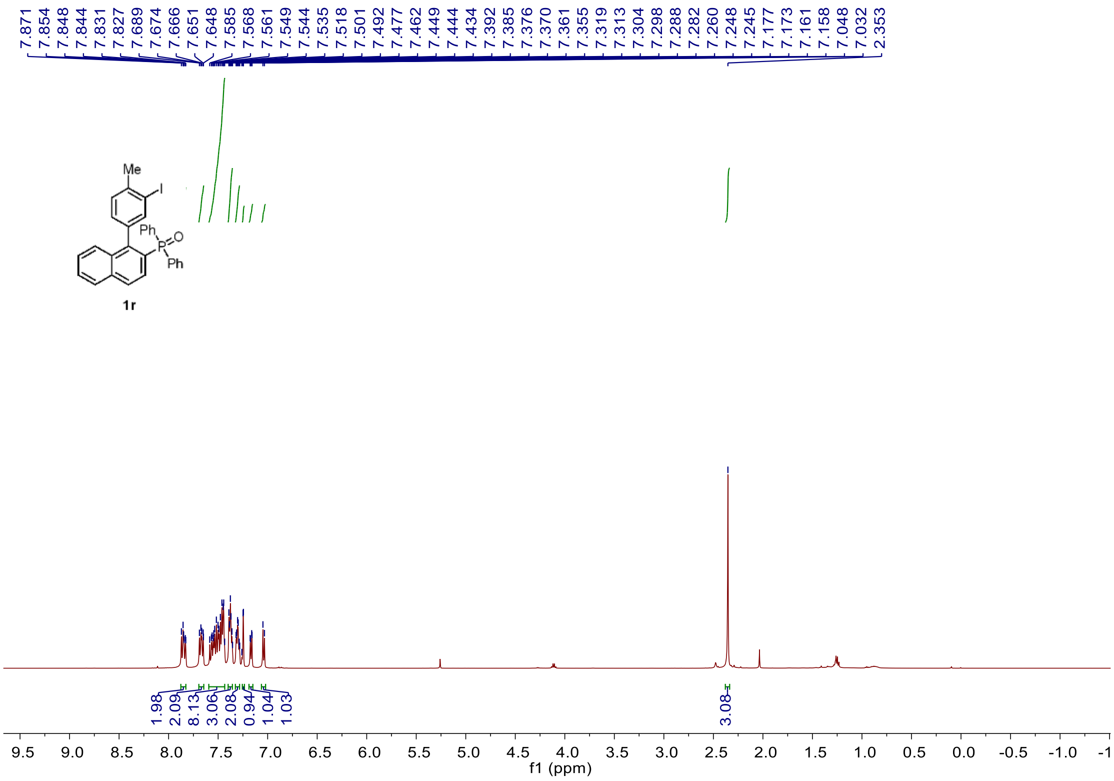
7.871 7.854 7.848 7.844 7.831 7.827 7.689 7.674 7.666 7.651 7.648 7.585 7.568 7.561 7.549 7.544 7.535 7.518 7.501 7.492 7.477 7.462 7.449 7.444 7.434 7.392 7.385 7.376 7.370 7.361 7.355 7.319 7.313 7.304 7.298 7.288 7.282 7.260 7.248 7.245 7.177 7.173 7.161 7.158 7.048 7.032 2.353
Me
I
Ph
P
O
Ph
1r
1.98 2.09 8.13 3.06 2.08 0.94 1.04 1.03
3.08
9.5 9.0 8.5 8.0 7.5 7.0 6.5 6.0 5.5 5.0 4.5 4.0 3.5 3.0 2.5 2.0 1.5 1.0 0.5 0.0 -0.5 -1.0 -1
f1 (ppm)

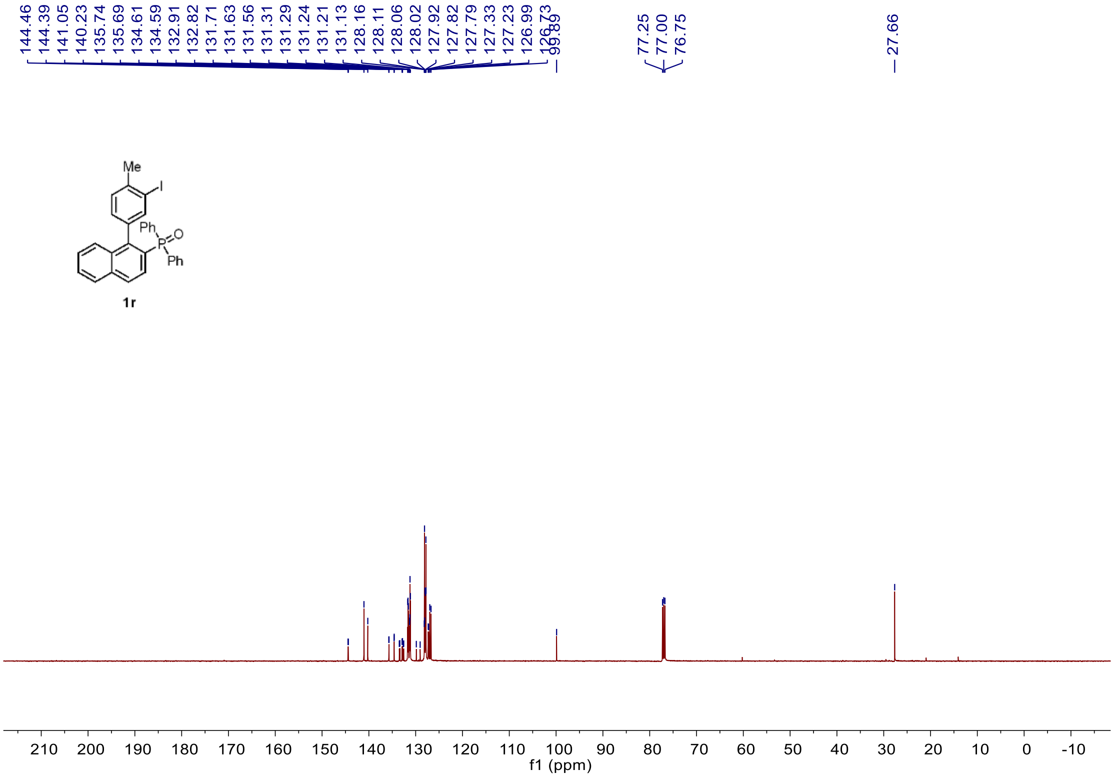
144.46 144.39 141.05 140.23 135.74 135.69 134.61 134.59 132.91 132.82 131.71 131.63 131.56 131.31 131.29 131.24 131.21 131.13 128.16 128.11 128.06 128.02 127.92 127.82 127.79 127.33 127.23 126.99 126.73 99.89
77.25 77.00 76.75
27.66
Me
I
Ph
P
O
Ph
1r
210 200 190 180 170 160 150 140 130 120 110 100 90 80 70 60 50 40 30 20 10 0 -10
f1 (ppm)

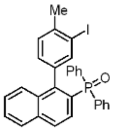

1r

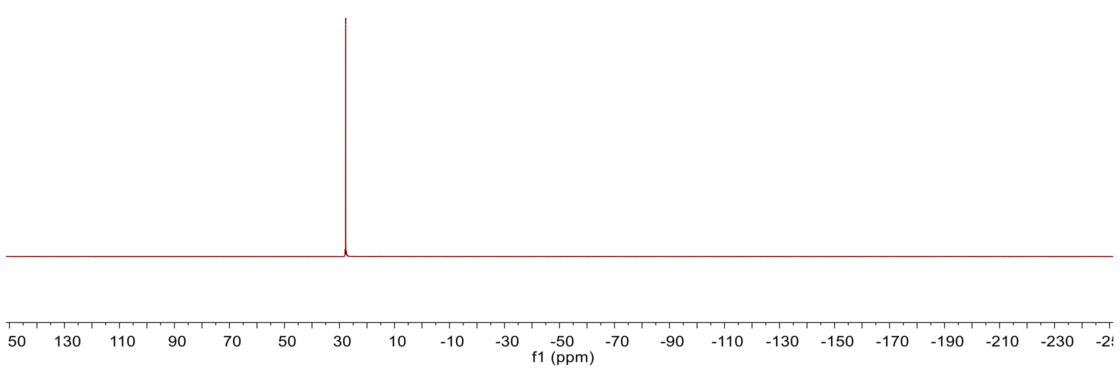

27.729
150 130 110 90 70 50 30 10 -10 -30 -50 -70 -90 -110 -130 -150 -170 -190 -210 -230
f1 (ppm)

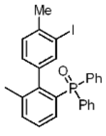

1s

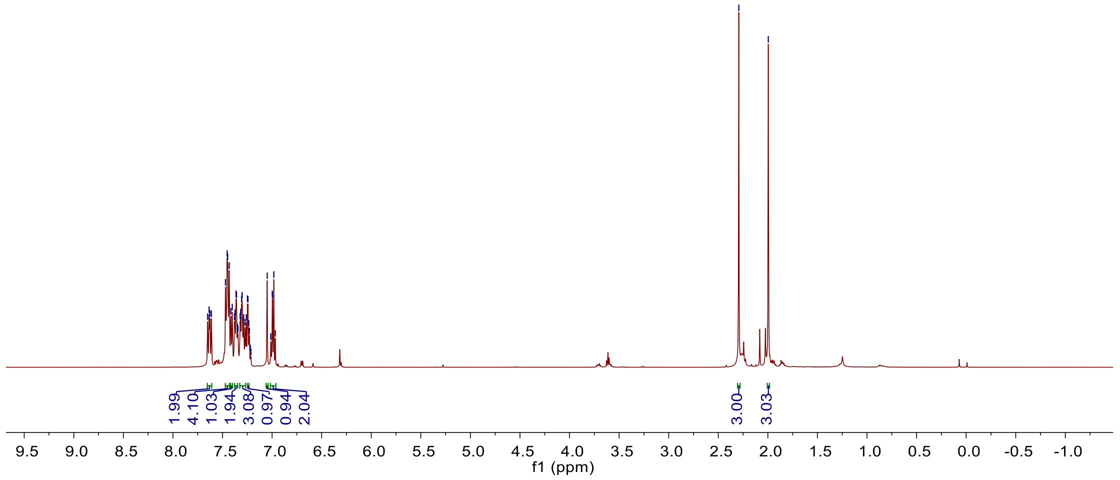

7.650
7.635
7.627
7.612
7.470
7.454
7.448
7.432
7.416
7.402
7.379
7.374
7.364
7.359
7.349
7.344
7.322
7.317
7.308
7.302
7.290
7.286
7.273
7.260
7.249
7.243
7.235
7.229
7.220
7.213
7.049
7.014
6.998
6.996
6.983
6.967
2.293
1.996
1.99
4.10
1.03
1.94
3.08
0.97
0.94
2.04
3.00
3.03
9.5 9.0 8.5 8.0 7.5 7.0 6.5 6.0 5.5 5.0 4.5 4.0 3.5 3.0 2.5 2.0 1.5 1.0 0.5 0.0 -0.5 -1.0
f1 (ppm)

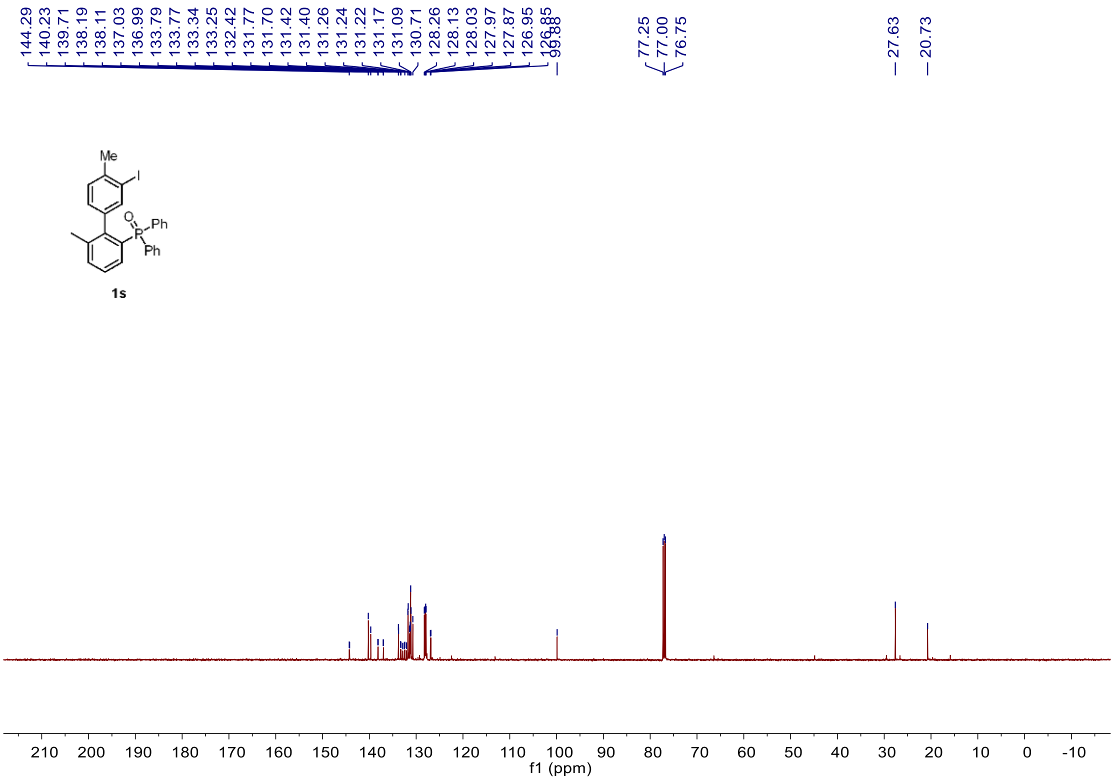
144.29
140.23
139.71
138.19
138.11
137.03
136.99
133.79
133.77
133.34
133.25
132.42
131.77
131.70
131.42
131.40
131.26
131.24
131.22
131.17
131.09
130.71
128.26
128.13
128.03
127.97
127.87
126.95
126.85
99.88
77.25
77.00
76.75
27.63
20.73
Me
I
O
P
Ph
Ph
1s
210 200 190 180 170 160 150 140 130 120 110 100 90 80 70 60 50 40 30 20 10 0 -10
f1 (ppm)

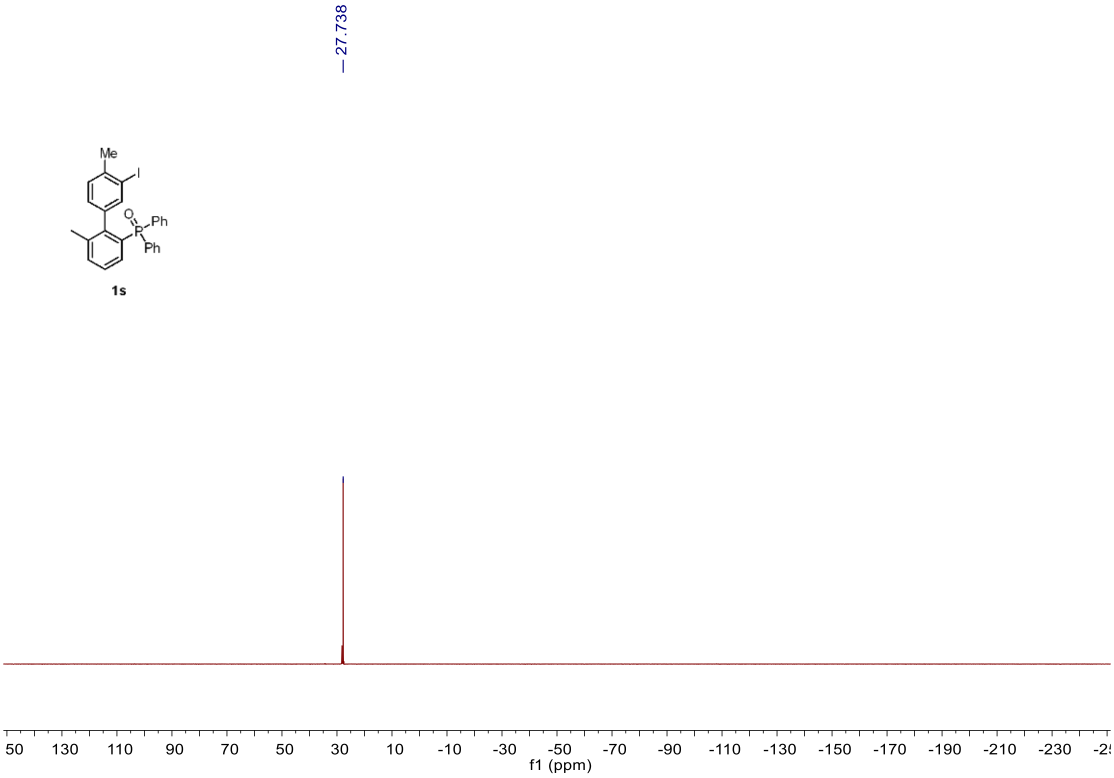
27.738
Me
I
O
P
Ph
Ph
1s
130 110 90 70 50 30 10 -10 -30 -50 -70 -90 -110 -130 -150 -170 -190 -210 -230
f1 (ppm)

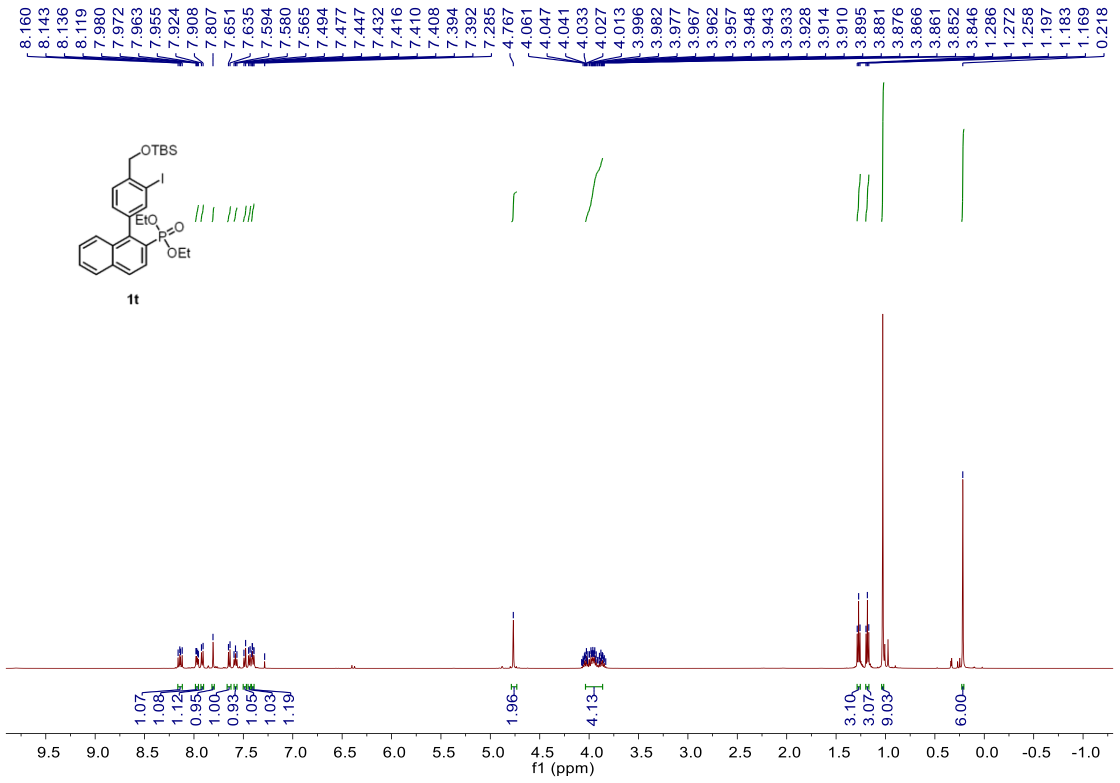
8.160 8.143 8.136 8.119 7.980 7.972 7.963 7.955 7.924 7.908 7.807 7.651 7.635 7.594 7.580 7.565 7.494 7.477 7.447 7.432 7.416 7.410 7.408 7.394 7.392 7.285 4.767 4.061 4.047 4.041 4.033 4.027 4.013 3.996 3.982 3.977 3.967 3.962 3.957 3.948 3.943 3.933 3.928 3.914 3.910 3.895 3.881 3.876 3.866 3.861 3.852 3.846 1.286 1.272 1.258 1.197 1.183 1.169 0.218
OTBS
I
EtO
P
O
OEt
1t
1.07 1.08 1.12 0.95 1.00 0.93 1.05 1.03 1.19 1.96 4.13 3.10 3.07 9.03 6.00
9.5 9.0 8.5 8.0 7.5 7.0 6.5 6.0 5.5 5.0 4.5 4.0 3.5 3.0 2.5 2.0 1.5 1.0 0.5 0.0 -0.5 -1.0
f1 (ppm)

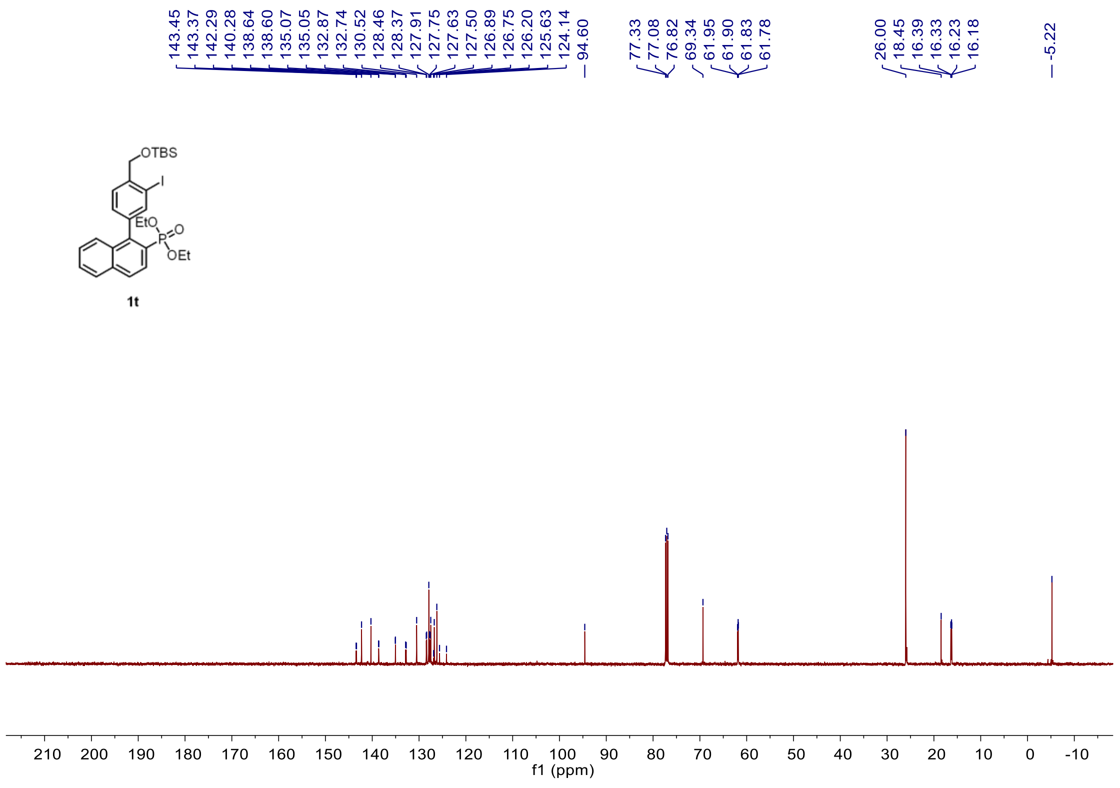
143.45 143.37 142.29 140.28 138.64 138.60 135.07 135.05 132.87 132.74 130.52 128.46 128.37 127.91 127.75 127.63 127.50 126.89 126.75 126.20 125.63 124.14 94.60 77.33 77.08 76.82 69.34 61.95 61.90 61.83 61.78 26.00 18.45 16.39 16.33 16.23 16.18 -5.22
OTBS
I
EtO
P
O
OEt
1t
210 200 190 180 170 160 150 140 130 120 110 100 90 80 70 60 50 40 30 20 10 0 -10
f1 (ppm)

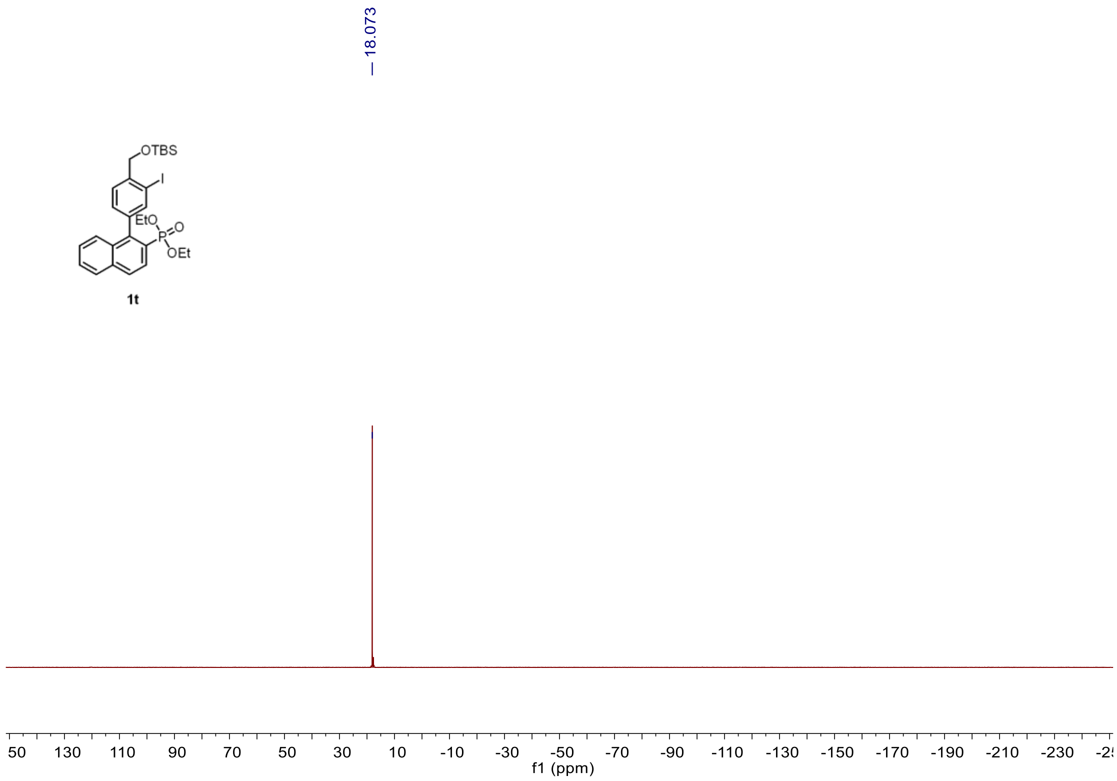
18.073
OTBS
I
EtO
P
O
OEt
1t
f1 (ppm)

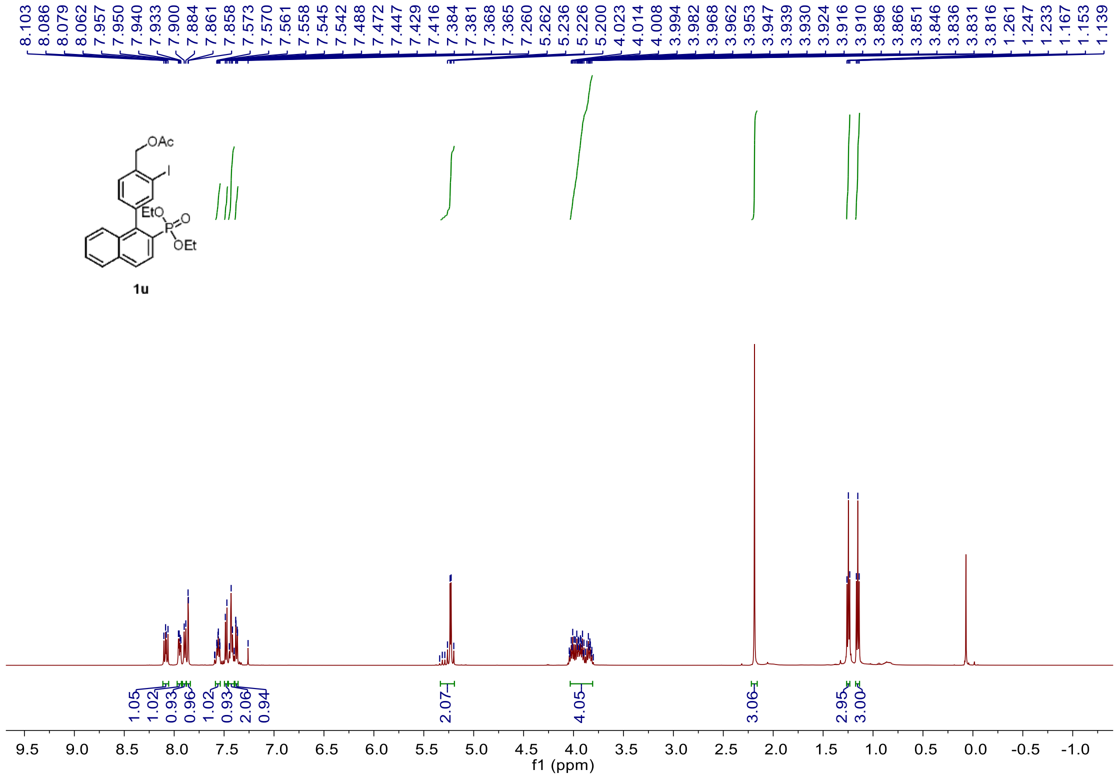
8.103 8.086 8.079 8.062 7.957 7.950 7.940 7.933 7.900 7.884 7.861 7.858 7.573 7.570 7.561 7.558 7.545 7.542 7.488 7.472 7.447 7.429 7.416 7.384 7.381 7.368 7.365 7.260 5.262 5.236 5.226 5.200 4.023 4.014 4.008 3.994 3.982 3.968 3.962 3.953 3.947 3.939 3.930 3.924 3.916 3.910 3.896 3.866 3.851 3.846 3.836 3.831 3.816 1.261 1.247 1.233 1.167 1.153 1.139
OAc
I
EtO
P
O
OEt
1u
1.05 1.02 0.93 0.96 1.02 0.93 2.06 0.94
2.07
4.05
3.06
2.95 3.00
f1 (ppm)

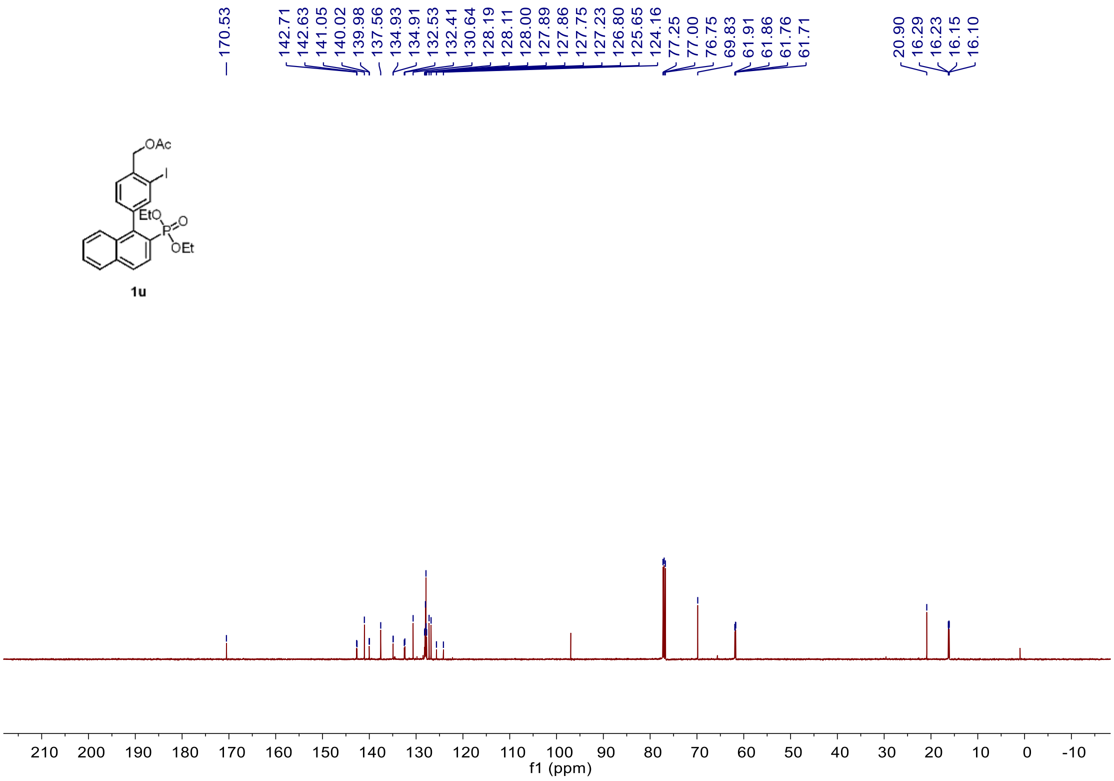

170.53
142.71
142.63
141.05
140.02
139.98
137.56
134.93
134.91
132.53
132.41
130.64
128.19
128.11
128.00
127.89
127.86
127.75
127.23
126.80
125.65
124.16
77.25
77.00
76.75
69.83
61.91
61.86
61.76
61.71
20.90
16.29
16.23
16.15
16.10
OAc
I
EtO
P
O
OEt
1u
210 200 190 180 170 160 150 140 130 120 110 100 90 80 70 60 50 40 30 20 10 0 -10
f1 (ppm)

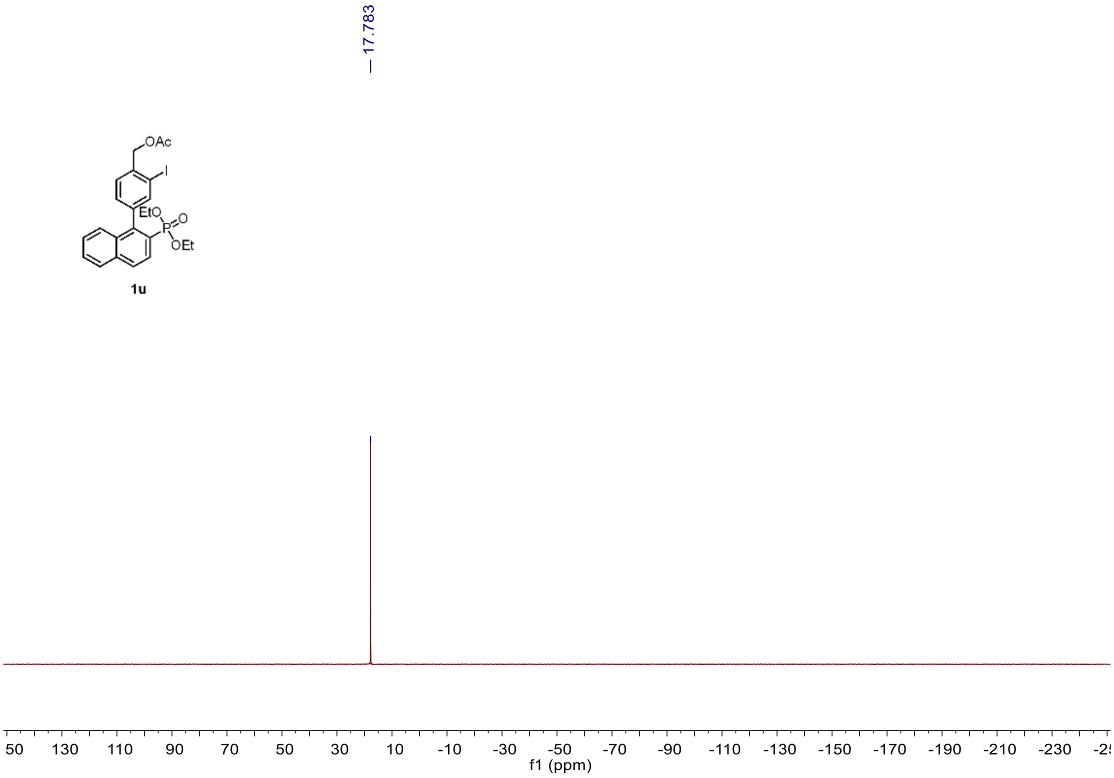

17.783
OAc
I
EtO
P
O
OEt
1u
50 130 110 90 70 50 30 10 -10 -30 -50 -70 -90 -110 -130 -150 -170 -190 -210 -230
f1 (ppm)

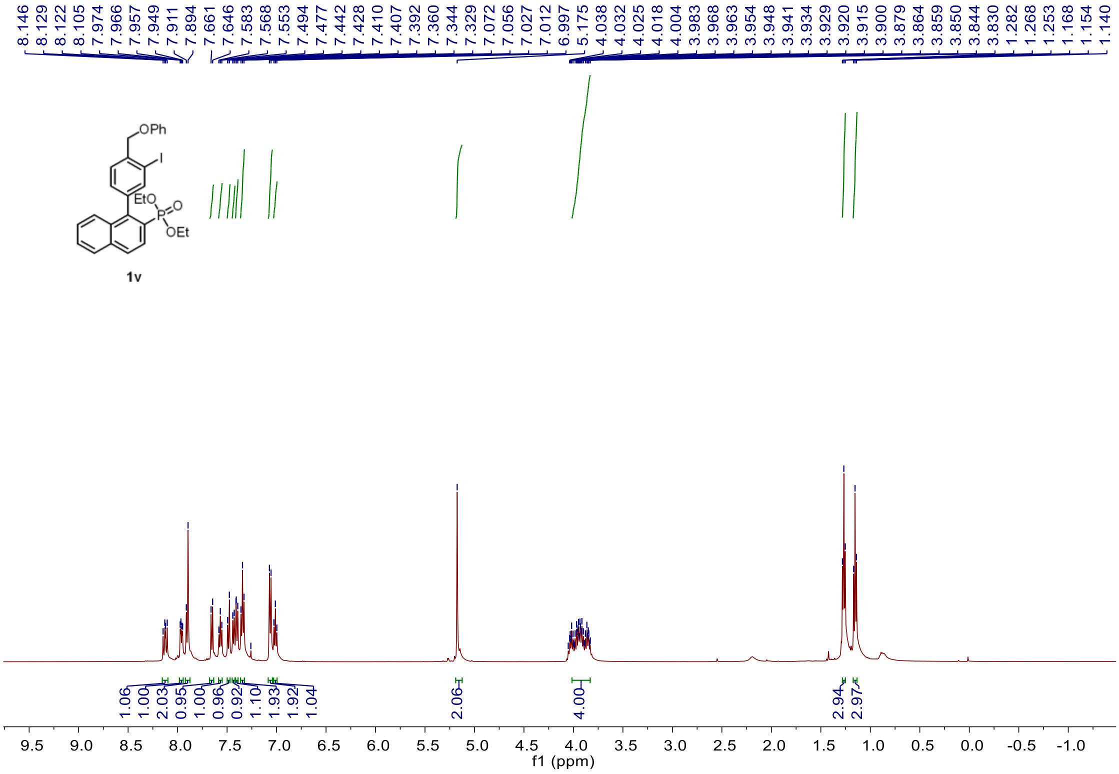
8.146
8.129
8.122
8.105
7.974
7.966
7.957
7.949
7.911
7.894
7.661
7.646
7.583
7.568
7.553
7.494
7.477
7.442
7.428
7.410
7.407
7.392
7.360
7.344
7.329
7.072
7.056
7.027
7.012
6.997
5.175
4.038
4.032
4.025
4.018
4.004
3.983
3.968
3.963
3.954
3.948
3.941
3.934
3.929
3.920
3.915
3.900
3.879
3.864
3.859
3.850
3.844
3.830
1.282
1.268
1.253
1.168
1.154
1.140
OPh
I
EtO
P
O
OEt
1v
1.06
1.00
2.03
0.95
1.00
0.96
0.92
1.10
1.93
1.92
1.04
2.06
4.00
2.94
2.97
9.5 9.0 8.5 8.0 7.5 7.0 6.5 6.0 5.5 5.0 4.5 4.0 3.5 3.0 2.5 2.0 1.5 1.0 0.5 0.0 -0.5 -1.0
f1 (ppm)

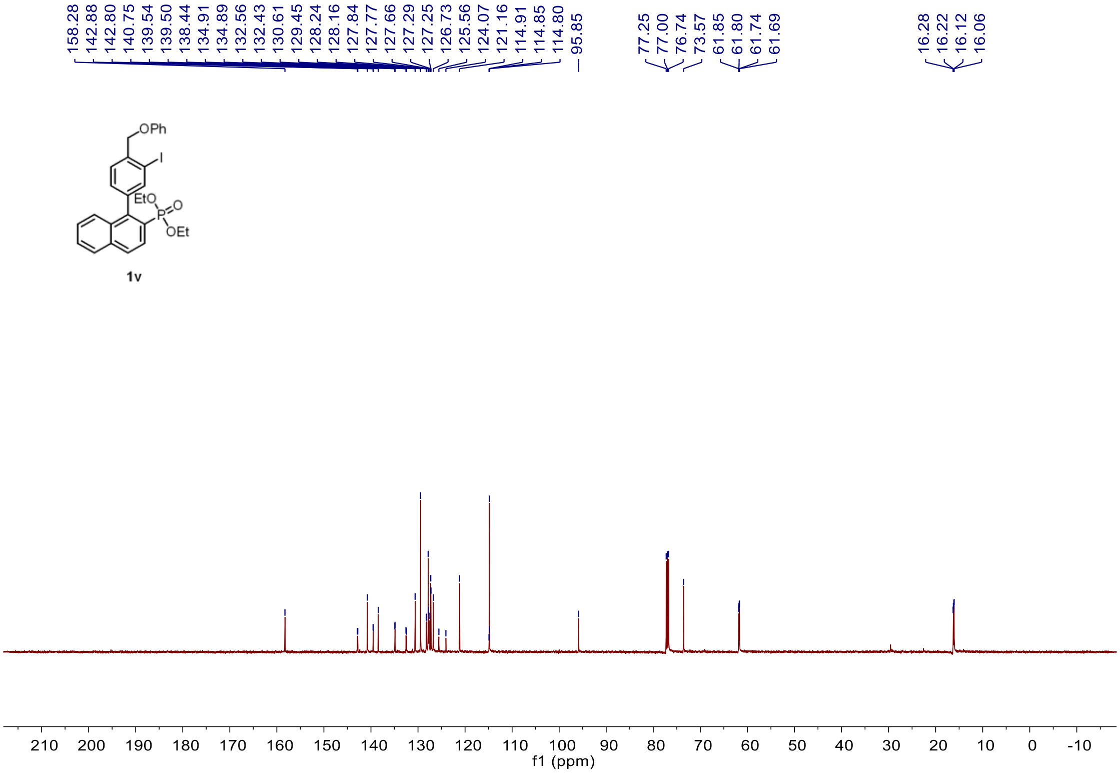
158.28
142.88
142.80
140.75
139.54
139.50
138.44
134.91
134.89
132.56
132.43
130.61
129.45
128.24
128.16
127.84
127.77
127.66
127.29
127.25
126.73
125.56
124.07
121.16
114.91
114.85
114.80
95.85
77.25
77.00
76.74
73.57
61.85
61.80
61.74
61.69
16.28
16.22
16.12
16.06
OPh
I
EtO
P
O
OEt
1v
210 200 190 180 170 160 150 140 130 120 110 100 90 80 70 60 50 40 30 20 10 0 -10
f1 (ppm)

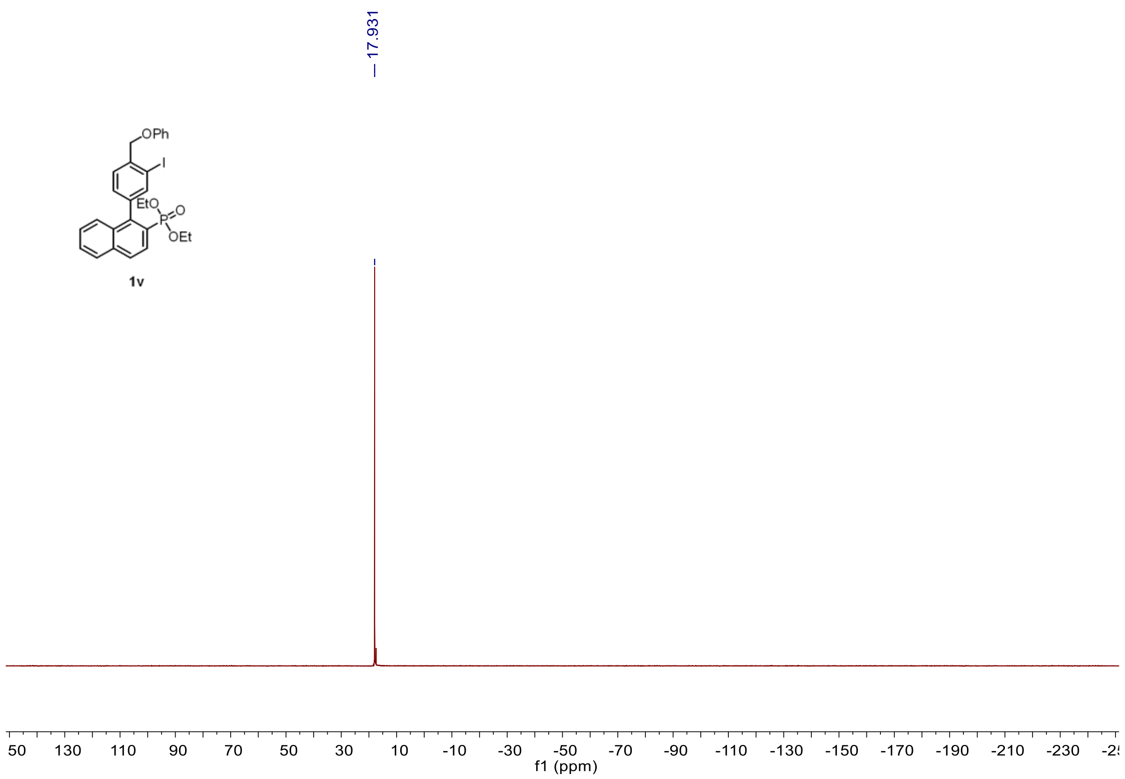

— 17.931
OPh
I
EtO
P
O
OEt
1v
f1 (ppm)

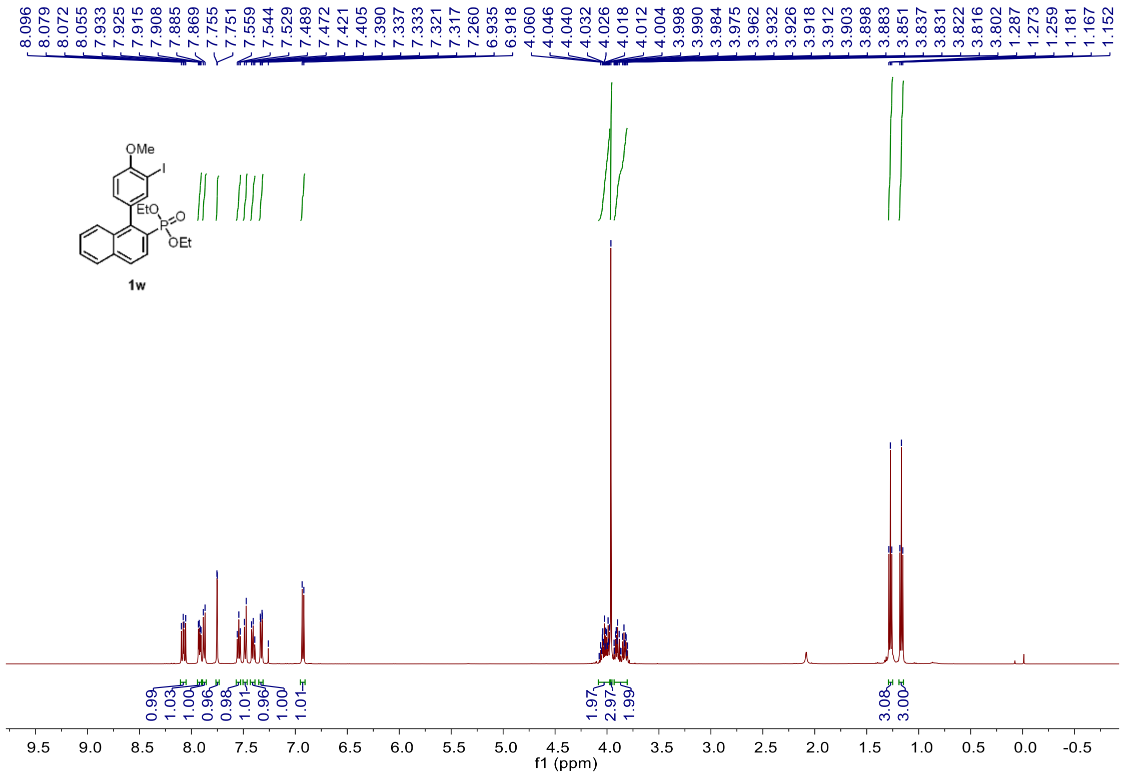

OMe
I
EtO
P
O
OEt
1w
f1 (ppm)

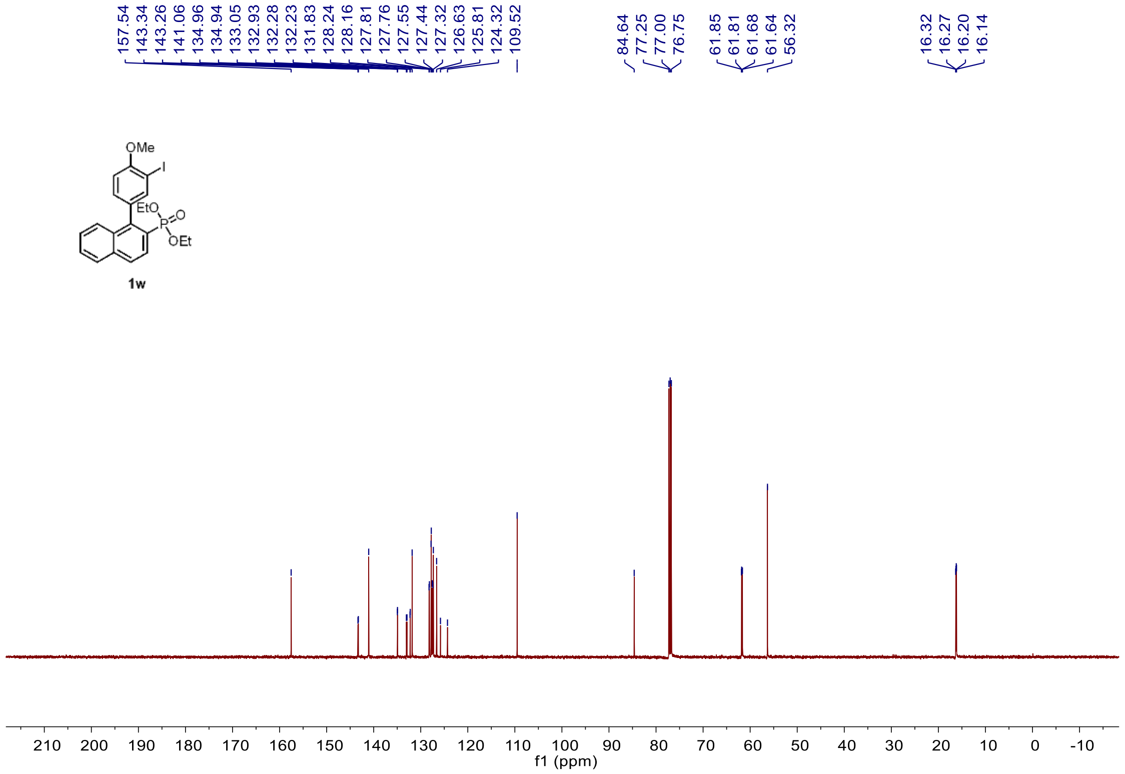
157.54
143.34
143.26
141.06
134.96
134.94
133.05
132.93
132.28
132.23
131.83
128.24
128.16
127.81
127.76
127.55
127.44
127.32
126.63
125.81
124.32
109.52
84.64
77.25
77.00
76.75
61.85
61.81
61.68
61.64
56.32
16.32
16.27
16.20
16.14
OMe
I
EtO
P
O
OEt
1w
210 200 190 180 170 160 150 140 130 120 110 100 90 80 70 60 50 40 30 20 10 0 -10
f1 (ppm)

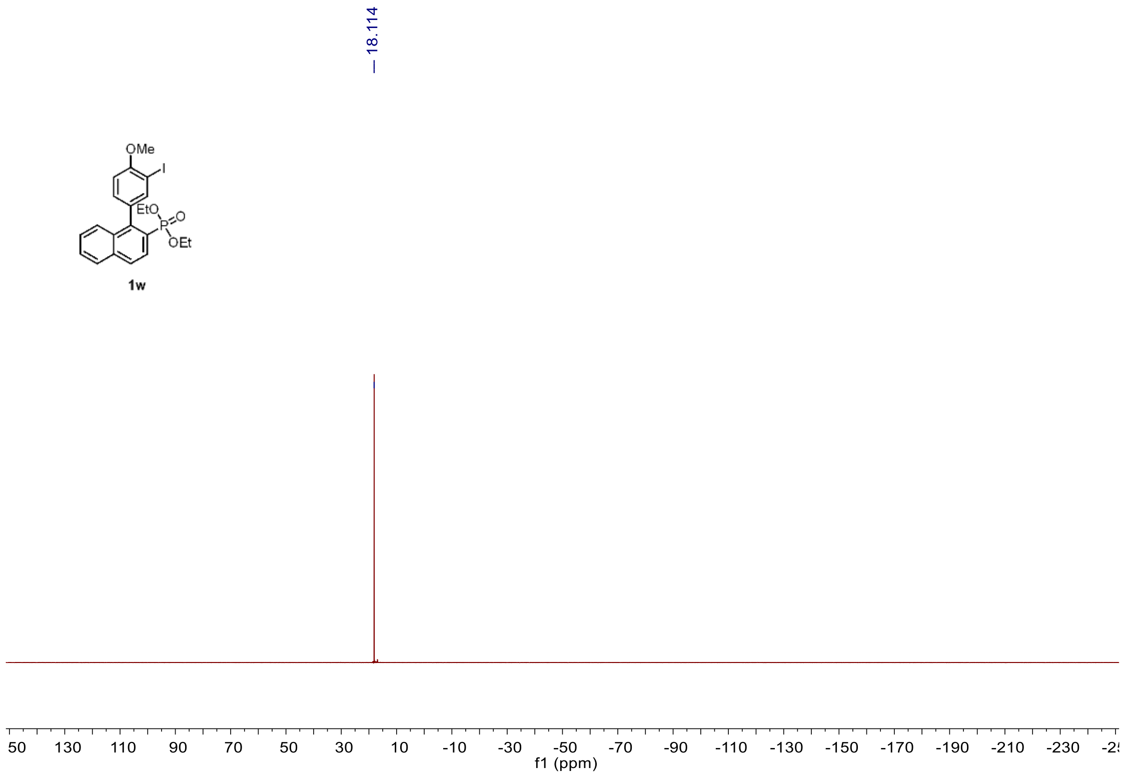
18.114
OMe
I
EtO
P
O
OEt
1w
50 130 110 90 70 50 30 10 -10 -30 -50 -70 -90 -110 -130 -150 -170 -190 -210 -230
f1 (ppm)

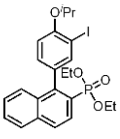

O^{i}Pr
I
EtO
P
O
OEt

1x

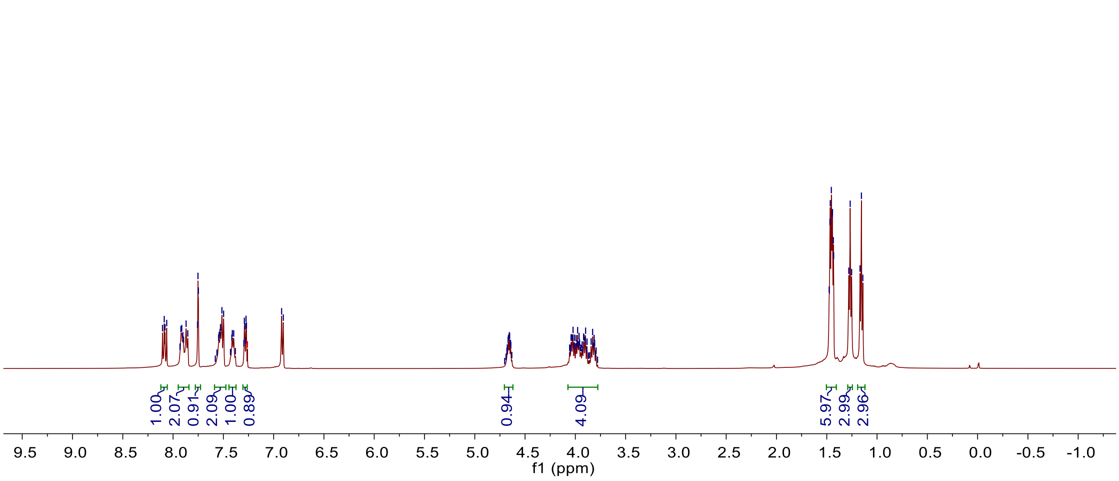

8.105 8.088 8.080 8.063 7.922 7.914 7.903 7.896 7.870 7.854 7.757 7.753 7.748 7.547 7.542 7.534 7.528 7.514 7.498 7.416 7.411 7.397 7.291 7.287 7.275 7.270 6.921 6.904 4.669 4.665 4.657 4.653 4.042 4.037 4.028 4.023 4.008 3.992 3.977 3.963 3.918 3.912 3.898 3.828 3.813 1.476 1.469 1.465 1.455 1.447 1.443 1.434 1.431 1.281 1.267 1.253 1.170 1.155 1.141
1.00 2.07 0.91 2.09 1.00 0.89 0.94 4.09 5.97 2.99 2.96
9.5 9.0 8.5 8.0 7.5 7.0 6.5 6.0 5.5 5.0 4.5 4.0 3.5 3.0 2.5 2.0 1.5 1.0 0.5 0.0 -0.5 -1.0
f1 (ppm)

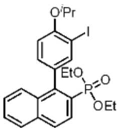

O^{i}Pr
I
EtO
P
O
OEt

1x

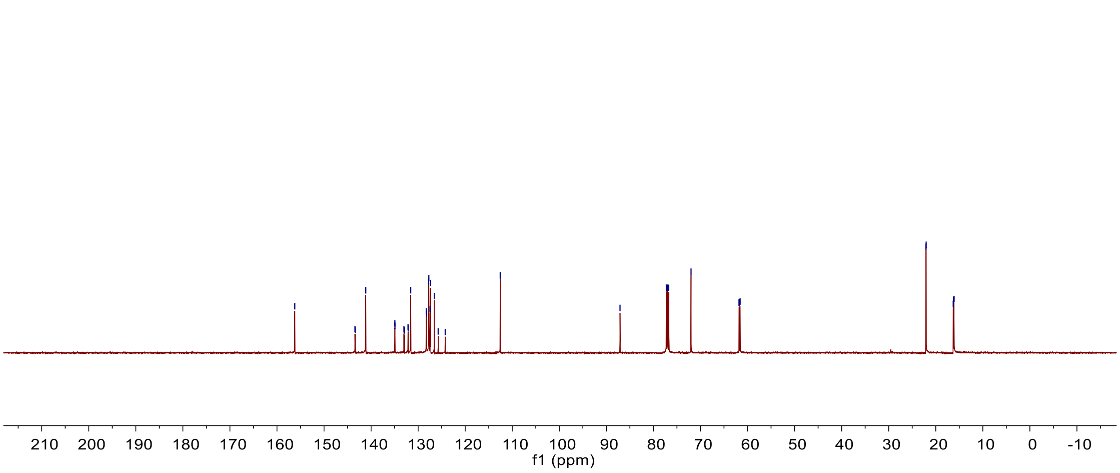

156.21 143.43 143.35 141.13 134.95 134.93 133.02 132.89 132.15 132.10 131.58 128.28 128.20 127.77 127.73 127.47 127.37 126.56 125.74 124.25 112.56 87.10 77.26 77.00 76.75 72.00 61.80 61.76 61.63 61.58 22.09 22.03 16.29 16.23 16.15 16.10
210 200 190 180 170 160 150 140 130 120 110 100 90 80 70 60 50 40 30 20 10 0 -10
f1 (ppm)

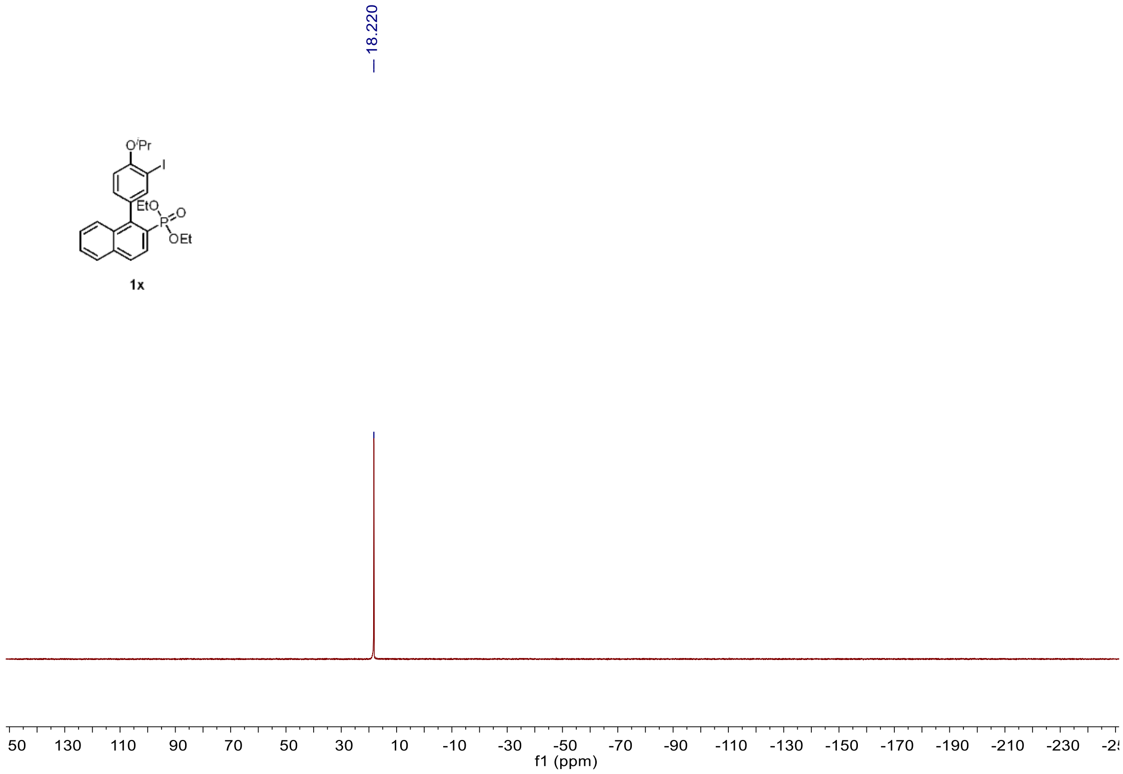

18.220
O^{i}Pr
I
EtO
P=O
OEt
1x
50 130 110 90 70 50 30 10 -10 -30 -50 -70 -90 -110 -130 -150 -170 -190 -210 -230
f1 (ppm)

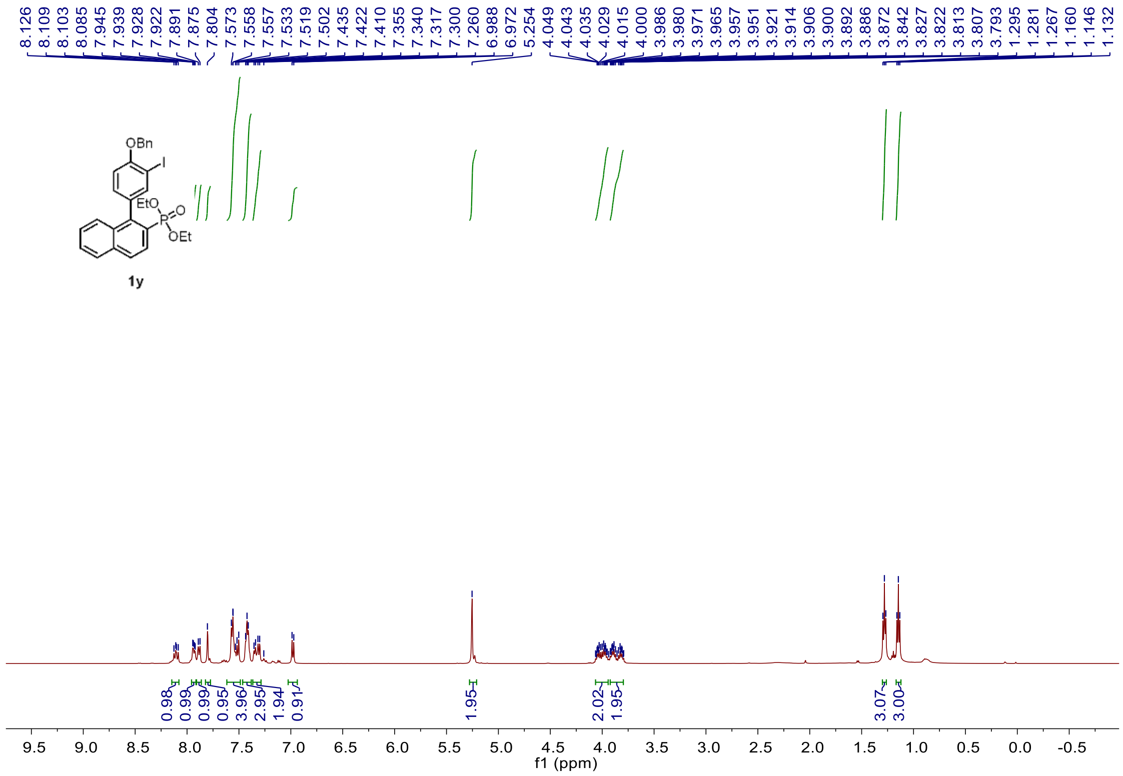

8.126 8.109 8.103 8.085 7.945 7.939 7.928 7.922 7.891 7.875 7.804 7.573 7.558 7.557 7.533 7.519 7.502 7.435 7.422 7.410 7.355 7.340 7.317 7.300 7.260 6.988 6.972 5.254 4.049 4.043 4.035 4.029 4.015 4.000 3.986 3.980 3.971 3.965 3.957 3.951 3.921 3.914 3.906 3.900 3.892 3.886 3.872 3.842 3.827 3.822 3.813 3.807 3.793 1.295 1.281 1.267 1.160 1.146 1.132
OBn
I
EtO
P=O
OEt
1y
0.98 0.99 0.99 0.95 3.96 2.95 1.94 0.91 1.95 2.02 1.95 3.07 3.00
9.5 9.0 8.5 8.0 7.5 7.0 6.5 6.0 5.5 5.0 4.5 4.0 3.5 3.0 2.5 2.0 1.5 1.0 0.5 0.0 -0.5
f1 (ppm)

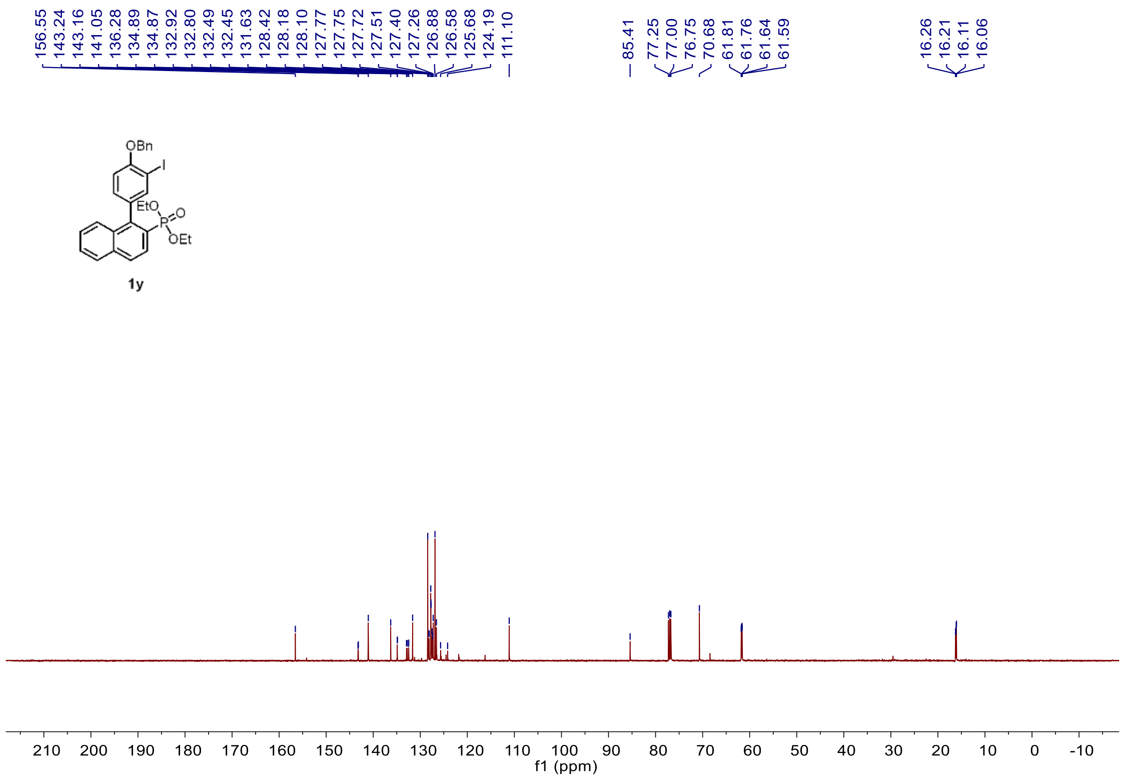
156.55
143.24
143.16
141.05
136.28
134.89
134.87
132.92
132.80
132.49
132.45
131.63
128.42
128.18
128.10
127.77
127.75
127.72
127.51
127.40
127.26
126.88
126.58
125.68
124.19
111.10
85.41
77.25
77.00
76.75
70.68
61.81
61.76
61.64
61.59
16.26
16.21
16.11
16.06
OBn
I
EtO
P
O
OEt
1y
f1 (ppm)

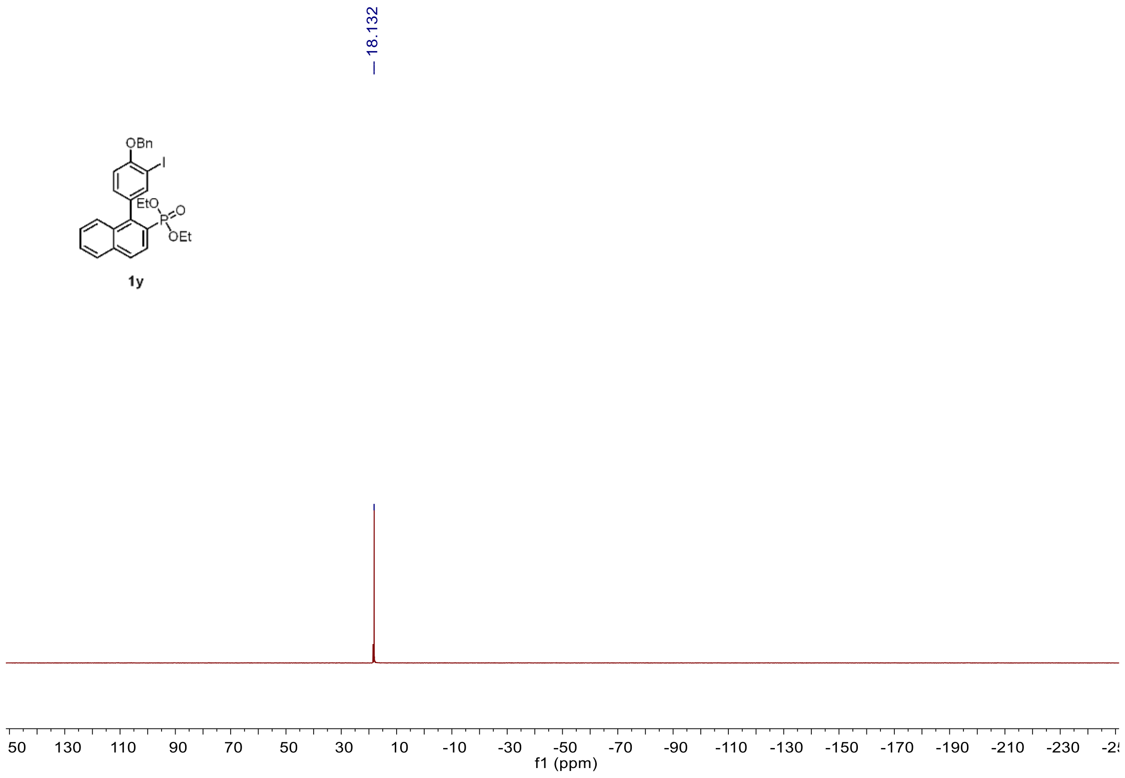
18.132
OBn
I
EtO
P
O
OEt
1y
f1 (ppm)

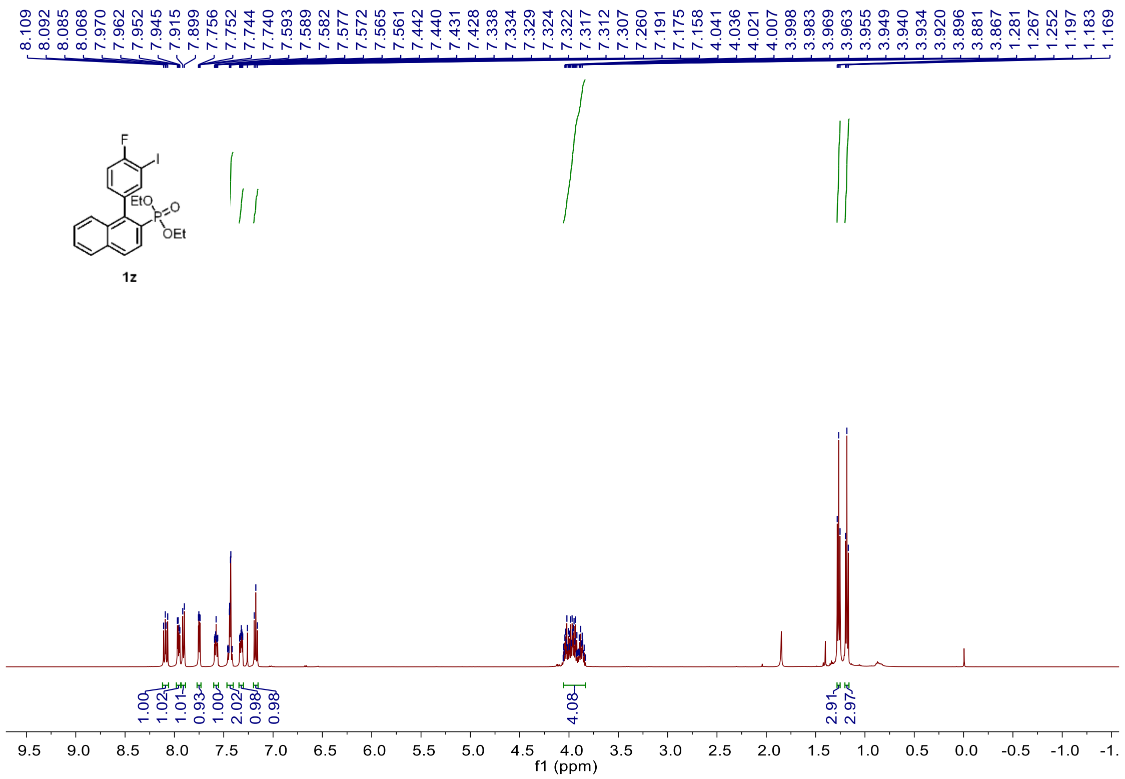
8.109
8.092
8.085
8.068
7.970
7.962
7.952
7.945
7.915
7.899
7.756
7.752
7.744
7.740
7.593
7.589
7.582
7.577
7.572
7.565
7.561
7.442
7.440
7.431
7.428
7.338
7.334
7.329
7.324
7.322
7.317
7.312
7.307
7.260
7.191
7.175
7.158
4.041
4.036
4.021
4.007
3.998
3.983
3.969
3.963
3.955
3.949
3.940
3.934
3.920
3.896
3.881
3.867
1.281
1.267
1.252
1.197
1.183
1.169
F
I
EtO
P
O
OEt
1z
1.00
1.02
1.01
0.93
1.00
2.02
0.98
0.98
4.08
2.91
2.97
f1 (ppm)

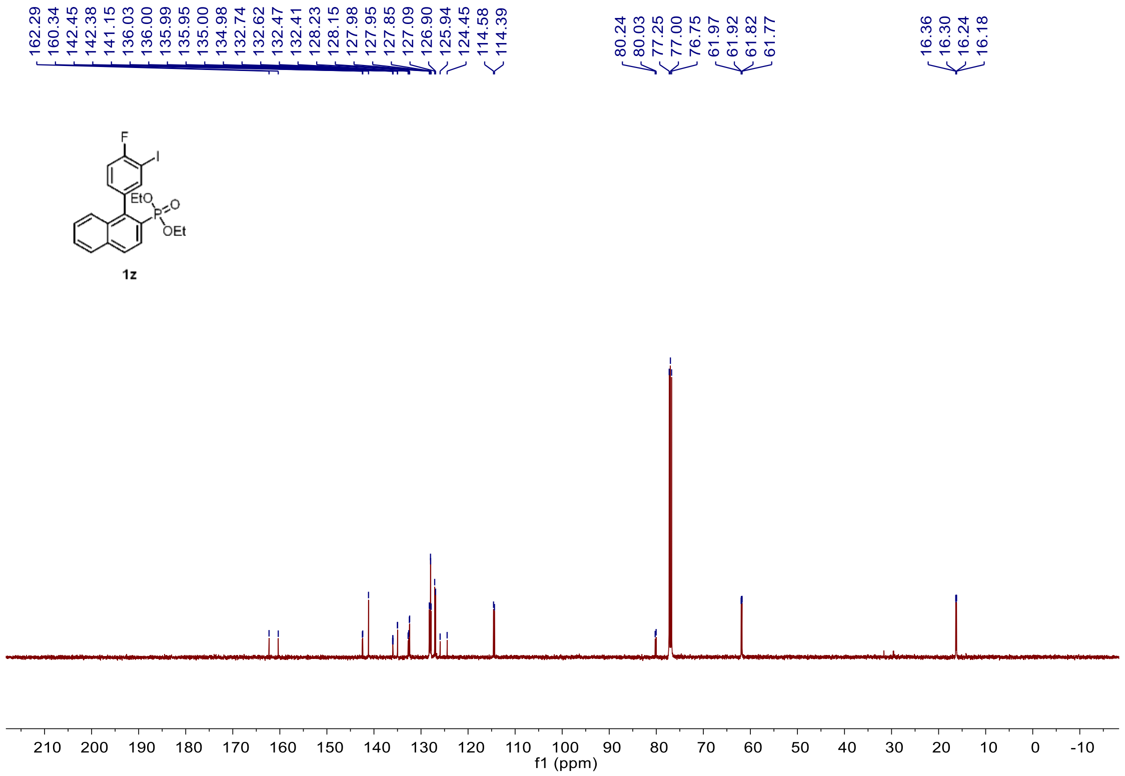
162.29
160.34
142.45
142.38
141.15
136.03
136.00
135.99
135.95
135.00
134.98
132.74
132.62
132.47
132.41
128.23
128.15
127.98
127.95
127.85
127.09
126.90
125.94
124.45
114.58
114.39
80.24
80.03
77.25
77.00
76.75
61.97
61.92
61.82
61.77
16.36
16.30
16.24
16.18
F
I
EtO
P
O
OEt
1z
f1 (ppm)

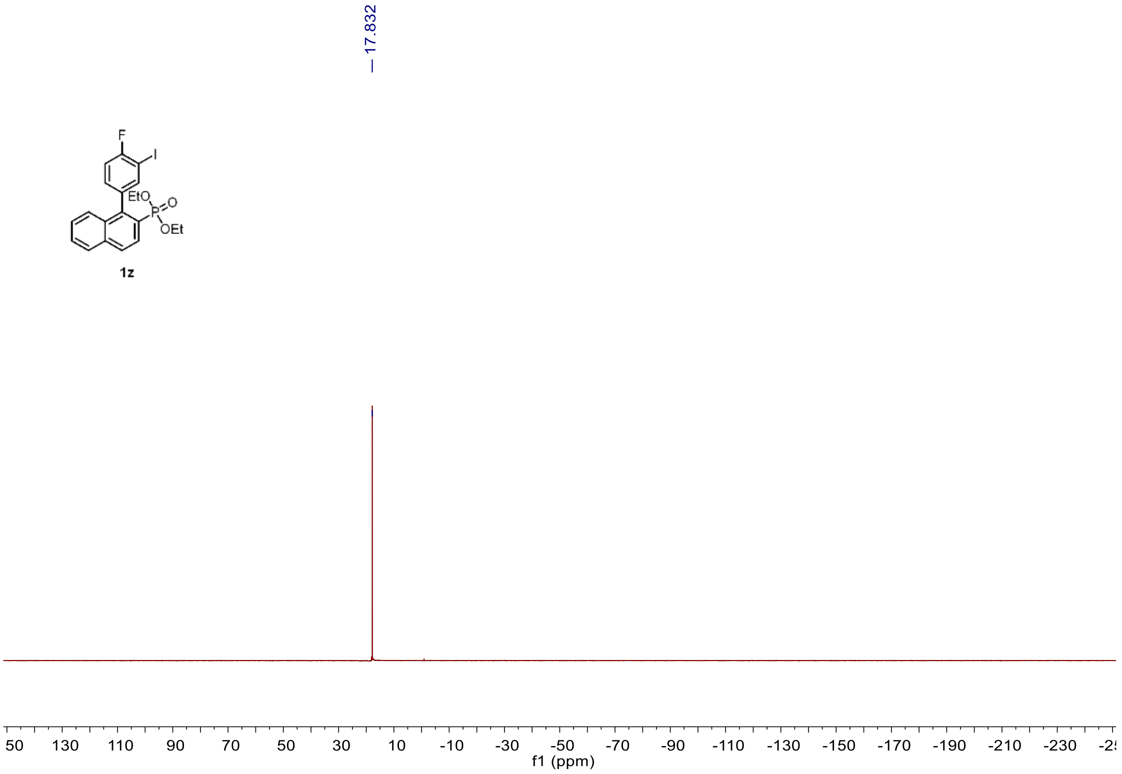
— 17.832
F
I
EtO
P
O
OEt
1z
50 130 110 90 70 50 30 10 -10 -30 -50 -70 -90 -110 -130 -150 -170 -190 -210 -230
f1 (ppm)

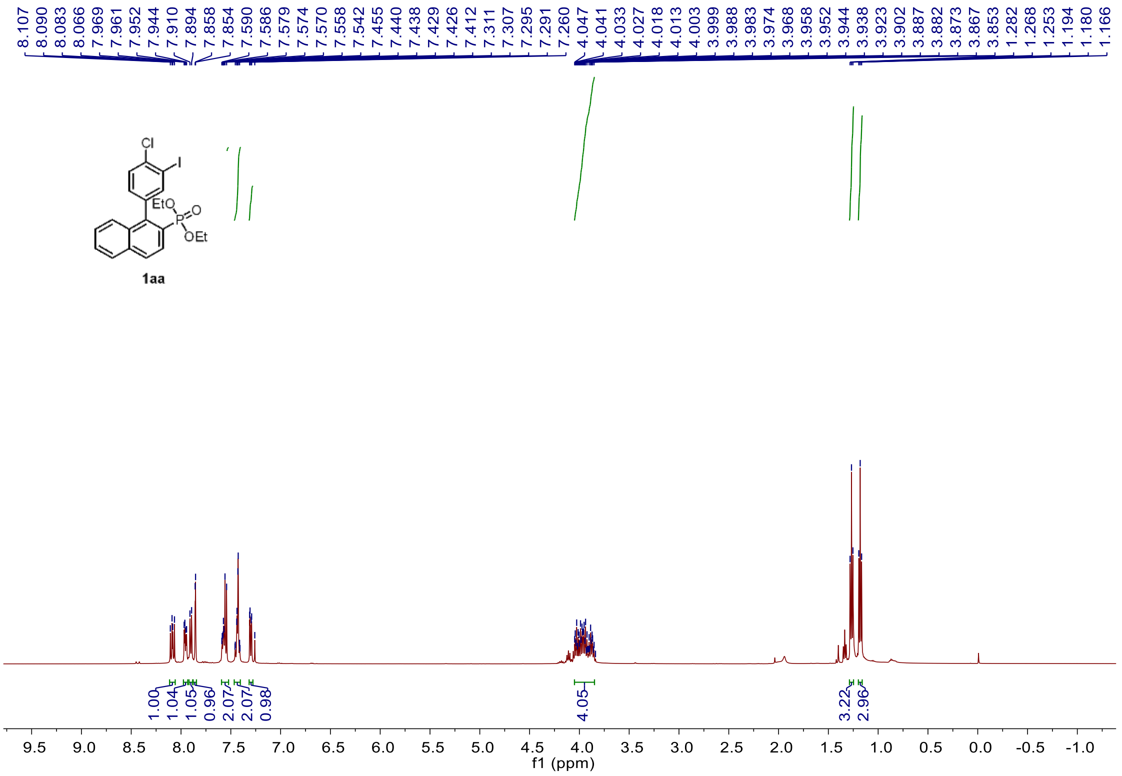
8.107 8.090 8.083 8.066 7.969 7.961 7.952 7.944 7.910 7.894 7.858 7.854 7.590 7.586 7.579 7.574 7.570 7.558 7.542 7.455 7.440 7.438 7.429 7.426 7.412 7.311 7.307 7.295 7.291 7.260 4.047 4.041 4.033 4.027 4.018 4.013 4.003 3.999 3.988 3.983 3.974 3.968 3.958 3.952 3.944 3.938 3.923 3.902 3.887 3.882 3.873 3.867 3.853 1.282 1.268 1.253 1.194 1.180 1.166
Cl
I
EtO
P
O
OEt
1aa
1.00 1.04 1.05 0.96 2.07 2.07 0.98
4.05
3.22 2.96
9.5 9.0 8.5 8.0 7.5 7.0 6.5 6.0 5.5 5.0 4.5 4.0 3.5 3.0 2.5 2.0 1.5 1.0 0.5 0.0 -0.5 -1.0
f1 (ppm)

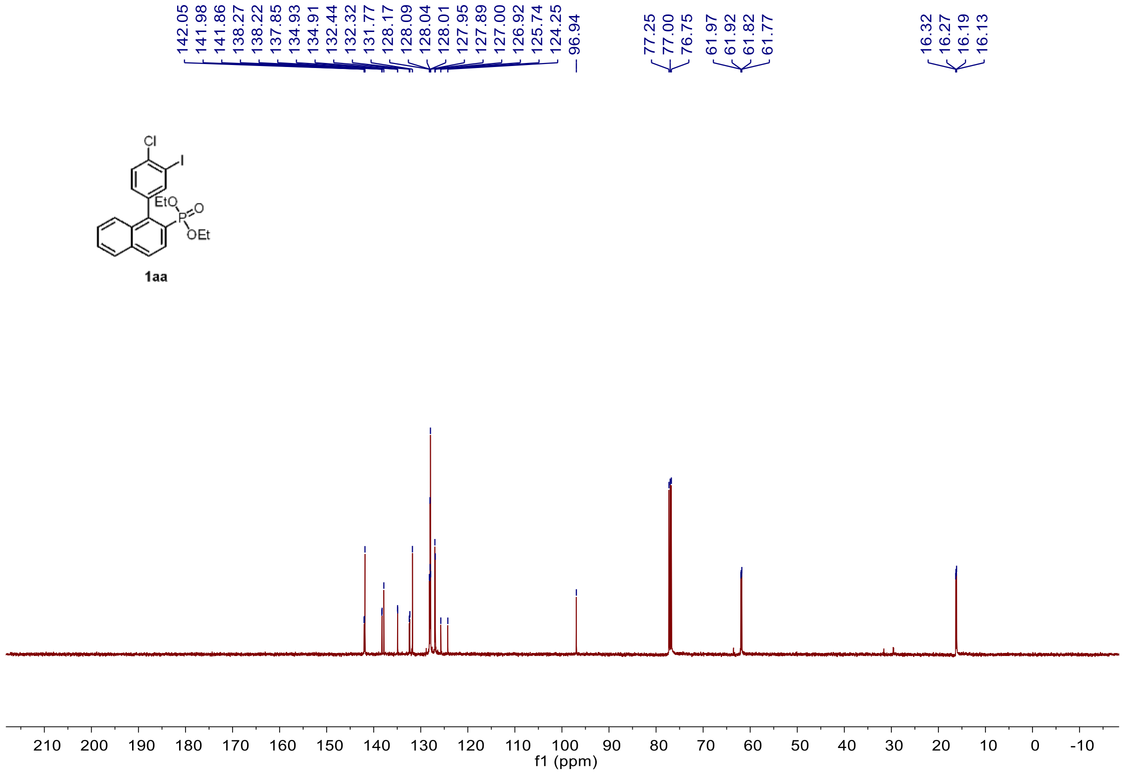
142.05
141.98
141.86
138.27
138.22
137.85
134.93
134.91
132.44
132.32
131.77
128.17
128.09
128.04
128.01
127.95
127.89
127.00
126.92
125.74
124.25
96.94
77.25
77.00
76.75
61.97
61.92
61.82
61.77
16.32
16.27
16.19
16.13
Cl
I
EtO
P
O
OEt
1aa
f1 (ppm)

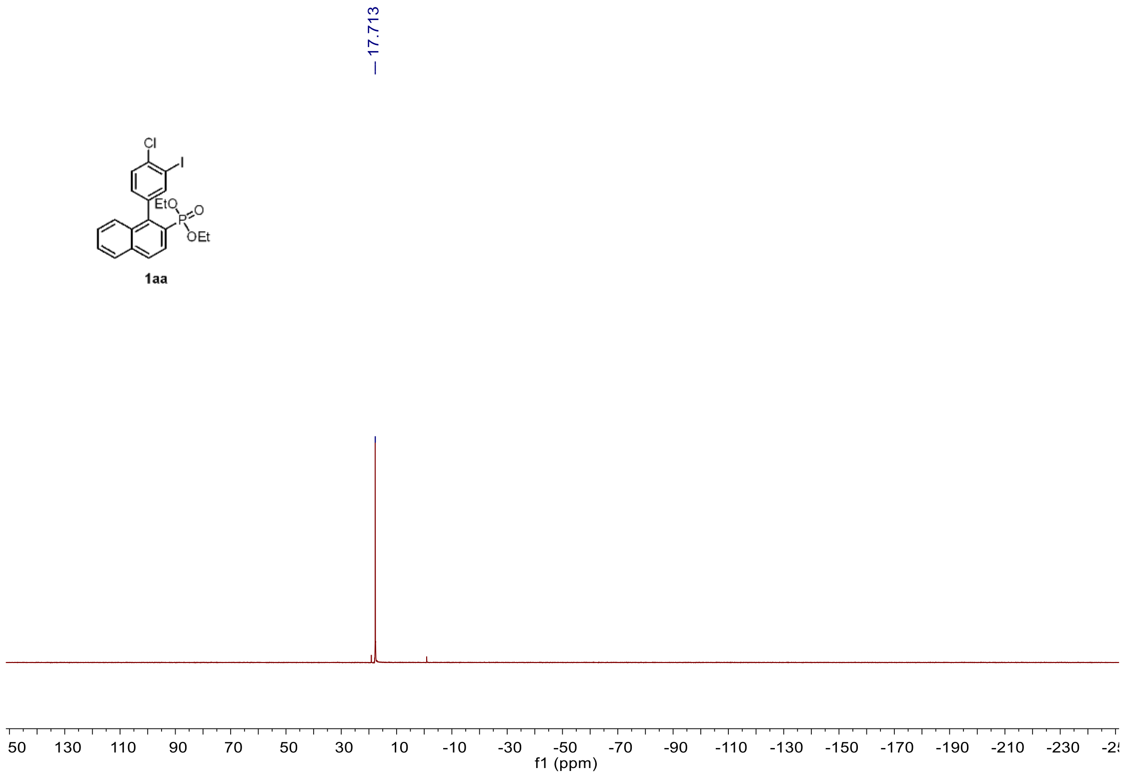
17.713
Cl
I
EtO
P
O
OEt
1aa
f1 (ppm)

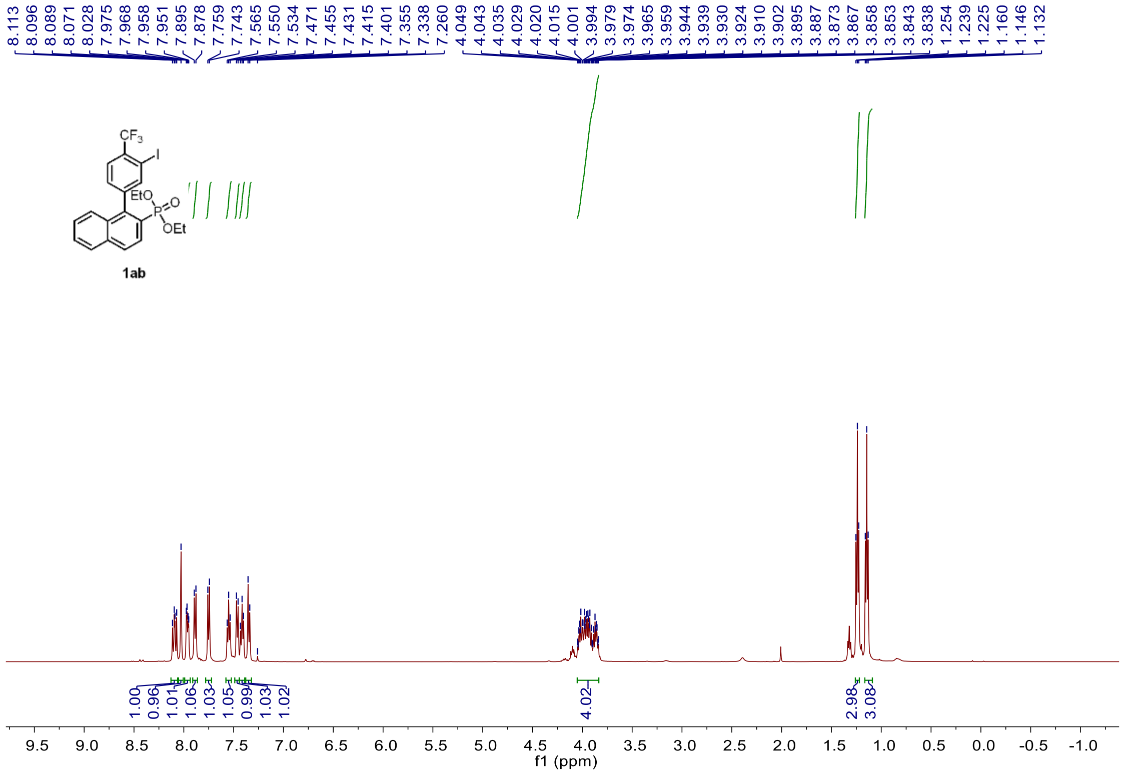
8.113
8.096
8.089
8.071
8.028
7.975
7.968
7.958
7.951
7.895
7.878
7.759
7.743
7.565
7.550
7.534
7.471
7.455
7.431
7.415
7.401
7.355
7.338
7.260
4.049
4.043
4.035
4.029
4.020
4.015
4.001
3.994
3.979
3.974
3.965
3.959
3.944
3.939
3.930
3.924
3.910
3.902
3.895
3.887
3.873
3.867
3.858
3.853
3.843
3.838
1.254
1.239
1.225
1.160
1.146
1.132
CF3
I
EtO
P
O
OEt
1ab
1.00
0.96
1.01
1.06
1.03
1.05
0.99
1.03
1.02
4.02
2.98
3.08
9.5 9.0 8.5 8.0 7.5 7.0 6.5 6.0 5.5 5.0 4.5 4.0 3.5 3.0 2.5 2.0 1.5 1.0 0.5 0.0 -0.5 -1.0
f1 (ppm)

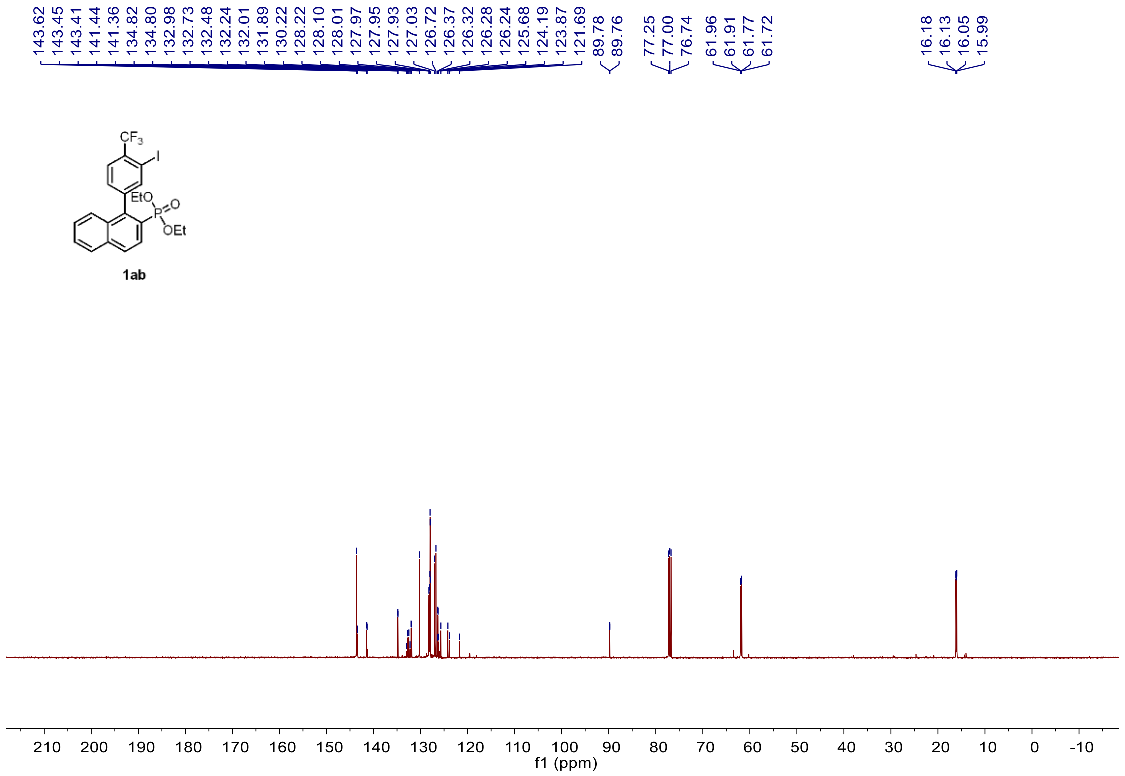
143.62
143.45
143.41
141.44
141.36
134.82
134.80
132.98
132.73
132.48
132.24
132.01
131.89
130.22
128.22
128.10
128.01
127.97
127.95
127.93
127.03
126.72
126.37
126.32
126.28
126.24
125.68
124.19
123.87
121.69
89.78
89.76
77.25
77.00
76.74
61.96
61.91
61.77
61.72
16.18
16.13
16.05
15.99
CF3
I
EtO
P
O
OEt
1ab
210 200 190 180 170 160 150 140 130 120 110 100 90 80 70 60 50 40 30 20 10 0 -10
f1 (ppm)

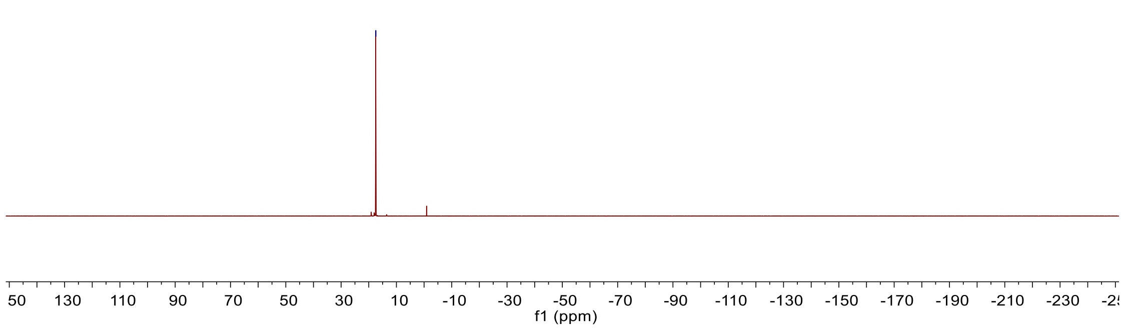
17.462
50 130 110 90 70 50 30 10 -10 -30 -50 -70 -90 -110 -130 -150 -170 -190 -210 -230
f1 (ppm)

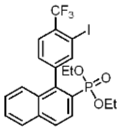
CF3
I
EtO
P
O
OEt

1ab

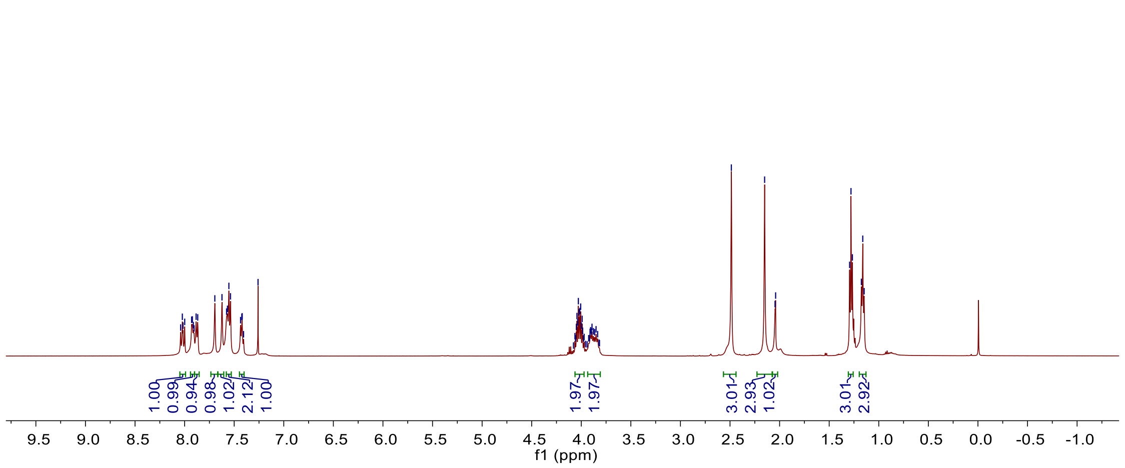
8.040
8.023
8.016
7.999
7.929
7.922
7.912
7.905
7.884
7.868
7.695
7.623
7.577
7.568
7.554
7.537
7.436
7.423
7.419
7.405
7.260
4.064
4.058
4.050
4.044
4.035
4.029
4.020
4.015
4.006
4.000
3.991
3.986
3.977
3.971
3.957
3.941
3.925
3.918
3.912
3.906
3.897
3.892
3.878
3.865
3.851
3.835
3.821
3.816
2.487
2.151
2.046
2.040
1.294
1.280
1.266
1.175
1.161
1.147
1.00
0.99
0.94
0.98
1.02
2.12
1.00
1.97
1.97
3.01
2.93
1.02
3.01
2.92
9.5 9.0 8.5 8.0 7.5 7.0 6.5 6.0 5.5 5.0 4.5 4.0 3.5 3.0 2.5 2.0 1.5 1.0 0.5 0.0 -0.5 -1.0
f1 (ppm)

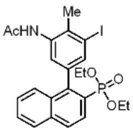
Me
AcHN
I
EtO
P
O
OEt

1ac

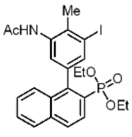
Me
AcHN
I
EtO
P
O
OEt

1ac

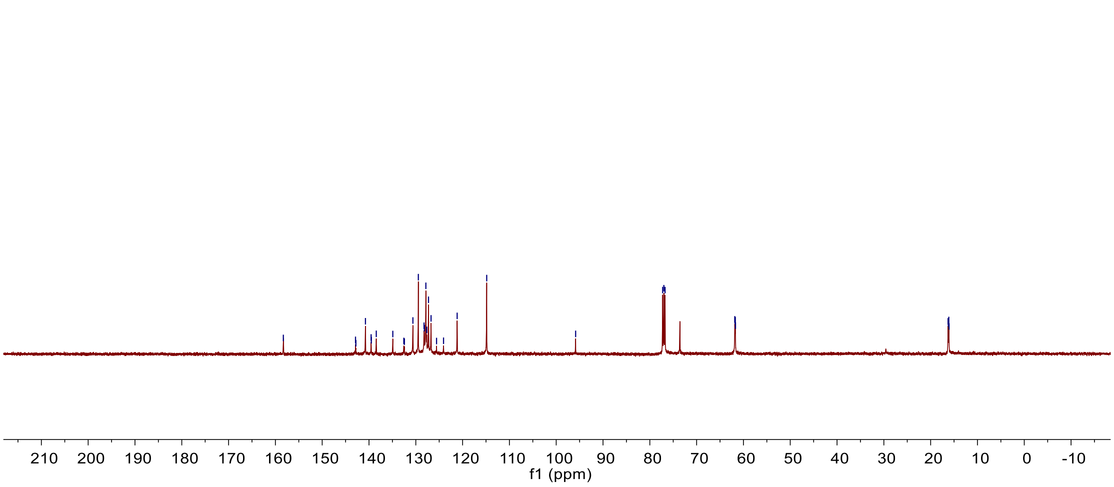
158.30
142.88
142.80
140.76
139.55
139.50
138.44
134.91
132.56
132.44
130.62
129.46
128.24
128.16
127.85
127.66
127.29
126.74
125.57
124.07
121.16
114.85
95.85
77.25
77.00
76.75
61.85
61.80
61.74
61.69
16.28
16.23
16.12
16.07
210 200 190 180 170 160 150 140 130 120 110 100 90 80 70 60 50 40 30 20 10 0 -10
f1 (ppm)

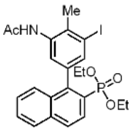
Me
AcHN
I
EtO
P
O
OEt

1ac

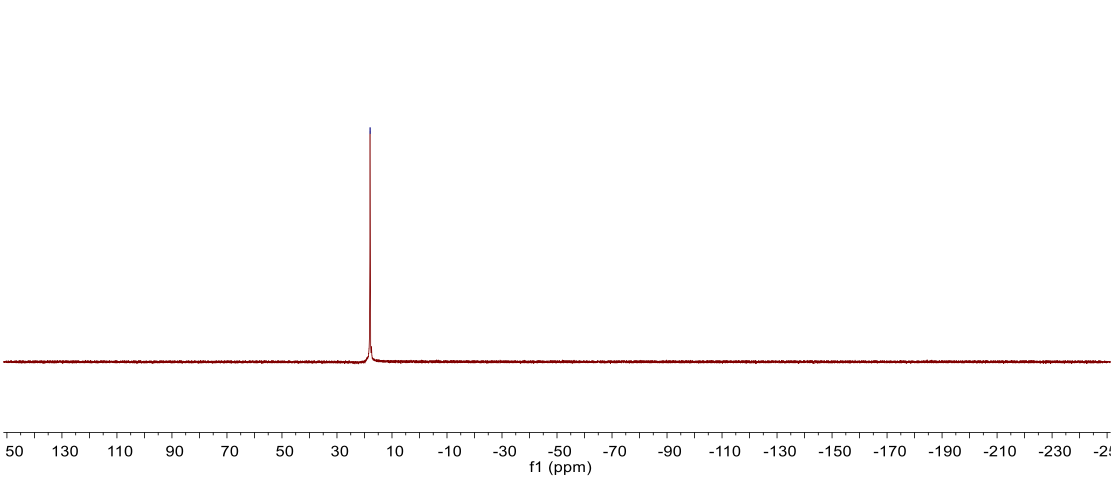
17.948
130 110 90 70 50 30 10 -10 -30 -50 -70 -90 -110 -130 -150 -170 -190 -210 -230
f1 (ppm)

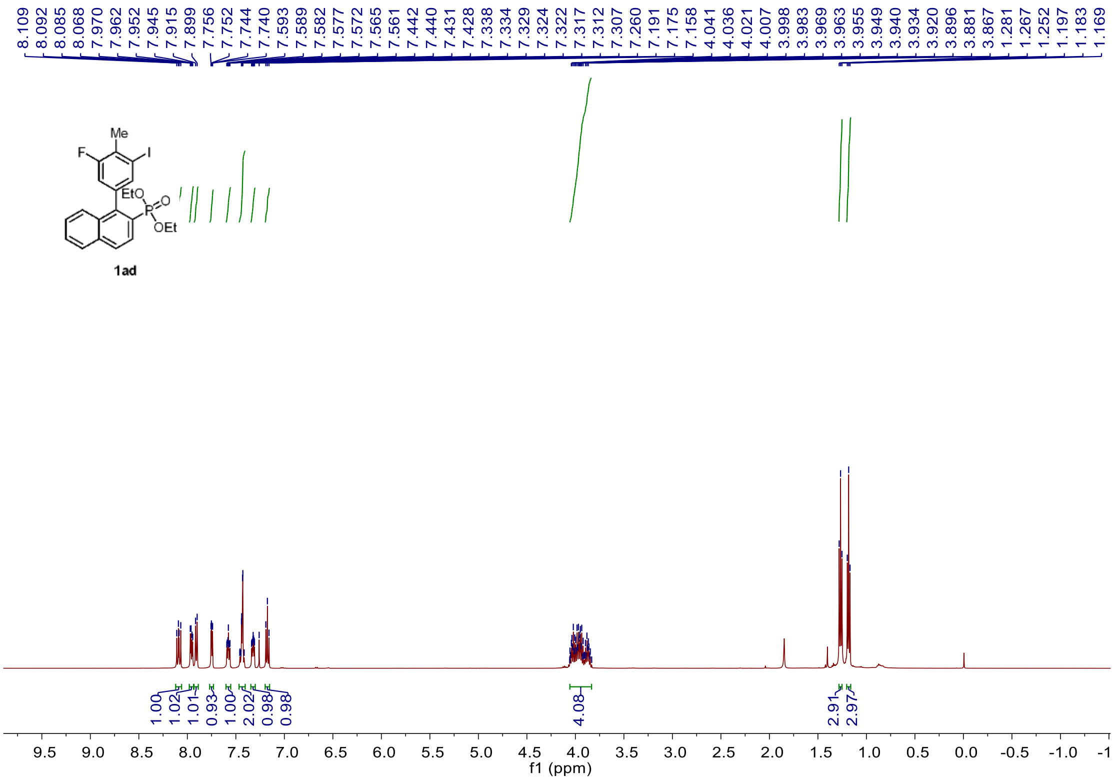
8.109 8.092 8.085 8.068 7.970 7.962 7.952 7.945 7.915 7.899 7.756 7.752 7.744 7.740 7.593 7.589 7.582 7.577 7.572 7.565 7.561 7.442 7.440 7.431 7.428 7.338 7.334 7.329 7.324 7.322 7.317 7.312 7.307 7.260 7.191 7.175 7.158 4.041 4.036 4.021 4.007 3.998 3.983 3.969 3.963 3.955 3.949 3.940 3.934 3.920 3.896 3.881 3.867 1.281 1.267 1.252 1.197 1.183 1.169
Me
F
I
EtO
P
O
OEt
1ad
1.00 1.02 1.01 0.93 1.00 2.02 0.98 0.98
4.08
2.91 2.97
9.5 9.0 8.5 8.0 7.5 7.0 6.5 6.0 5.5 5.0 4.5 4.0 3.5 3.0 2.5 2.0 1.5 1.0 0.5 0.0 -0.5 -1.0 -1
f1 (ppm)

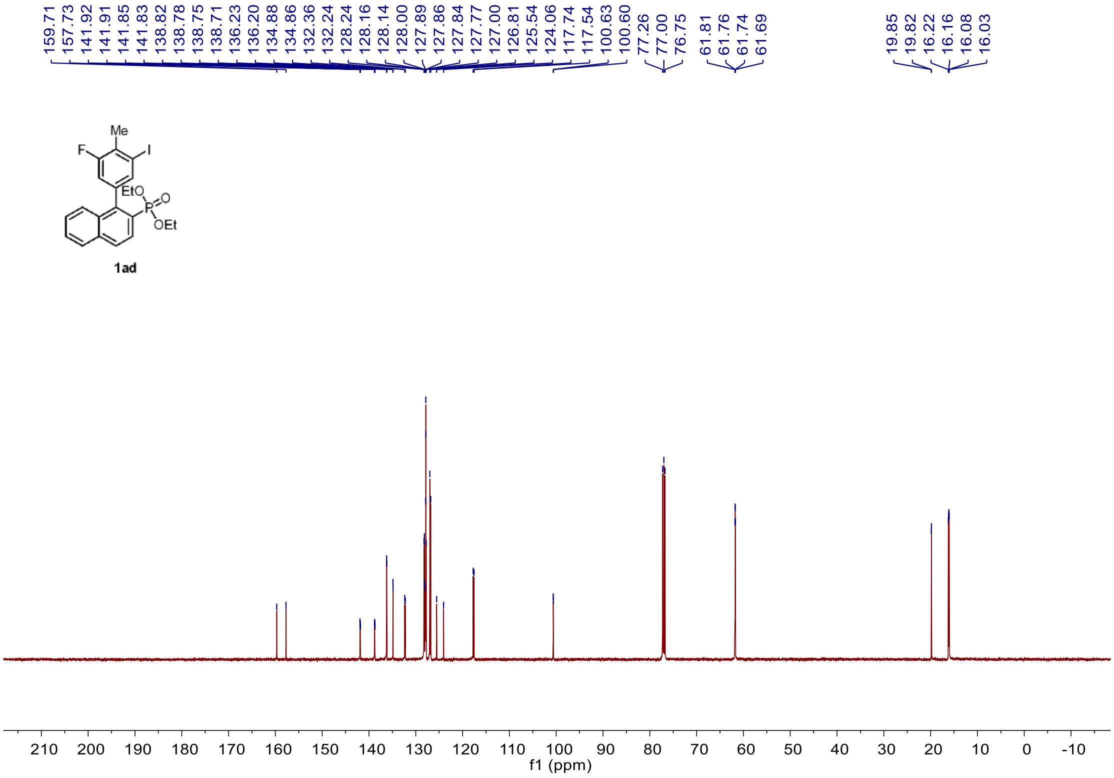
159.71 157.73 141.92 141.91 141.85 141.83 138.82 138.78 138.75 138.71 136.23 136.20 134.88 134.86 132.36 132.24 128.24 128.16 128.14 128.00 127.89 127.86 127.84 127.77 127.00 126.81 125.54 124.06 117.74 117.54 100.63 100.60 77.26 77.00 76.75 61.81 61.76 61.74 61.69 19.85 19.82 16.22 16.16 16.08 16.03
Me
F
I
EtO
P
O
OEt
1ad
210 200 190 180 170 160 150 140 130 120 110 100 90 80 70 60 50 40 30 20 10 0 -10
f1 (ppm)

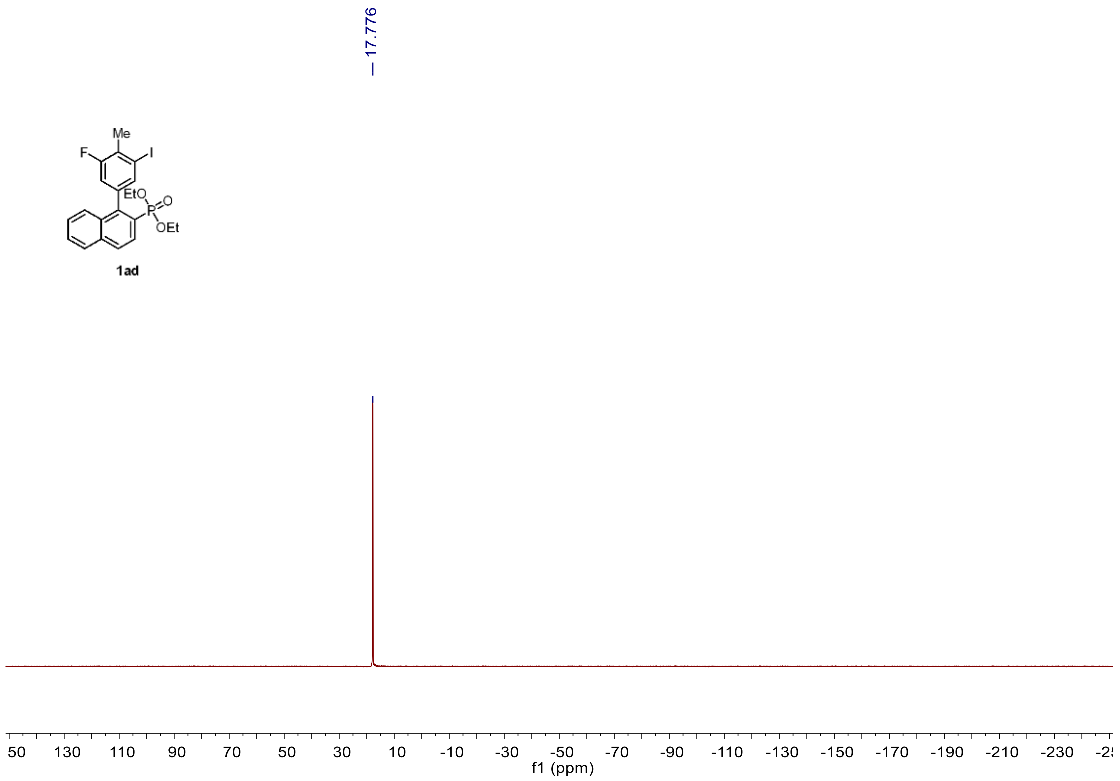
Me
F
I
EtO
P
O
OEt
17.776
50 130 110 90 70 50 30 10 -10 -30 -50 -70 -90 -110 -130 -150 -170 -190 -210 -230
f1 (ppm)

1ad

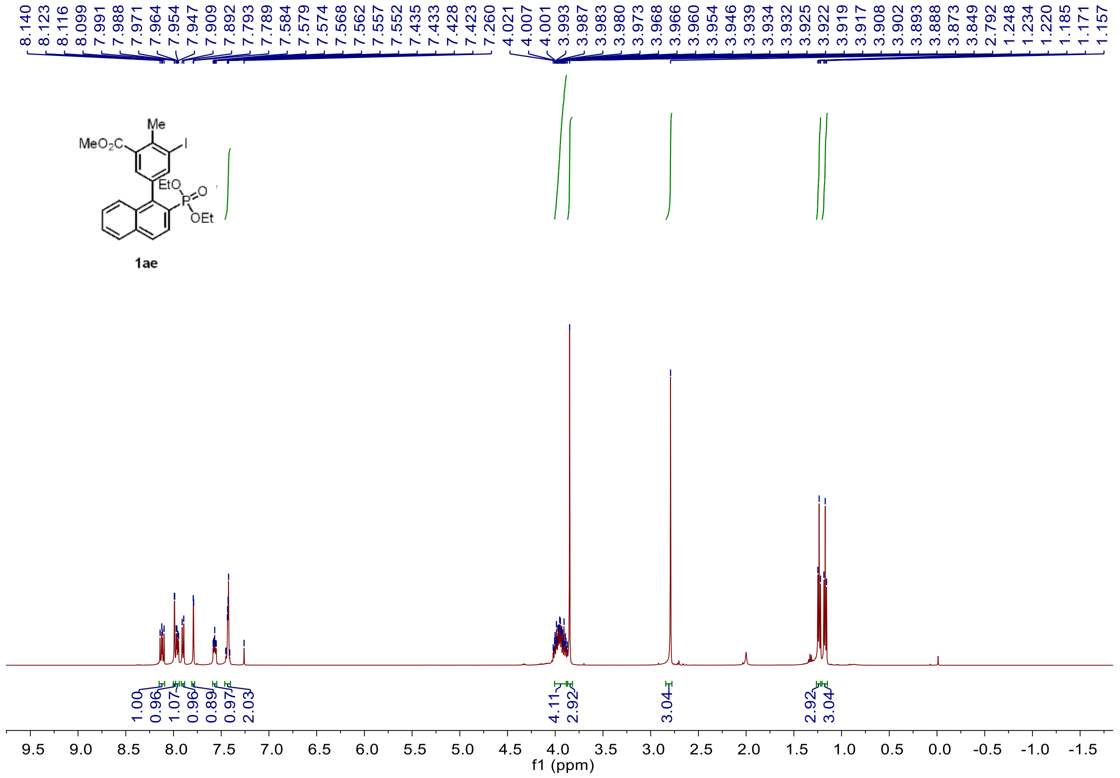
Me
MeO2C
I
EtO
P
O
OEt
8.140 8.123 8.116 8.099 7.991 7.988 7.971 7.964 7.954 7.947 7.909 7.892 7.793 7.789 7.584 7.579 7.574 7.568 7.562 7.557 7.552 7.435 7.433 7.428 7.423 7.260 4.021 4.007 4.001 3.993 3.987 3.983 3.980 3.973 3.968 3.966 3.960 3.954 3.946 3.939 3.934 3.932 3.925 3.922 3.919 3.917 3.908 3.902 3.893 3.888 3.873 3.849 2.792 1.248 1.234 1.220 1.185 1.171 1.157
1.00 0.96 1.07 0.96 0.89 0.97 2.03
4.11 2.92
3.04
2.92 3.04
9.5 9.0 8.5 8.0 7.5 7.0 6.5 6.0 5.5 5.0 4.5 4.0 3.5 3.0 2.5 2.0 1.5 1.0 0.5 0.0 -0.5 -1.0 -1.5
f1 (ppm)

1ae

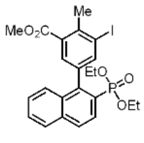
Me
MeO2C
I
EtO
P
O
OEt

1ae

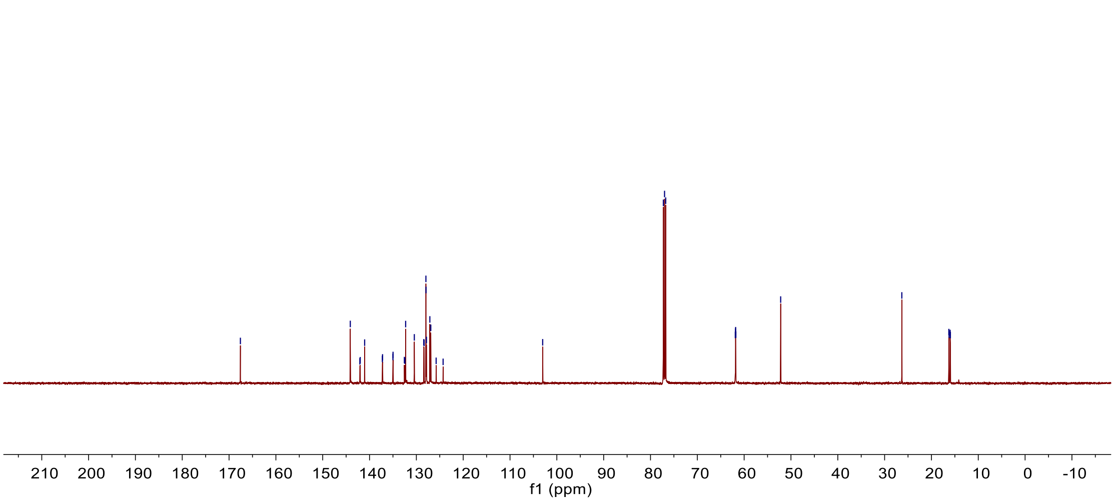
167.58
144.11
142.07
141.99
141.05
137.26
137.22
135.00
134.98
132.56
132.44
132.29
130.44
128.42
128.34
127.96
127.94
127.85
127.12
126.92
125.78
124.29
103.02
77.25
77.00
76.75
61.89
61.85
61.82
61.77
52.20
26.33
16.29
16.23
16.01
15.95
210 200 190 180 170 160 150 140 130 120 110 100 90 80 70 60 50 40 30 20 10 0 -10
f1 (ppm)

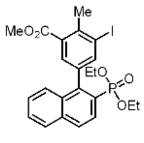
Me
MeO2C
I
EtO
P
O
OEt

1ae

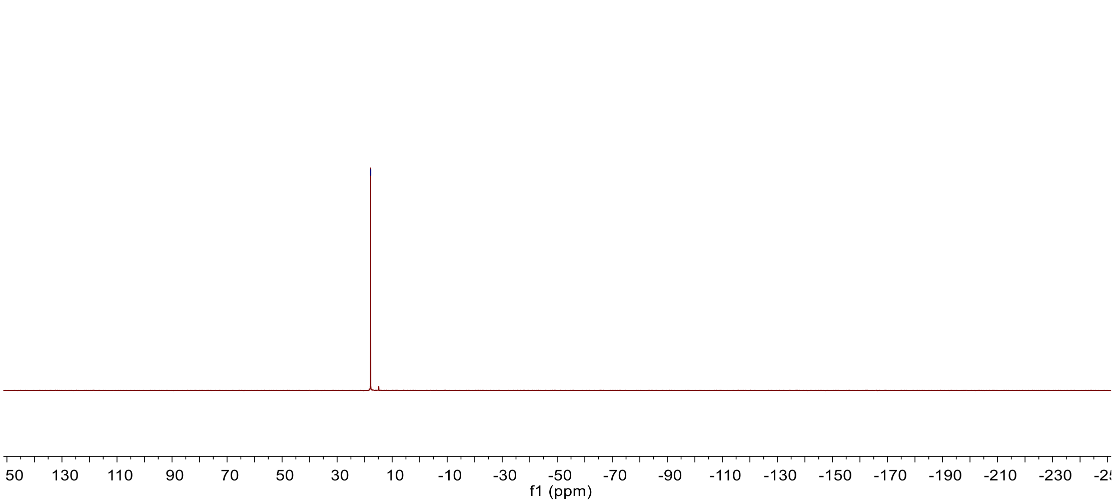
17.809
50 130 110 90 70 50 30 10 -10 -30 -50 -70 -90 -110 -130 -150 -170 -190 -210 -230
f1 (ppm)

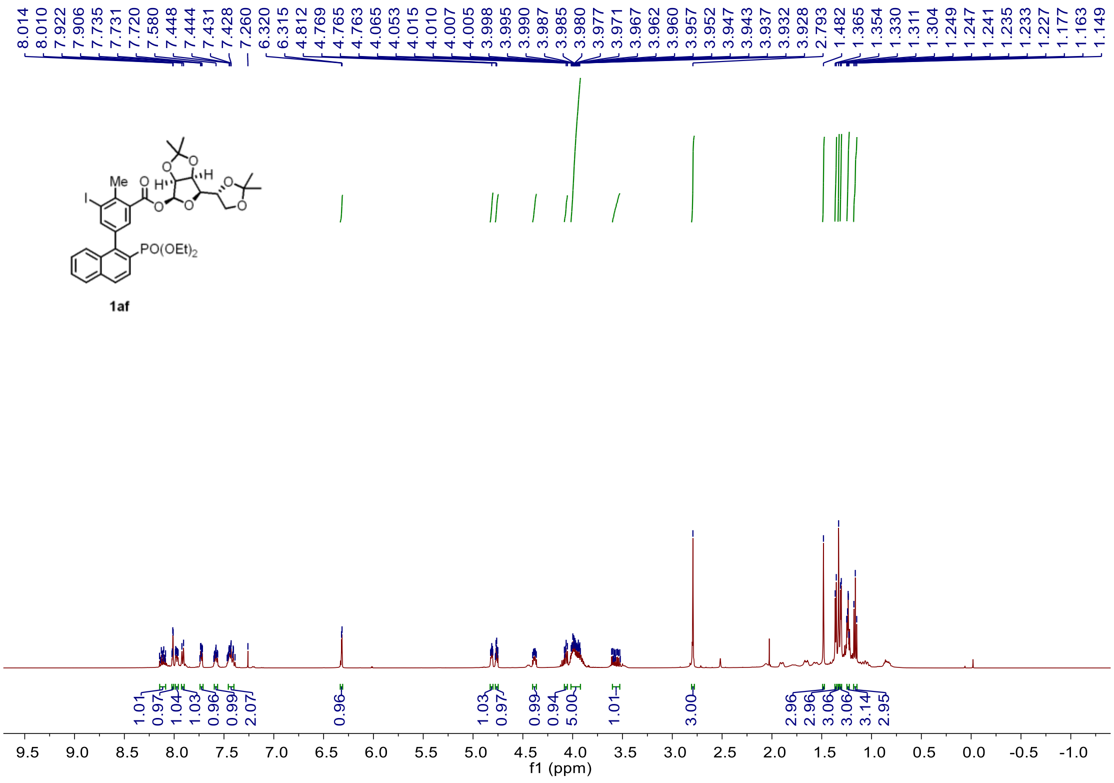
8.014
8.010
7.922
7.906
7.735
7.731
7.720
7.580
7.448
7.444
7.431
7.428
7.260
6.320
6.315
4.812
4.769
4.765
4.763
4.065
4.053
4.015
4.010
4.007
4.005
3.998
3.995
3.990
3.987
3.985
3.980
3.977
3.971
3.967
3.962
3.960
3.957
3.952
3.947
3.943
3.937
3.932
3.928
2.793
1.482
1.365
1.354
1.330
1.311
1.304
1.249
1.247
1.241
1.235
1.233
1.227
1.177
1.163
1.149
Me
I
O
H
PO(OEt)₂
1af
1.01
0.97
1.04
1.03
0.96
0.99
2.07
0.96
1.03
0.97
0.99
0.94
5.00
1.01
3.00
2.96
2.96
3.06
3.06
3.14
2.95
9.5 9.0 8.5 8.0 7.5 7.0 6.5 6.0 5.5 5.0 4.5 4.0 3.5 3.0 2.5 2.0 1.5 1.0 0.5 0.0 -0.5 -1.0
f1 (ppm)

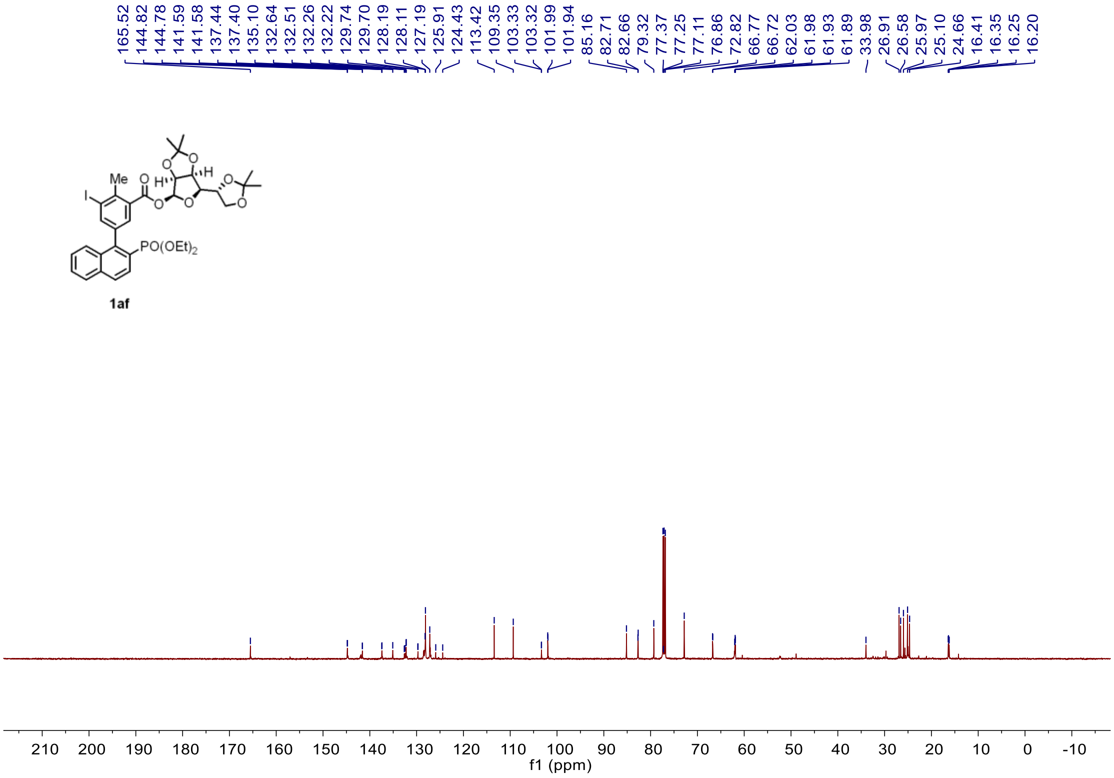
165.52
144.82
144.78
141.59
141.58
137.44
137.40
135.10
132.64
132.51
132.26
132.22
129.74
129.70
128.19
128.11
127.19
125.91
124.43
113.42
109.35
103.33
103.32
101.99
101.94
85.16
82.71
82.66
79.32
77.37
77.25
77.11
76.86
72.82
66.77
66.72
62.03
61.98
61.93
61.89
33.98
26.91
26.58
25.97
25.10
24.66
16.41
16.35
16.25
16.20
Me
I
O
H
PO(OEt)₂
1af
210 200 190 180 170 160 150 140 130 120 110 100 90 80 70 60 50 40 30 20 10 0 -10
f1 (ppm)

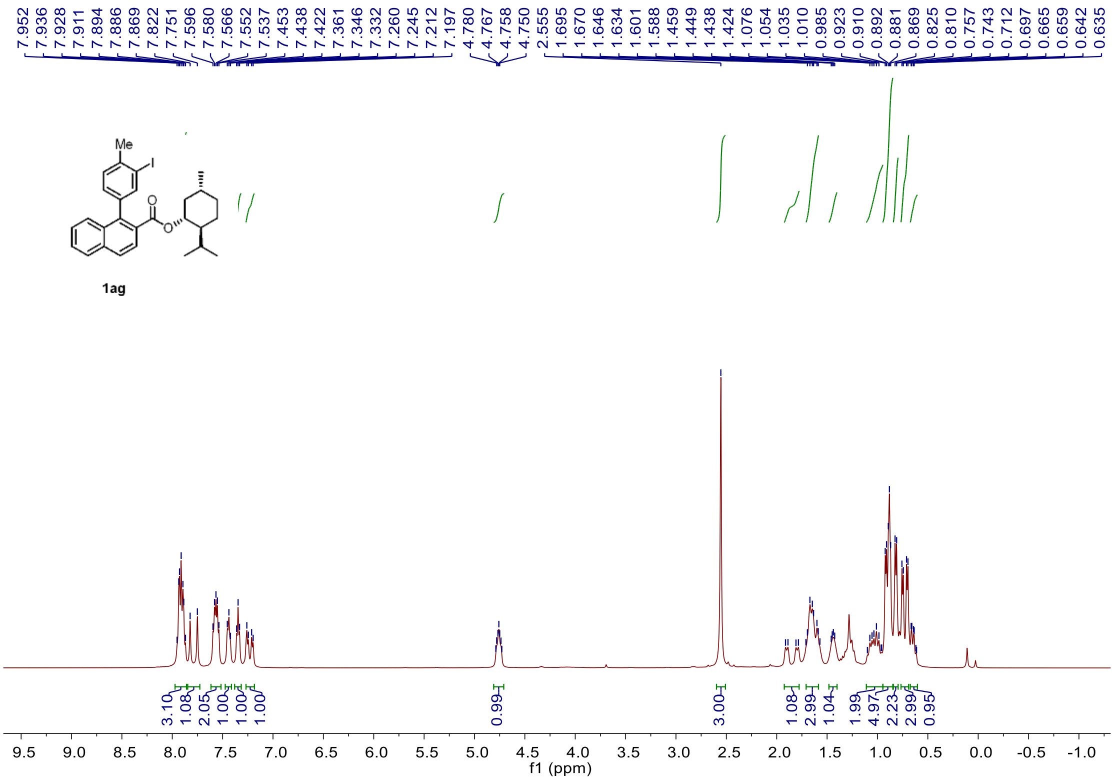
7.952 7.936 7.928 7.911 7.894 7.886 7.869 7.822 7.751 7.596 7.580 7.566 7.552 7.537 7.453 7.438 7.422 7.361 7.346 7.332 7.260 7.245 7.212 7.197 4.780 4.767 4.758 4.750 2.555 1.695 1.670 1.646 1.634 1.601 1.588 1.459 1.449 1.438 1.424 1.076 1.054 1.035 1.010 0.985 0.923 0.910 0.892 0.881 0.869 0.825 0.810 0.757 0.743 0.712 0.697 0.665 0.659 0.642 0.635
Me
I
O
O
1ag
3.10 1.08 2.05 1.00 1.00 1.00 0.99 3.00 1.08 2.99 1.04 1.99 4.97 2.23 2.99 0.95
9.5 9.0 8.5 8.0 7.5 7.0 6.5 6.0 5.5 5.0 4.5 4.0 3.5 3.0 2.5 2.0 1.5 1.0 0.5 0.0 -0.5 -1.0
f1 (ppm)

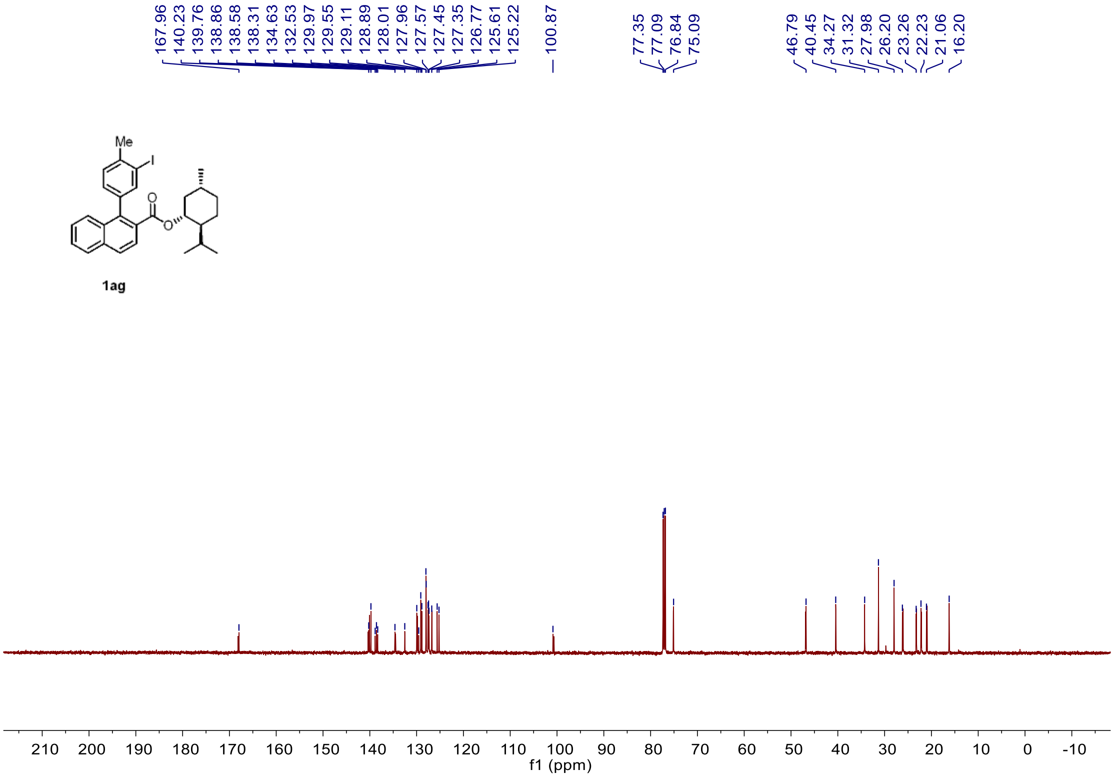
167.96 140.23 139.76 138.86 138.58 138.31 134.63 132.53 129.97 129.55 129.11 128.89 128.01 127.96 127.57 127.45 127.35 126.77 125.61 125.22 100.87 77.35 77.09 76.84 75.09 46.79 40.45 34.27 31.32 27.98 26.20 23.26 22.23 21.06 16.20
Me
I
O
O
1ag
210 200 190 180 170 160 150 140 130 120 110 100 90 80 70 60 50 40 30 20 10 0 -10
f1 (ppm)

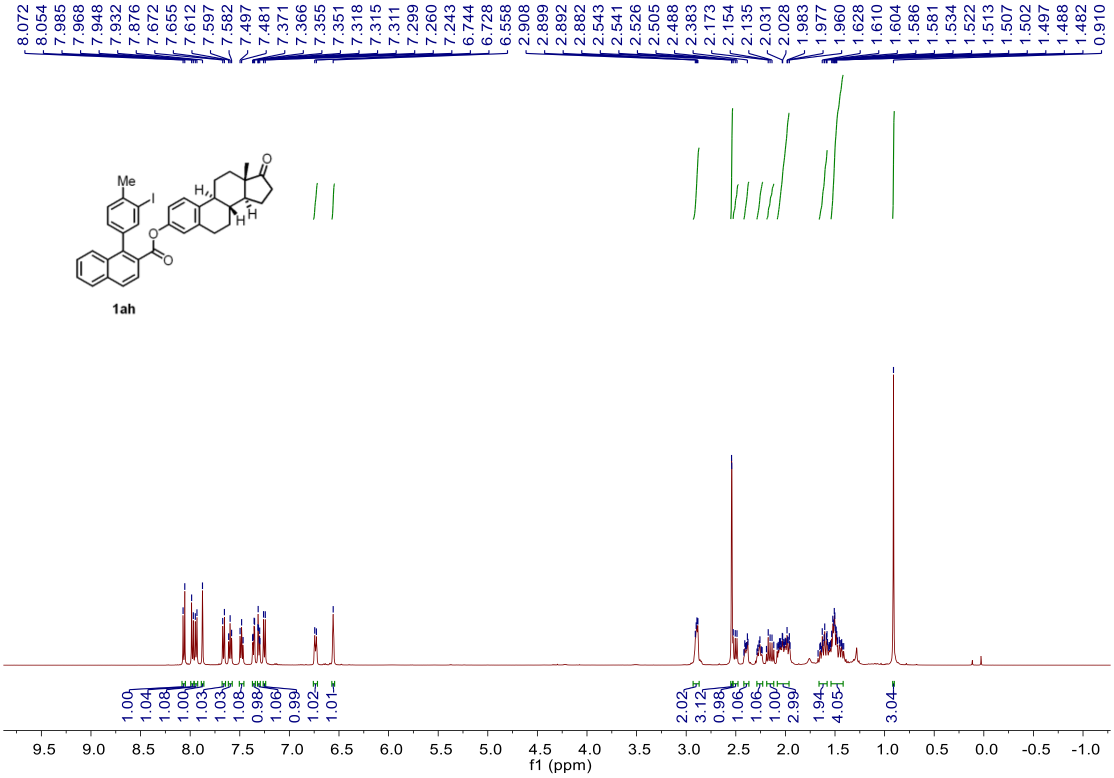
8.072 8.054 7.985 7.968 7.948 7.932 7.876 7.672 7.655 7.612 7.597 7.582 7.497 7.481 7.371 7.366 7.355 7.351 7.318 7.315 7.311 7.299 7.260 7.243 6.744 6.728 6.558 2.908 2.899 2.892 2.882 2.543 2.541 2.526 2.505 2.488 2.383 2.173 2.154 2.135 2.031 2.028 1.983 1.977 1.960 1.628 1.610 1.604 1.586 1.581 1.534 1.522 1.513 1.507 1.502 1.497 1.488 1.482 0.910
Me
I
O
H
1ah
1.00 1.04 1.08 1.00 1.03 1.03 1.08 0.98 1.06 0.99 1.02 1.01
2.02 3.12 0.98 1.06 1.06 1.00 2.99 1.94 4.05 3.04
9.5 9.0 8.5 8.0 7.5 7.0 6.5 6.0 5.5 5.0 4.5 4.0 3.5 3.0 2.5 2.0 1.5 1.0 0.5 0.0 -0.5 -1.0
f1 (ppm)

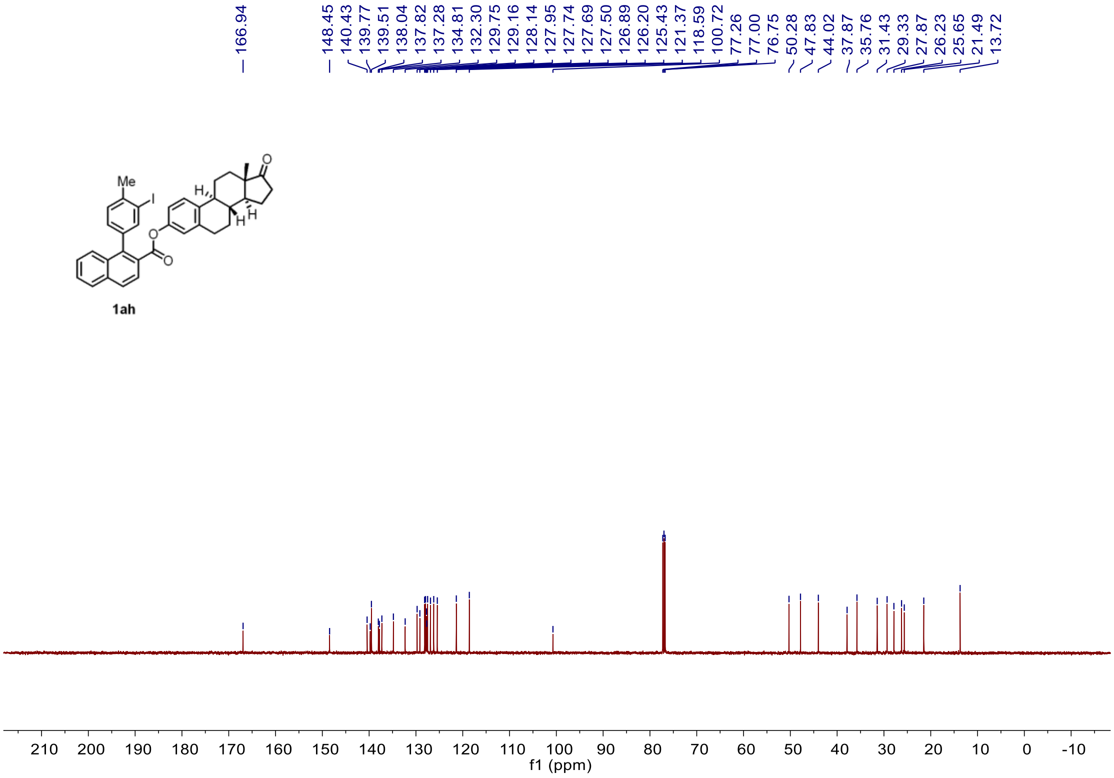
166.94
148.45 140.43 139.77 139.51 138.04 137.82 137.28 134.81 132.30 129.75 129.16 128.14 127.95 127.74 127.69 127.50 126.89 126.20 125.43 121.37 118.59 100.72 77.26 77.00 76.75 50.28 47.83 44.02 37.87 35.76 31.43 29.33 27.87 26.23 25.65 21.49 13.72
Me
I
O
H
1ah
210 200 190 180 170 160 150 140 130 120 110 100 90 80 70 60 50 40 30 20 10 0 -10
f1 (ppm)

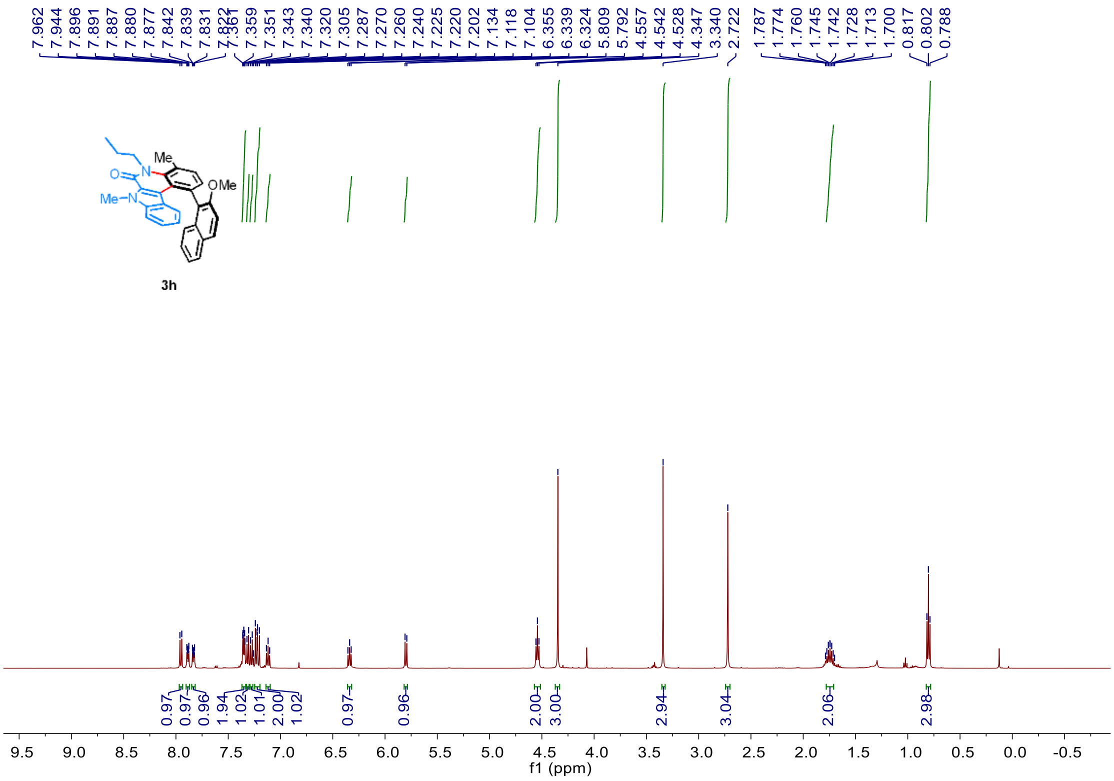
7.962
7.944
7.896
7.891
7.887
7.880
7.877
7.842
7.839
7.831
7.822
7.361
7.359
7.351
7.343
7.340
7.320
7.305
7.287
7.270
7.260
7.240
7.225
7.220
7.202
7.134
7.118
7.104
6.355
6.339
6.324
5.809
5.792
4.557
4.542
4.528
4.347
3.340
2.722
1.787
1.774
1.760
1.745
1.742
1.728
1.713
1.700
0.817
0.802
0.788
Me
O
N
OMe
Me–N
0.97
0.97
0.96
1.94
1.02
1.01
2.00
1.02
0.97
0.96
2.00
3.00
2.94
3.04
2.06
2.98
9.5 9.0 8.5 8.0 7.5 7.0 6.5 6.0 5.5 5.0 4.5 4.0 3.5 3.0 2.5 2.0 1.5 1.0 0.5 0.0 -0.5
f1 (ppm)

3h

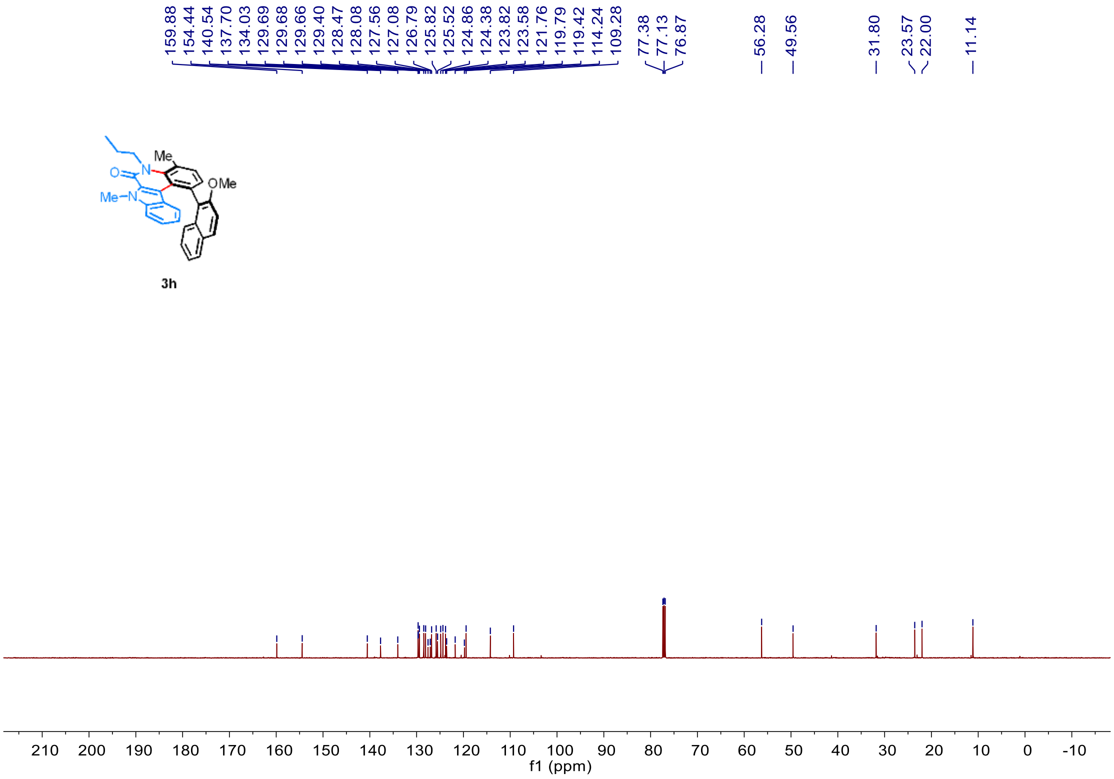
159.88
154.44
140.54
137.70
134.03
129.69
129.68
129.66
129.40
128.47
128.08
127.56
127.08
126.79
125.82
125.52
124.86
124.38
123.82
123.58
121.76
119.79
119.42
114.24
109.28
77.38
77.13
76.87
56.28
49.56
31.80
23.57
22.00
11.14
Me
O
N
OMe
Me–N
210 200 190 180 170 160 150 140 130 120 110 100 90 80 70 60 50 40 30 20 10 0 -10
f1 (ppm)

3h

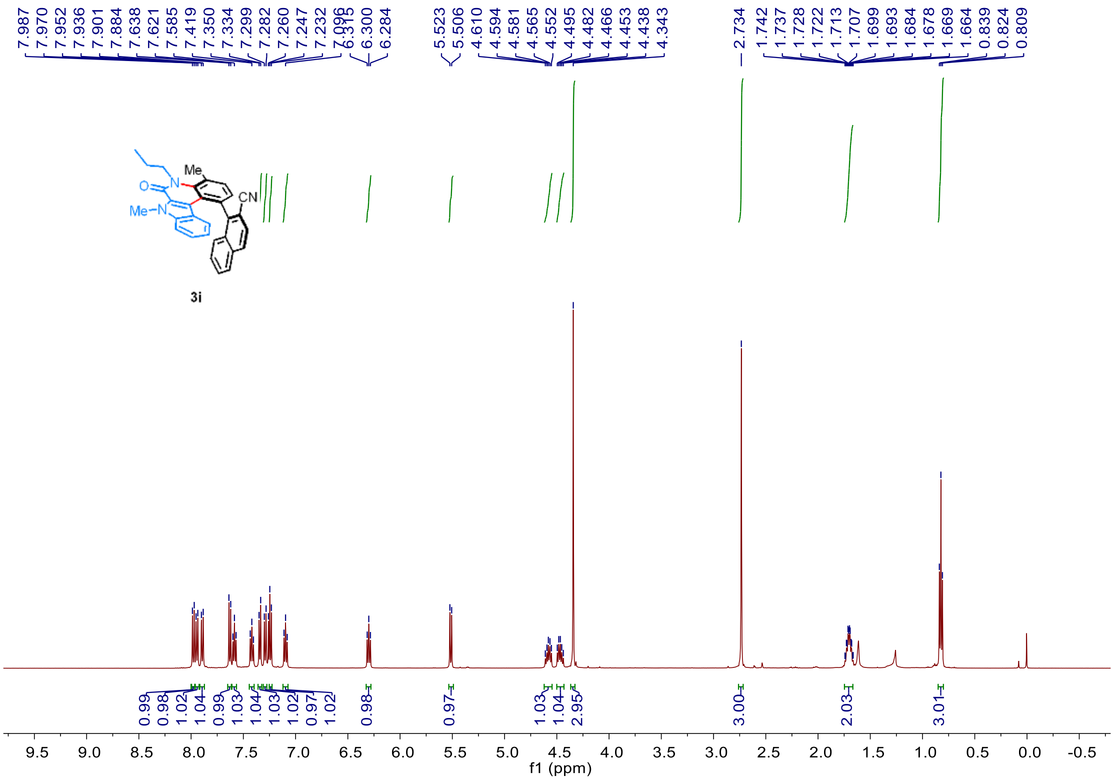
7.987
7.970
7.952
7.936
7.901
7.884
7.638
7.621
7.585
7.419
7.350
7.334
7.299
7.282
7.260
7.247
7.232
7.096
6.315
6.300
6.284
5.523
5.506
4.610
4.594
4.581
4.565
4.552
4.495
4.482
4.466
4.453
4.438
4.343
2.734
1.742
1.737
1.728
1.722
1.713
1.707
1.699
1.693
1.684
1.678
1.669
1.664
0.839
0.824
0.809
Me
CN
O
N
Me–N
3i
0.99
0.98
1.02
1.04
0.99
1.03
1.04
1.03
1.02
0.97
1.02
0.98
0.97
1.03
1.04
2.95
3.00
2.03
3.01
9.5
9.0
8.5
8.0
7.5
7.0
6.5
6.0
5.5
5.0
4.5
4.0
3.5
3.0
2.5
2.0
1.5
1.0
0.5
0.0
-0.5
f1 (ppm)

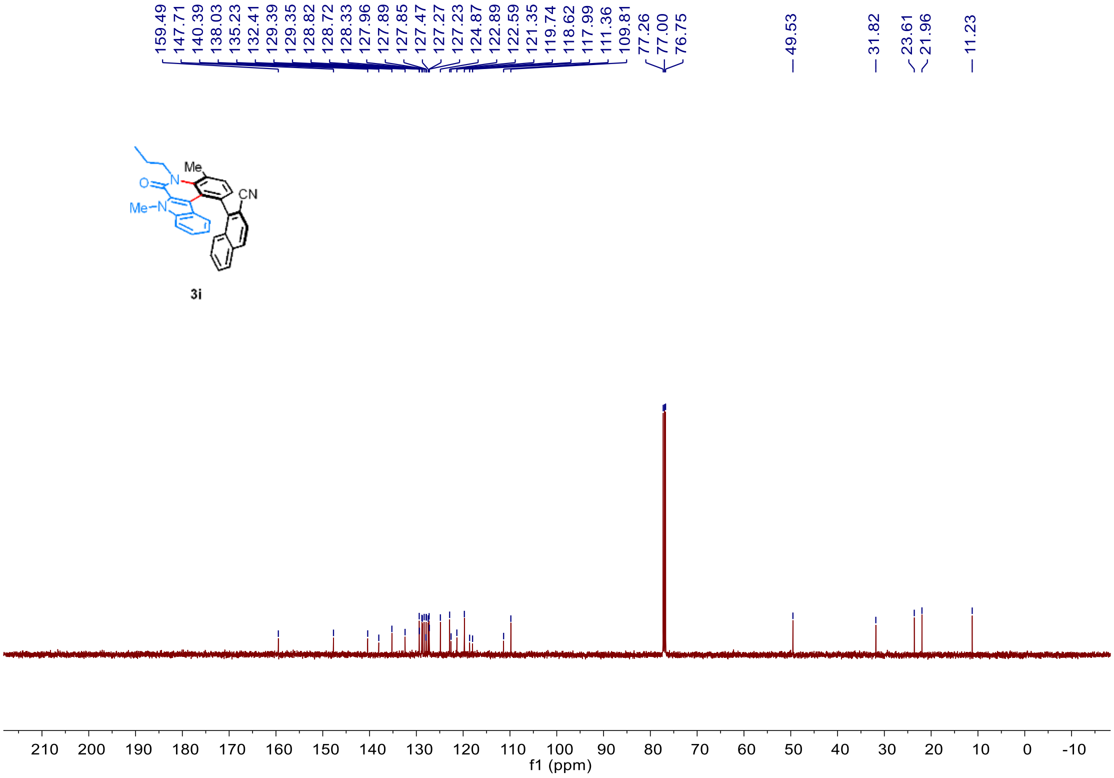
159.49
147.71
140.39
138.03
135.23
132.41
129.39
129.35
128.82
128.72
128.33
127.96
127.89
127.85
127.47
127.27
127.23
124.87
122.89
122.59
121.35
119.74
118.62
117.99
111.36
109.81
77.26
77.00
76.75
49.53
31.82
23.61
21.96
11.23
Me
CN
O
N
Me–N
3i
210
200
190
180
170
160
150
140
130
120
110
100
90
80
70
60
50
40
30
20
10
0
-10
f1 (ppm)

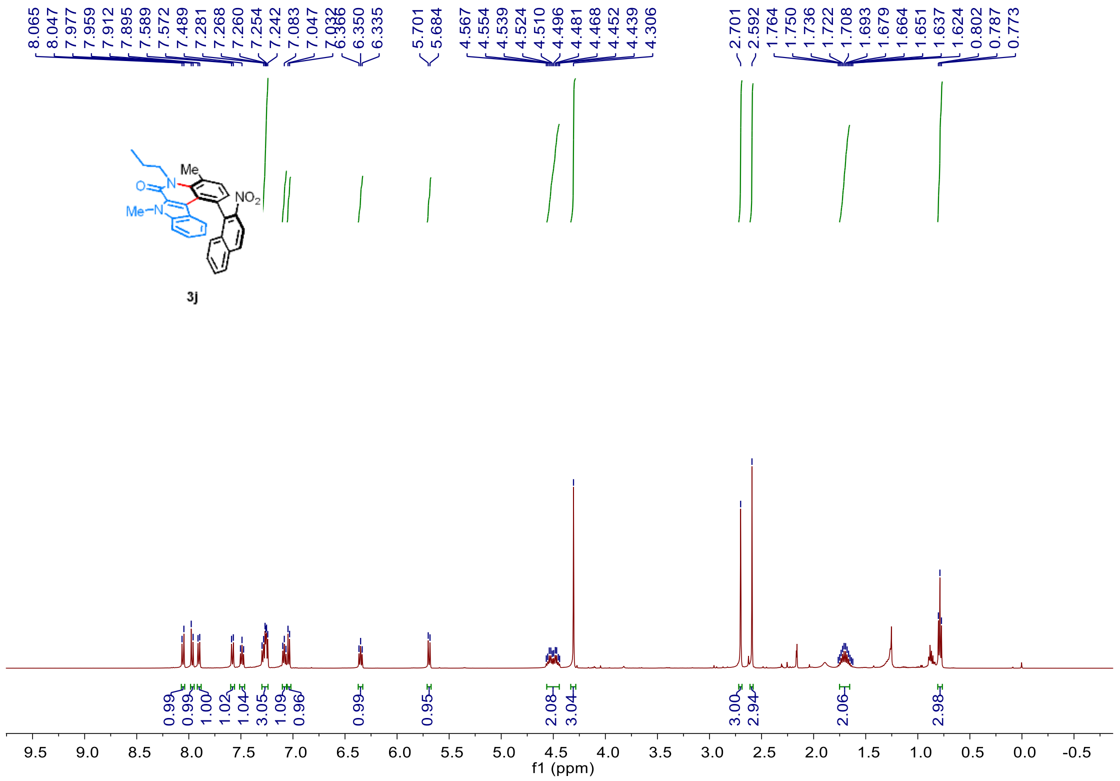

8.065
8.047
7.977
7.959
7.912
7.895
7.589
7.572
7.489
7.281
7.268
7.260
7.254
7.242
7.083
7.047
7.032
6.366
6.350
6.335
5.701
5.684
4.567
4.554
4.539
4.524
4.510
4.496
4.481
4.468
4.452
4.439
4.306
2.701
2.592
1.764
1.750
1.736
1.722
1.708
1.693
1.679
1.664
1.651
1.637
1.624
0.802
0.787
0.773
Me
NO2
O
N
Me
N
3j
0.99
0.99
1.00
1.02
1.04
3.05
1.09
0.96
0.99
0.95
2.08
3.04
3.00
2.94
2.06
2.98
9.5
9.0
8.5
8.0
7.5
7.0
6.5
6.0
5.5
5.0
4.5
4.0
3.5
3.0
2.5
2.0
1.5
1.0
0.5
0.0
-0.5
f1 (ppm)

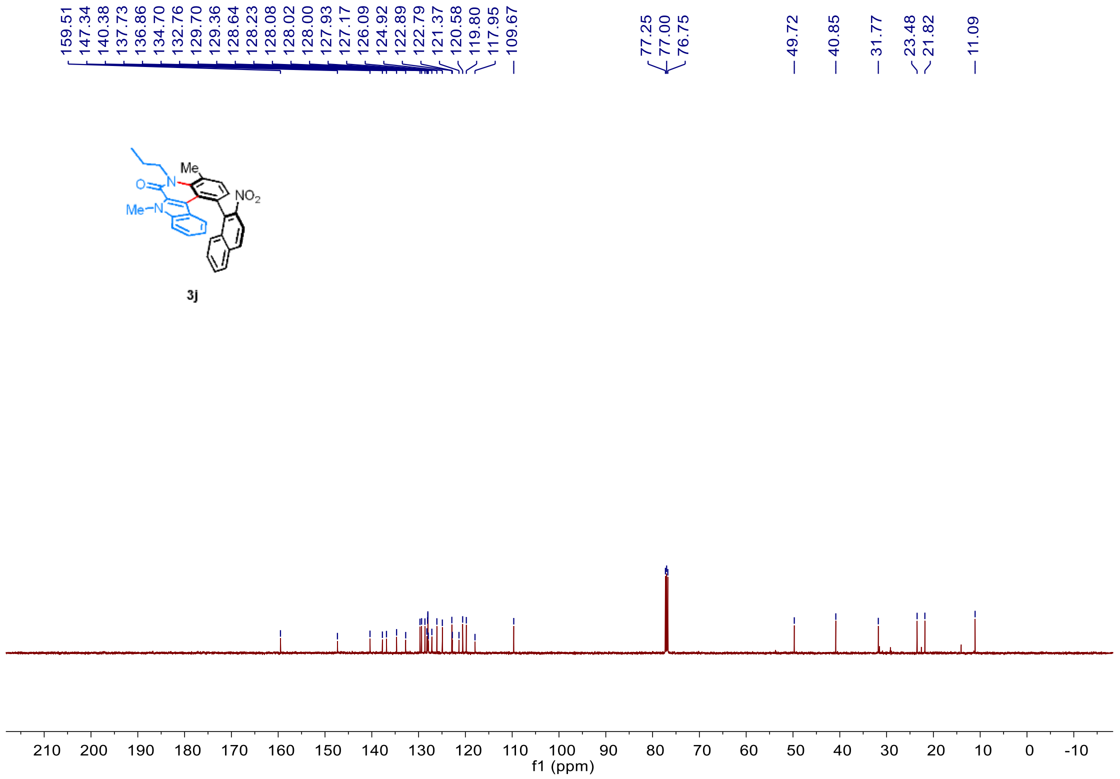

159.51
147.34
140.38
137.73
136.86
134.70
132.76
129.70
129.36
128.64
128.23
128.08
128.02
128.00
127.93
127.17
126.09
124.92
122.89
122.79
121.37
120.58
119.80
117.95
109.67
77.25
77.00
76.75
49.72
40.85
31.77
23.48
21.82
11.09
Me
NO2
O
N
Me
N
3j
210
200
190
180
170
160
150
140
130
120
110
100
90
80
70
60
50
40
30
20
10
0
-10
f1 (ppm)

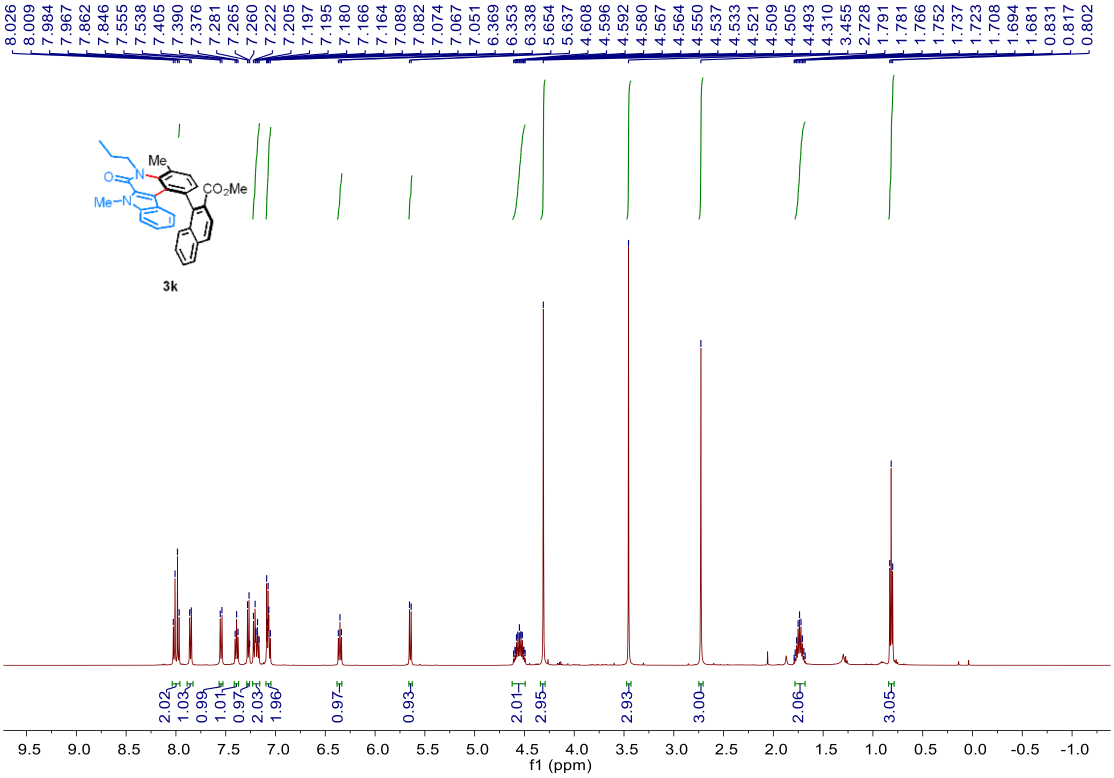
8.026 8.009 7.984 7.967 7.862 7.846 7.555 7.538 7.405 7.390 7.376 7.281 7.265 7.260 7.222 7.205 7.197 7.195 7.180 7.166 7.164 7.089 7.082 7.074 7.067 7.051 6.369 6.353 6.338 5.654 5.637 4.608 4.596 4.592 4.580 4.567 4.564 4.550 4.537 4.533 4.521 4.509 4.505 4.493 4.310 3.455 2.728 1.791 1.781 1.766 1.752 1.737 1.723 1.708 1.694 1.681 0.831 0.817 0.802
Me
CO2Me
O
N
Me–N
3k
2.02 1.03 0.99 1.01 0.97 2.03 1.96 0.97 0.93 2.01 2.95 2.93 3.00 2.06 3.05
9.5 9.0 8.5 8.0 7.5 7.0 6.5 6.0 5.5 5.0 4.5 4.0 3.5 3.0 2.5 2.0 1.5 1.0 0.5 0.0 -0.5 -1.0
f1 (ppm)

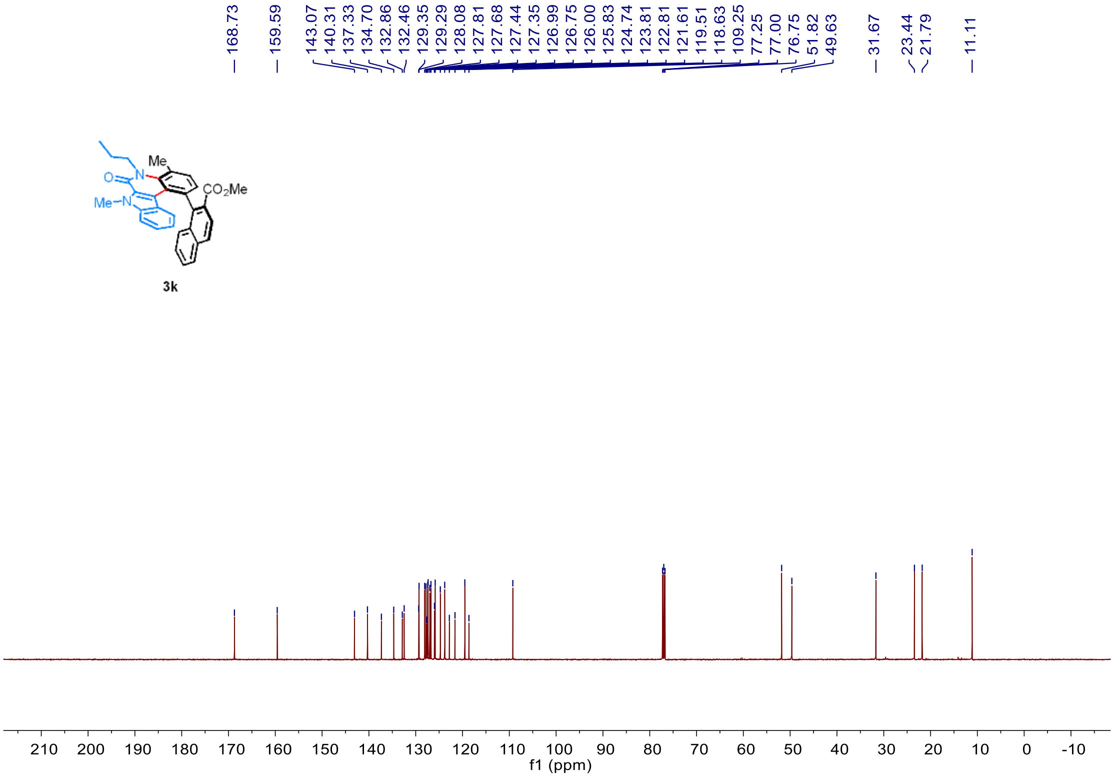
168.73 159.59 143.07 140.31 137.33 134.70 132.86 132.46 129.35 129.29 128.08 127.81 127.68 127.44 127.35 126.99 126.75 126.00 125.83 124.74 123.81 122.81 121.61 119.51 118.63 109.25 77.25 77.00 76.75 51.82 49.63 31.67 23.44 21.79 11.11
Me
CO2Me
O
N
Me–N
3k
210 200 190 180 170 160 150 140 130 120 110 100 90 80 70 60 50 40 30 20 10 0 -10
f1 (ppm)

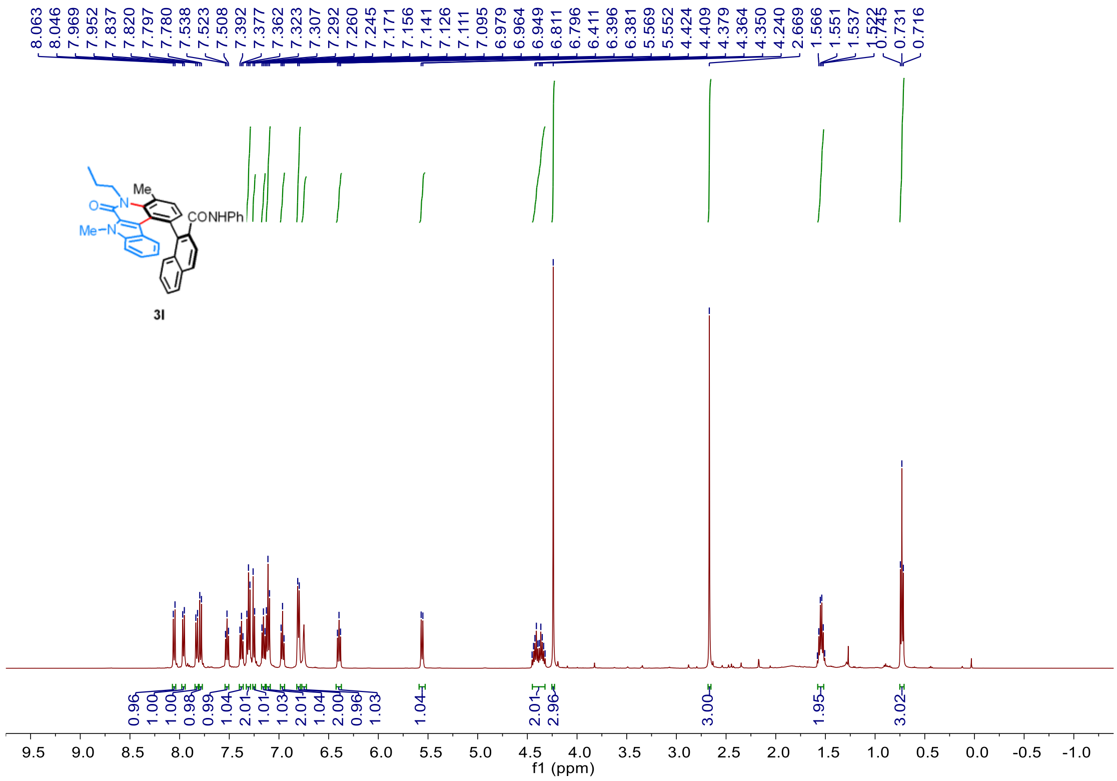

8.063
8.046
7.969
7.952
7.837
7.820
7.797
7.780
7.538
7.523
7.508
7.392
7.377
7.362
7.323
7.307
7.292
7.260
7.245
7.171
7.156
7.141
7.126
7.111
7.095
6.979
6.964
6.949
6.811
6.796
6.411
6.396
6.381
5.569
5.552
4.424
4.409
4.379
4.364
4.350
4.240
2.669
1.566
1.551
1.537
1.522
0.745
0.731
0.716
Me
O
N
Me–N
CONHPh
3l
0.96
1.00
0.98
0.99
1.04
2.01
1.01
1.03
2.01
1.04
2.00
0.96
1.03
1.04
2.01
2.96
3.00
1.95
3.02
9.5 9.0 8.5 8.0 7.5 7.0 6.5 6.0 5.5 5.0 4.5 4.0 3.5 3.0 2.5 2.0 1.5 1.0 0.5 0.0 -0.5 -1.0
f1 (ppm)

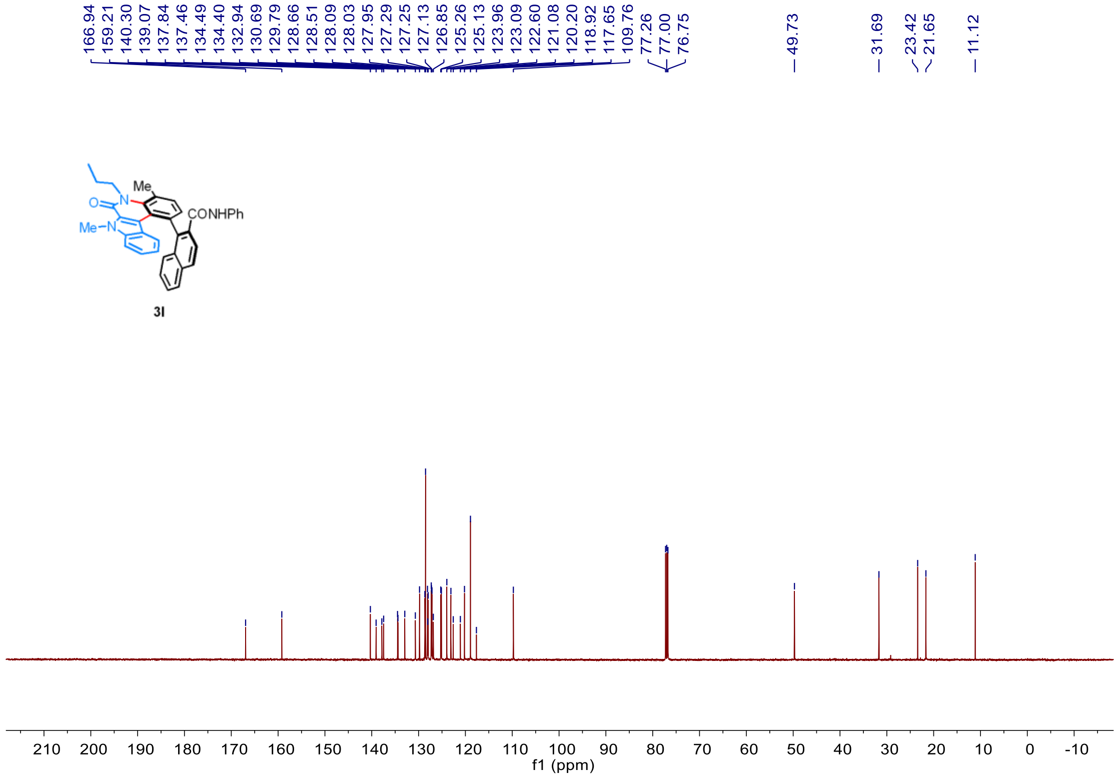

166.94
159.21
140.30
139.07
137.84
137.46
134.49
134.40
132.94
130.69
129.79
128.66
128.51
128.09
128.03
127.95
127.29
127.25
127.13
126.85
125.26
125.13
123.96
123.09
122.60
121.08
120.20
118.92
117.65
109.76
77.26
77.00
76.75
49.73
31.69
23.42
21.65
11.12
Me
O
N
Me–N
CONHPh
3l
210 200 190 180 170 160 150 140 130 120 110 100 90 80 70 60 50 40 30 20 10 0 -10
f1 (ppm)

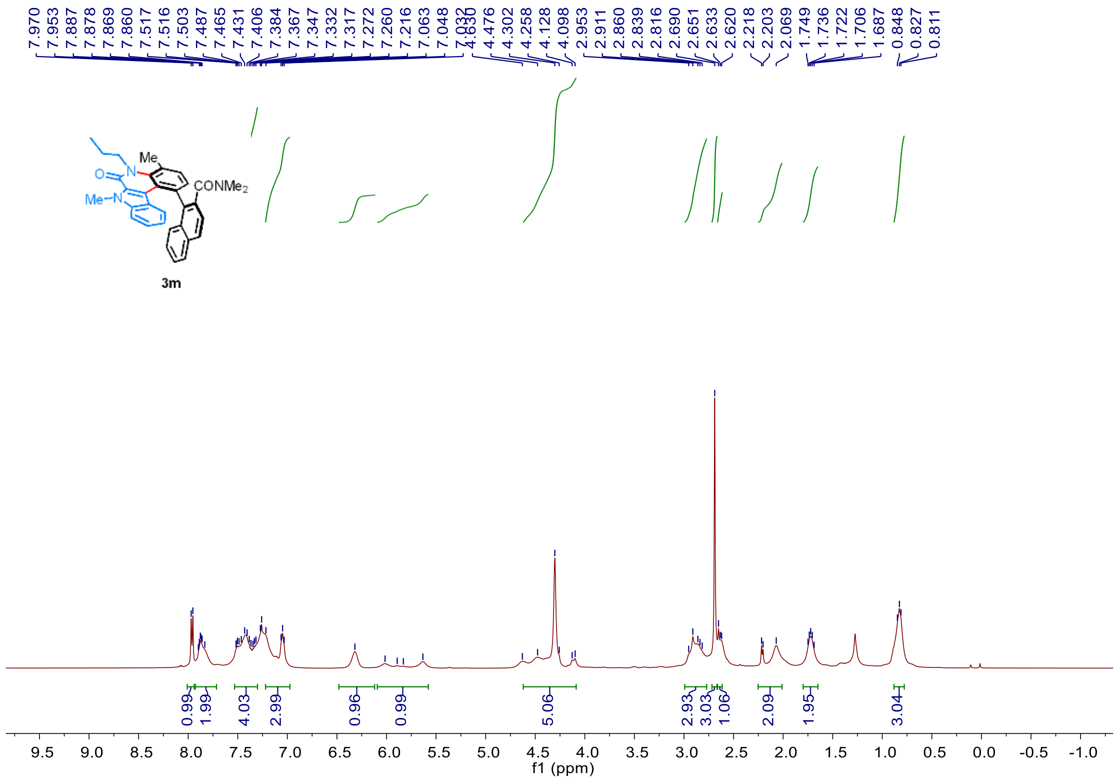

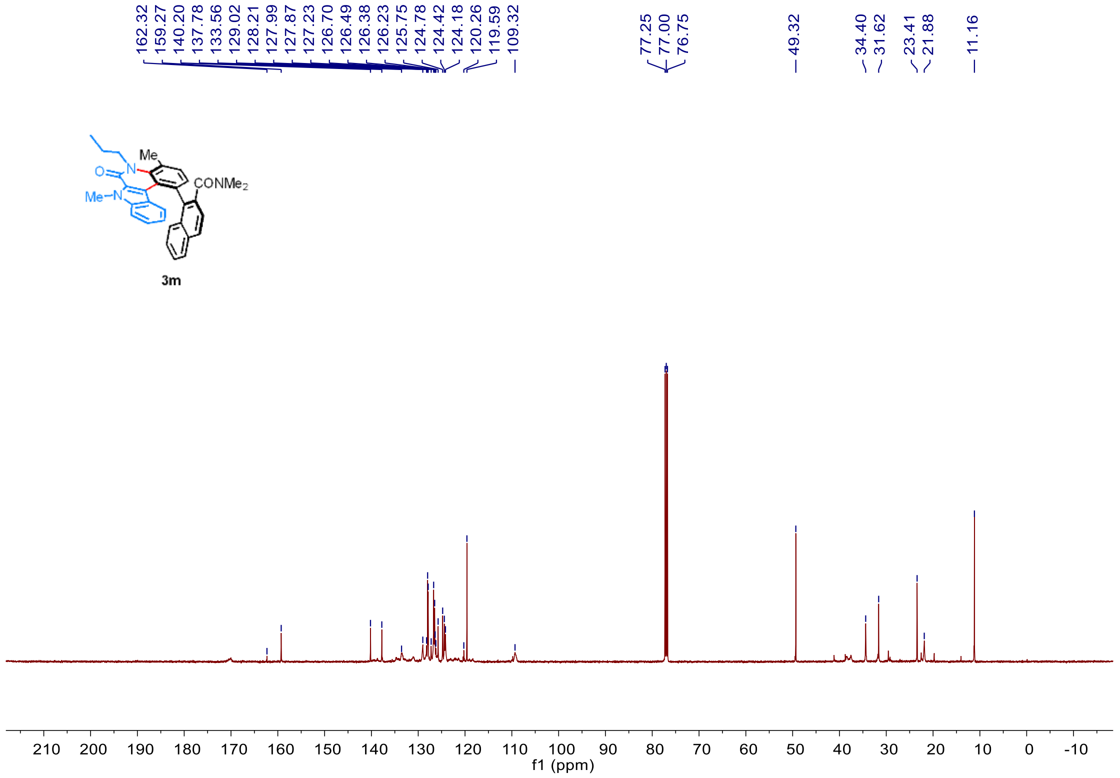

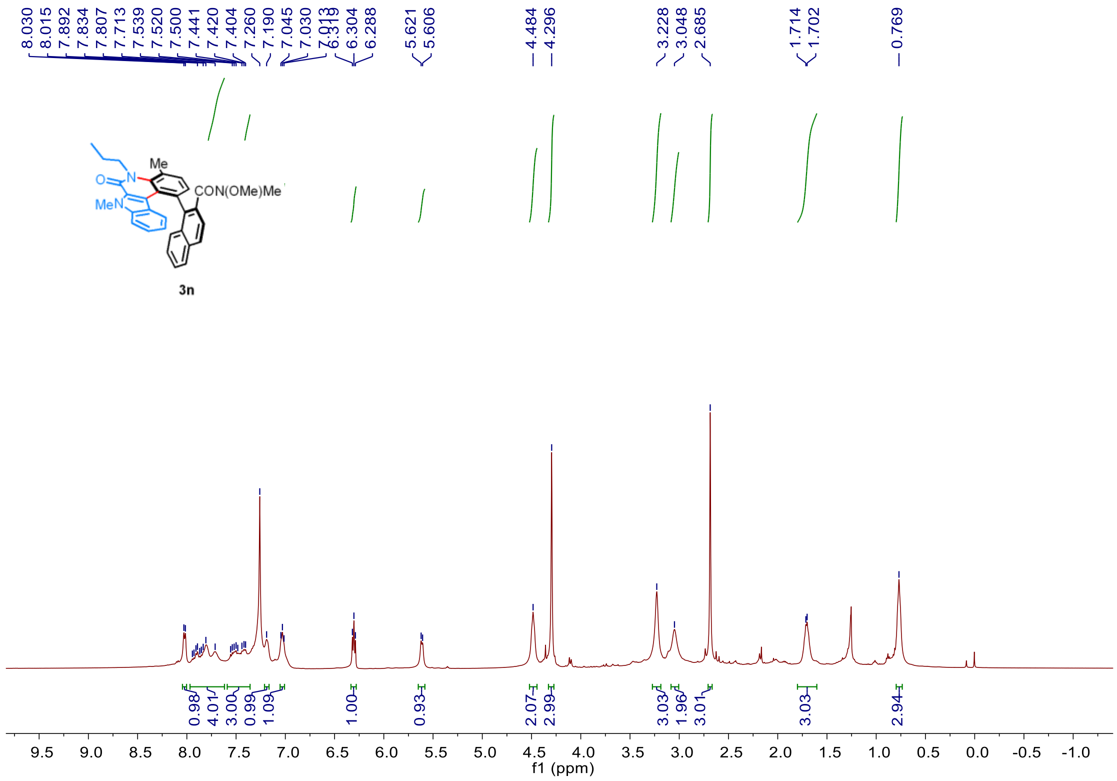

8.030
8.015
7.892
7.834
7.807
7.713
7.539
7.520
7.500
7.441
7.420
7.404
7.260
7.190
7.045
7.030
7.013
6.319
6.304
6.288
5.621
5.606
4.484
4.296
3.228
3.048
2.685
1.714
1.702
0.769
Me
CON(OMe)Me
MeN
O
N
0.98
4.01
3.00
0.99
1.09
1.00
0.93
2.07
2.99
3.03
1.96
3.01
3.03
2.94
f1 (ppm)

3n

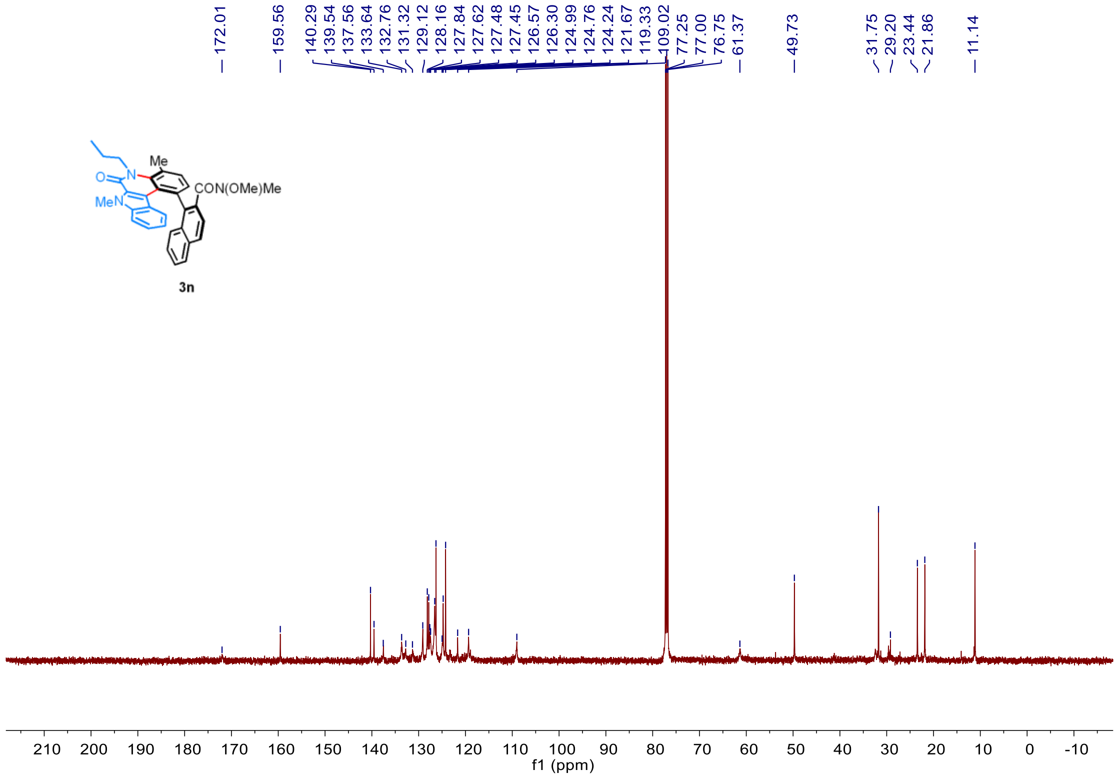

172.01
159.56
140.29
139.54
137.56
133.64
132.76
131.32
129.12
128.16
127.84
127.62
127.48
127.45
126.57
126.30
124.99
124.76
124.24
121.67
119.33
109.02
77.25
77.00
76.75
61.37
49.73
31.75
29.20
23.44
21.86
11.14
Me
CON(OMe)Me
MeN
O
N
f1 (ppm)

3n

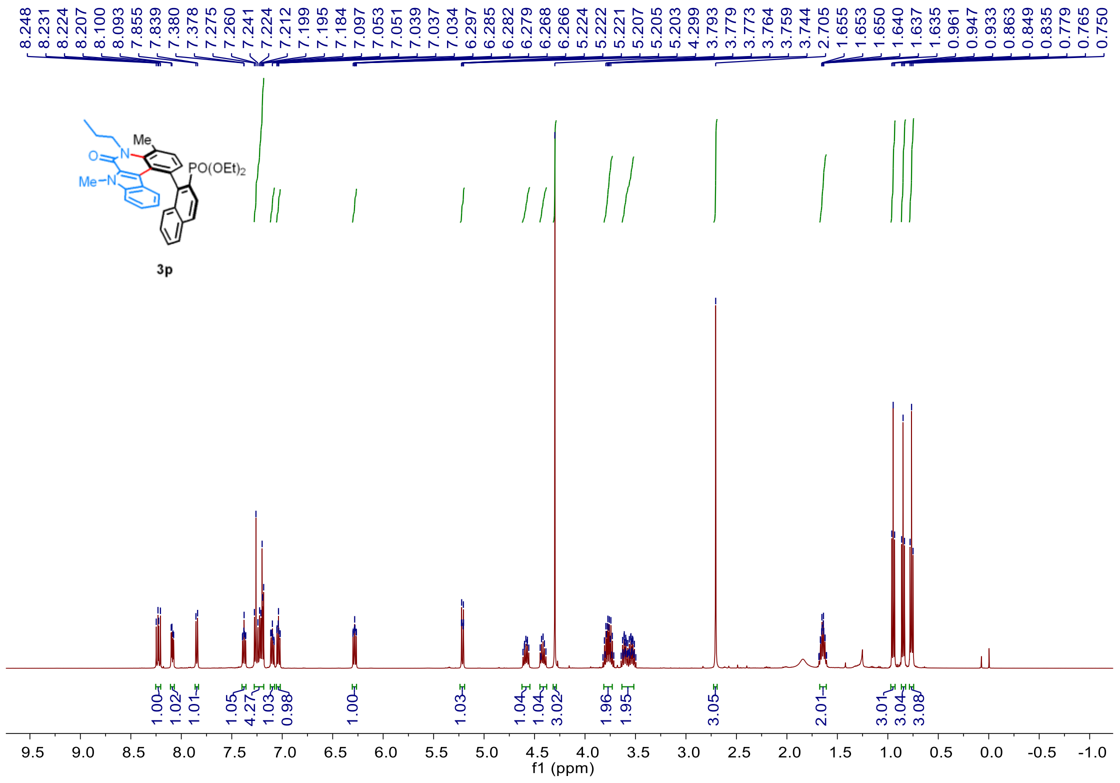

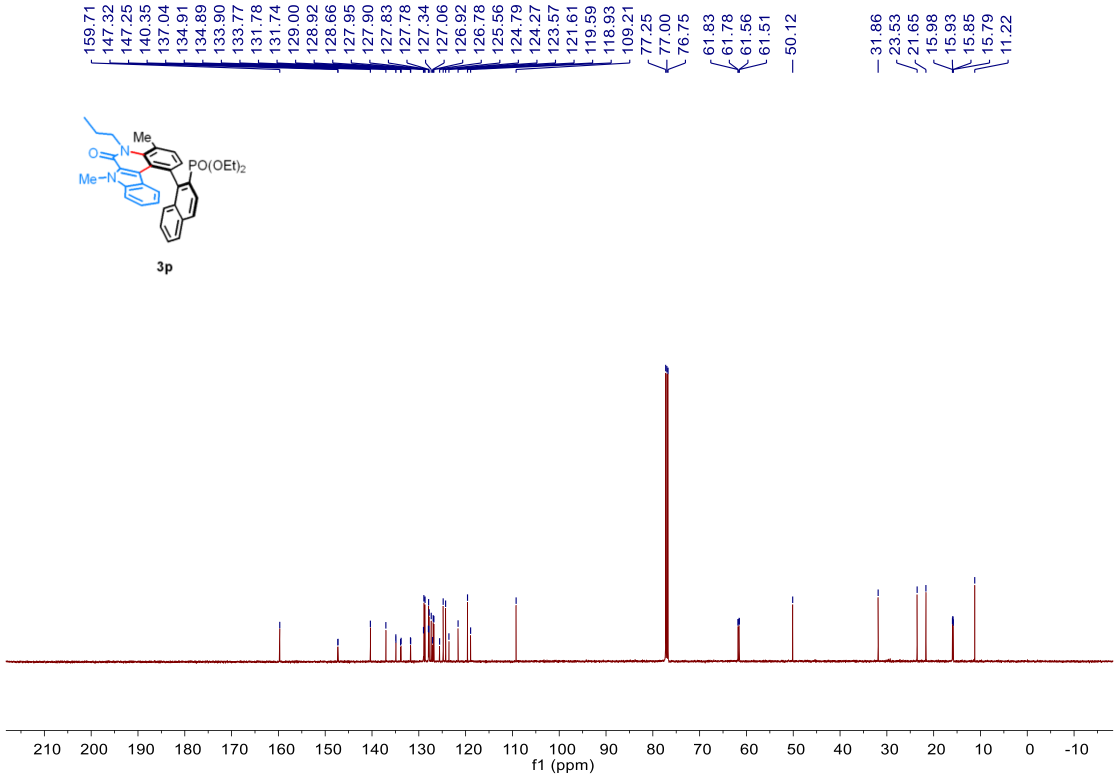

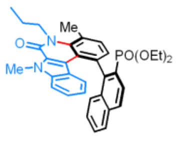

Me
PO(OEt)2
Me–N

3p

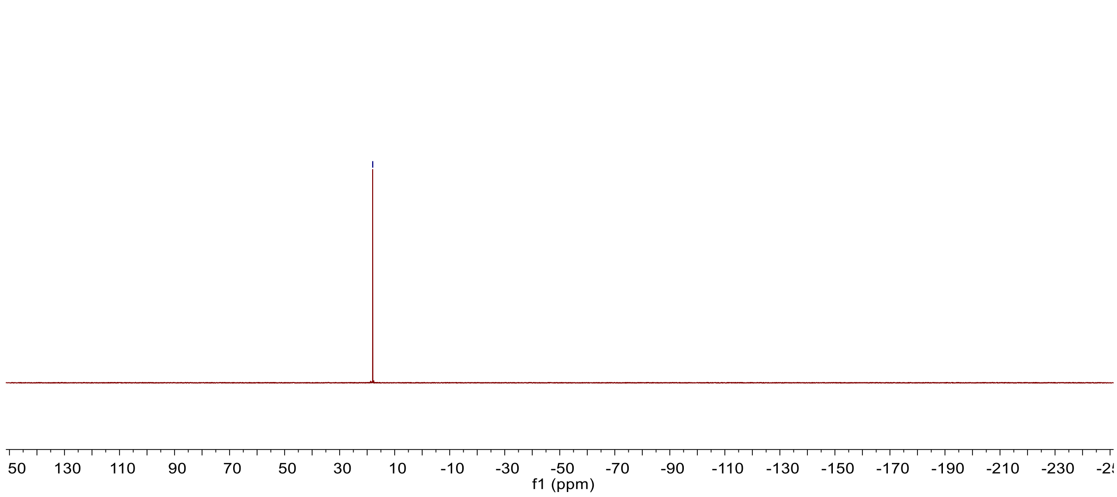

— 17.974
50 130 110 90 70 50 30 10 -10 -30 -50 -70 -90 -110 -130 -150 -170 -190 -210 -230
f1 (ppm)

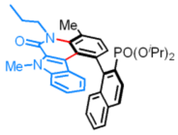

Me
PO(OiPr)2
Me–N

3q

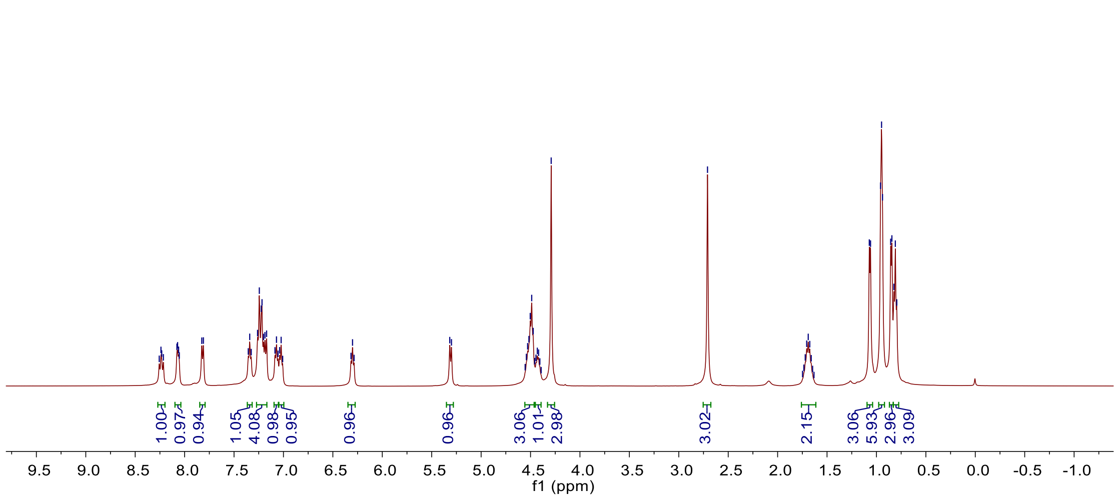

8.257 8.239 8.232 8.215 8.078 8.071 8.061 8.054 7.826 7.810 7.356 7.341 7.326 7.262 7.244 7.224 7.217 7.202 7.187 7.170 7.086 7.070 7.055 7.039 7.023 7.008 6.317 6.301 6.285 5.317 5.300 4.542 4.529 4.516 4.503 4.488 4.473 4.457 4.445 4.431 4.418 4.405 4.291 2.709 1.721 1.706 1.690 1.675 1.660 1.072 1.060 0.959 0.948 0.939 0.856 0.844 0.824 0.809 0.794
1.00 0.97 0.94 1.05 4.08 0.98 0.95 0.96 0.96 3.06 1.01 2.98 3.02 2.15 3.06 5.93 2.96 3.09
9.5 9.0 8.5 8.0 7.5 7.0 6.5 6.0 5.5 5.0 4.5 4.0 3.5 3.0 2.5 2.0 1.5 1.0 0.5 0.0 -0.5 -1.0
f1 (ppm)

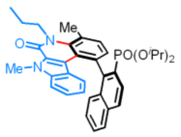
Me
N
O
Me
N
PO(O^{i}Pr)$_2$

3q

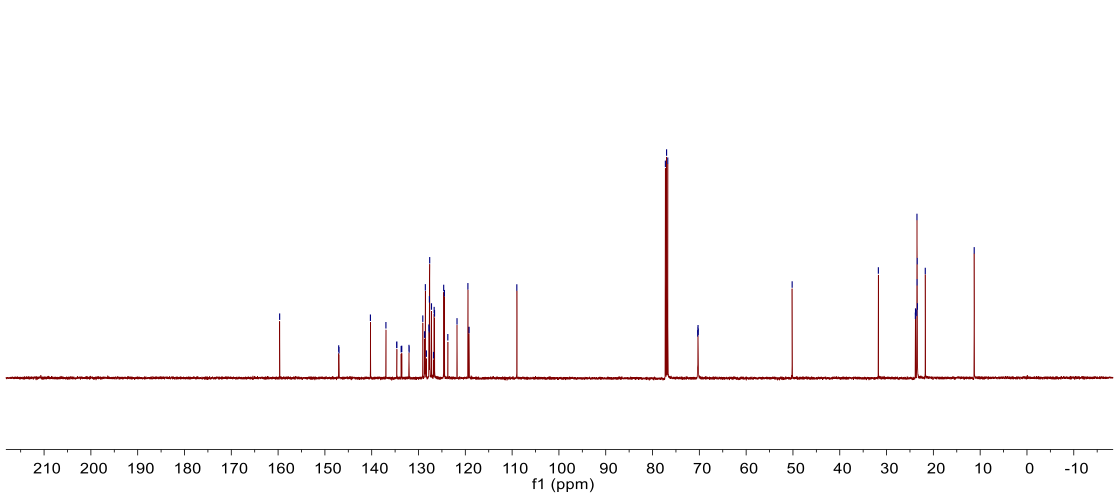
159.67
147.06
146.98
140.27
136.96
134.66
134.64
133.70
133.58
132.04
132.00
129.09
128.71
128.63
128.54
128.32
127.79
127.72
127.67
127.61
127.23
126.79
126.66
126.58
124.62
124.46
123.74
121.77
119.45
119.19
108.99
77.25
77.00
76.75
70.36
70.31
70.28
70.22
50.17
31.75
23.84
23.81
23.55
23.50
23.49
23.45
23.41
21.73
11.27
210 200 190 180 170 160 150 140 130 120 110 100 90 80 70 60 50 40 30 20 10 0 -10
f1 (ppm)

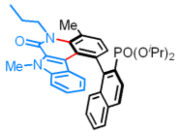
Me
N
O
Me
N
PO(O^{i}Pr)$_2$

3q

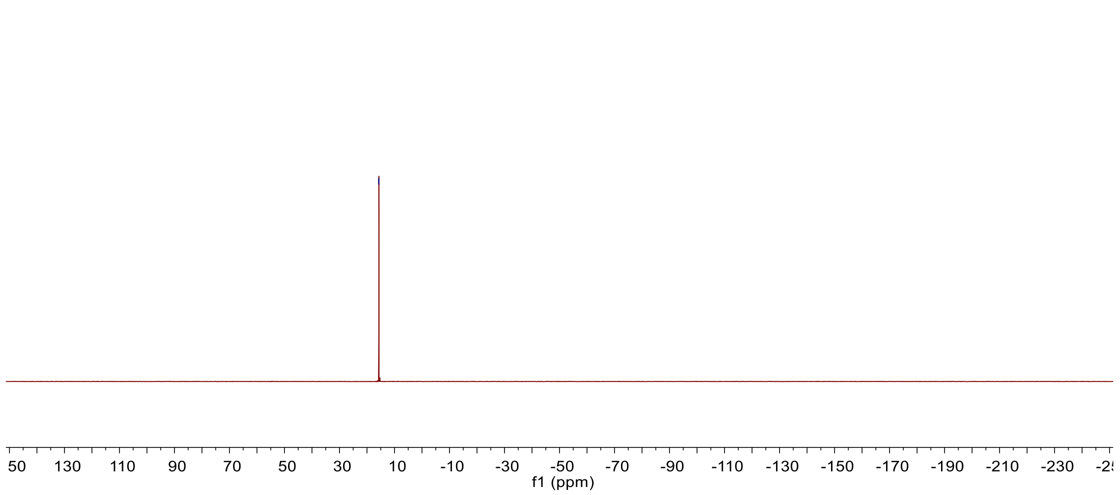
15.695
50 130 110 90 70 50 30 10 -10 -30 -50 -70 -90 -110 -130 -150 -170 -190 -210 -230
f1 (ppm)

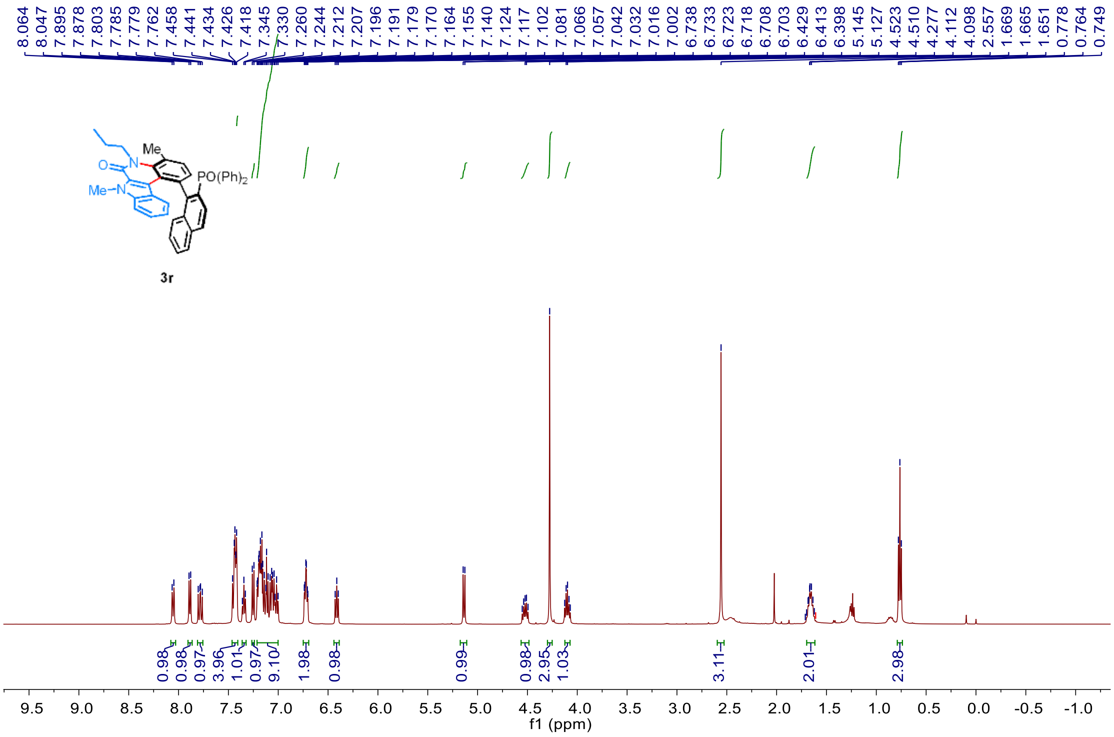
8.064 8.047 7.895 7.878 7.803 7.785 7.779 7.762 7.458 7.441 7.434 7.426 7.418 7.345 7.330 7.260 7.244 7.212 7.207 7.196 7.191 7.179 7.170 7.164 7.155 7.140 7.124 7.117 7.102 7.081 7.066 7.057 7.042 7.032 7.016 7.002 6.738 6.733 6.723 6.718 6.708 6.703 6.429 6.413 6.398 5.145 5.127 4.523 4.510 4.277 4.112 4.098 2.557 1.669 1.665 1.651 0.778 0.764 0.749
Me
O
N
Me
N
PO(Ph)2
3r
0.98 0.98 0.97 3.96 1.01 0.97 9.10 1.98 0.98 0.99 0.98 2.95 1.03 3.11 2.01 2.98
9.5 9.0 8.5 8.0 7.5 7.0 6.5 6.0 5.5 5.0 4.5 4.0 3.5 3.0 2.5 2.0 1.5 1.0 0.5 0.0 -0.5 -1.0
f1 (ppm)

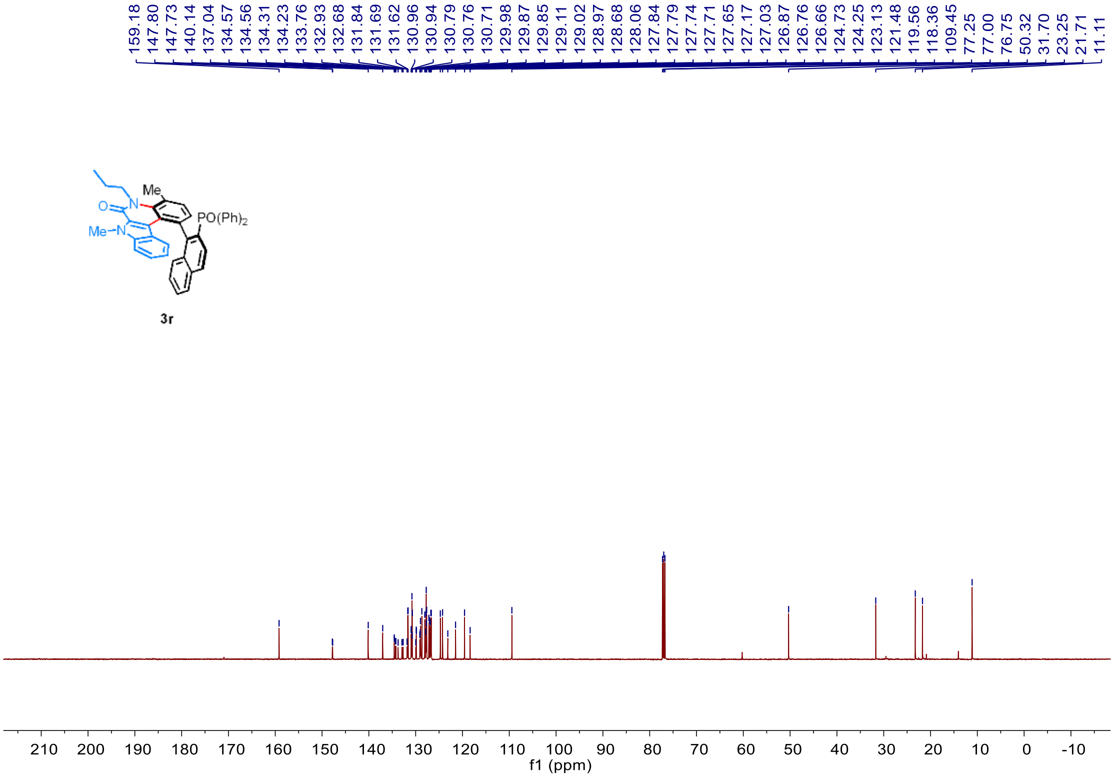
159.18 147.80 147.73 140.14 137.04 134.57 134.56 134.31 134.23 133.76 132.93 132.68 131.84 131.69 131.62 130.96 130.94 130.79 130.76 130.71 129.98 129.87 129.85 129.11 129.02 128.97 128.68 128.06 127.84 127.79 127.74 127.71 127.65 127.17 127.03 126.87 126.76 126.66 124.73 124.25 123.13 121.48 119.56 118.36 109.45 77.25 77.00 76.75 50.32 31.70 23.25 21.71 11.11
Me
O
N
Me
N
PO(Ph)2
3r
210 200 190 180 170 160 150 140 130 120 110 100 90 80 70 60 50 40 30 20 10 0 -10
f1 (ppm)

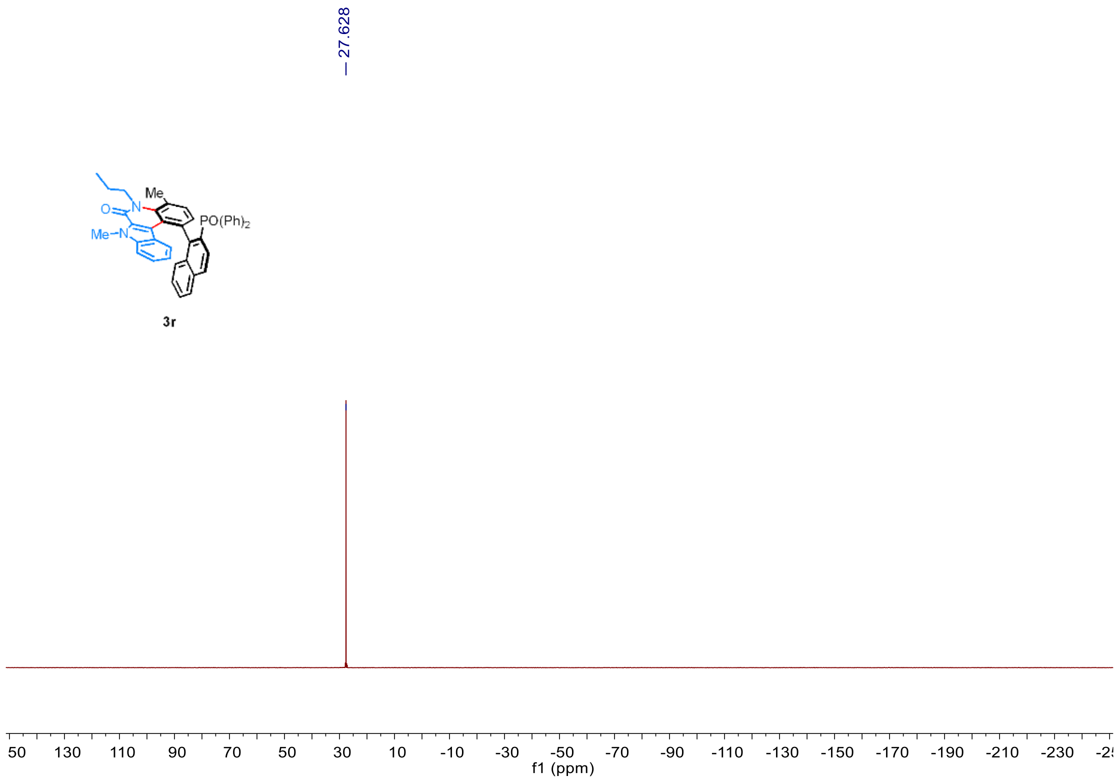
27.628
Me
O
N
Me
N
PO(Ph)2
3r
50 130 110 90 70 50 30 10 -10 -30 -50 -70 -90 -110 -130 -150 -170 -190 -210 -230
f1 (ppm)

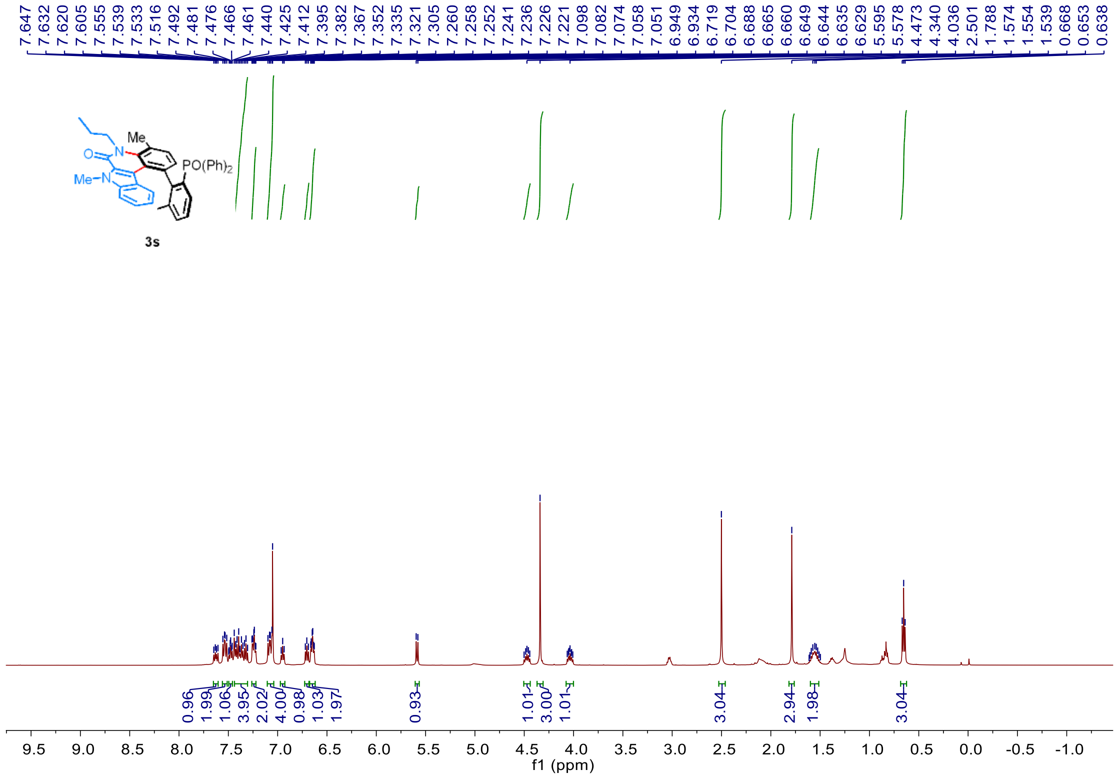
7.647 7.632 7.620 7.605 7.555 7.539 7.533 7.516 7.492 7.481 7.476 7.466 7.461 7.440 7.425 7.412 7.395 7.382 7.367 7.352 7.335 7.321 7.305 7.260 7.258 7.252 7.241 7.236 7.226 7.221 7.098 7.082 7.074 7.058 7.051 6.949 6.934 6.719 6.704 6.688 6.665 6.660 6.649 6.644 6.635 6.629 5.595 5.578 4.473 4.340 4.036 2.501 1.788 1.574 1.554 1.539 0.668 0.653 0.638
Me
O
N
Me
N
PO(Ph)2
3s
0.96 1.99 1.06 3.95 2.02 4.00 0.98 1.03 1.97 0.93 1.01 3.00 1.01 3.04 2.94 1.98 3.04
9.5 9.0 8.5 8.0 7.5 7.0 6.5 6.0 5.5 5.0 4.5 4.0 3.5 3.0 2.5 2.0 1.5 1.0 0.5 0.0 -0.5 -1.0
f1 (ppm)

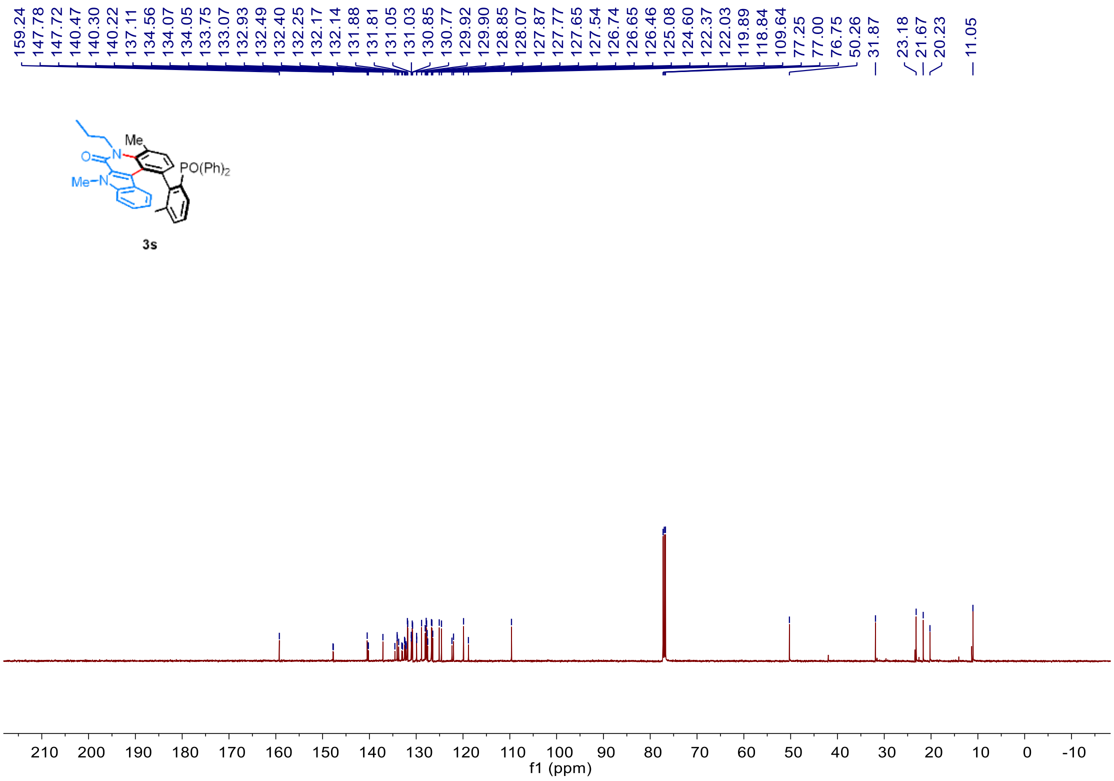
159.24
147.78
147.72
140.47
140.30
140.22
137.11
134.56
134.07
134.05
133.75
133.07
132.93
132.49
132.40
132.25
132.17
132.14
131.88
131.81
131.05
131.03
130.85
130.77
129.92
129.90
128.85
128.07
127.87
127.77
127.65
127.54
126.74
126.65
126.46
125.08
124.60
122.37
122.03
119.89
118.84
109.64
77.25
77.00
76.75
50.26
31.87
23.18
21.67
20.23
11.05
Me
PO(Ph)2
O
N
Me
N
3s
f1 (ppm)

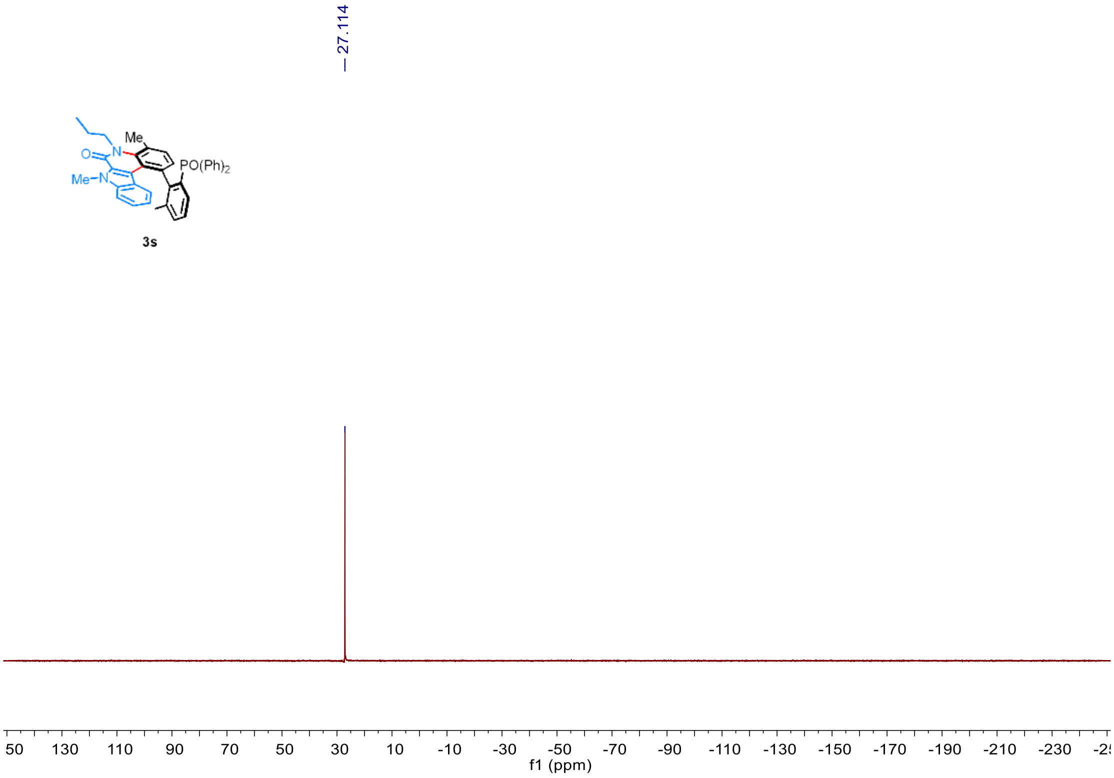
27.114
Me
PO(Ph)2
O
N
Me
N
3s
f1 (ppm)

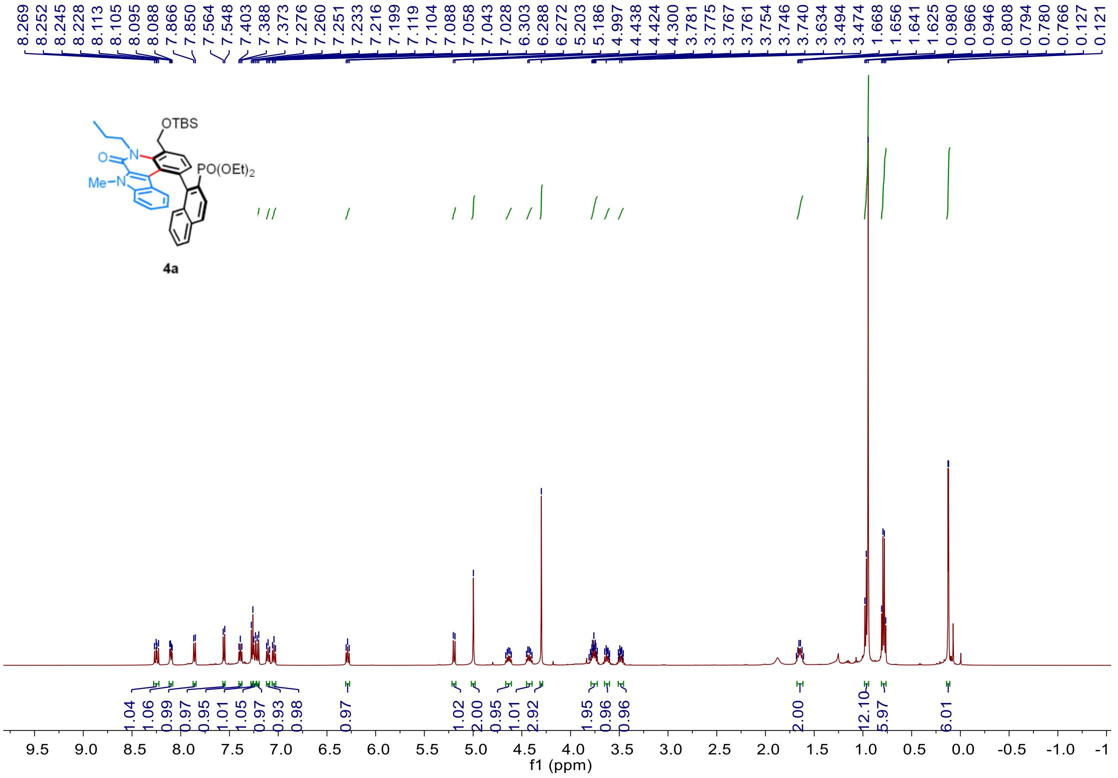
8.269 8.252 8.245 8.228 8.113 8.105 8.095 8.088 7.866 7.850 7.564 7.548 7.403 7.388 7.373 7.276 7.260 7.251 7.233 7.216 7.199 7.119 7.104 7.088 7.058 7.043 7.028 6.303 6.288 6.272 5.203 5.186 4.997 4.438 4.424 4.300 3.781 3.775 3.767 3.761 3.754 3.746 3.740 3.634 3.494 3.474 1.668 1.656 1.641 1.625 0.980 0.966 0.946 0.808 0.794 0.780 0.766 0.127 0.121
OTBS
PO(OEt)2
Me–N
4a
1.04 1.06 0.99 0.97 0.95 1.01 1.05 0.97 0.93 0.98 0.97 1.02 2.00 0.95 1.01 2.92 1.95 0.96 0.96 2.00 12.10 5.97 6.01
9.5 9.0 8.5 8.0 7.5 7.0 6.5 6.0 5.5 5.0 4.5 4.0 3.5 3.0 2.5 2.0 1.5 1.0 0.5 0.0 -0.5 -1.0
f1 (ppm)

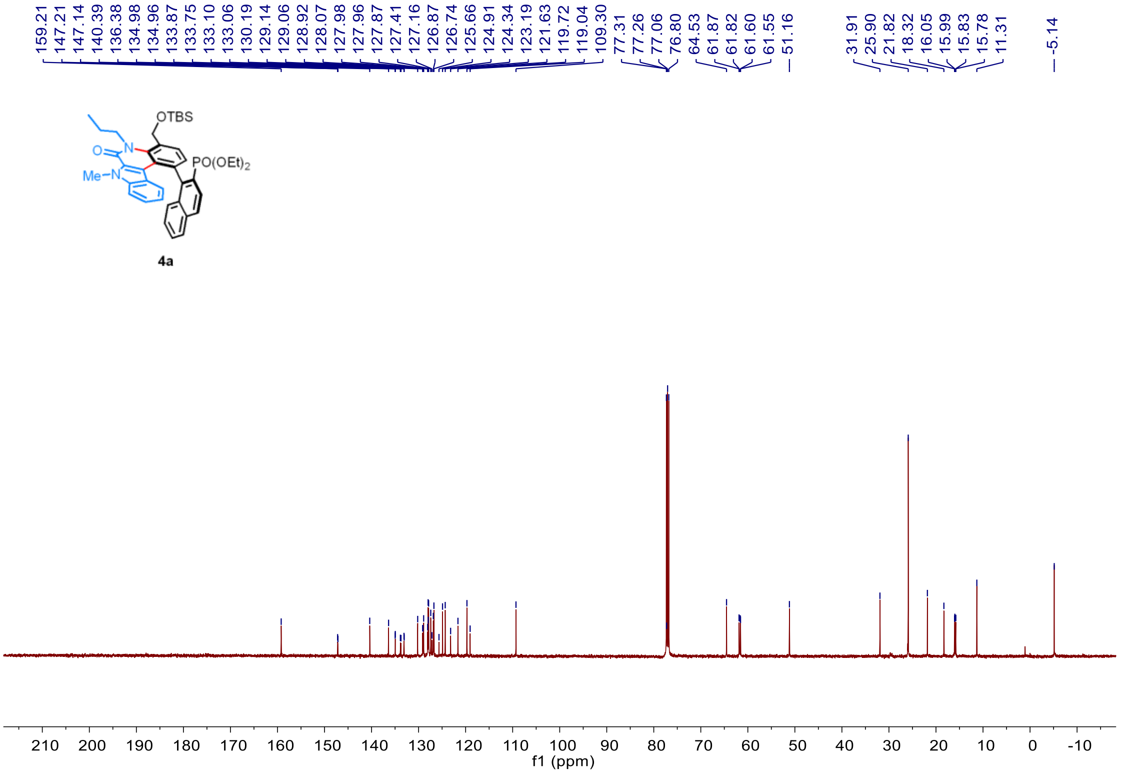
159.21 147.21 147.14 140.39 136.38 134.98 134.96 133.87 133.75 133.10 133.06 130.19 129.14 129.06 128.92 128.07 127.98 127.96 127.87 127.41 127.16 126.87 126.74 125.66 124.91 124.34 123.19 121.63 119.72 119.04 109.30 77.31 77.26 77.06 76.80 64.53 61.87 61.82 61.60 61.55 51.16 31.91 25.90 21.82 18.32 16.05 15.99 15.83 15.78 11.31 -5.14
OTBS
PO(OEt)2
Me–N
4a
210 200 190 180 170 160 150 140 130 120 110 100 90 80 70 60 50 40 30 20 10 0 -10
f1 (ppm)

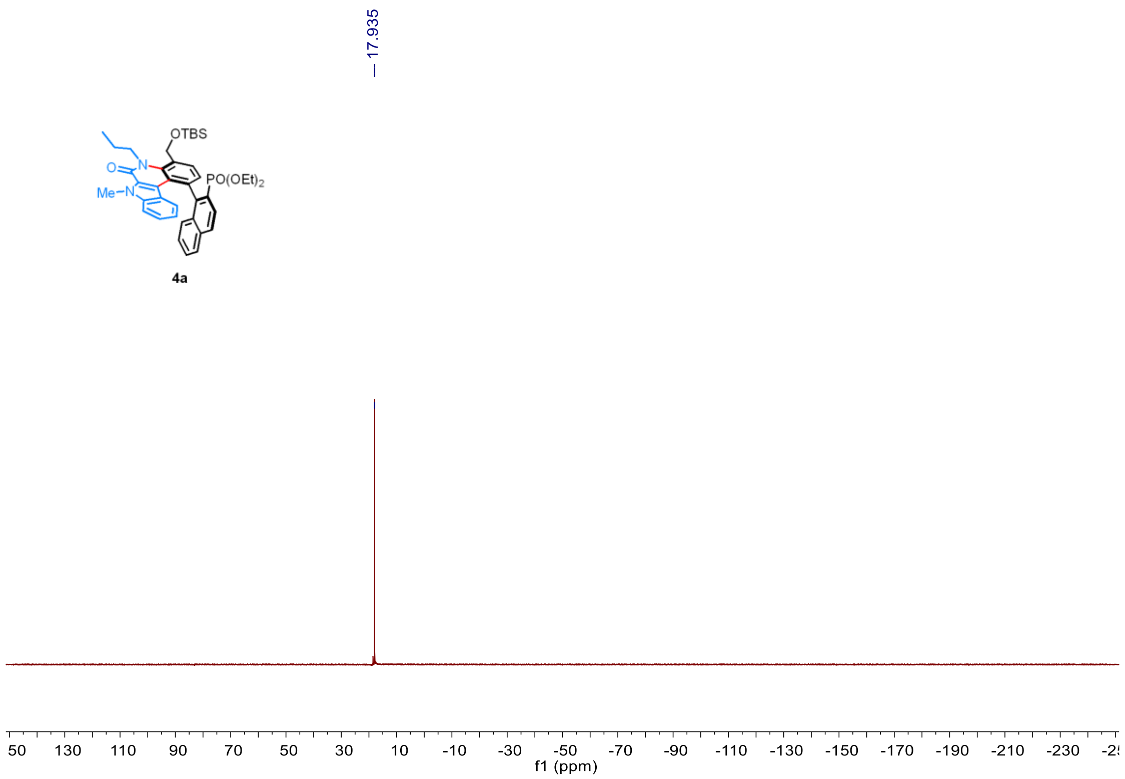

17.935
OTBS
O
N
Me
PO(OEt)2
4a
50 130 110 90 70 50 30 10 -10 -30 -50 -70 -90 -110 -130 -150 -170 -190 -210 -230
f1 (ppm)

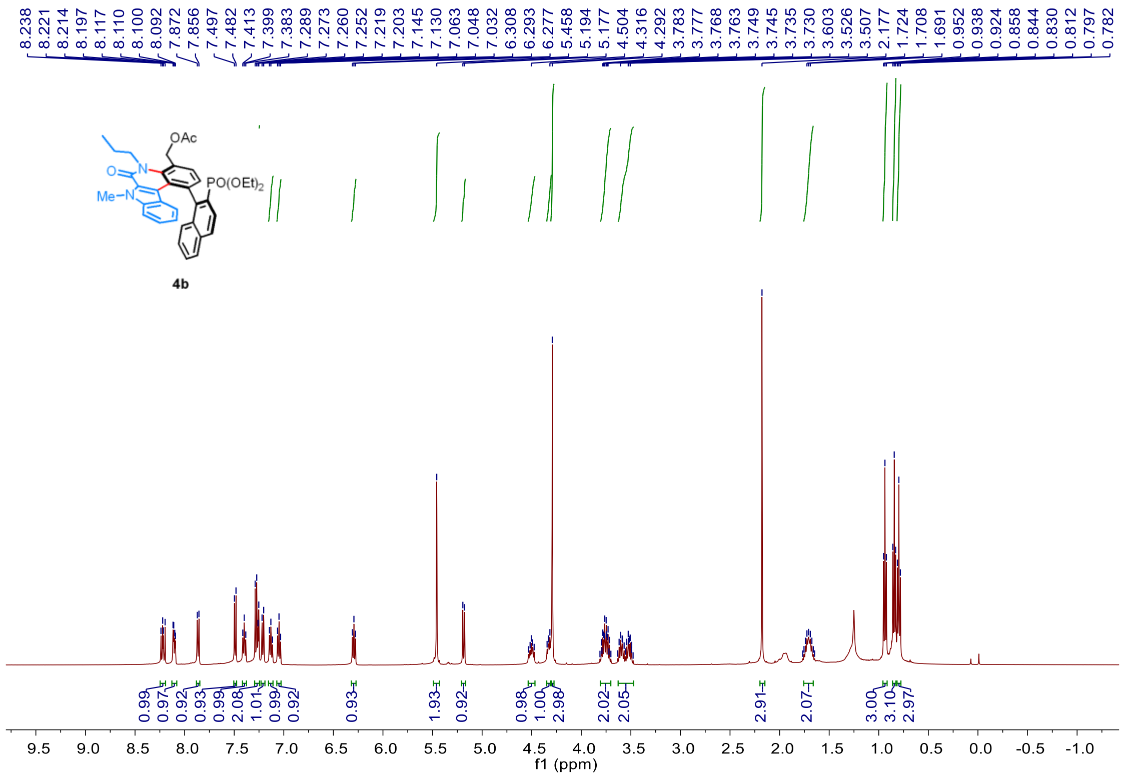

8.238 8.221 8.214 8.197 8.117 8.110 8.100 8.092 7.872 7.856 7.497 7.482 7.413 7.399 7.383 7.289 7.273 7.260 7.252 7.219 7.203 7.145 7.130 7.063 7.048 7.032 6.308 6.293 6.277 5.458 5.194 5.177 4.504 4.316 4.292 3.783 3.777 3.768 3.763 3.749 3.745 3.735 3.730 3.603 3.526 3.507 2.177 1.724 1.708 1.691 0.952 0.938 0.924 0.858 0.844 0.830 0.812 0.797 0.782
OAc
O
N
Me
PO(OEt)2
4b
0.99 0.97 0.92 0.93 0.99 2.08 1.01 0.99 0.92 0.93 1.93 0.92 0.98 1.00 2.98 2.02 2.05 2.91 2.07 3.00 3.10 2.97
9.5 9.0 8.5 8.0 7.5 7.0 6.5 6.0 5.5 5.0 4.5 4.0 3.5 3.0 2.5 2.0 1.5 1.0 0.5 0.0 -0.5 -1.0
f1 (ppm)

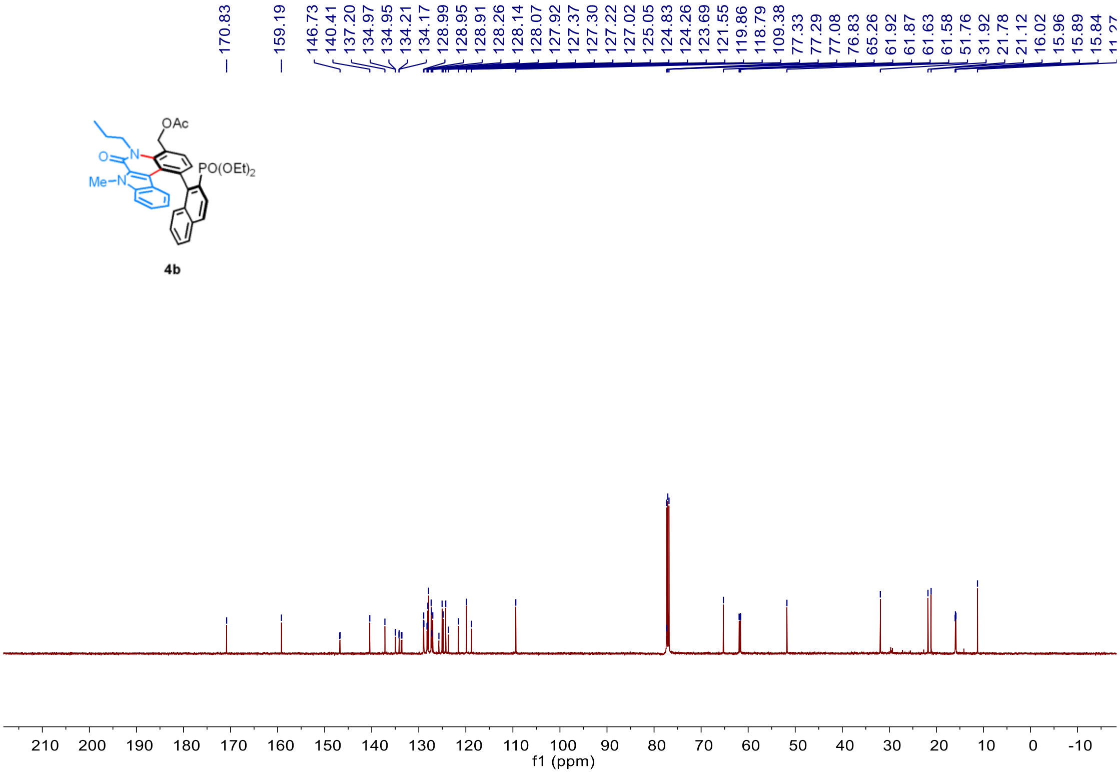
170.83
159.19
146.73
140.41
137.20
134.97
134.95
134.21
134.17
128.99
128.95
128.91
128.26
128.14
128.07
127.92
127.37
127.30
127.22
127.02
125.05
124.83
124.26
123.69
121.55
119.86
118.79
109.38
77.33
77.29
77.08
76.83
65.26
61.92
61.87
61.63
61.58
51.76
31.92
21.78
21.12
16.02
15.96
15.89
15.84
OAc
O
N
Me–N
PO(OEt)2
4b
210 200 190 180 170 160 150 140 130 120 110 100 90 80 70 60 50 40 30 20 10 0 -10
f1 (ppm)

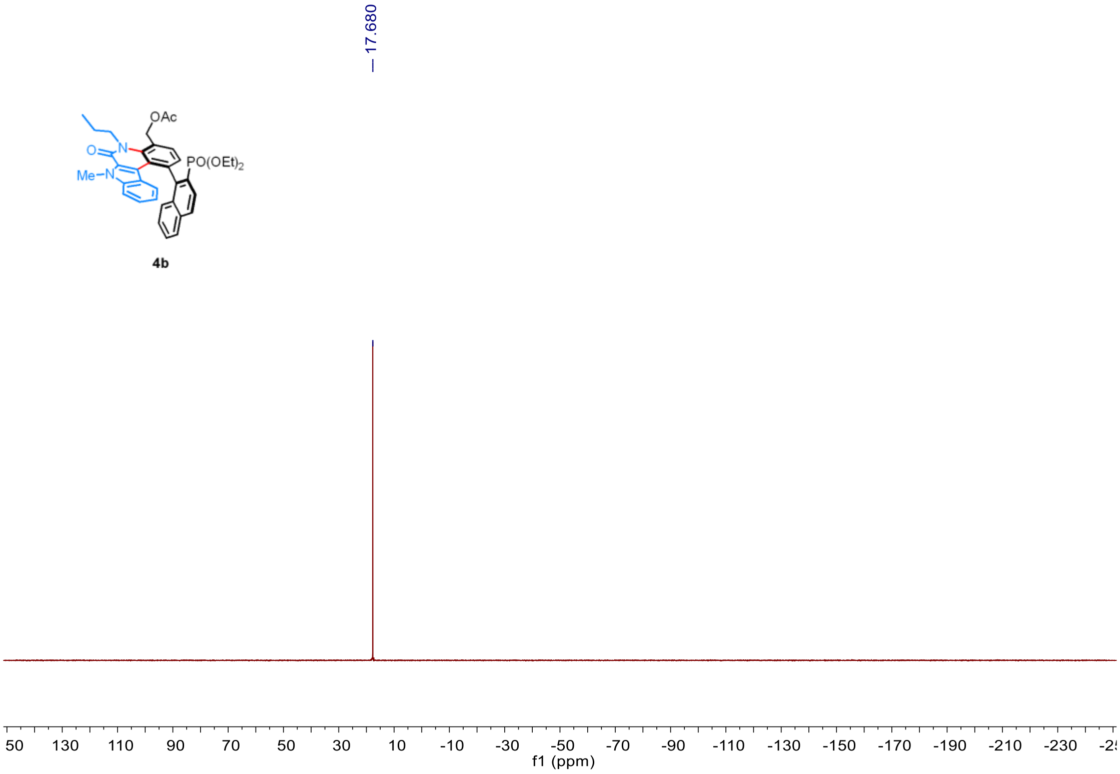
17.680
OAc
O
N
Me–N
PO(OEt)2
4b
50 130 110 90 70 50 30 10 -10 -30 -50 -70 -90 -110 -130 -150 -170 -190 -210 -230
f1 (ppm)

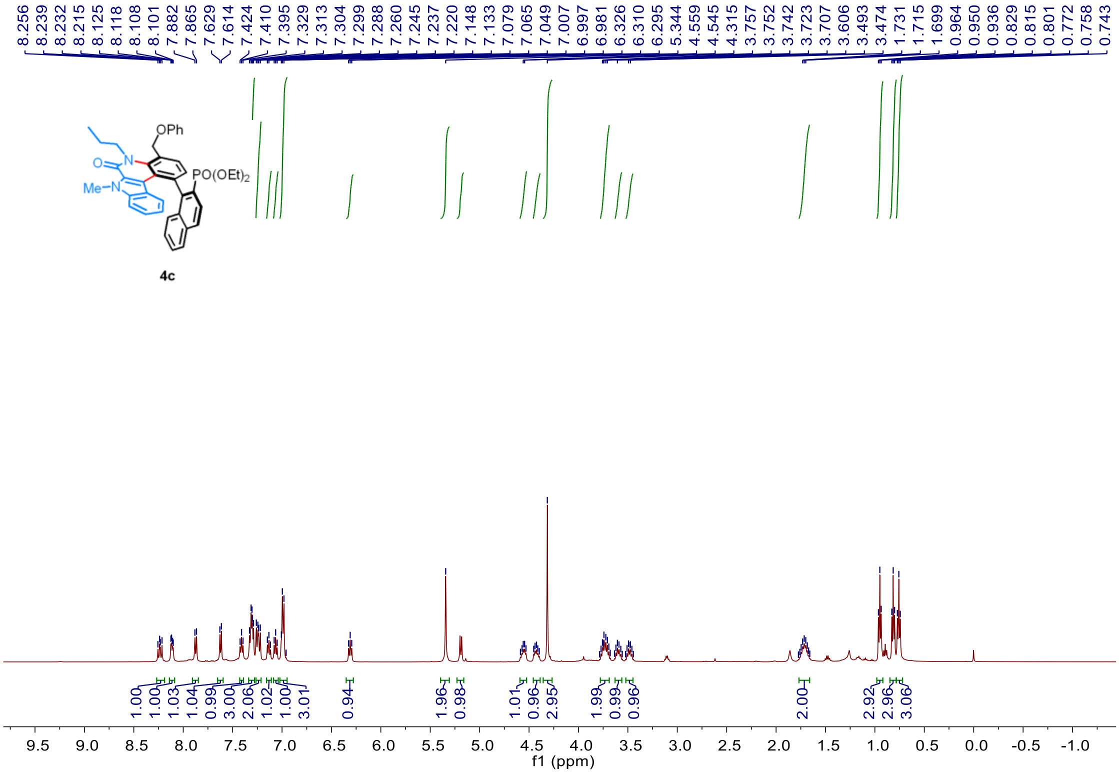
8.256
8.239
8.232
8.215
8.125
8.118
8.108
8.101
7.882
7.865
7.629
7.614
7.424
7.410
7.395
7.329
7.313
7.304
7.299
7.288
7.260
7.245
7.237
7.220
7.148
7.133
7.079
7.065
7.049
7.007
6.997
6.981
6.326
6.310
6.295
5.344
4.559
4.545
4.315
3.757
3.752
3.742
3.723
3.707
3.606
3.493
3.474
1.731
1.715
1.699
0.964
0.950
0.936
0.829
0.815
0.801
0.772
0.758
0.743
OPh
O
N
PO(OEt)2
Me–N
4c
1.00
1.00
1.03
1.04
0.99
3.00
2.06
1.02
1.00
3.01
0.94
1.96
0.98
1.01
0.96
2.95
1.99
0.99
0.96
2.00
2.92
2.96
3.06
9.5 9.0 8.5 8.0 7.5 7.0 6.5 6.0 5.5 5.0 4.5 4.0 3.5 3.0 2.5 2.0 1.5 1.0 0.5 0.0 -0.5 -1.0
f1 (ppm)

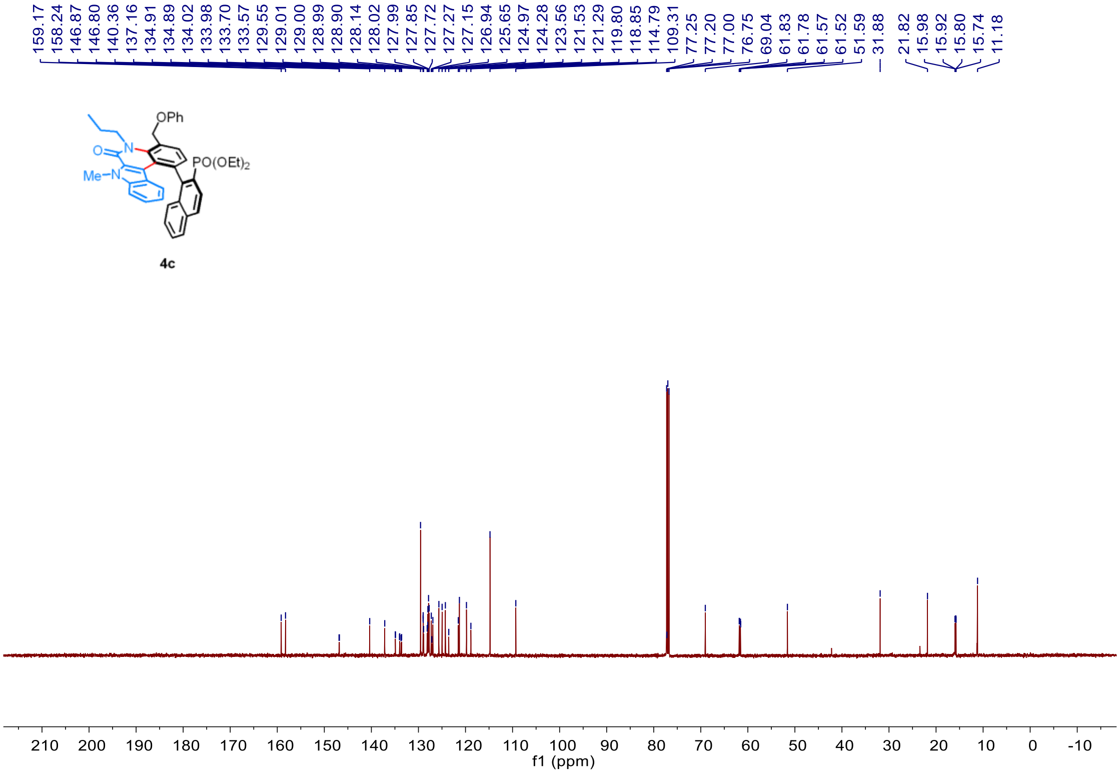
159.17
158.24
146.87
140.80
140.36
137.16
134.91
134.89
134.02
133.98
133.70
133.57
129.55
129.01
129.00
128.99
128.90
128.14
128.02
127.99
127.85
127.72
127.27
127.15
126.94
125.65
124.97
124.28
123.56
121.53
121.29
119.80
118.85
114.79
109.31
77.25
77.20
77.00
76.75
69.04
61.83
61.78
61.57
61.52
51.59
31.88
21.82
15.98
15.92
15.80
15.74
11.18
OPh
O
N
PO(OEt)2
Me–N
4c
210 200 190 180 170 160 150 140 130 120 110 100 90 80 70 60 50 40 30 20 10 0 -10
f1 (ppm)

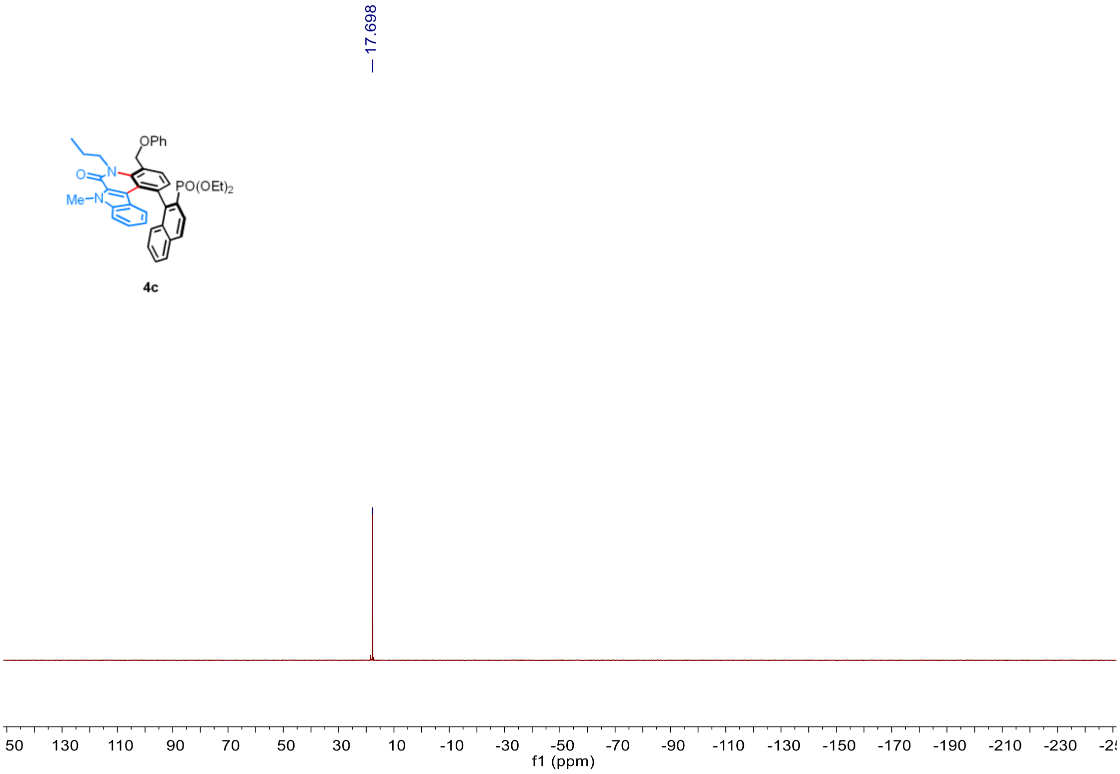
17.698
OPh
PO(OEt)2
O
N
Me
4c
f1 (ppm)
50 130 110 90 70 50 30 10 -10 -30 -50 -70 -90 -110 -130 -150 -170 -190 -210 -230

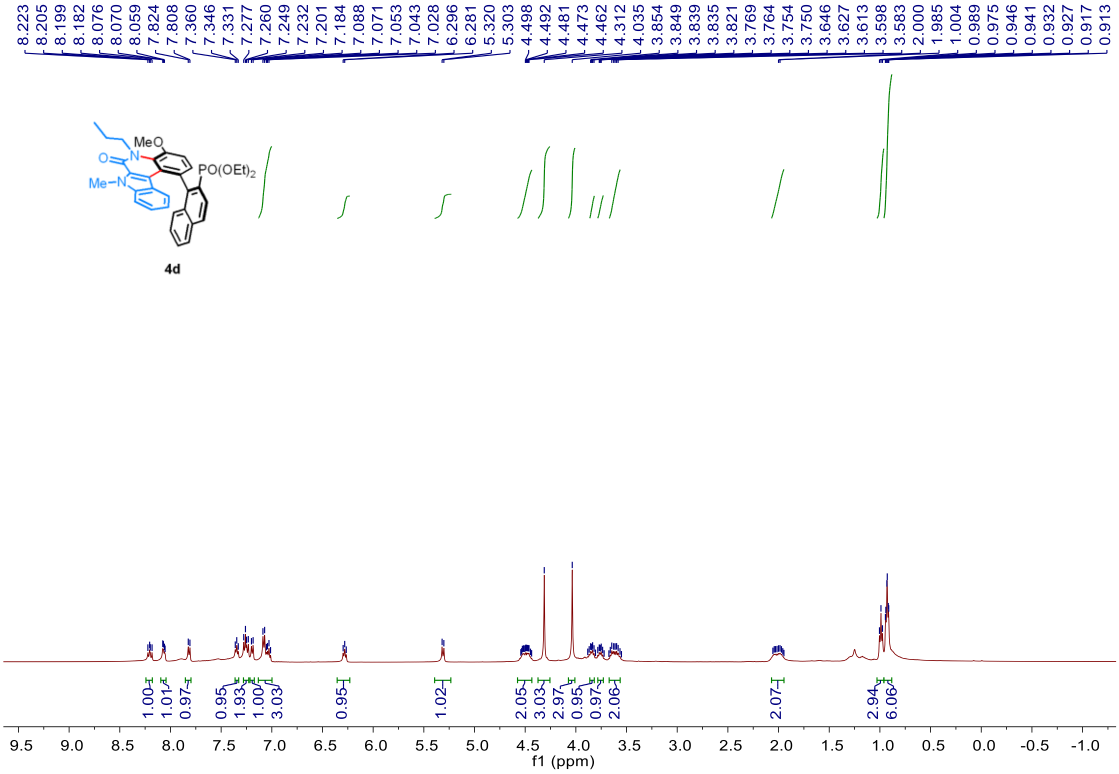
8.223 8.205 8.199 8.182 8.076 8.070 8.059 7.824 7.808 7.360 7.346 7.331 7.277 7.260 7.249 7.232 7.201 7.184 7.088 7.071 7.053 7.043 7.028 6.296 6.281 5.320 5.303 4.498 4.492 4.481 4.473 4.462 4.312 4.035 3.854 3.849 3.839 3.835 3.821 3.769 3.764 3.754 3.750 3.646 3.627 3.613 3.598 3.583 2.000 1.985 1.004 0.989 0.975 0.946 0.941 0.932 0.927 0.917 0.913
MeO
PO(OEt)2
O
N
Me
4d
1.00 1.01 0.97 0.95 1.93 1.00 3.03 0.95 1.02 2.05 3.03 2.97 0.95 0.97 2.06 2.07 2.94 6.06
9.5 9.0 8.5 8.0 7.5 7.0 6.5 6.0 5.5 5.0 4.5 4.0 3.5 3.0 2.5 2.0 1.5 1.0 0.5 0.0 -0.5 -1.0
f1 (ppm)

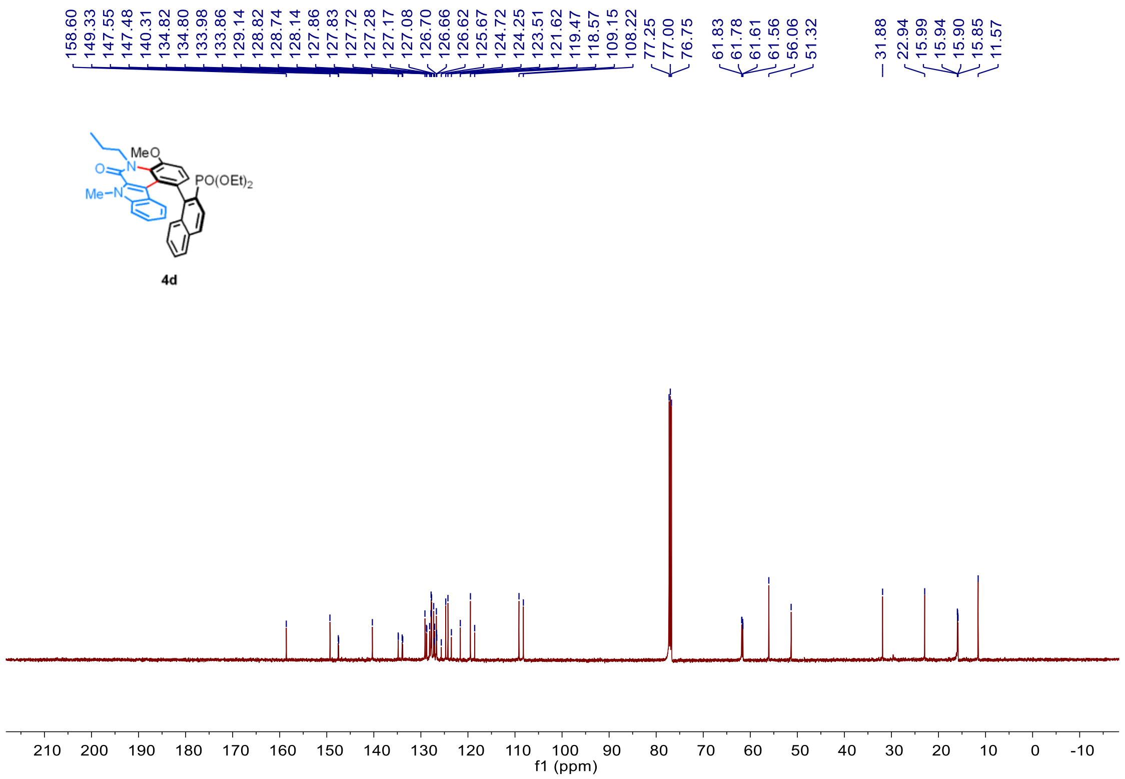
158.60
149.33
147.55
147.48
140.31
134.82
134.80
133.98
133.86
129.14
128.82
128.74
128.14
127.86
127.83
127.72
127.28
127.17
127.08
126.70
126.66
126.62
125.67
124.72
124.25
123.51
121.62
119.47
118.57
109.15
108.22
77.25
77.00
76.75
61.83
61.78
61.61
61.56
56.06
51.32
31.88
22.94
15.99
15.94
15.90
15.85
11.57
MeO
O
N
Me
N
PO(OEt)2
4d
f1 (ppm)

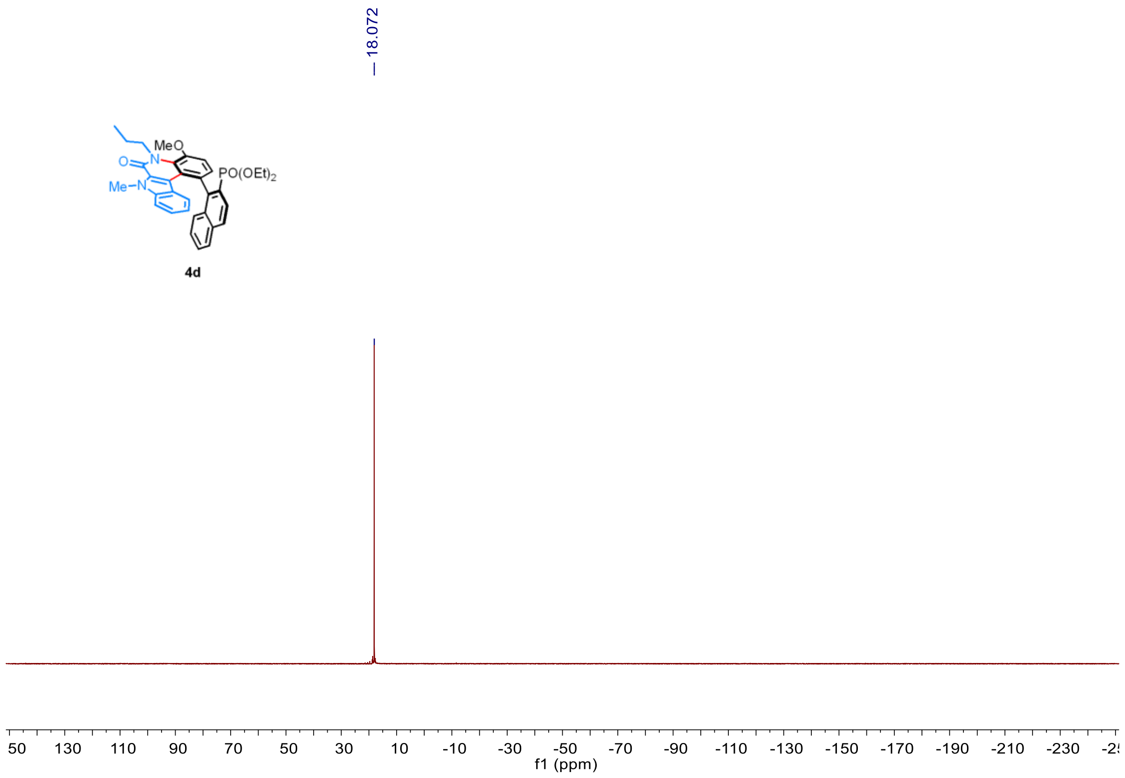
18.072
MeO
O
N
Me
N
PO(OEt)2
4d
f1 (ppm)

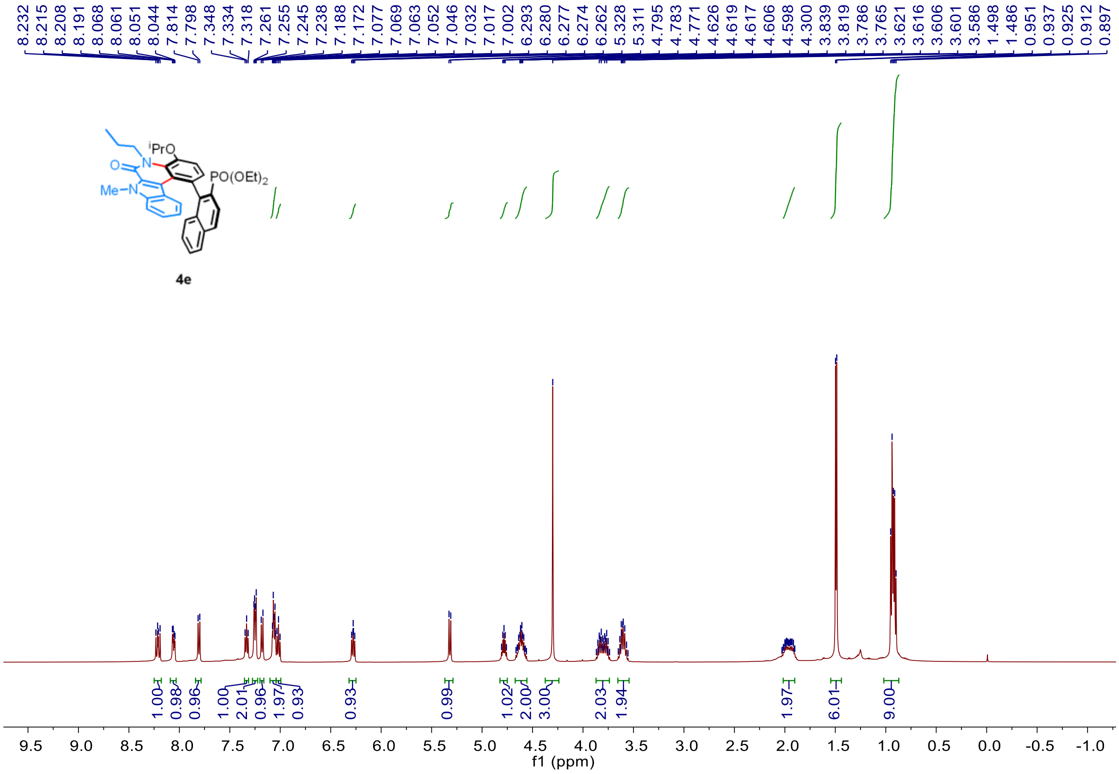
8.232
8.215
8.208
8.191
8.068
8.061
8.051
8.044
7.814
7.798
7.348
7.334
7.318
7.261
7.255
7.245
7.238
7.188
7.172
7.077
7.069
7.063
7.052
7.046
7.032
7.017
7.002
6.293
6.280
6.277
6.274
6.262
5.328
5.311
4.795
4.783
4.771
4.626
4.619
4.617
4.606
4.598
4.300
3.839
3.819
3.786
3.765
3.621
3.616
3.606
3.601
3.586
1.498
1.486
0.951
0.937
0.925
0.912
0.897
iPrO
N
O
Me–N
PO(OEt)2
4e
1.00
0.98
0.96
1.00
2.01
0.96
1.97
0.93
0.93
0.99
1.02
2.00
3.00
2.03
1.94
1.97
6.01
9.00
9.5
9.0
8.5
8.0
7.5
7.0
6.5
6.0
5.5
5.0
4.5
4.0
3.5
3.0
2.5
2.0
1.5
1.0
0.5
0.0
-0.5
-1.0
f1 (ppm)

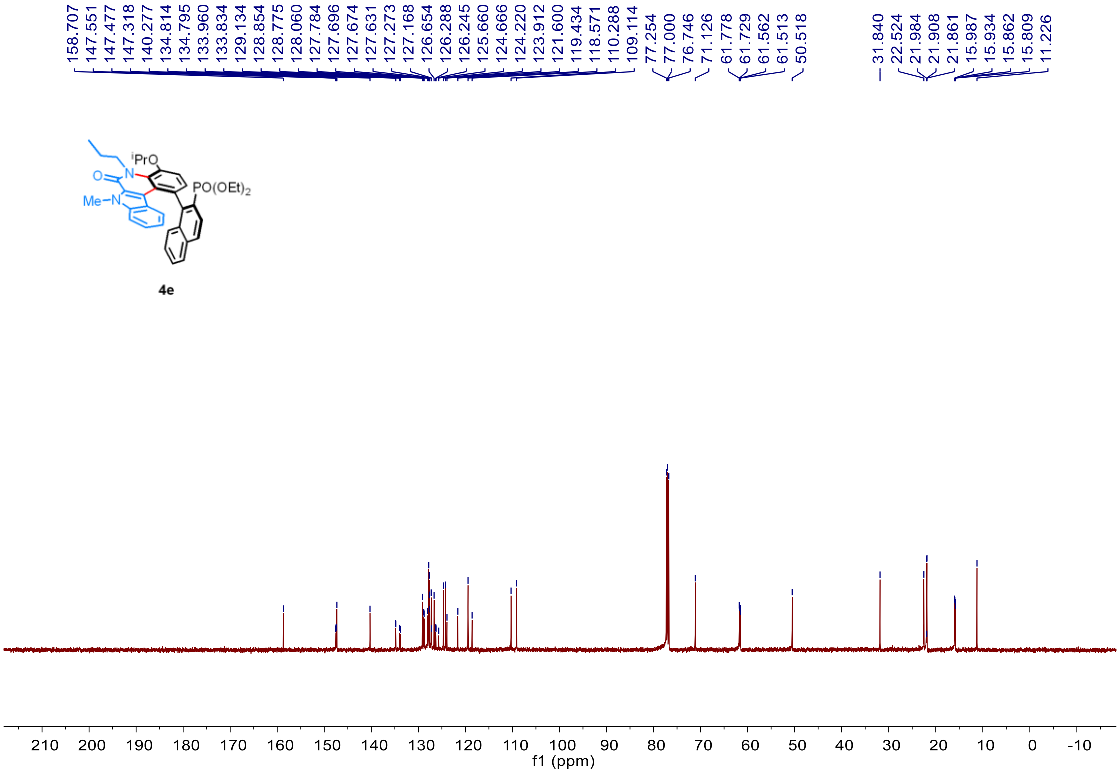
158.707
147.551
147.477
147.318
140.277
134.814
134.795
133.960
133.834
129.134
128.854
128.775
128.060
127.784
127.696
127.674
127.631
127.273
127.168
126.654
126.288
126.245
125.660
124.666
124.220
123.912
121.600
119.434
118.571
110.288
109.114
77.254
77.000
76.746
71.126
61.778
61.729
61.562
61.513
50.518
31.840
22.524
21.984
21.908
21.861
15.987
15.934
15.862
15.809
11.226
iPrO
N
O
Me–N
PO(OEt)2
4e
210
200
190
180
170
160
150
140
130
120
110
100
90
80
70
60
50
40
30
20
10
0
-10
f1 (ppm)

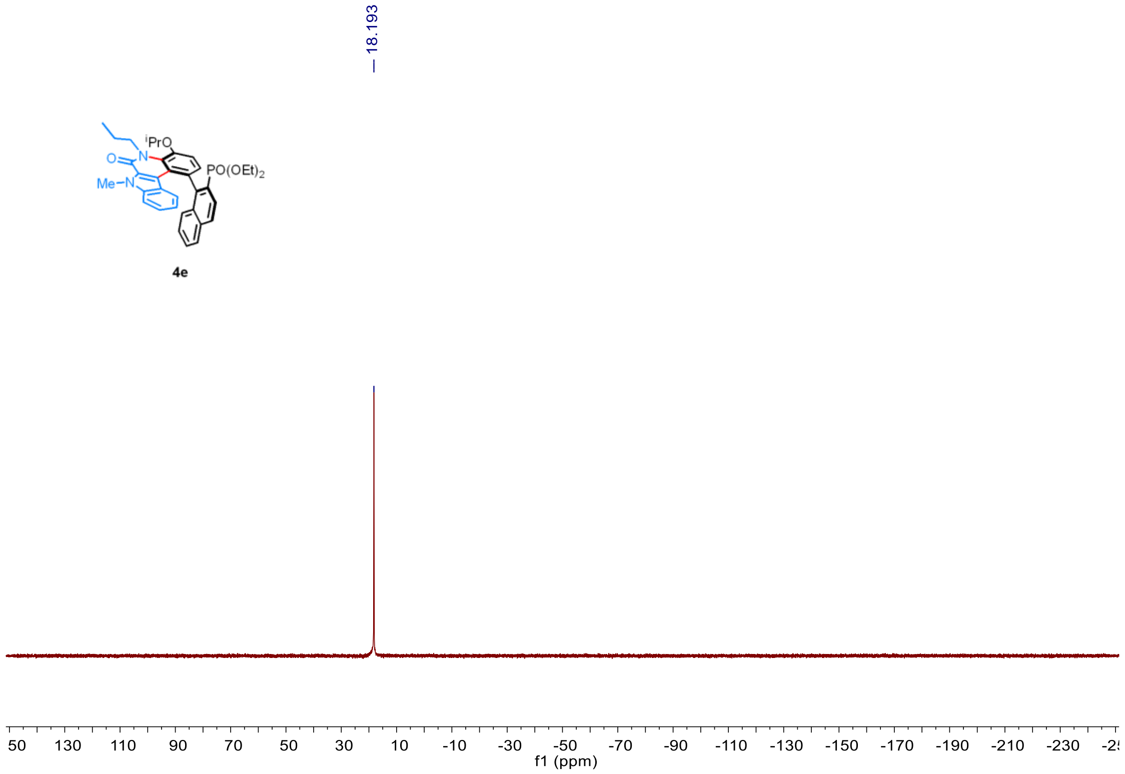

18.193
ⁱPrO
PO(OEt)2
Me–N
O
N
4e
50 130 110 90 70 50 30 10 -10 -30 -50 -70 -90 -110 -130 -150 -170 -190 -210 -230
f1 (ppm)

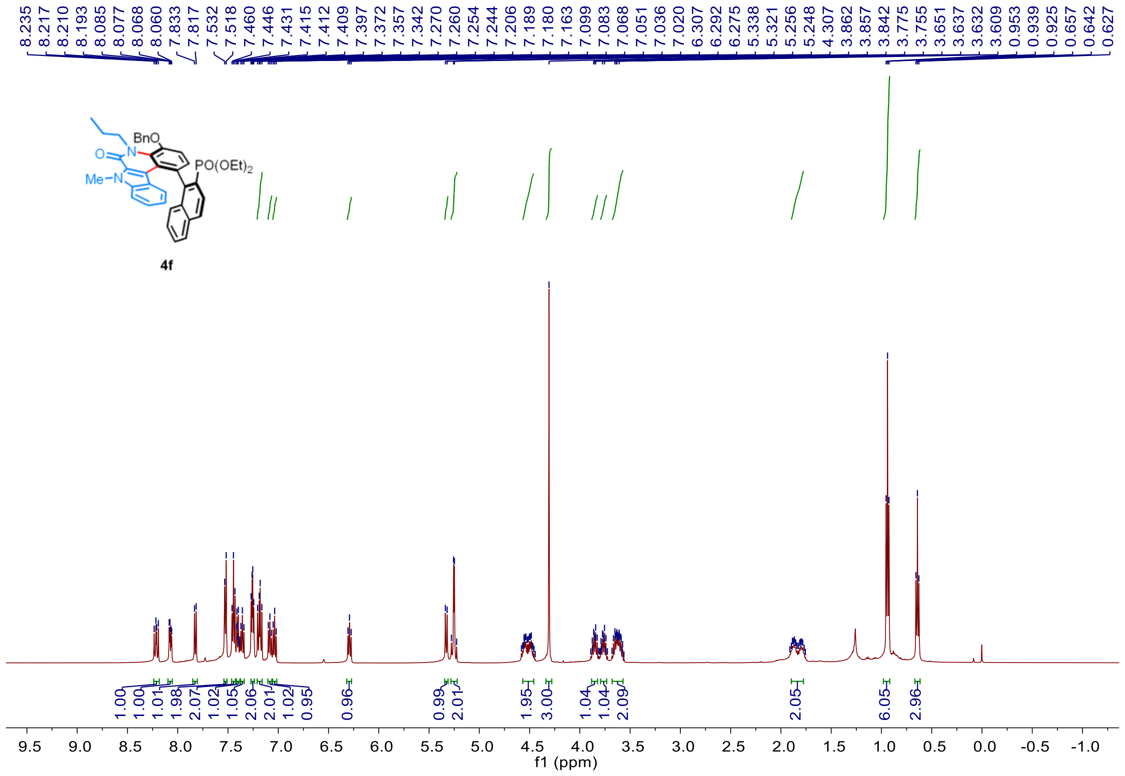

8.235 8.217 8.210 8.193 8.085 8.077 8.068 8.060 7.833 7.817 7.532 7.518 7.460 7.446 7.431 7.415 7.412 7.409 7.397 7.372 7.357 7.342 7.270 7.260 7.254 7.244 7.206 7.189 7.180 7.163 7.099 7.083 7.068 7.051 7.036 7.020 6.307 6.292 6.275 5.338 5.321 5.256 5.248 4.307 3.862 3.857 3.842 3.775 3.755 3.651 3.637 3.632 3.609 0.953 0.939 0.925 0.657 0.642 0.627
BnO
PO(OEt)2
Me–N
O
N
4f
1.00 1.00 1.01 1.98 2.07 1.02 1.05 2.06 2.01 1.02 0.95 0.96 0.99 2.01 1.95 3.00 1.04 1.04 2.09 2.05 6.05 2.96
9.5 9.0 8.5 8.0 7.5 7.0 6.5 6.0 5.5 5.0 4.5 4.0 3.5 3.0 2.5 2.0 1.5 1.0 0.5 0.0 -0.5 -1.0
f1 (ppm)

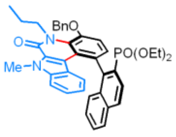
BnO
N
O
Me–N
PO(OEt)2

4f

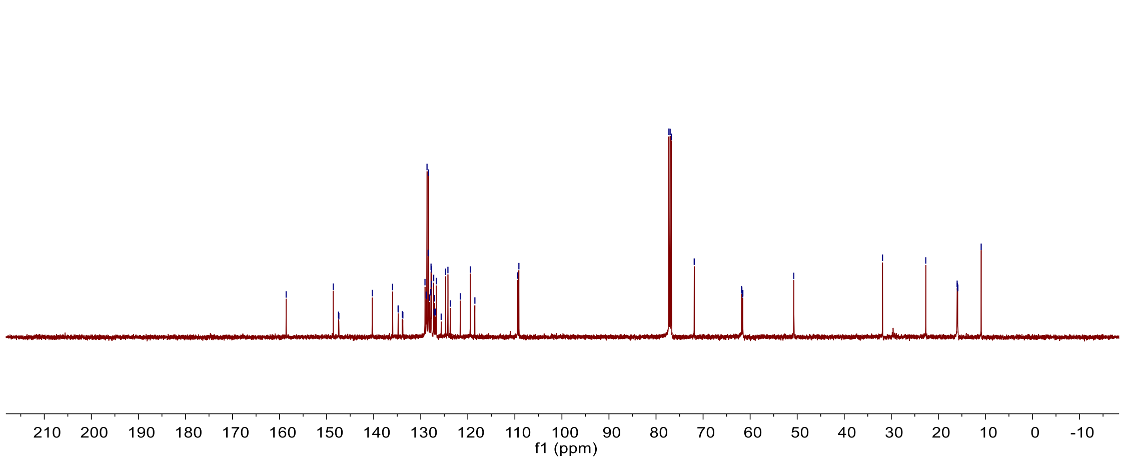
158.60
148.59
147.48
147.40
140.29
135.98
134.81
134.79
133.93
133.80
129.10
128.83
128.75
128.66
128.41
128.32
128.12
127.86
127.81
127.74
127.72
127.25
127.17
127.05
126.93
126.89
126.69
125.66
124.71
124.22
123.72
121.60
119.47
118.49
109.40
109.14
77.25
77.00
76.75
71.88
61.81
61.76
61.60
61.55
50.72
31.86
22.67
16.01
15.96
15.90
15.85
10.90
210 200 190 180 170 160 150 140 130 120 110 100 90 80 70 60 50 40 30 20 10 0 -10
f1 (ppm)

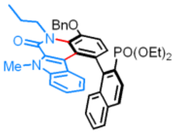
BnO
N
O
Me–N
PO(OEt)2

4f

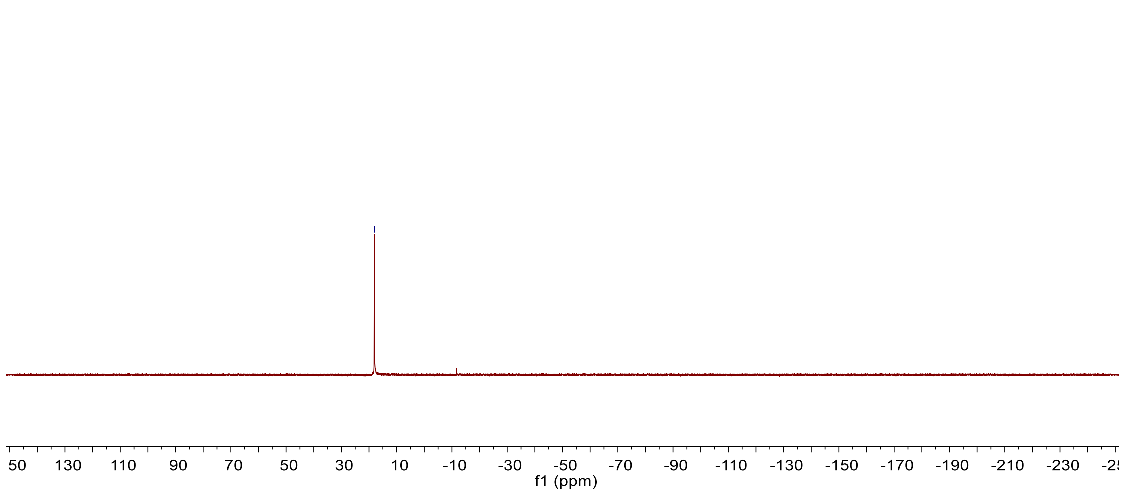
18.035
130 110 90 70 50 30 10 -10 -30 -50 -70 -90 -110 -130 -150 -170 -190 -210 -230
f1 (ppm)

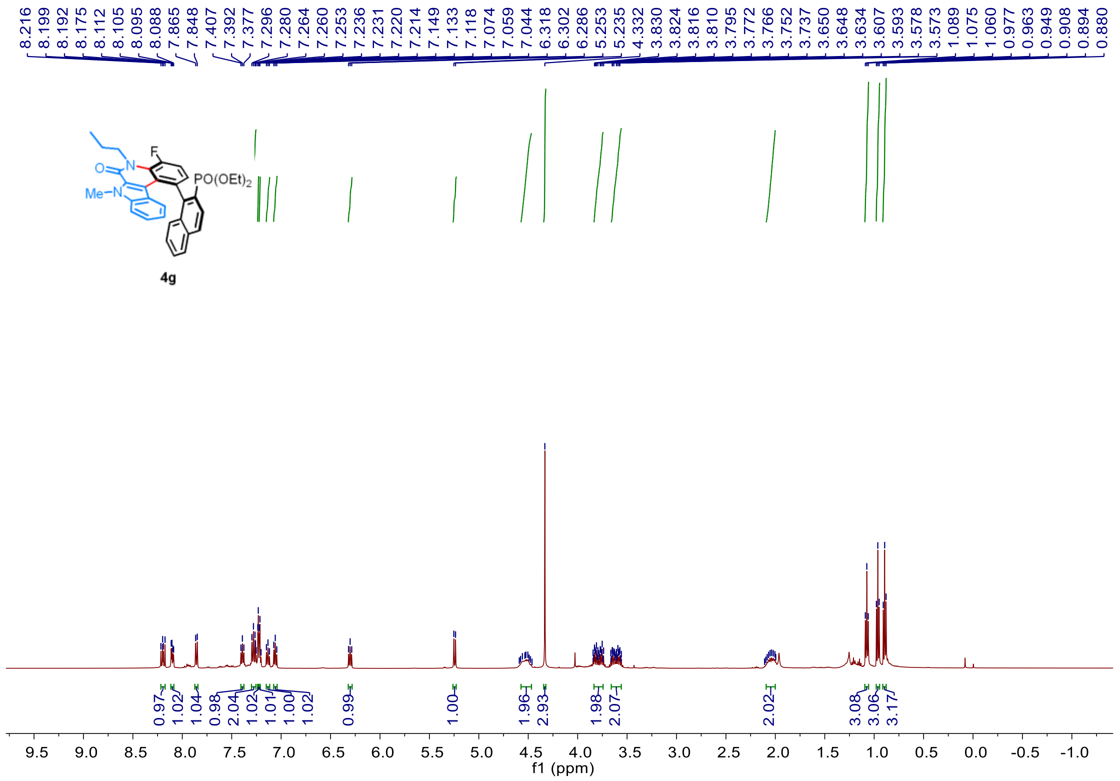
8.216
8.199
8.192
8.175
8.112
8.105
8.095
8.088
7.865
7.848
7.407
7.392
7.377
7.296
7.280
7.264
7.260
7.253
7.236
7.231
7.220
7.214
7.149
7.133
7.118
7.074
7.059
7.044
6.318
6.302
6.286
5.253
5.235
4.332
3.830
3.824
3.816
3.810
3.795
3.772
3.766
3.752
3.737
3.650
3.648
3.634
3.607
3.593
3.578
3.573
1.089
1.075
1.060
0.977
0.963
0.949
0.908
0.894
0.880
F
O
N
PO(OEt)$_2$
Me
4g
0.97
1.02
1.04
0.98
2.04
1.02
1.01
1.00
1.02
0.99
1.00
1.96
2.93
1.98
2.07
2.02
3.08
3.06
3.17
9.5 9.0 8.5 8.0 7.5 7.0 6.5 6.0 5.5 5.0 4.5 4.0 3.5 3.0 2.5 2.0 1.5 1.0 0.5 0.0 -0.5 -1.0
f1 (ppm)

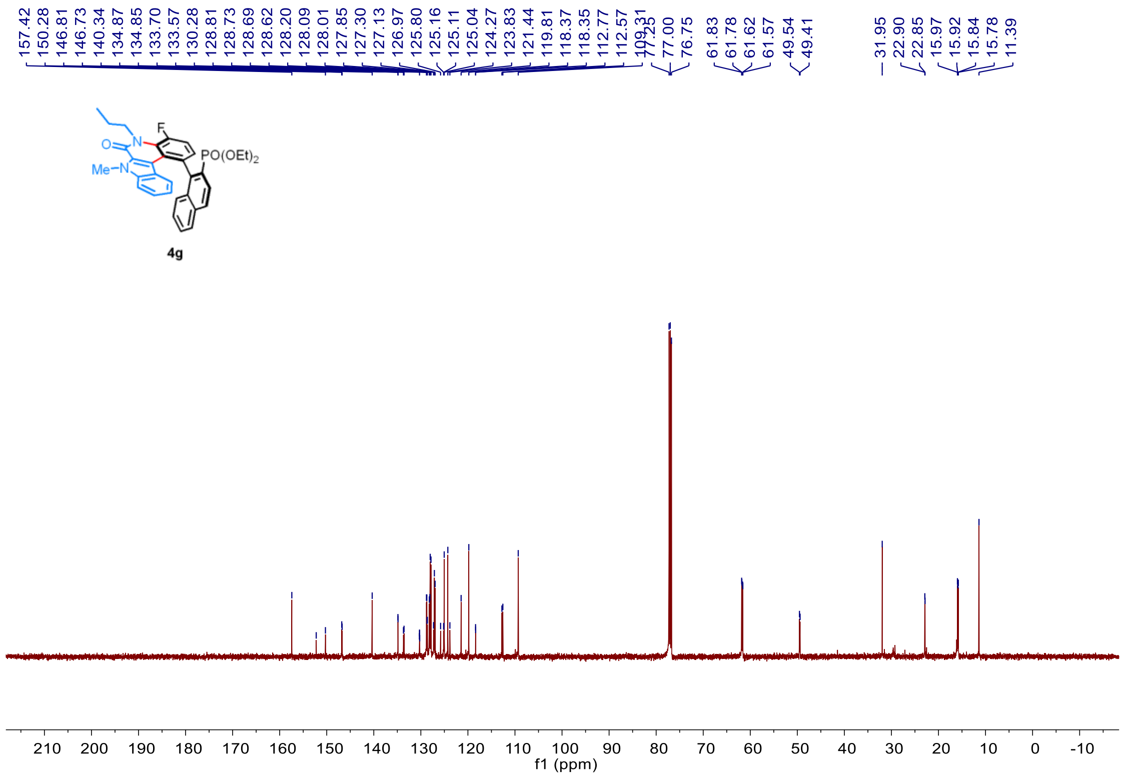
157.42
150.28
146.81
146.73
140.34
134.87
134.85
133.70
133.57
130.28
128.81
128.73
128.69
128.62
128.20
128.09
128.01
127.85
127.30
127.13
126.97
125.80
125.16
125.11
125.04
124.27
123.83
121.44
119.81
118.37
118.35
112.77
112.57
109.31
77.25
77.00
76.75
61.83
61.78
61.62
61.57
49.54
49.41
31.95
22.90
22.85
15.97
15.92
15.84
15.78
11.39
F
O
N
PO(OEt)$_2$
Me
4g
210 200 190 180 170 160 150 140 130 120 110 100 90 80 70 60 50 40 30 20 10 0 -10
f1 (ppm)

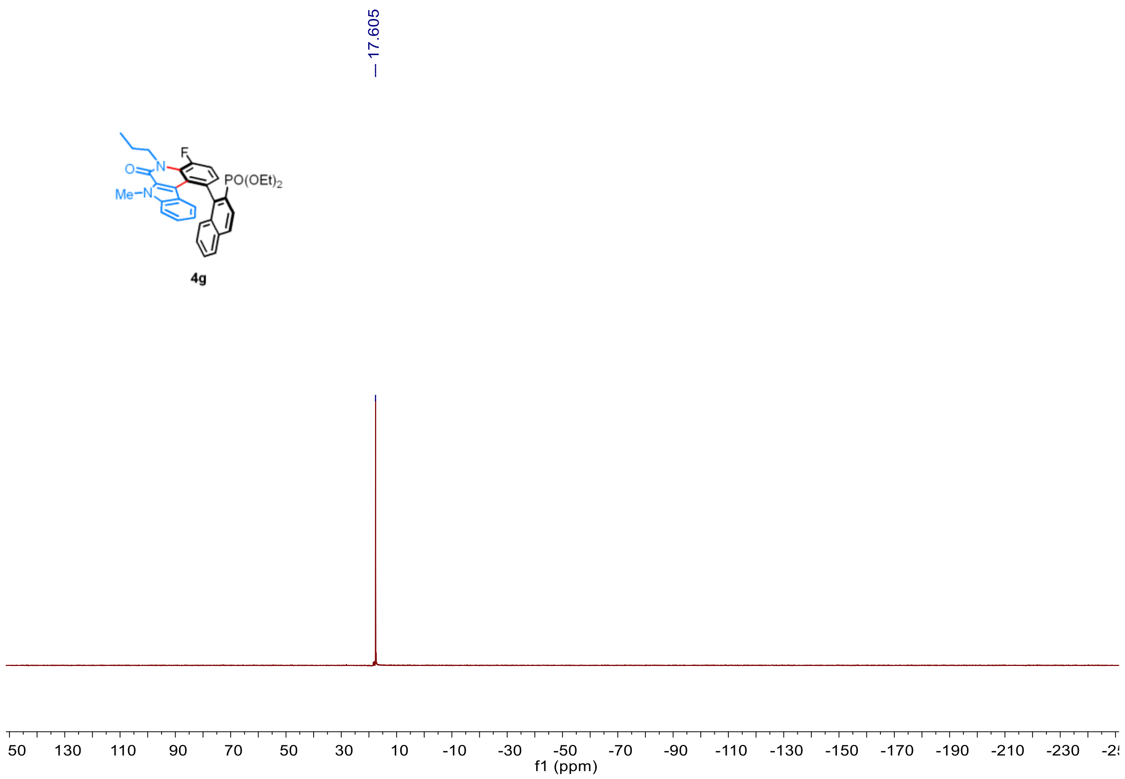
17.605
F
N
O
Me–N
PO(OEt)2
4g
50 130 110 90 70 50 30 10 -10 -30 -50 -70 -90 -110 -130 -150 -170 -190 -210 -230
f1 (ppm)

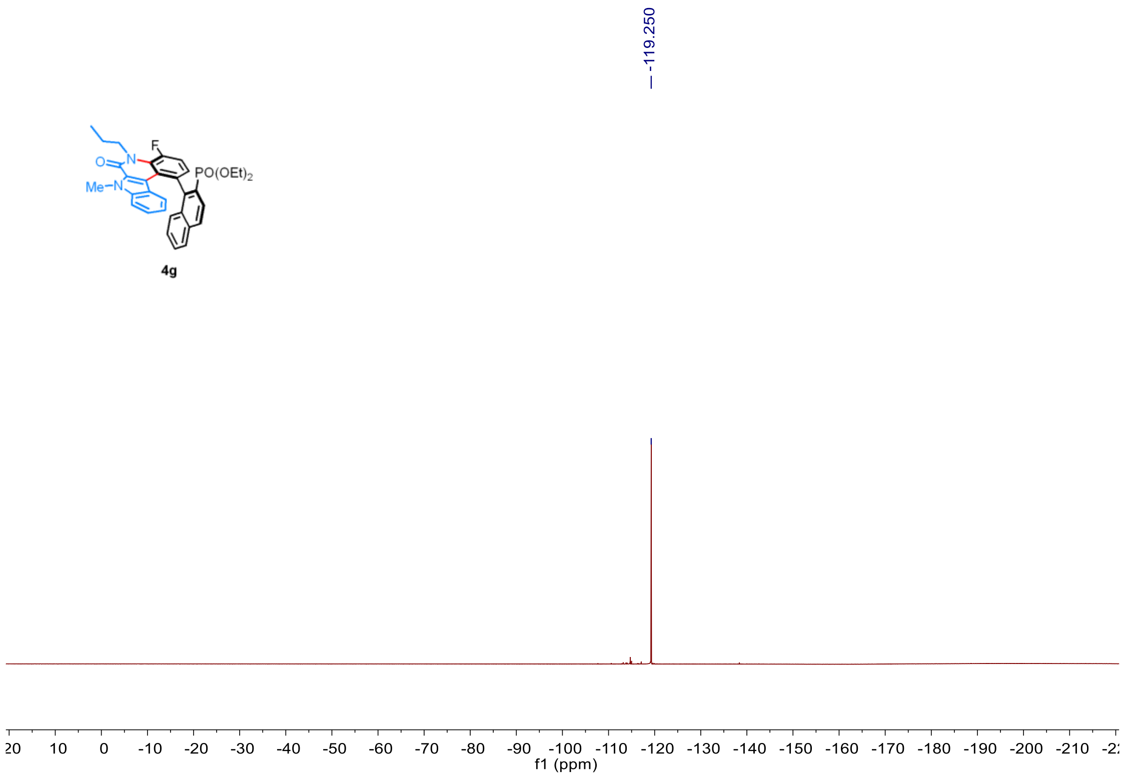
-119.250
F
N
O
Me–N
PO(OEt)2
4g
20 10 0 -10 -20 -30 -40 -50 -60 -70 -80 -90 -100 -110 -120 -130 -140 -150 -160 -170 -180 -190 -200 -210
f1 (ppm)

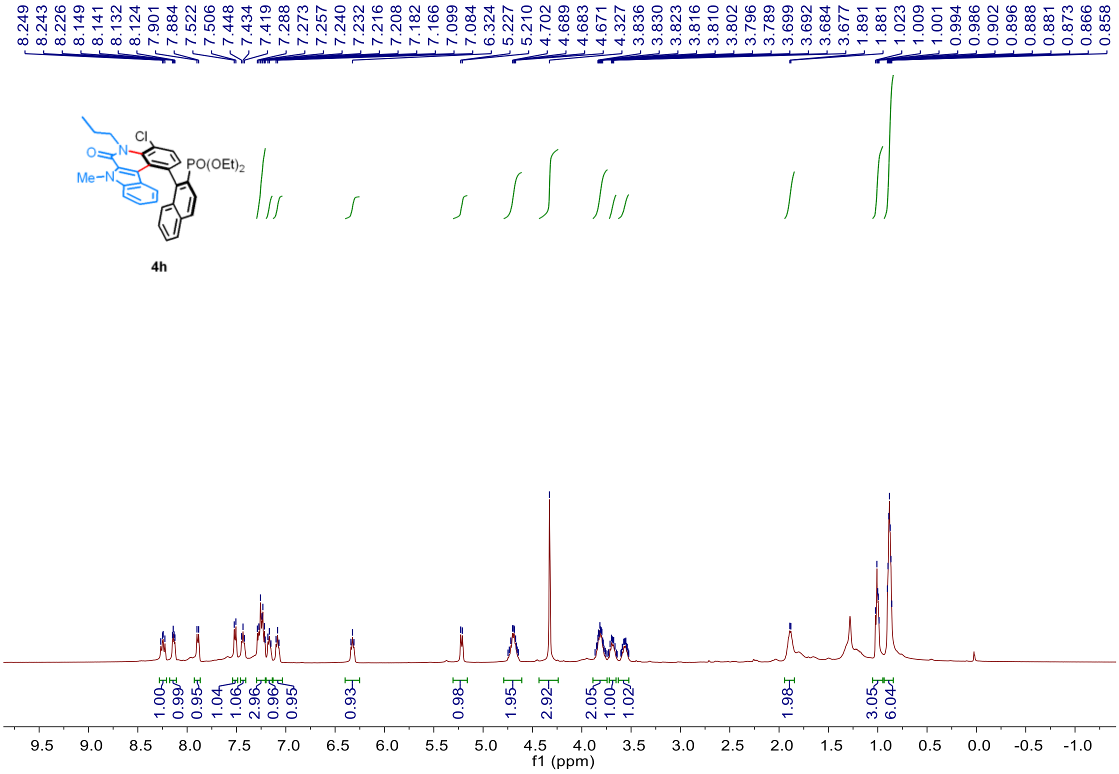

8.249 8.243 8.226 8.149 8.141 8.132 8.124 7.901 7.884 7.522 7.506 7.448 7.434 7.419 7.288 7.273 7.257 7.240 7.232 7.216 7.208 7.182 7.166 7.099 7.084 6.324 5.227 5.210 4.702 4.689 4.683 4.671 4.327 3.836 3.830 3.823 3.816 3.810 3.802 3.796 3.789 3.699 3.692 3.684 3.677 1.891 1.881 1.023 1.009 1.001 0.994 0.986 0.902 0.896 0.888 0.881 0.873 0.866 0.858
Cl
N
O
Me
PO(OEt)$_2$
4h
1.00 0.99 0.95 1.04 1.06 2.96 0.96 0.95 0.93 0.98 1.95 2.92 2.05 1.00 1.02 1.98 3.05 6.04
9.5 9.0 8.5 8.0 7.5 7.0 6.5 6.0 5.5 5.0 4.5 4.0 3.5 3.0 2.5 2.0 1.5 1.0 0.5 0.0 -0.5 -1.0
f1 (ppm)

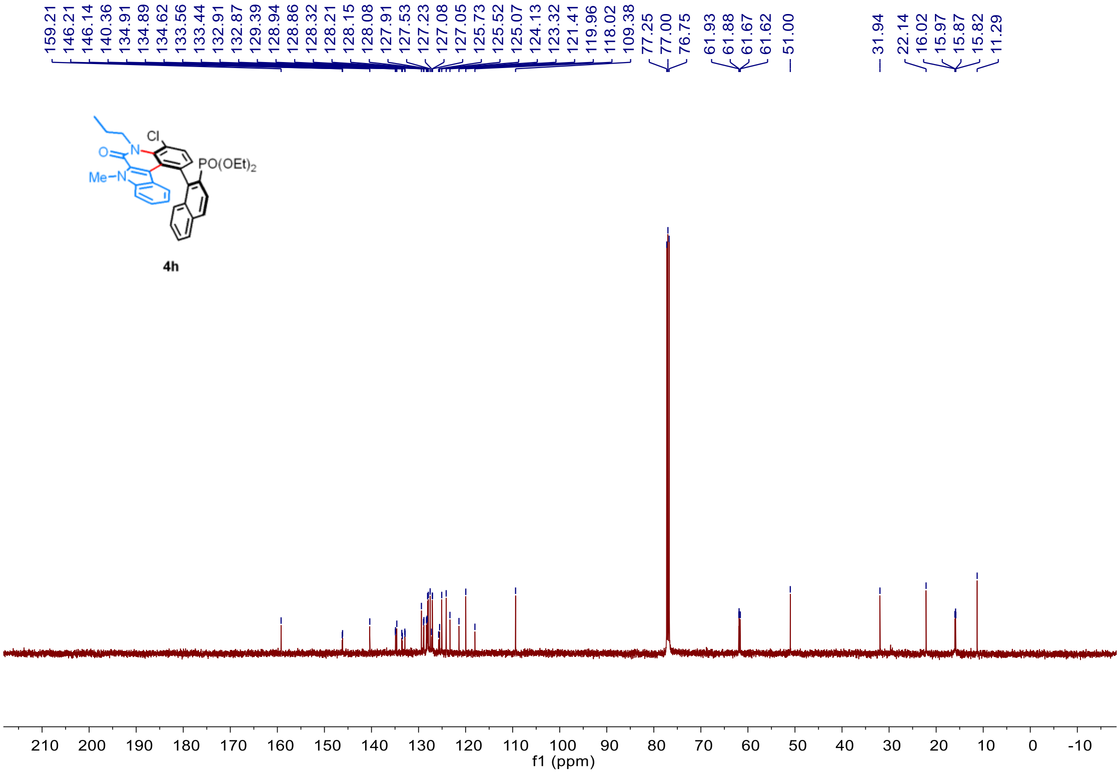

159.21 146.21 146.14 140.36 134.91 134.89 134.62 133.56 133.44 132.91 132.87 129.39 128.94 128.86 128.32 128.21 128.15 128.08 127.91 127.53 127.23 127.08 127.05 125.73 125.52 125.07 124.13 123.32 121.41 119.96 118.02 109.38 77.25 77.00 76.75 61.93 61.88 61.67 61.62 51.00 31.94 22.14 16.02 15.97 15.87 15.82 11.29
Cl
N
O
Me
PO(OEt)$_2$
4h
210 200 190 180 170 160 150 140 130 120 110 100 90 80 70 60 50 40 30 20 10 0 -10
f1 (ppm)

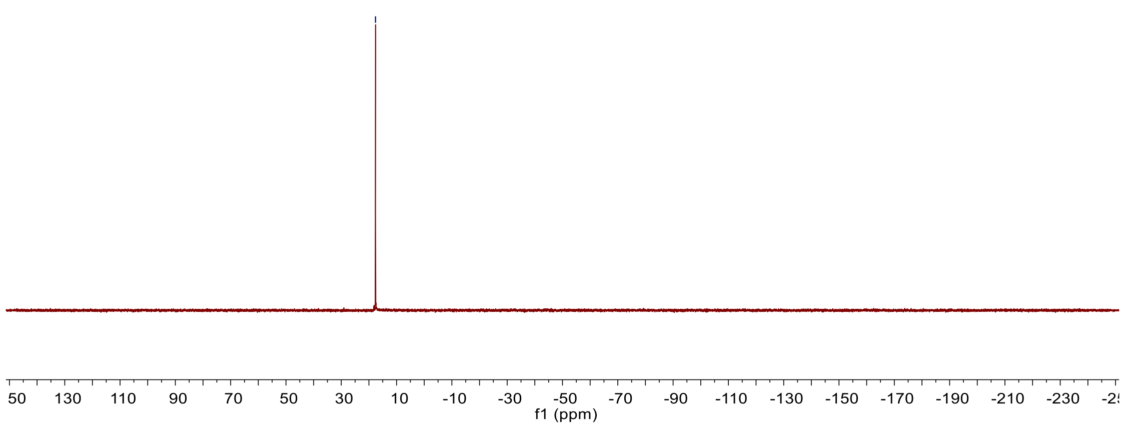
17.611
f1 (ppm)
50 130 110 90 70 50 30 10 -10 -30 -50 -70 -90 -110 -130 -150 -170 -190 -210 -230

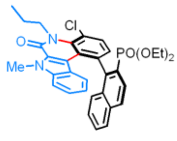
Cl
O
N
PO(OEt)2
Me
N

4h

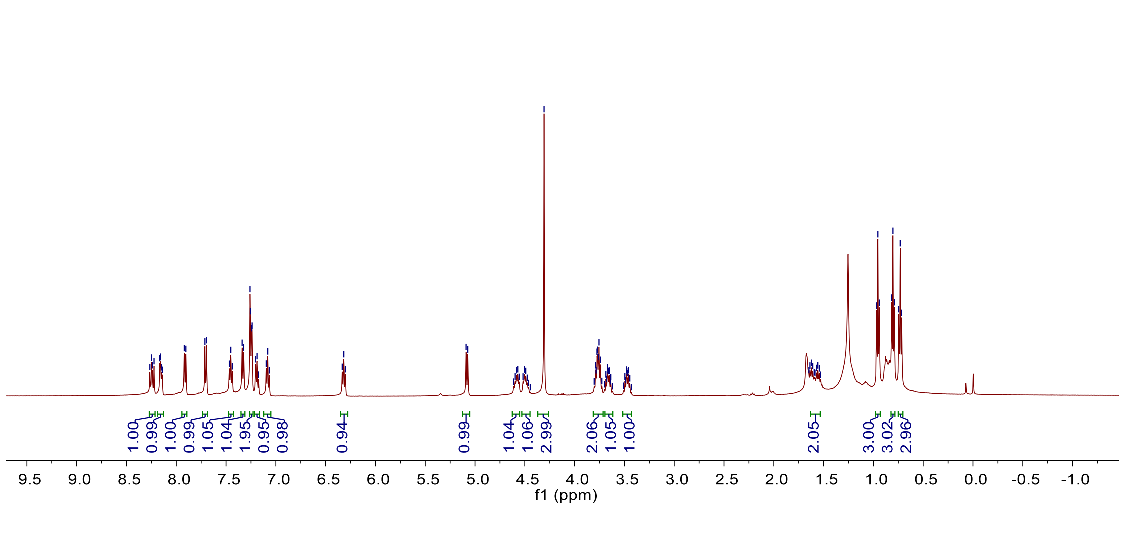
8.265 8.248 8.241 8.223 8.165 8.158 8.148 8.141 7.920 7.903 7.712 7.696 7.467 7.452 7.438 7.339 7.323 7.260 7.257 7.245 7.239 7.204 7.189 7.097 7.081 6.334 6.318 6.303 5.090 5.073 4.585 4.570 4.504 4.307 3.790 3.776 3.770 3.761 3.755 3.741 3.671 3.481 3.460 1.647 1.637 1.623 1.609 1.572 1.558 1.544 0.969 0.955 0.941 0.818 0.804 0.790 0.746 0.731 0.716
1.00 0.99 1.00 0.99 1.05 1.04 1.95 0.95 0.98 0.94 0.99 1.04 1.06 2.99 2.06 1.05 1.00 2.05 3.00 3.02 2.96
9.5 9.0 8.5 8.0 7.5 7.0 6.5 6.0 5.5 5.0 4.5 4.0 3.5 3.0 2.5 2.0 1.5 1.0 0.5 0.0 -0.5 -1.0
f1 (ppm)

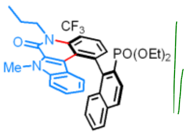
CF3
O
N
PO(OEt)2
Me
N

4i

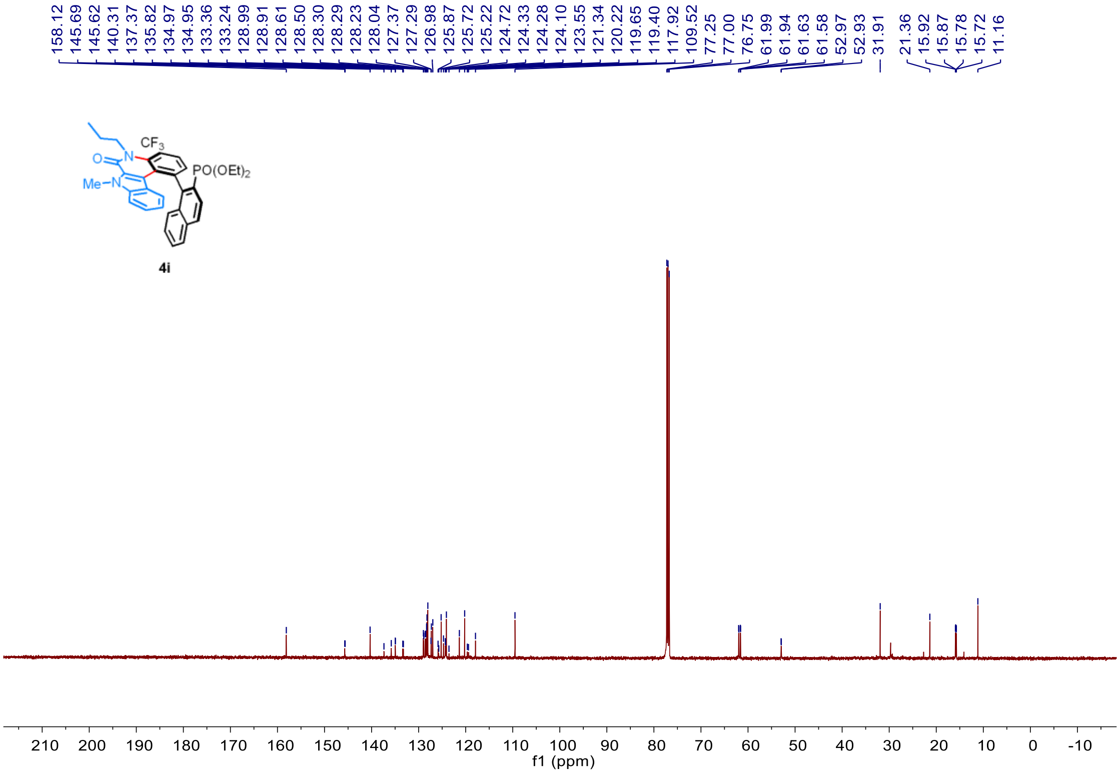
158.12
145.69
145.62
140.31
137.37
135.82
134.97
134.95
133.36
133.24
128.99
128.91
128.61
128.50
128.30
128.29
128.23
128.04
127.37
127.29
126.98
125.87
125.72
125.22
124.72
124.33
124.28
124.10
123.55
121.34
120.22
119.65
119.40
117.92
109.52
77.25
77.00
76.75
61.99
61.94
61.63
61.58
52.97
52.93
31.91
21.36
15.92
15.87
15.78
15.72
11.16
CF3
O
N
Me
PO(OEt)2
4i
210 200 190 180 170 160 150 140 130 120 110 100 90 80 70 60 50 40 30 20 10 0 -10
f1 (ppm)

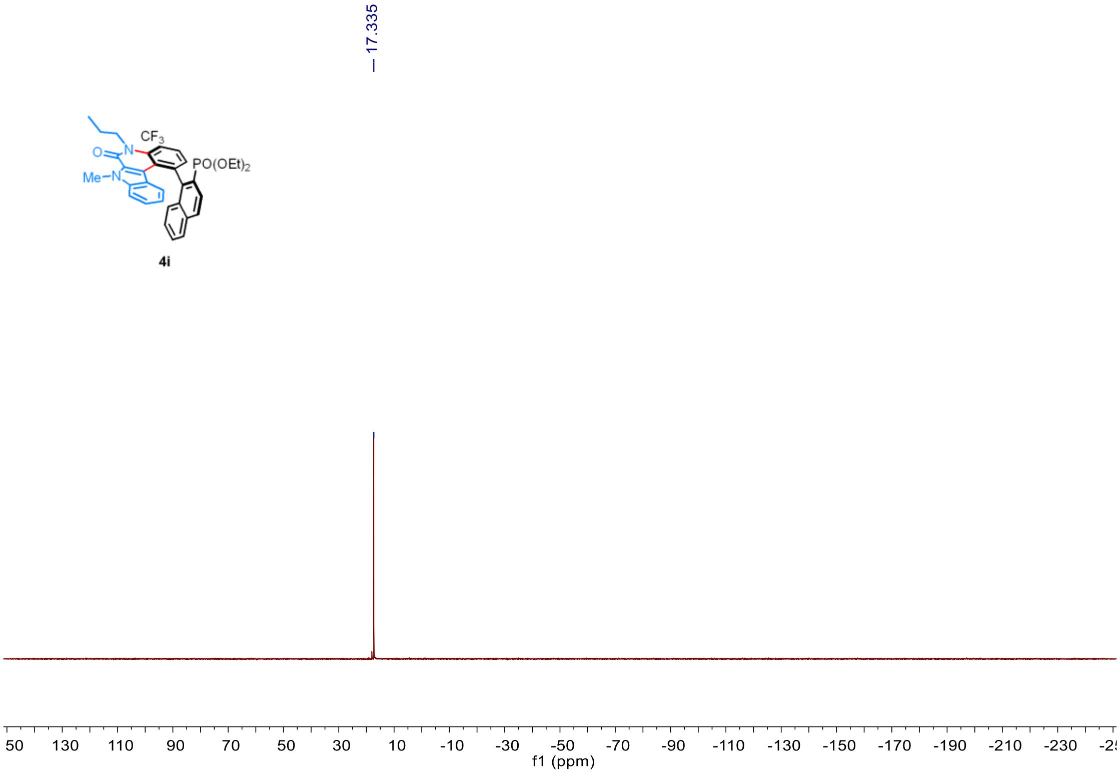
17.335
CF3
O
N
Me
PO(OEt)2
4i
130 110 90 70 50 30 10 -10 -30 -50 -70 -90 -110 -130 -150 -170 -190 -210 -230
f1 (ppm)

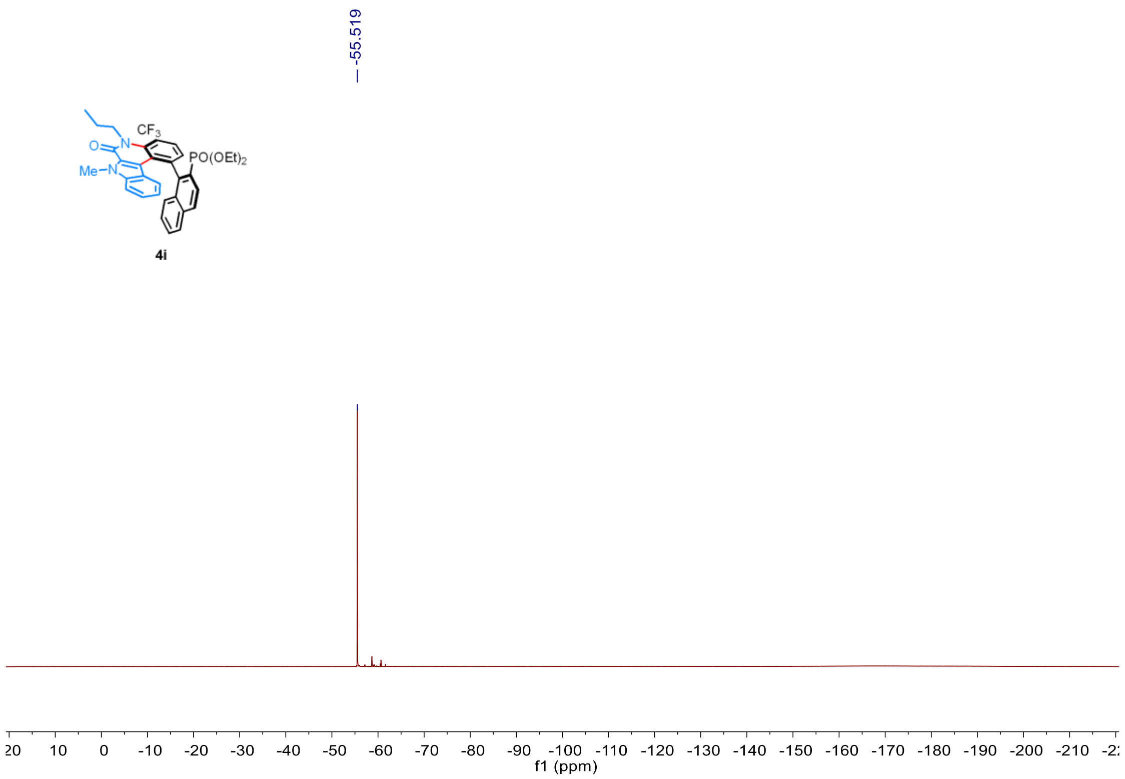
-55.519
CF3
PO(OEt)2
Me
4i
20 10 0 -10 -20 -30 -40 -50 -60 -70 -80 -90 -100 -110 -120 -130 -140 -150 -160 -170 -180 -190 -200 -210
f1 (ppm)

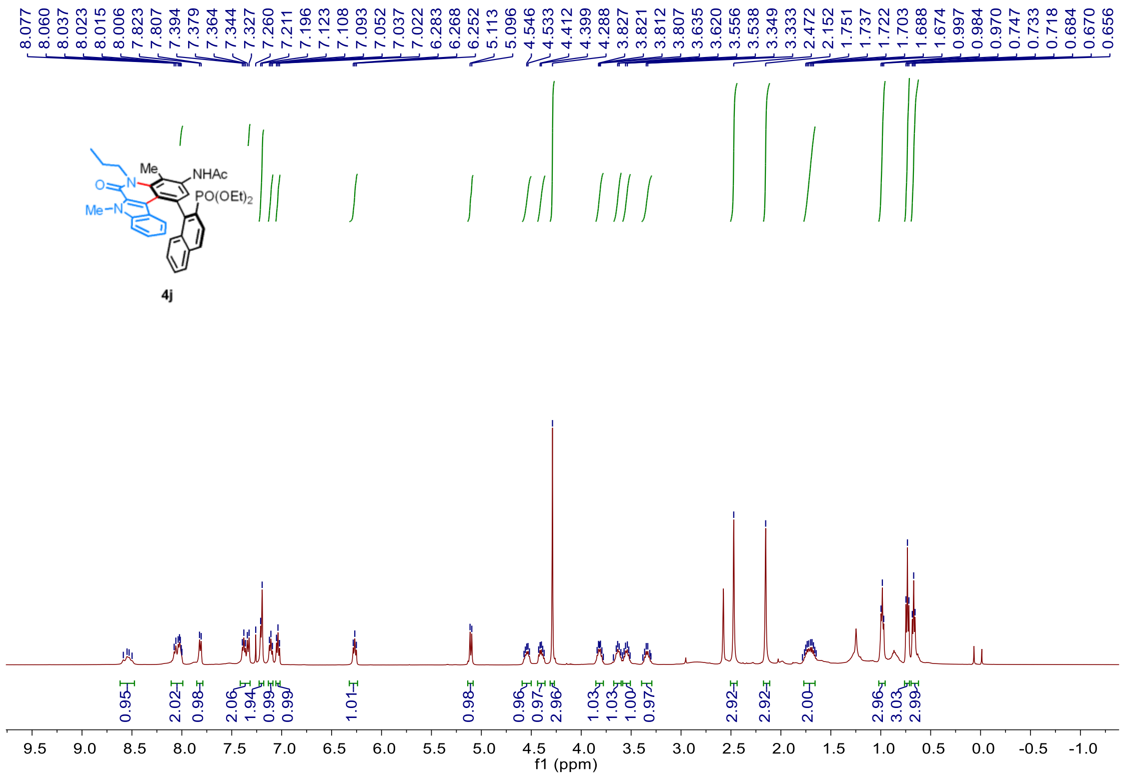
8.077 8.060 8.037 8.023 8.015 8.006 7.823 7.807 7.394 7.379 7.364 7.344 7.327 7.260 7.211 7.196 7.123 7.108 7.093 7.052 7.037 7.022 6.283 6.268 6.252 5.113 5.096 4.546 4.533 4.412 4.399 4.288 3.827 3.821 3.812 3.807 3.635 3.620 3.556 3.538 3.349 3.333 2.472 2.152 1.751 1.737 1.722 1.703 1.688 1.674 0.997 0.984 0.970 0.747 0.733 0.718 0.684 0.670 0.656
Me
NHAc
PO(OEt)2
4j
0.95 2.02 0.98 2.06 1.94 0.99 0.99 1.01 0.98 0.96 0.97 2.96 1.03 1.03 1.00 0.97 2.92 2.92 2.00 2.96 3.03 2.99
9.5 9.0 8.5 8.0 7.5 7.0 6.5 6.0 5.5 5.0 4.5 4.0 3.5 3.0 2.5 2.0 1.5 1.0 0.5 0.0 -0.5 -1.0
f1 (ppm)

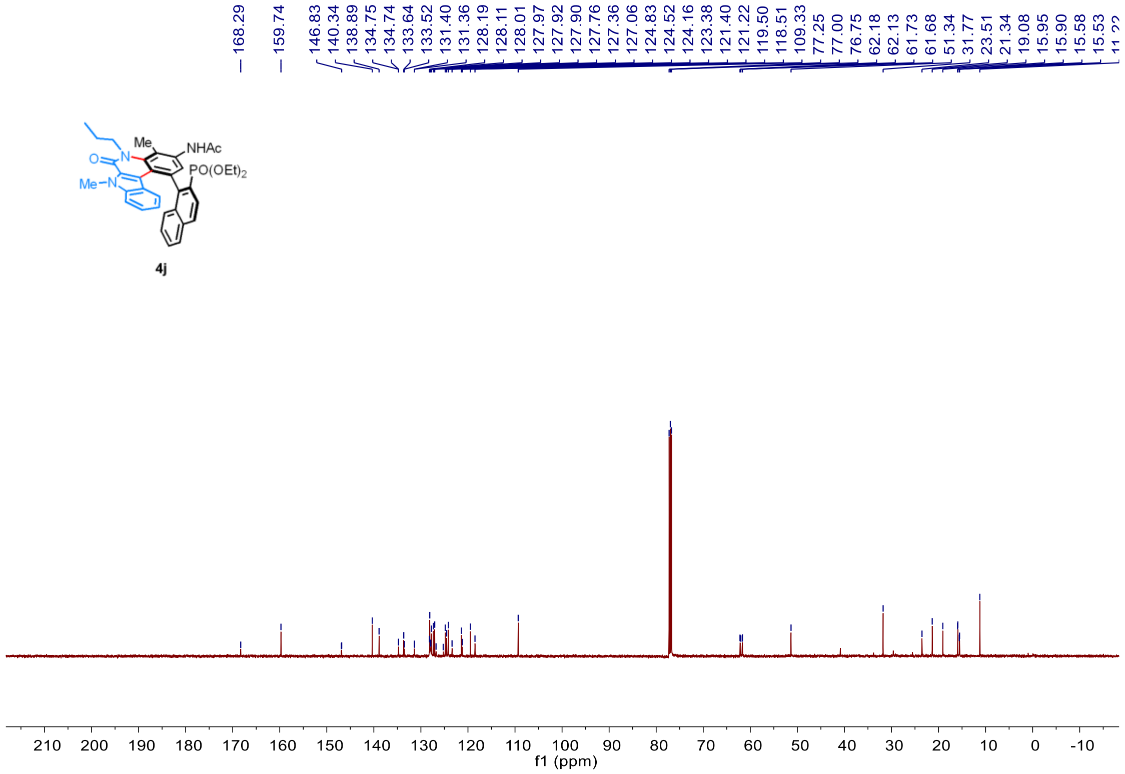
168.29
159.74
146.83
140.34
138.89
134.75
134.74
133.64
133.52
131.40
131.36
128.19
128.11
128.01
127.97
127.92
127.90
127.76
127.36
127.06
124.83
124.52
124.16
123.38
121.40
121.22
119.50
118.51
109.33
77.25
77.00
76.75
62.18
62.13
61.73
61.68
51.34
31.77
23.51
21.34
19.08
15.95
15.90
15.58
15.53
Me
NHAc
O
N
PO(OEt)2
Me
N
4j
210 200 190 180 170 160 150 140 130 120 110 100 90 80 70 60 50 40 30 20 10 0 -10
f1 (ppm)

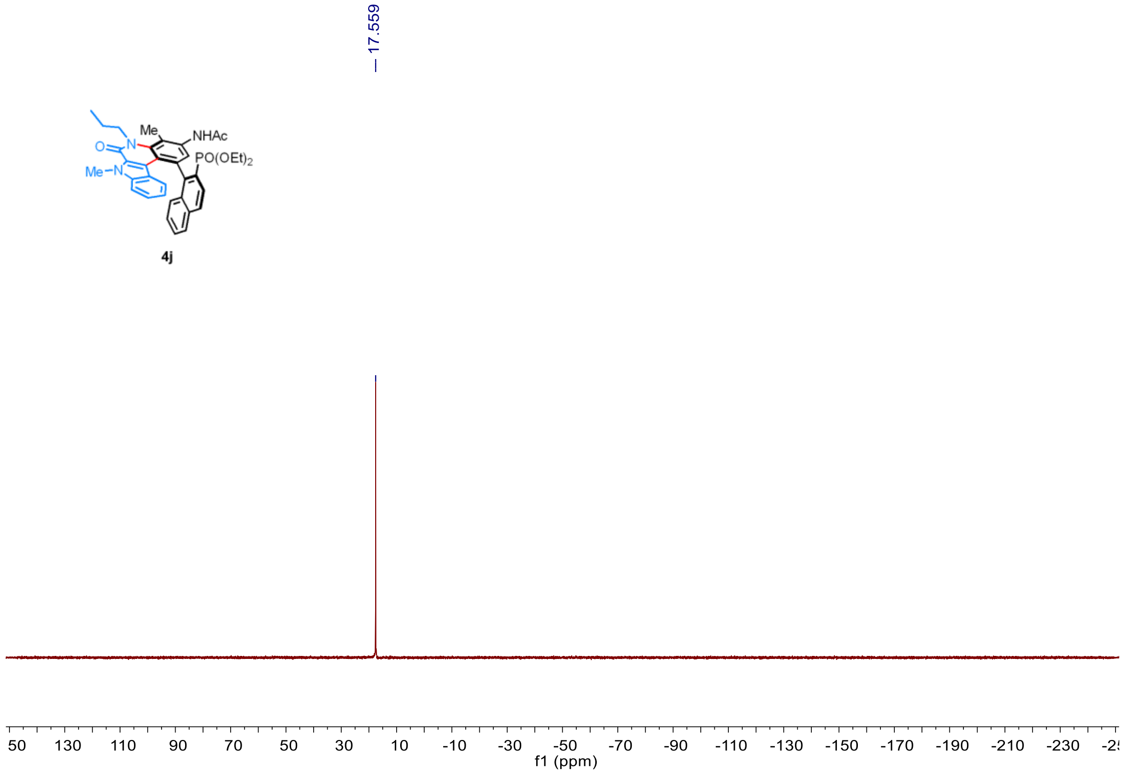
17.559
Me
NHAc
O
N
PO(OEt)2
Me
N
4j
50 130 110 90 70 50 30 10 -10 -30 -50 -70 -90 -110 -130 -150 -170 -190 -210 -230
f1 (ppm)

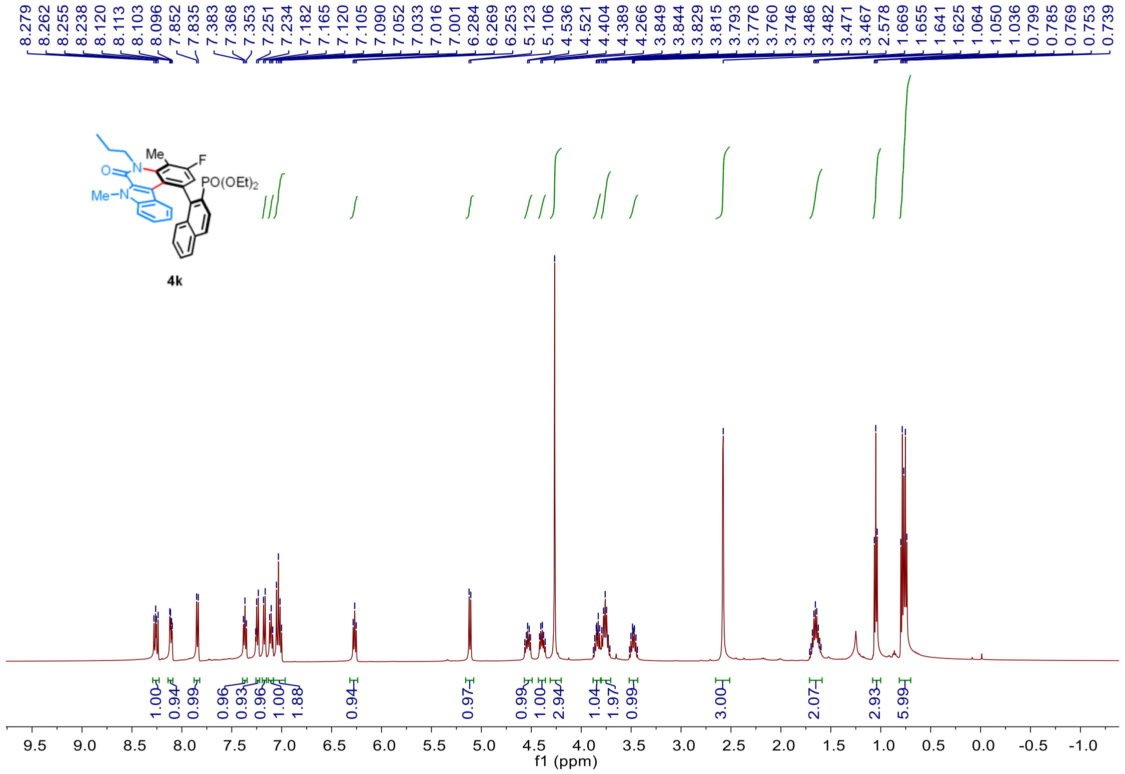
8.279 8.262 8.255 8.238 8.120 8.113 8.103 8.096 7.852 7.835 7.383 7.368 7.353 7.251 7.234 7.182 7.165 7.120 7.105 7.090 7.052 7.033 7.016 7.001 6.284 6.269 6.253 5.123 5.106 4.536 4.521 4.404 4.389 4.266 3.849 3.844 3.829 3.815 3.793 3.776 3.760 3.746 3.486 3.482 3.471 3.467 2.578 1.669 1.655 1.641 1.625 1.064 1.050 1.036 0.799 0.785 0.769 0.753 0.739
Me
F
O
N
PO(OEt)2
Me–N
4k
1.00 0.94 0.99 0.96 0.93 0.96 1.00 1.88 0.94 0.97 0.99 1.00 2.94 1.04 1.97 0.99 3.00 2.07 2.93 5.99
9.5 9.0 8.5 8.0 7.5 7.0 6.5 6.0 5.5 5.0 4.5 4.0 3.5 3.0 2.5 2.0 1.5 1.0 0.5 0.0 -0.5 -1.0
f1 (ppm)

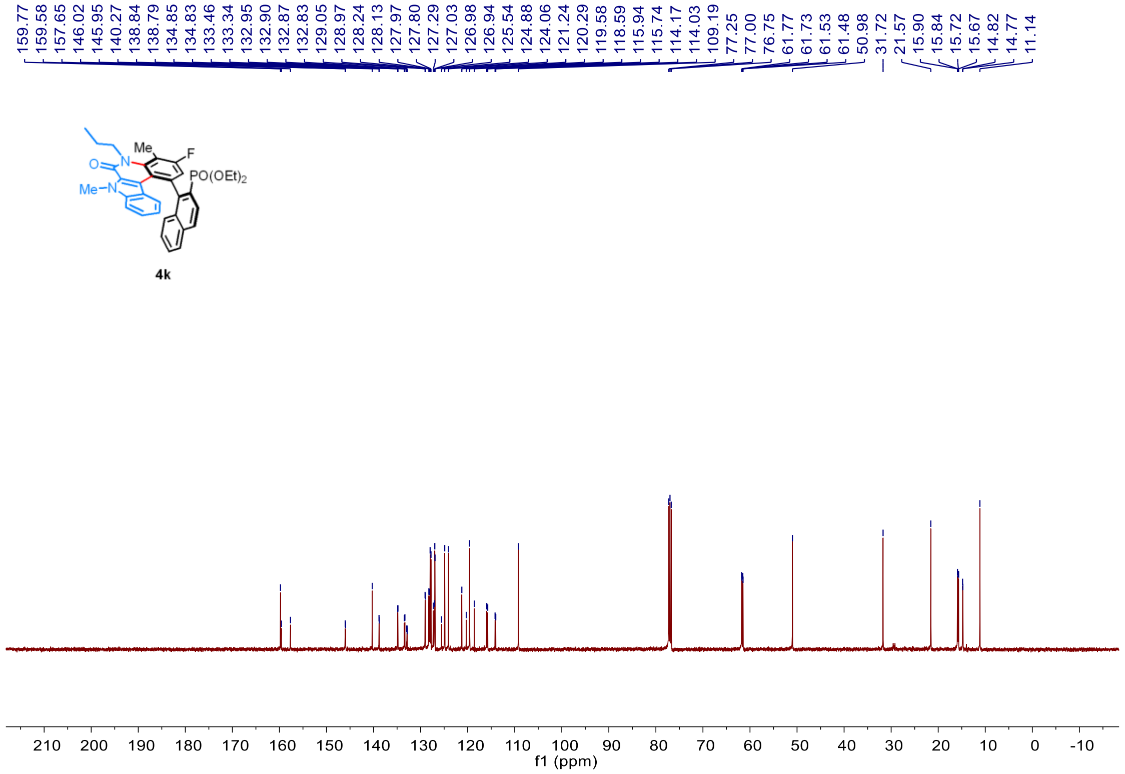
159.77 159.58 157.65 146.02 145.95 140.27 138.84 138.79 134.85 134.83 133.46 133.34 132.95 132.90 132.87 132.83 129.05 128.97 128.24 128.13 127.97 127.80 127.29 127.03 126.98 126.94 125.54 124.88 124.06 121.24 120.29 119.58 118.59 115.94 115.74 114.17 114.03 109.19 77.25 77.00 76.75 61.77 61.73 61.53 61.48 50.98 31.72 21.57 15.90 15.84 15.72 15.67 14.82 14.77 11.14
Me
F
O
N
PO(OEt)2
Me–N
4k
210 200 190 180 170 160 150 140 130 120 110 100 90 80 70 60 50 40 30 20 10 0 -10
f1 (ppm)

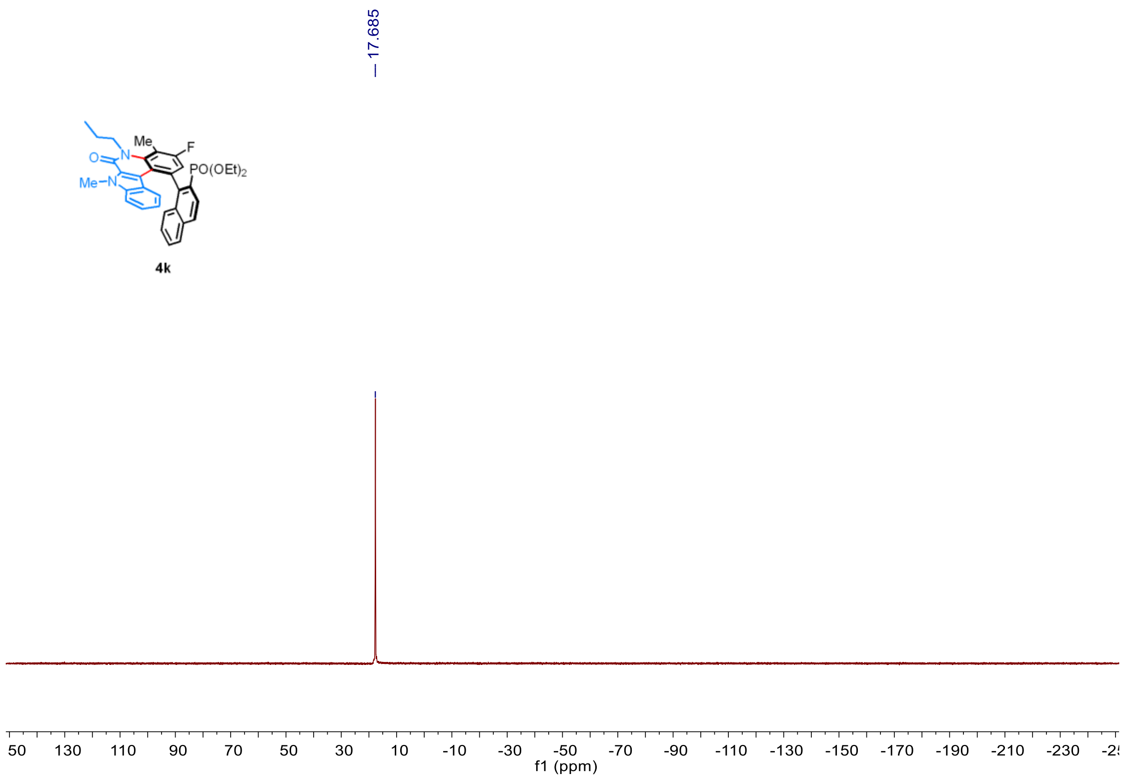

17.685
Me
F
O
N
PO(OEt)2
Me–N
4k
50 130 110 90 70 50 30 10 -10 -30 -50 -70 -90 -110 -130 -150 -170 -190 -210 -230
f1 (ppm)

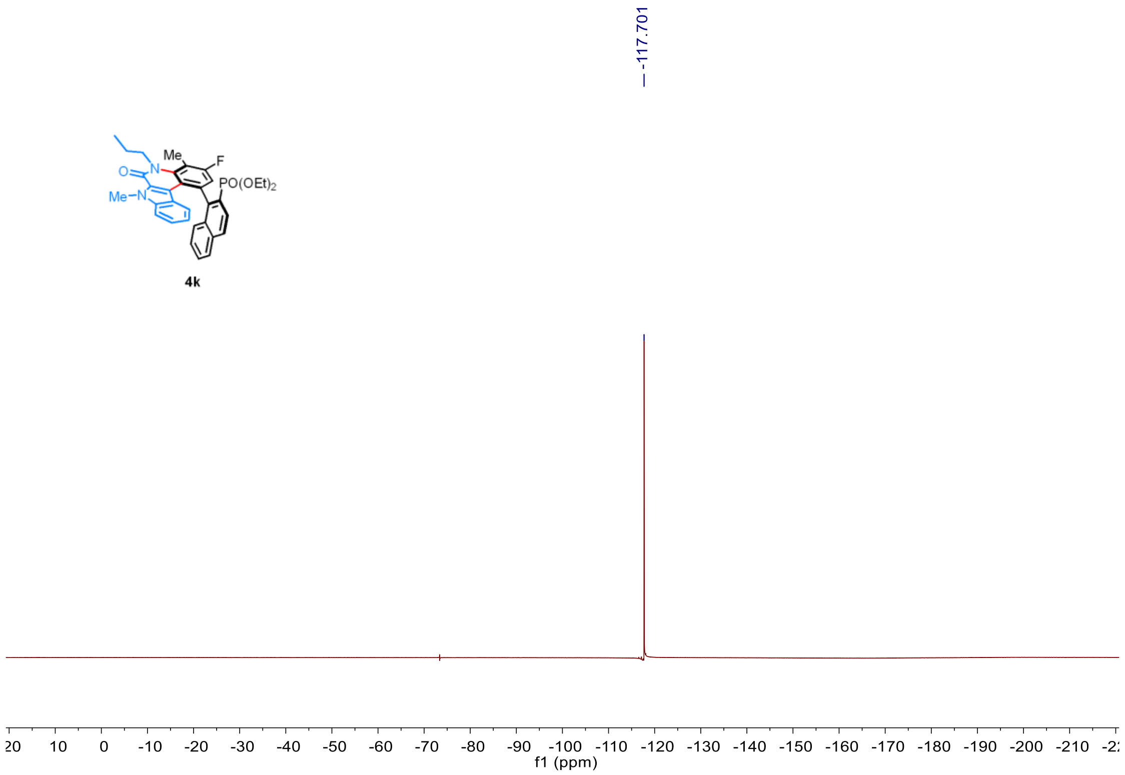

-117.701
Me
F
O
N
PO(OEt)2
Me–N
4k
20 10 0 -10 -20 -30 -40 -50 -60 -70 -80 -90 -100 -110 -120 -130 -140 -150 -160 -170 -180 -190 -200 -210
f1 (ppm)

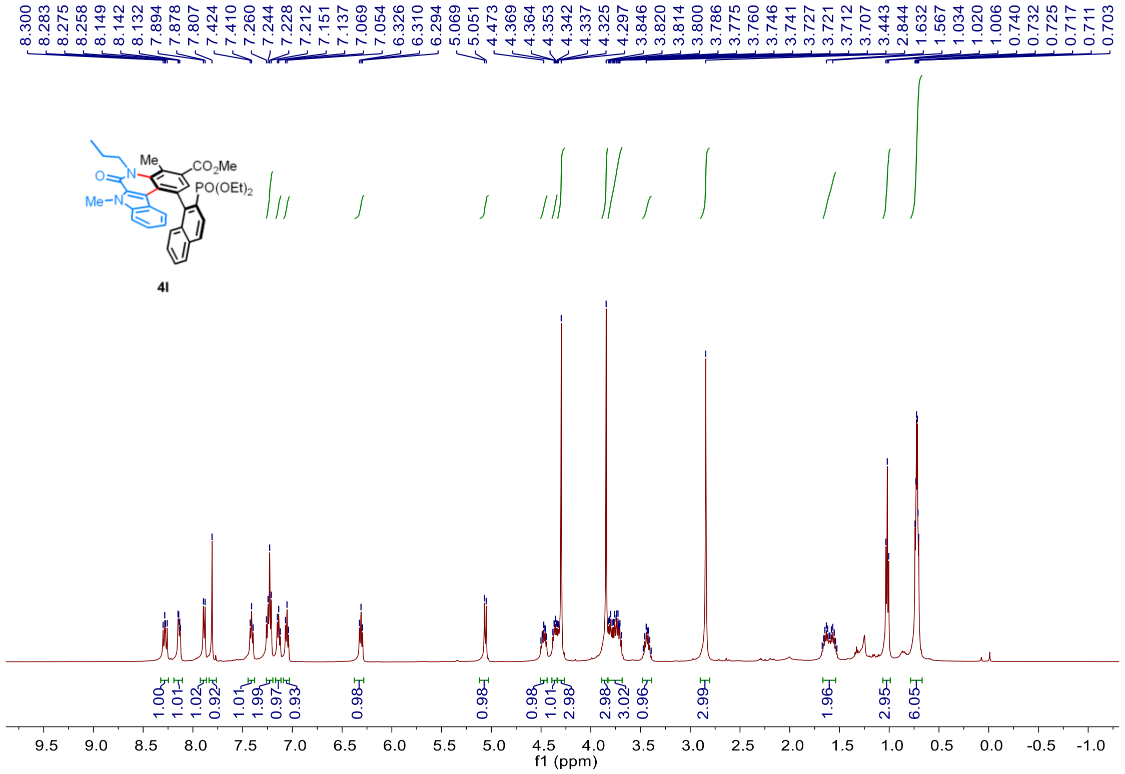

8.300 8.283 8.275 8.258 8.149 8.142 8.132 7.894 7.878 7.807 7.424 7.410 7.260 7.244 7.228 7.212 7.151 7.137 7.069 7.054 6.326 6.310 6.294 5.069 5.051 4.473 4.369 4.364 4.353 4.342 4.337 4.325 4.297 3.846 3.820 3.814 3.800 3.786 3.775 3.760 3.746 3.741 3.727 3.721 3.712 3.707 3.443 2.844 1.632 1.567 1.034 1.020 1.006 0.740 0.732 0.725 0.717 0.711 0.703
Me
CO_2Me
O
N
$PO(OEt)_2$
Me–N
4l
1.00 1.01 1.02 0.92 1.01 1.99 0.97 0.93 0.98 0.98 0.98 1.01 2.98 2.98 3.02 0.96 2.99 1.96 2.95 6.05
9.5 9.0 8.5 8.0 7.5 7.0 6.5 6.0 5.5 5.0 4.5 4.0 3.5 3.0 2.5 2.0 1.5 1.0 0.5 0.0 -0.5 -1.0
f1 (ppm)

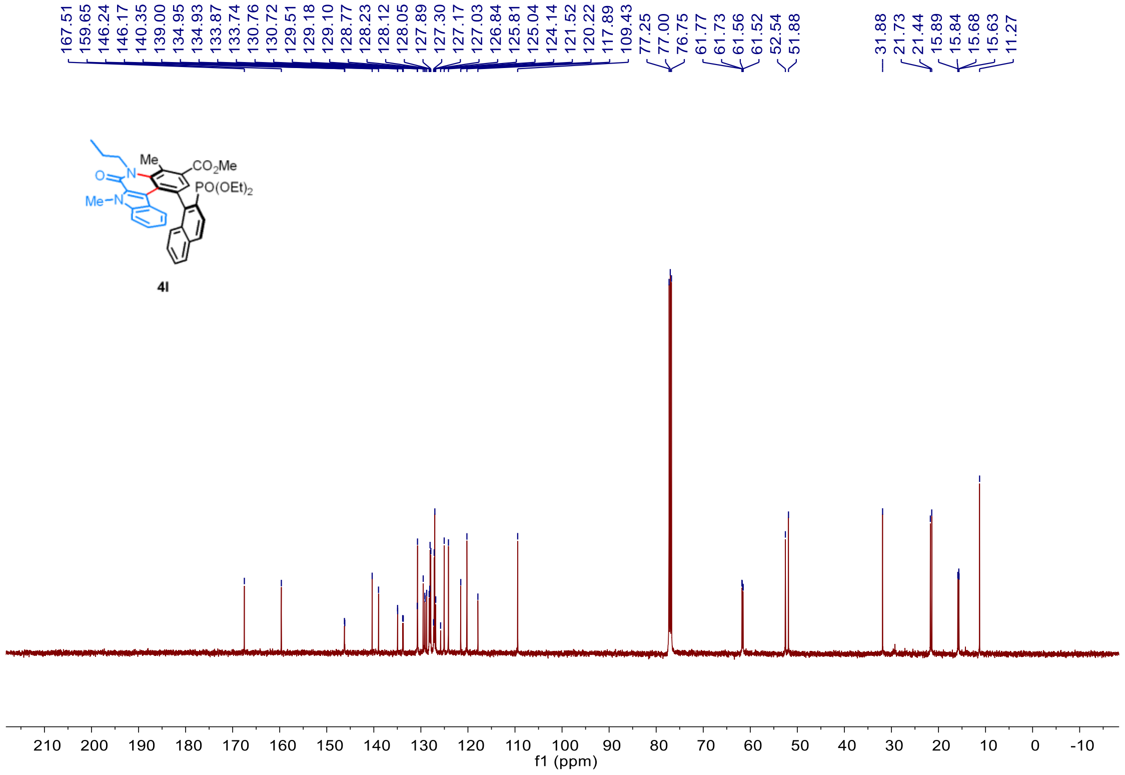

167.51 159.65 146.24 146.17 140.35 139.00 134.95 134.93 133.87 133.74 130.76 130.72 129.51 129.18 129.10 128.77 128.23 128.12 128.05 127.89 127.30 127.17 127.03 126.84 125.81 125.04 124.14 121.52 120.22 117.89 109.43 77.25 77.00 76.75 61.77 61.73 61.56 61.52 52.54 51.88 31.88 21.73 21.44 15.89 15.84 15.68 15.63 11.27
Me
CO_2Me
O
N
$PO(OEt)_2$
Me–N
4l
210 200 190 180 170 160 150 140 130 120 110 100 90 80 70 60 50 40 30 20 10 0 -10
f1 (ppm)

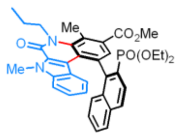
Me
CO2Me
PO(OEt)2
O
N
Me–N

4l

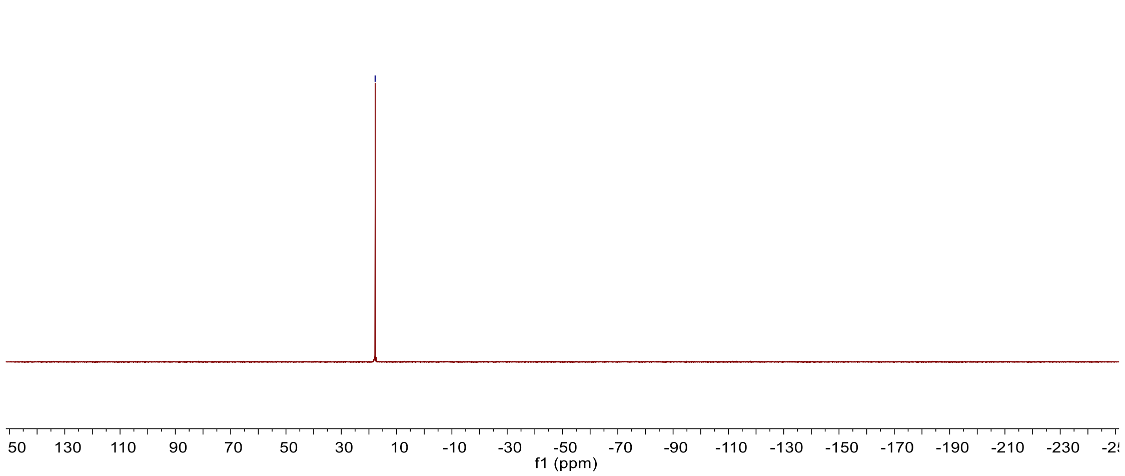
17.746
f1 (ppm)

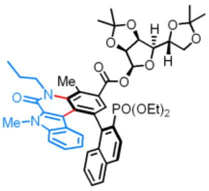
H
O
Me
PO(OEt)2
Me–N

4m

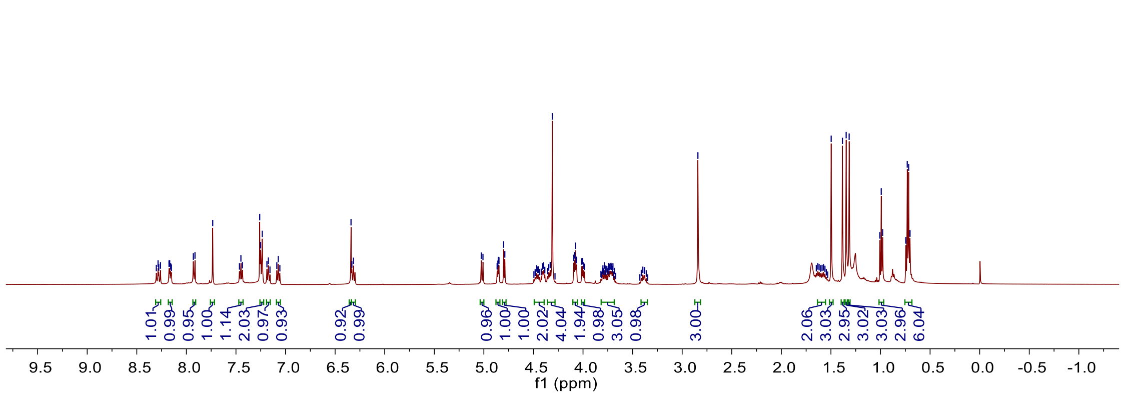
8.286
8.261
8.175
8.168
8.158
7.930
7.913
7.734
7.464
7.450
7.435
7.260
7.251
7.234
7.188
7.173
7.088
7.073
6.340
6.331
6.315
5.026
5.009
4.866
4.859
4.855
4.847
4.800
4.789
4.408
4.398
4.331
4.310
4.093
4.083
4.077
4.069
4.065
4.013
4.004
3.995
3.785
3.745
3.728
3.719
3.698
2.842
1.632
1.497
1.384
1.346
1.316
1.007
0.993
0.979
0.745
0.730
0.716
0.702
1.01
0.99
0.95
1.00
1.14
2.03
0.97
0.93
0.92
0.99
0.96
1.00
1.00
2.02
4.04
1.94
0.98
3.05
0.98
3.00
2.06
3.03
2.95
3.02
3.03
2.96
6.04
f1 (ppm)

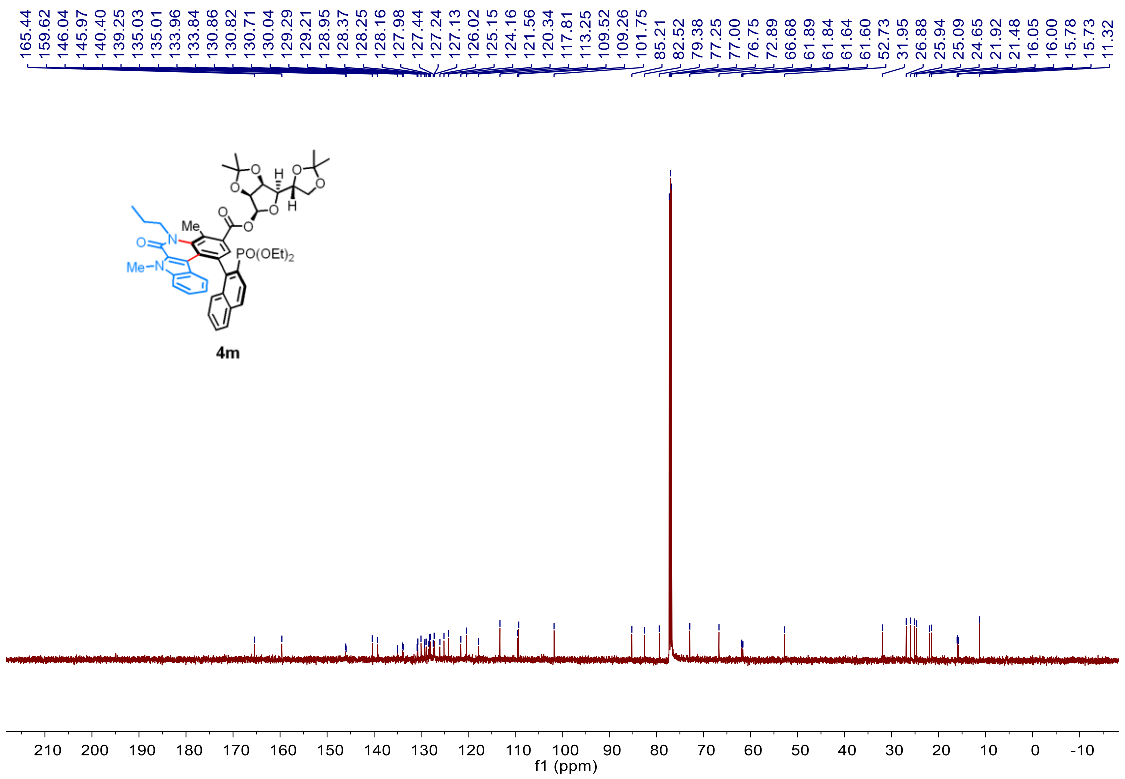
165.44
159.62
146.04
145.97
140.40
139.25
135.03
135.01
133.96
133.84
130.86
130.82
130.71
130.04
129.29
129.21
128.95
128.37
128.25
128.16
127.98
127.44
127.24
127.13
126.02
125.15
124.16
121.56
120.34
117.81
113.25
109.52
109.26
101.75
85.21
82.52
79.38
77.25
77.00
76.75
72.89
66.68
61.89
61.84
61.64
61.60
52.73
31.95
26.88
25.94
25.09
24.65
21.92
21.48
16.05
16.00
15.78
15.73
11.32
Me
PO(OEt)$_2$
Me
O
N
H
4m
f1 (ppm)

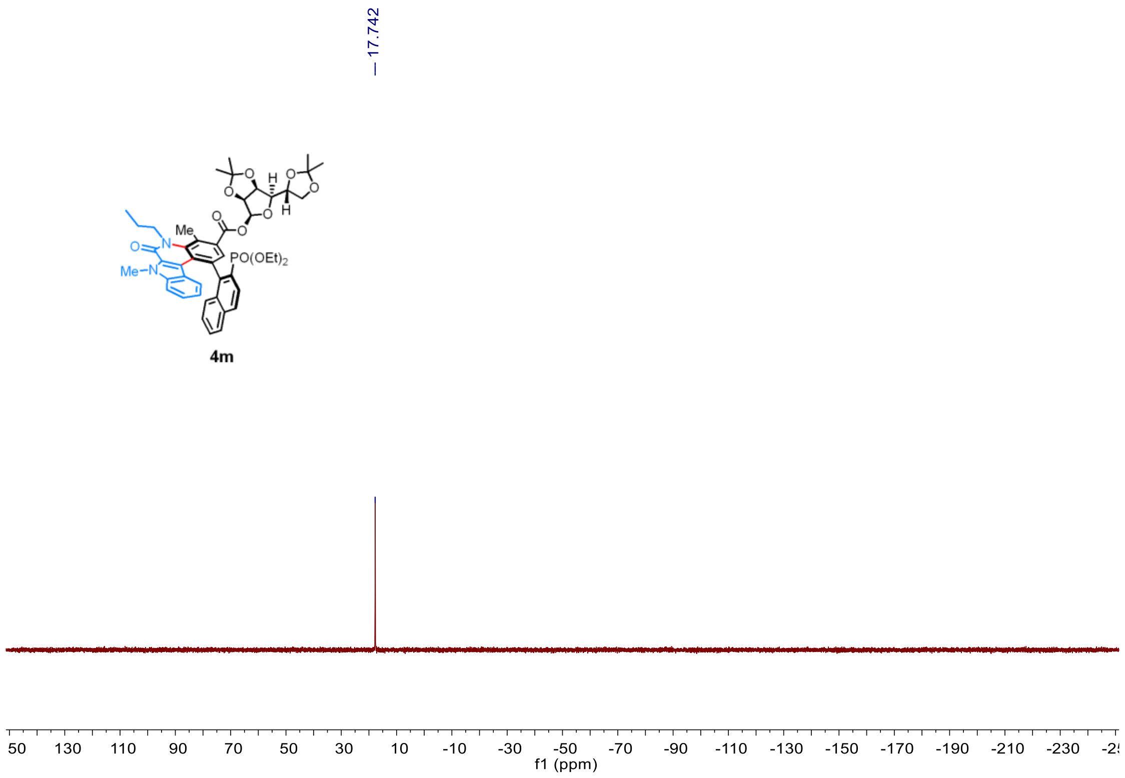
17.742
PO(OEt)$_2$
Me
4m
f1 (ppm)

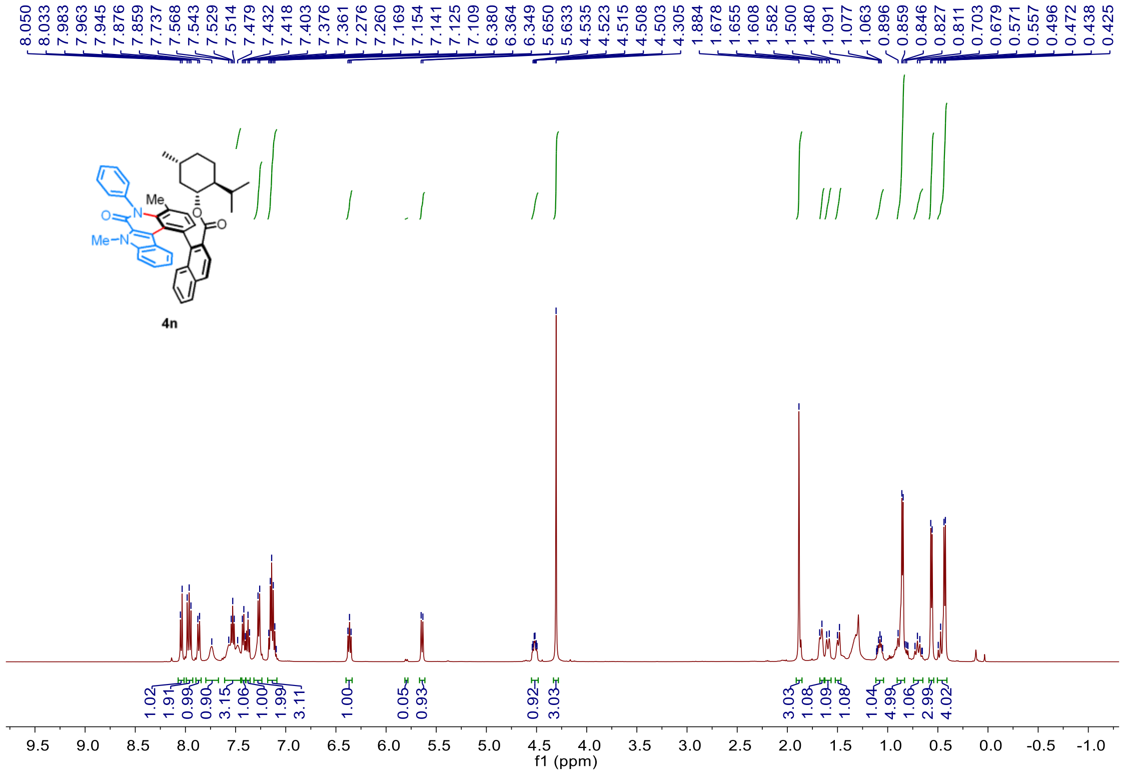
8.050 8.033 7.983 7.963 7.945 7.876 7.859 7.737 7.568 7.543 7.529 7.514 7.479 7.432 7.418 7.403 7.376 7.361 7.276 7.260 7.169 7.154 7.141 7.125 7.109 6.380 6.364 6.349 5.650 5.633 4.535 4.523 4.515 4.508 4.503 4.305 1.884 1.678 1.655 1.608 1.582 1.500 1.480 1.091 1.077 1.063 0.896 0.859 0.846 0.827 0.811 0.703 0.679 0.571 0.557 0.496 0.472 0.438 0.425
Me
O
N
Me–N
O
O
4n
1.02 1.91 0.99 0.90 3.15 1.06 1.00 1.99 3.11 1.00 0.05 0.93 0.92 3.03 3.03 1.08 1.09 1.08 1.04 4.99 1.06 2.99 4.02
9.5 9.0 8.5 8.0 7.5 7.0 6.5 6.0 5.5 5.0 4.5 4.0 3.5 3.0 2.5 2.0 1.5 1.0 0.5 0.0 -0.5 -1.0
f1 (ppm)

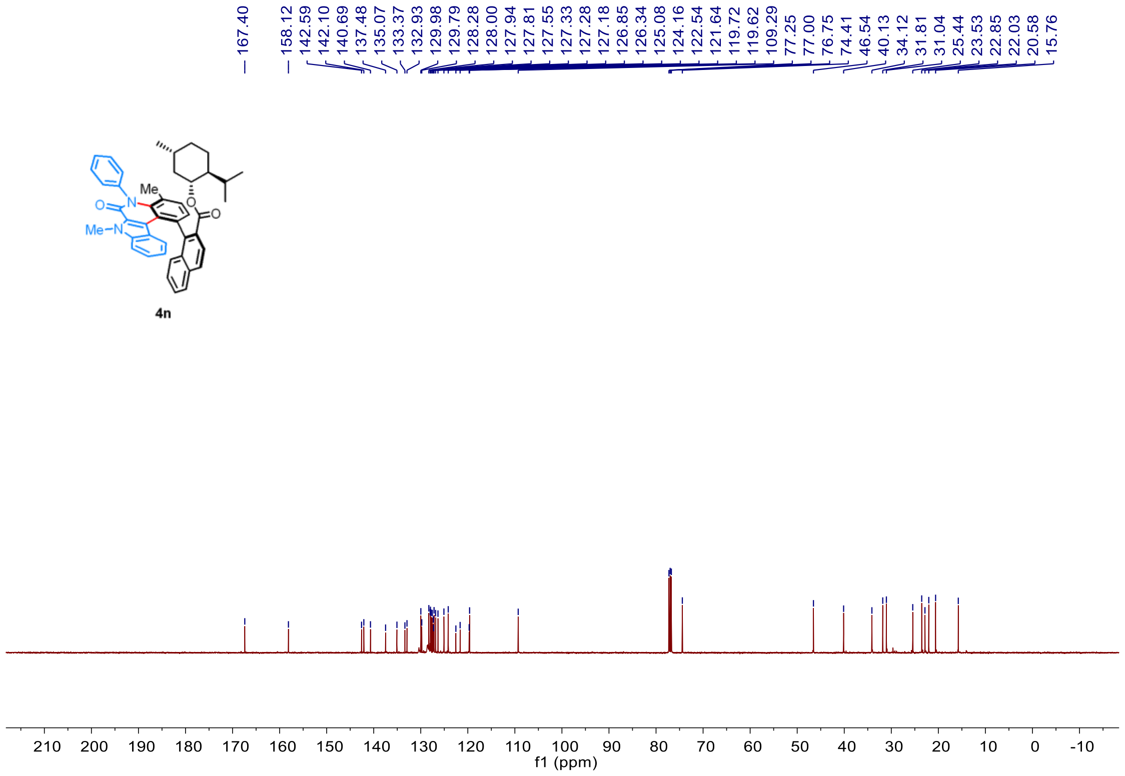
167.40 158.12 142.59 142.10 140.69 137.48 135.07 133.37 132.93 129.98 129.79 128.28 128.00 127.94 127.81 127.55 127.33 127.28 127.18 126.85 126.34 125.08 124.16 122.54 121.64 119.72 109.29 77.25 77.00 76.75 74.41 46.54 40.13 34.12 31.81 31.04 25.44 23.53 22.85 22.03 20.58 15.76
Me
O
N
Me–N
O
O
4n
210 200 190 180 170 160 150 140 130 120 110 100 90 80 70 60 50 40 30 20 10 0 -10
f1 (ppm)

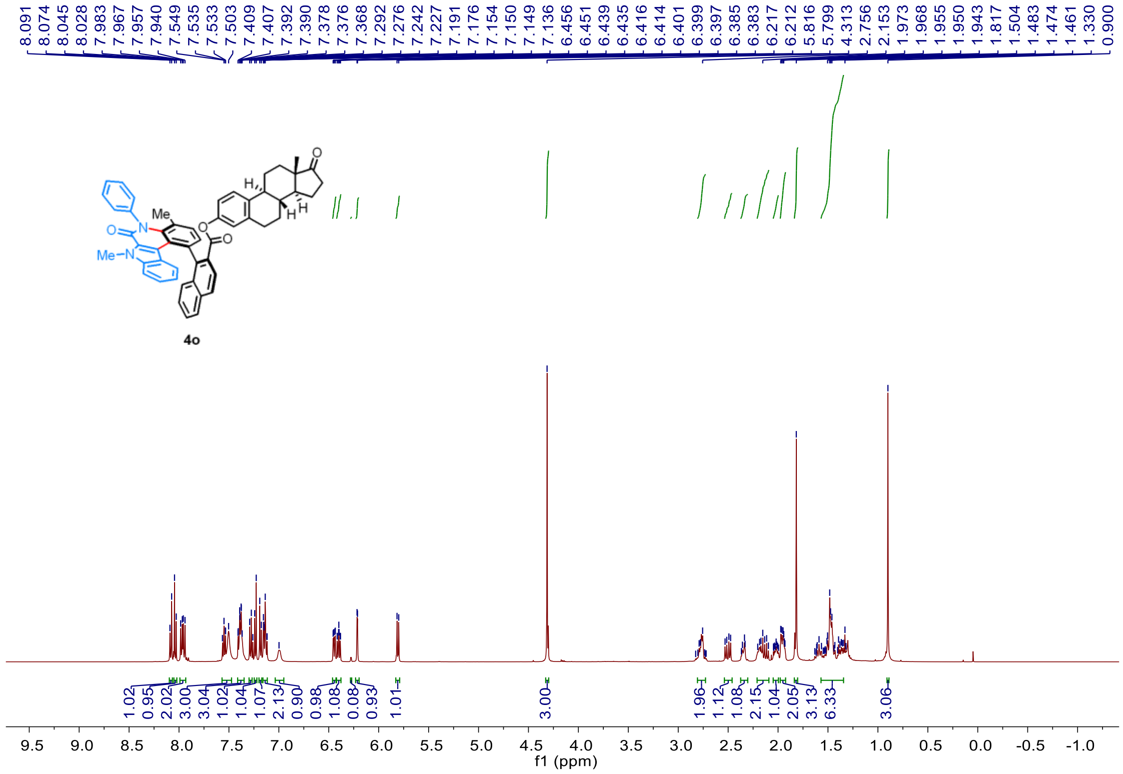
8.091 8.074 8.045 8.028 7.983 7.967 7.957 7.940 7.549 7.535 7.533 7.503 7.409 7.407 7.392 7.390 7.378 7.376 7.368 7.292 7.276 7.242 7.227 7.191 7.176 7.154 7.150 7.149 7.136 6.456 6.451 6.439 6.435 6.416 6.414 6.401 6.399 6.397 6.385 6.383 6.217 6.212 5.816 5.799 4.313 2.756 2.153 1.973 1.968 1.955 1.950 1.943 1.817 1.504 1.483 1.474 1.461 1.330 0.900
Me
O
N
Me–N
H
H
H
O
O
O
4o
1.02 0.95 2.02 3.00 3.04 1.02 1.04 1.07 2.13 0.90 0.98 1.08 0.08 0.93 1.01 3.00 1.96 1.12 1.08 2.15 1.04 2.05 3.13 6.33 3.06
9.5 9.0 8.5 8.0 7.5 7.0 6.5 6.0 5.5 5.0 4.5 4.0 3.5 3.0 2.5 2.0 1.5 1.0 0.5 0.0 -0.5 -1.0
f1 (ppm)

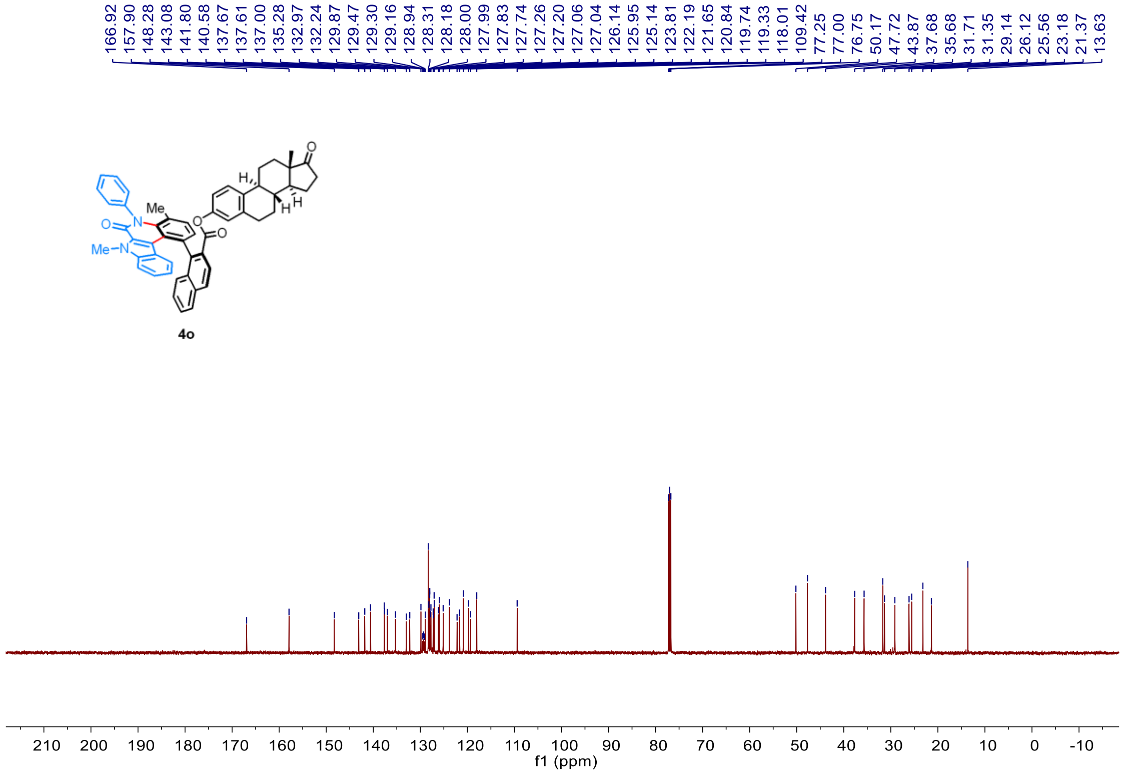
166.92 157.90 148.28 143.08 141.80 140.58 137.67 137.61 137.00 135.28 132.97 132.24 129.87 129.47 129.30 129.16 128.94 128.31 128.18 128.00 127.99 127.83 127.74 127.26 127.20 127.06 127.04 126.14 125.95 125.14 123.81 122.19 121.65 120.84 119.74 119.33 118.01 109.42 77.25 77.00 76.75 50.17 47.72 43.87 37.68 35.68 31.71 31.35 29.14 26.12 25.56 23.18 21.37 13.63
Me
O
N
Me–N
H
H
H
O
O
O
4o
210 200 190 180 170 160 150 140 130 120 110 100 90 80 70 60 50 40 30 20 10 0 -10
f1 (ppm)

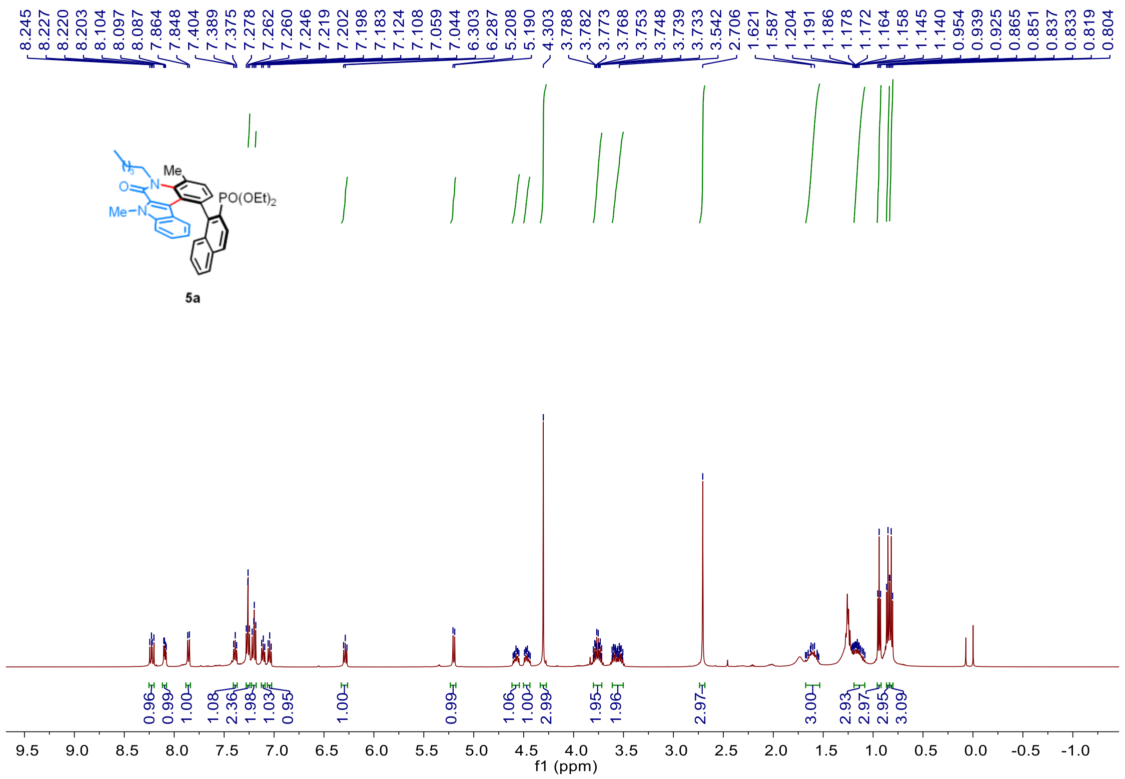
8.245
8.227
8.220
8.203
8.104
8.097
8.087
7.864
7.848
7.404
7.389
7.375
7.278
7.262
7.260
7.246
7.219
7.202
7.198
7.183
7.124
7.108
7.059
7.044
6.303
6.287
5.208
5.190
4.303
3.788
3.782
3.773
3.768
3.753
3.748
3.739
3.733
3.542
2.706
1.621
1.587
1.204
1.191
1.186
1.178
1.172
1.164
1.158
1.145
1.140
0.954
0.939
0.925
0.865
0.851
0.837
0.833
0.819
0.804
Me
O
N
PO(OEt)2
Me–N
5a
0.96
0.99
1.00
1.08
2.36
1.98
1.03
0.95
1.00
0.99
1.06
1.00
2.99
1.95
1.96
2.97
3.00
2.93
2.97
2.95
3.09
9.5 9.0 8.5 8.0 7.5 7.0 6.5 6.0 5.5 5.0 4.5 4.0 3.5 3.0 2.5 2.0 1.5 1.0 0.5 0.0 -0.5 -1.0
f1 (ppm)

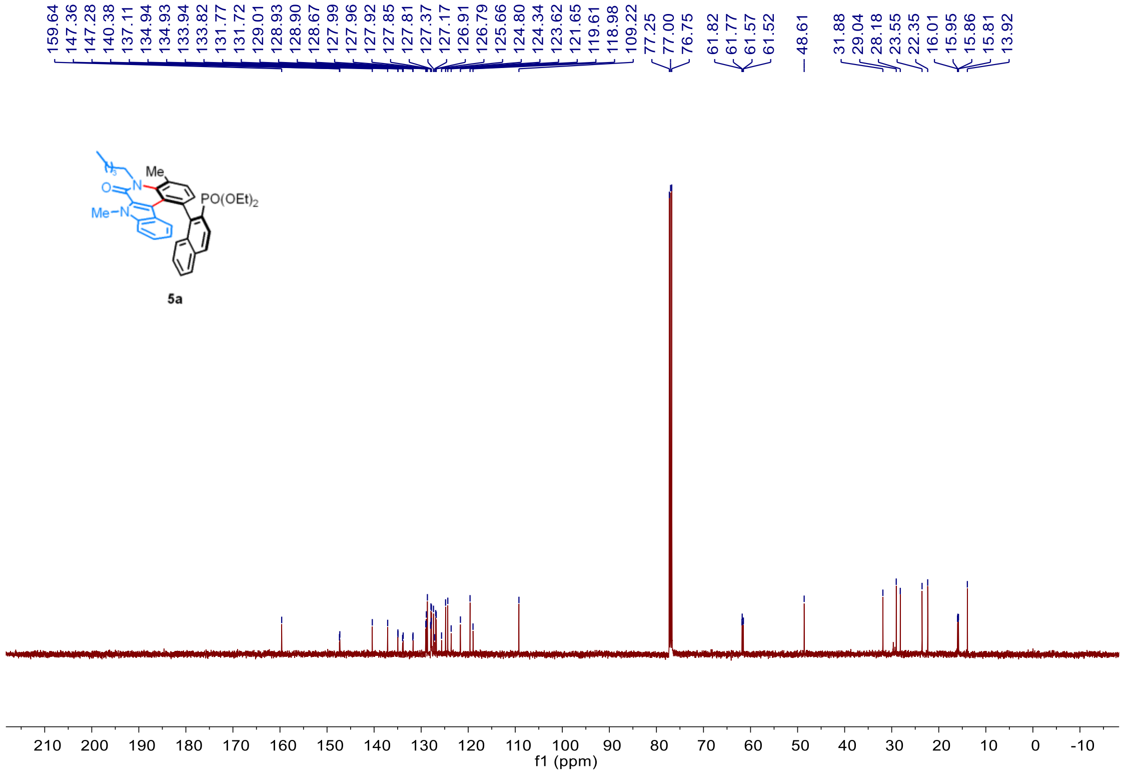
159.64
147.36
147.28
140.38
137.11
134.94
134.93
133.94
133.82
131.77
131.72
129.01
128.93
128.90
128.67
127.99
127.96
127.92
127.85
127.81
127.37
127.17
126.91
126.79
125.66
124.80
124.34
123.62
121.65
119.61
118.98
109.22
77.25
77.00
76.75
61.82
61.77
61.57
61.52
48.61
31.88
29.04
28.18
23.55
22.35
16.01
15.95
15.86
15.81
13.92
Me
O
N
PO(OEt)2
Me–N
5a
210 200 190 180 170 160 150 140 130 120 110 100 90 80 70 60 50 40 30 20 10 0 -10
f1 (ppm)

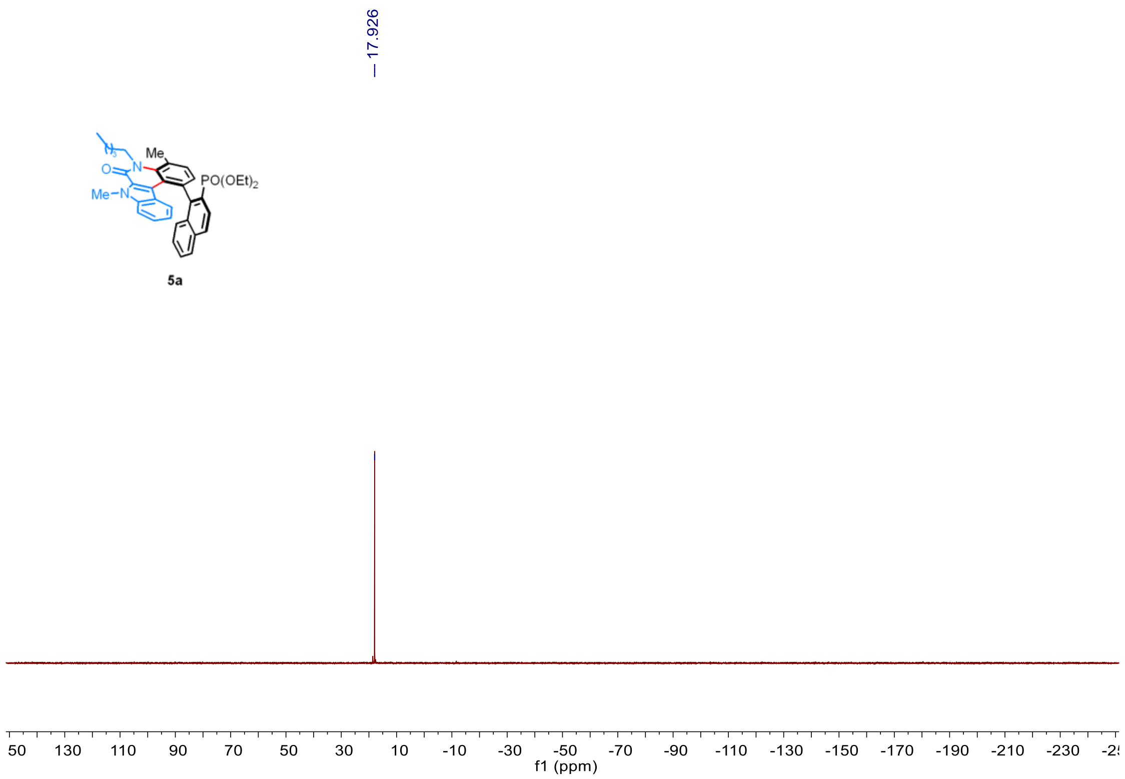
— 17.926
Me
PO(OEt)2
O
N
Me–N
5a
50 130 110 90 70 50 30 10 -10 -30 -50 -70 -90 -110 -130 -150 -170 -190 -210 -230
f1 (ppm)

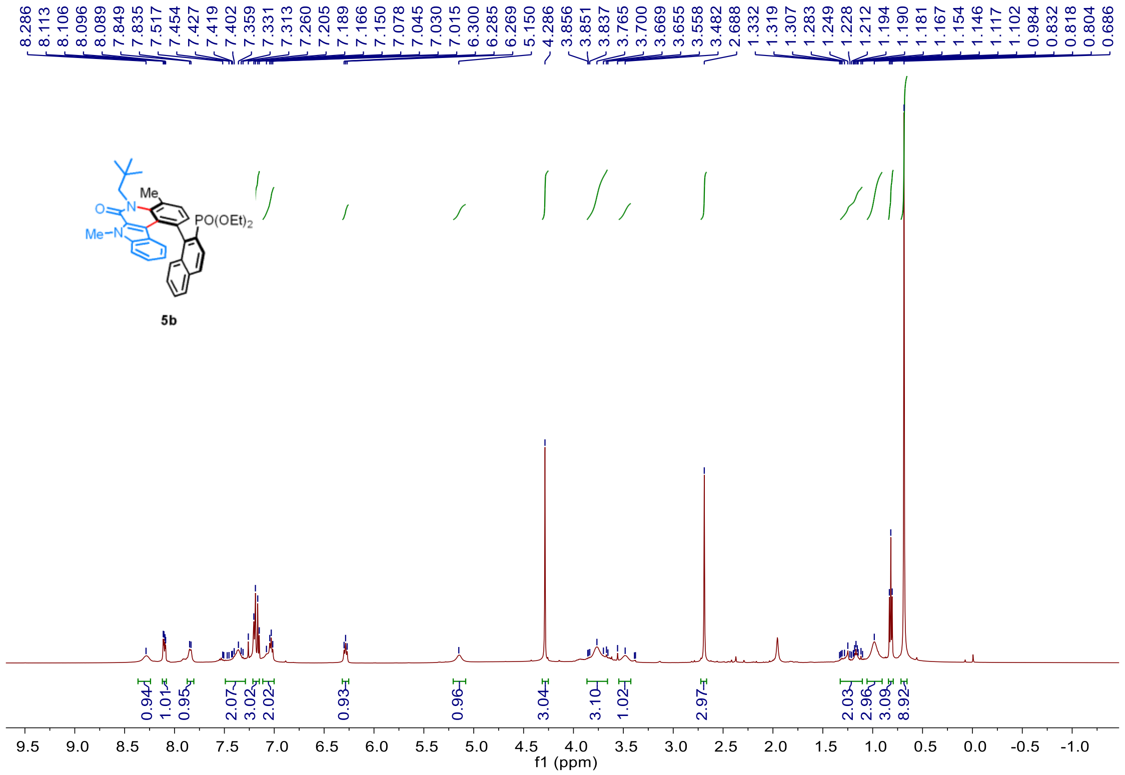
8.286 8.113 8.106 8.096 8.089 7.849 7.835 7.517 7.454 7.427 7.419 7.402 7.359 7.331 7.313 7.260 7.205 7.189 7.166 7.150 7.078 7.045 7.030 7.015 6.300 6.285 6.269 5.150 4.286 3.856 3.851 3.837 3.765 3.700 3.669 3.655 3.558 3.482 2.688 1.332 1.319 1.307 1.283 1.249 1.228 1.212 1.194 1.190 1.181 1.167 1.154 1.146 1.117 1.102 0.984 0.832 0.818 0.804 0.686
Me
PO(OEt)2
O
N
Me–N
5b
0.94 1.01 0.95 2.07 3.02 2.02 0.93 0.96 3.04 3.10 1.02 2.97 2.03 2.96 3.09 8.92
9.5 9.0 8.5 8.0 7.5 7.0 6.5 6.0 5.5 5.0 4.5 4.0 3.5 3.0 2.5 2.0 1.5 1.0 0.5 0.0 -0.5 -1.0
f1 (ppm)

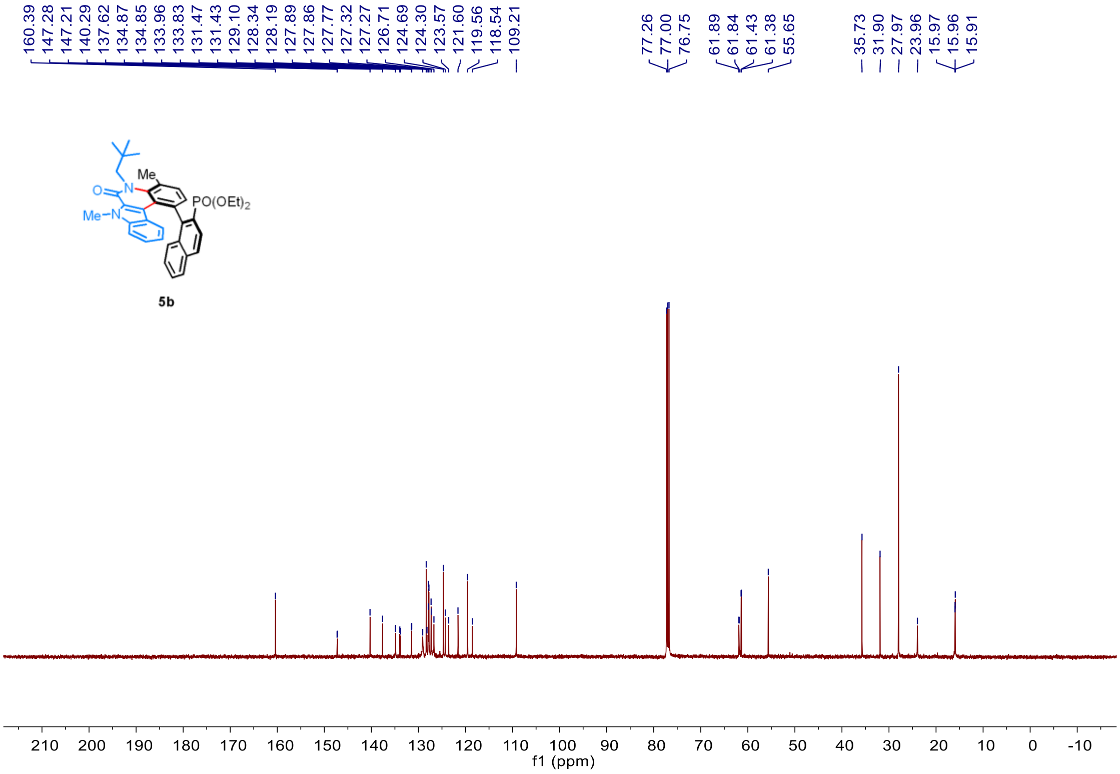
160.39 147.28 147.21 140.29 137.62 134.87 134.85 133.96 133.83 131.47 131.43 129.10 128.34 128.19 127.89 127.86 127.77 127.32 127.27 126.71 124.69 124.30 123.57 121.60 119.56 118.54 109.21
77.26 77.00 76.75
61.89 61.84 61.43 61.38 55.65
35.73 31.90 27.97 23.96 15.97 15.96 15.91
Me
PO(OEt)2
O
N
Me–N
5b
210 200 190 180 170 160 150 140 130 120 110 100 90 80 70 60 50 40 30 20 10 0 -10
f1 (ppm)

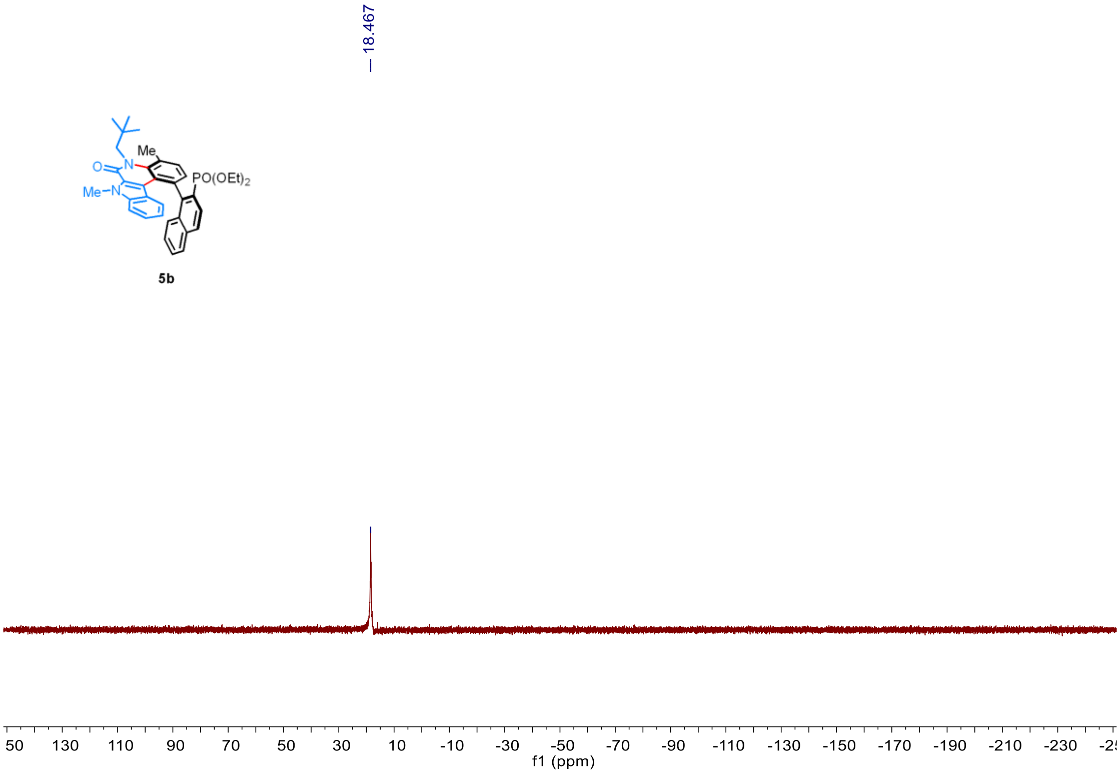
18.467
Me
PO(OEt)2
O
N
Me–N
5b
50 130 110 90 70 50 30 10 -10 -30 -50 -70 -90 -110 -130 -150 -170 -190 -210 -230
f1 (ppm)

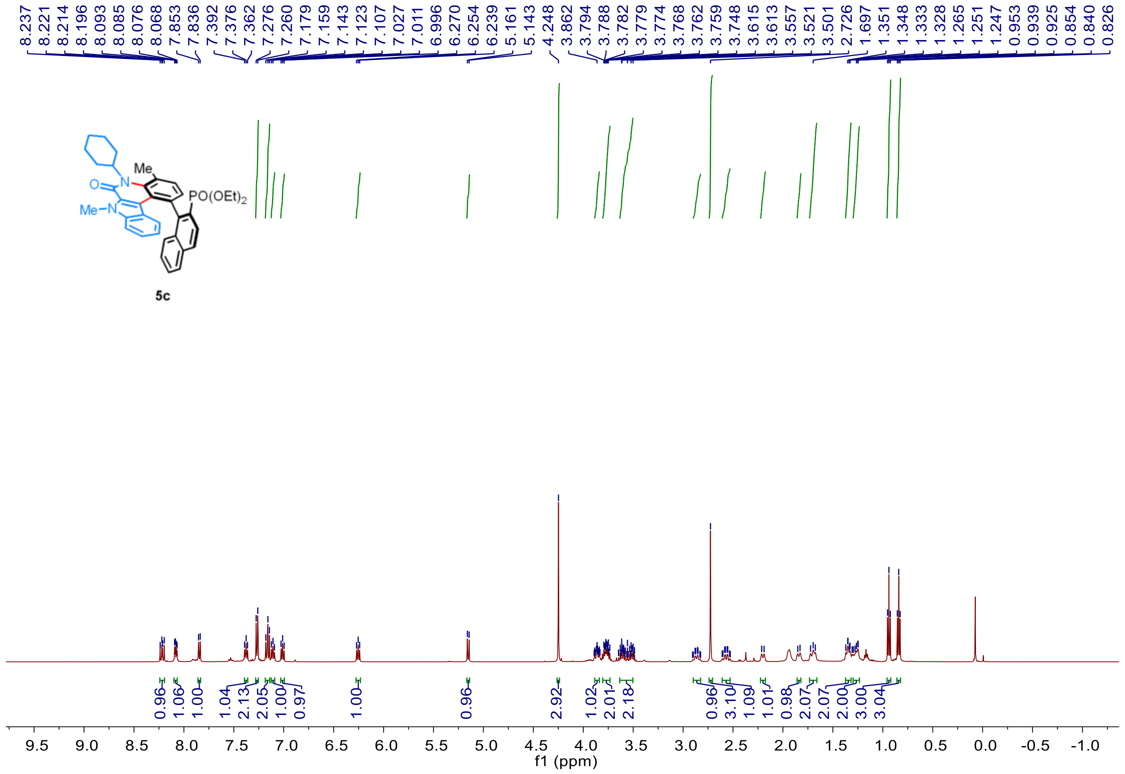
8.237
8.221
8.214
8.196
8.093
8.085
8.076
8.068
7.853
7.836
7.392
7.376
7.362
7.276
7.260
7.179
7.159
7.143
7.123
7.107
7.027
7.011
6.996
6.270
6.254
6.239
5.161
5.143
4.248
3.862
3.794
3.788
3.782
3.779
3.774
3.768
3.762
3.759
3.748
3.615
3.613
3.557
3.521
3.501
2.726
1.697
1.351
1.348
1.333
1.328
1.265
1.251
1.247
0.953
0.939
0.925
0.854
0.840
0.826
Me
O
N
PO(OEt)$_2$
Me
N
5c
0.96
1.06
1.00
1.04
2.13
2.05
1.00
0.97
1.00
0.96
2.92
1.02
2.01
2.18
0.96
3.10
1.09
1.01
0.98
2.07
2.07
2.00
3.00
3.04
9.5
9.0
8.5
8.0
7.5
7.0
6.5
6.0
5.5
5.0
4.5
4.0
3.5
3.0
2.5
2.0
1.5
1.0
0.5
0.0
-0.5
-1.0
f1 (ppm)

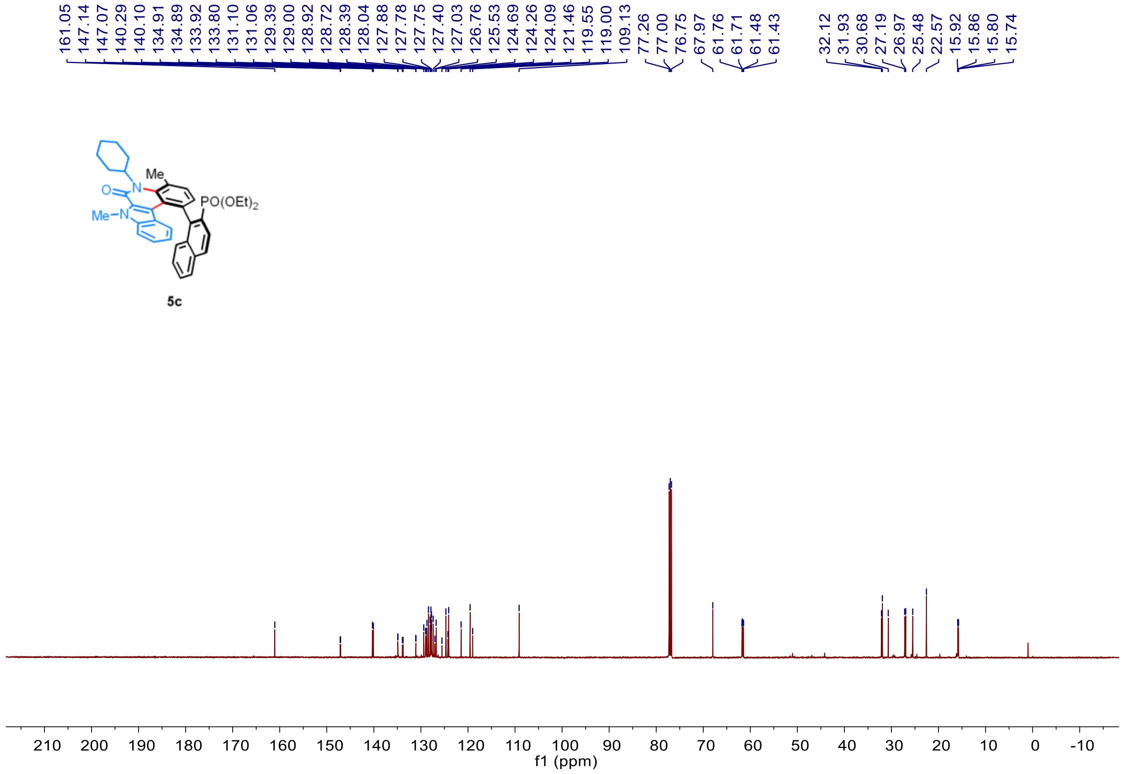
161.05
147.14
147.07
140.29
140.10
134.91
134.89
133.92
133.80
131.10
131.06
129.39
129.00
128.92
128.72
128.39
128.04
127.88
127.78
127.75
127.40
127.03
126.76
125.53
124.69
124.26
124.09
121.46
119.55
119.00
109.13
77.26
77.00
76.75
67.97
61.76
61.71
61.48
61.43
32.12
31.93
30.68
27.19
26.97
25.48
22.57
15.92
15.86
15.80
15.74
Me
O
N
PO(OEt)$_2$
Me
N
5c
210
200
190
180
170
160
150
140
130
120
110
100
90
80
70
60
50
40
30
20
10
0
-10
f1 (ppm)

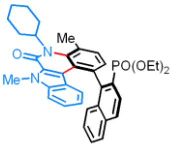

5c

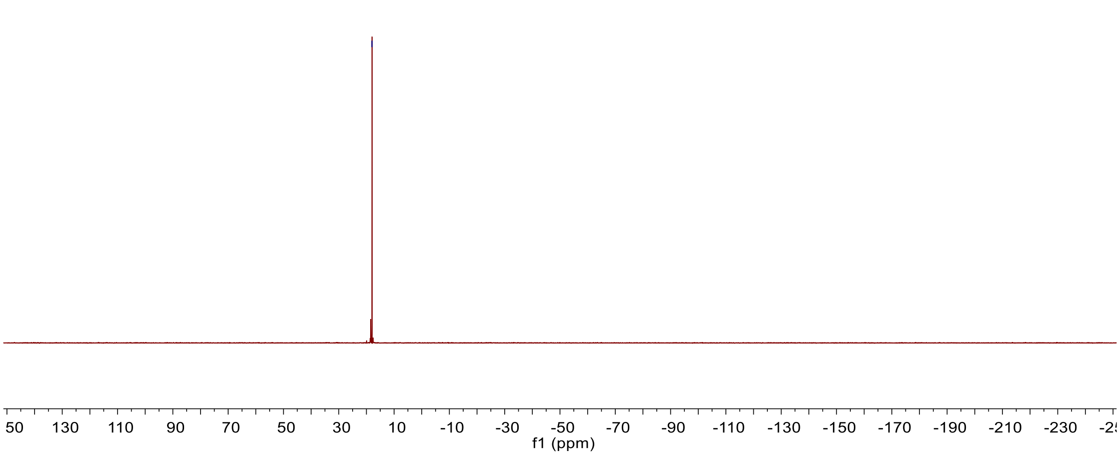

17.987
50 130 110 90 70 50 30 10 -10 -30 -50 -70 -90 -110 -130 -150 -170 -190 -210 -230
f1 (ppm)

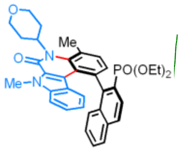

5d

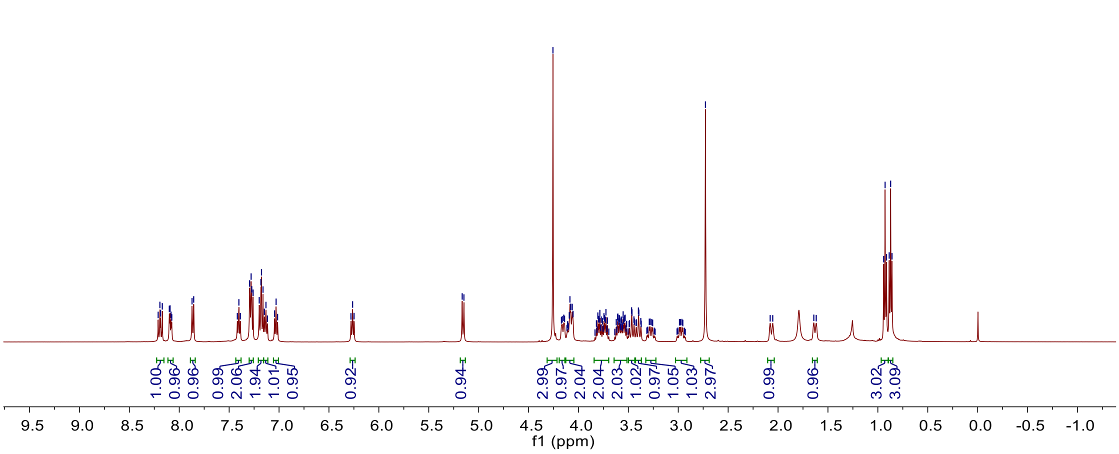

8.211 8.194 8.187 8.170 8.099 8.092 8.082 8.075 7.872 7.855 7.418 7.402 7.388 7.295 7.280 7.274 7.260 7.199 7.182 7.176 7.161 7.147 7.131 7.045 7.029 7.015 6.279 6.263 6.248 5.165 5.148 4.255 4.147 4.092 4.085 4.078 4.062 4.054 3.806 3.786 3.744 3.724 3.607 3.605 3.552 3.532 3.468 3.464 3.399 3.395 2.727 1.642 1.617 0.941 0.927 0.913 0.886 0.872 0.858
1.00 0.96 0.96 0.99 2.06 1.94 1.01 0.95 0.92 0.94 2.99 0.97 2.04 2.04 2.03 1.02 0.97 1.05 1.03 2.97 0.99 0.96 3.02 3.09
9.5 9.0 8.5 8.0 7.5 7.0 6.5 6.0 5.5 5.0 4.5 4.0 3.5 3.0 2.5 2.0 1.5 1.0 0.5 0.0 -0.5 -1.0
f1 (ppm)

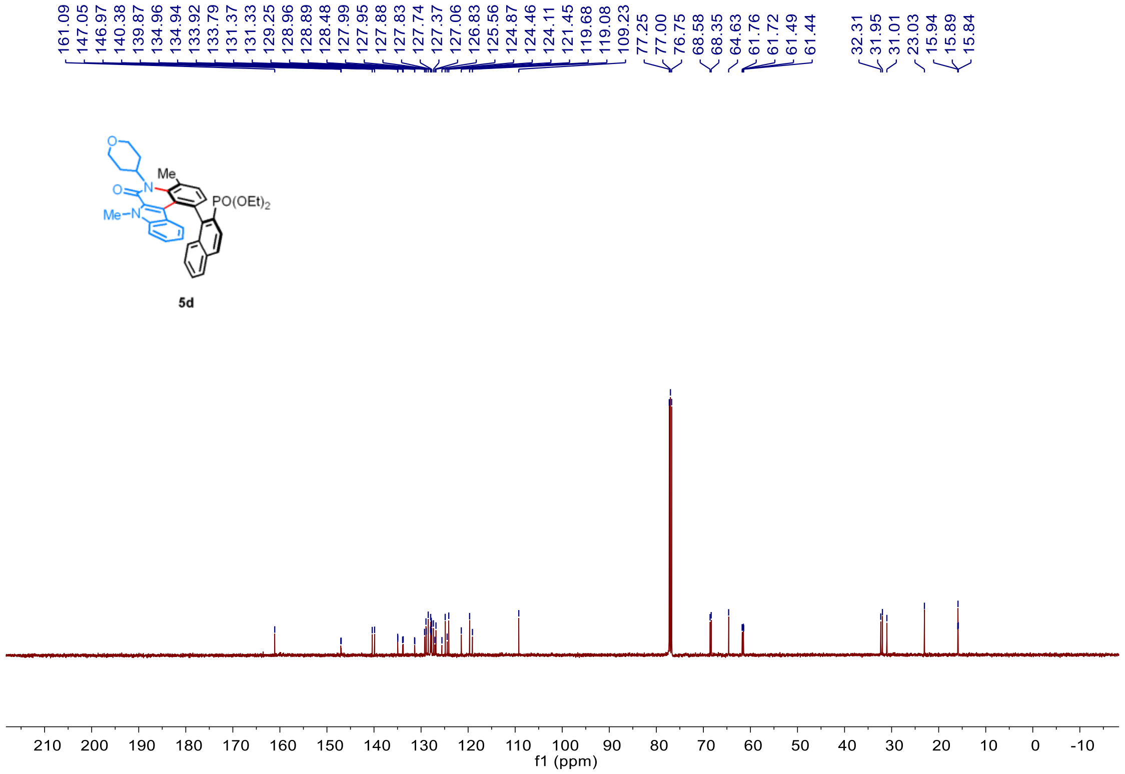
161.09
147.05
146.97
140.38
139.87
134.96
134.94
133.92
133.79
131.37
131.33
129.25
128.96
128.89
128.48
127.99
127.95
127.88
127.83
127.74
127.37
127.06
126.83
125.56
124.87
124.46
124.11
121.45
119.68
119.08
109.23
77.25
77.00
76.75
68.58
68.35
64.63
61.76
61.72
61.49
61.44
32.31
31.95
31.01
23.03
15.94
15.89
15.84
O
N
Me
O
Me
N
PO(OEt)2
5d
210 200 190 180 170 160 150 140 130 120 110 100 90 80 70 60 50 40 30 20 10 0 -10
f1 (ppm)

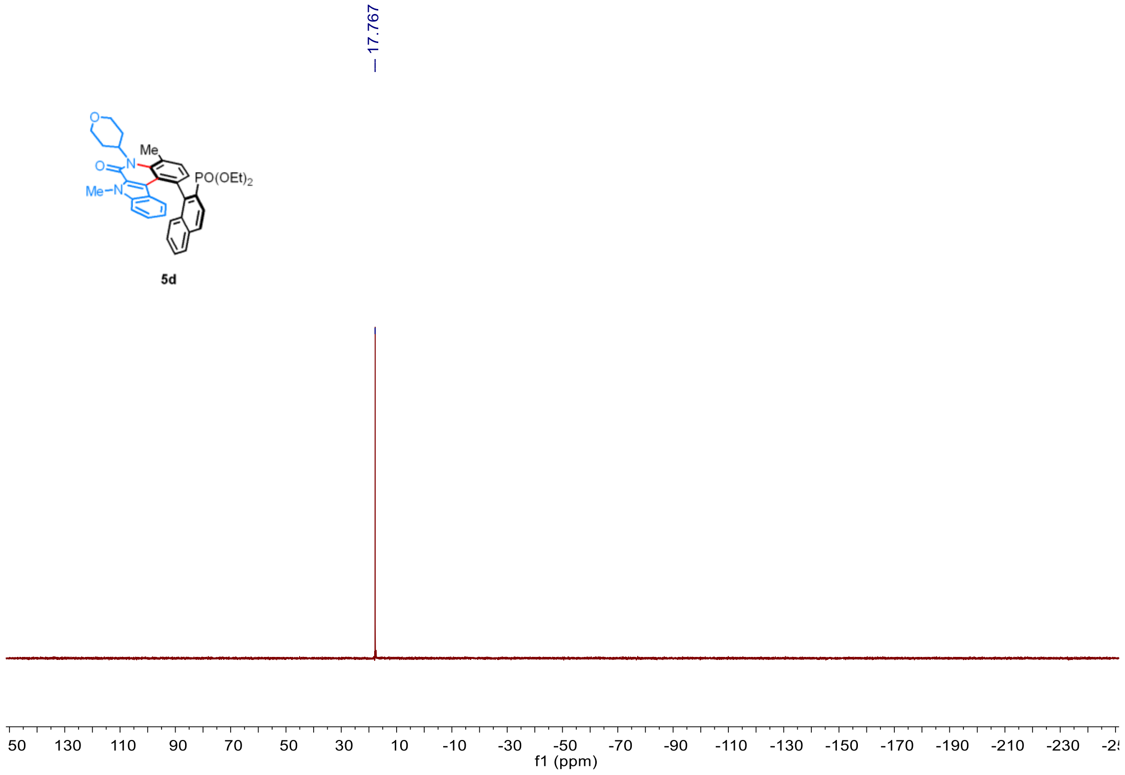
17.767
O
N
Me
O
Me
N
PO(OEt)2
5d
50 130 110 90 70 50 30 10 -10 -30 -50 -70 -90 -110 -130 -150 -170 -190 -210 -230
f1 (ppm)

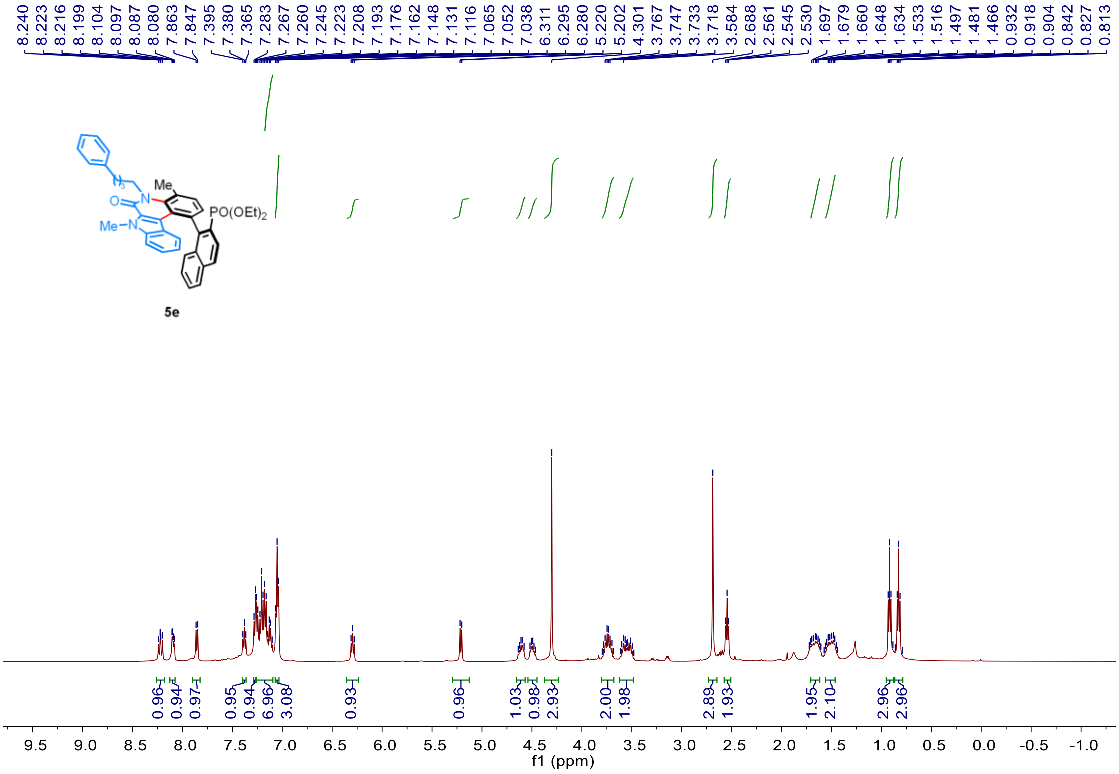
8.240 8.223 8.216 8.199 8.104 8.097 8.087 8.080 7.863 7.847 7.395 7.380 7.365 7.283 7.267 7.260 7.245 7.223 7.208 7.193 7.176 7.162 7.148 7.131 7.116 7.065 7.052 7.038 6.311 6.295 6.280 5.220 5.202 4.301 3.767 3.747 3.733 3.718 3.584 2.688 2.561 2.545 2.530 1.697 1.679 1.660 1.648 1.634 1.533 1.516 1.497 1.481 1.466 0.932 0.918 0.904 0.842 0.827 0.813
Me
O
N
Me–N
PO(OEt)2
5e
0.96 0.94 0.97 0.95 0.94 6.96 3.08 0.93 0.96 1.03 0.98 2.93 2.00 1.98 2.89 1.93 1.95 2.10 2.96 2.96
9.5 9.0 8.5 8.0 7.5 7.0 6.5 6.0 5.5 5.0 4.5 4.0 3.5 3.0 2.5 2.0 1.5 1.0 0.5 0.0 -0.5 -1.0
f1 (ppm)

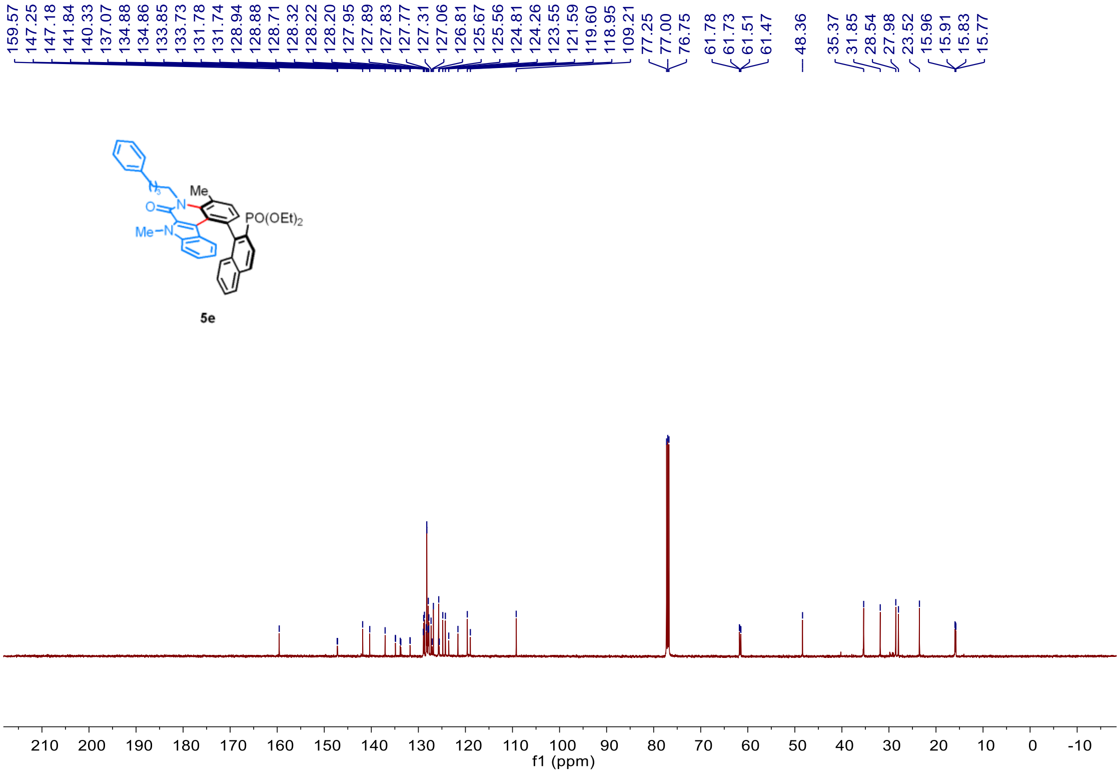
159.57 147.25 147.18 141.84 140.33 137.07 134.88 134.86 133.85 133.73 131.78 131.74 128.94 128.88 128.71 128.32 128.22 128.20 127.95 127.89 127.83 127.77 127.31 127.06 126.81 125.67 125.56 124.81 124.26 123.55 121.59 119.60 118.95 109.21 77.25 77.00 76.75 61.78 61.73 61.51 61.47 48.36 35.37 31.85 28.54 27.98 23.52 15.96 15.91 15.83 15.77
Me
O
N
Me–N
PO(OEt)2
5e
210 200 190 180 170 160 150 140 130 120 110 100 90 80 70 60 50 40 30 20 10 0 -10
f1 (ppm)

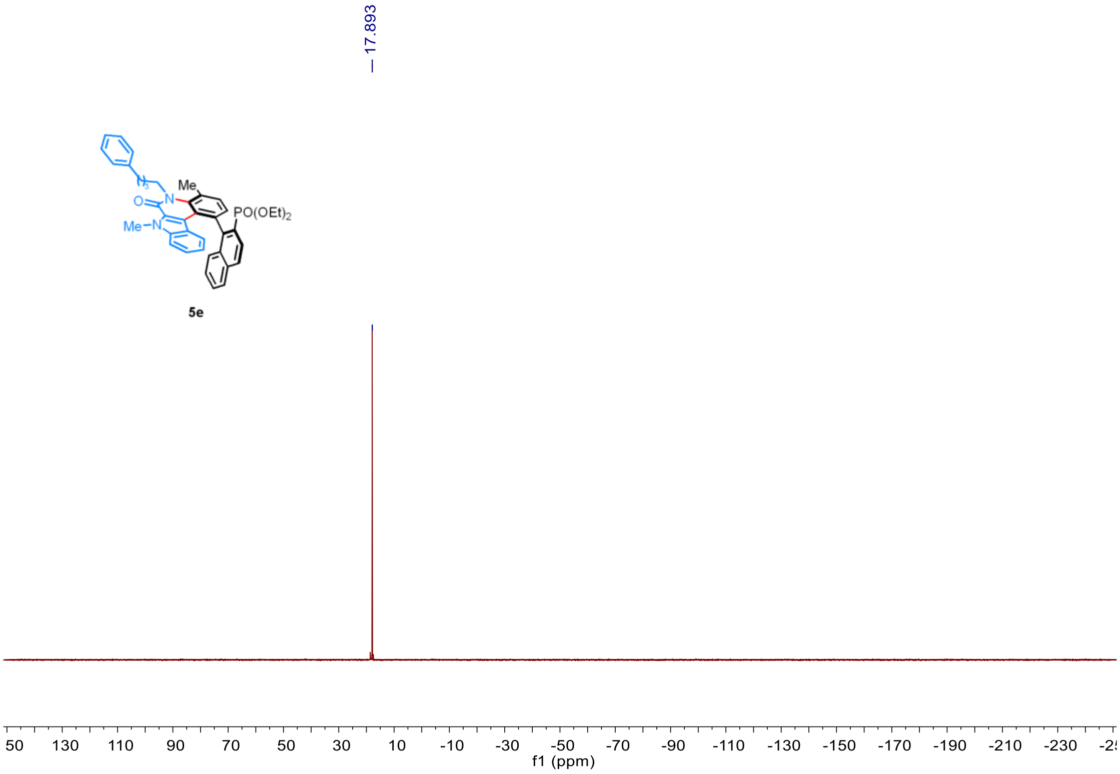

17.893
PO(OEt)2
Me
O
N
Me–N
5e
50 130 110 90 70 50 30 10 -10 -30 -50 -70 -90 -110 -130 -150 -170 -190 -210 -230
f1 (ppm)

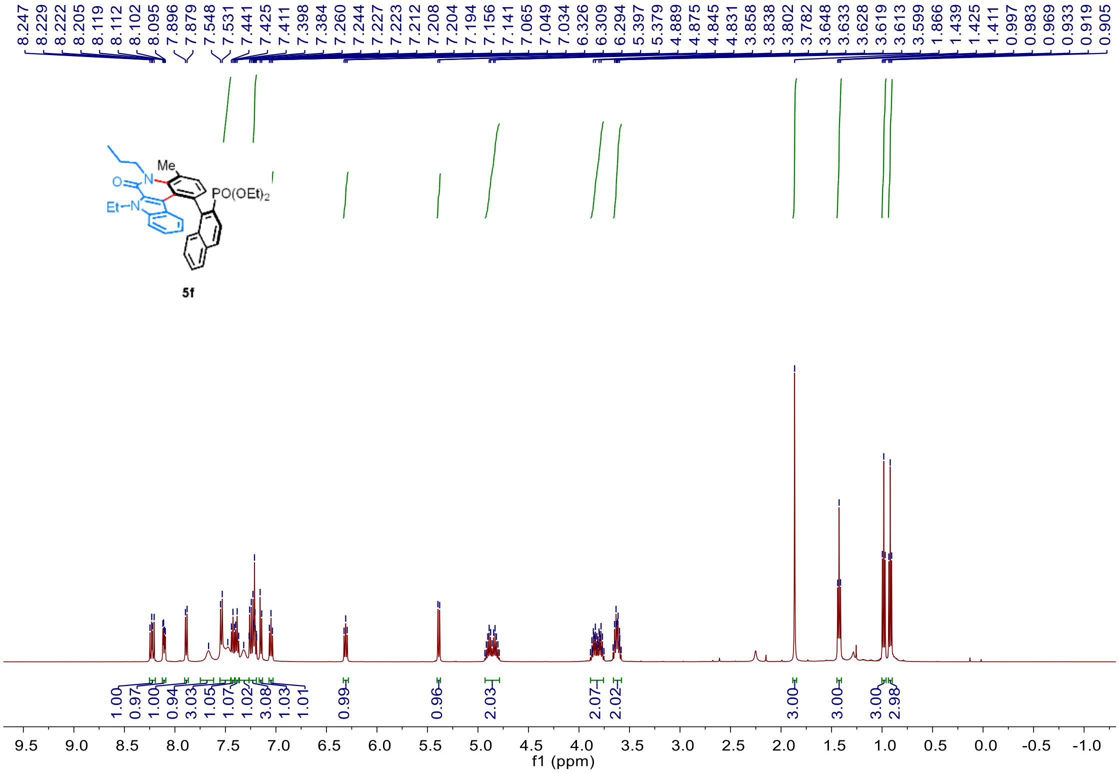

8.247 8.229 8.222 8.205 8.119 8.112 8.102 8.095 7.896 7.879 7.548 7.531 7.441 7.425 7.411 7.398 7.384 7.260 7.244 7.227 7.223 7.212 7.208 7.204 7.194 7.156 7.141 7.065 7.049 7.034 6.326 6.309 6.294 5.397 5.379 4.889 4.875 4.845 4.831 3.858 3.838 3.802 3.782 3.648 3.633 3.628 3.619 3.613 3.599 1.866 1.439 1.425 1.411 0.997 0.983 0.969 0.933 0.919 0.905
PO(OEt)2
Me
O
N
Et–N
5f
1.00 0.97 1.00 0.94 3.03 1.05 1.07 1.02 3.08 1.03 1.01 0.99 0.96 2.03 2.07 2.02 3.00 3.00 3.00 2.98
9.5 9.0 8.5 8.0 7.5 7.0 6.5 6.0 5.5 5.0 4.5 4.0 3.5 3.0 2.5 2.0 1.5 1.0 0.5 0.0 -0.5 -1.0
f1 (ppm)

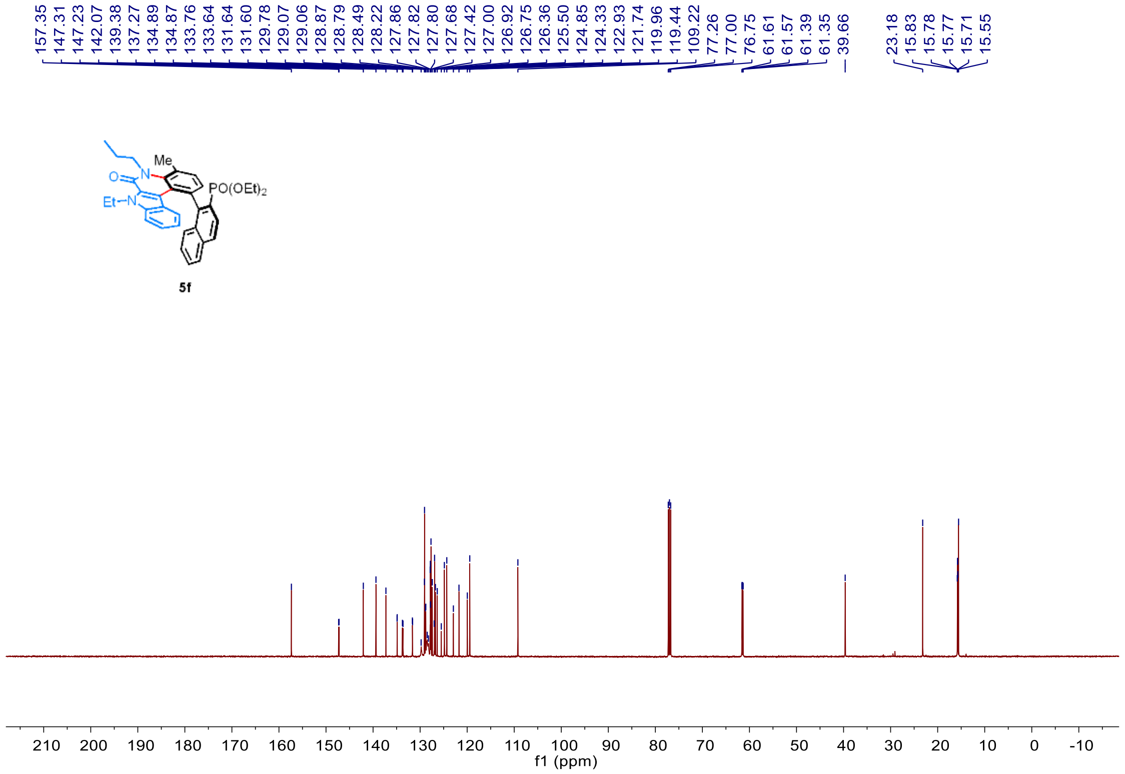
157.35
147.31
147.23
142.07
139.38
137.27
134.89
134.87
133.76
133.64
131.64
131.60
129.78
129.07
129.06
128.87
128.79
128.49
128.22
127.86
127.82
127.80
127.68
127.42
127.00
126.92
126.75
126.36
125.50
124.85
124.33
122.93
121.74
119.96
119.44
109.22
77.26
77.00
76.75
61.61
61.57
61.39
61.35
39.66
23.18
15.83
15.78
15.77
15.71
15.55
Me
O
N
PO(OEt)2
Et
5f
210 200 190 180 170 160 150 140 130 120 110 100 90 80 70 60 50 40 30 20 10 0 -10
f1 (ppm)

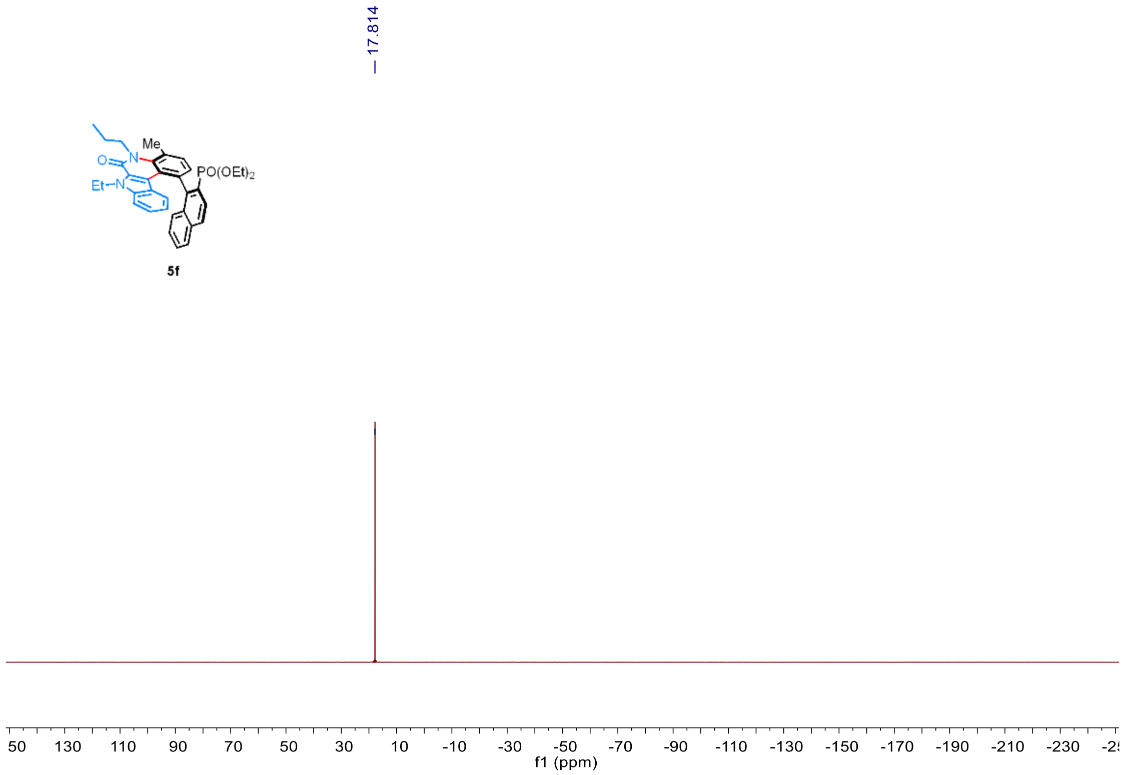
17.814
Me
O
N
PO(OEt)2
Et
5f
50 130 110 90 70 50 30 10 -10 -30 -50 -70 -90 -110 -130 -150 -170 -190 -210 -230
f1 (ppm)

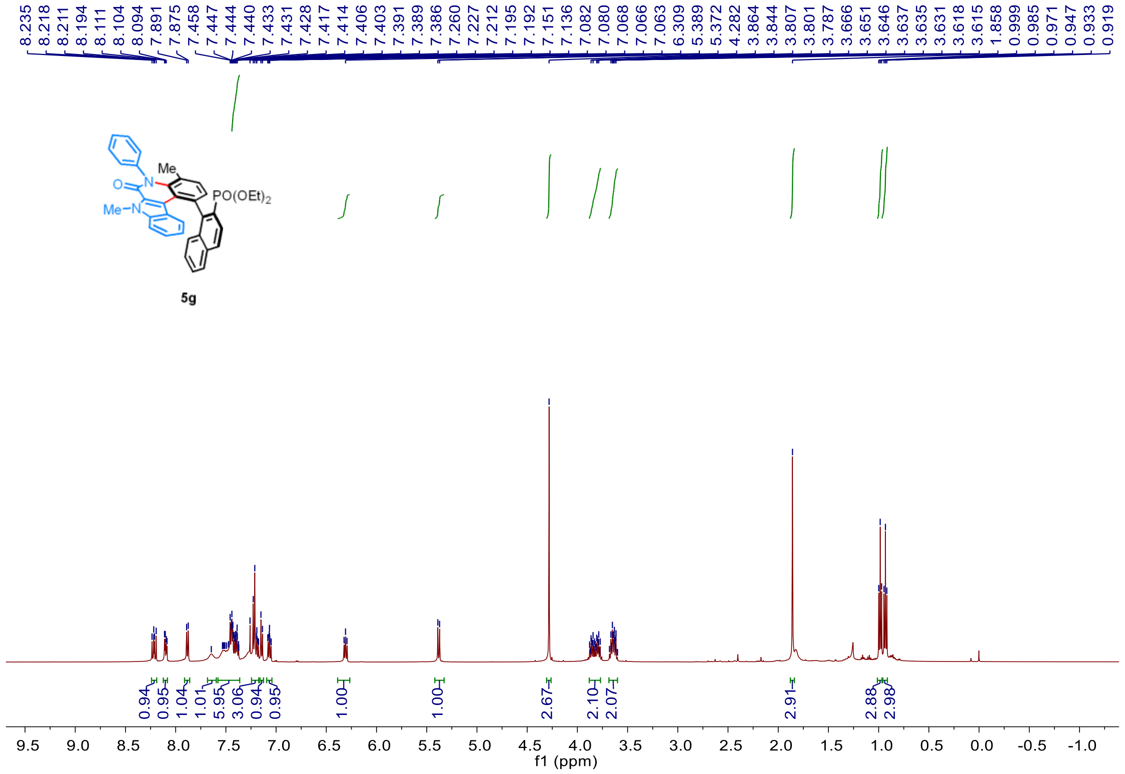
8.235 8.218 8.211 8.194 8.111 8.104 8.094 7.891 7.875 7.458 7.447 7.444 7.440 7.433 7.431 7.428 7.417 7.414 7.406 7.403 7.391 7.389 7.386 7.260 7.227 7.212 7.195 7.192 7.151 7.136 7.082 7.080 7.068 7.066 7.063 6.309 5.389 5.372 4.282 3.864 3.844 3.807 3.801 3.787 3.666 3.651 3.646 3.637 3.635 3.631 3.618 3.615 1.858 0.999 0.985 0.971 0.947 0.933 0.919
Me
PO(OEt)2
O
N
Me
5g
0.94 0.95 1.04 1.01 5.95 3.06 0.94 0.95 1.00 1.00 2.67 2.10 2.07 2.91 2.88 2.98
9.5 9.0 8.5 8.0 7.5 7.0 6.5 6.0 5.5 5.0 4.5 4.0 3.5 3.0 2.5 2.0 1.5 1.0 0.5 0.0 -0.5 -1.0
f1 (ppm)

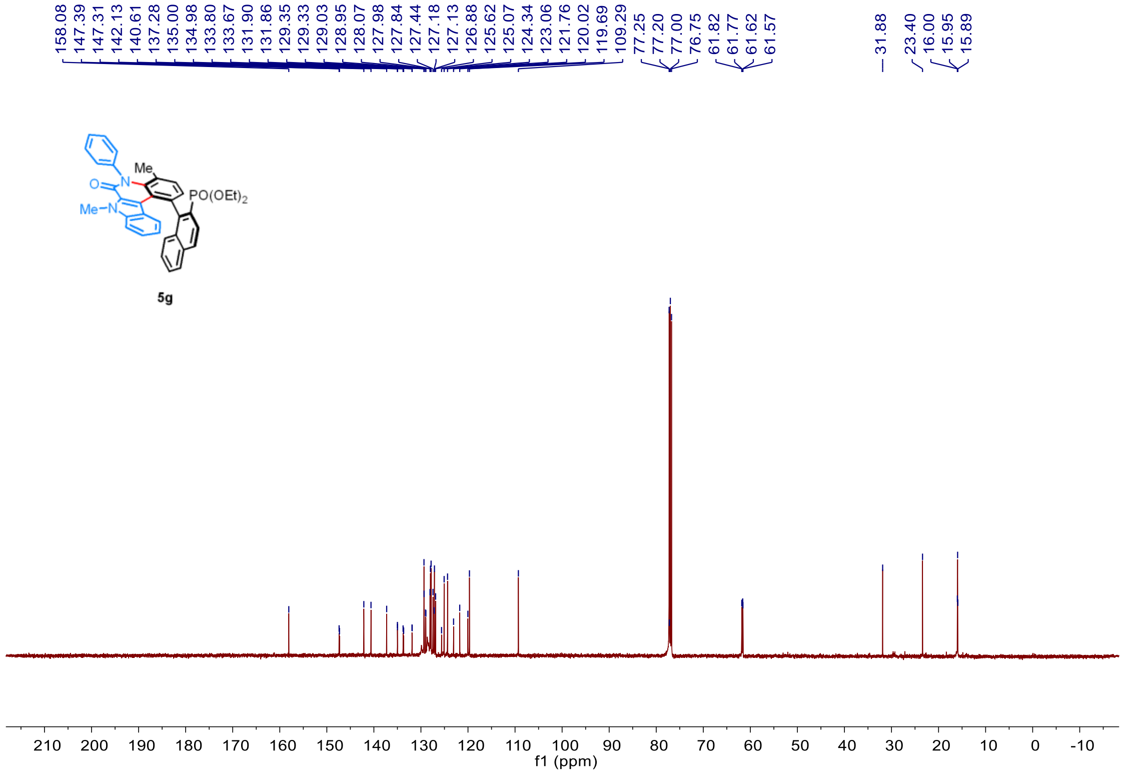
158.08 147.39 147.31 142.13 140.61 137.28 135.00 134.98 133.80 133.67 131.90 131.86 129.35 129.33 129.03 128.95 128.07 127.98 127.84 127.44 127.18 127.13 126.88 125.62 125.07 124.34 123.06 121.76 120.02 119.69 109.29 77.25 77.20 77.00 76.75 61.82 61.77 61.62 61.57 31.88 23.40 16.00 15.95 15.89
Me
PO(OEt)2
O
N
Me
5g
210 200 190 180 170 160 150 140 130 120 110 100 90 80 70 60 50 40 30 20 10 0 -10
f1 (ppm)

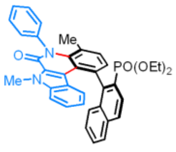
Me
PO(OEt)2
O
N
Me–N

5g

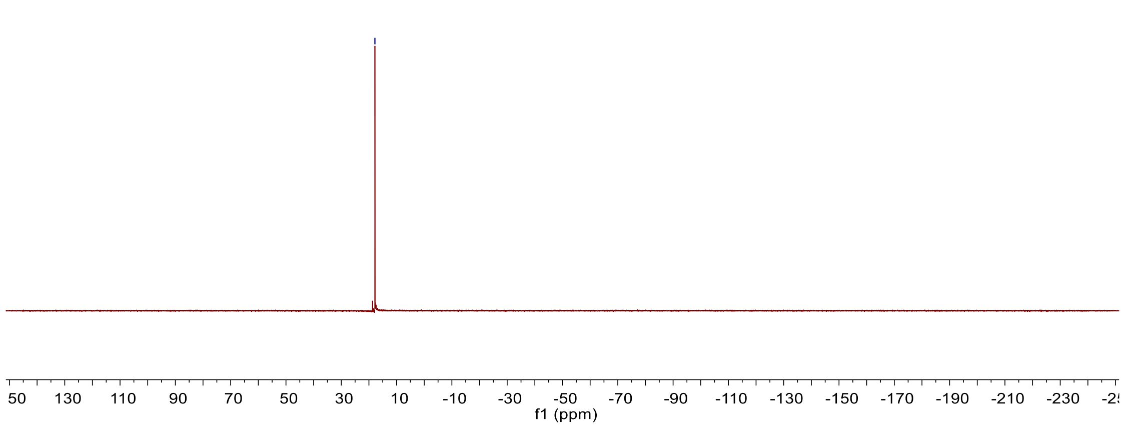
17.832
50 130 110 90 70 50 30 10 -10 -30 -50 -70 -90 -110 -130 -150 -170 -190 -210 -230
f1 (ppm)

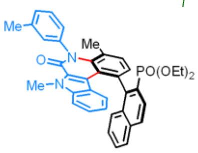
Me
Me
PO(OEt)2
O
N
Me–N

5h

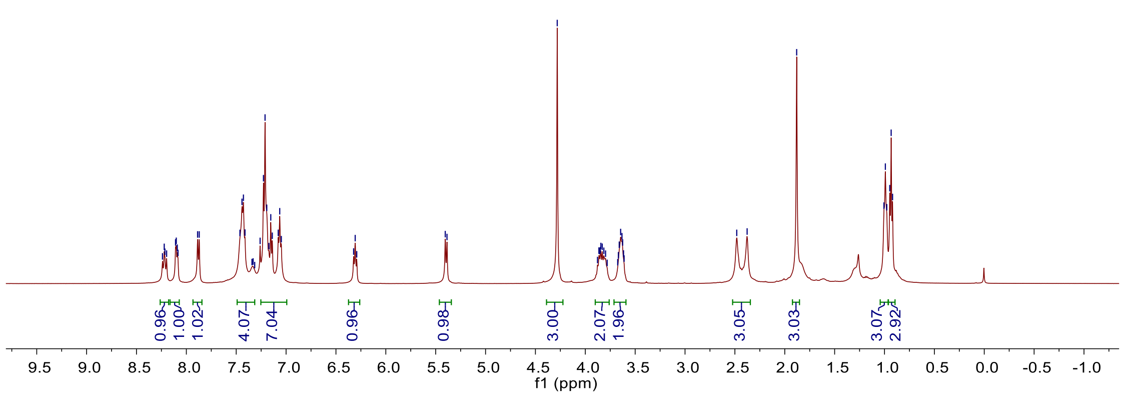
8.239 8.222 8.214 8.198 8.108 8.101 8.091 8.084 7.887 7.871 7.463 7.442 7.428 7.412 7.342 7.330 7.260 7.227 7.211 7.194 7.172 7.153 7.138 7.079 7.064 7.048 6.323 6.307 6.292 5.404 5.387 4.281 3.878 3.864 3.858 3.850 3.844 3.830 3.814 3.797 3.781 3.675 3.668 3.660 3.646 3.632 3.625 3.617 3.611 2.481 2.378 1.881 1.006 0.992 0.977 0.947 0.933
0.96 1.00 1.02 4.07 7.04 0.96 0.98 3.00 2.07 1.96 3.05 3.03 3.07 2.92
9.5 9.0 8.5 8.0 7.5 7.0 6.5 6.0 5.5 5.0 4.5 4.0 3.5 3.0 2.5 2.0 1.5 1.0 0.5 0.0 -0.5 -1.0
f1 (ppm)

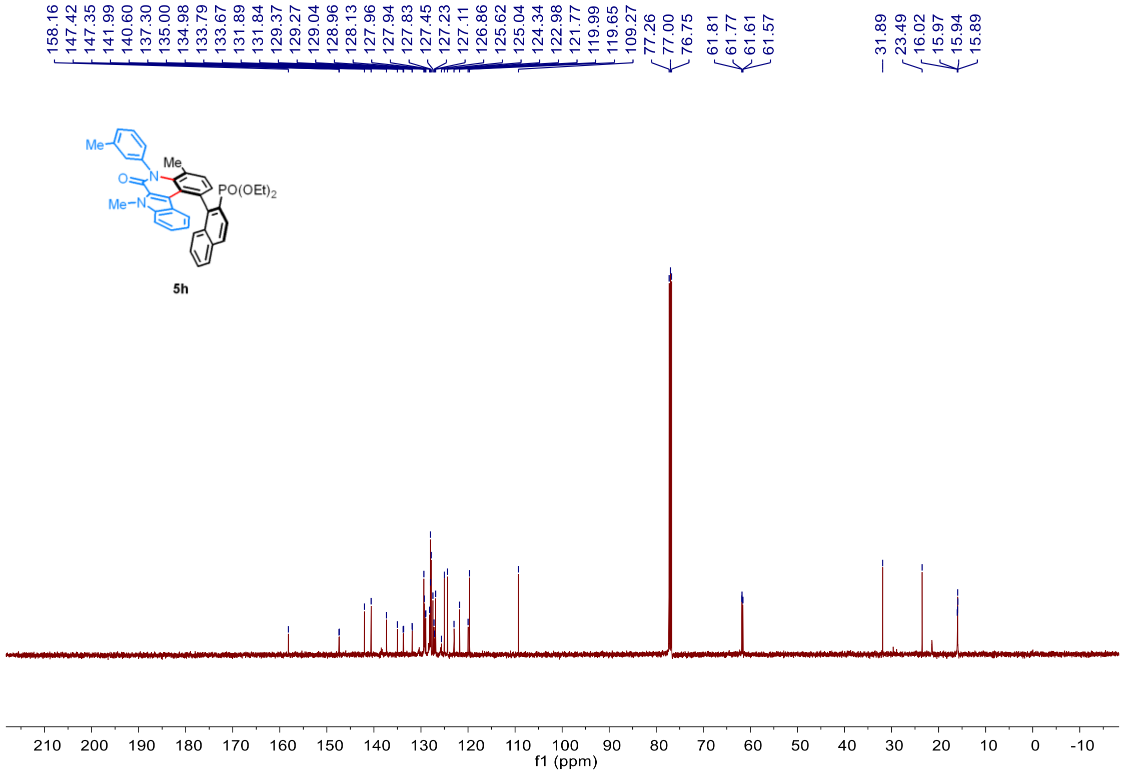
158.16
147.42
147.35
141.99
140.60
137.30
135.00
134.98
133.79
133.67
131.89
131.84
129.37
129.27
129.04
128.96
128.13
127.96
127.94
127.83
127.45
127.23
127.11
126.86
125.62
125.04
124.34
122.98
121.77
119.99
119.65
109.27
77.26
77.00
76.75
61.81
61.77
61.61
61.57
31.89
23.49
16.02
15.97
15.94
15.89
Me
Me
O
N
Me
N
PO(OEt)2
5h
f1 (ppm)

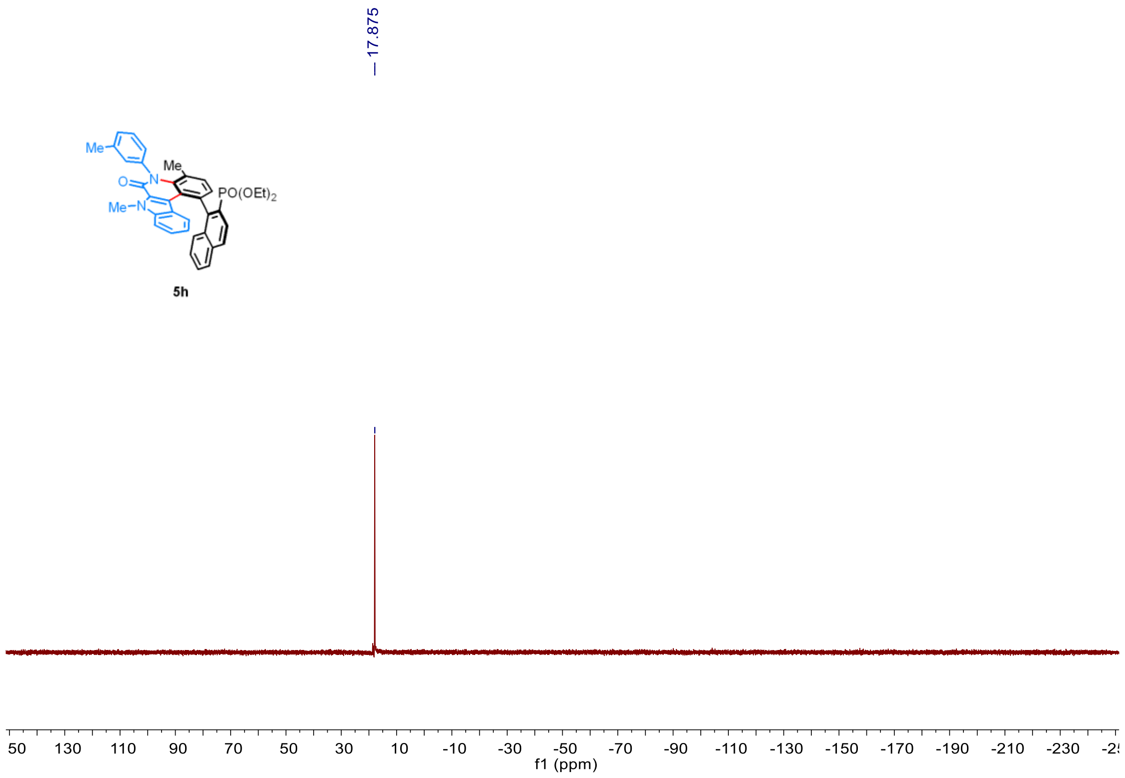
17.875
Me
Me
O
N
Me
N
PO(OEt)2
5h
f1 (ppm)

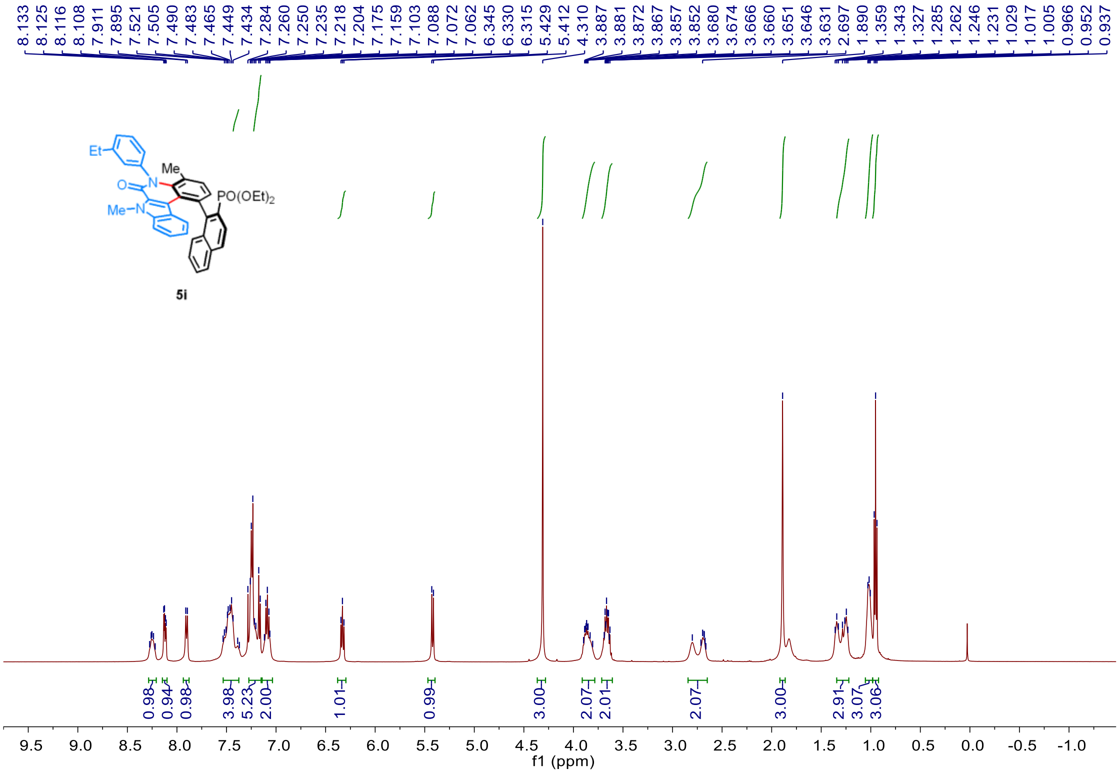
8.133
8.125
8.116
8.108
7.911
7.895
7.521
7.505
7.490
7.483
7.465
7.449
7.434
7.284
7.260
7.250
7.235
7.218
7.204
7.175
7.159
7.103
7.088
7.072
7.062
6.345
6.330
6.315
5.429
5.412
4.310
3.887
3.881
3.872
3.867
3.857
3.852
3.680
3.674
3.666
3.660
3.651
3.646
3.631
2.697
1.890
1.359
1.343
1.327
1.285
1.262
1.246
1.231
1.029
1.017
1.005
0.966
0.952
0.937
Et
O
N
Me
PO(OEt)$_2$
Me–N
5i
0.98
0.94
0.98
3.98
5.23
2.00
1.01
0.99
3.00
2.07
2.01
2.07
3.00
2.91
3.07
3.06
9.5
9.0
8.5
8.0
7.5
7.0
6.5
6.0
5.5
5.0
4.5
4.0
3.5
3.0
2.5
2.0
1.5
1.0
0.5
0.0
-0.5
-1.0
f1 (ppm)

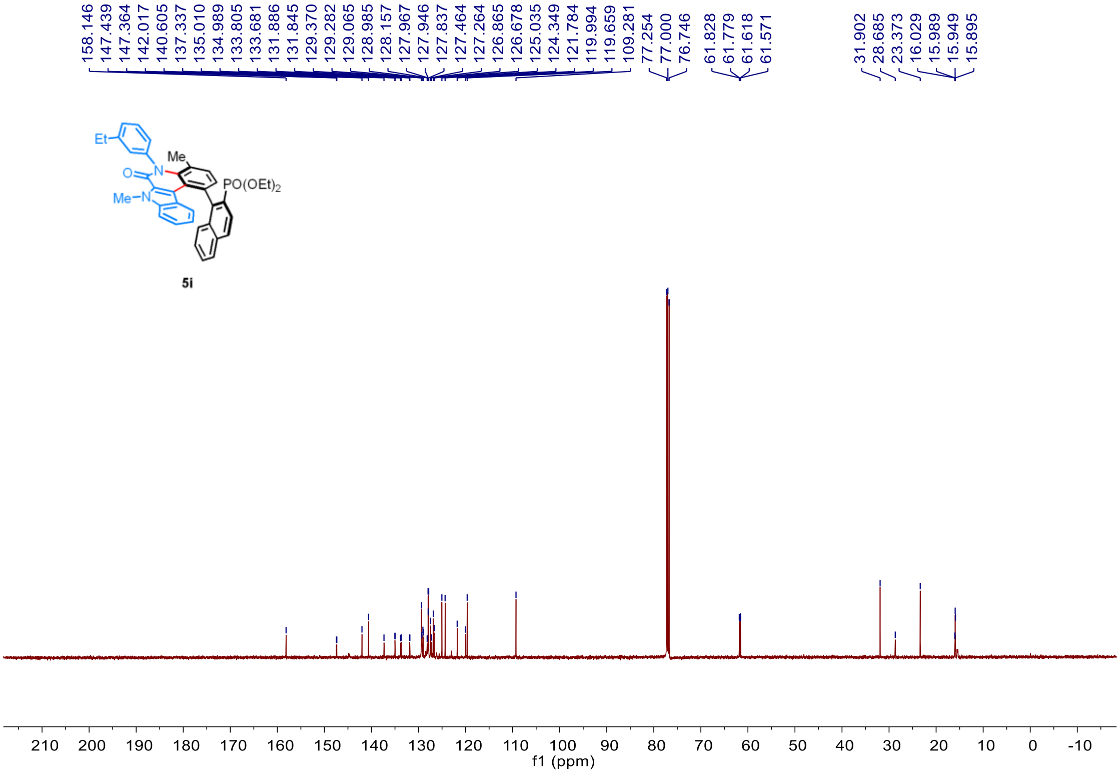
158.146
147.439
147.364
142.017
140.605
137.337
135.010
134.989
133.805
133.681
131.886
131.845
129.370
129.282
129.065
128.985
128.157
127.967
127.946
127.837
127.464
127.264
126.865
126.678
125.035
124.349
121.784
119.994
119.659
109.281
77.254
77.000
76.746
61.828
61.779
61.618
61.571
31.902
28.685
23.373
16.029
15.989
15.949
15.895
Et
O
N
Me
PO(OEt)$_2$
Me–N
5i
210
200
190
180
170
160
150
140
130
120
110
100
90
80
70
60
50
40
30
20
10
0
-10
f1 (ppm)

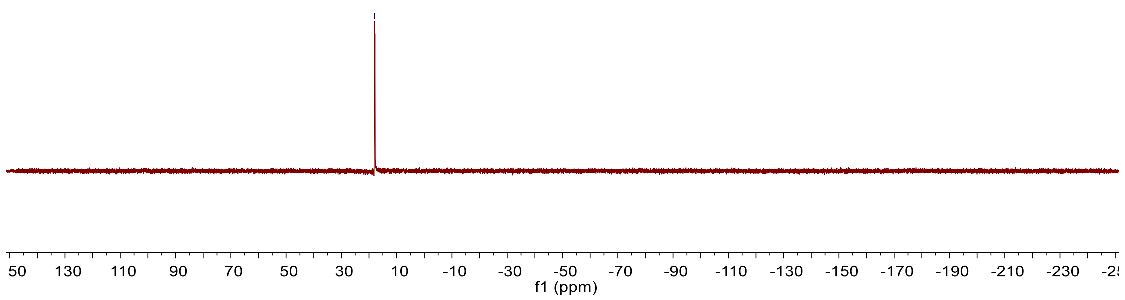
18.007
50 130 110 90 70 50 30 10 -10 -30 -50 -70 -90 -110 -130 -150 -170 -190 -210 -230
f1 (ppm)

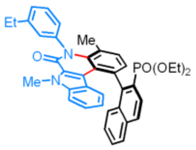
Et
N
O
Me
Me–N
PO(OEt)$_2$

5i

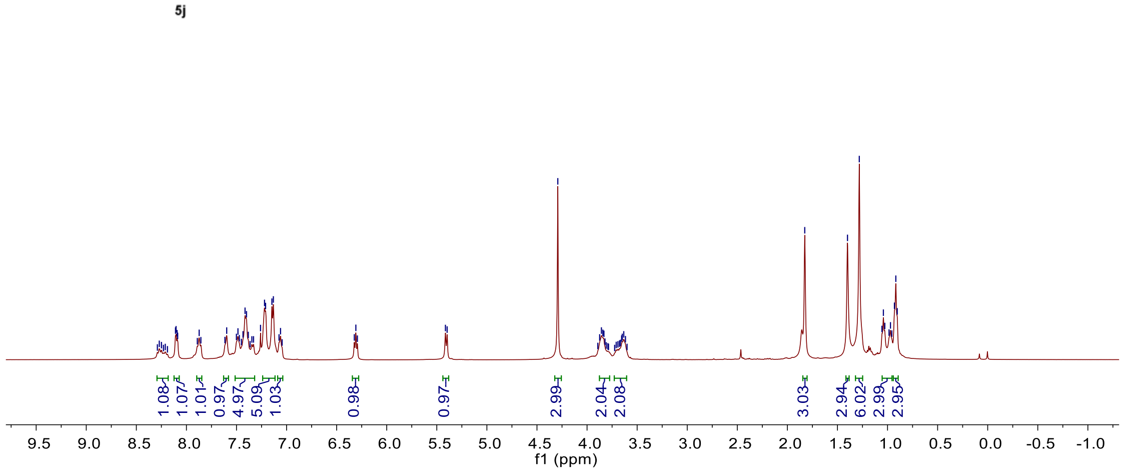
8.271 8.109 8.103 8.093 8.087 7.891 7.873 7.856 7.615 7.598 7.501 7.485 7.472 7.451 7.435 7.415 7.400 7.382 7.365 7.347 7.330 7.260 7.220 7.210 7.146 7.132 7.075 7.060 7.044 6.324 6.308 6.293 5.414 5.397 4.292 3.874 3.857 3.842 3.832 3.821 3.692 3.676 3.667 3.655 3.648 3.634 3.617 1.827 1.400 1.282 1.056 1.041 1.027 0.985 0.971 0.957 0.931 0.917 0.903
1.08 1.07 1.01 0.97 4.97 5.09 1.03 0.98 0.97 2.99 2.04 2.08 3.03 2.94 6.02 2.99 2.95
9.5 9.0 8.5 8.0 7.5 7.0 6.5 6.0 5.5 5.0 4.5 4.0 3.5 3.0 2.5 2.0 1.5 1.0 0.5 0.0 -0.5 -1.0
f1 (ppm)

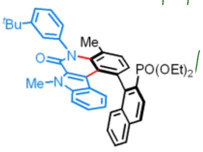
^{t}Bu
N
O
Me
Me–N
PO(OEt)$_2$

5j

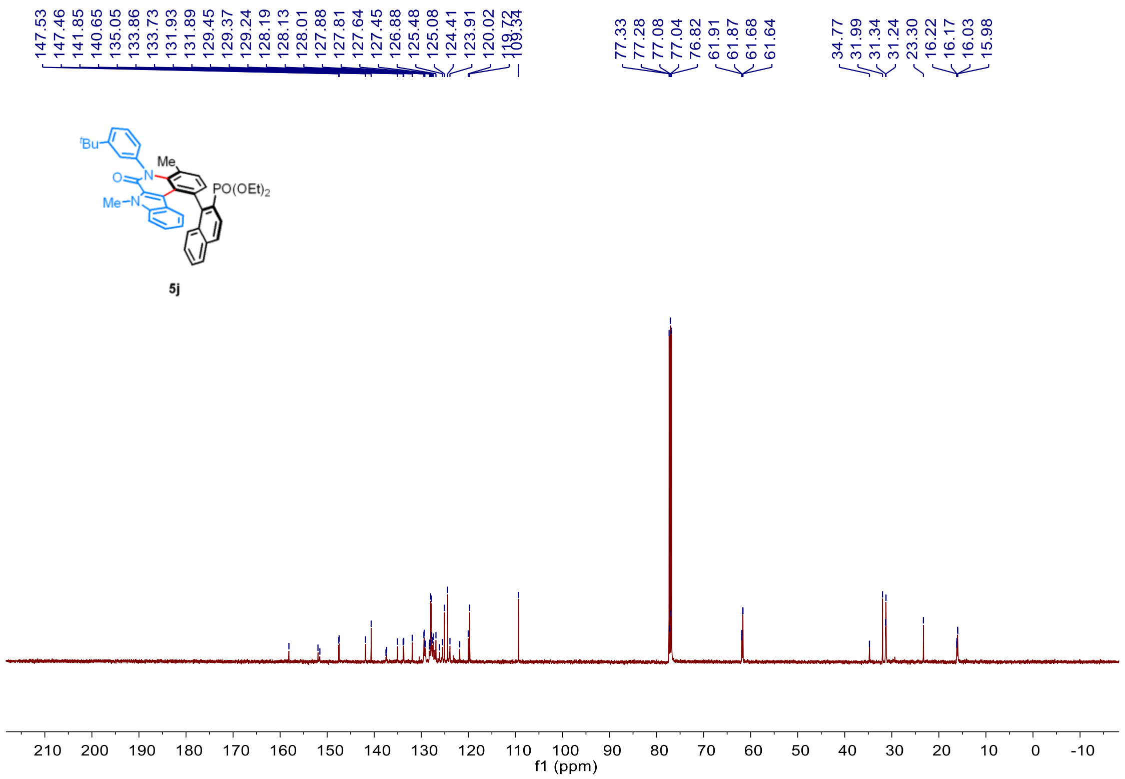

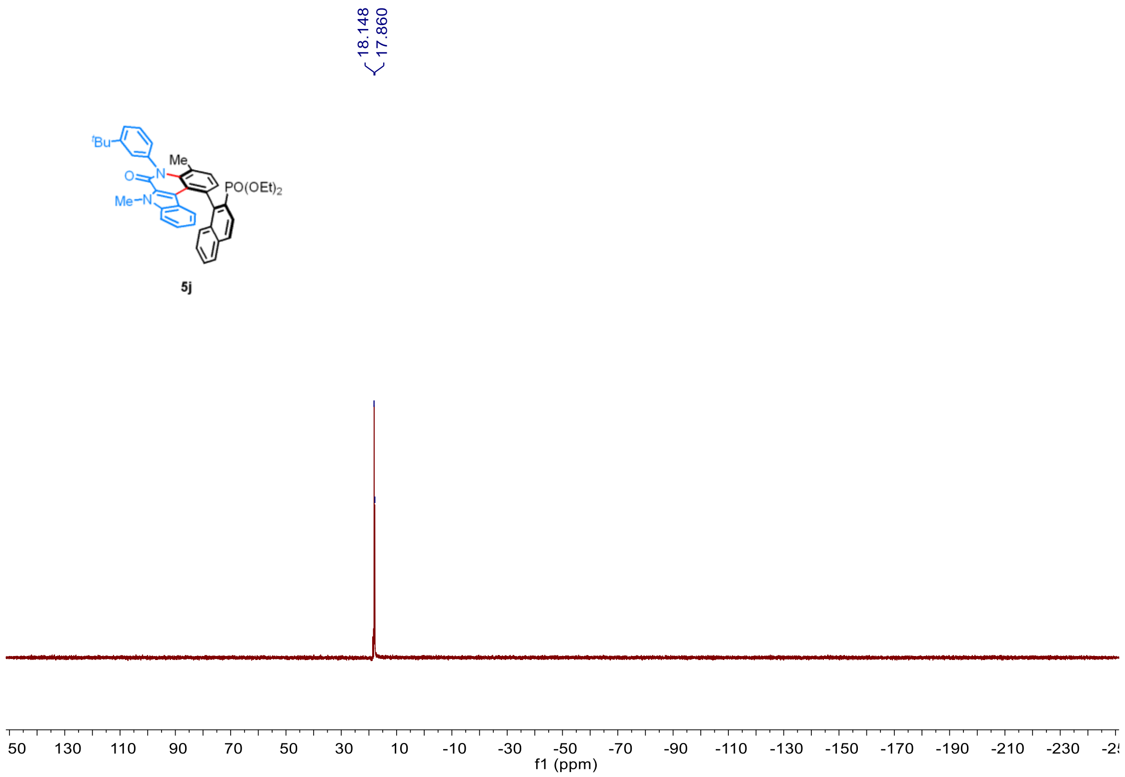

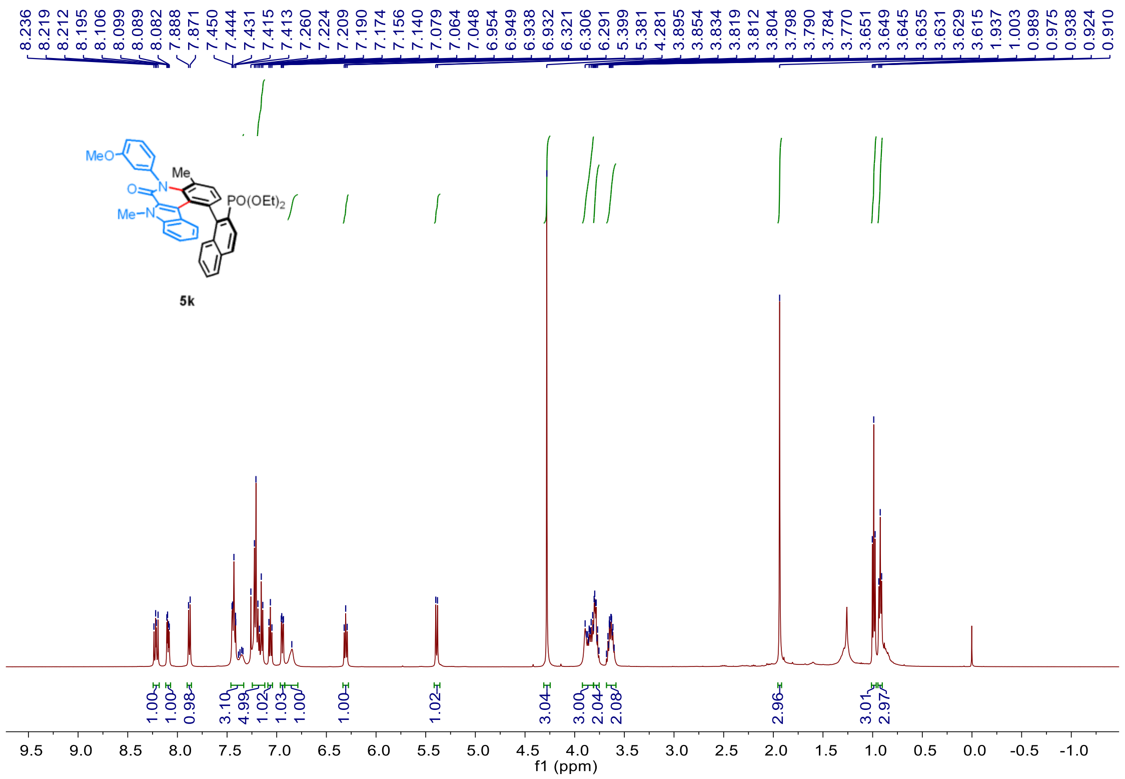

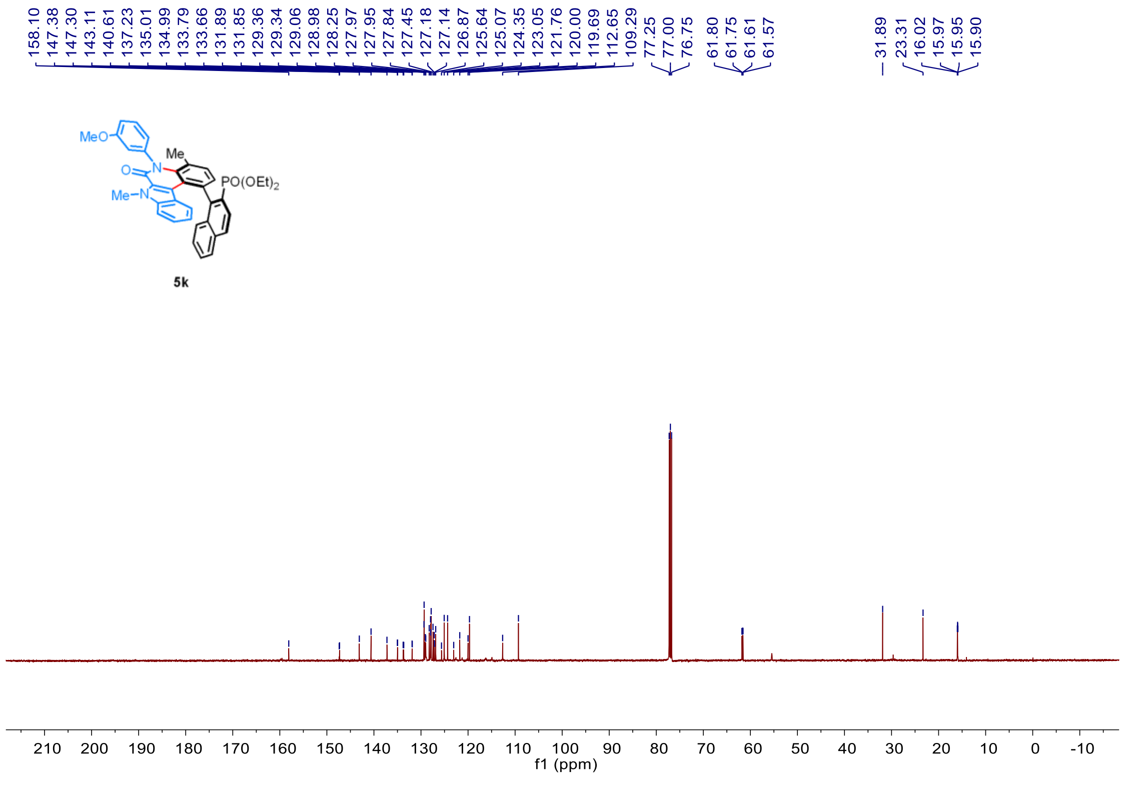

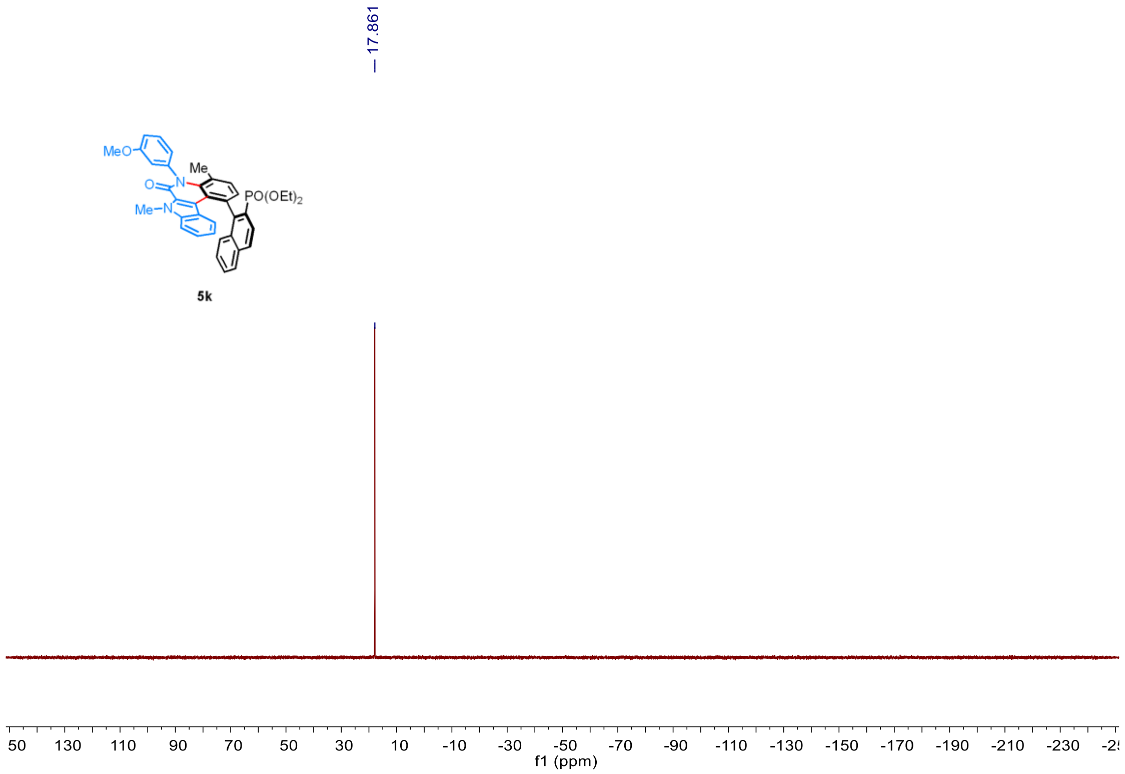

17.861
MeO
O
N
Me
Me−N
PO(OEt)2
5k
50 130 110 90 70 50 30 10 -10 -30 -50 -70 -90 -110 -130 -150 -170 -190 -210 -230
f1 (ppm)

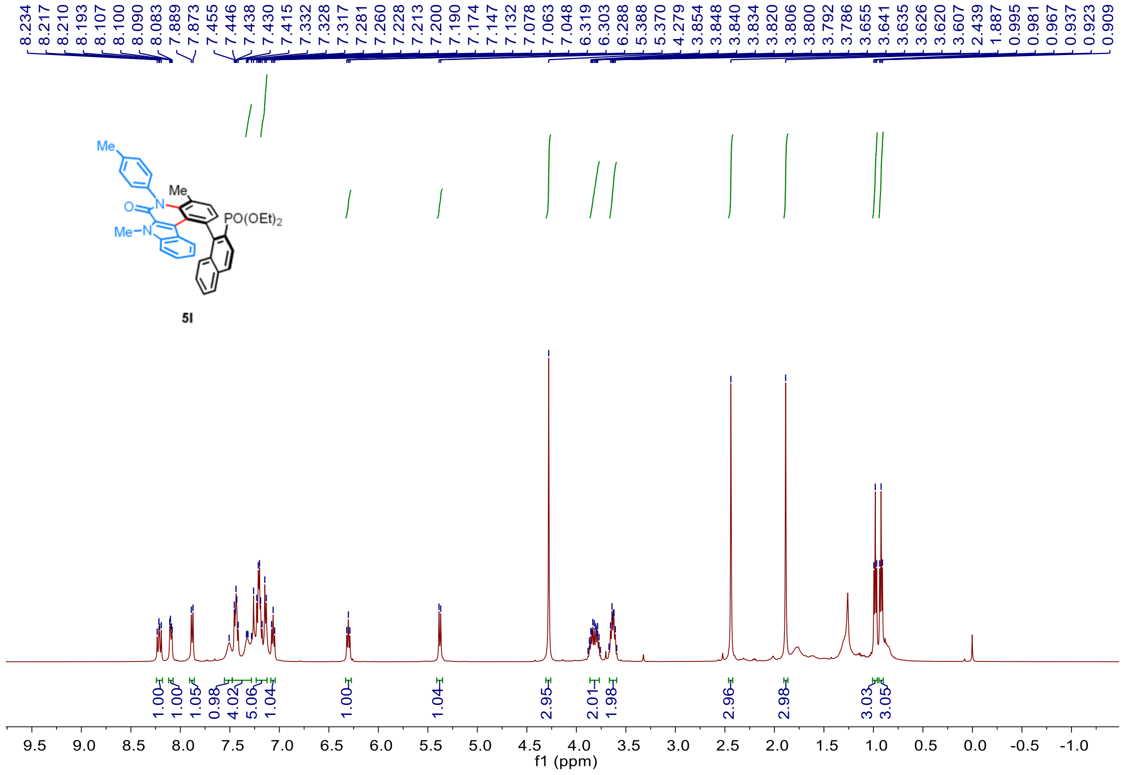

8.234 8.217 8.210 8.193 8.107 8.100 8.090 8.083 7.889 7.873 7.455 7.446 7.438 7.430 7.415 7.332 7.328 7.317 7.281 7.260 7.228 7.213 7.200 7.190 7.174 7.147 7.132 7.078 7.063 7.048 6.319 6.303 6.288 5.388 5.370 4.279 3.854 3.848 3.840 3.834 3.820 3.806 3.800 3.792 3.786 3.655 3.641 3.635 3.626 3.620 3.607 2.439 1.887 0.995 0.981 0.967 0.937 0.923 0.909
Me
O
N
Me
Me−N
PO(OEt)2
5l
1.00 1.00 1.05 0.98 4.02 5.06 1.04 1.00 1.04 2.95 2.01 1.98 2.96 2.98 3.03 3.05
9.5 9.0 8.5 8.0 7.5 7.0 6.5 6.0 5.5 5.0 4.5 4.0 3.5 3.0 2.5 2.0 1.5 1.0 0.5 0.0 -0.5 -1.0
f1 (ppm)

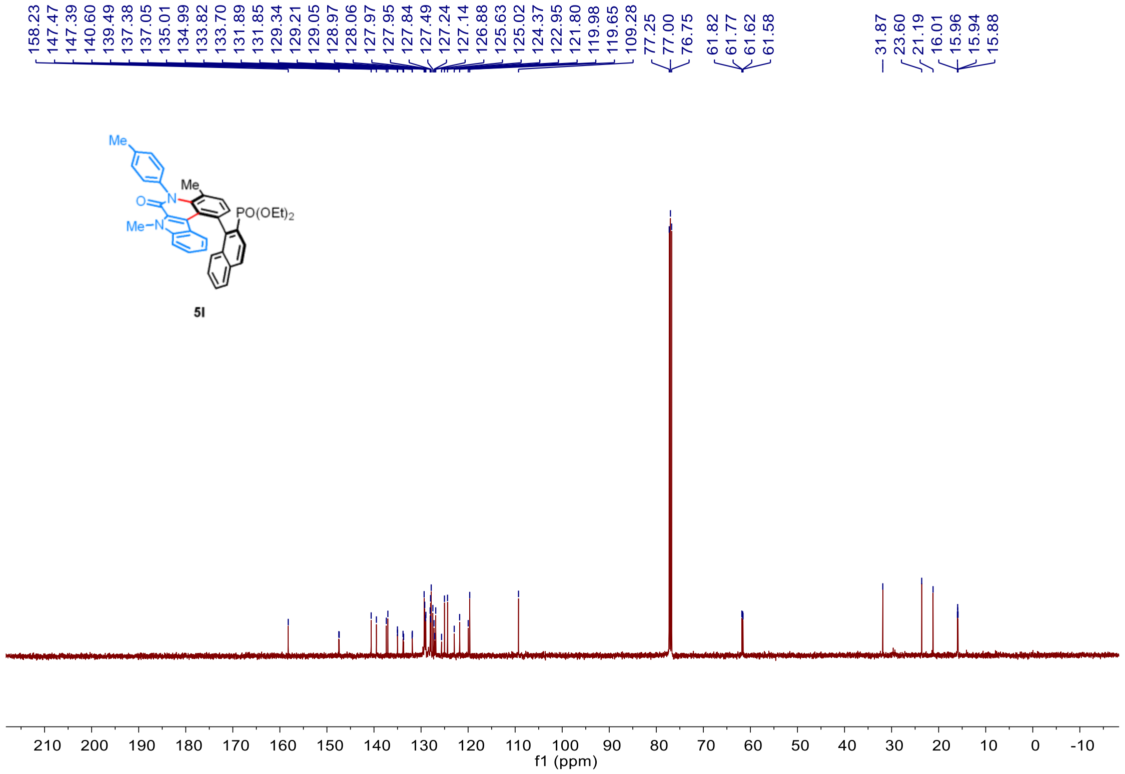
158.23
147.47
147.39
140.60
139.49
137.38
137.05
135.01
134.99
133.82
133.70
131.89
131.85
129.34
129.21
129.05
128.97
128.06
127.97
127.95
127.84
127.49
127.24
127.14
126.88
125.63
125.02
124.37
122.95
121.80
119.98
119.65
109.28
77.25
77.00
76.75
61.82
61.77
61.62
61.58
31.87
23.60
21.19
16.01
15.96
15.94
15.88
Me
Me
O
N
Me
N
PO(OEt)2
5l
210 200 190 180 170 160 150 140 130 120 110 100 90 80 70 60 50 40 30 20 10 0 -10
f1 (ppm)

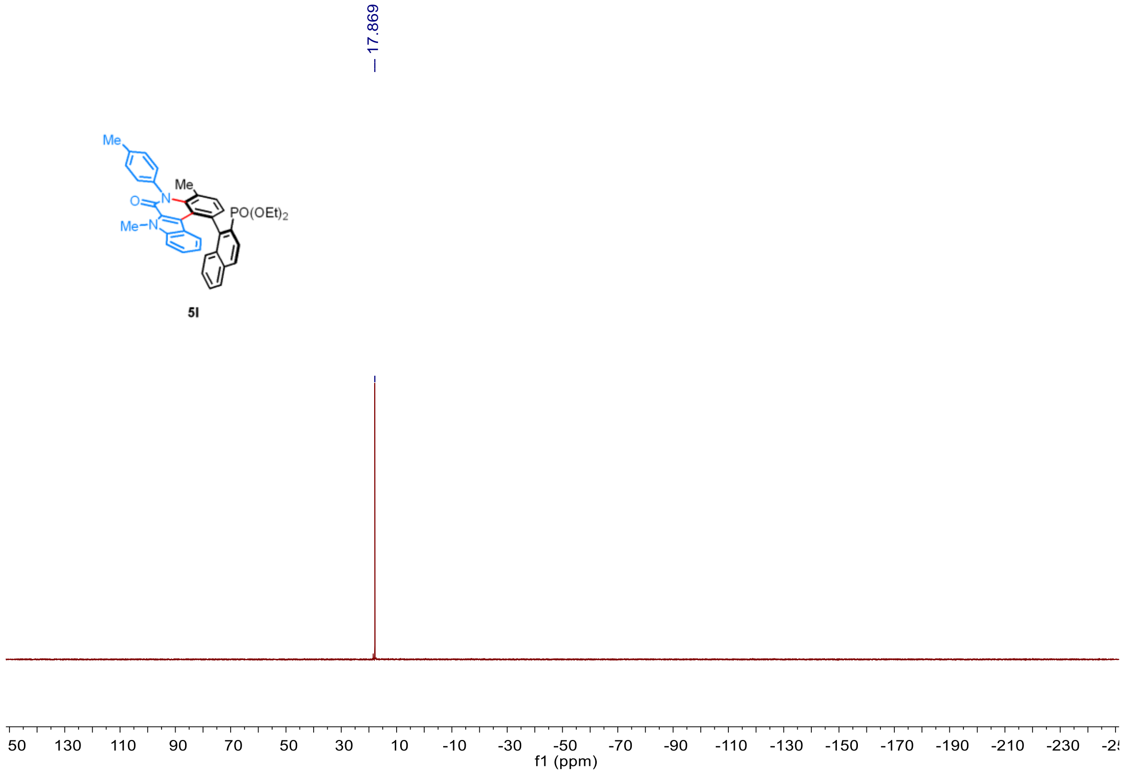
17.869
Me
Me
O
N
Me
N
PO(OEt)2
5l
50 130 110 90 70 50 30 10 -10 -30 -50 -70 -90 -110 -130 -150 -170 -190 -210 -230
f1 (ppm)

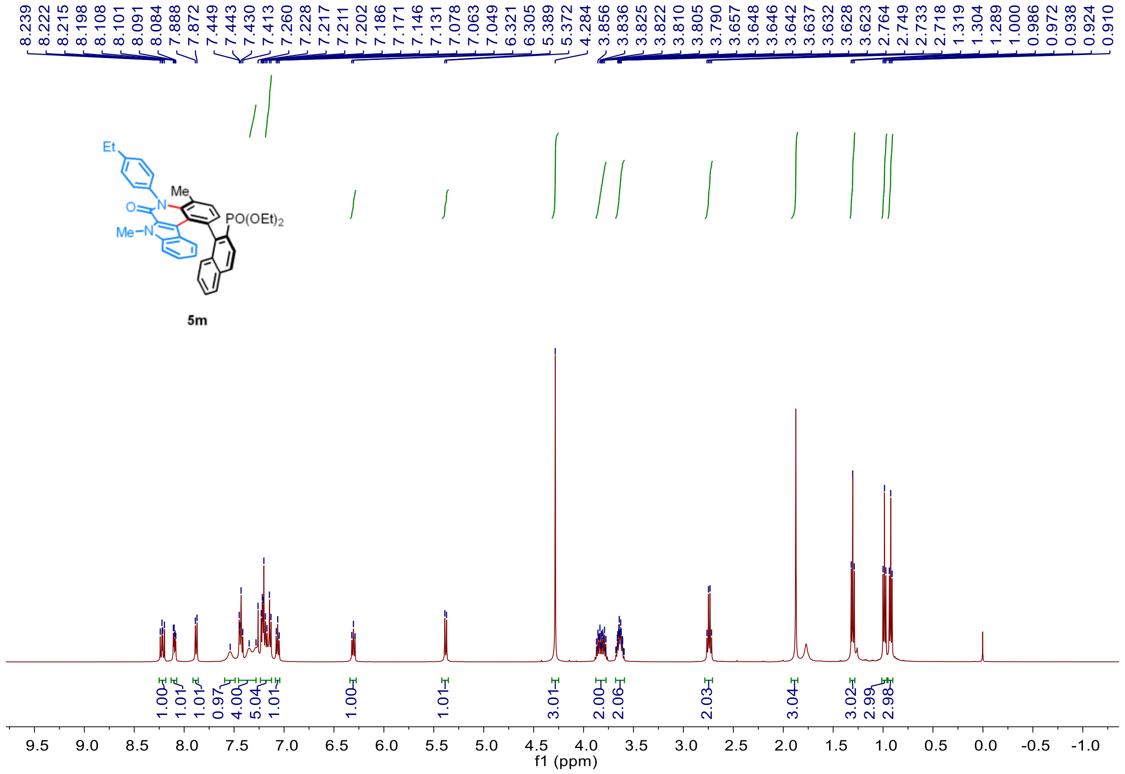
8.239 8.222 8.215 8.198 8.108 8.101 8.091 8.084 7.888 7.872 7.449 7.443 7.430 7.413 7.260 7.228 7.217 7.211 7.202 7.186 7.171 7.146 7.131 7.078 7.063 7.049 6.321 6.305 5.389 5.372 4.284 3.856 3.836 3.825 3.822 3.810 3.805 3.790 3.657 3.648 3.646 3.642 3.637 3.632 3.628 3.623 2.764 2.749 2.733 2.718 1.319 1.304 1.289 1.000 0.986 0.972 0.938 0.924 0.910
Et
O
N
Me
PO(OEt)2
Me
N
5m
1.00 1.01 1.01 0.97 4.00 5.04 1.01 1.00 1.01 3.01 2.00 2.06 2.03 3.04 3.02 2.99 2.98
9.5 9.0 8.5 8.0 7.5 7.0 6.5 6.0 5.5 5.0 4.5 4.0 3.5 3.0 2.5 2.0 1.5 1.0 0.5 0.0 -0.5 -1.0
f1 (ppm)

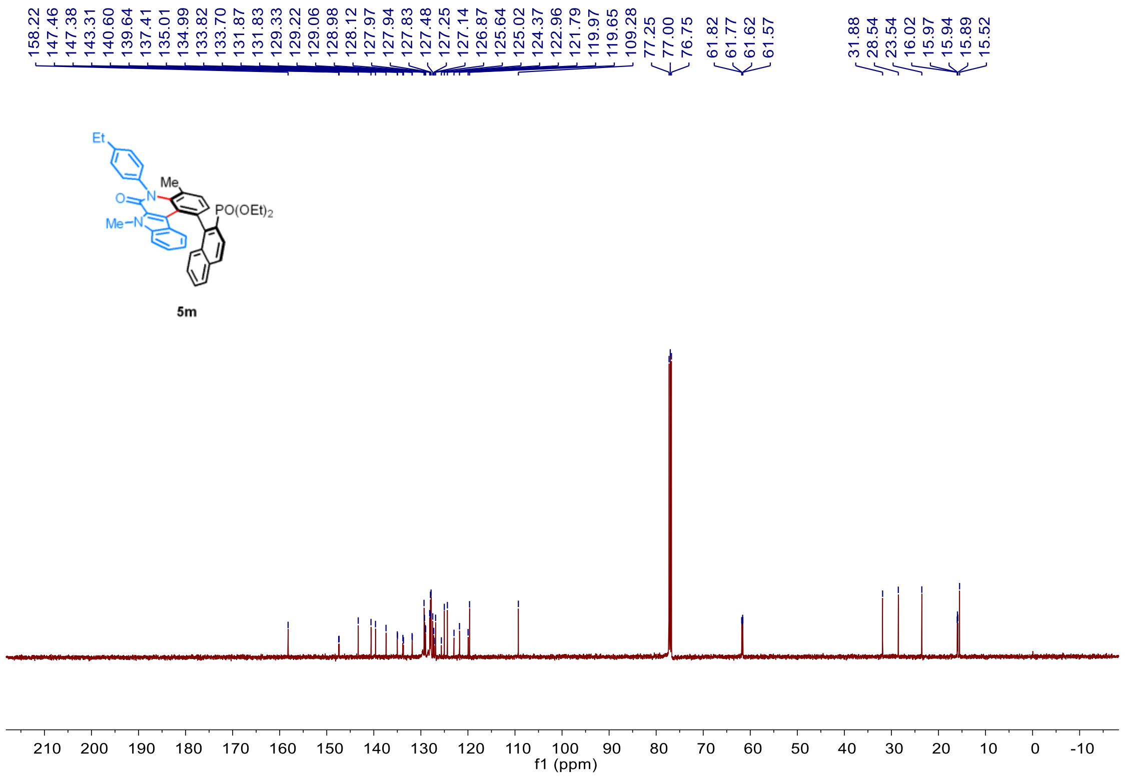
158.22 147.46 147.38 143.31 140.60 139.64 137.41 135.01 134.99 133.82 133.70 131.87 131.83 129.33 129.22 129.06 128.98 128.12 127.97 127.94 127.83 127.48 127.25 127.14 126.87 125.64 125.02 124.37 122.96 121.79 119.97 119.65 109.28 77.25 77.00 76.75 61.82 61.77 61.62 61.57 31.88 28.54 23.54 16.02 15.97 15.94 15.89 15.52
Et
O
N
Me
PO(OEt)2
Me
N
5m
210 200 190 180 170 160 150 140 130 120 110 100 90 80 70 60 50 40 30 20 10 0 -10
f1 (ppm)

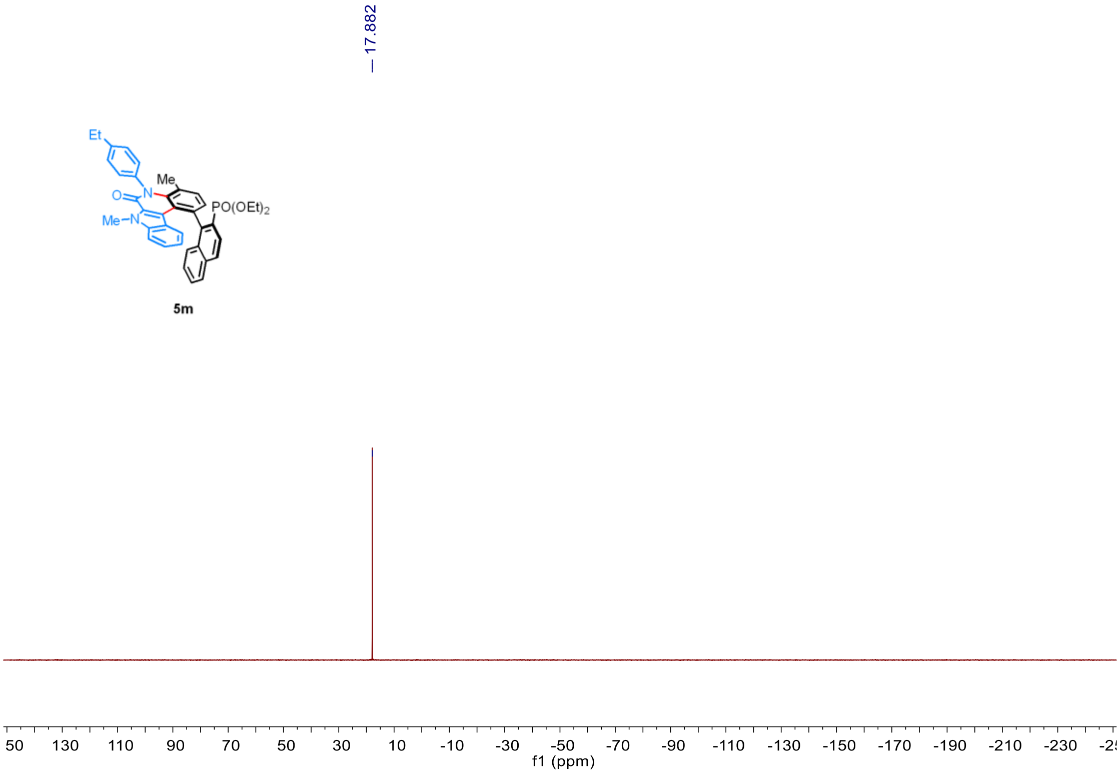

17.882
Et
Me
O
N
Me–N
PO(OEt)2
5m
f1 (ppm)

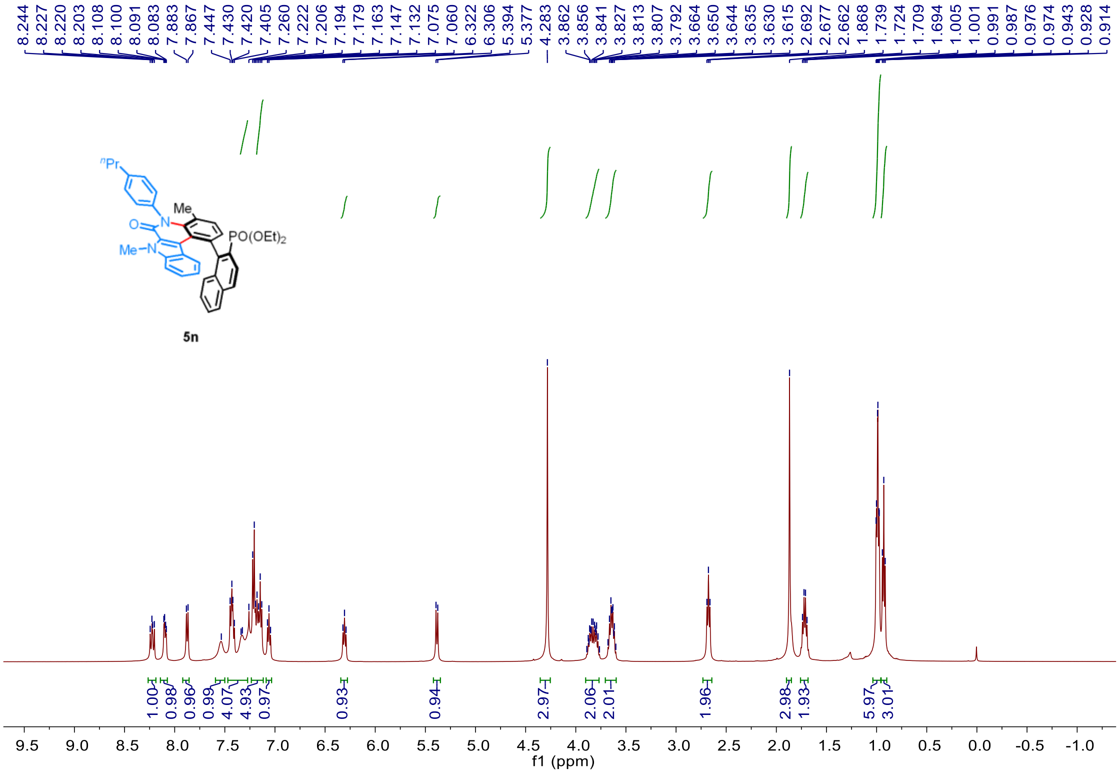

8.244 8.227 8.220 8.203 8.108 8.100 8.091 8.083 7.883 7.867 7.447 7.430 7.420 7.405 7.260 7.222 7.206 7.194 7.179 7.163 7.147 7.132 7.075 7.060 6.322 6.306 5.394 5.377 4.283 3.862 3.856 3.841 3.827 3.813 3.807 3.792 3.664 3.650 3.644 3.635 3.630 3.615 2.692 2.677 2.662 1.868 1.739 1.724 1.709 1.694 1.005 1.001 0.991 0.987 0.976 0.974 0.943 0.928 0.914
nPr
Me
O
N
Me–N
PO(OEt)2
5n
1.00 0.98 0.96 0.99 4.07 4.93 0.97 0.93 0.94 2.97 2.06 2.01 1.96 2.98 1.93 5.97 3.01
f1 (ppm)

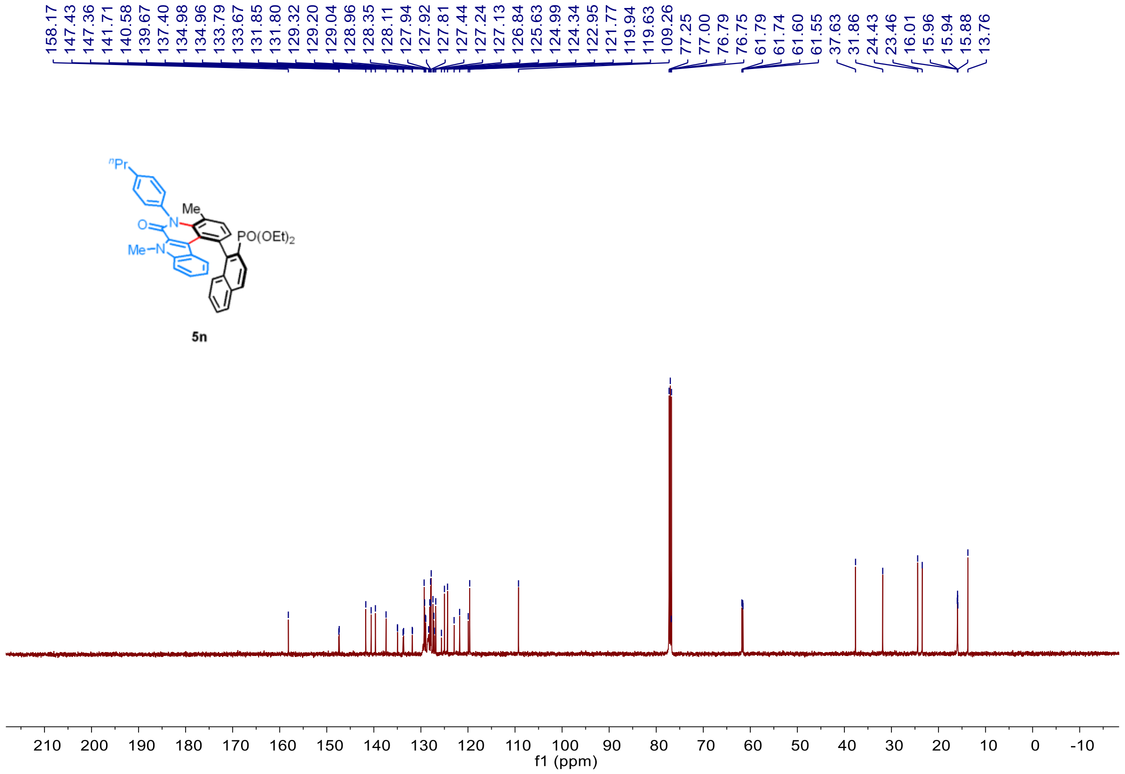
158.17
147.43
147.36
141.71
140.58
139.67
137.40
134.98
134.96
133.79
133.67
131.85
131.80
129.32
129.20
129.04
128.96
128.35
128.11
127.94
127.92
127.81
127.44
127.24
127.13
126.84
125.63
124.99
124.34
122.95
121.77
119.94
119.63
109.26
77.25
77.00
76.79
76.75
61.79
61.74
61.60
61.55
37.63
31.86
24.43
23.46
16.01
15.96
15.94
15.88
13.76
nPr
Me
O
N
PO(OEt)2
Me–N
5n
210 200 190 180 170 160 150 140 130 120 110 100 90 80 70 60 50 40 30 20 10 0 -10
f1 (ppm)

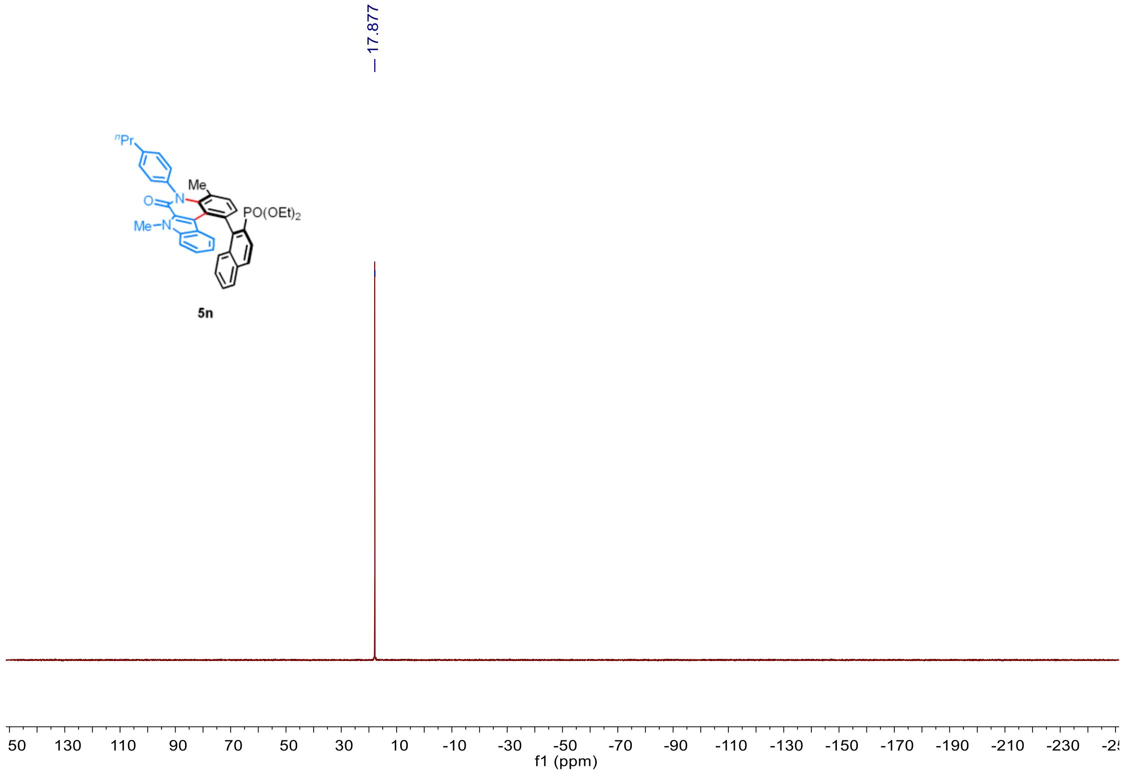
17.877
nPr
Me
O
N
PO(OEt)2
Me–N
5n
50 130 110 90 70 50 30 10 -10 -30 -50 -70 -90 -110 -130 -150 -170 -190 -210 -230
f1 (ppm)

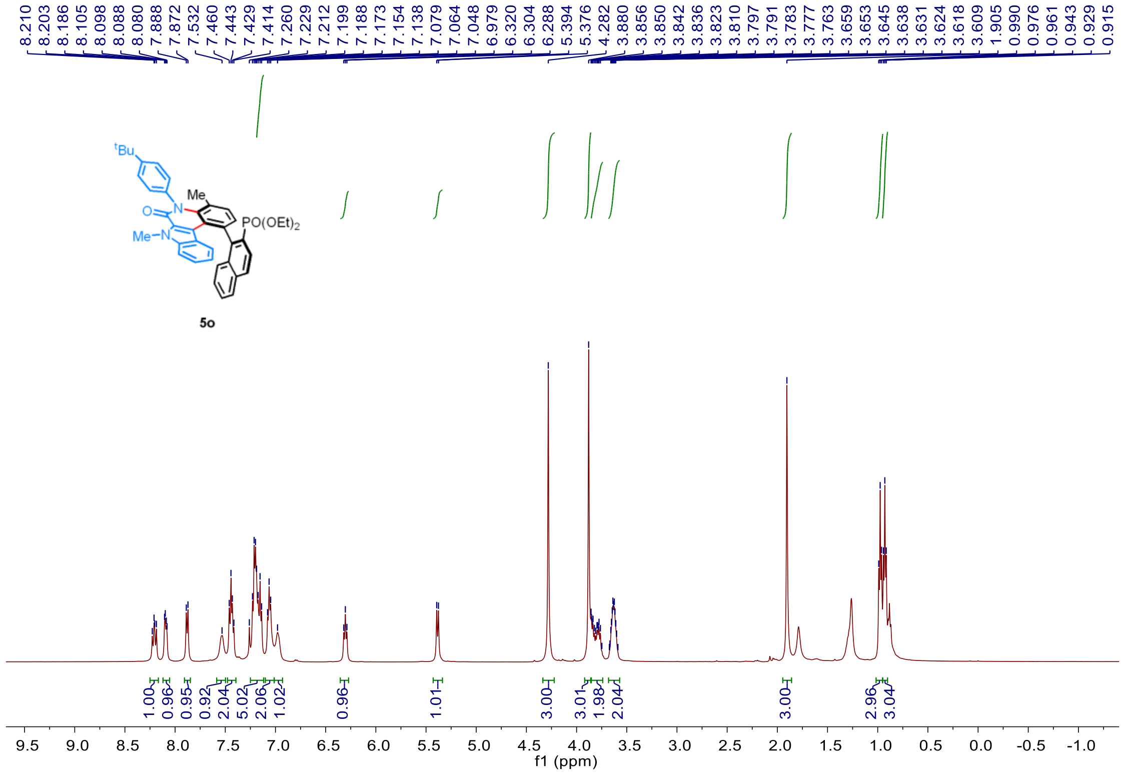
8.210 8.203 8.186 8.105 8.098 8.088 8.080 7.888 7.872 7.532 7.460 7.443 7.429 7.414 7.260 7.229 7.212 7.199 7.188 7.173 7.154 7.138 7.079 7.064 7.048 6.979 6.320 6.304 6.288 5.394 5.376 4.282 3.880 3.856 3.850 3.842 3.836 3.823 3.810 3.797 3.791 3.783 3.777 3.763 3.659 3.653 3.645 3.638 3.631 3.624 3.618 3.609 1.905 0.990 0.976 0.961 0.943 0.929 0.915
tBu
Me
O
N
Me
N
PO(OEt)2
5o
1.00 0.96 0.95 0.92 2.04 5.02 2.06 1.02 0.96 1.01 3.00 3.01 1.98 2.04 3.00 2.96 3.04
9.5 9.0 8.5 8.0 7.5 7.0 6.5 6.0 5.5 5.0 4.5 4.0 3.5 3.0 2.5 2.0 1.5 1.0 0.5 0.0 -0.5 -1.0
f1 (ppm)

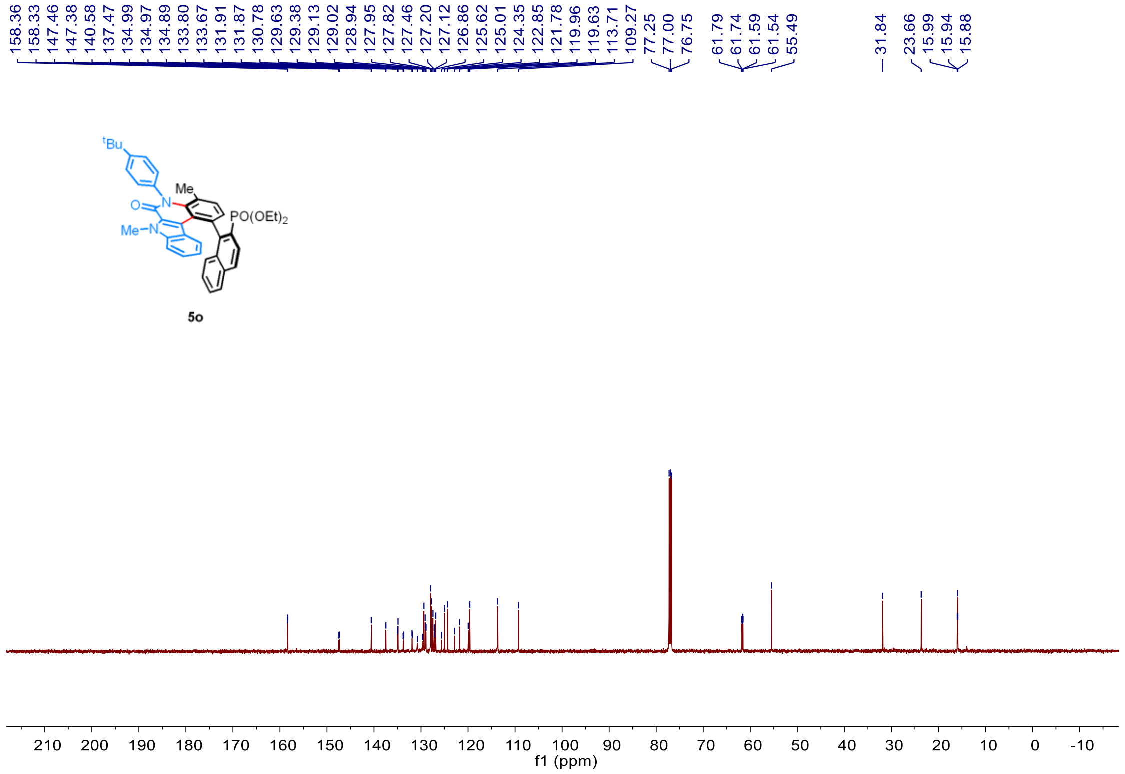
158.36 158.33 147.46 147.38 140.58 137.47 134.99 134.97 134.89 133.80 133.67 131.91 131.87 130.78 129.63 129.38 129.13 129.02 128.94 127.95 127.82 127.46 127.20 127.12 126.86 125.62 125.01 124.35 122.85 121.78 119.96 119.63 113.71 109.27 77.25 77.00 76.75 61.79 61.74 61.59 61.54 55.49 31.84 23.66 15.99 15.94 15.88
tBu
Me
O
N
Me
N
PO(OEt)2
5o
210 200 190 180 170 160 150 140 130 120 110 100 90 80 70 60 50 40 30 20 10 0 -10
f1 (ppm)

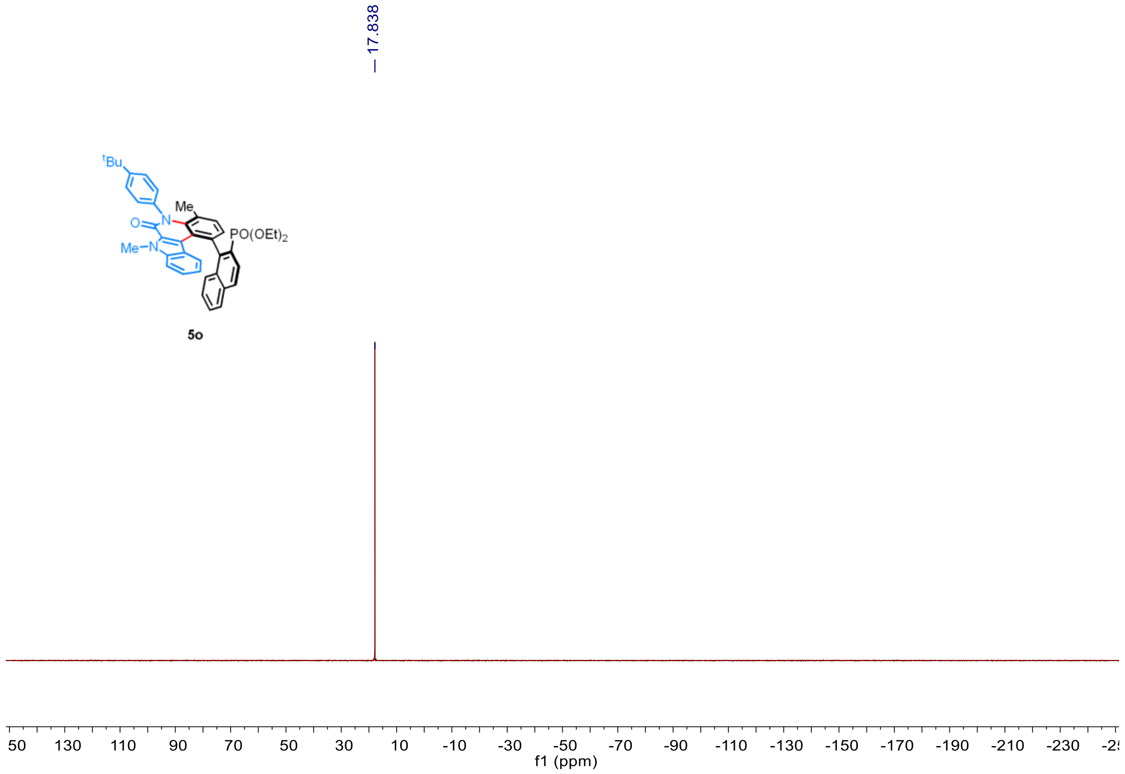

17.838
ᵗBu
Me
PO(OEt)₂
O
N
Me–N
50 130 110 90 70 50 30 10 -10 -30 -50 -70 -90 -110 -130 -150 -170 -190 -210 -230
f1 (ppm)

5o

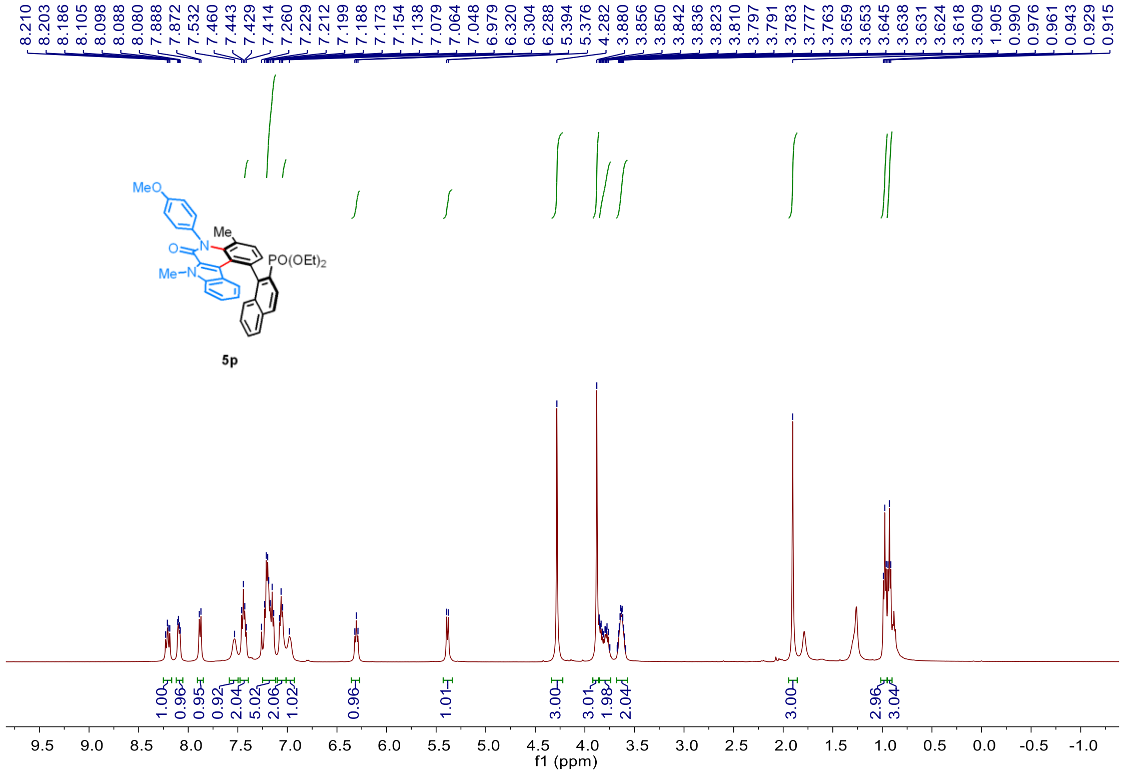

8.210 8.203 8.186 8.105 8.098 8.088 8.080 7.888 7.872 7.532 7.460 7.443 7.429 7.414 7.260 7.229 7.212 7.199 7.188 7.173 7.154 7.138 7.079 7.064 7.048 6.979 6.320 6.304 6.288 5.394 5.376 4.282 3.880 3.856 3.850 3.842 3.836 3.823 3.810 3.797 3.791 3.783 3.777 3.763 3.659 3.653 3.645 3.638 3.631 3.624 3.618 3.609 1.905 0.990 0.976 0.961 0.943 0.929 0.915
MeO
Me
PO(OEt)₂
O
N
Me–N
1.00 0.96 0.95 0.92 2.04 5.02 2.06 1.02 0.96 1.01 3.00 3.01 1.98 2.04 3.00 2.96 3.04
9.5 9.0 8.5 8.0 7.5 7.0 6.5 6.0 5.5 5.0 4.5 4.0 3.5 3.0 2.5 2.0 1.5 1.0 0.5 0.0 -0.5 -1.0
f1 (ppm)

5p

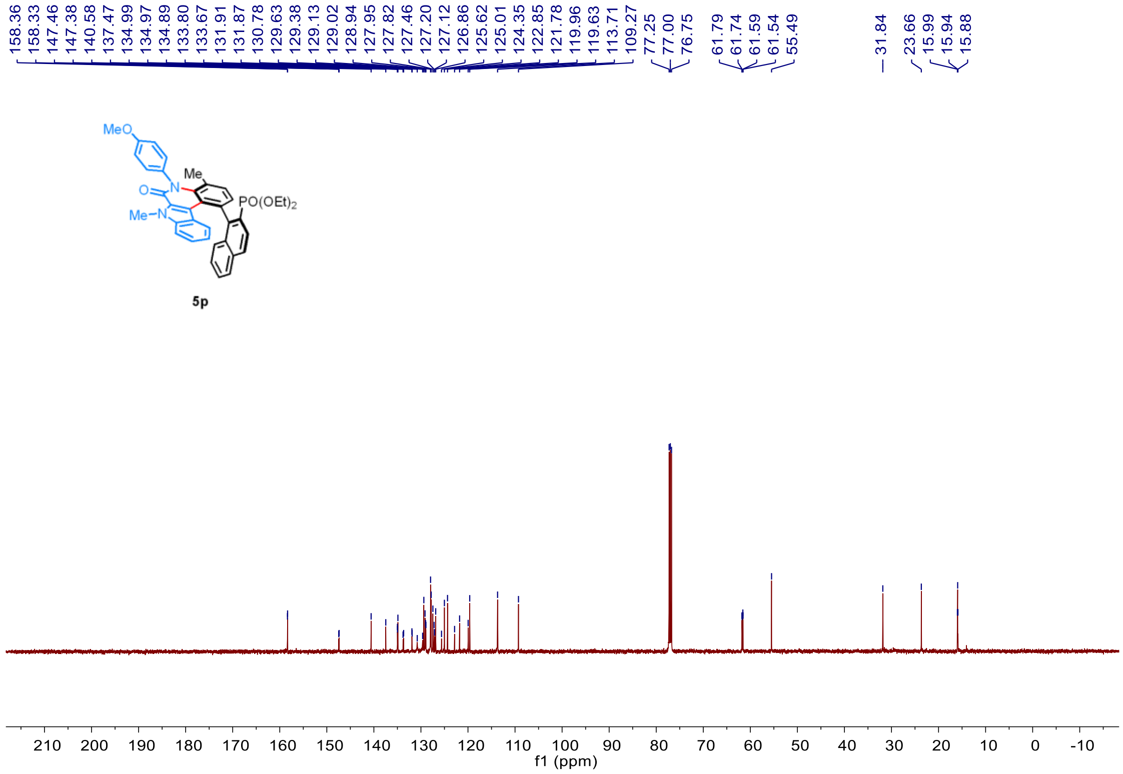
158.36
158.33
147.46
147.38
140.58
137.47
134.99
134.97
134.89
133.80
133.67
131.91
131.87
130.78
129.63
129.38
129.13
129.02
128.94
127.95
127.82
127.46
127.20
127.12
126.86
125.62
125.01
124.35
122.85
121.78
119.96
119.63
113.71
109.27
77.25
77.00
76.75
61.79
61.74
61.59
61.54
55.49
31.84
23.66
15.99
15.94
15.88
MeO
Me
N
O
PO(OEt)2
Me–N
5p
210 200 190 180 170 160 150 140 130 120 110 100 90 80 70 60 50 40 30 20 10 0 -10
f1 (ppm)

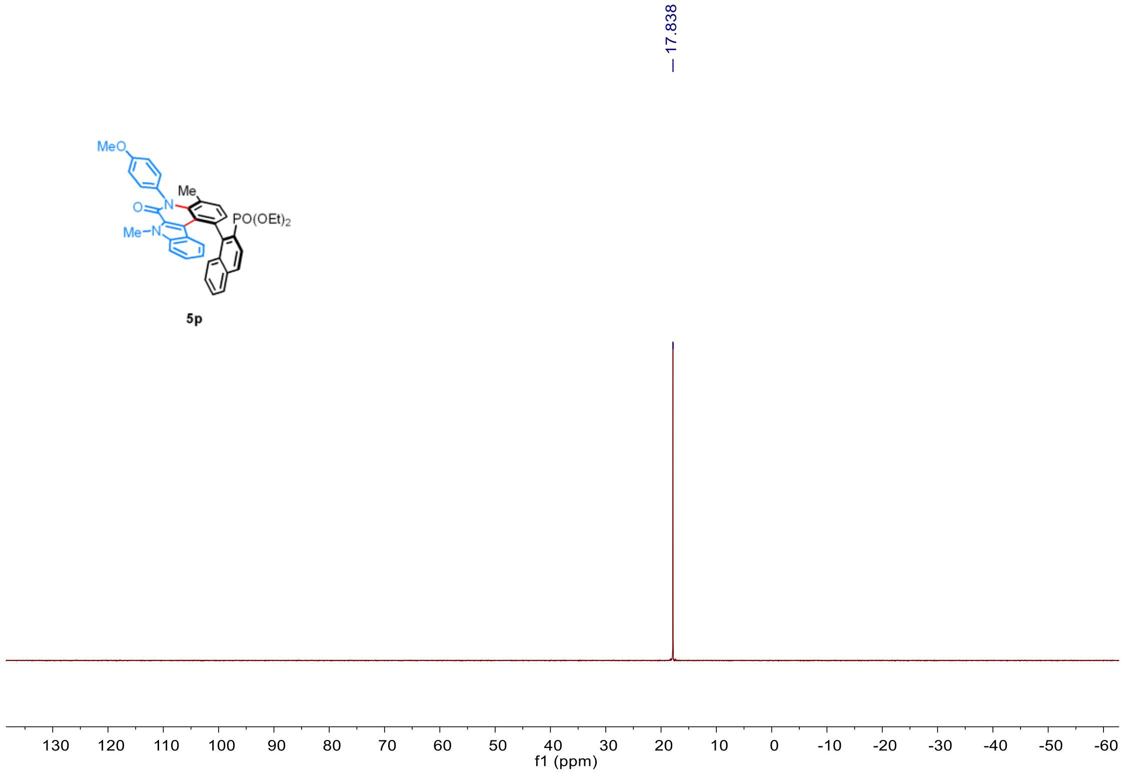
17.838
MeO
Me
N
O
PO(OEt)2
Me–N
5p
130 120 110 100 90 80 70 60 50 40 30 20 10 0 -10 -20 -30 -40 -50 -60
f1 (ppm)

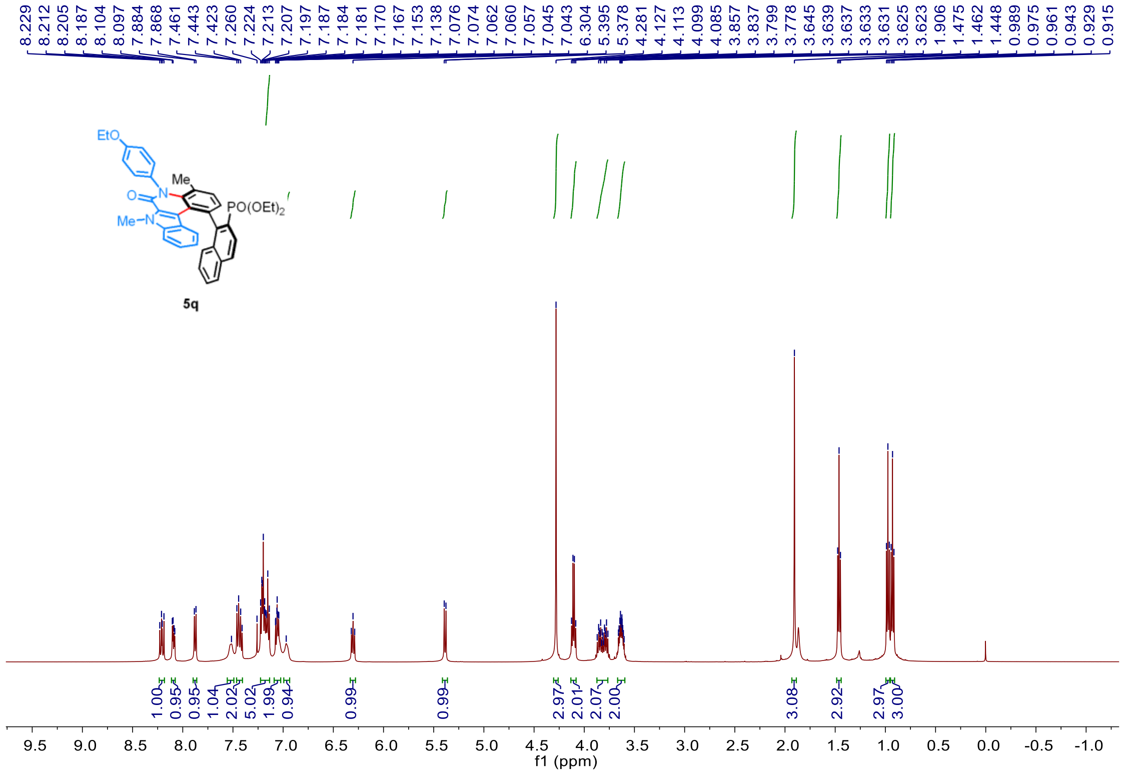
8.229 8.212 8.205 8.187 8.104 8.097 7.884 7.868 7.461 7.443 7.423 7.260 7.224 7.213 7.207 7.197 7.187 7.184 7.181 7.170 7.167 7.153 7.138 7.076 7.074 7.062 7.060 7.057 7.045 7.043 6.304 5.395 5.378 4.281 4.127 4.113 4.099 4.085 3.857 3.837 3.799 3.778 3.645 3.639 3.637 3.633 3.631 3.625 3.623 1.906 1.475 1.462 1.448 0.989 0.975 0.961 0.943 0.929 0.915
EtO
Me
N
O
Me–N
PO(OEt)2
5q
1.00 0.95 0.95 1.04 2.02 5.02 1.99 0.94 0.99 0.99 2.97 2.01 2.07 2.00 3.08 2.92 2.97 3.00
9.5 9.0 8.5 8.0 7.5 7.0 6.5 6.0 5.5 5.0 4.5 4.0 3.5 3.0 2.5 2.0 1.5 1.0 0.5 0.0 -0.5 -1.0
f1 (ppm)

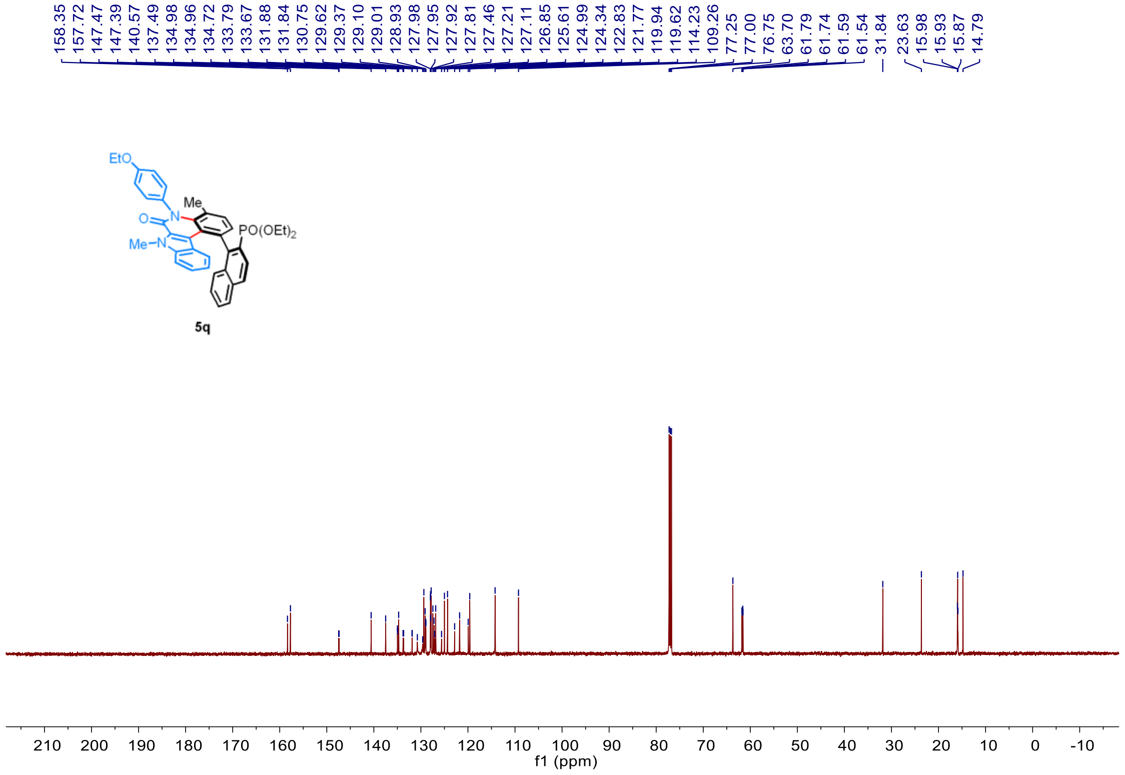
158.35 157.72 147.47 147.39 140.57 137.49 134.98 134.96 134.72 133.79 133.67 131.88 131.84 130.75 129.62 129.37 129.10 129.01 128.93 127.98 127.95 127.92 127.81 127.46 127.21 127.11 126.85 125.61 124.99 124.34 122.83 121.77 119.94 119.62 114.23 109.26 77.25 77.00 76.75 63.70 61.79 61.74 61.59 61.54 31.84 23.63 15.98 15.93 15.87 14.79
EtO
Me
N
O
Me–N
PO(OEt)2
5q
210 200 190 180 170 160 150 140 130 120 110 100 90 80 70 60 50 40 30 20 10 0 -10
f1 (ppm)

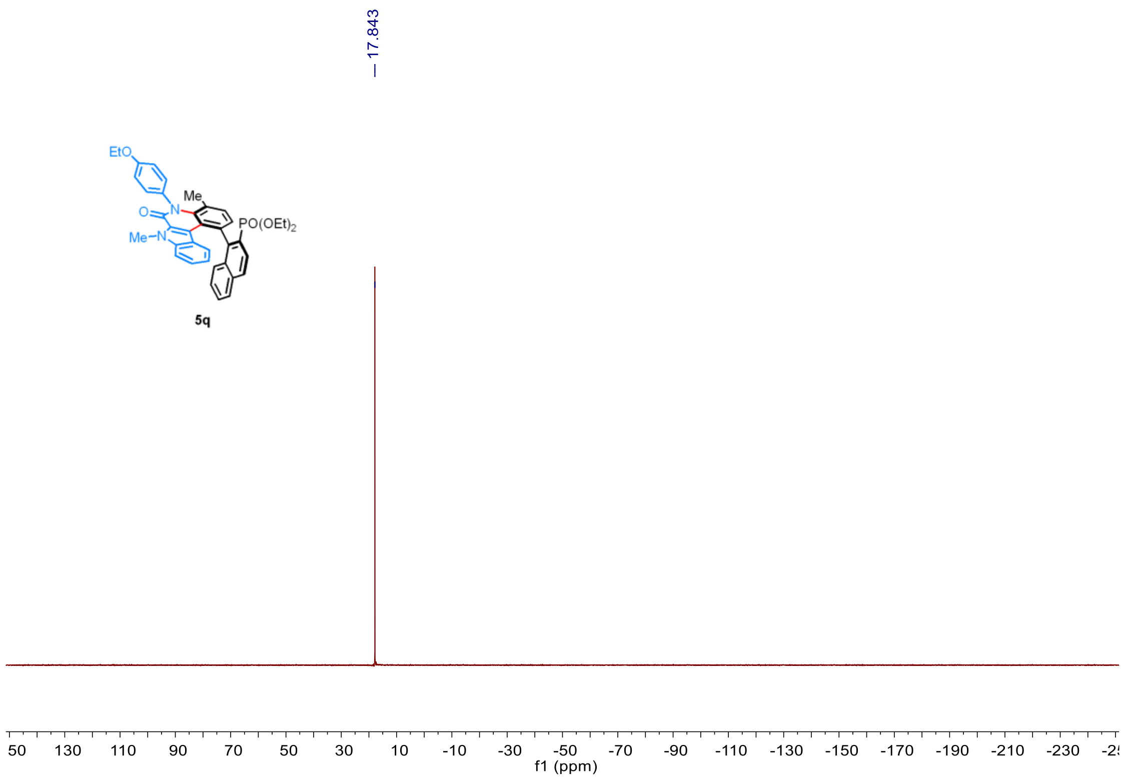

— 17.843
EtO
Me
O
N
Me–N
PO(OEt)2
50 130 110 90 70 50 30 10 -10 -30 -50 -70 -90 -110 -130 -150 -170 -190 -210 -230
f1 (ppm)

5q

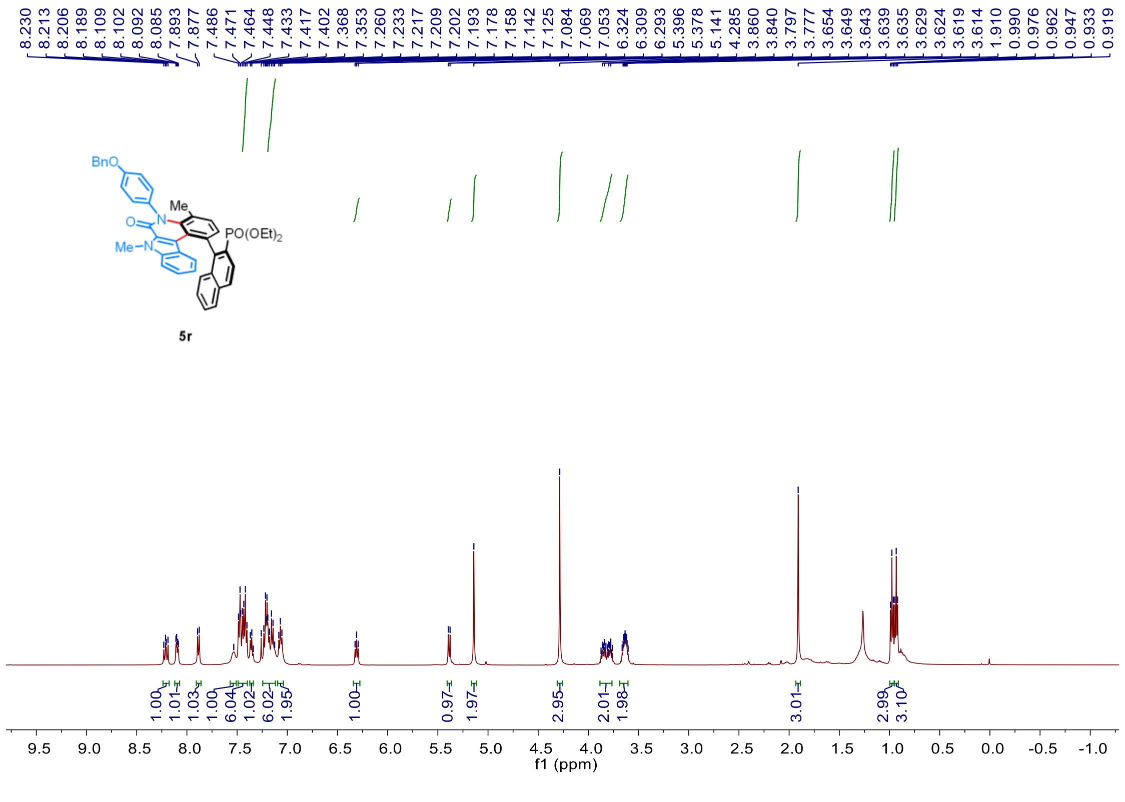

8.230 8.213 8.206 8.189 8.109 8.102 8.092 8.085 7.893 7.877 7.486 7.471 7.464 7.448 7.433 7.417 7.402 7.368 7.353 7.260 7.233 7.217 7.209 7.202 7.193 7.178 7.158 7.142 7.125 7.084 7.069 7.053 6.324 6.309 6.293 5.396 5.378 5.141 4.285 3.860 3.840 3.797 3.777 3.654 3.649 3.643 3.639 3.635 3.629 3.624 3.619 3.614 1.910 0.990 0.976 0.962 0.947 0.933 0.919
BnO
Me
O
N
Me–N
PO(OEt)2
1.00 1.01 1.03 1.00 6.04 1.02 6.02 1.95 1.00 0.97 1.97 2.95 2.01 1.98 3.01 2.99 3.10
9.5 9.0 8.5 8.0 7.5 7.0 6.5 6.0 5.5 5.0 4.5 4.0 3.5 3.0 2.5 2.0 1.5 1.0 0.5 0.0 -0.5 -1.0
f1 (ppm)

5r

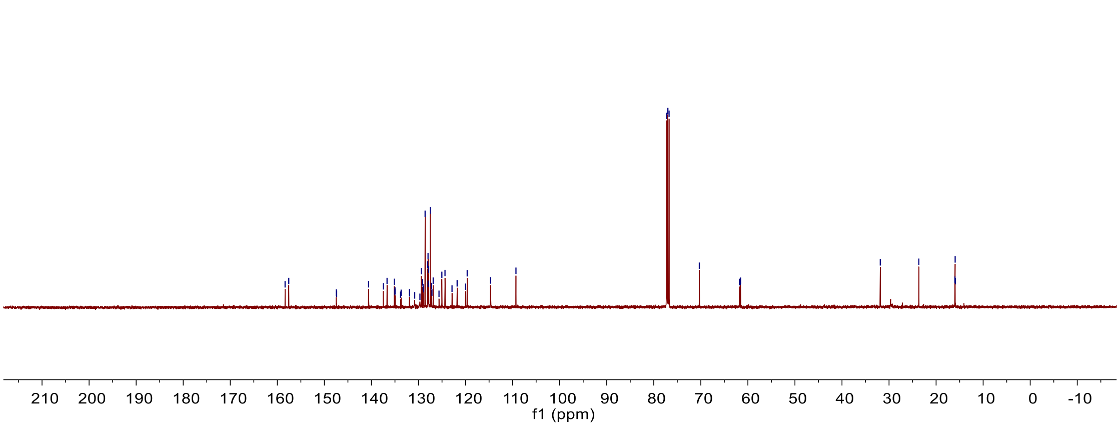

158.35
157.57
147.47
147.39
140.60
137.47
136.68
135.14
135.01
134.99
133.81
133.69
131.93
131.88
130.80
129.70
129.40
129.16
129.03
128.95
128.59
128.04
127.97
127.95
127.84
127.49
127.20
127.14
126.88
125.64
125.03
124.37
122.87
121.79
119.99
119.65
114.69
109.28
77.26
77.00
76.75
70.32
61.80
61.76
61.61
61.56
31.86
23.67
16.00
15.95
15.90
210 200 190 180 170 160 150 140 130 120 110 100 90 80 70 60 50 40 30 20 10 0 -10
f1 (ppm)

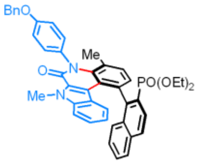

BnO
Me
O
N
Me−N
PO(OEt)$_2$

5r

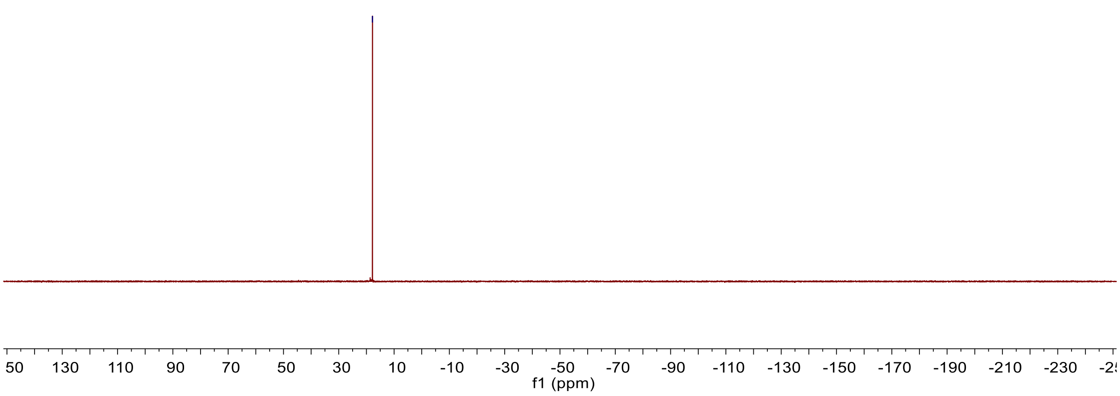

17.813
50 130 110 90 70 50 30 10 -10 -30 -50 -70 -90 -110 -130 -150 -170 -190 -210 -230 -2!
f1 (ppm)

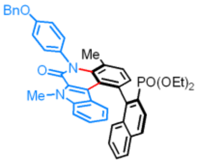

BnO
Me
O
N
Me−N
PO(OEt)$_2$

5r

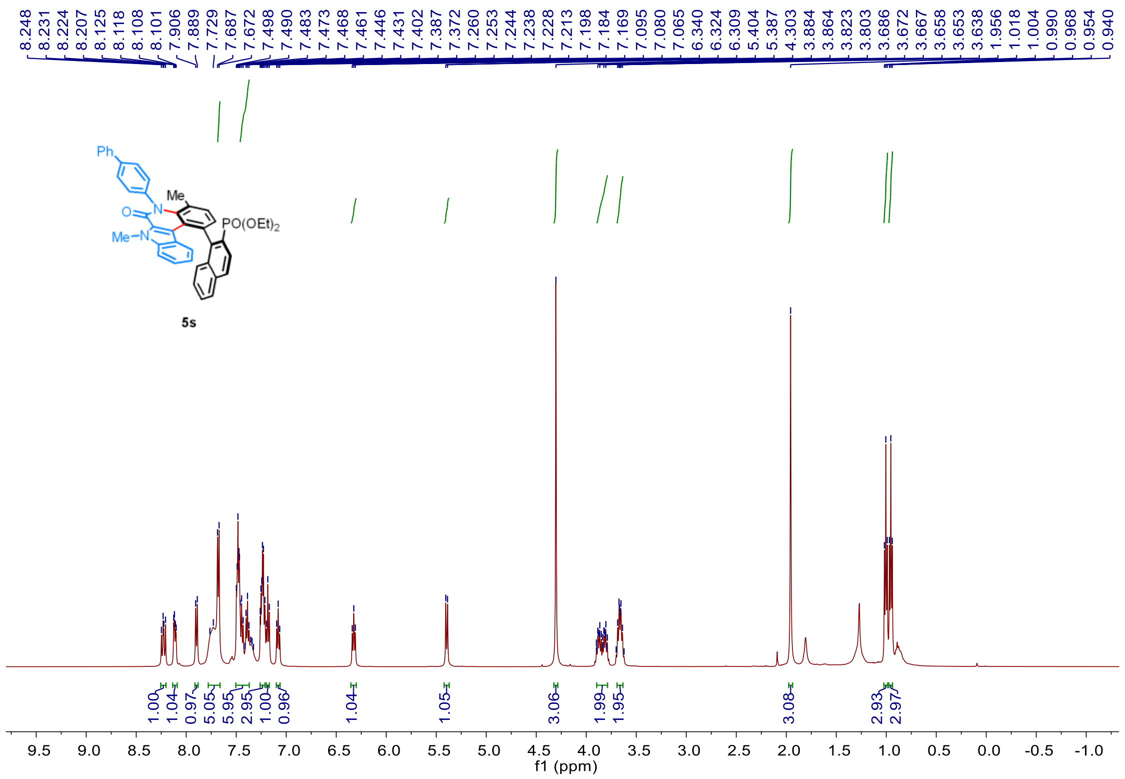
8.248
8.231
8.224
8.207
8.125
8.118
8.108
8.101
7.906
7.889
7.729
7.687
7.672
7.498
7.490
7.483
7.473
7.468
7.461
7.446
7.431
7.402
7.387
7.372
7.260
7.253
7.244
7.238
7.228
7.213
7.198
7.184
7.169
7.095
7.080
7.065
6.340
6.324
6.309
5.404
5.387
4.303
3.884
3.864
3.823
3.803
3.686
3.672
3.667
3.658
3.653
3.638
1.956
1.018
1.004
0.990
0.968
0.954
0.940
Ph
Me
O
N
PO(OEt)2
Me–N
5s
1.00
1.04
0.97
5.05
5.95
2.95
1.00
0.96
1.04
1.05
3.06
1.99
1.95
3.08
2.93
2.97
f1 (ppm)

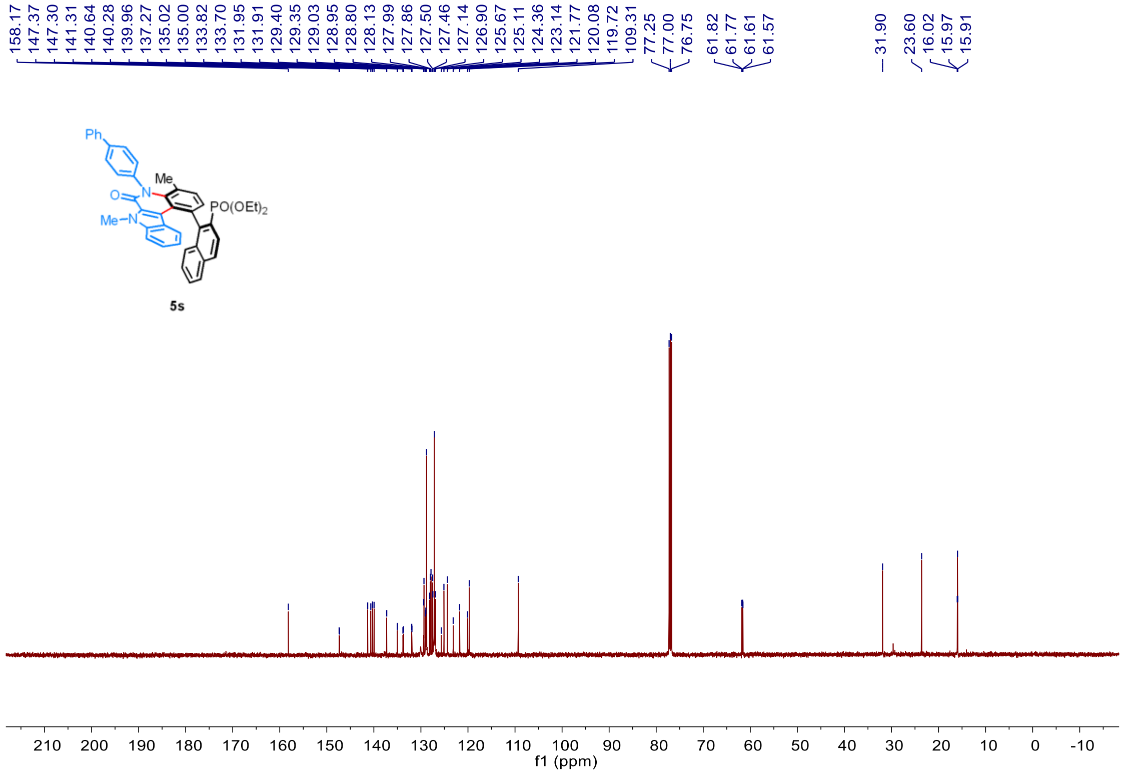
158.17
147.37
147.30
141.31
140.64
140.28
139.96
137.27
135.02
135.00
133.82
133.70
131.95
131.91
129.40
129.35
129.03
128.95
128.80
128.13
127.99
127.86
127.50
127.46
127.14
126.90
125.67
125.11
124.36
123.14
121.77
120.08
119.72
109.31
77.25
77.00
76.75
61.82
61.77
61.61
61.57
31.90
23.60
16.02
15.97
15.91
Ph
Me
O
N
PO(OEt)2
Me–N
5s
f1 (ppm)

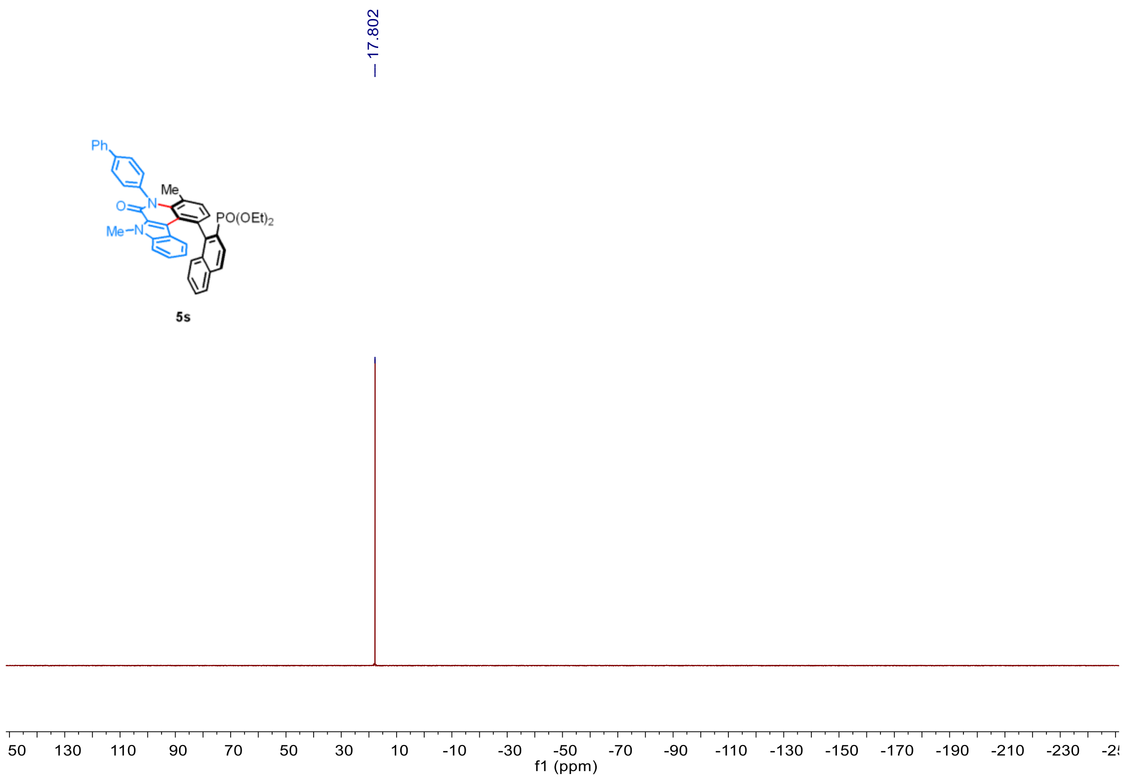

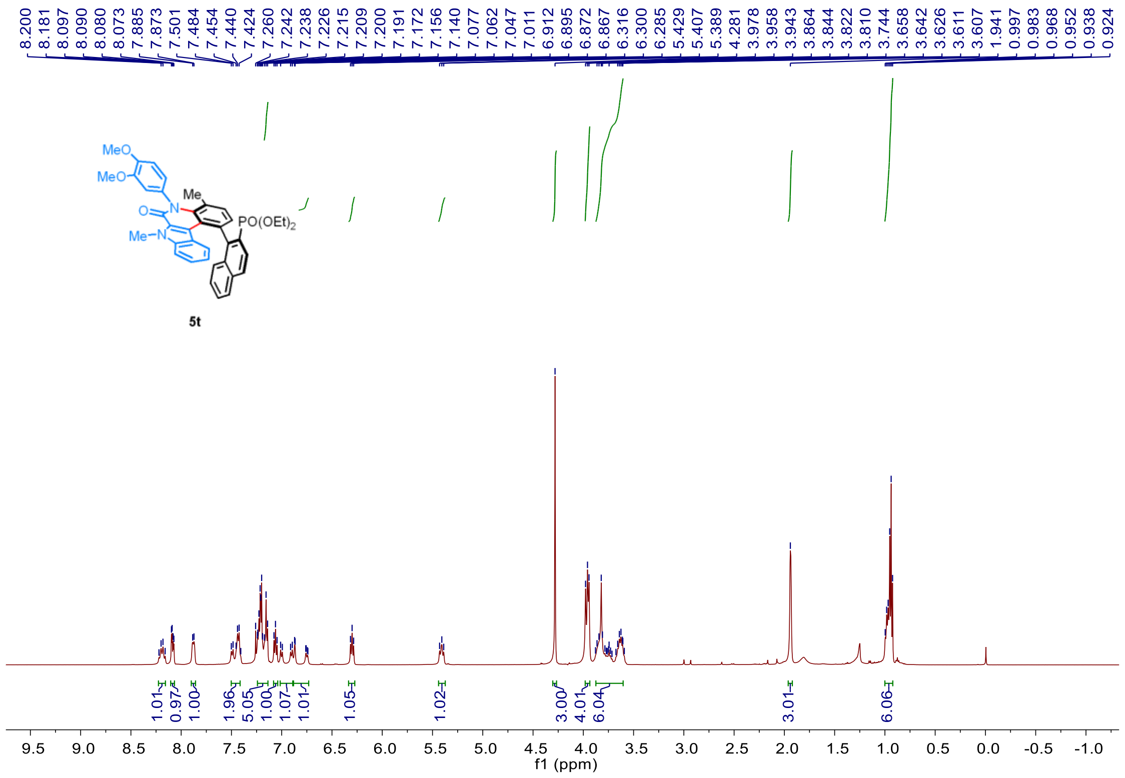

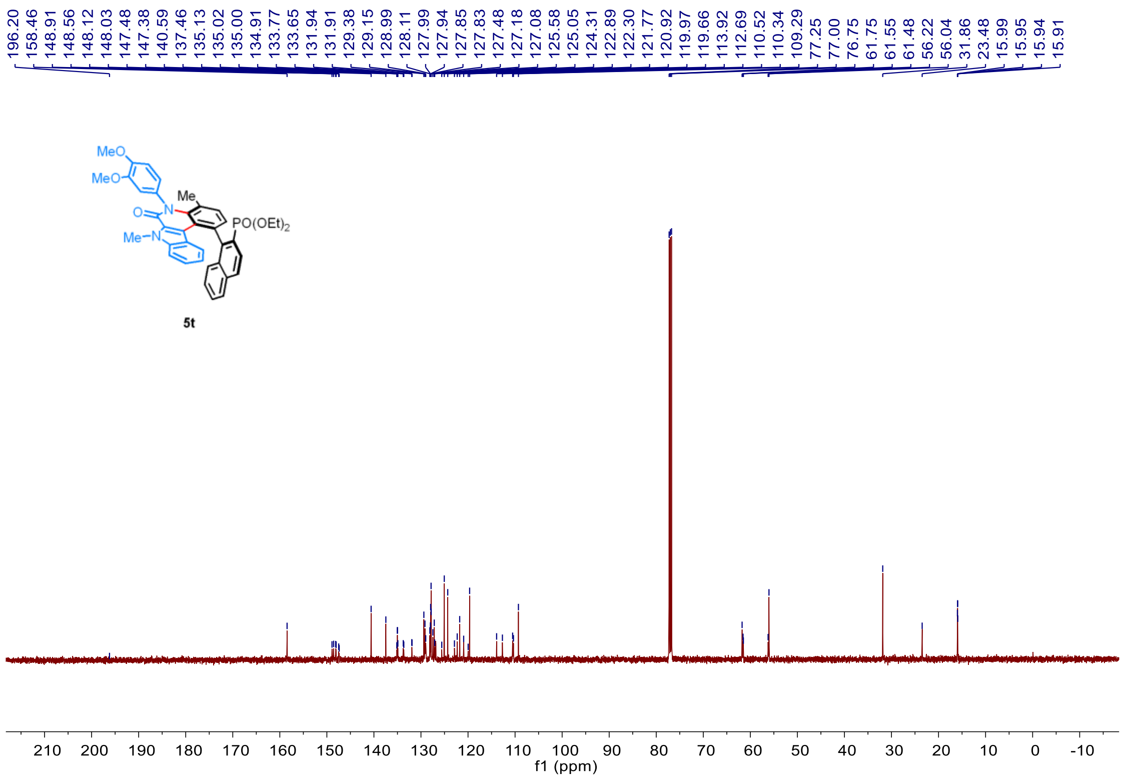

196.20
158.46
148.91
148.56
148.12
148.03
147.48
147.38
140.59
137.46
135.13
135.02
135.00
134.91
133.77
133.65
131.94
131.91
129.38
129.15
128.99
128.11
127.99
127.94
127.85
127.83
127.48
127.18
127.08
125.58
125.05
124.31
122.89
122.30
121.77
120.92
119.97
119.66
113.92
112.69
110.52
110.34
109.29
77.25
77.00
76.75
61.75
61.55
61.48
56.22
56.04
31.86
23.48
15.99
15.95
15.94
15.91
MeO
MeO
Me
O
N
Me–N
PO(OEt)$_2$
5t
210 200 190 180 170 160 150 140 130 120 110 100 90 80 70 60 50 40 30 20 10 0 -10
f1 (ppm)

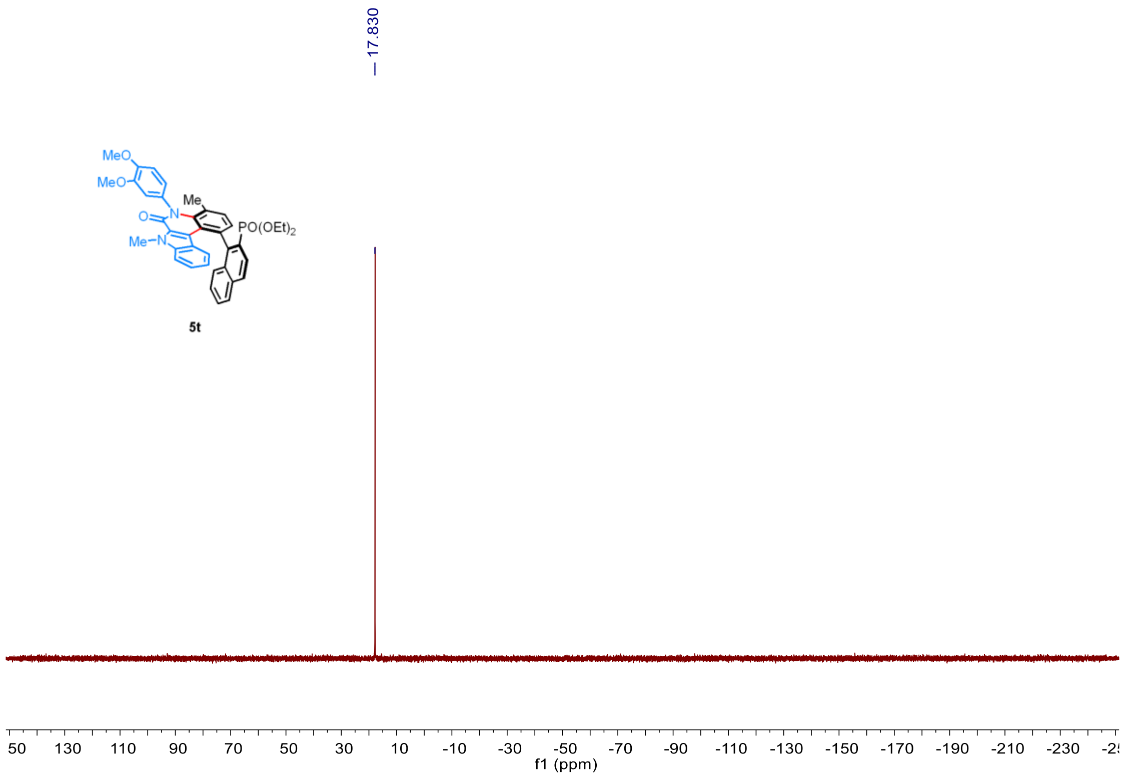

17.830
MeO
MeO
Me
O
N
Me–N
PO(OEt)$_2$
5t
50 130 110 90 70 50 30 10 -10 -30 -50 -70 -90 -110 -130 -150 -170 -190 -210 -230
f1 (ppm)

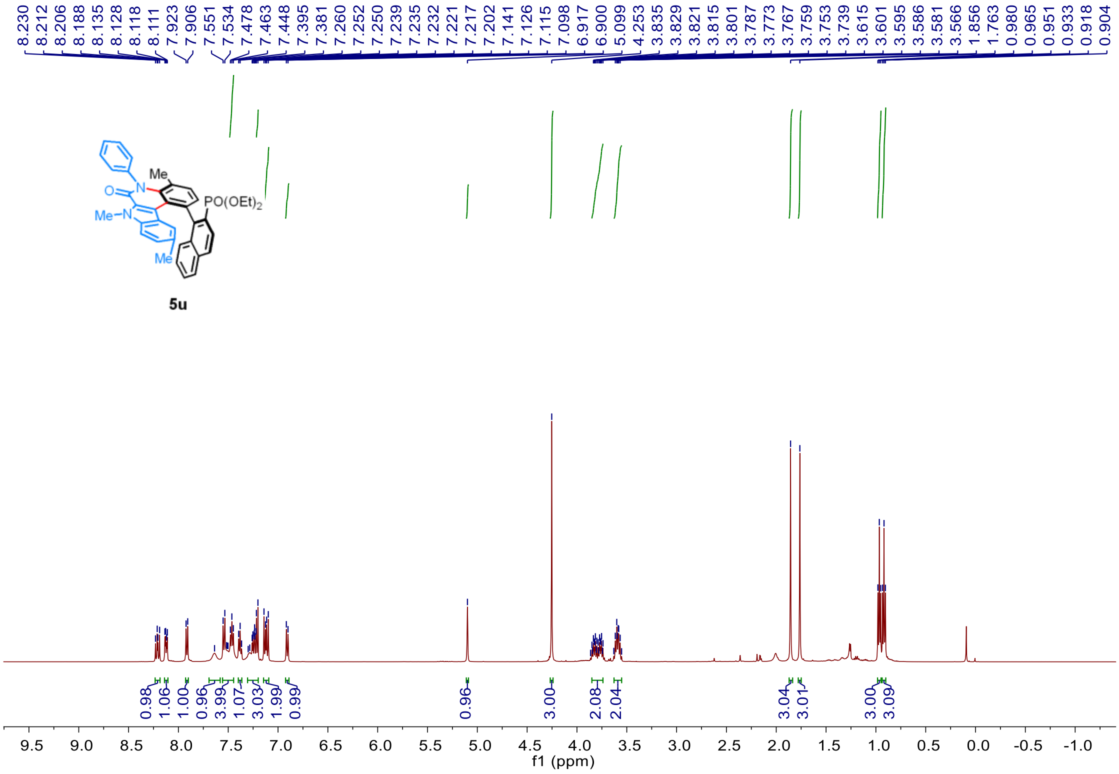
8.230
8.212
8.206
8.188
8.135
8.128
8.118
8.111
7.923
7.906
7.551
7.534
7.478
7.463
7.448
7.395
7.381
7.260
7.252
7.250
7.239
7.235
7.232
7.221
7.217
7.202
7.141
7.126
7.115
7.098
6.917
6.900
5.099
4.253
3.835
3.829
3.821
3.815
3.801
3.787
3.773
3.767
3.759
3.753
3.739
3.615
3.601
3.595
3.586
3.581
3.566
1.856
1.763
0.980
0.965
0.951
0.933
0.918
0.904
Me
O
N
PO(OEt)2
Me
N
Me
5u
0.98
1.06
1.00
0.96
3.99
1.07
3.03
1.99
0.99
0.96
3.00
2.08
2.04
3.04
3.01
3.00
3.09
9.5
9.0
8.5
8.0
7.5
7.0
6.5
6.0
5.5
5.0
4.5
4.0
3.5
3.0
2.5
2.0
1.5
1.0
0.5
0.0
-0.5
-1.0
f1 (ppm)

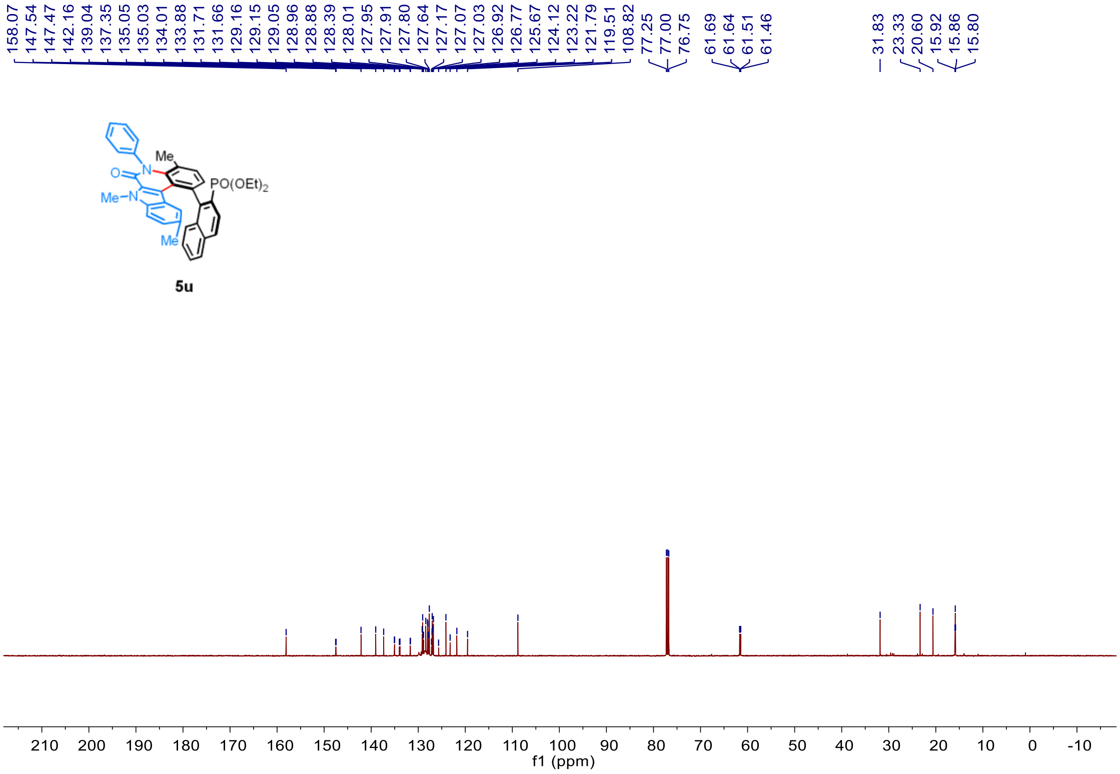
158.07
147.54
147.47
142.16
139.04
137.35
135.05
135.03
134.01
133.88
131.71
131.66
129.16
129.15
129.05
128.96
128.88
128.39
128.01
127.95
127.91
127.80
127.64
127.17
127.07
127.03
126.92
126.77
125.67
124.12
123.22
121.79
119.51
108.82
77.25
77.00
76.75
61.69
61.64
61.51
61.46
31.83
23.33
20.60
15.92
15.86
15.80
Me
O
N
PO(OEt)2
Me
N
Me
5u
210
200
190
180
170
160
150
140
130
120
110
100
90
80
70
60
50
40
30
20
10
0
-10
f1 (ppm)

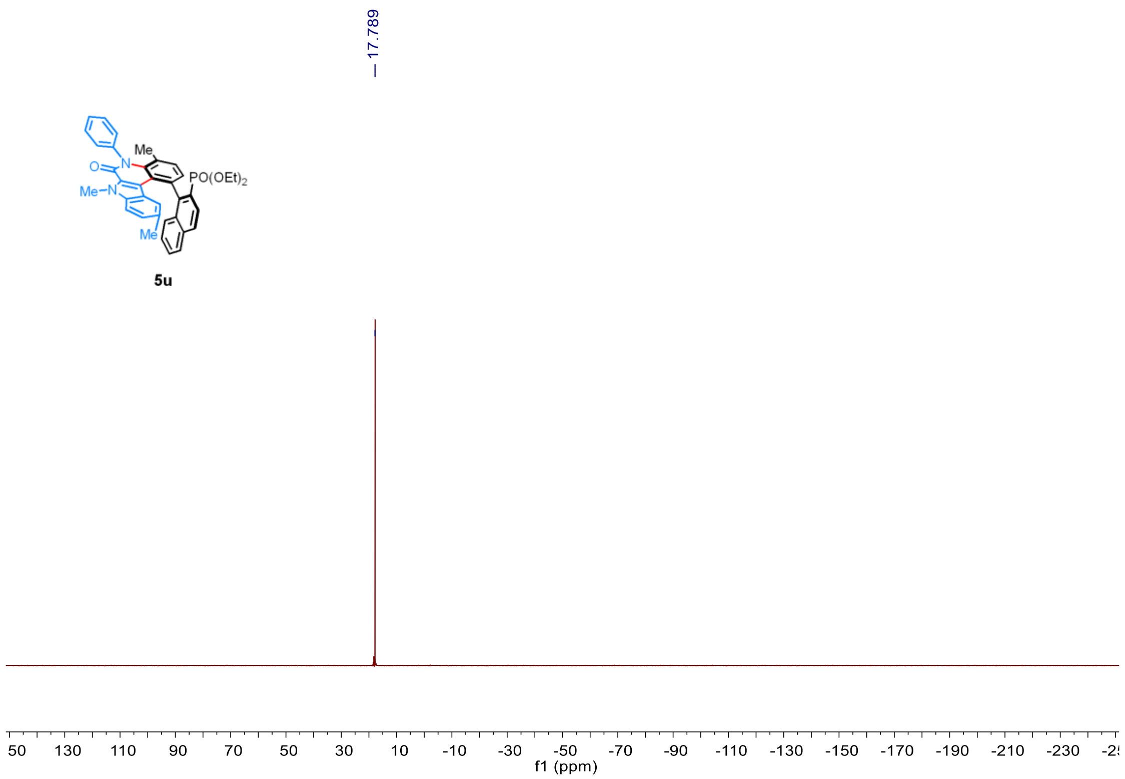

17.789
Me
N
O
Me–N
PO(OEt)2
Me
5u
f1 (ppm)

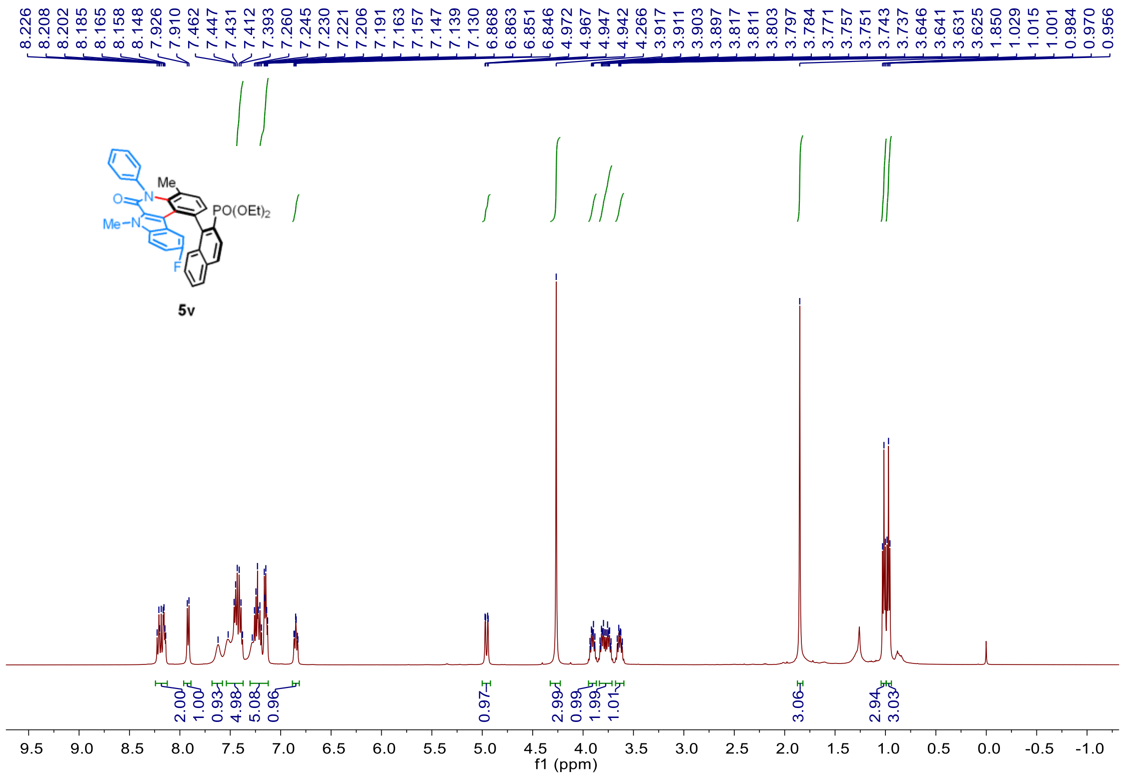

8.226 8.208 8.202 8.185 8.165 8.158 8.148 7.926 7.910 7.462 7.447 7.431 7.412 7.393 7.260 7.245 7.230 7.221 7.206 7.191 7.163 7.157 7.147 7.139 7.130 6.868 6.863 6.851 6.846 4.972 4.967 4.947 4.942 4.266 3.917 3.911 3.903 3.897 3.817 3.811 3.803 3.797 3.784 3.771 3.757 3.751 3.743 3.737 3.646 3.641 3.631 3.625 1.850 1.029 1.015 1.001 0.984 0.970 0.956
Me
N
O
Me–N
PO(OEt)2
F
5v
2.00 1.00 0.93 4.98 5.08 0.96 0.97 2.99 0.99 1.99 1.01 3.06 2.94 3.03
f1 (ppm)

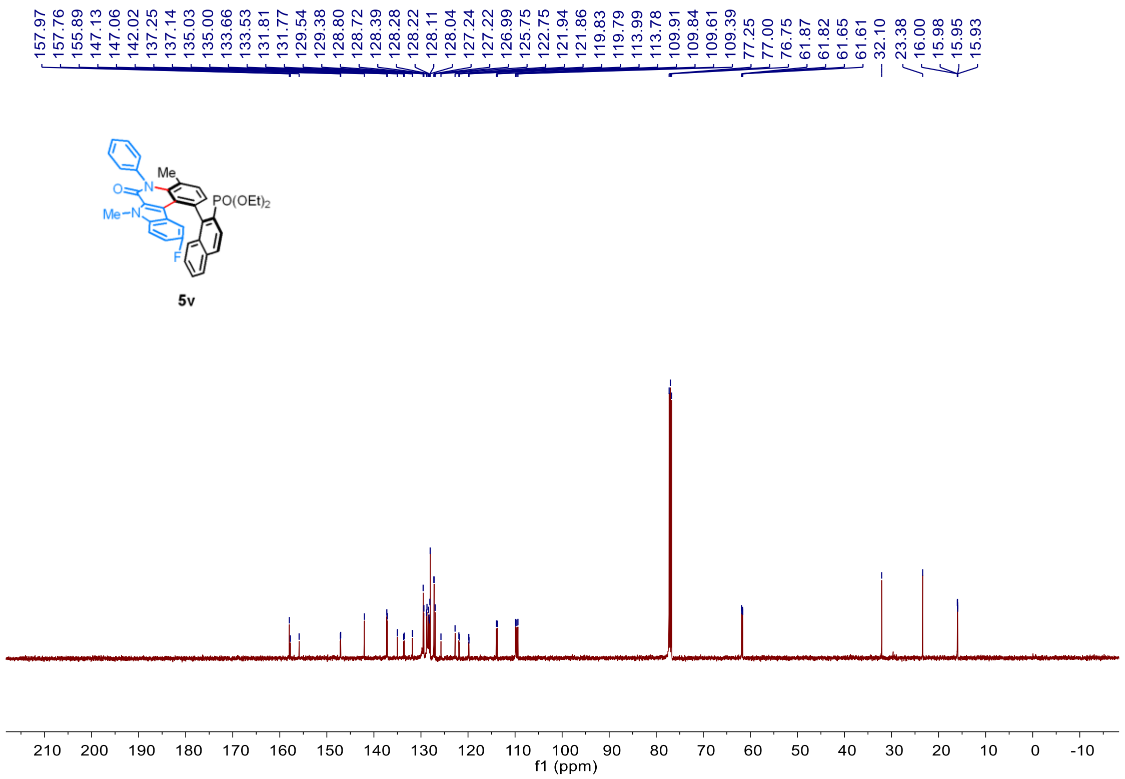
157.97
157.76
155.89
147.13
147.06
142.02
137.25
137.14
135.03
135.00
133.66
133.53
131.81
131.77
129.54
129.38
128.80
128.72
128.39
128.28
128.22
128.11
128.04
127.24
127.22
126.99
125.75
122.75
121.94
121.86
119.83
119.79
113.99
113.78
109.91
109.84
109.61
109.39
77.25
77.00
76.75
61.87
61.82
61.65
61.61
32.10
23.38
16.00
15.98
15.95
15.93
Me
N
O
PO(OEt)2
Me
N
F
5v
210
200
190
180
170
160
150
140
130
120
110
100
90
80
70
60
50
40
30
20
10
0
-10
f1 (ppm)

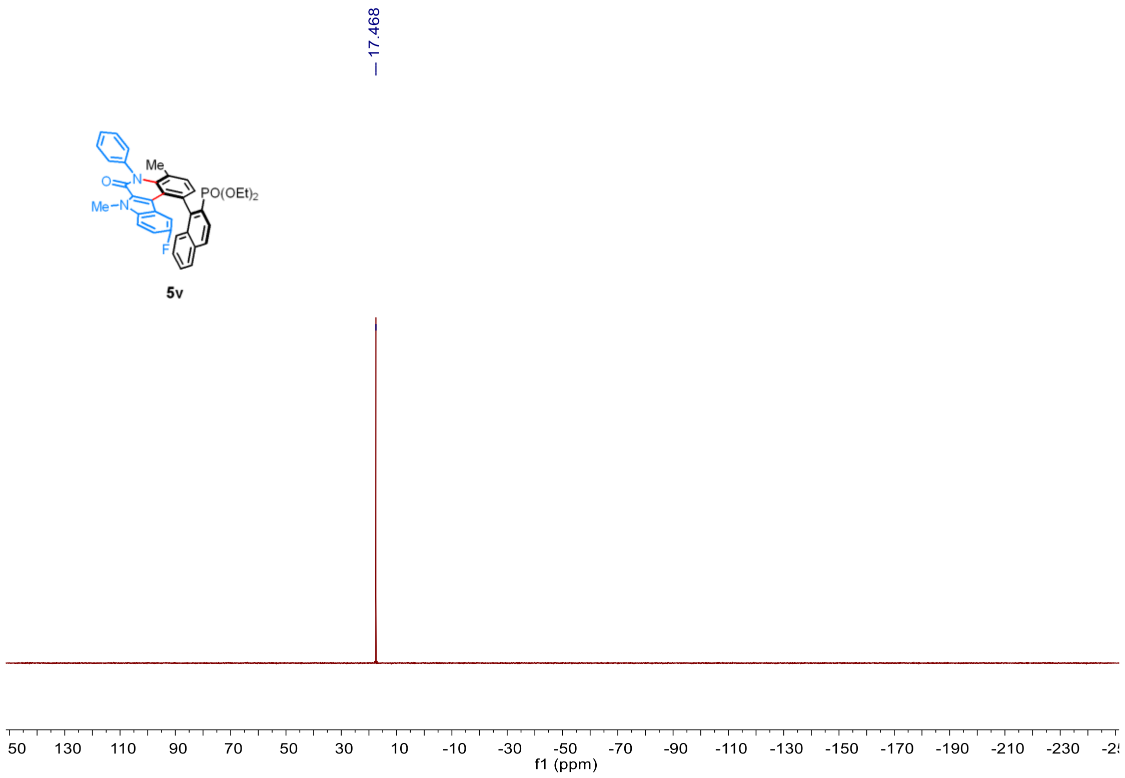
17.468
Me
N
O
PO(OEt)2
Me
N
F
5v
f1 (ppm)

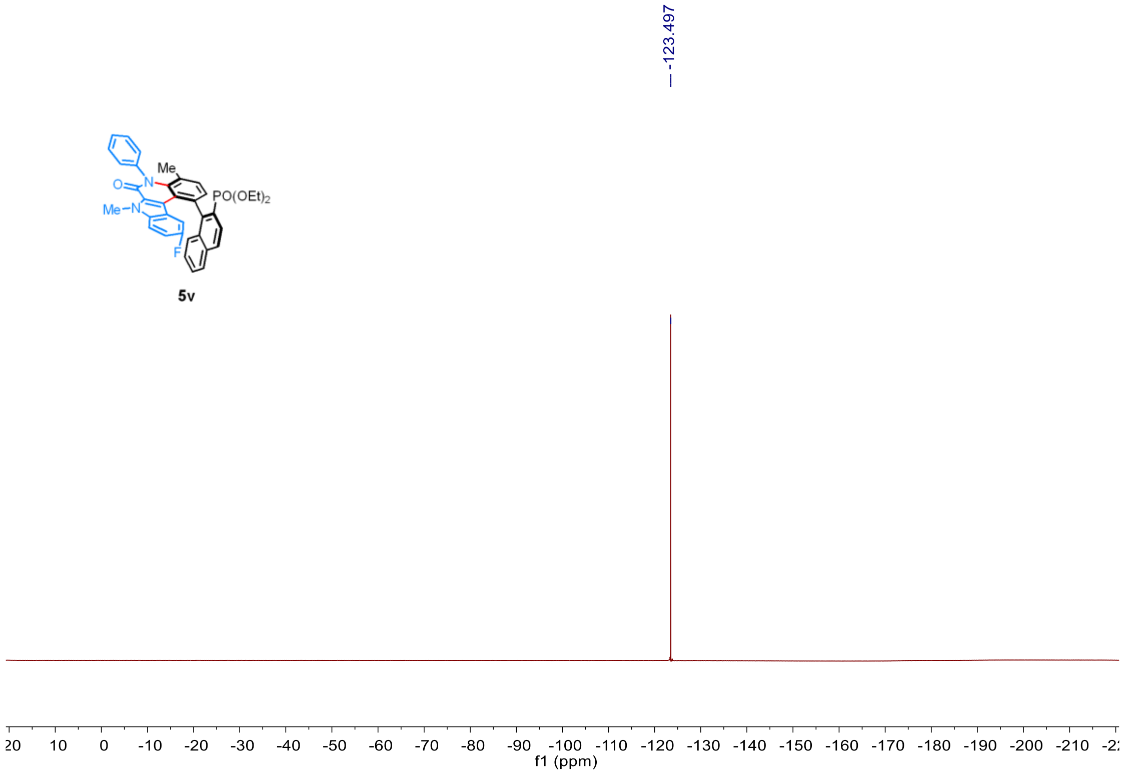
Me
PO(OEt)2
O
N
Me–N
F
5v
-123.497
20 10 0 -10 -20 -30 -40 -50 -60 -70 -80 -90 -100 -110 -120 -130 -140 -150 -160 -170 -180 -190 -200 -210
f1 (ppm)

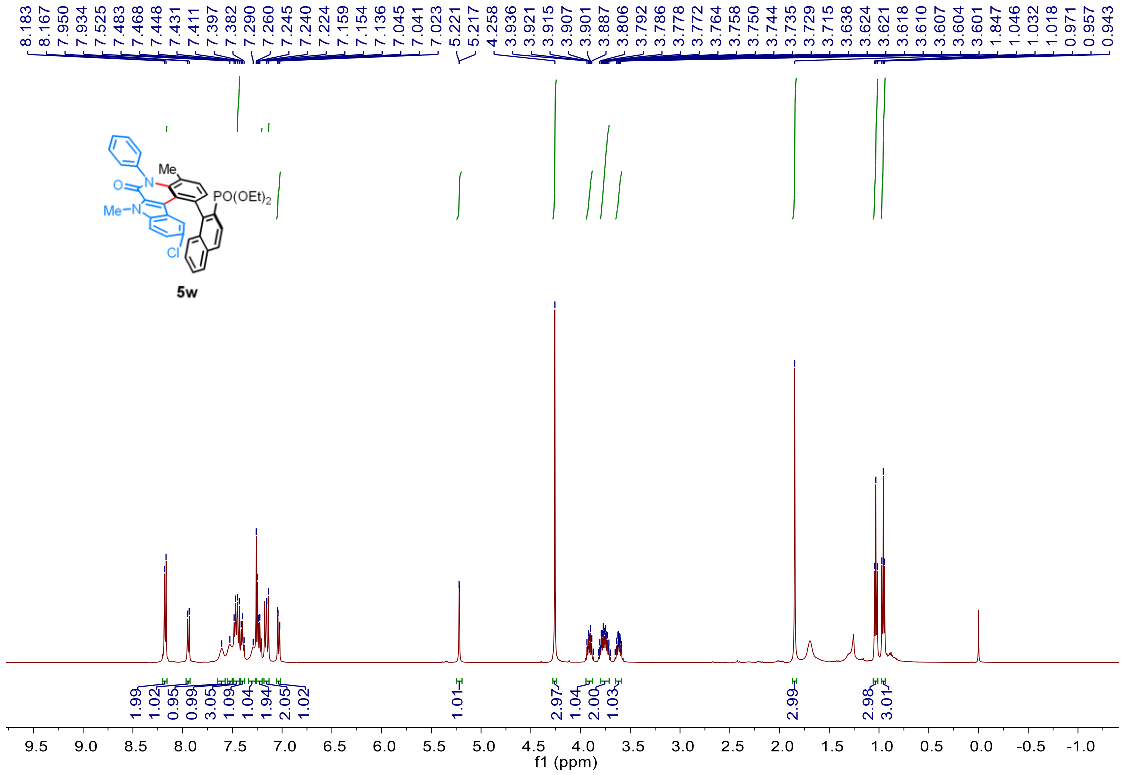
Me
PO(OEt)2
O
N
Me–N
Cl
5w
8.183 8.167 7.950 7.934 7.525 7.483 7.468 7.448 7.431 7.411 7.397 7.382 7.290 7.260 7.245 7.240 7.224 7.159 7.154 7.136 7.045 7.041 7.023 5.221 5.217 4.258 3.936 3.921 3.915 3.907 3.901 3.887 3.806 3.792 3.786 3.778 3.772 3.764 3.758 3.750 3.744 3.735 3.729 3.715 3.638 3.624 3.621 3.618 3.610 3.607 3.604 3.601 1.847 1.046 1.032 1.018 0.971 0.957 0.943
1.99 1.02 0.95 0.99 3.05 1.09 1.04 1.94 2.05 1.02 1.01 2.97 1.04 2.00 1.03 2.99 2.98 3.01
9.5 9.0 8.5 8.0 7.5 7.0 6.5 6.0 5.5 5.0 4.5 4.0 3.5 3.0 2.5 2.0 1.5 1.0 0.5 0.0 -0.5 -1.0
f1 (ppm)

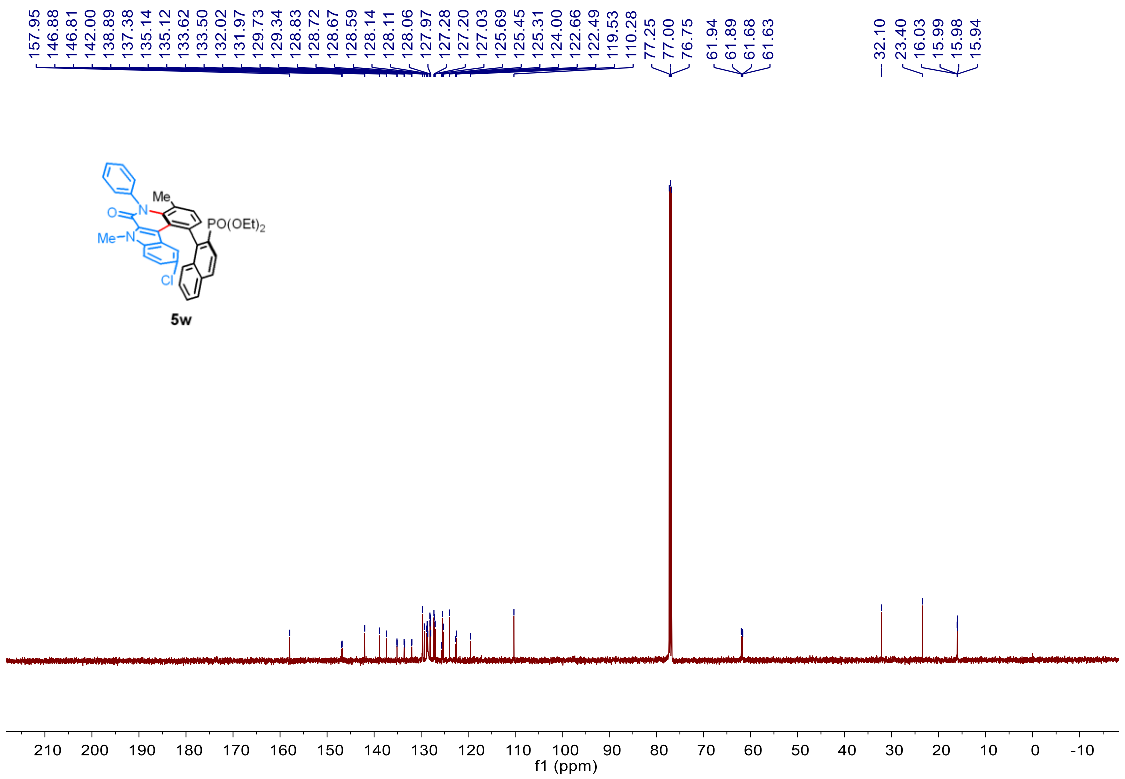
157.95
146.88
146.81
142.00
138.89
137.38
135.14
135.12
133.62
133.50
132.02
131.97
129.73
129.34
128.83
128.72
128.67
128.59
128.14
128.11
128.06
127.97
127.28
127.20
127.03
125.69
125.45
125.31
124.00
122.66
122.49
119.53
110.28
77.25
77.00
76.75
61.94
61.89
61.68
61.63
32.10
23.40
16.03
15.99
15.98
15.94
Me
N
O
Me
N
PO(OEt)2
Cl
5w
210 200 190 180 170 160 150 140 130 120 110 100 90 80 70 60 50 40 30 20 10 0 -10
f1 (ppm)

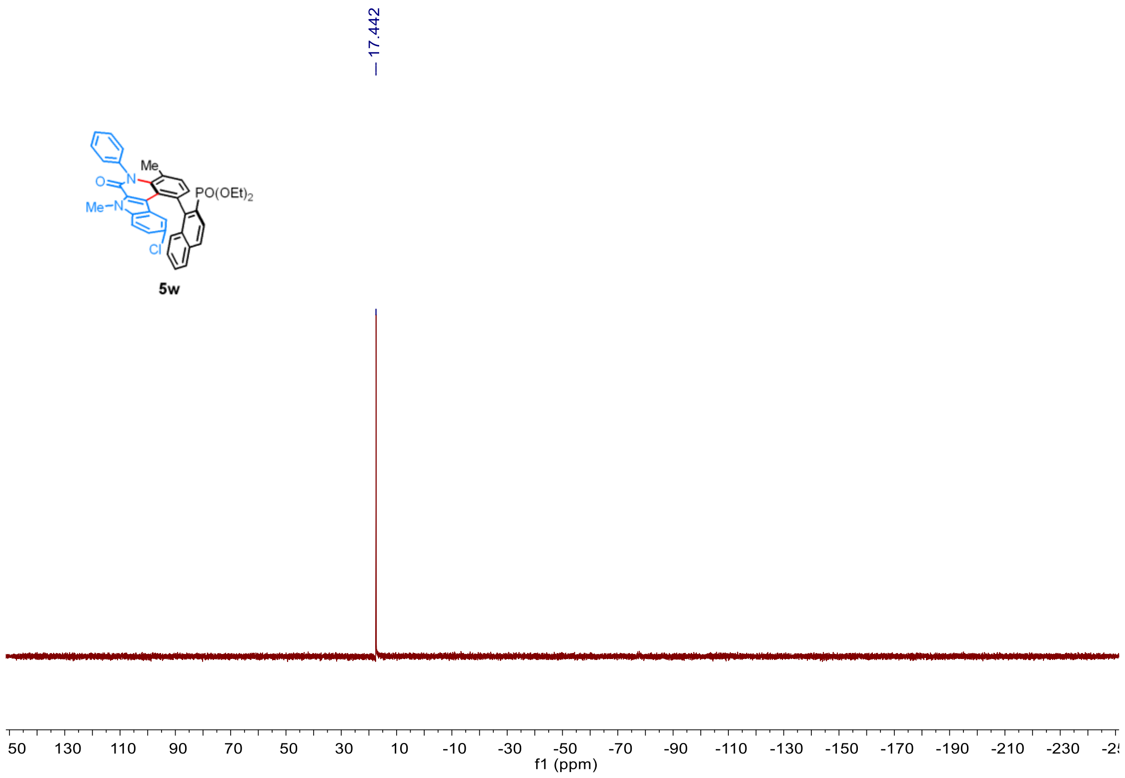
17.442
Me
N
O
Me
N
PO(OEt)2
Cl
5w
50 130 110 90 70 50 30 10 -10 -30 -50 -70 -90 -110 -130 -150 -170 -190 -210 -230
f1 (ppm)

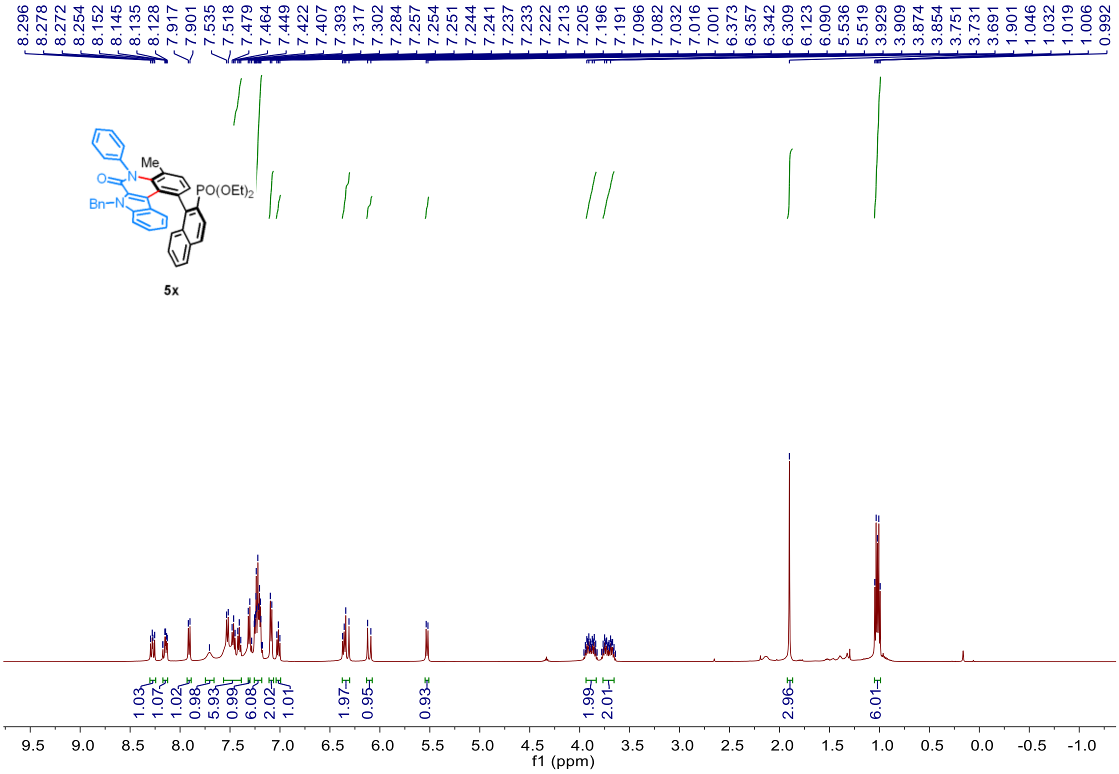
8.296 8.278 8.272 8.254 8.152 8.145 8.135 8.128 7.917 7.901 7.535 7.518 7.479 7.464 7.449 7.422 7.407 7.393 7.317 7.302 7.284 7.257 7.254 7.251 7.244 7.241 7.237 7.233 7.222 7.213 7.205 7.196 7.191 7.096 7.082 7.032 7.016 7.001 6.373 6.357 6.342 6.309 6.123 6.090 5.536 5.519 3.929 3.909 3.874 3.854 3.751 3.731 3.691 1.901 1.046 1.032 1.019 1.006 0.992
Me
PO(OEt)2
O
N
Bn–N
5x
1.03 1.07 1.02 0.98 5.93 0.99 6.08 2.02 1.01 1.97 0.95 0.93 1.99 2.01 2.96 6.01
9.5 9.0 8.5 8.0 7.5 7.0 6.5 6.0 5.5 5.0 4.5 4.0 3.5 3.0 2.5 2.0 1.5 1.0 0.5 0.0 -0.5 -1.0
f1 (ppm)

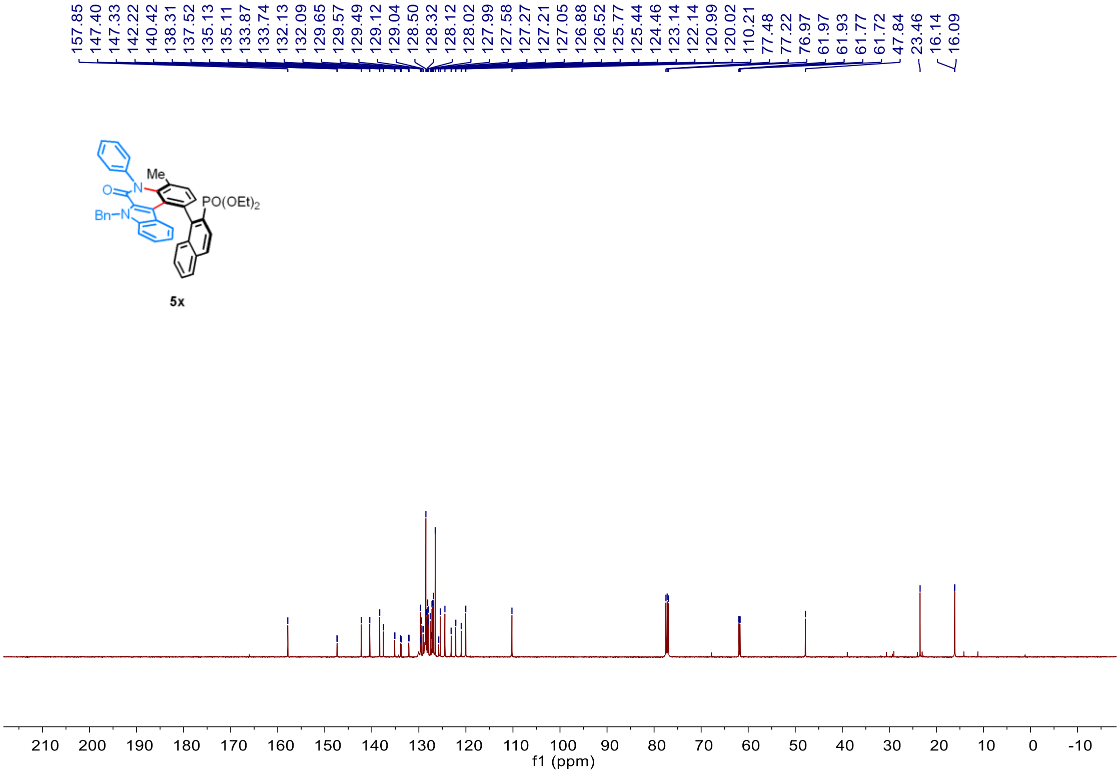
157.85 147.40 147.33 142.22 140.42 138.31 137.52 135.13 135.11 133.87 133.74 132.13 132.09 129.65 129.57 129.49 129.12 129.04 128.50 128.32 128.12 128.02 127.99 127.58 127.27 127.21 127.05 126.88 126.52 125.77 125.44 124.46 123.14 122.14 120.99 120.02 110.21 77.48 77.22 76.97 61.97 61.93 61.77 61.72 47.84 23.46 16.14 16.09
Me
PO(OEt)2
O
N
Bn–N
5x
210 200 190 180 170 160 150 140 130 120 110 100 90 80 70 60 50 40 30 20 10 0 -10
f1 (ppm)

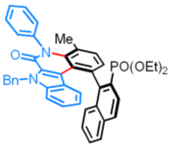

Me
O
N
Bn–N
PO(OEt)2

5x

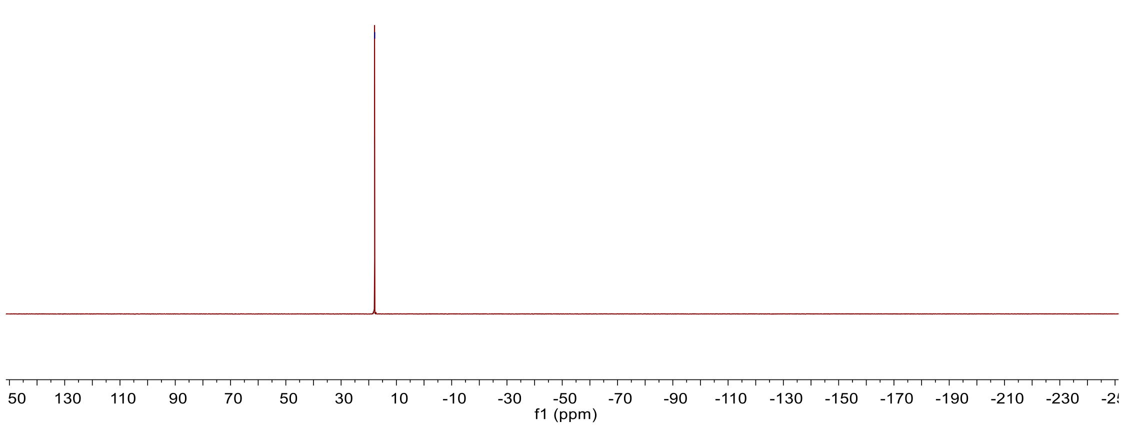

17.876
f1 (ppm)

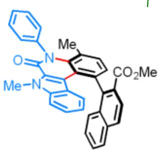

Me
O
N
Me–N
CO2Me

5y

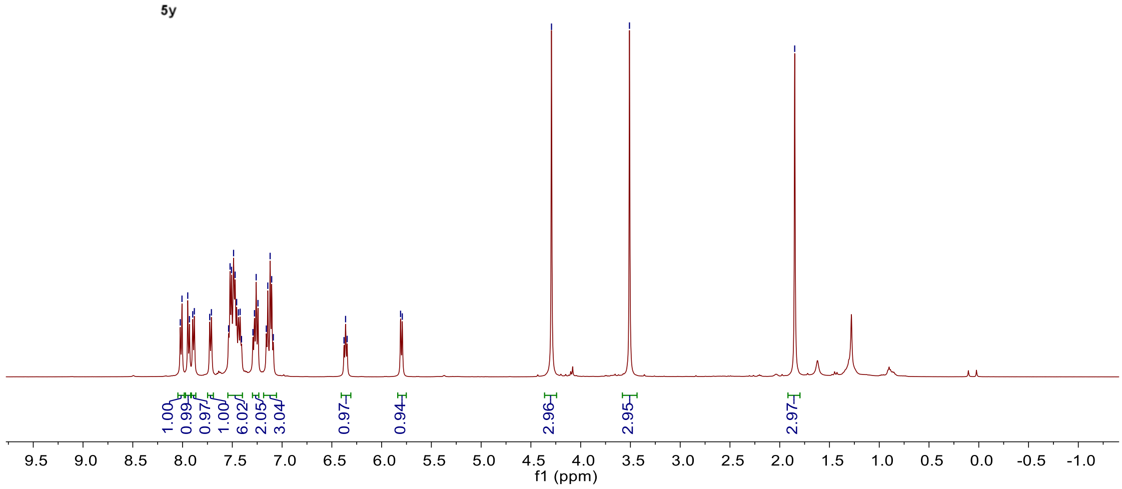

8.023
8.006
7.948
7.931
7.898
7.882
7.727
7.710
7.537
7.522
7.508
7.487
7.472
7.456
7.439
7.421
7.408
7.293
7.277
7.260
7.242
7.158
7.143
7.120
7.104
7.088
6.378
6.362
6.346
5.809
5.792
4.293
3.510
1.850
1.00
0.99
0.97
1.00
6.02
2.05
3.04
0.97
0.94
2.96
2.95
2.97
f1 (ppm)

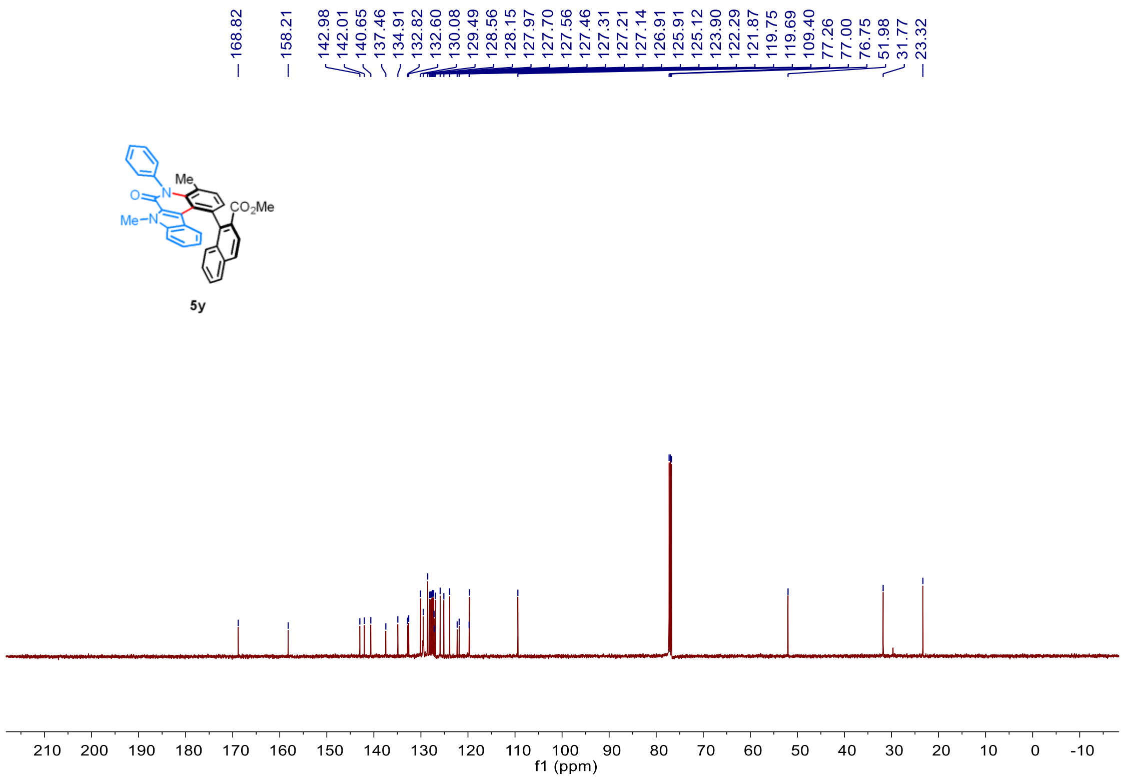
168.82
158.21
142.98
142.01
140.65
137.46
134.91
132.82
132.60
130.08
129.49
128.56
128.15
127.97
127.70
127.56
127.46
127.31
127.21
127.14
126.91
125.91
125.12
123.90
122.29
121.87
119.75
119.69
109.40
77.26
77.00
76.75
51.98
31.77
23.32
Me
CO2Me
O
N
Me–N
210 200 190 180 170 160 150 140 130 120 110 100 90 80 70 60 50 40 30 20 10 0 -10
f1 (ppm)

5y

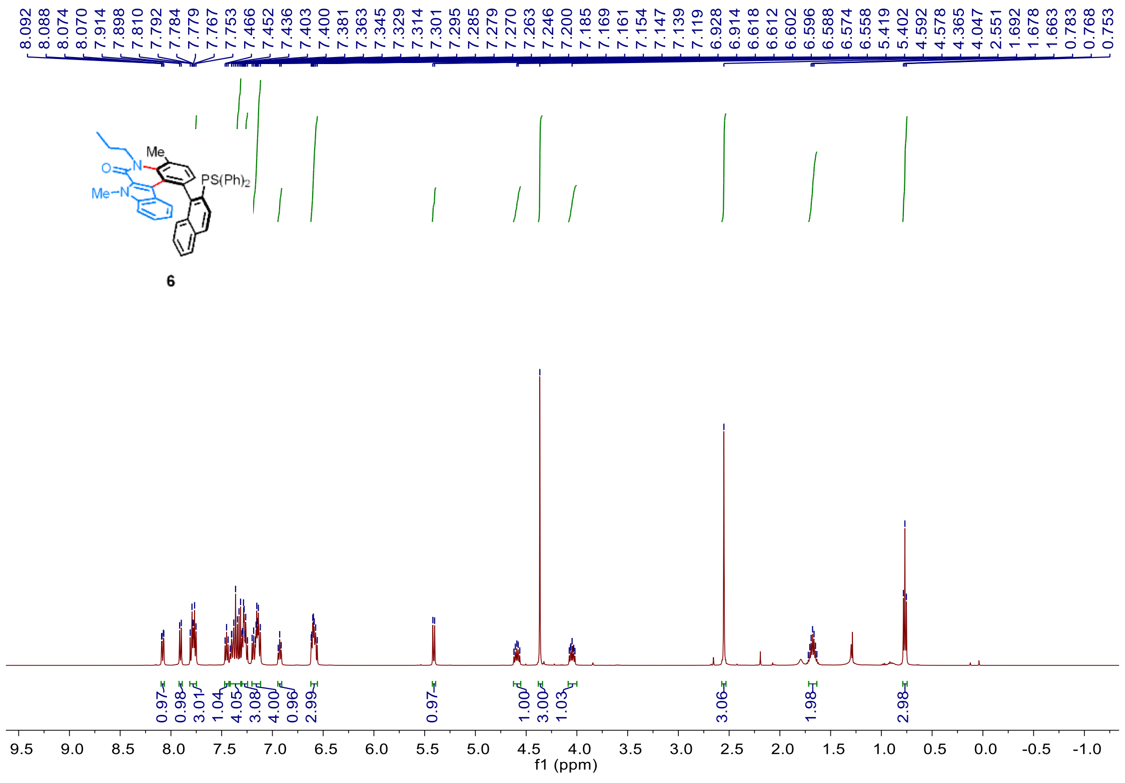
8.092
8.088
8.074
8.070
7.914
7.898
7.810
7.792
7.784
7.779
7.767
7.753
7.466
7.452
7.436
7.403
7.400
7.381
7.363
7.345
7.329
7.314
7.301
7.295
7.285
7.279
7.270
7.263
7.246
7.200
7.185
7.169
7.161
7.154
7.147
7.139
7.119
6.928
6.914
6.618
6.612
6.602
6.596
6.588
6.574
6.558
5.419
5.402
4.592
4.578
4.365
4.047
2.551
1.692
1.678
1.663
0.783
0.768
0.753
Me
PS(Ph)2
O
N
Me–N
0.97
0.98
3.01
1.04
4.05
3.08
4.00
0.96
2.99
0.97
1.00
3.00
1.03
3.06
1.98
2.98
9.5 9.0 8.5 8.0 7.5 7.0 6.5 6.0 5.5 5.0 4.5 4.0 3.5 3.0 2.5 2.0 1.5 1.0 0.5 0.0 -0.5 -1.0
f1 (ppm)

6

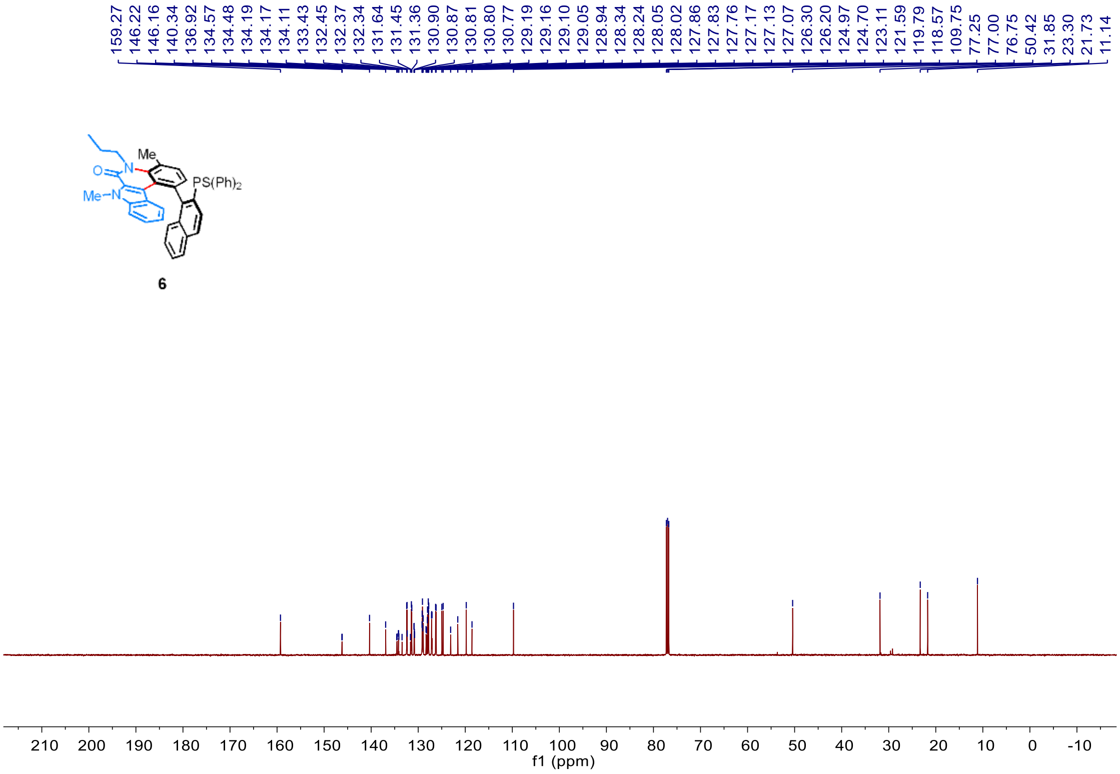
159.27
146.22
146.16
140.34
136.92
134.57
134.48
134.19
134.17
134.11
133.43
132.45
132.37
132.34
131.64
131.45
131.36
130.90
130.87
130.81
130.80
130.77
129.19
129.16
129.10
129.05
128.94
128.34
128.24
128.05
128.02
127.86
127.83
127.76
127.17
127.13
127.07
126.30
126.20
124.97
124.70
123.11
121.59
119.79
118.57
109.75
77.25
77.00
76.75
50.42
31.85
23.30
21.73
11.14
Me
O
N
Me–N
PS(Ph)2
210 200 190 180 170 160 150 140 130 120 110 100 90 80 70 60 50 40 30 20 10 0 -10
f1 (ppm)

6

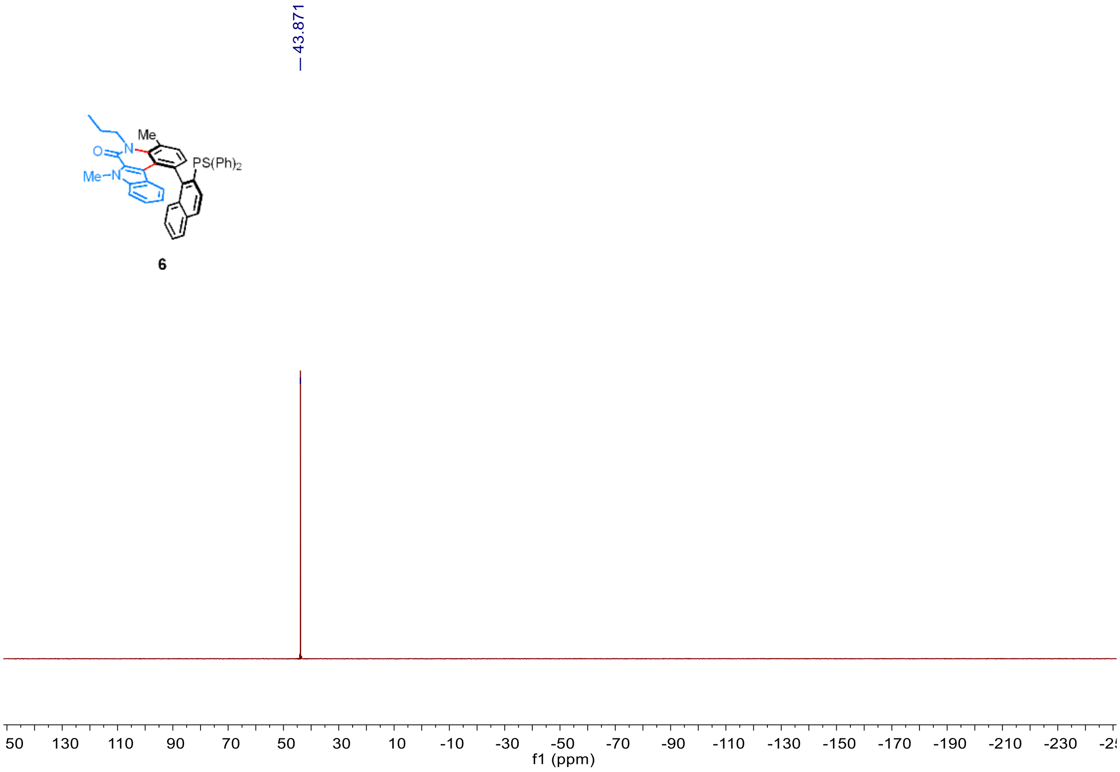
43.871
Me
O
N
Me–N
PS(Ph)2
50 130 110 90 70 50 30 10 -10 -30 -50 -70 -90 -110 -130 -150 -170 -190 -210 -230
f1 (ppm)

6

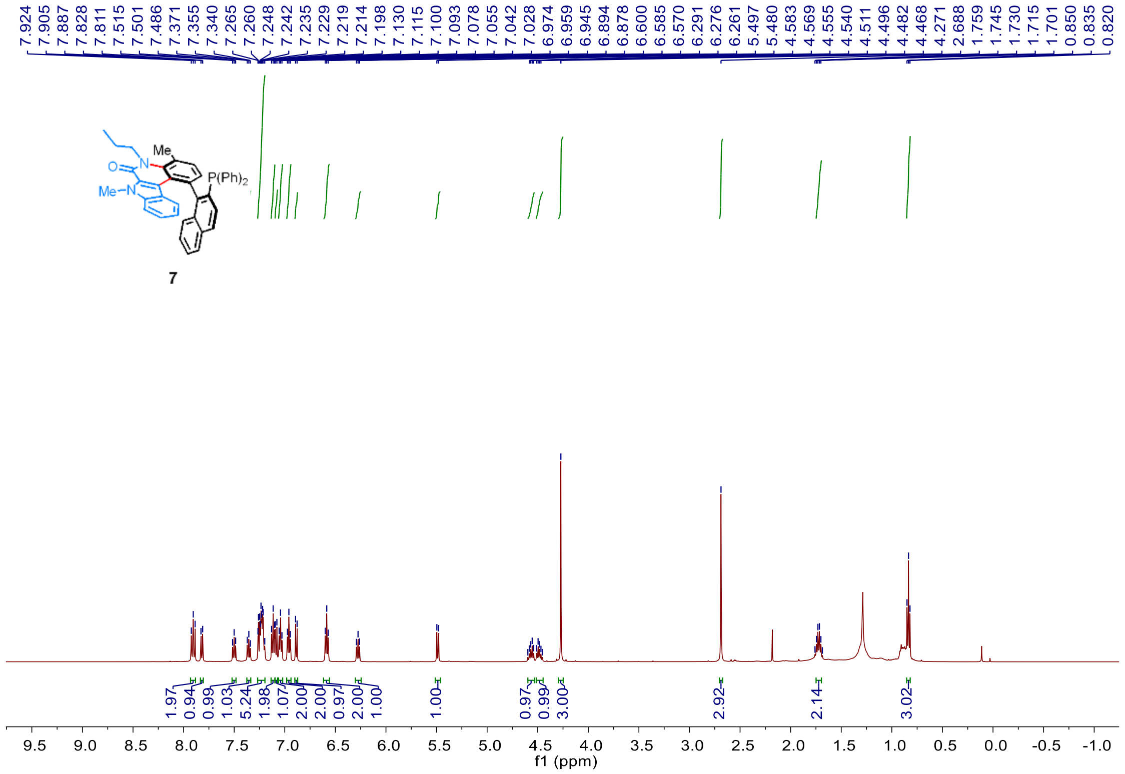
7.924 7.905 7.887 7.828 7.811 7.515 7.501 7.486 7.371 7.355 7.340 7.265 7.260 7.248 7.242 7.235 7.229 7.219 7.214 7.198 7.130 7.115 7.100 7.093 7.078 7.055 7.042 7.028 6.974 6.959 6.945 6.894 6.878 6.600 6.585 6.570 6.291 6.276 6.261 5.497 5.480 4.583 4.569 4.555 4.540 4.511 4.496 4.482 4.468 4.271 2.688 1.759 1.745 1.730 1.715 1.701 0.850 0.835 0.820
Me
O
N
P(Ph)2
Me
1.97 0.94 0.99 1.03 5.24 1.98 1.07 2.00 2.00 0.97 2.00 1.00 1.00 0.97 0.99 3.00 2.92 2.14 3.02
f1 (ppm)

7

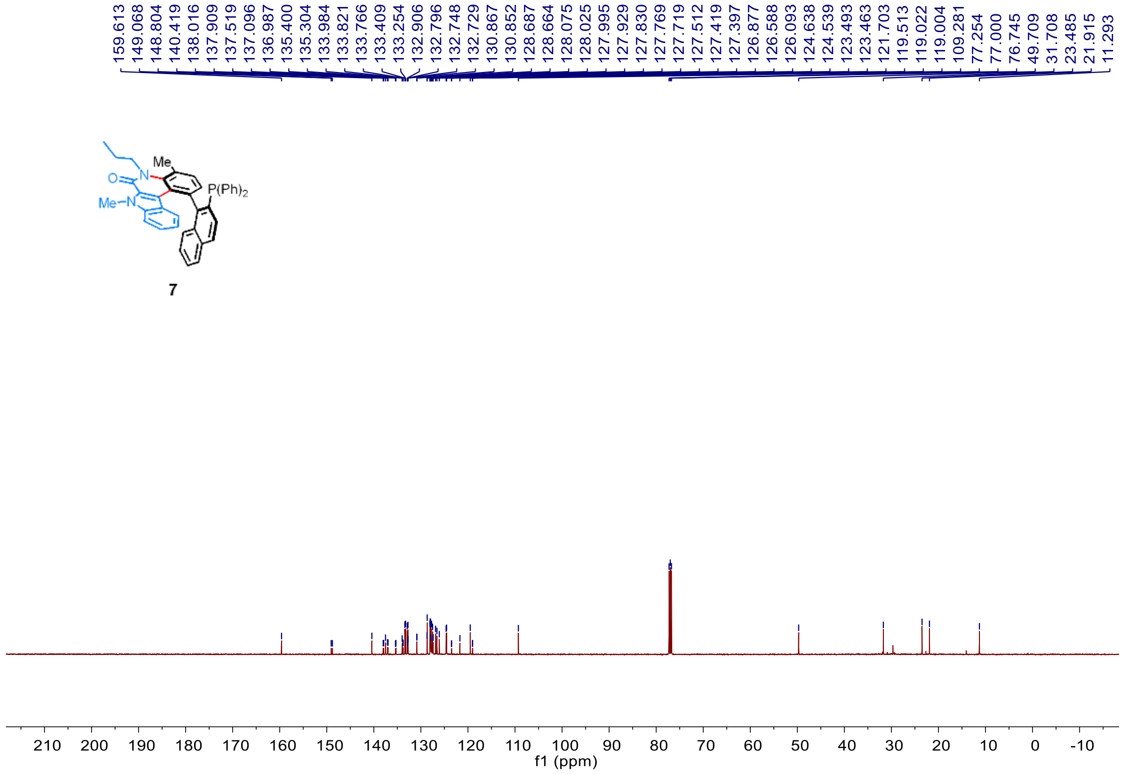
159.613 149.068 148.804 140.419 138.016 137.909 137.519 137.096 136.987 135.400 135.304 133.984 133.821 133.766 133.409 133.254 132.906 132.796 132.748 132.729 130.867 130.852 128.687 128.664 128.075 128.025 127.995 127.929 127.830 127.769 127.719 127.512 127.419 127.397 126.877 126.588 126.093 124.638 124.539 123.493 123.463 121.703 119.513 119.022 119.004 109.281 77.254 77.000 76.745 49.709 31.708 23.485 21.915 11.293
Me
O
N
P(Ph)2
Me
f1 (ppm)

7

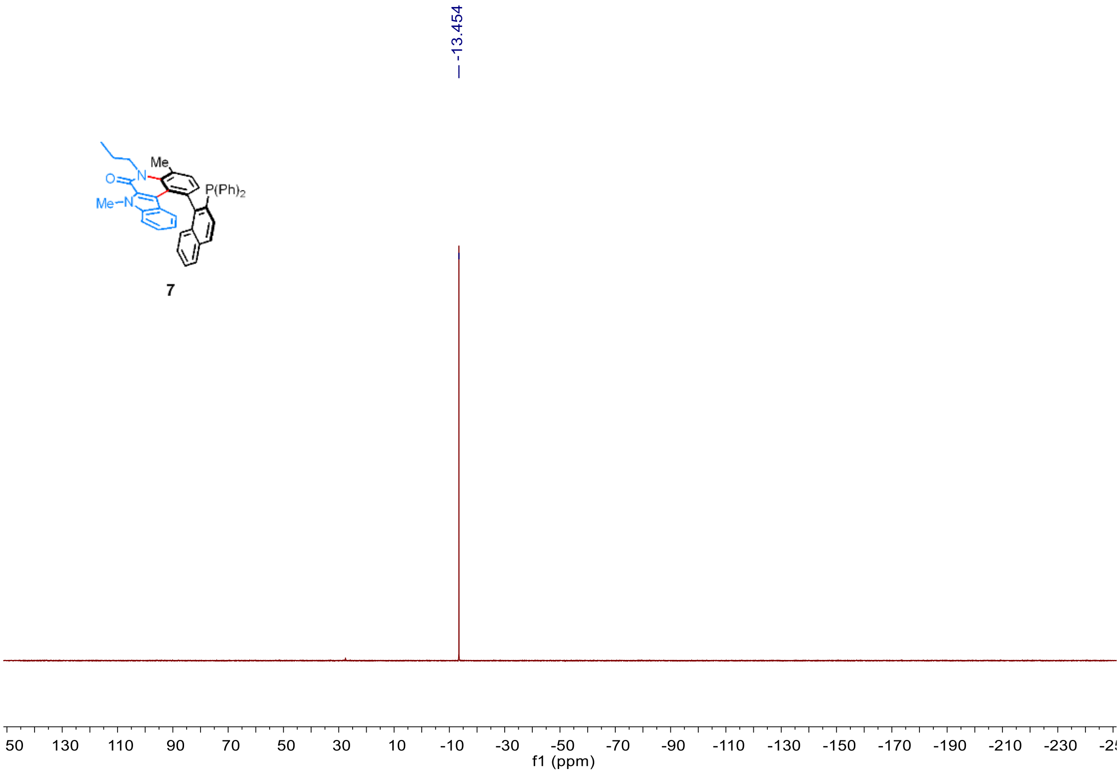

-13.454
Me
O
N
Me
N
P(Ph)2
7
50 130 110 90 70 50 30 10 -10 -30 -50 -70 -90 -110 -130 -150 -170 -190 -210 -230
f1 (ppm)

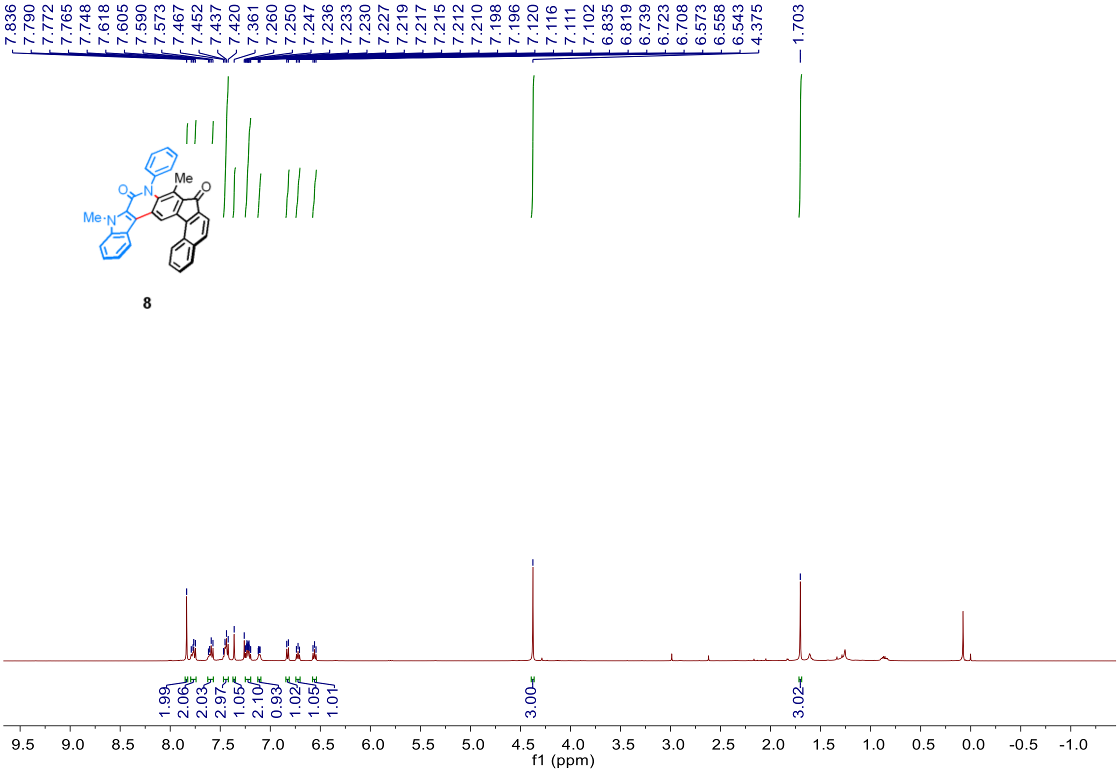

7.836 7.790 7.772 7.765 7.748 7.618 7.605 7.590 7.573 7.467 7.452 7.437 7.420 7.361 7.260 7.250 7.247 7.236 7.233 7.230 7.227 7.219 7.217 7.215 7.212 7.210 7.198 7.196 7.120 7.116 7.111 7.102 6.835 6.819 6.739 6.723 6.708 6.573 6.558 6.543 4.375
1.703
O
N
Me
O
Me
N
8
1.99 2.06 2.03 2.97 1.05 2.10 0.93 1.02 1.05 1.01
3.00
3.02
9.5 9.0 8.5 8.0 7.5 7.0 6.5 6.0 5.5 5.0 4.5 4.0 3.5 3.0 2.5 2.0 1.5 1.0 0.5 0.0 -0.5 -1.0
f1 (ppm)

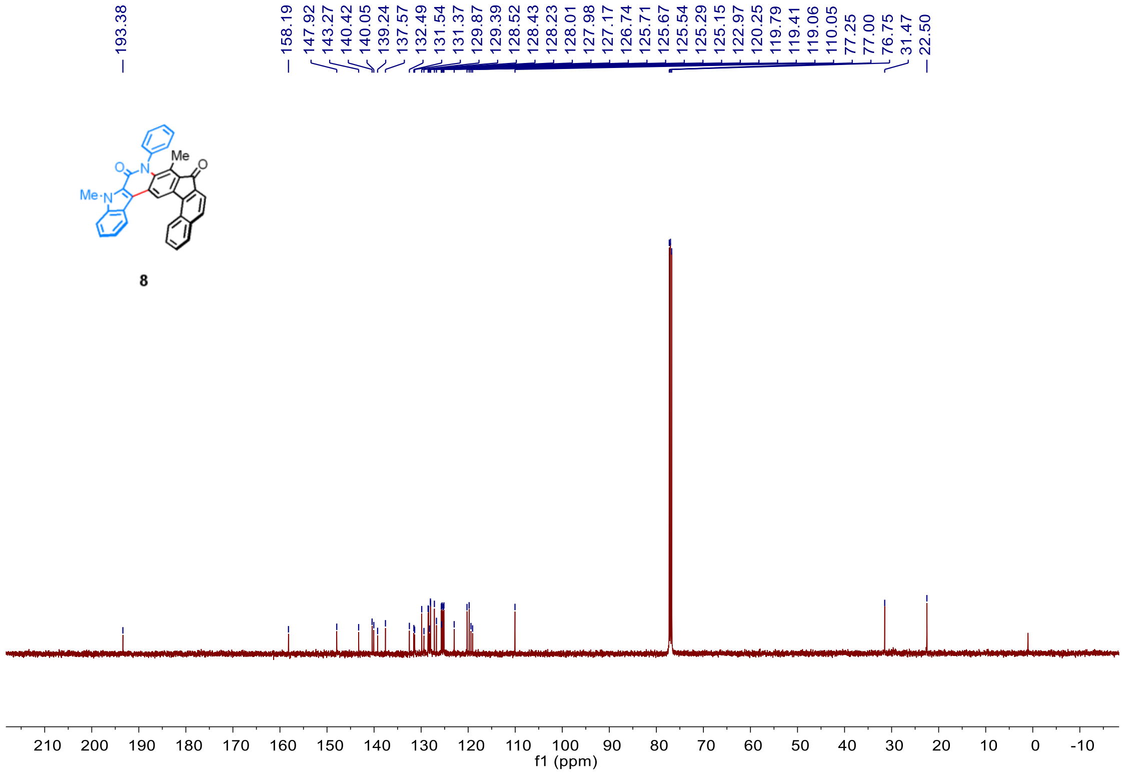
193.38
158.19
147.92
143.27
140.42
140.05
139.24
137.57
132.49
131.54
131.37
129.87
129.39
128.52
128.43
128.23
128.01
127.98
127.17
126.74
125.71
125.67
125.54
125.29
125.15
122.97
120.25
119.79
119.41
119.06
110.05
77.25
77.00
76.75
31.47
22.50
Me
O
N
Me-N
O
8
210 200 190 180 170 160 150 140 130 120 110 100 90 80 70 60 50 40 30 20 10 0 -10
f1 (ppm)

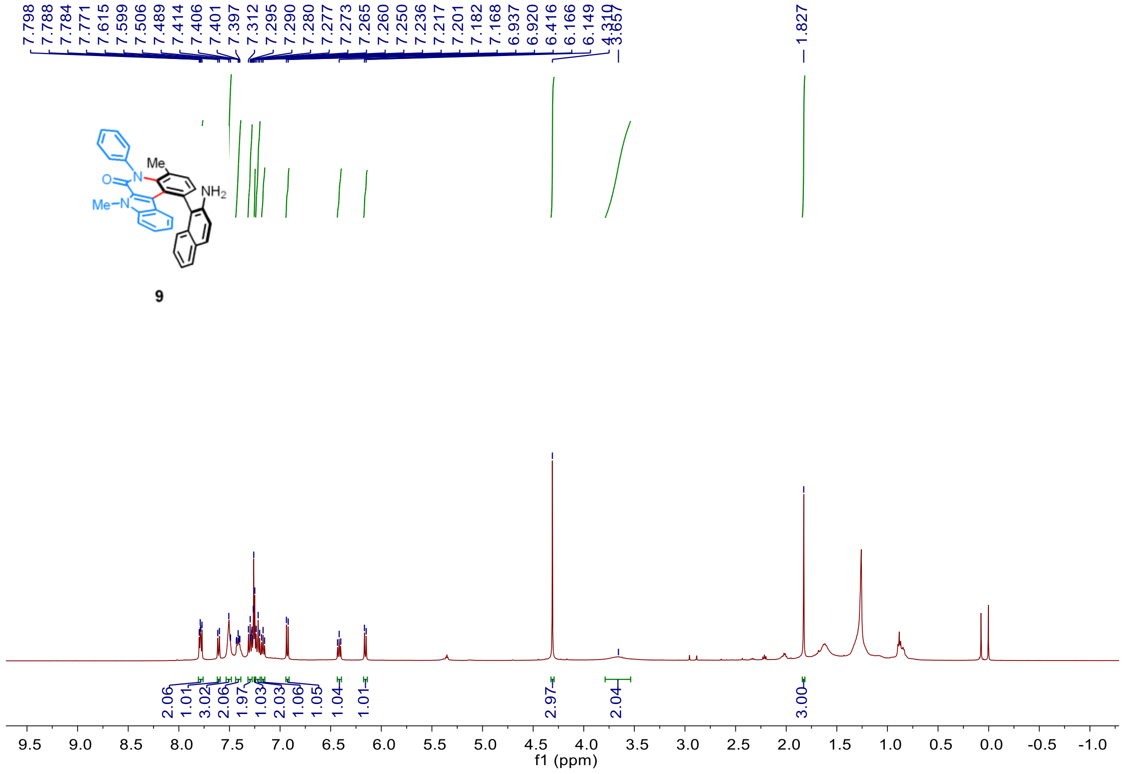
7.798
7.788
7.784
7.771
7.615
7.599
7.506
7.489
7.414
7.406
7.401
7.397
7.312
7.295
7.290
7.280
7.277
7.273
7.265
7.260
7.250
7.236
7.217
7.201
7.182
7.168
6.937
6.920
6.416
6.166
6.149
4.310
3.657
1.827
Me
O
N
Me-N
NH_2
9
2.06
1.01
3.02
2.06
1.97
1.03
2.03
1.06
1.05
1.04
1.01
2.97
2.04
3.00
9.5 9.0 8.5 8.0 7.5 7.0 6.5 6.0 5.5 5.0 4.5 4.0 3.5 3.0 2.5 2.0 1.5 1.0 0.5 0.0 -0.5 -1.0
f1 (ppm)

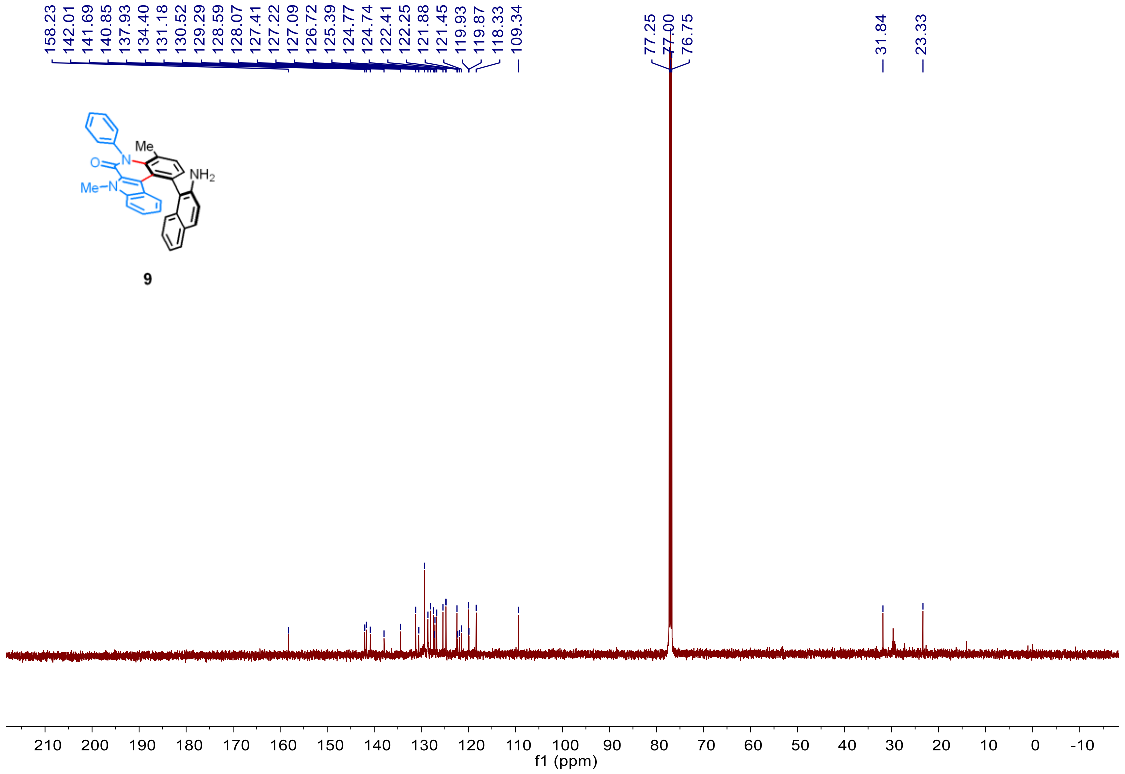
158.23
142.01
141.69
140.85
137.93
134.40
131.18
130.52
129.29
128.59
128.07
127.41
127.22
127.09
126.72
125.39
124.77
124.74
122.41
122.25
121.88
121.45
119.93
119.87
118.33
109.34
77.25
77.00
76.75
31.84
23.33
Me
O
N
Me–N
NH2
9
210 200 190 180 170 160 150 140 130 120 110 100 90 80 70 60 50 40 30 20 10 0 -10
f1 (ppm)

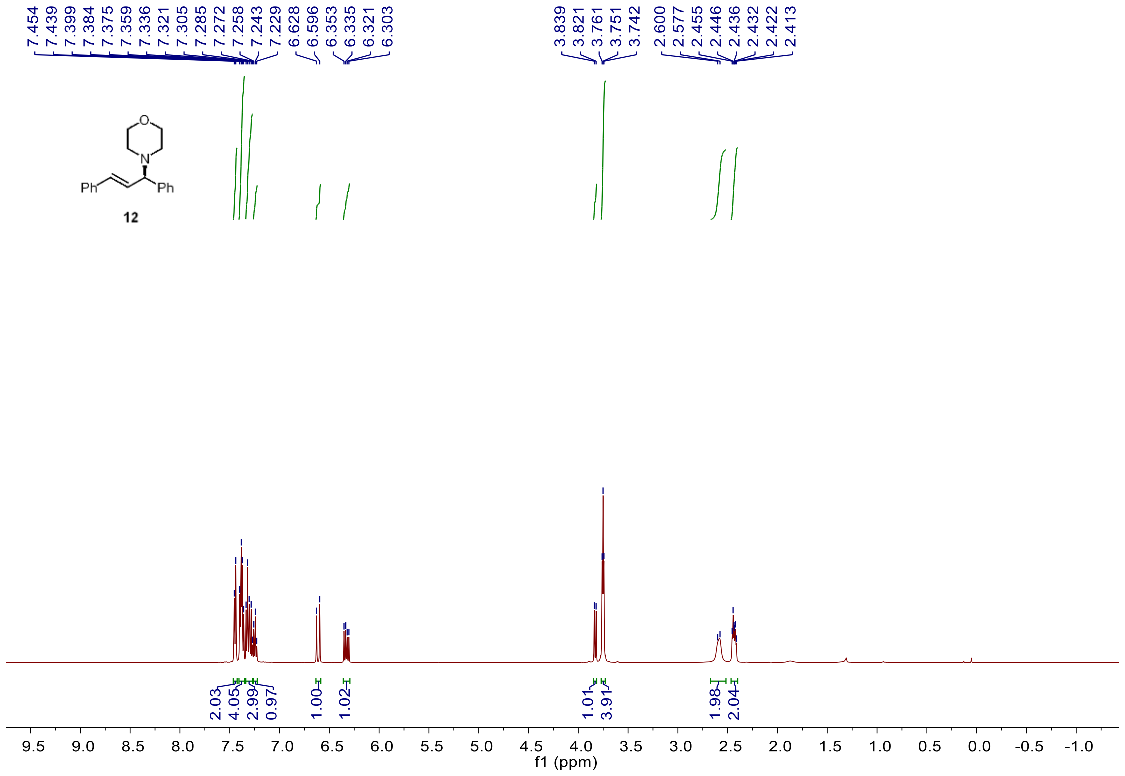
7.454
7.439
7.399
7.384
7.375
7.359
7.336
7.321
7.305
7.285
7.272
7.258
7.243
7.229
6.628
6.596
6.353
6.335
6.321
6.303
3.839
3.821
3.761
3.751
3.742
2.600
2.577
2.455
2.446
2.436
2.432
2.422
2.413
O
N
Ph
Ph
12
2.03
4.05
2.99
0.97
1.00
1.02
1.01
3.91
1.98
2.04
9.5 9.0 8.5 8.0 7.5 7.0 6.5 6.0 5.5 5.0 4.5 4.0 3.5 3.0 2.5 2.0 1.5 1.0 0.5 0.0 -0.5 -1.0
f1 (ppm)

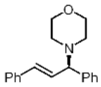
O
N
Ph
Ph

12

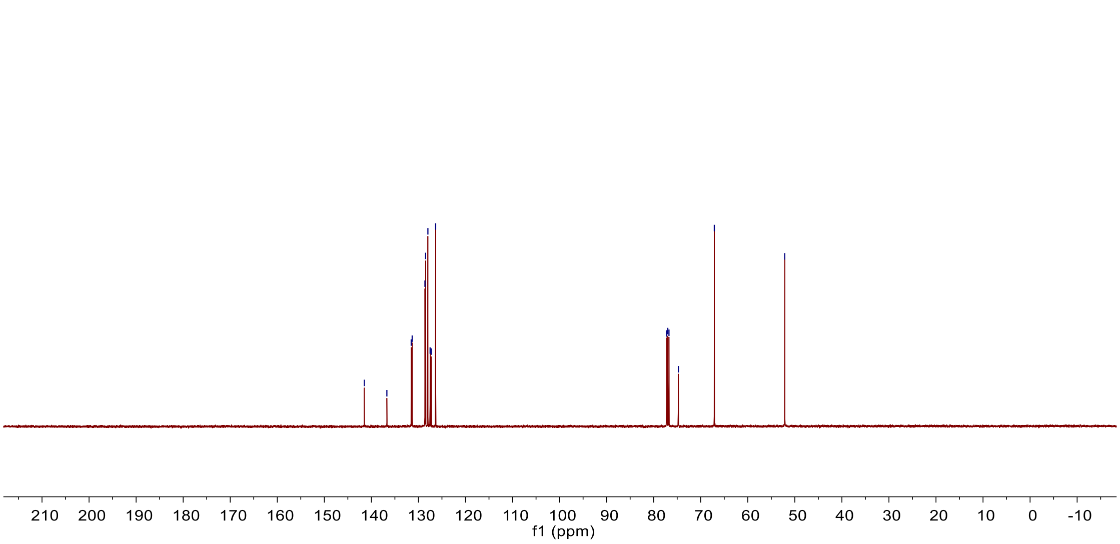
141.50
136.70
131.53
131.33
128.62
128.47
127.99
127.52
127.26
126.34
77.25
77.00
76.75
74.75
67.11
52.14
210 200 190 180 170 160 150 140 130 120 110 100 90 80 70 60 50 40 30 20 10 0 -10
f1 (ppm)

11. HPLC Charts

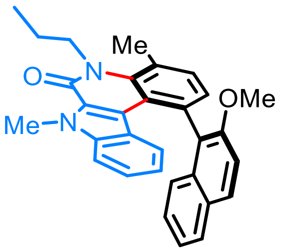

3h

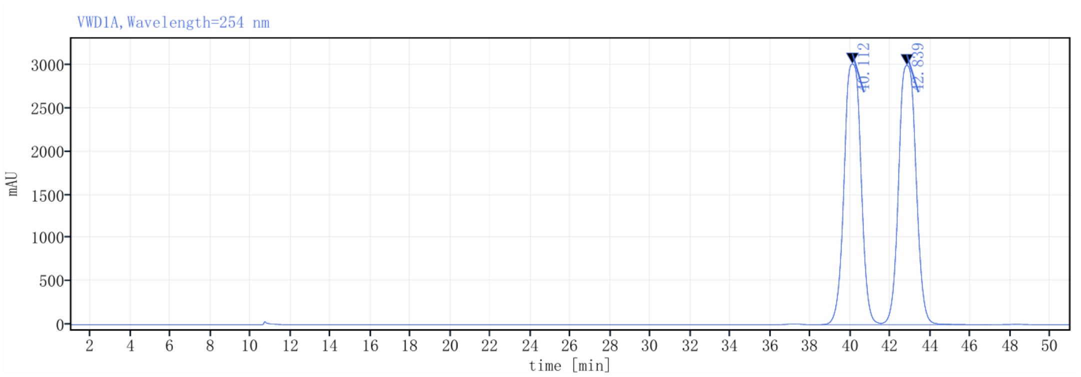

Signal:	VWD1A, Wavelength=254 nm			
RetTime [min]	Width [min]	Area [mAu*s]	Height [mAu]	Area [%]
40.112	3.41	177574.38	3013.92	49.49
42.839	5.31	181220.60	2998.05	50.51

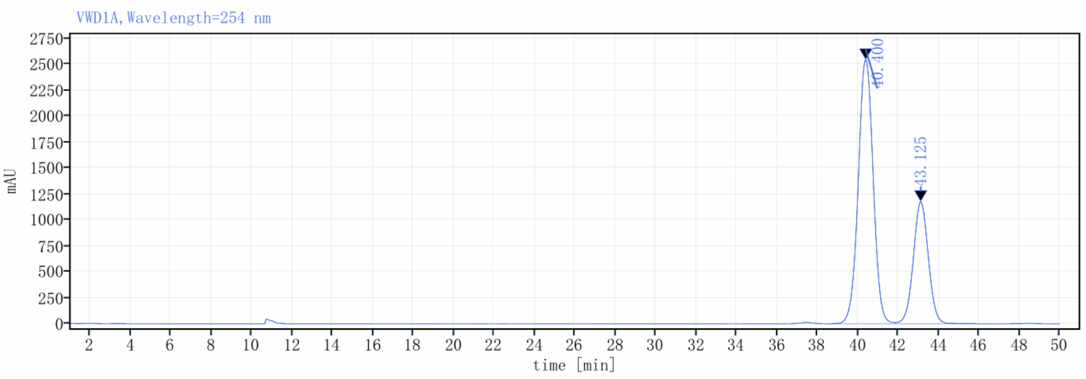

Signal:	VWD1A, Wavelength=254 nm			
RetTime [min]	Width [min]	Area [mAu*s]	Height [mAu]	Area [%]
40.400	3.44	131076.59	2544.27	68.31
43.125	5.10	60806.43	1175.53	31.69

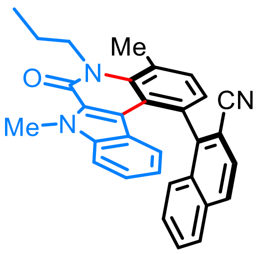

3i

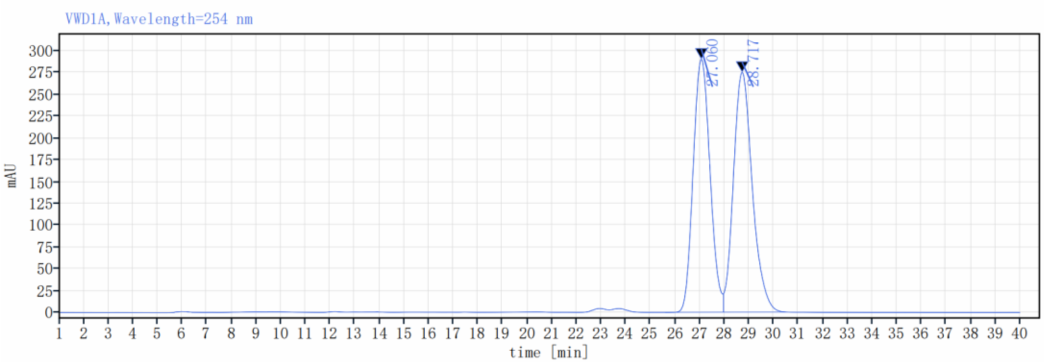

Signal: VWD1A, Wavelength=254 nm

RetTime [min]	Width [min]	Area [mAu*s]	Height [mAu]	Area [%]
27.060	2.37	13859.96	290.40	48.59
28.717	2.51	14661.93	275.17	51.41

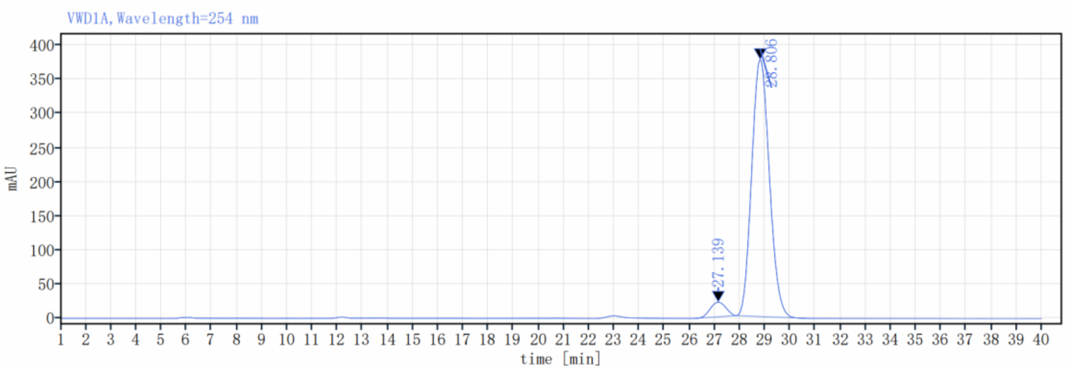

Signal: VWD1A, Wavelength=254 nm

RetTime [min]	Width [min]	Area [mAu*s]	Height [mAu]	Area [%]
27.139	1.64	918.14	21.34	4.75
28.806	2.78	18413.74	377.00	95.25

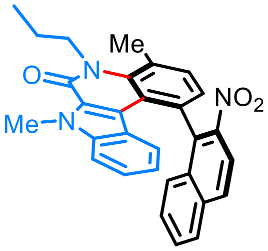

3j

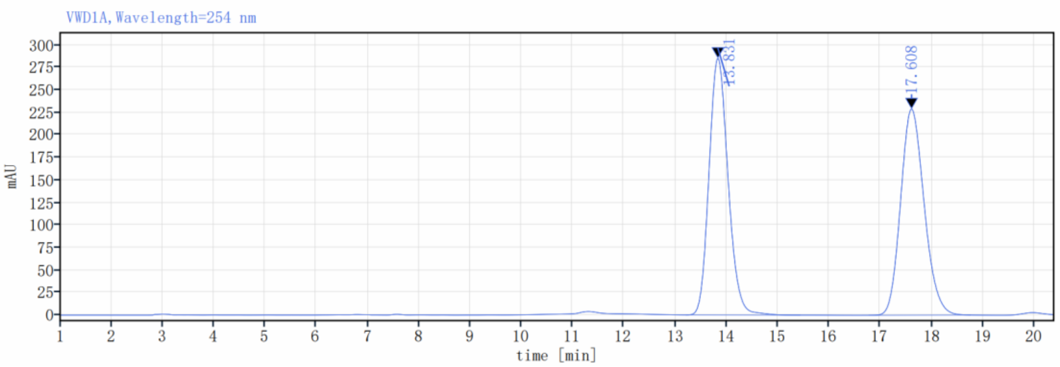

Signal: VWD1A, Wavelength=254 nm

RetTime [min]	Width [min]	Area [mAu*s]	Height [mAu]	Area [%]
13.831	2.23	7375.13	285.37	50.21
17.608	1.99	7313.01	229.20	49.79

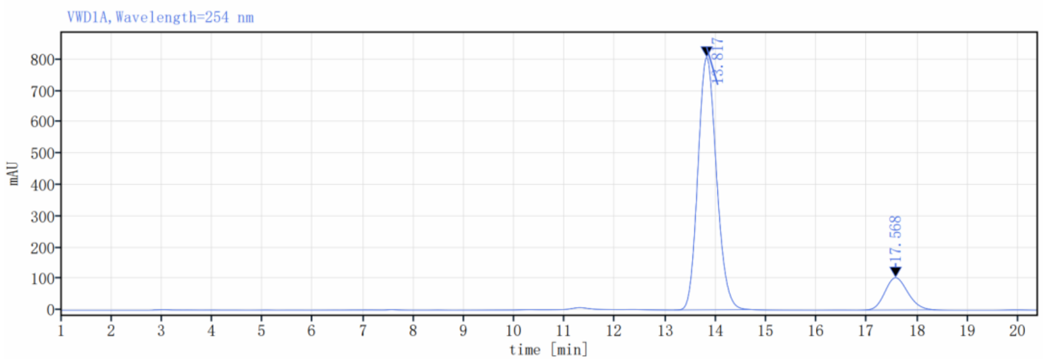

Signal: VWD1A, Wavelength=254 nm

RetTime [min]	Width [min]	Area [mAu*s]	Height [mAu]	Area [%]
13.817	1.54	20653.38	806.55	86.39
17.568	1.54	3252.47	103.14	13.61

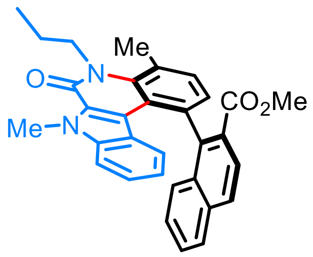

3k

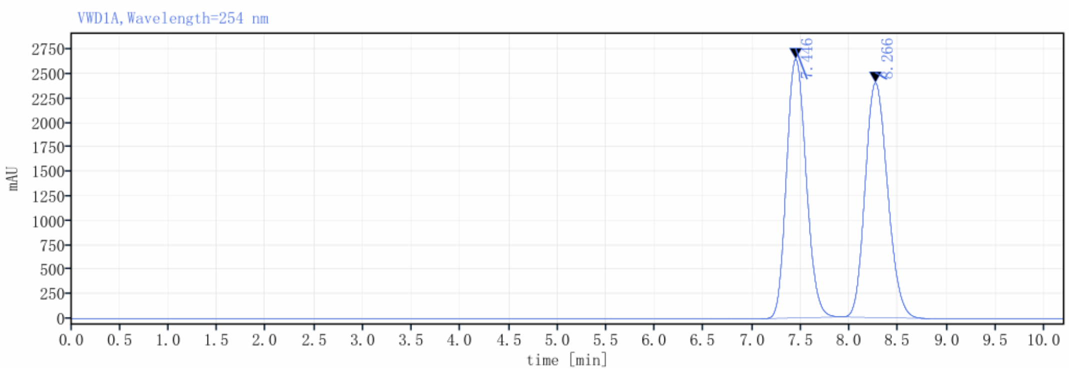

Signal: VWD1A, Wavelength=254 nm

RetTime [min]	Width [min]	Area [mAu*s]	Height [mAu]	Area [%]
7.446	0.81	37137.53	2642.72	49.13
8.266	0.92	38447.14	2396.21	50.87

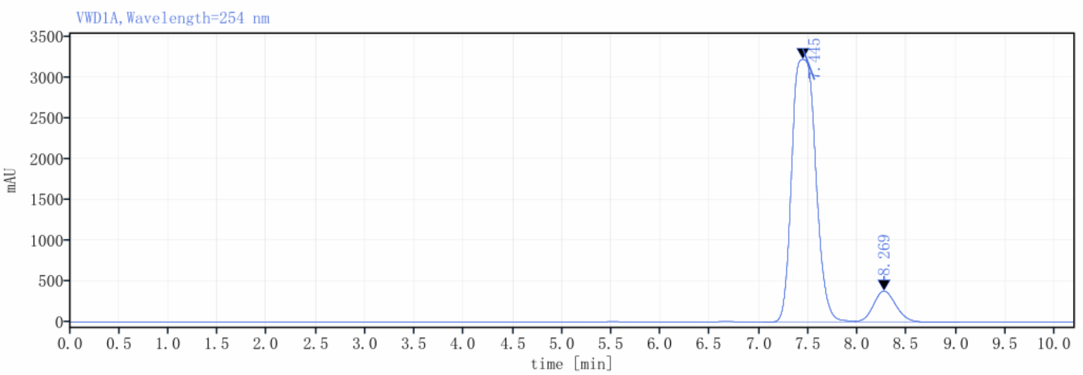

Signal: VWD1A, Wavelength=254 nm

RetTime [min]	Width [min]	Area [mAu*s]	Height [mAu]	Area [%]
7.445	0.94	54699.20	3233.32	89.94
8.269	1.06	6117.56	382.96	10.06

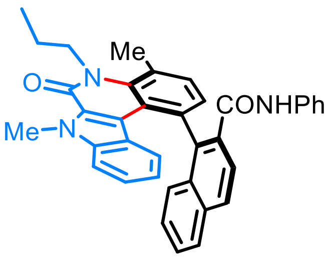

3l

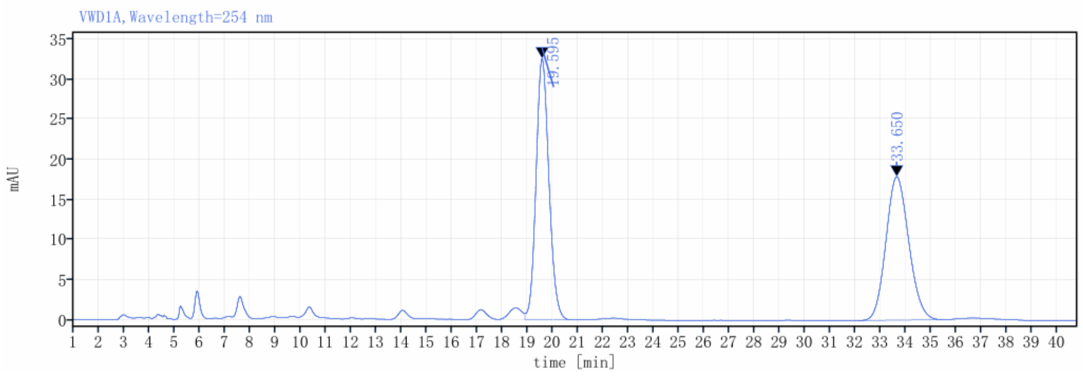

Signal:	VWD1A, Wavelength=254 nm			
RetTime [min]	Width [min]	Area [mAu*s]	Height [mAu]	Area [%]
19.595	1.72	1150.42	32.52	50.52
33.650	3.40	1126.70	17.88	49.48

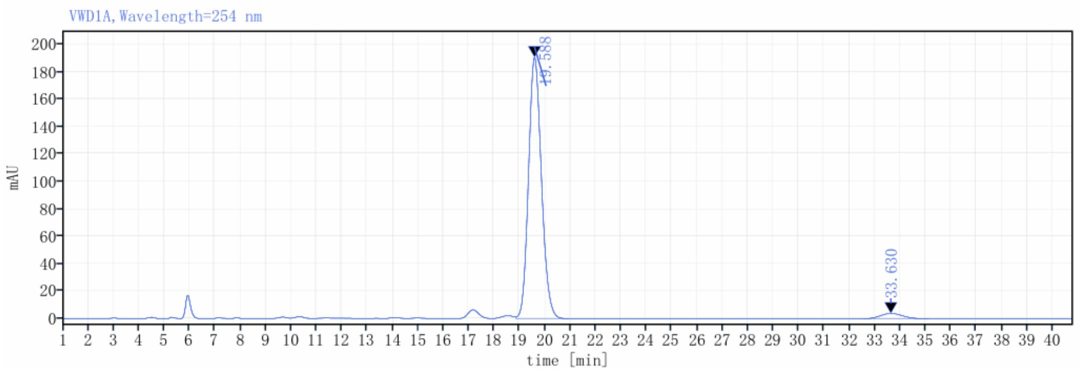

Signal:	VWD1A, Wavelength=254 nm			
RetTime [min]	Width [min]	Area [mAu*s]	Height [mAu]	Area [%]
19.588	2.10	6751.69	190.47	96.56
33.630	2.58	240.55	3.92	3.44

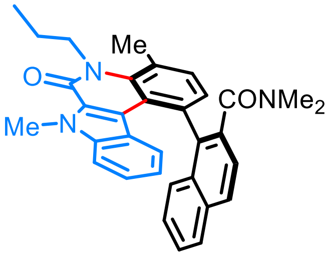

3m

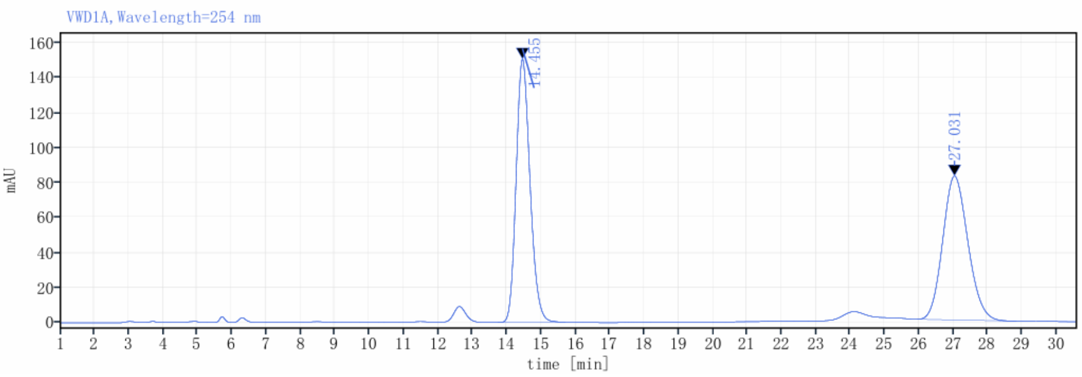

**Signal:**	VWD1A, Wavelength=254 nm			
RetTime [min]	Width [min]	Area [mAu*s]	Height [mAu]	Area [%]
14.455	1.96	4242.73	150.74	50.27
27.031	2.89	4196.71	82.40	49.73

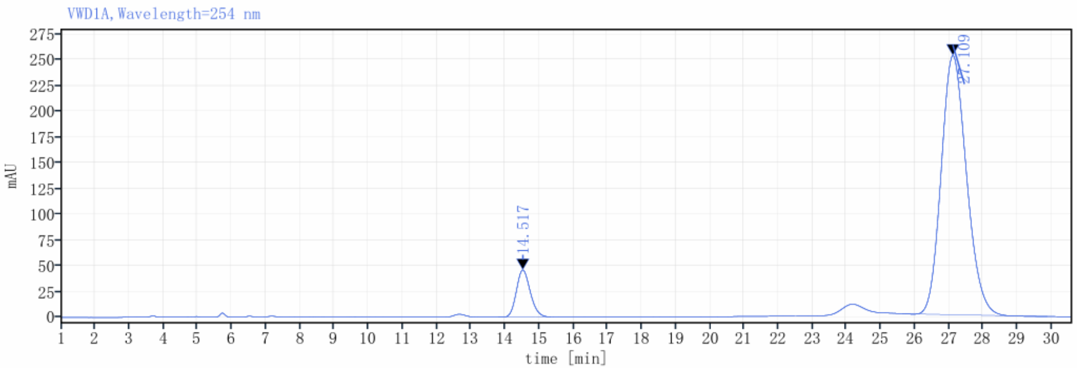

**Signal:**	VWD1A, Wavelength=254 nm			
RetTime [min]	Width [min]	Area [mAu*s]	Height [mAu]	Area [%]
14.517	1.54	1295.26	45.90	9.07
27.109	4.24	12978.77	251.79	90.93

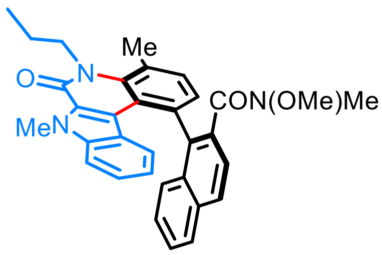

3n

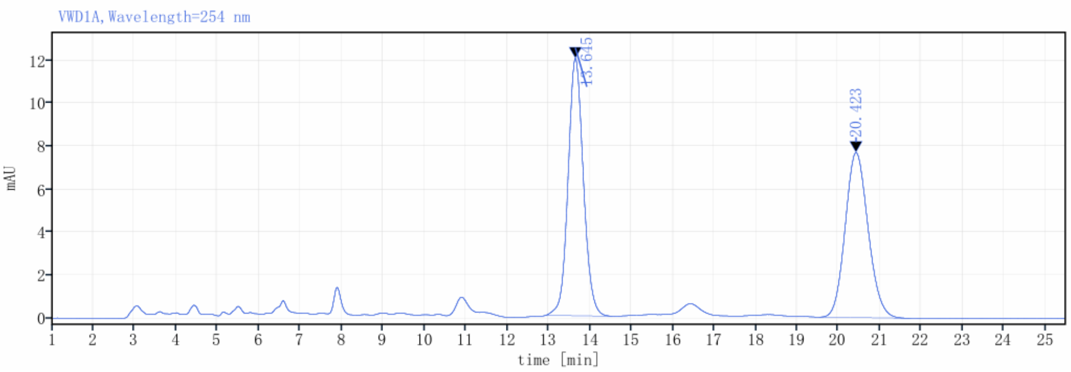

Signal: VWD1A, Wavelength=254 nm

RetTime [min]	Width [min]	Area [mAu*s]	Height [mAu]	Area [%]
13.645	1.62	308.15	11.97	51.09
20.423	2.18	295.06	7.68	48.91

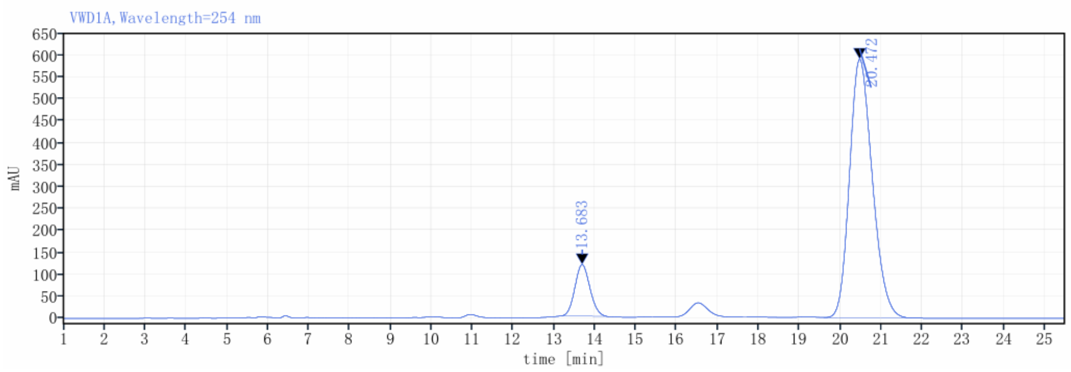

Signal: VWD1A, Wavelength=254 nm

RetTime [min]	Width [min]	Area [mAu*s]	Height [mAu]	Area [%]
13.683	1.11	2998.31	117.53	11.57
20.472	2.40	22925.46	590.41	88.43

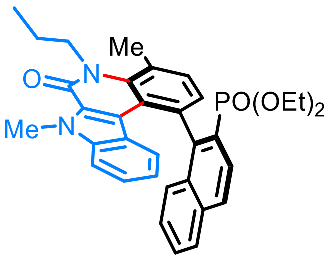

3p

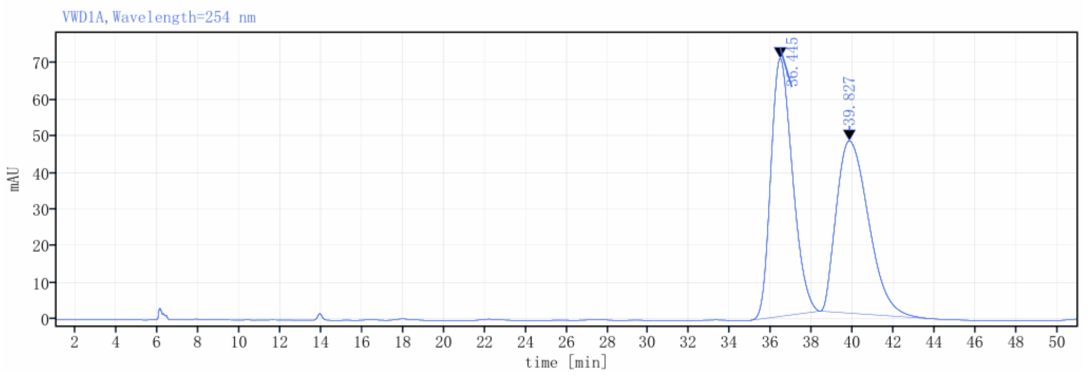

**Signal:**	VWD1A, Wavelength=254 nm			
RetTime [min]	Width [min]	Area [mAu*s]	Height [mAu]	Area [%]
36.445	3.40	5212.29	70.56	50.20
39.827	5.59	5171.21	47.10	49.80

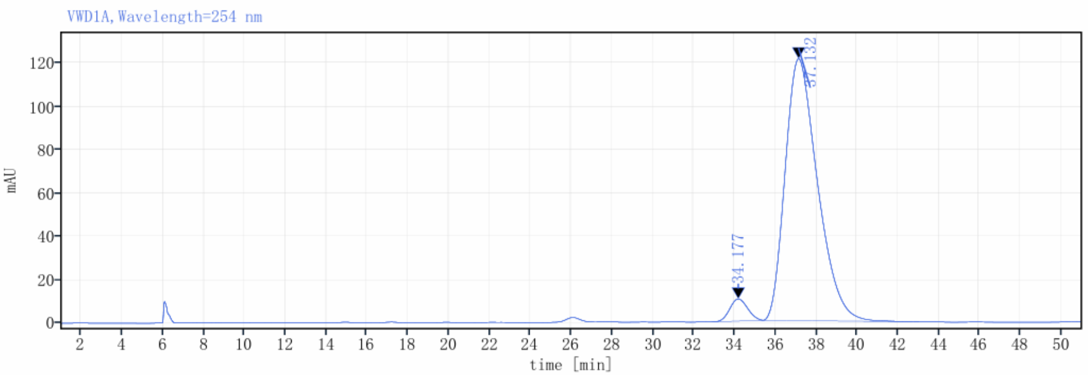

**Signal:**	VWD1A, Wavelength=254 nm			
RetTime [min]	Width [min]	Area [mAu*s]	Height [mAu]	Area [%]
34.177	2.50	618.88	10.23	4.42
37.132	7.63	13371.85	121.04	95.58

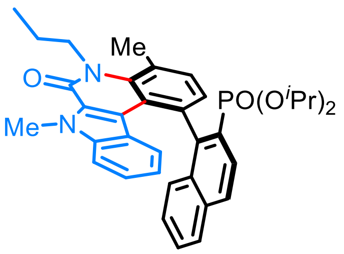

3q

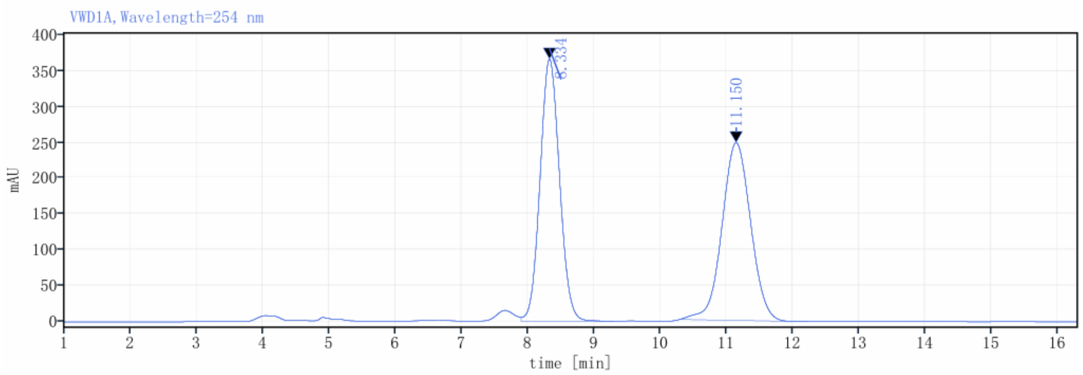

**Signal:**	VWD1A, Wavelength=254 nm			
RetTime [min]	Width [min]	Area [mAu*s]	Height [mAu]	Area [%]
8.334	1.40	7473.05	365.18	50.13
11.150	1.92	7435.38	247.56	49.87

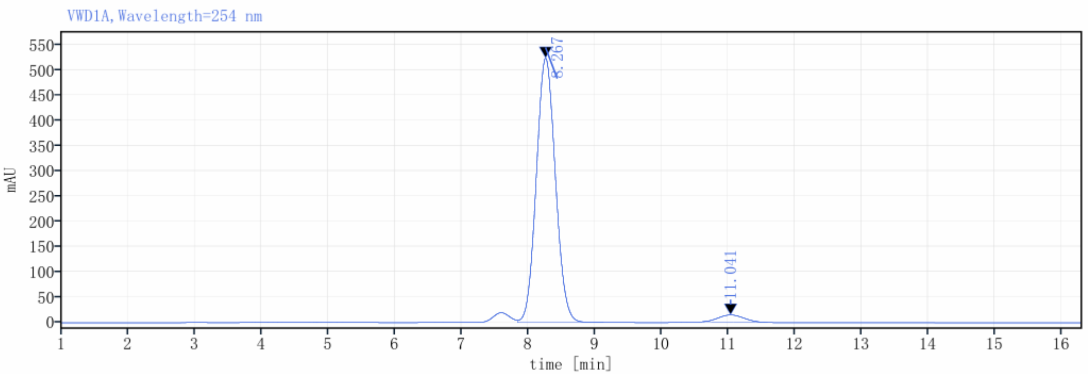

**Signal:**	VWD1A, Wavelength=254 nm			
RetTime [min]	Width [min]	Area [mAu*s]	Height [mAu]	Area [%]
8.267	1.29	10425.06	522.61	96.55
11.041	0.96	372.46	14.00	3.45

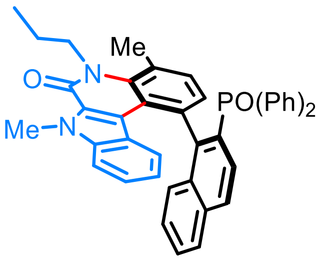

3r

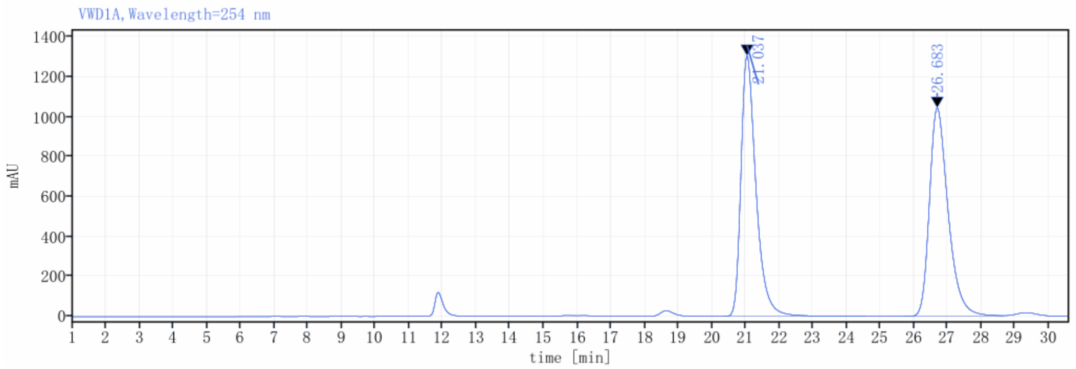

Signal: VWD1A, Wavelength=254 nm

RetTime [min]	Width [min]	Area [mAu*s]	Height [mAu]	Area [%]
21.037	4.23	40552.95	1304.20	50.06
26.683	2.91	40463.13	1043.59	49.94

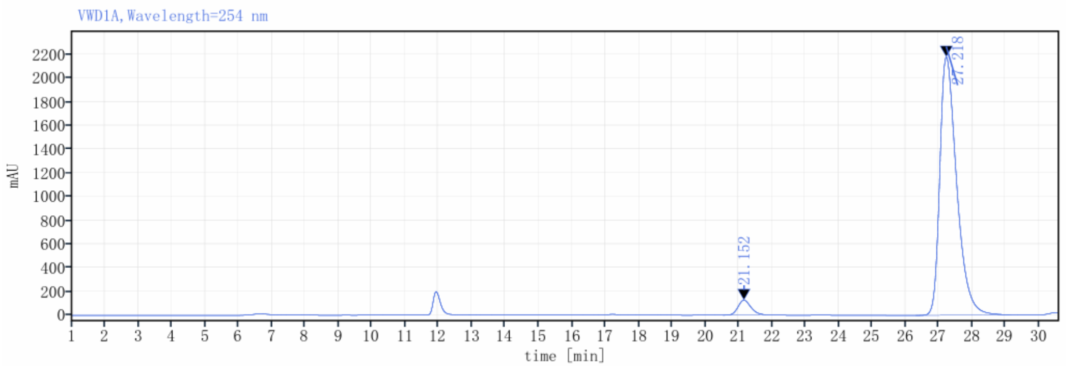

Signal: VWD1A, Wavelength=254 nm

RetTime [min]	Width [min]	Area [mAu*s]	Height [mAu]	Area [%]
21.152	1.19	3215.21	123.64	4.03
27.218	2.82	76587.36	2178.52	95.97

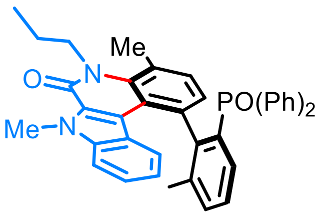

3s

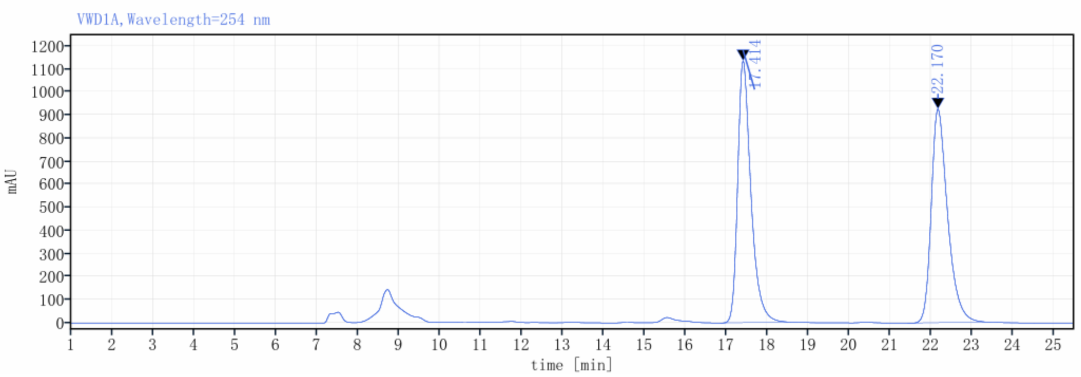

Signal:	VWD1A, Wavelength=254 nm			
RetTime [min]	Width [min]	Area [mAu*s]	Height [mAu]	Area [%]
17.414	1.63	25419.75	1134.41	49.76
22.170	1.88	25662.60	925.79	50.24

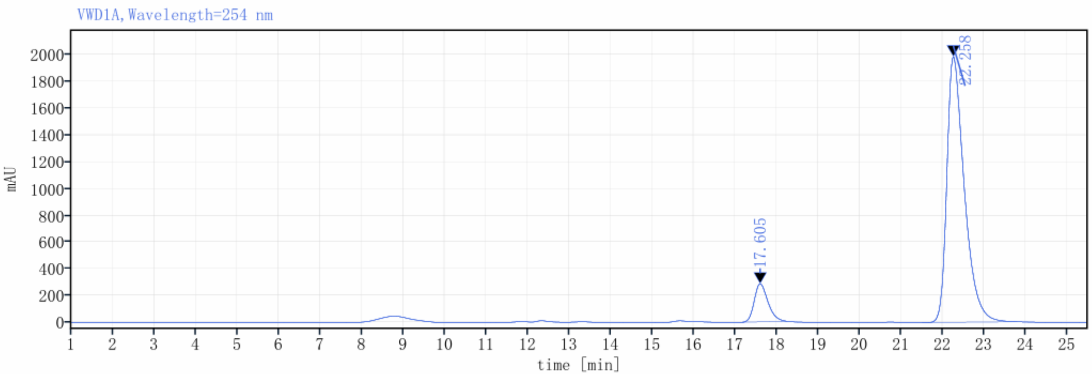

Signal:	VWD1A, Wavelength=254 nm			
RetTime [min]	Width [min]	Area [mAu*s]	Height [mAu]	Area [%]
17.605	1.08	6523.21	285.11	10.11
22.258	2.28	58021.65	1985.75	89.89

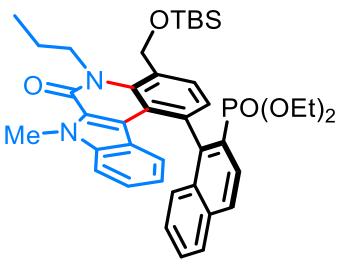

4a

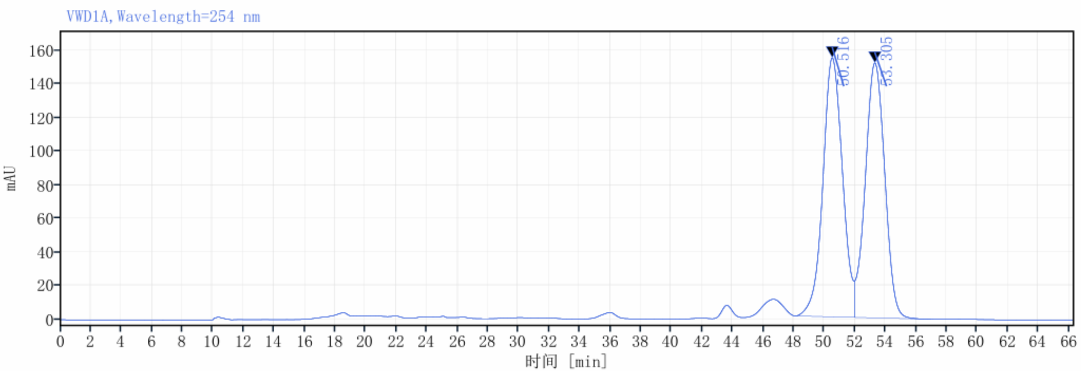

Signal:	VWD1A, Wavelength=254 nm			
**RetTime [min]**	**Width [min]**	**Area [mAu*s]**	**Height [mAu]**	**Area [%]**
50.516	3.95	13771.04	154.09	50.67
53.305	4.95	13407.56	151.62	49.33

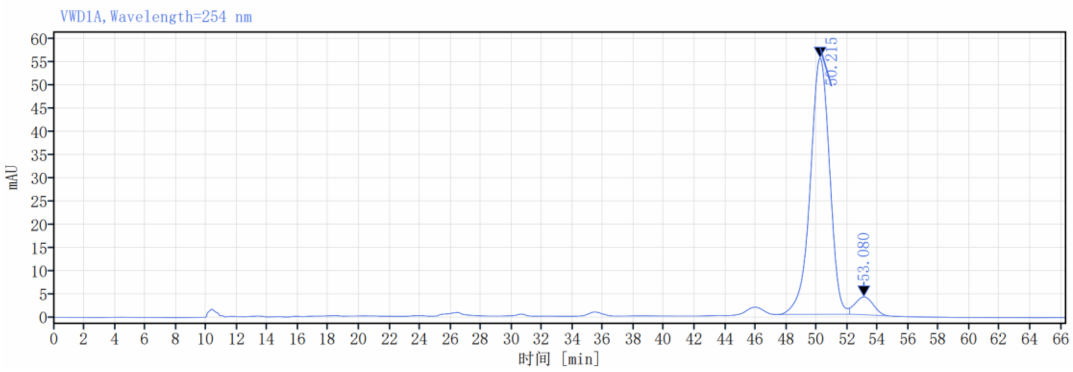

Signal:	VWD1A, Wavelength=254 nm			
**RetTime [min]**	**Width [min]**	**Area [mAu*s]**	**Height [mAu]**	**Area [%]**
50.215	4.96	4887.34	55.19	93.88
53.080	2.50	318.86	3.86	6.12

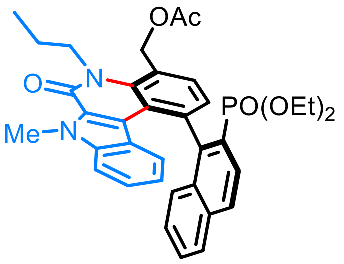

4b

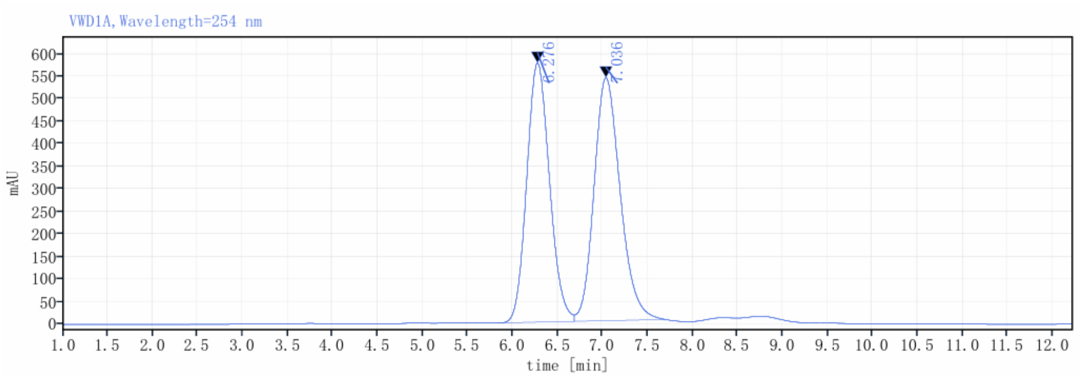

Signal: VWD1A, Wavelength=254 nm

RetTime [min]	Width [min]	Area [mAu*s]	Height [mAu]	Area [%]
6.276	0.85	10148.10	574.99	48.19
7.036	1.01	10909.13	539.31	51.81

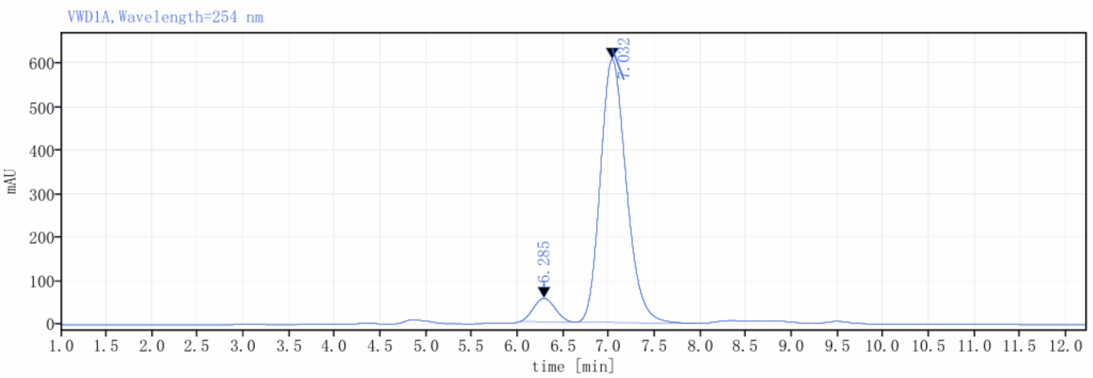

Signal: VWD1A, Wavelength=254 nm

RetTime [min]	Width [min]	Area [mAu*s]	Height [mAu]	Area [%]
6.285	0.58	885.92	53.91	6.90
7.032	1.36	11958.02	604.94	93.10

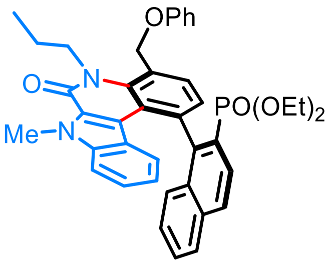

4c

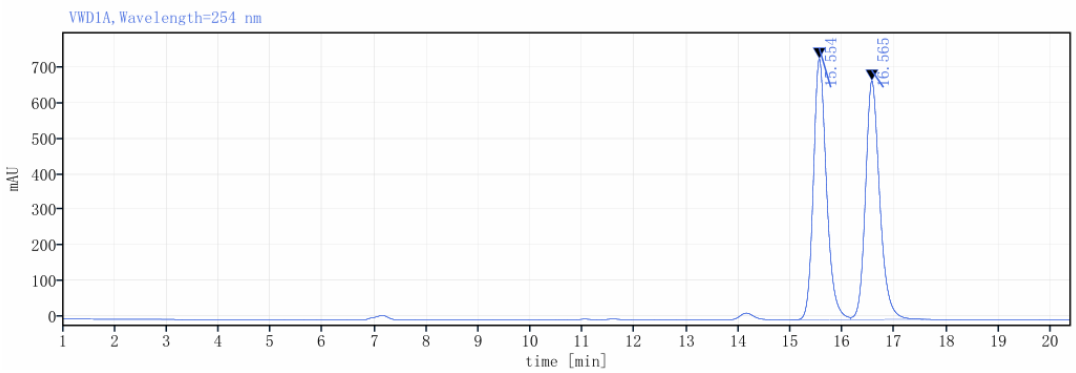

Signal: VWD1A, Wavelength=254 nm

RetTime [min]	Width [min]	Area [mAu*s]	Height [mAu]	Area [%]
15.554	1.15	12874.92	733.42	50.17
16.565	1.40	12788.46	670.95	49.83

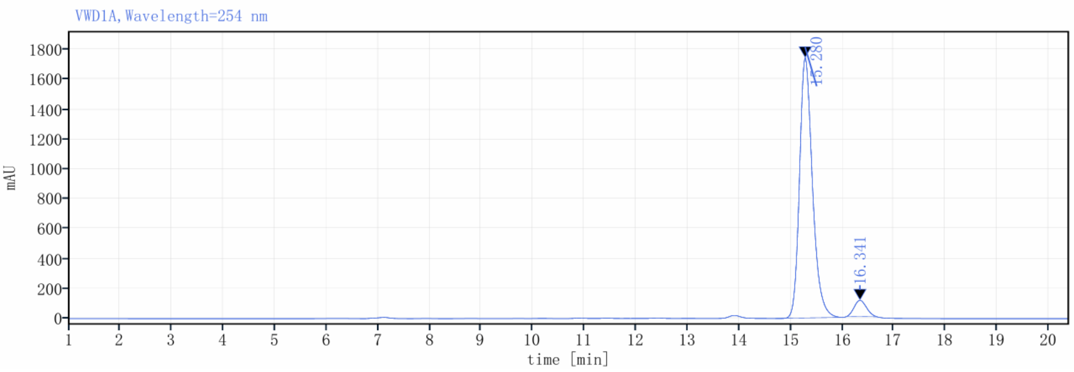

Signal: VWD1A, Wavelength=254 nm

RetTime [min]	Width [min]	Area [mAu*s]	Height [mAu]	Area [%]
15.280	1.32	30377.94	1741.54	94.46
16.341	0.58	1782.99	108.43	5.54

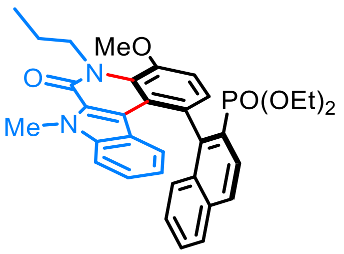

4d

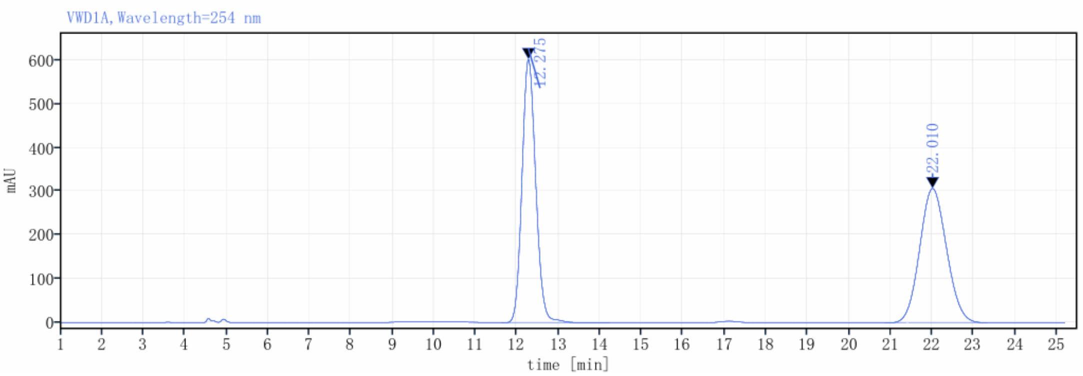

Signal: VWD1A, Wavelength=254 nm

RetTime [min]	Width [min]	Area [mAu*s]	Height [mAu]	Area [%]
12.275	2.61	13847.54	601.59	50.00
22.010	2.47	13846.16	307.01	50.00

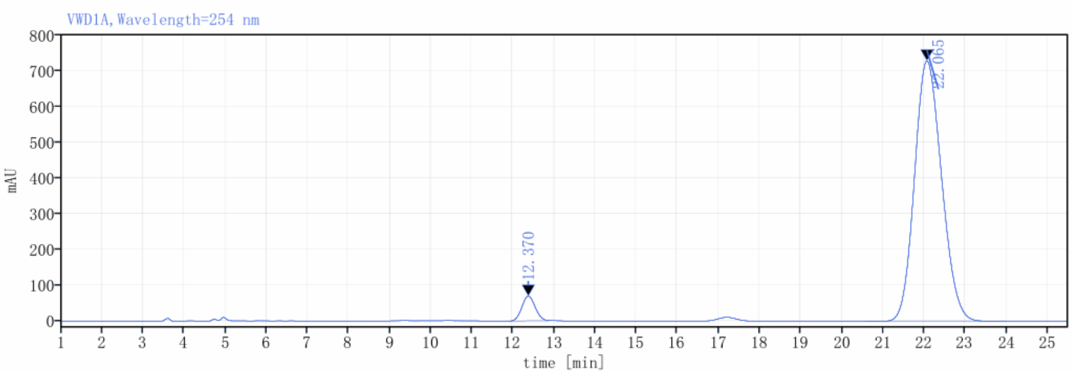

Signal: VWD1A, Wavelength=254 nm

RetTime [min]	Width [min]	Area [mAu*s]	Height [mAu]	Area [%]
12.370	1.06	1564.15	69.79	4.46
22.065	3.43	33469.95	730.03	95.54

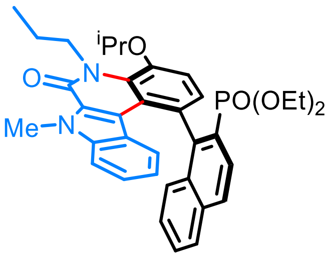

4e

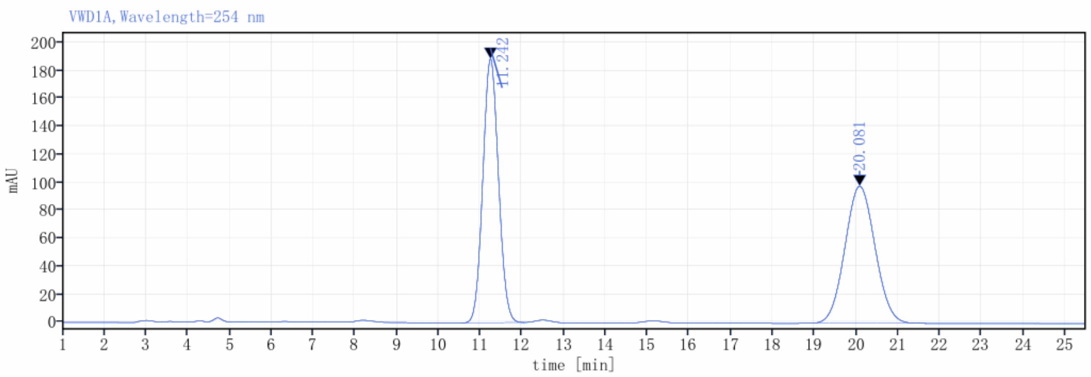

Signal: VWD1A, Wavelength=254 nm

RetTime [min]	Width [min]	Area [mAu*s]	Height [mAu]	Area [%]
11.242	1.51	4907.65	188.68	50.03
20.081	2.64	4901.08	97.88	49.97

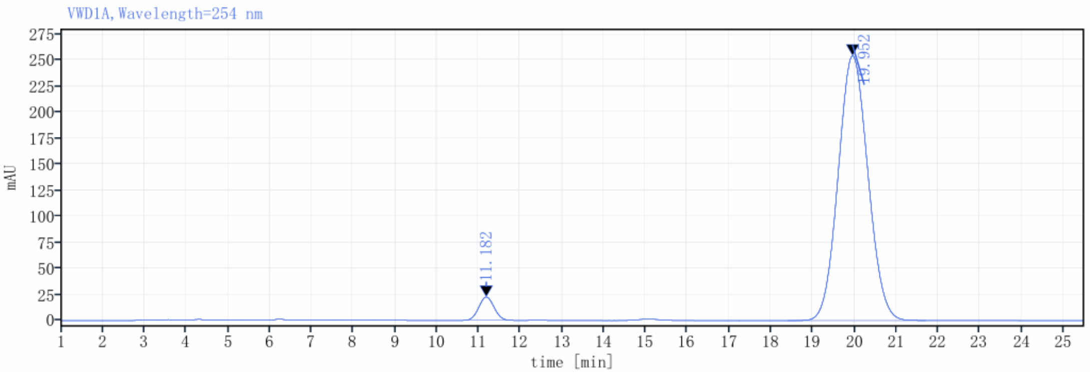

Signal: VWD1A, Wavelength=254 nm

RetTime [min]	Width [min]	Area [mAu*s]	Height [mAu]	Area [%]
11.182	1.14	573.06	22.44	4.35
19.952	3.31	12608.44	254.31	95.65

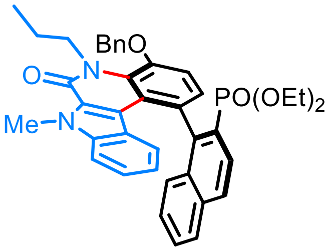

4f

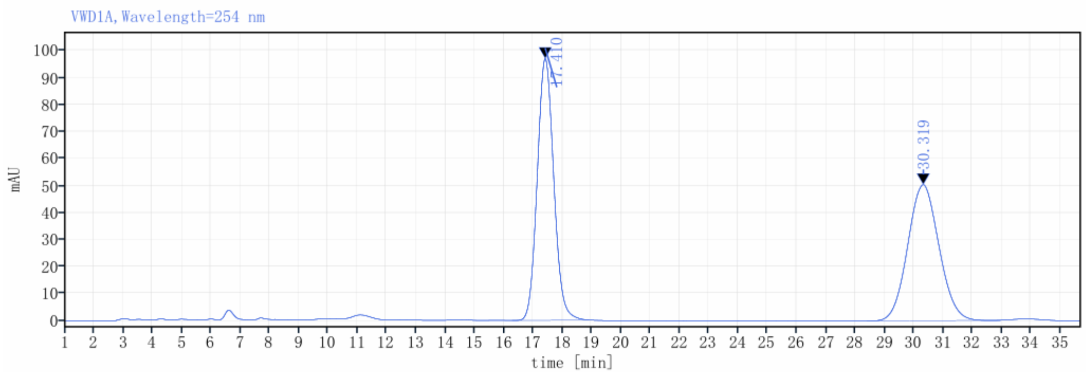

Signal: VWD1A, Wavelength=254 nm

RetTime [min]	Width [min]	Area [mAu*s]	Height [mAu]	Area [%]
17.410	2.63	3810.00	97.05	50.53
30.319	4.54	3730.75	50.44	49.47

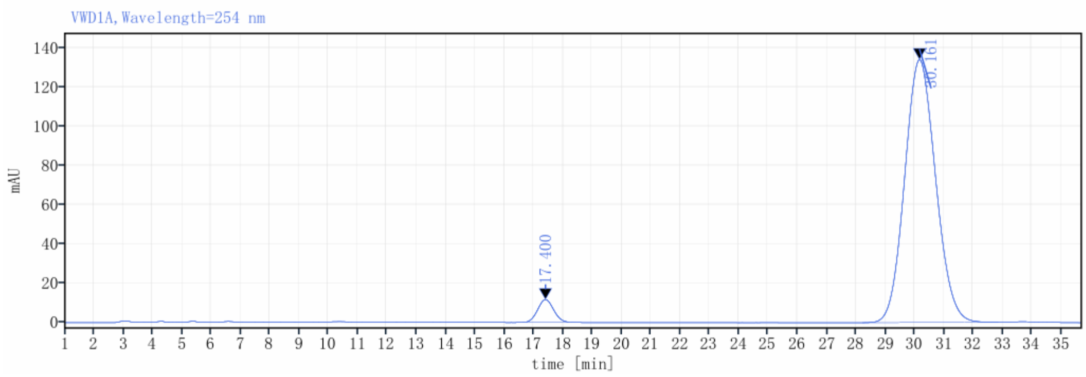

Signal: VWD1A, Wavelength=254 nm

RetTime [min]	Width [min]	Area [mAu*s]	Height [mAu]	Area [%]
17.400	1.41	416.66	11.37	4.07
30.161	4.63	9826.92	133.96	95.93

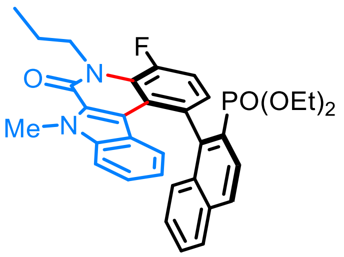

4g

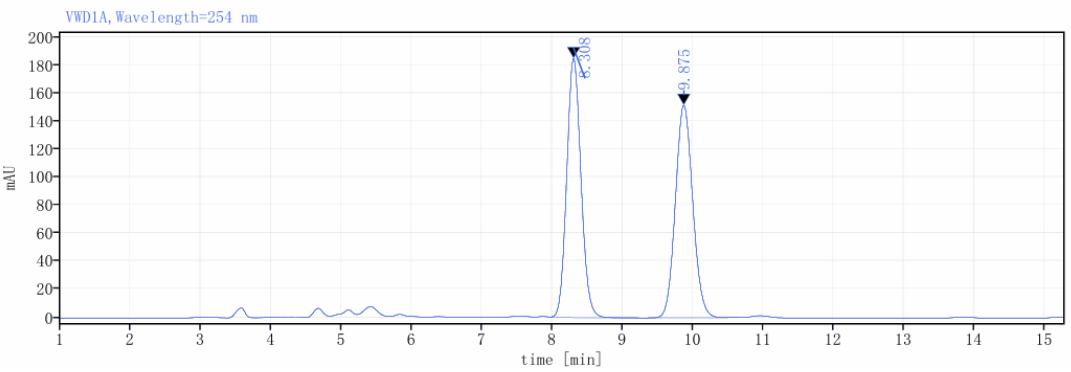

Signal: VWD1A, Wavelength=254 nm

RetTime [min]	Width [min]	Area [mAu*s]	Height [mAu]	Area [%]
8.308	1.26	2611.61	185.27	50.05
9.875	1.30	2606.26	151.90	49.95

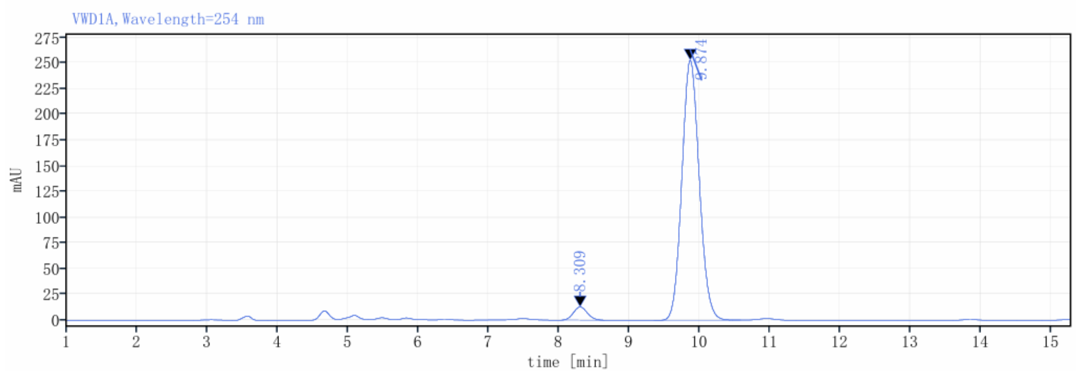

Signal: VWD1A, Wavelength=254 nm

RetTime [min]	Width [min]	Area [mAu*s]	Height [mAu]	Area [%]
8.309	0.60	176.71	12.84	3.91
9.874	1.23	4343.75	252.91	96.09

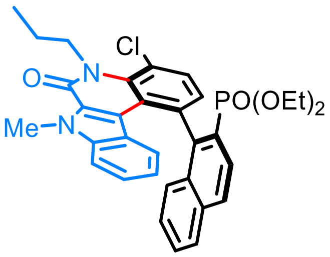

4h

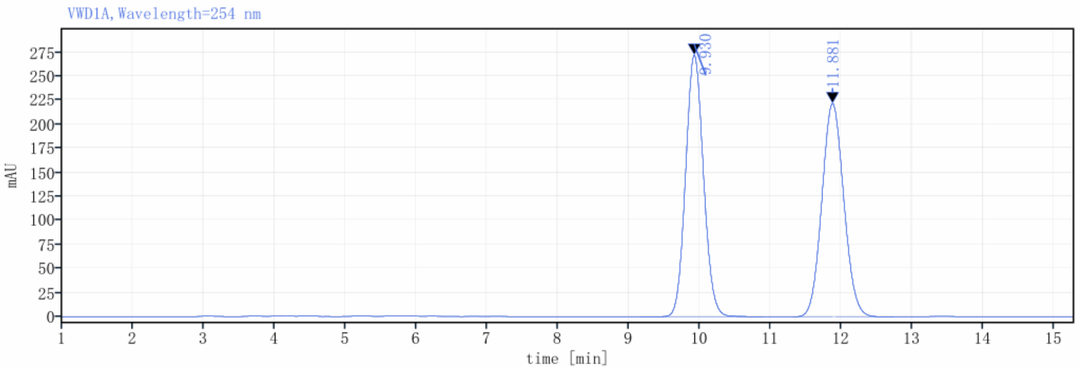

**Signal:**	VWD1A, Wavelength=254 nm			
RetTime [min]	Width [min]	Area [mAu*s]	Height [mAu]	Area [%]
9.930	1.74	4785.10	272.02	50.09
11.881	1.43	4767.46	221.74	49.91

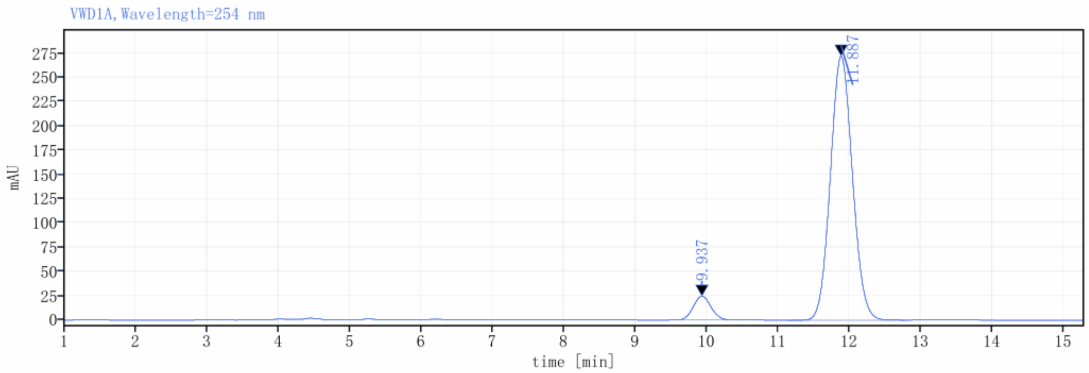

**Signal:**	VWD1A, Wavelength=254 nm			
RetTime [min]	Width [min]	Area [mAu*s]	Height [mAu]	Area [%]
9.937	0.65	404.76	24.08	6.47
11.887	1.62	5850.60	272.34	93.53

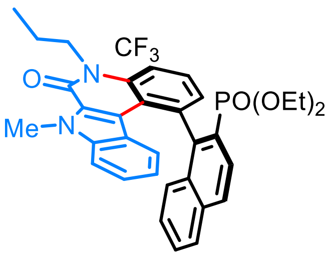

4i

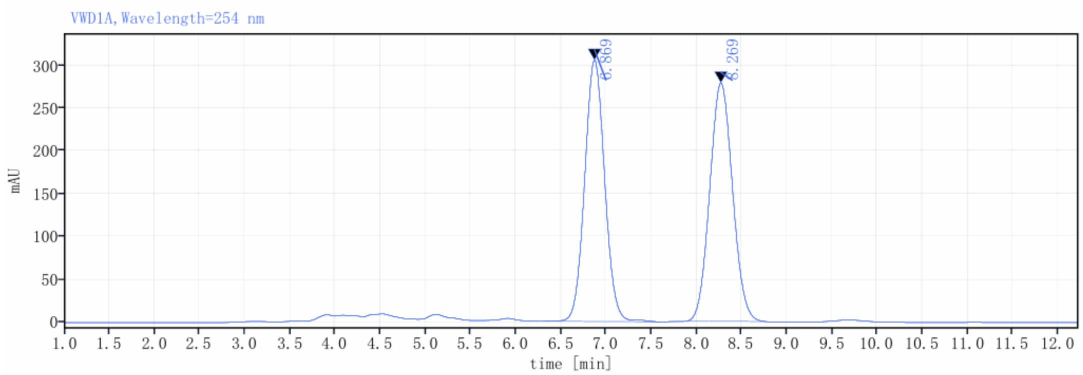

Signal: VWD1A, Wavelength=254 nm

RetTime [min]	Width [min]	Area [mAu*s]	Height [mAu]	Area [%]
6.869	1.26	4696.49	304.72	49.06
8.269	0.86	4876.23	277.86	50.94

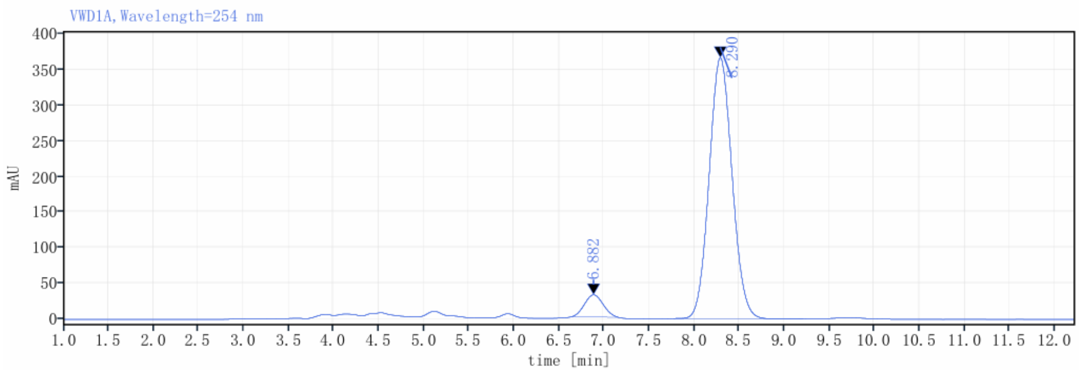

Signal: VWD1A, Wavelength=254 nm

RetTime [min]	Width [min]	Area [mAu*s]	Height [mAu]	Area [%]
6.882	0.51	444.81	30.94	6.44
8.290	1.44	6464.33	365.23	93.56

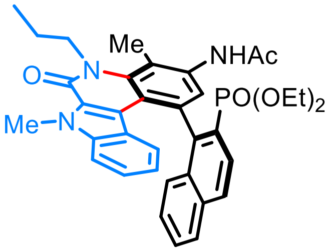

4j

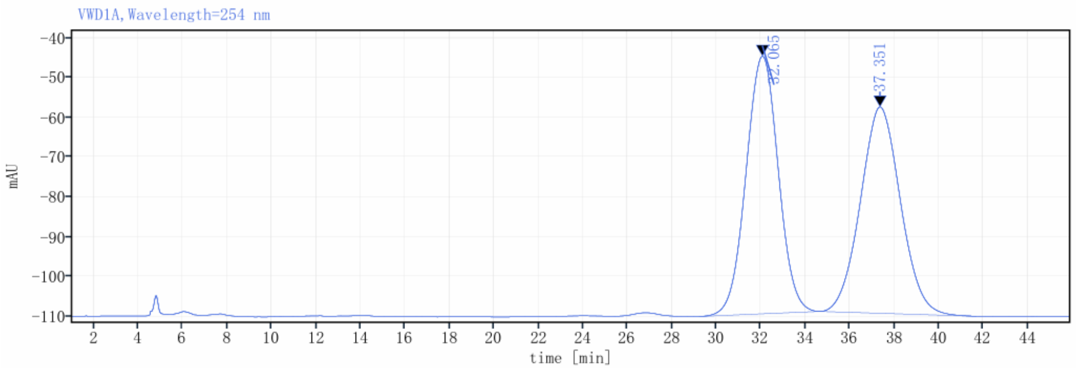

Signal: VWD1A, Wavelength=254 nm

RetTime [min]	Width [min]	Area [mAu*s]	Height [mAu]	Area [%]
32.065	5.40	6535.21	64.74	50.19
37.351	7.05	6484.53	51.87	49.81

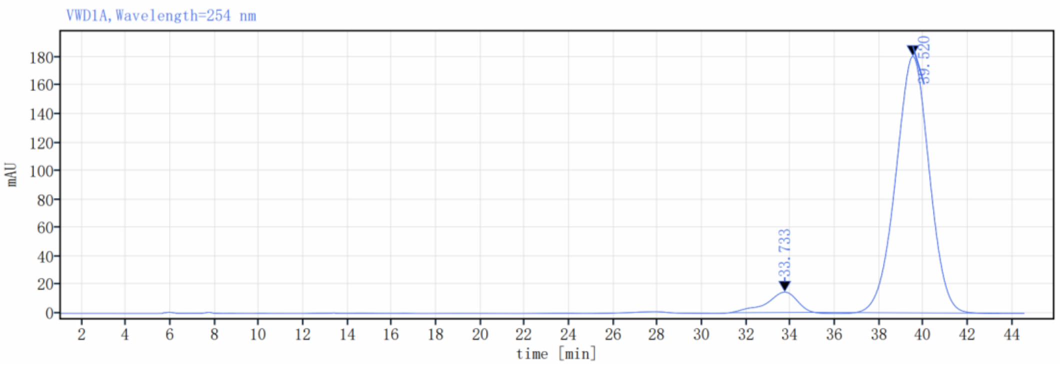

Signal: VWD1A, Wavelength=254 nm

RetTime [min]	Width [min]	Area [mAu*s]	Height [mAu]	Area [%]
33.733	3.80	1378.31	14.31	6.81
39.520	8.39	18870.31	180.15	93.19

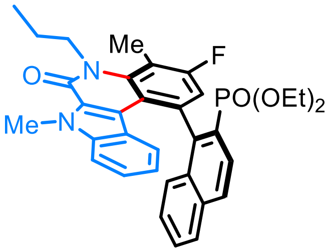

4k

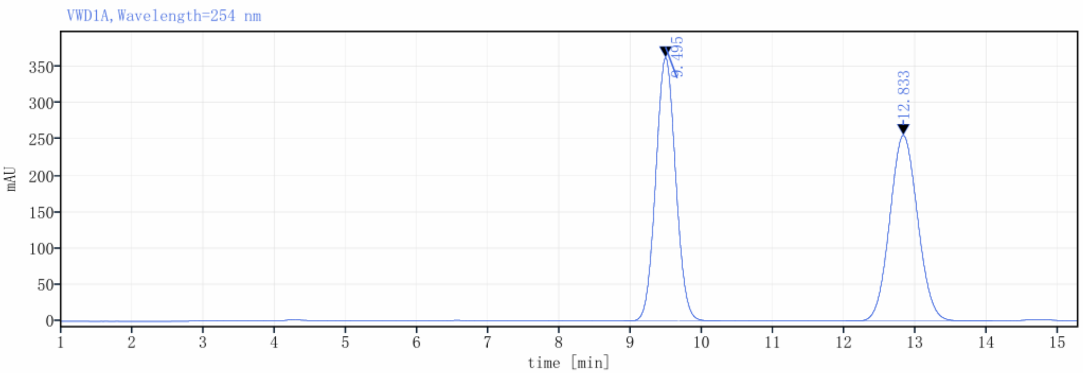

**Signal:**	VWD1A, Wavelength=254 nm			
RetTime [min]	Width [min]	Area [mAu*s]	Height [mAu]	Area [%]
9.495	1.35	7038.14	362.05	50.32
12.833	2.05	6948.15	255.33	49.68

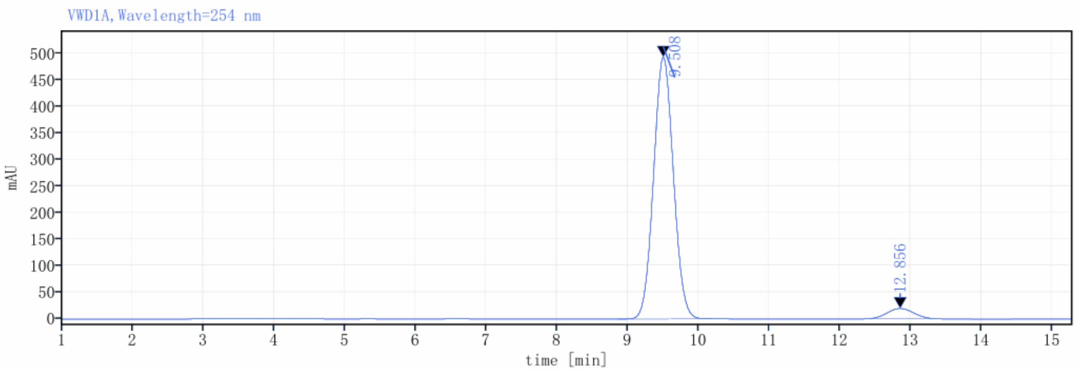

**Signal:**	VWD1A, Wavelength=254 nm			
RetTime [min]	Width [min]	Area [mAu*s]	Height [mAu]	Area [%]
9.508	1.34	9614.41	493.54	95.20
12.856	0.89	485.14	19.10	4.80

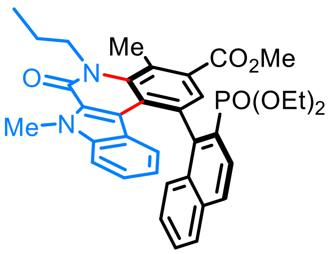

4l

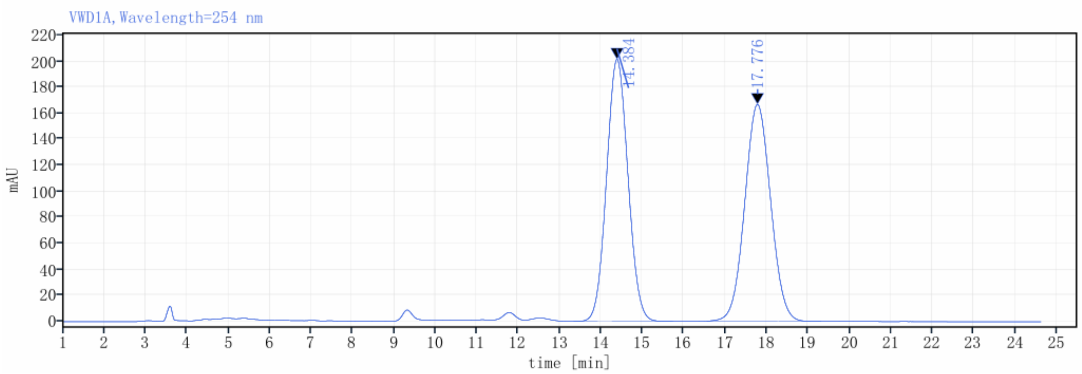

Signal:	VWD1A, Wavelength=254 nm			
RetTime [min]	Width [min]	Area [mAu*s]	Height [mAu]	Area [%]
14.384	2.39	7262.34	201.55	50.02
17.776	2.76	7257.44	166.82	49.98

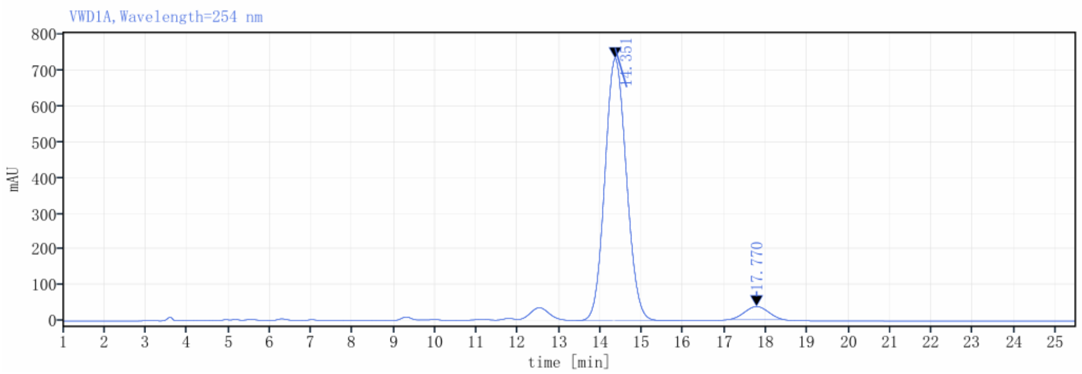

Signal:	VWD1A, Wavelength=254 nm			
RetTime [min]	Width [min]	Area [mAu*s]	Height [mAu]	Area [%]
14.351	2.51	26588.51	733.53	95.02
17.770	1.29	1394.96	35.88	4.98

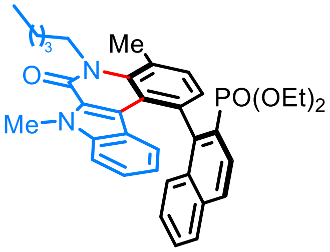

5a

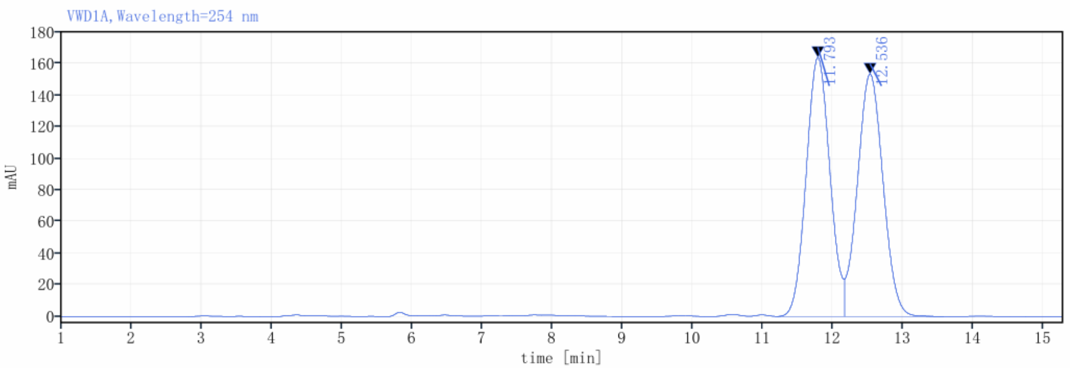

Signal: VWD1A, Wavelength=254 nm

RetTime [min]	Width [min]	Area [mAu*s]	Height [mAu]	Area [%]
11.793	0.97	3857.50	163.28	49.86
12.536	1.41	3879.90	152.88	50.14

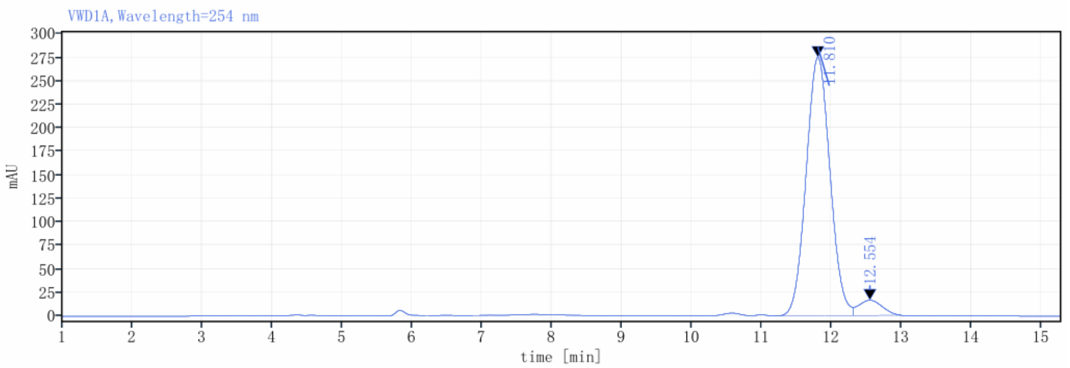

Signal: VWD1A, Wavelength=254 nm

RetTime [min]	Width [min]	Area [mAu*s]	Height [mAu]	Area [%]
11.810	1.13	6532.78	274.81	94.52
12.554	0.68	378.88	16.11	5.48

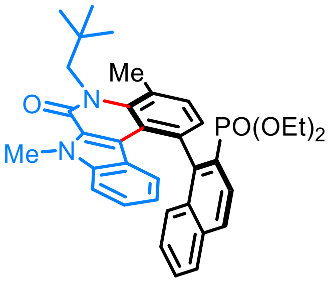

5b

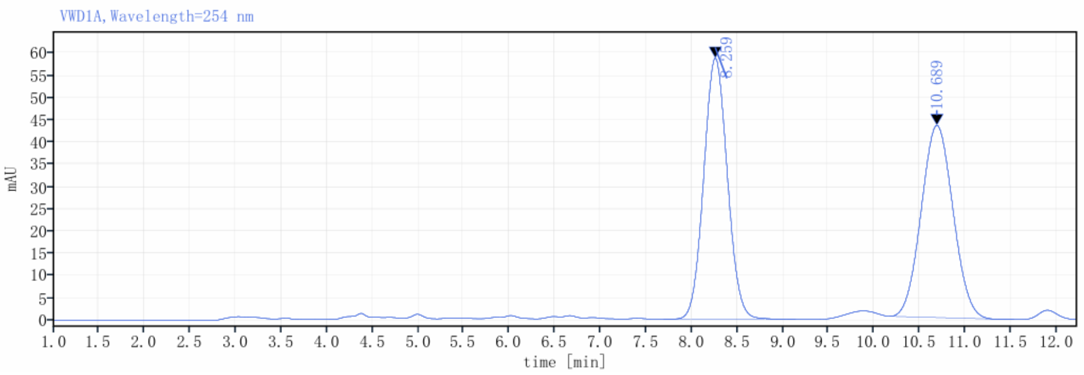

Signal: VWD1A, Wavelength=254 nm

RetTime [min]	Width [min]	Area [mAu*s]	Height [mAu]	Area [%]
8.259	1.45	1060.65	58.78	50.28
10.689	1.36	1048.91	43.26	49.72

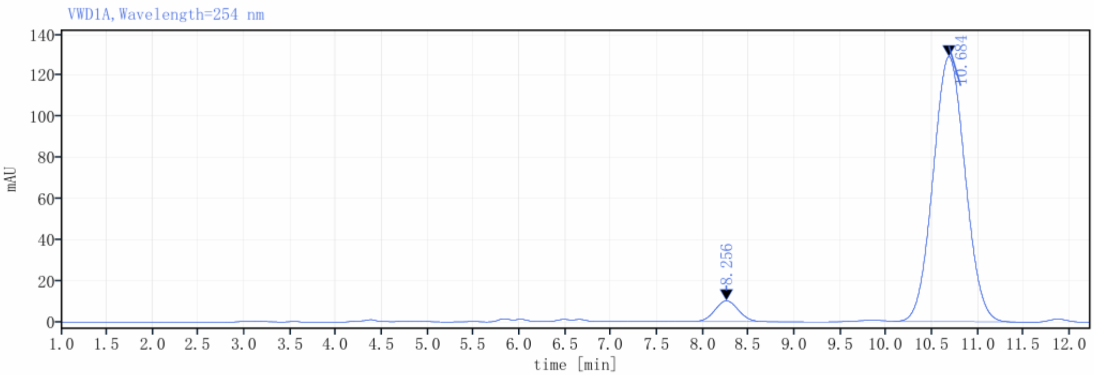

Signal: VWD1A, Wavelength=254 nm

RetTime [min]	Width [min]	Area [mAu*s]	Height [mAu]	Area [%]
8.256	0.64	168.06	9.84	5.09
10.684	1.58	3133.35	128.88	94.91

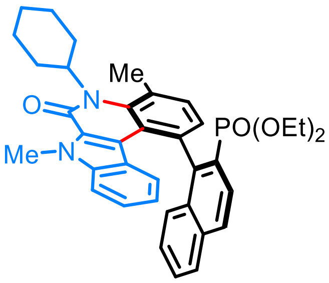

5c

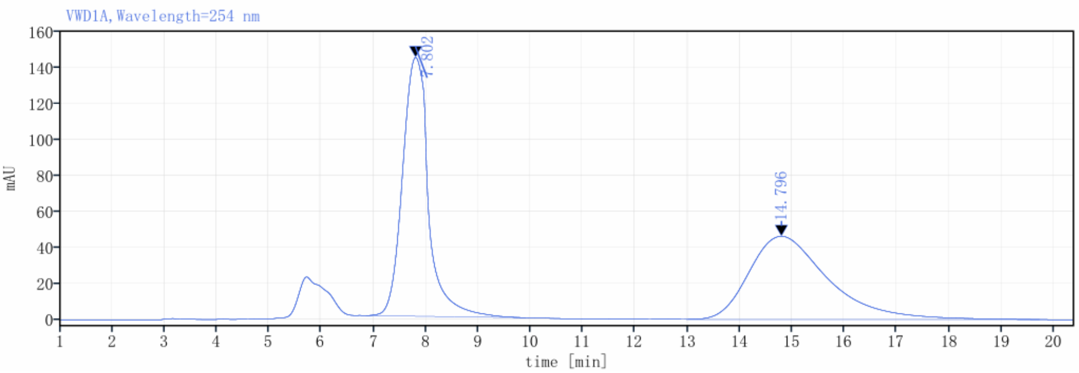

Signal: VWD1A, Wavelength=254 nm

RetTime [min]	Width [min]	Area [mAu*s]	Height [mAu]	Area [%]
7.802	3.04	4974.10	143.82	50.66
14.796	6.79	4844.97	46.23	49.34

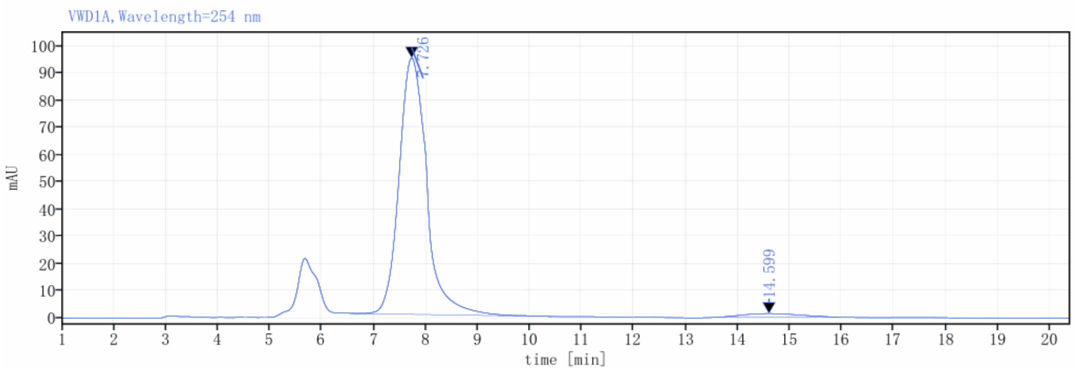

Signal: VWD1A, Wavelength=254 nm

RetTime [min]	Width [min]	Area [mAu*s]	Height [mAu]	Area [%]
7.726	3.85	3494.47	94.43	97.16
14.599	2.33	102.03	1.34	2.84

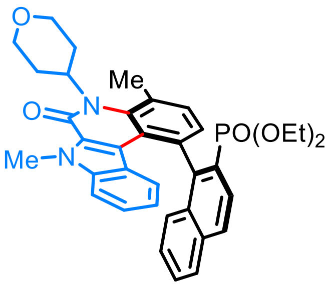

5d

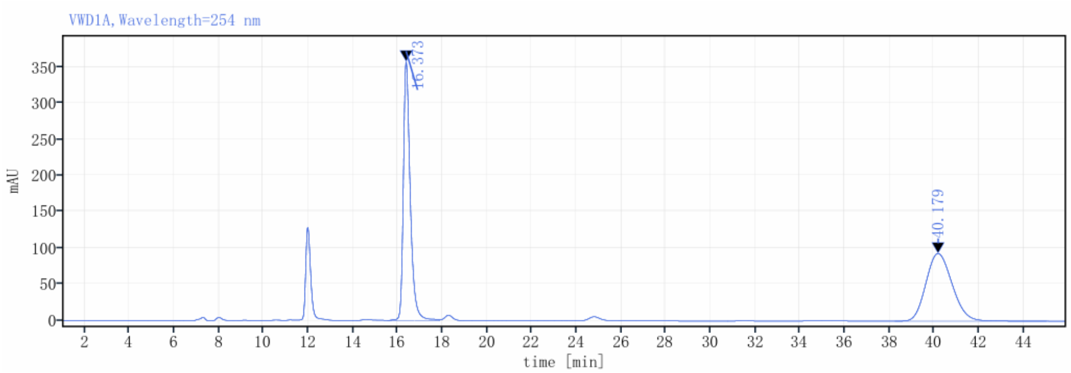

Signal:	VWD1A, Wavelength=254 nm			
RetTime [min]	Width [min]	Area [mAu*s]	Height [mAu]	Area [%]
16.373	2.34	7669.30	357.15	50.20
40.179	6.78	7607.66	93.40	49.80

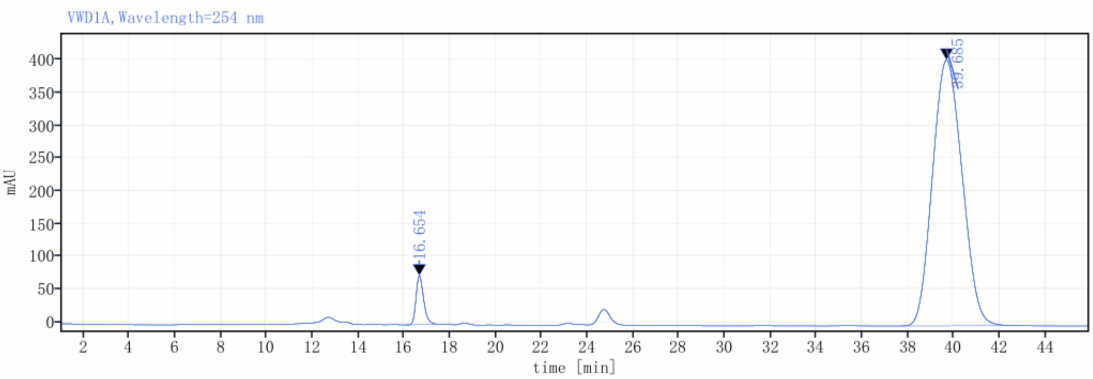

Signal:	VWD1A, Wavelength=254 nm			
RetTime [min]	Width [min]	Area [mAu*s]	Height [mAu]	Area [%]
16.654	1.36	1713.93	74.55	4.48
39.685	5.12	36527.47	405.58	95.52

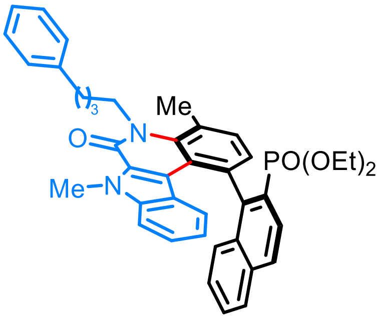

5e

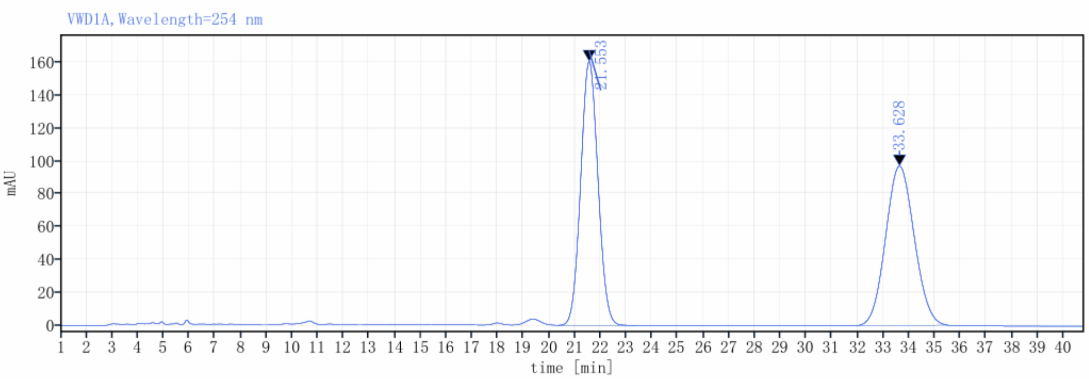

Signal: VWD1A, Wavelength=254 nm

RetTime [min]	Width [min]	Area [mAu*s]	Height [mAu]	Area [%]
21.553	3.04	7540.20	161.20	50.00
33.628	3.56	7540.71	97.37	50.00

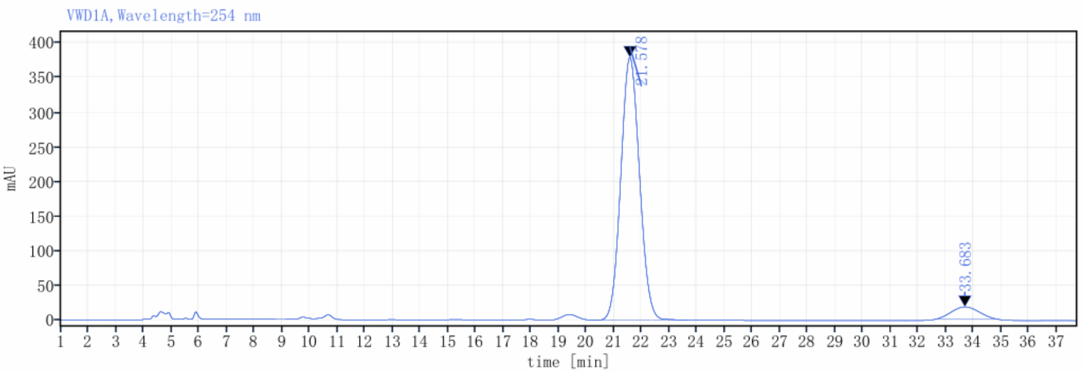

Signal: VWD1A, Wavelength=254 nm

RetTime [min]	Width [min]	Area [mAu*s]	Height [mAu]	Area [%]
21.578	3.54	17818.73	379.46	93.41
33.683	2.30	1258.07	17.95	6.59

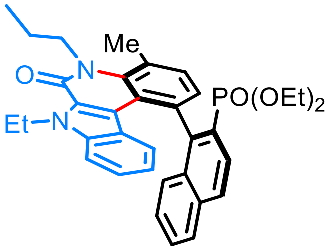

5f

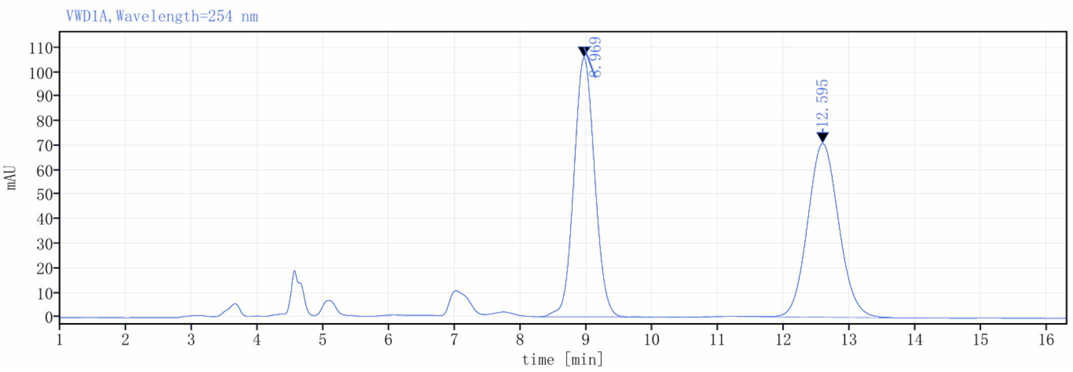

Signal:	VWD1A, Wavelength=254 nm			
RetTime [min]	Width [min]	Area [mAu*s]	Height [mAu]	Area [%]
8.969	1.36	2321.91	105.71	49.92
12.595	2.63	2329.33	70.90	50.08

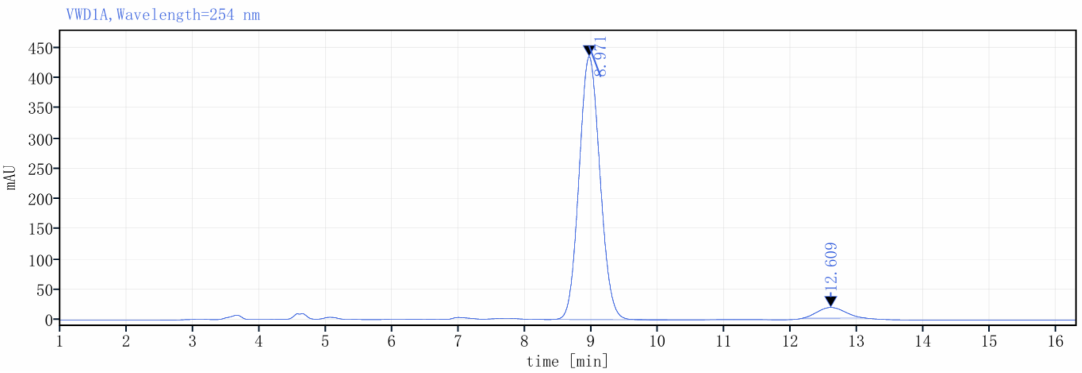

Signal:	VWD1A, Wavelength=254 nm			
RetTime [min]	Width [min]	Area [mAu*s]	Height [mAu]	Area [%]
8.971	1.93	9524.52	435.66	94.92
12.609	0.95	509.50	17.73	5.08

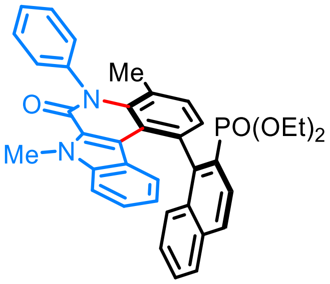

5g

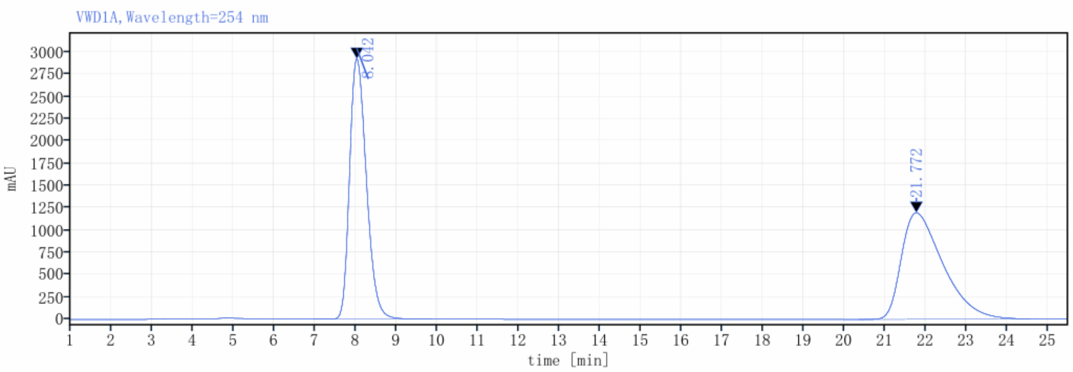

Signal: VWD1A,Wavelength=254 nm

RetTime [min]	Width [min]	Area [mAu*s]	Height [mAu]	Area [%]
8.042	2.90	83969.36	2920.62	49.81
21.772	4.21	84613.82	1193.82	50.19

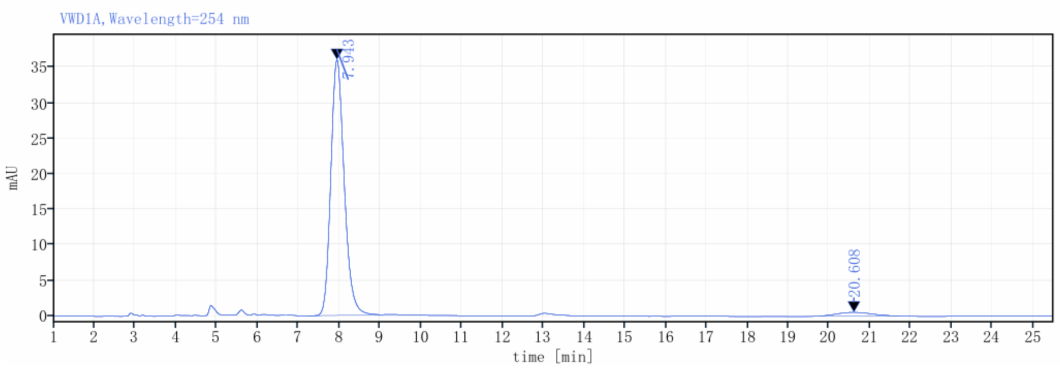

Signal: VWD1A,Wavelength=254 nm

RetTime [min]	Width [min]	Area [mAu*s]	Height [mAu]	Area [%]
7.943	1.79	821.31	35.97	96.45
20.608	1.92	30.26	0.51	3.55

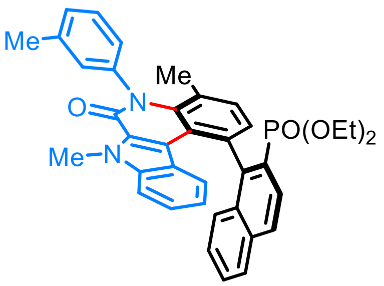

5h

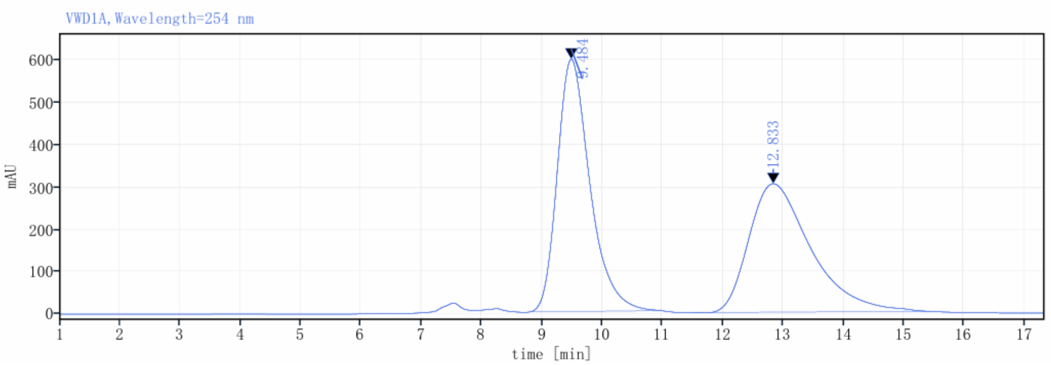

Signal: VWD1A, Wavelength=254 nm

RetTime [min]	Width [min]	Area [mAu*s]	Height [mAu]	Area [%]
9.484	2.17	22499.66	597.73	50.98
12.833	3.94	21635.43	304.22	49.02

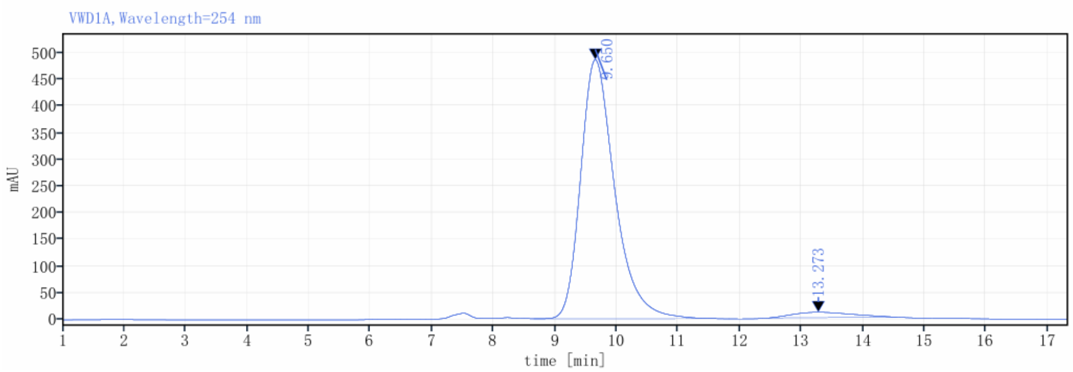

Signal: VWD1A, Wavelength=254 nm

RetTime [min]	Width [min]	Area [mAu*s]	Height [mAu]	Area [%]
9.650	2.96	18674.94	485.04	96.44
13.273	2.09	689.80	10.13	3.56

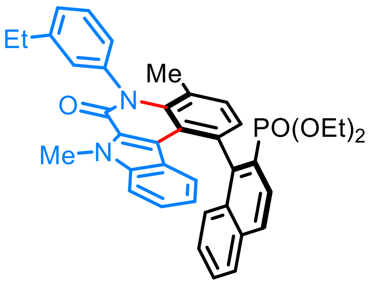

5i

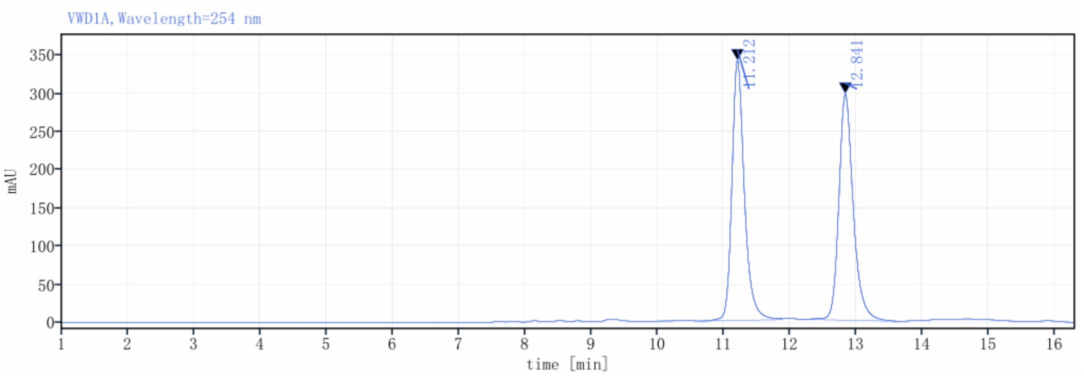

**Signal:**	VWD1A, Wavelength=254 nm			
RetTime [min]	Width [min]	Area [mAu*s]	Height [mAu]	Area [%]
11.212	1.20	4472.37	341.14	49.87
12.841	1.48	4496.17	297.11	50.13

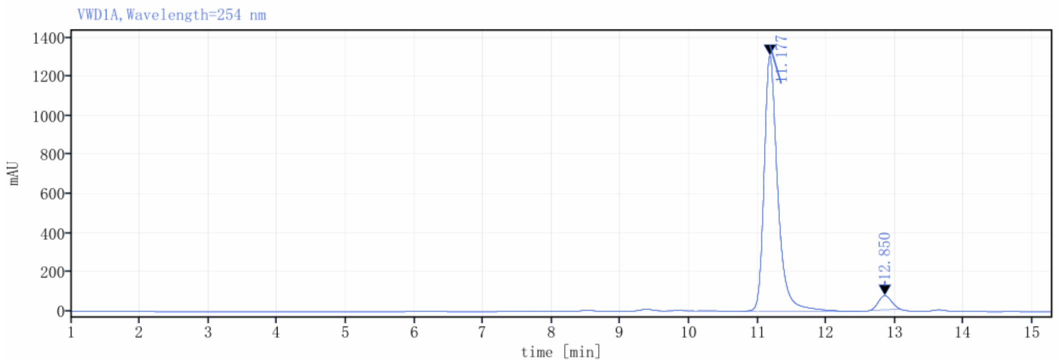

**Signal:**	VWD1A, Wavelength=254 nm			
RetTime [min]	Width [min]	Area [mAu*s]	Height [mAu]	Area [%]
11.177	1.75	17950.79	1305.63	95.00
12.850	0.44	945.57	72.11	5.00

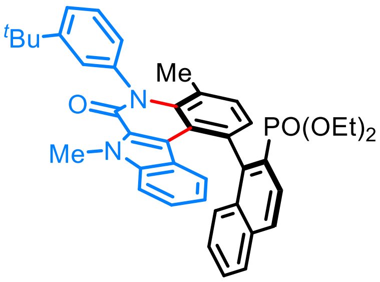

5j

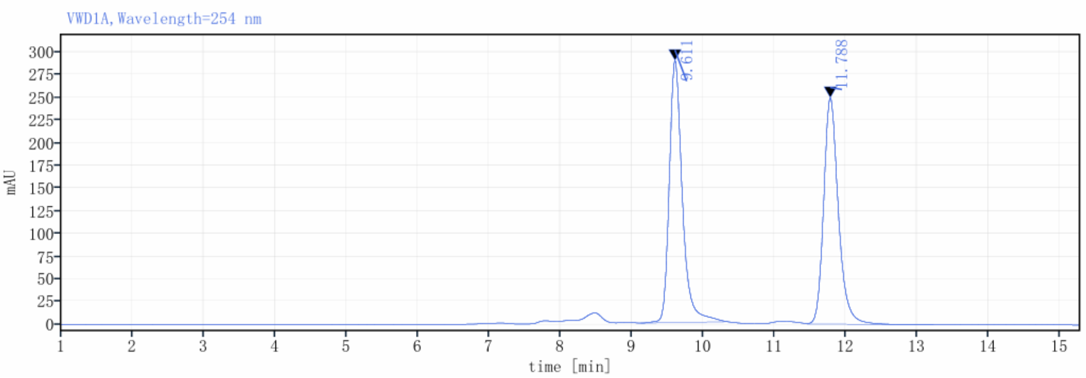

Signal: VWD1A, Wavelength=254 nm

RetTime [min]	Width [min]	Area [mAu*s]	Height [mAu]	Area [%]
9.611	1.21	3673.89	288.61	50.67
11.788	1.77	3577.41	249.23	49.33

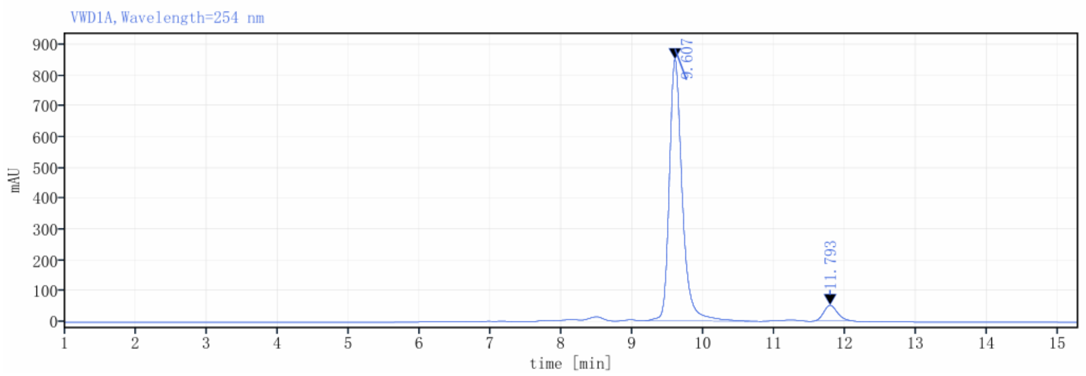

Signal: VWD1A, Wavelength=254 nm

RetTime [min]	Width [min]	Area [mAu*s]	Height [mAu]	Area [%]
9.607	1.46	10768.11	848.87	94.55
11.793	0.47	621.23	48.78	5.45

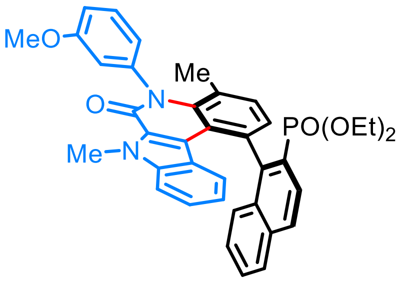

5k

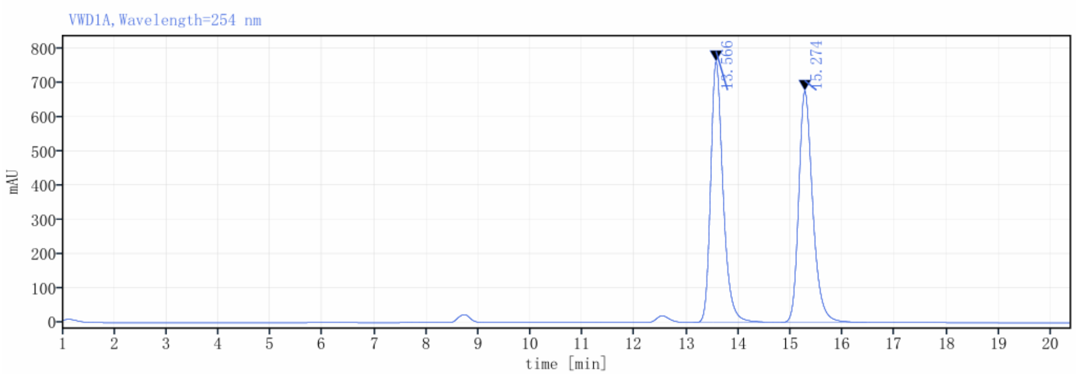

Signal: VWD1A, Wavelength=254 nm

RetTime [min]	Width [min]	Area [mAu*s]	Height [mAu]	Area [%]
13.566	1.37	12641.02	763.18	50.05
15.274	1.65	12616.25	677.15	49.95

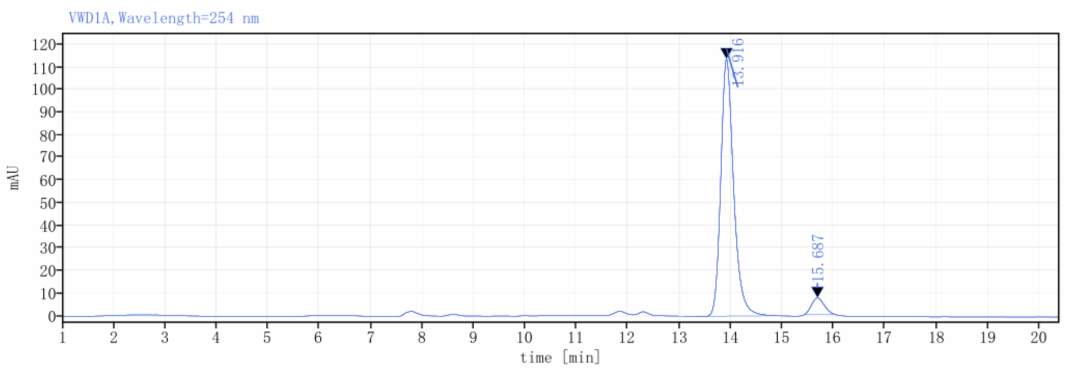

Signal: VWD1A, Wavelength=254 nm

RetTime [min]	Width [min]	Area [mAu*s]	Height [mAu]	Area [%]
13.916	1.52	1984.07	113.61	93.89
15.687	0.62	129.13	7.44	6.11

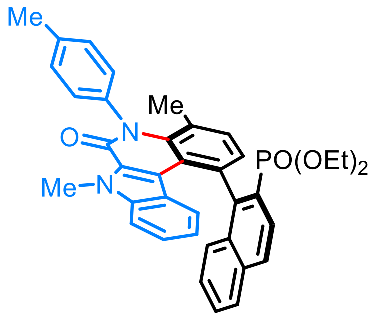

5l

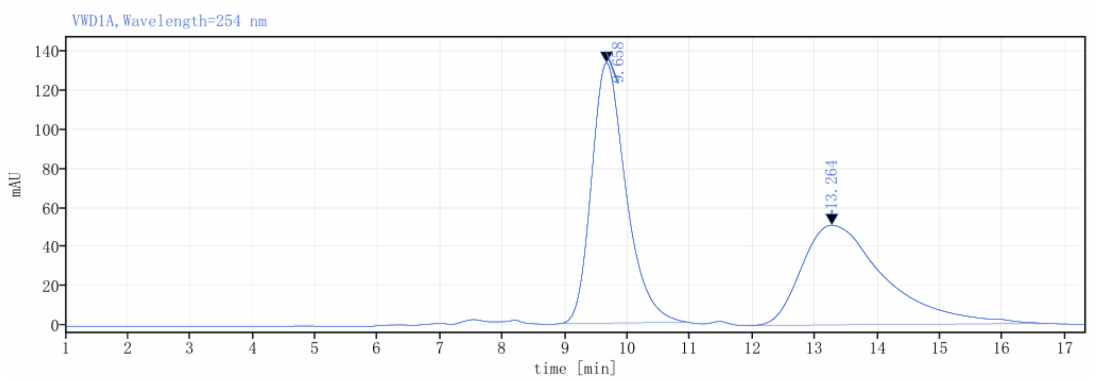

Signal:	VWD1A, Wavelength=254 nm			
RetTime [min]	Width [min]	Area [mAu*s]	Height [mAu]	Area [%]
9.658	2.03	5091.41	132.84	51.92
13.264	4.79	4715.65	50.97	48.08

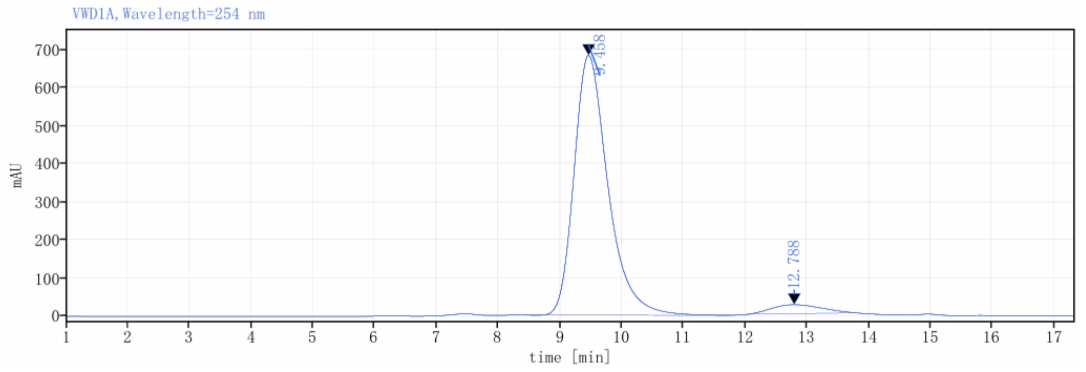

Signal:	VWD1A, Wavelength=254 nm			
RetTime [min]	Width [min]	Area [mAu*s]	Height [mAu]	Area [%]
9.458	3.32	26114.54	683.79	95.20
12.788	1.74	1317.54	23.55	4.80

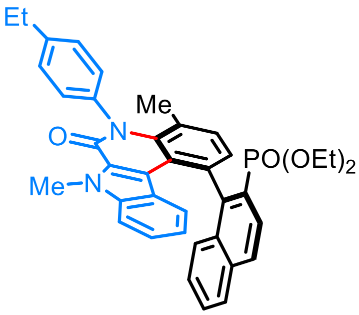

5m

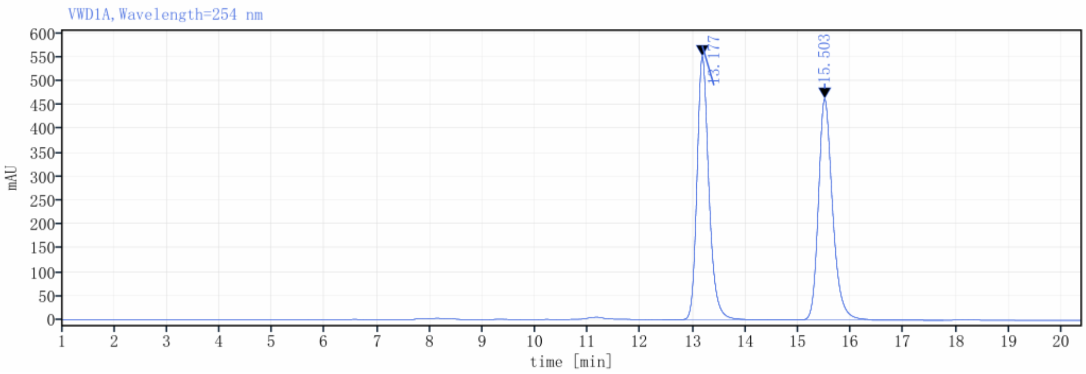

Signal: VWD1A, Wavelength=254 nm

RetTime [min]	Width [min]	Area [mAu*s]	Height [mAu]	Area [%]
13.177	2.49	8729.11	551.98	50.12
15.503	2.84	8685.84	463.18	49.88

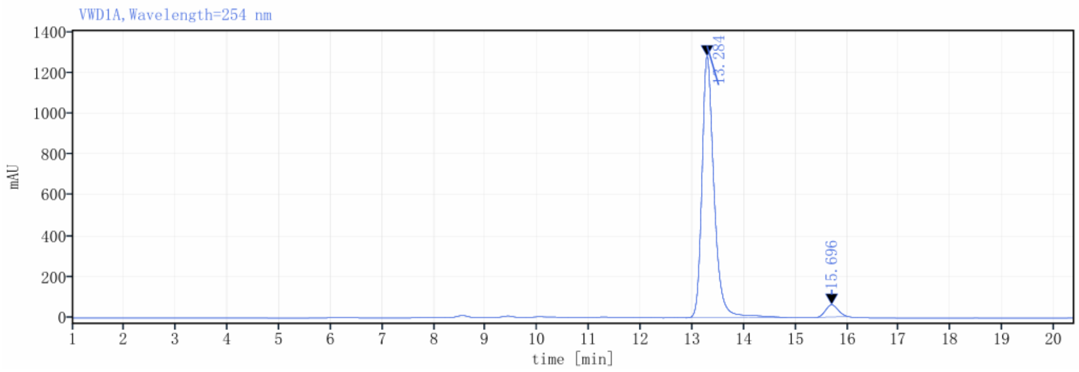

Signal: VWD1A, Wavelength=254 nm

RetTime [min]	Width [min]	Area [mAu*s]	Height [mAu]	Area [%]
13.284	1.96	20859.85	1280.45	95.44
15.696	0.60	996.37	59.27	4.56

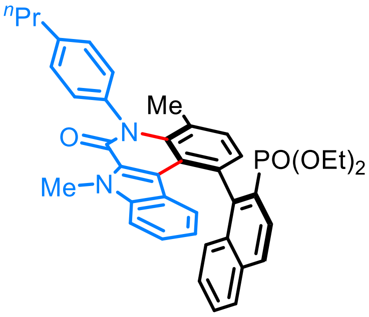

5n

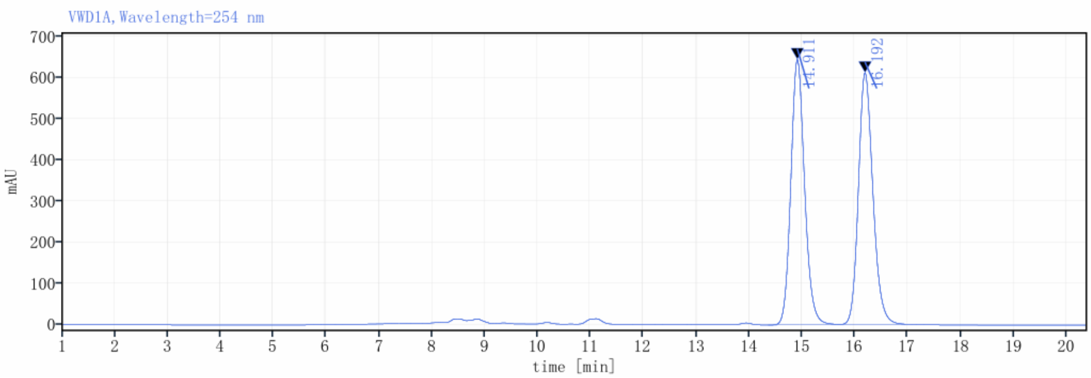

Signal: VWD1A, Wavelength=254 nm

RetTime [min]	Width [min]	Area [mAu*s]	Height [mAu]	Area [%]
14.911	1.27	11634.84	645.58	50.06
16.192	1.32	11606.38	611.88	49.94

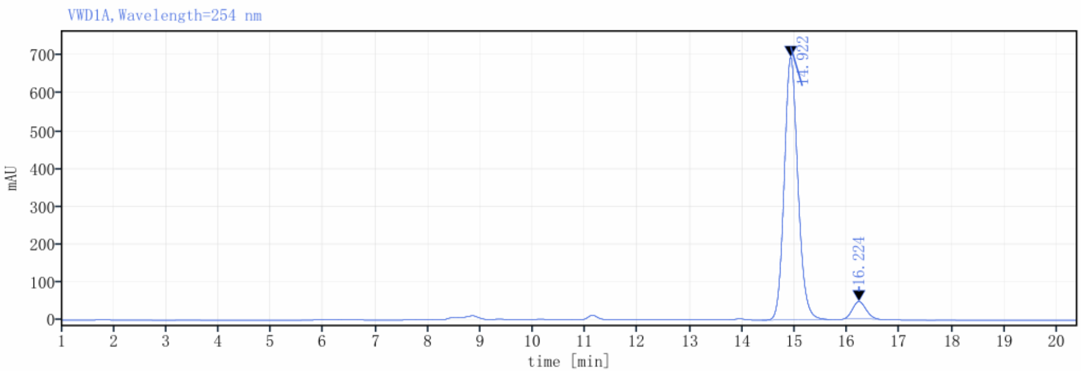

Signal: VWD1A, Wavelength=254 nm

RetTime [min]	Width [min]	Area [mAu*s]	Height [mAu]	Area [%]
14.922	1.29	12582.82	695.29	94.04
16.224	0.65	797.15	45.52	5.96

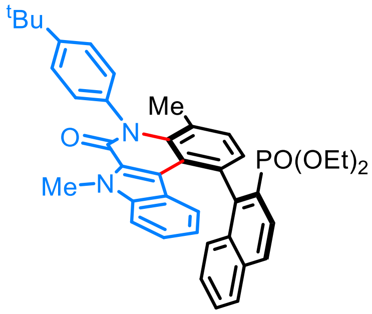

5o

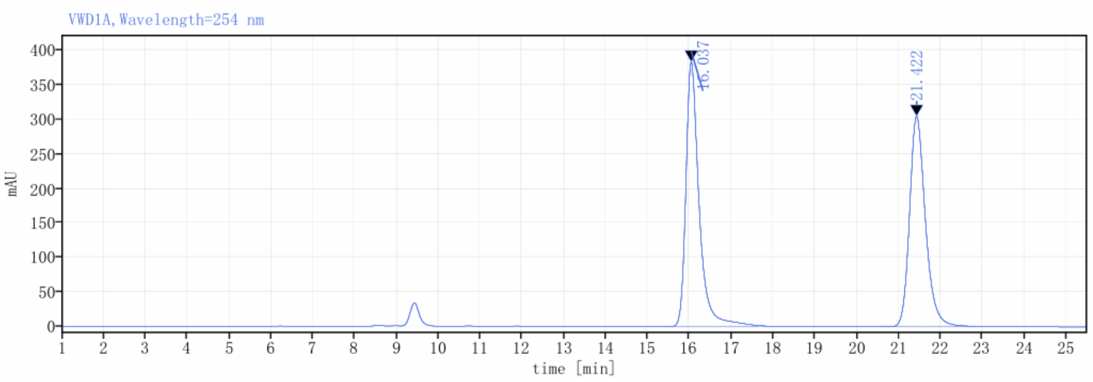

Signal: VWD1A, Wavelength=254 nm

RetTime [min]	Width [min]	Area [mAu*s]	Height [mAu]	Area [%]
16.037	2.38	8442.73	383.84	51.28
21.422	2.25	8020.53	305.23	48.72

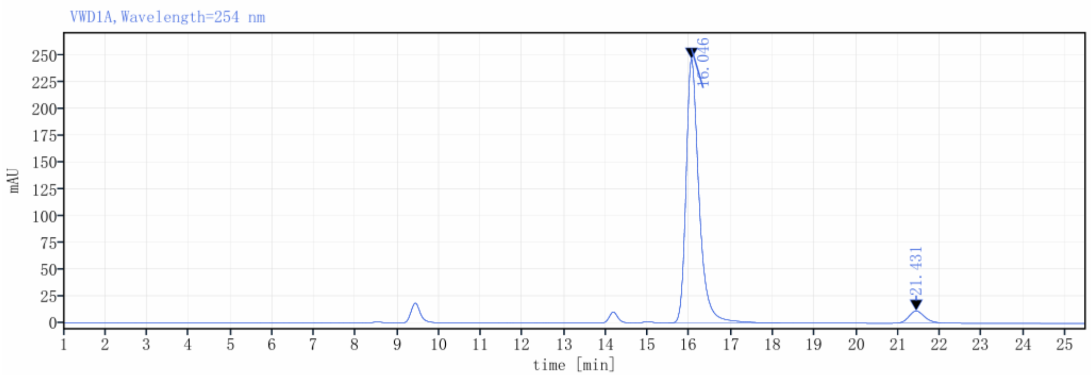

Signal: VWD1A, Wavelength=254 nm

RetTime [min]	Width [min]	Area [mAu*s]	Height [mAu]	Area [%]
16.046	2.68	5354.16	247.18	95.02
21.431	1.02	280.55	11.15	4.98

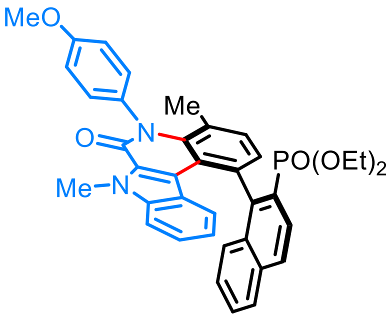

5p

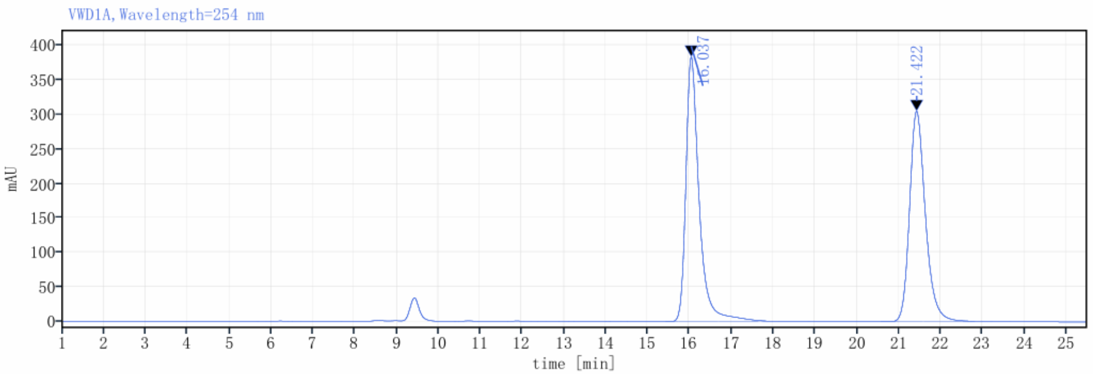

Signal: VWD1A, Wavelength=254 nm

RetTime [min]	Width [min]	Area [mAu*s]	Height [mAu]	Area [%]
16.037	2.38	8442.73	383.84	51.28
21.422	2.25	8020.53	305.23	48.72

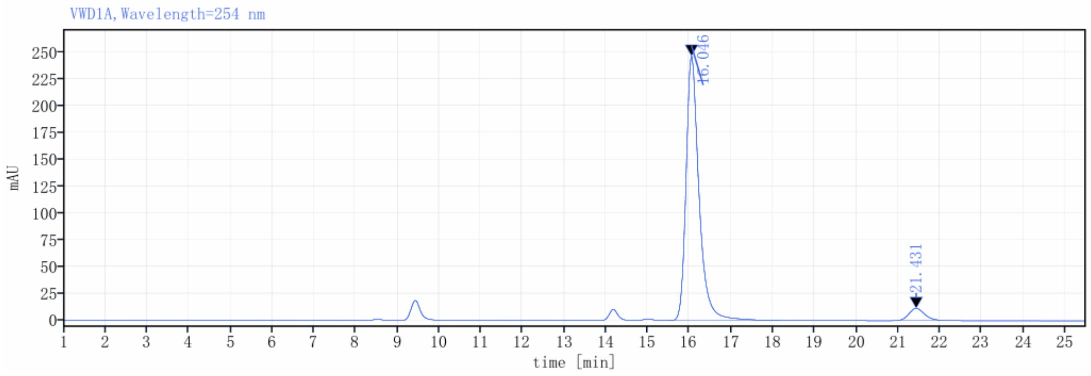

Signal: VWD1A, Wavelength=254 nm

RetTime [min]	Width [min]	Area [mAu*s]	Height [mAu]	Area [%]
16.046	2.68	5354.16	247.18	95.02
21.431	1.02	280.55	11.15	4.98

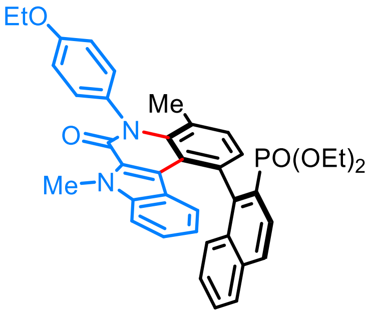

5q

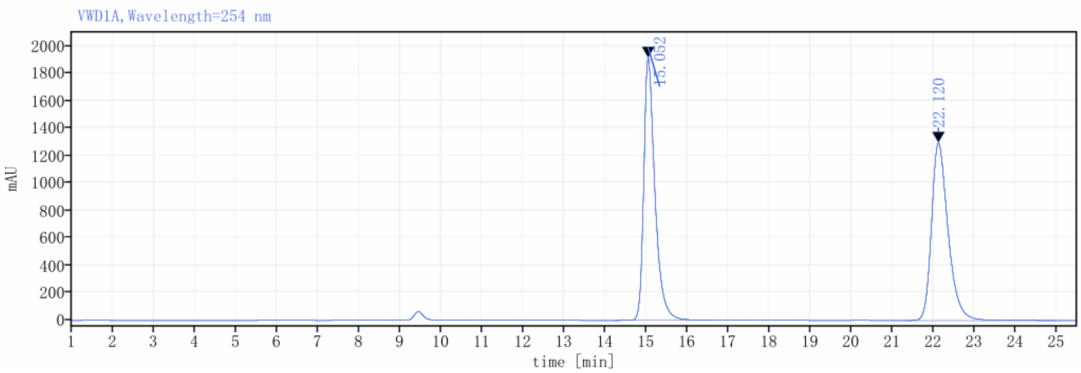

**Signal:**	VWD1A, Wavelength=254 nm			
RetTime [min]	Width [min]	Area [mAu*s]	Height [mAu]	Area [%]
15.052	2.42	36227.38	1911.09	49.99
22.120	3.65	36236.34	1292.99	50.01

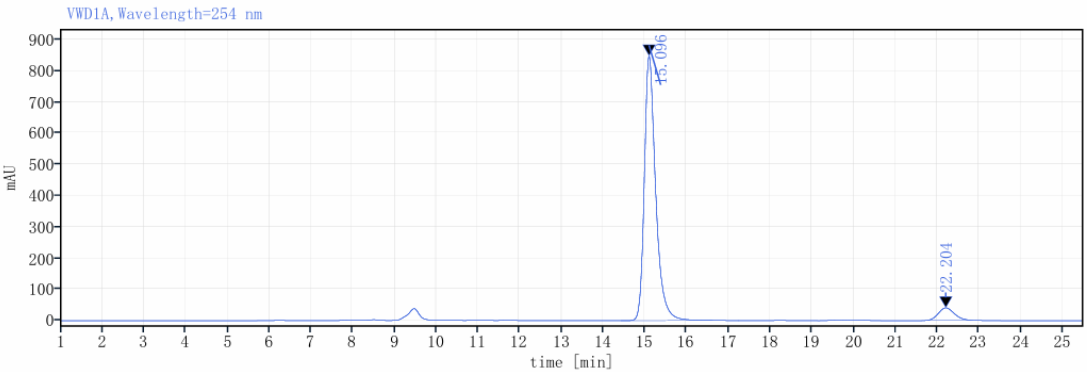

**Signal:**	VWD1A, Wavelength=254 nm			
RetTime [min]	Width [min]	Area [mAu*s]	Height [mAu]	Area [%]
15.096	2.19	16008.98	848.06	93.94
22.204	1.05	1032.26	39.65	6.06

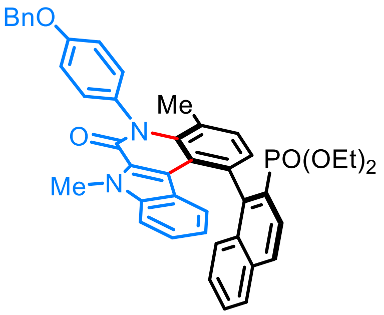

5r

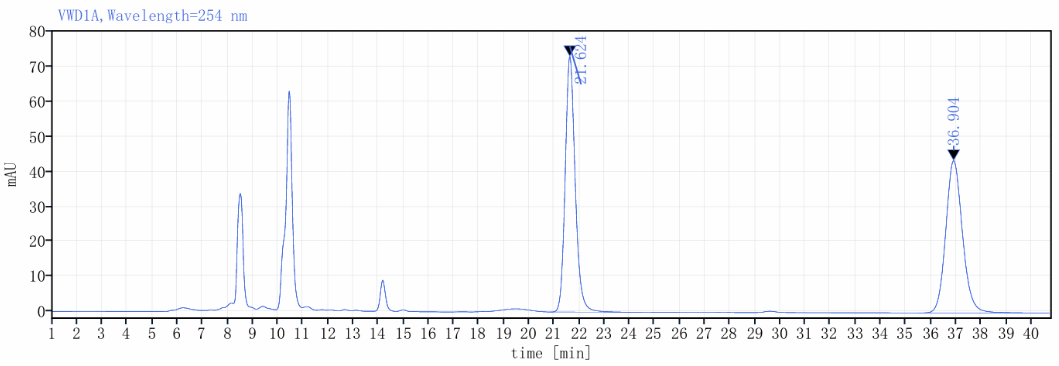

**Signal:**	VWD1A, Wavelength=254 nm			
RetTime [min]	Width [min]	Area [mAu*s]	Height [mAu]	Area [%]
21.624	3.25	2024.99	73.22	50.12
36.904	4.66	2015.38	43.64	49.88

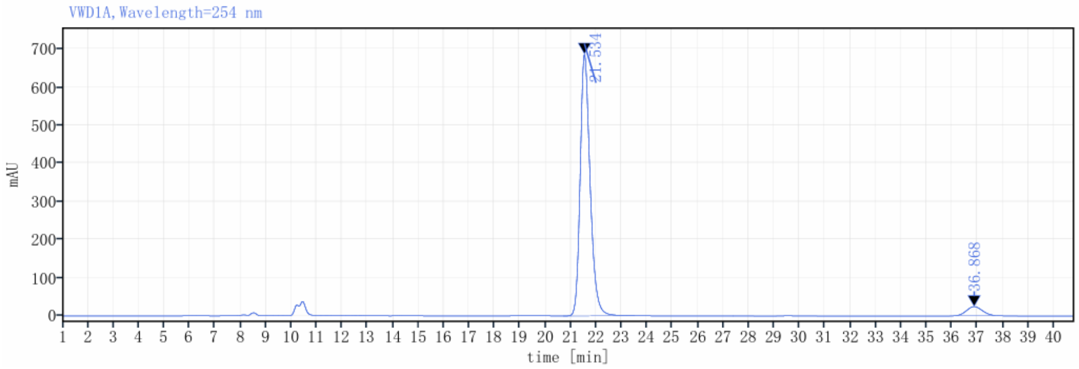

**Signal:**	VWD1A, Wavelength=254 nm			
RetTime [min]	Width [min]	Area [mAu*s]	Height [mAu]	Area [%]
21.534	2.82	18704.43	687.76	94.98
36.868	1.53	988.50	23.53	5.02

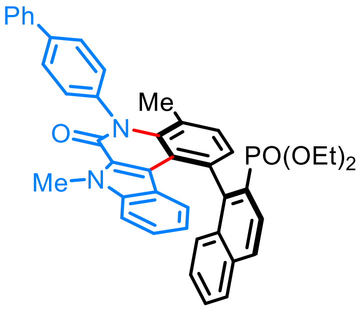

5s

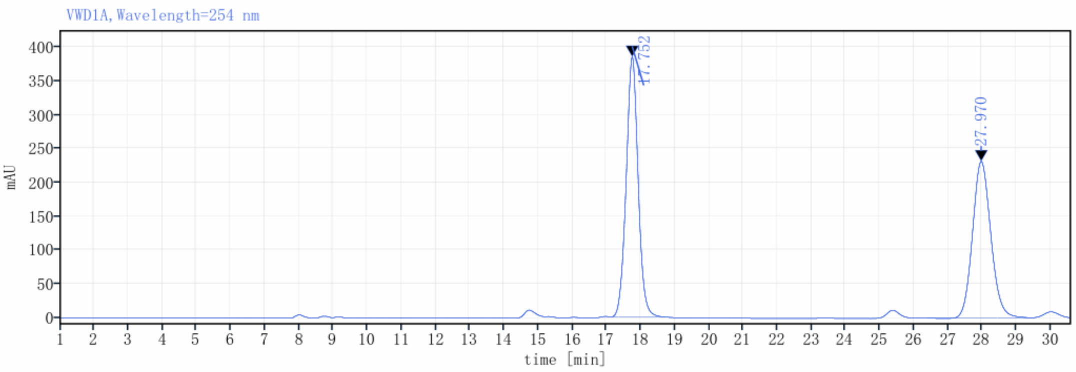

Signal: VWD1A, Wavelength=254 nm

RetTime [min]	Width [min]	Area [mAu*s]	Height [mAu]	Area [%]
17.752	1.53	9318.11	384.17	52.10
27.970	3.12	8565.48	231.44	47.90

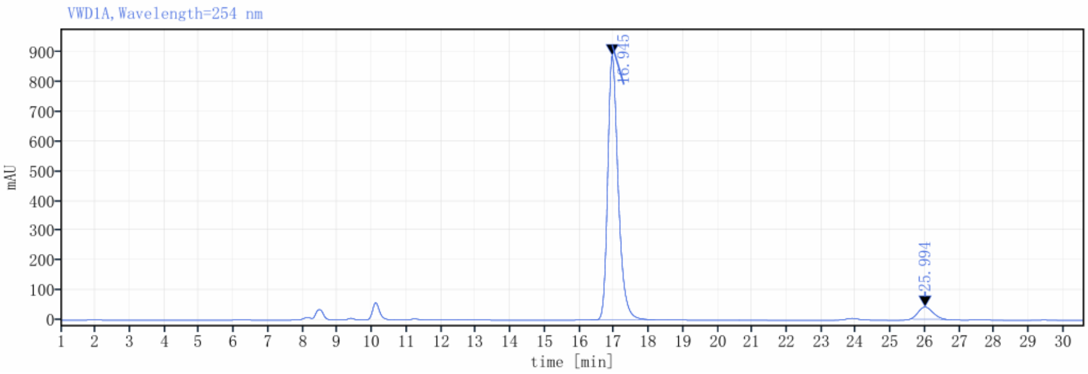

Signal: VWD1A, Wavelength=254 nm

RetTime [min]	Width [min]	Area [mAu*s]	Height [mAu]	Area [%]
16.945	1.87	18730.61	888.96	93.97
25.994	1.05	1202.93	40.99	6.03

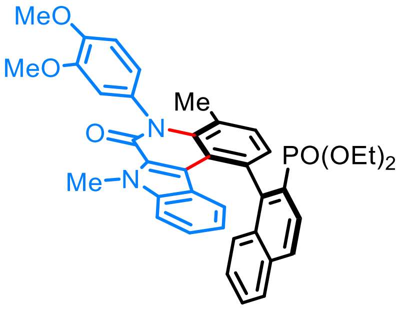

5t

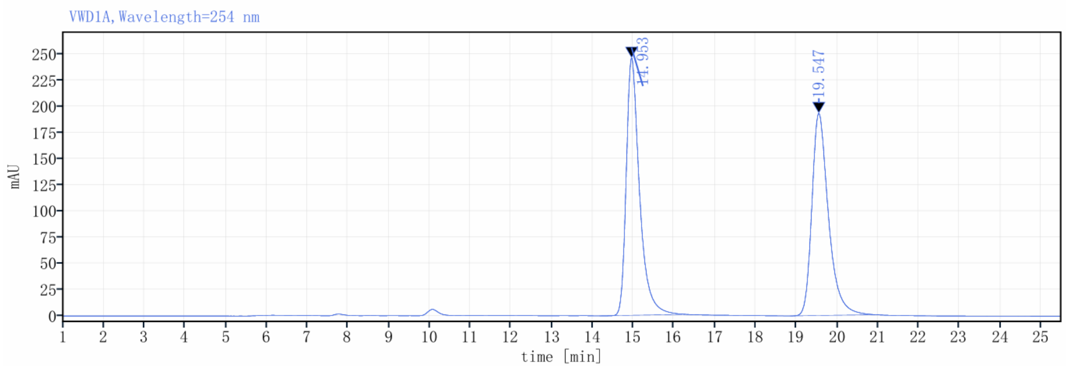

**Signal:**	VWD1A, Wavelength=254 nm			
RetTime [min]	Width [min]	Area [mAu*s]	Height [mAu]	Area [%]
14.953	2.11	5558.29	245.59	50.50
19.547	2.07	5449.24	193.01	49.50

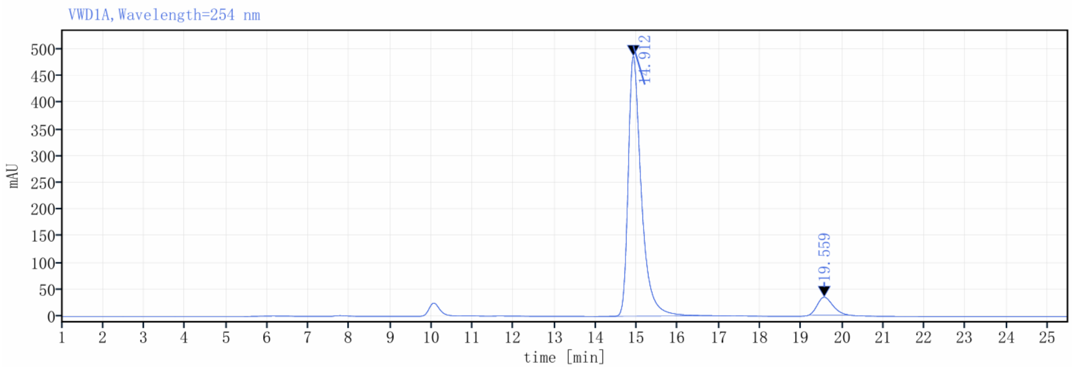

**Signal:**	VWD1A, Wavelength=254 nm			
RetTime [min]	Width [min]	Area [mAu*s]	Height [mAu]	Area [%]
14.912	3.23	10838.26	487.27	92.50
19.559	1.01	878.70	33.60	7.50

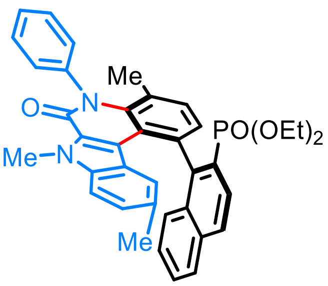

5u

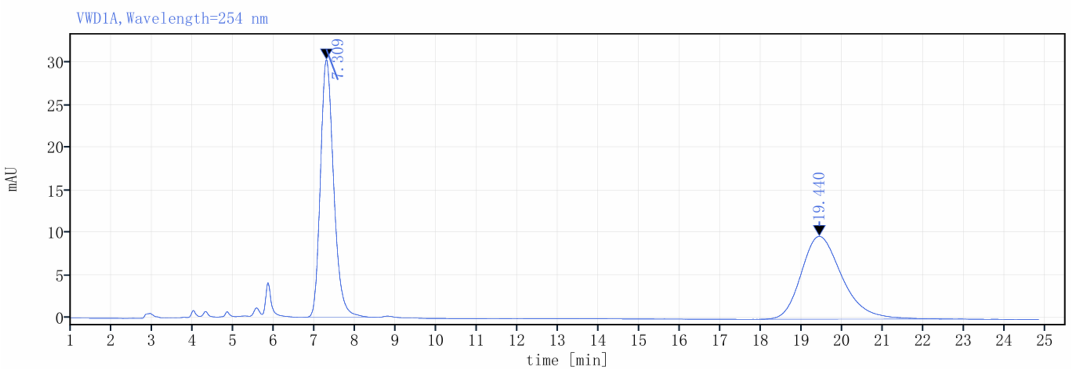

**Signal:**	VWD1A, Wavelength=254 nm			
RetTime [min]	Width [min]	Area [mAu*s]	Height [mAu]	Area [%]
7.309	1.84	688.61	30.36	50.26
19.440	4.44	681.50	9.76	49.74

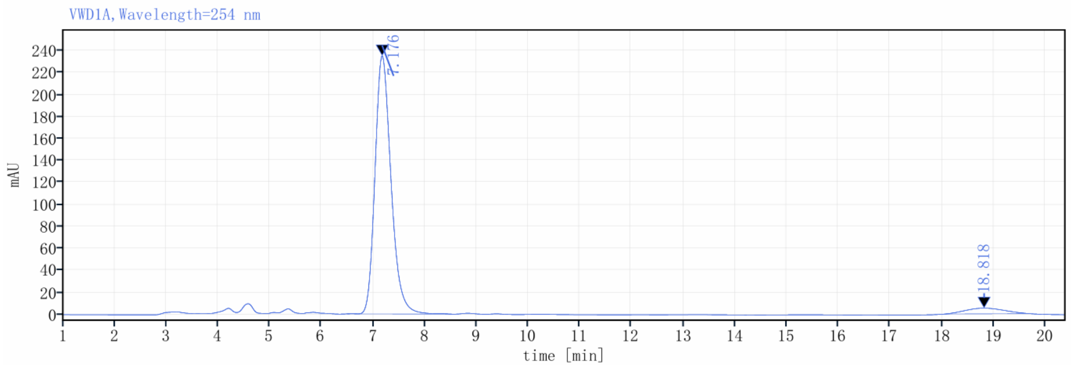

**Signal:**	VWD1A, Wavelength=254 nm			
RetTime [min]	Width [min]	Area [mAu*s]	Height [mAu]	Area [%]
7.176	2.10	5064.38	234.87	94.97
18.818	1.62	267.95	5.15	5.03

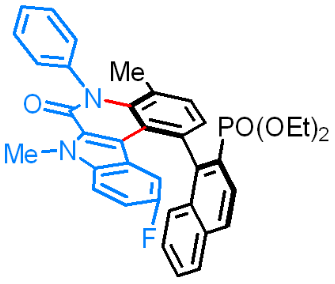

5v

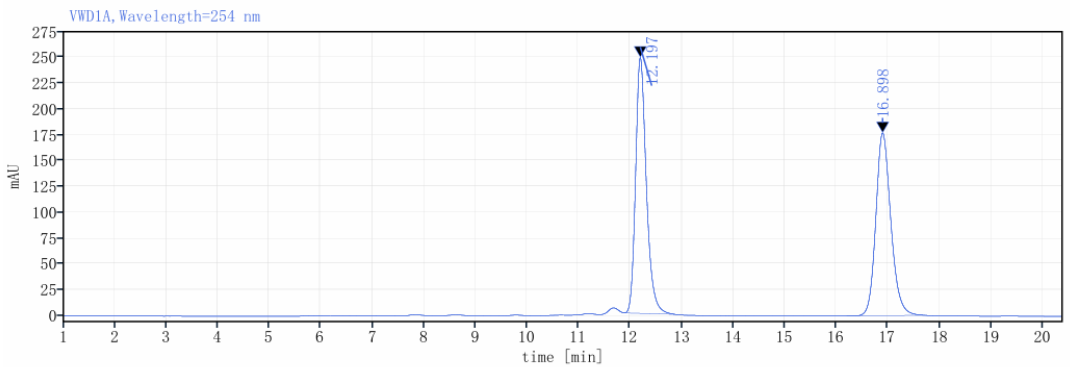

**Signal:**	VWD1A, Wavelength=254 nm			
RetTime [min]	Width [min]	Area [mAu*s]	Height [mAu]	Area [%]
12.197	0.98	3583.55	247.47	50.09
16.898	1.48	3570.09	176.81	49.91

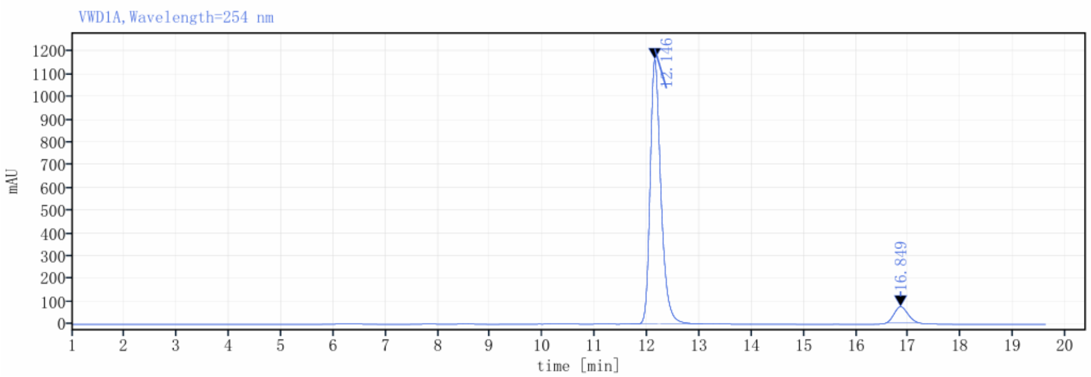

**Signal:**	VWD1A, Wavelength=254 nm			
RetTime [min]	Width [min]	Area [mAu*s]	Height [mAu]	Area [%]
12.146	1.38	16608.90	1163.93	92.96
16.849	0.62	1258.27	70.76	7.04

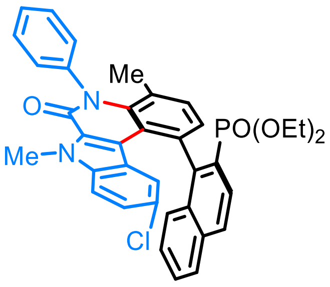

5w

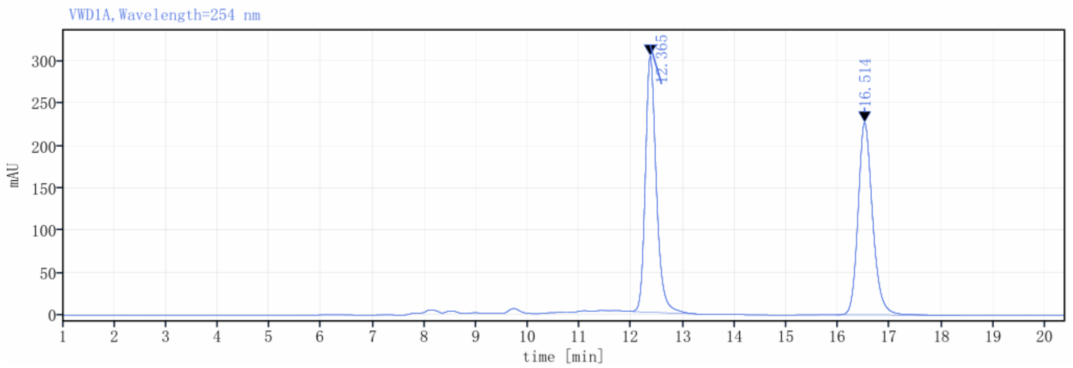

**Signal:**	VWD1A, Wavelength=254 nm			
RetTime [min]	Width [min]	Area [mAu*s]	Height [mAu]	Area [%]
12.365	1.38	4468.60	302.91	50.22
16.514	2.37	4429.84	227.19	49.78

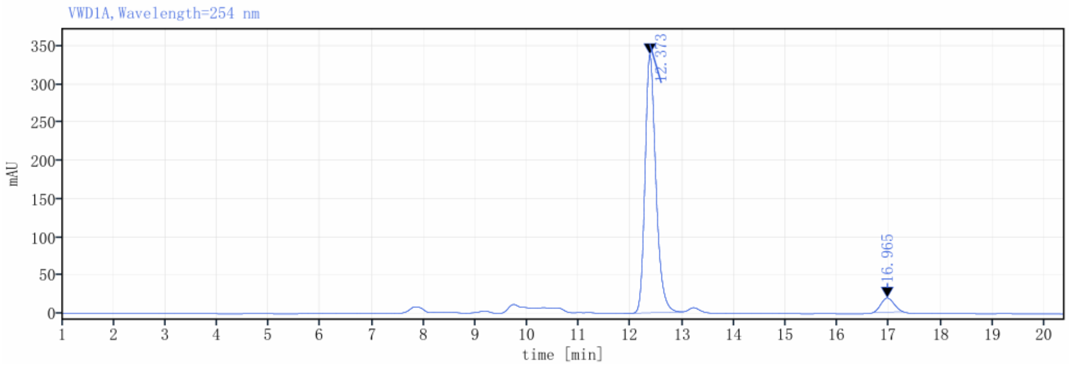

**Signal:**	VWD1A, Wavelength=254 nm			
RetTime [min]	Width [min]	Area [mAu*s]	Height [mAu]	Area [%]
12.373	1.15	4912.23	338.57	93.91
16.965	0.58	318.50	18.39	6.09

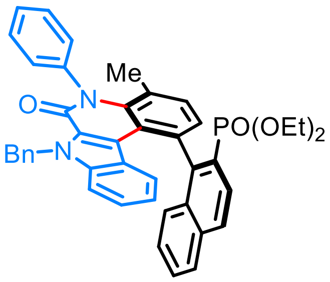

5x

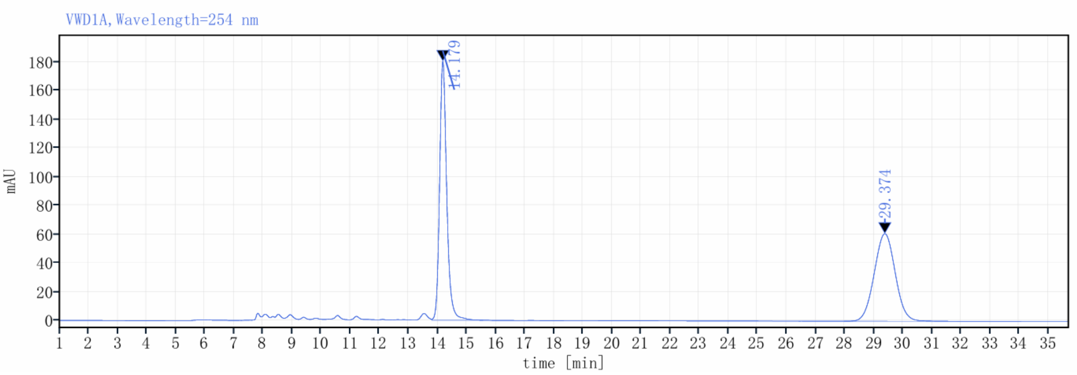

**Signal:**	VWD1A, Wavelength=254 nm			
RetTime [min]	Width [min]	Area [mAu*s]	Height [mAu]	Area [%]
14.179	1.76	3059.76	180.08	50.04
29.374	4.22	3054.33	60.93	49.96

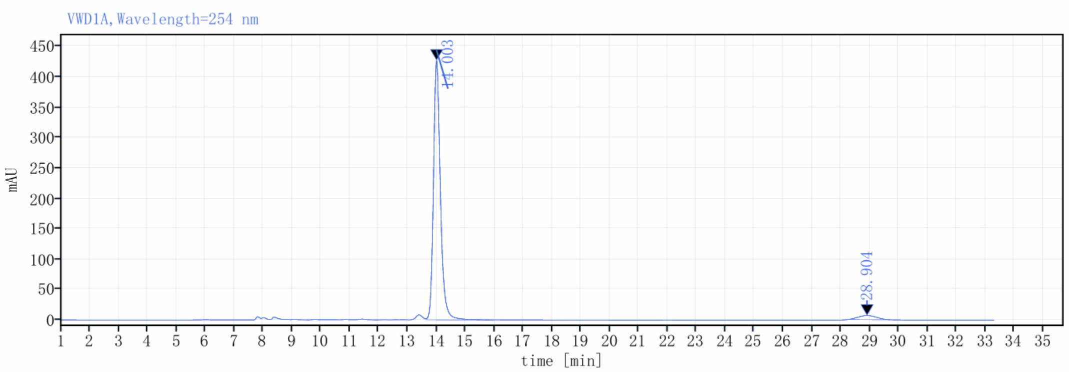

**Signal:**	VWD1A, Wavelength=254 nm			
RetTime [min]	Width [min]	Area [mAu*s]	Height [mAu]	Area [%]
14.003	2.20	7123.49	427.36	96.06
28.904	1.44	292.53	6.69	3.94

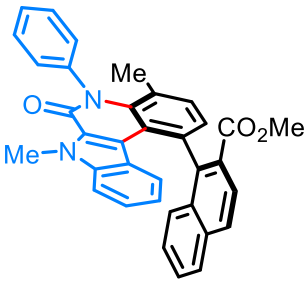

5y

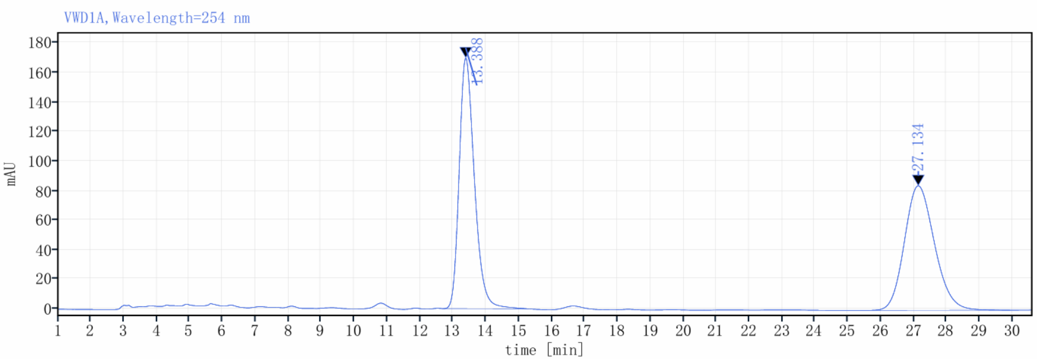

Signal: VWD1A, Wavelength=254 nm

RetTime [min]	Width [min]	Area [mAu*s]	Height [mAu]	Area [%]
13.388	2.58	5457.98	170.00	50.49
27.134	4.55	5351.55	84.42	49.51

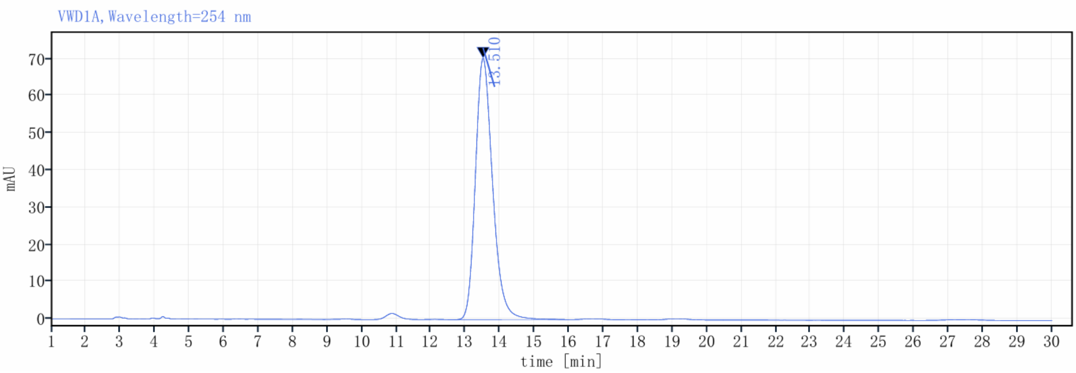

Signal: VWD1A, Wavelength=254 nm

RetTime [min]	Width [min]	Area [mAu*s]	Height [mAu]	Area [%]
13.510	3.35	2439.56	70.34	100.00

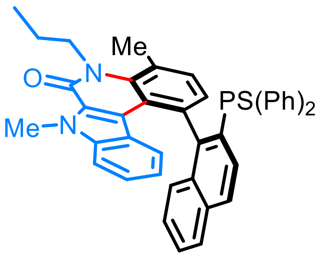

6

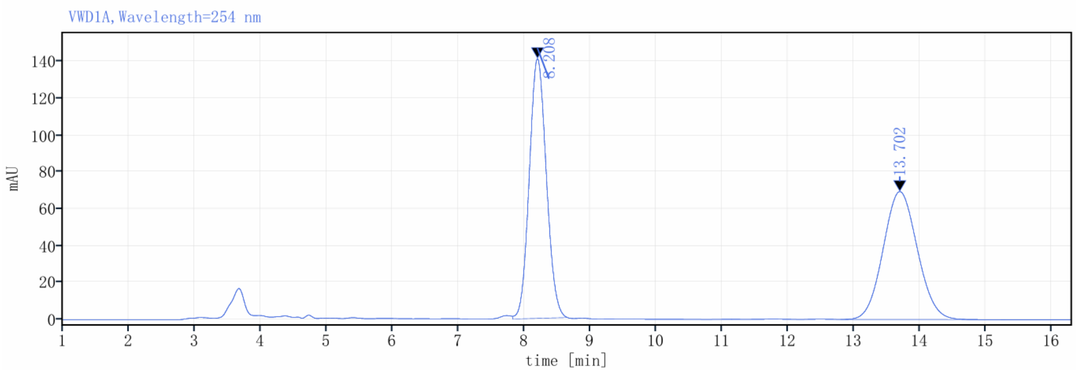

**Signal:**	VWD1A, Wavelength=254 nm			
RetTime [min]	Width [min]	Area [mAu*s]	Height [mAu]	Area [%]
8.208	0.83	2537.82	141.13	50.35
13.702	2.49	2502.15	69.50	49.65

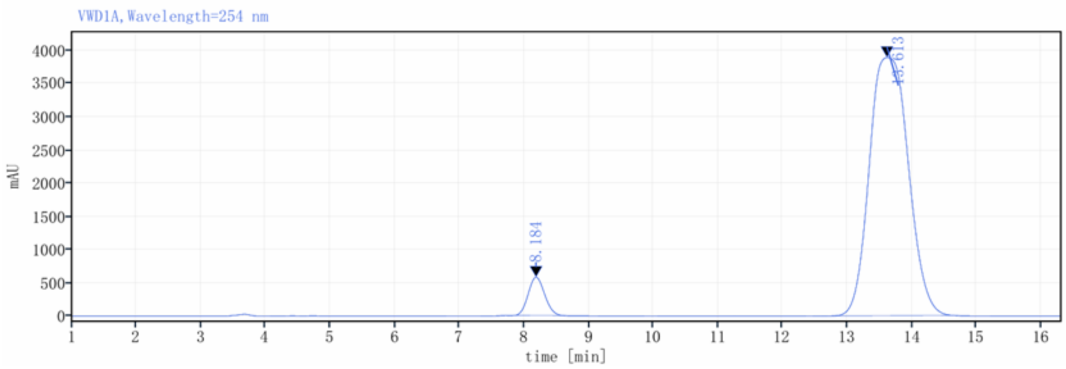

**Signal:**	VWD1A, Wavelength=254 nm			
RetTime [min]	Width [min]	Area [mAu*s]	Height [mAu]	Area [%]
8.184	0.77	10232.30	578.72	5.88
13.613	2.04	163840.45	3894.05	94.12

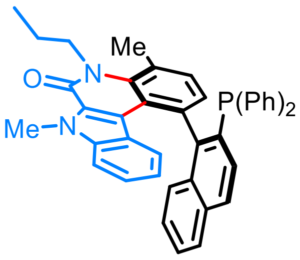

7

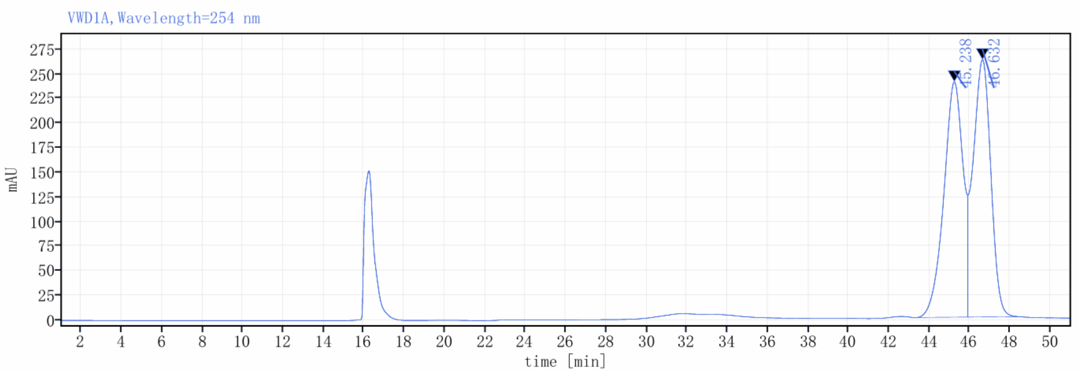

**Signal:**	VWD1A, Wavelength=254 nm			
RetTime [min]	Width [min]	Area [mAu*s]	Height [mAu]	Area [%]
45.238	2.70	15814.47	238.87	49.82
46.632	2.50	15931.86	261.15	50.18

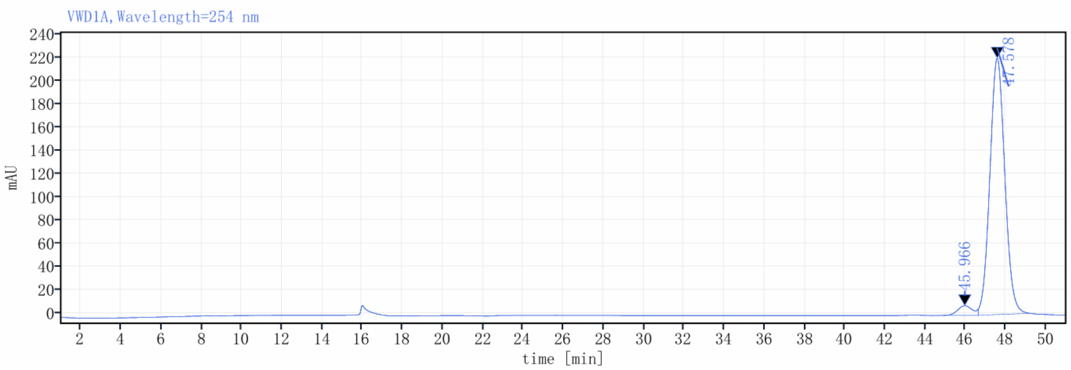

**Signal:**	VWD1A, Wavelength=254 nm			
RetTime [min]	Width [min]	Area [mAu*s]	Height [mAu]	Area [%]
45.966	1.71	429.15	8.27	3.62
47.578	2.56	11421.32	221.25	96.38

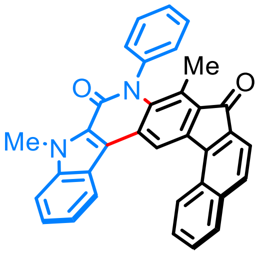

8

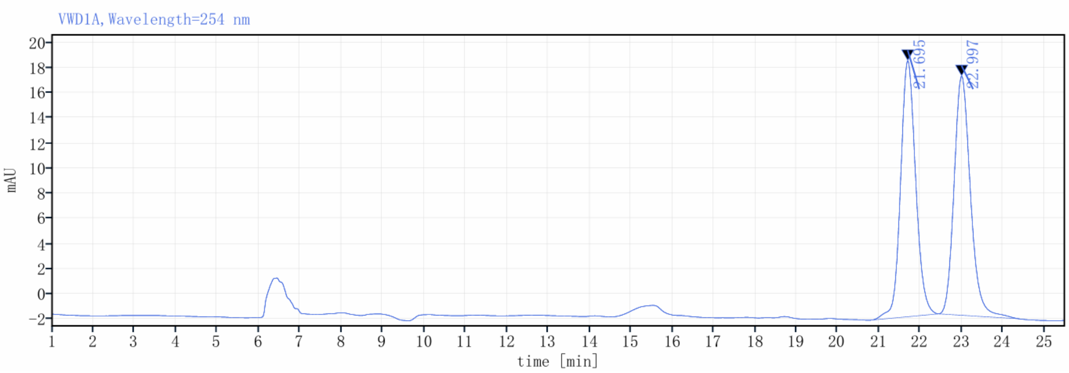

Signal: VWD1A, Wavelength=254 nm

RetTime [min]	Width [min]	Area [mAu*s]	Height [mAu]	Area [%]
21.695	1.65	516.71	20.35	49.67
22.997	1.95	523.56	19.01	50.33

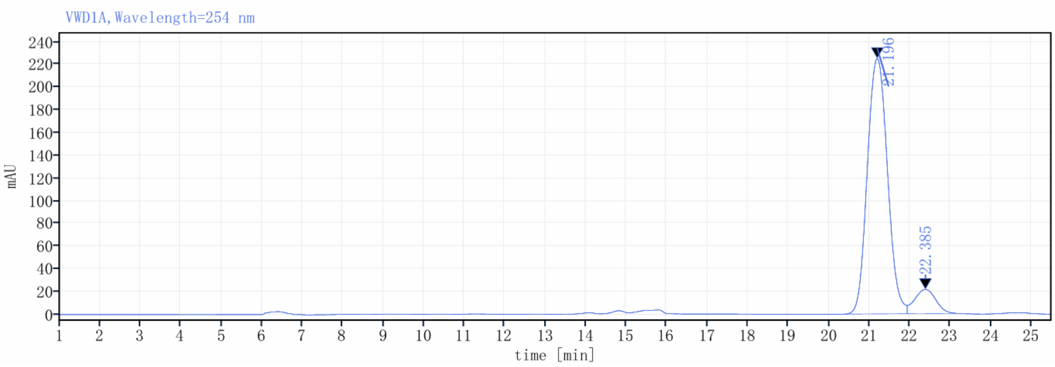

Signal: VWD1A, Wavelength=254 nm

RetTime [min]	Width [min]	Area [mAu*s]	Height [mAu]	Area [%]
21.196	1.54	7763.87	224.97	91.00
22.385	1.19	767.70	21.22	9.00

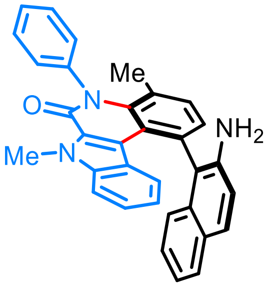

9

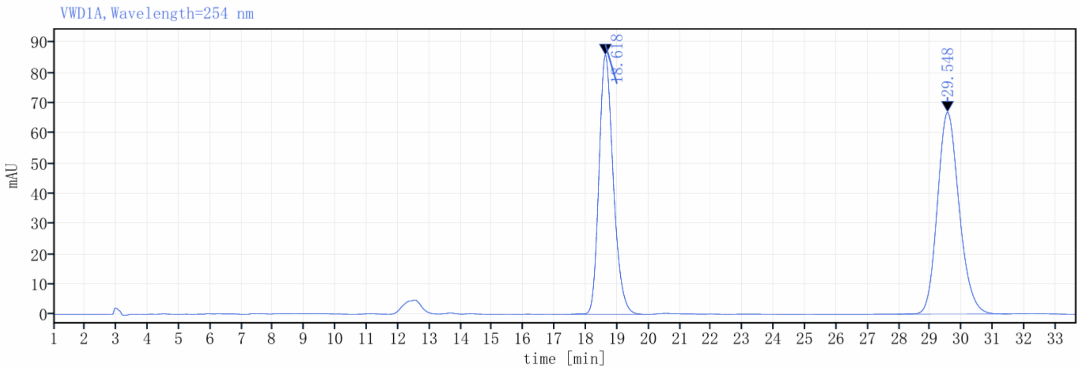

**Signal:**	VWD1A, Wavelength=254 nm			
RetTime [min]	Width [min]	Area [mAu*s]	Height [mAu]	Area [%]
18.618	2.59	2722.78	85.89	45.80
29.548	3.54	3222.29	66.84	54.20

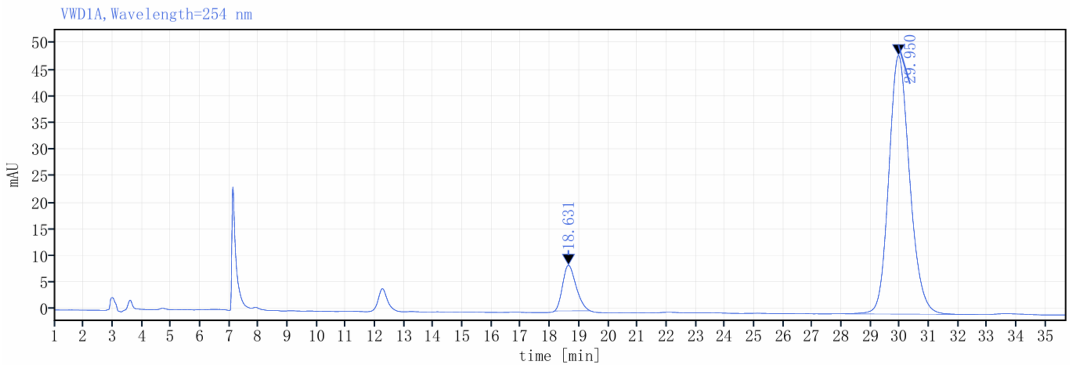

**Signal:**	VWD1A, Wavelength=254 nm			
RetTime [min]	Width [min]	Area [mAu*s]	Height [mAu]	Area [%]
18.631	1.22	273.45	8.57	10.18
29.950	4.69	2413.92	48.55	89.82

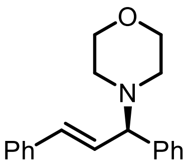

12

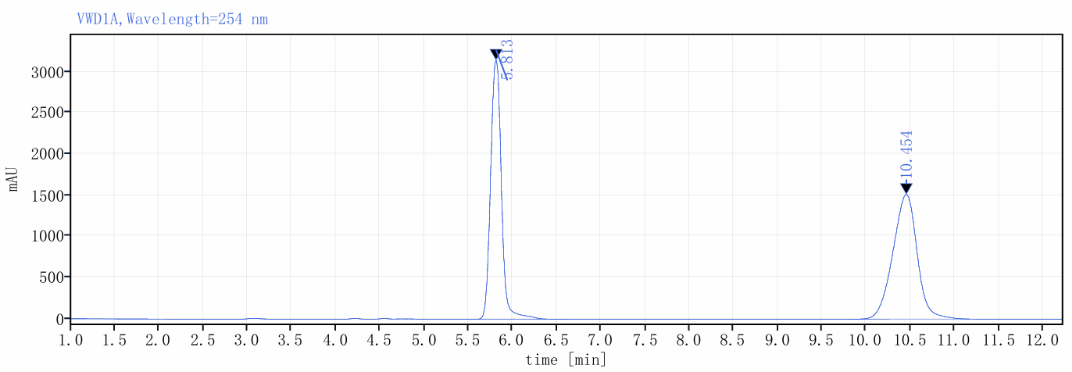

**Signal:**	VWD1A, Wavelength=254 nm			
RetTime [min]	Width [min]	Area [mAu*s]	Height [mAu]	Area [%]
5.813	1.10	26790.91	3150.44	48.05
10.454	1.36	28963.53	1516.39	51.95

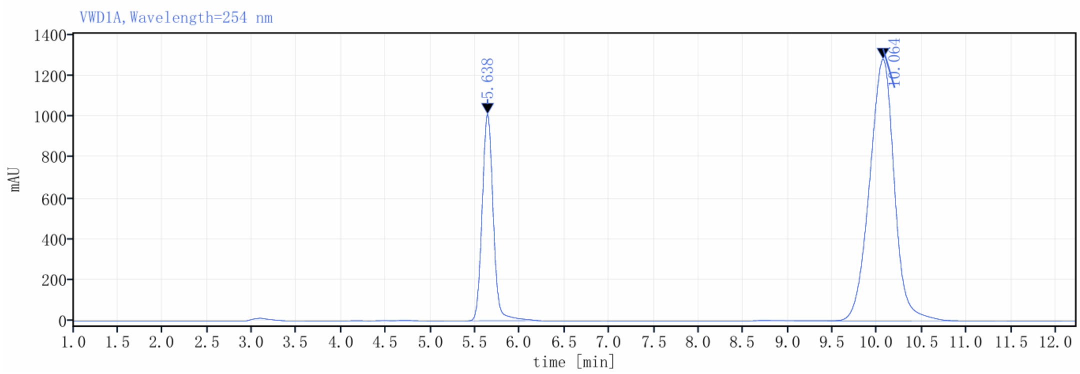

**Signal:**	VWD1A, Wavelength=254 nm			
RetTime [min]	Width [min]	Area [mAu*s]	Height [mAu]	Area [%]
5.638	0.82	8739.16	1012.70	26.13
10.064	1.88	24708.99	1283.50	73.87
